# Supplementary material for: Identifying efficient linkage strategies for men (IDEaL): a study protocol for an individually randomised control trial
Source: BMJ Open. 2023 Jul 12;13(7):e070896. doi: 10.1136/bmjopen-2022-070896 (PMC10347494; doi:10.1136/bmjopen-2022-070896)
Supplement: Supplementary data [file bmjopen-2022-070896supp003.pdf]

IN-DEPTH INTERVIEW FOR MEN – MIDLINE

Section 1: Pre-Trial Experiences

1. At some point in the past, you stopped taking ARV's (or didn't start ART), which is why you were recruited into this study. Can you walk me through some of the reasons that it was difficult to stay on (or start) ART in the past?

Section 2: Trial Experience

1. As a part of your participation in this study, you may have been visited a couple of times by different people. Did anything **bad** happen as a result of these visits?
2. Did someone discover your HIV status as a result of the study or as a result of taking ART since you enrolled?
3. How many times have you met with a health care worker (Patient Supporter/Nurse) about HIV (excluding ART clinic visits)?
    1. *If yes:* Where did you meet this person?
4. When this health care workers (Patient Supporter/Nurse) met with you, you may have gone through this Men's Counseling Flip Chart [SHOW THE FLIP CHART].
    1. Have you ever seen this flip chart?
        - i. IF YES: How was the counseling from this flip chart different from the counseling you have received in the past (before joining the study)?
        - ii. IF YES: What topic/idea from the flip chart was the most helpful to you?
        - iii. IF YES: You described to me earlier that you have missed ART in the past because of [X] reason. Did the new counseling with this flip chart help you deal with [X]? How?
        - iv. IF NO: When you met with the health care worker, can you briefly describe what you discussed?
5. As a part of the study. you may have been offered to be given ART in the home/community.
    1. Did you decide to get ART at home/in the community? *If NO: Skip to Question 6.*
    2. Why did you make the choice you made?
    3. Usually health care workers don't give people a choice of what service you get. But for you, you were given the option between home or facility. How did having this choice (having a say) make you feel?
6. How do you currently get ARTs?
    1. IF at HOME/COMMUNITY: How many times have you gotten your medication at home?
    2. If at the FACILITY: When you went back to the facility, did the did a health care worker/patient supporter offer you any other services?
        - i. What did they do?
        - ii. Was this helpful for you? Why/why not?
7. Has either HCW been supporting you or chatting with you about anything else in addition to HIV or ART?
    1. Do you ever contact either HCW directly to chat or ask questions?
        - i. IF YES: How often do you talk with them? What do you talk about?
    2. Is your relationship with either HCW different to the ones you have had with other HCWs before joing the study? How?
        - i. IF YES: Do you think this relationship helps you more than your relationship with other HCWs? IF YES: How?
8. Now think about overall your interactions with the health care workers you interacted with since you started the study – the Patient Supporter and the Nurse who brought you ART. What did you like about your intereactions with them? How do you think it helps you with ART?

9. With everything in life, there are some things we like and some things that could be a little better. What did you NOT like about your interactions with the Patient Supporter and the Nurse who brought you ART?
10. Is there anything else you think you need a health care worker or the health facility could do differently in order for you to be comfortable taking ART regularly? Is there anything else you need?

Section 3: Initiation
Now I'd like to ask you about what has happened with your ARVs since you were enrolled in the study.

11. Did you start taking ART (again) since enrolling in the study?
    If YES:
    a. Why did you (re) initiate ART? What convinced you it was good to take medication (again)?
    b. Who was most influential in your decision to start ART (again)?
    If NO:
    c. Why have you not initiated ART since enrolling in the study?
    d. Has anyone influenced you to not start ART? Who? Why?
    e. Is there anything that could motivate/help you to initiate ART?
12. We know that starting ART can be difficult. What do you think is the most difficult thing about re-starting ART for you
13. Was there anything that made it easy for you to (re)start ART? (opposite of probes below)
    1. For those who opted for HOME-BASED: Do you think getting ART at home helped you re-engage in care? Why/why not?
14. For the next set of questions please feel free to be honest. There is no right or wrong answer.
    1. Do you feel MORE confident you can stay on ARTs in the future? Why/why not?
    2. Is there someone in your life now that encourages you?
    3. Do you have a better relationship with HCWs / or trust them more? Why/why not?
    4. Do you have a plan so that you don't run out of ARVs if youre away or busy?
    5. Do you think ARVs help you reach your goals for making money and for your family? Why/why not?

IN-DEPTH INTERVIEW FOR MEN – ENDLINE

Section 1: Pre-Trial Experiences

1. Think about when you stopped taking ART **before** joining the study. Can you remember why you stopped swallowing ART pills?
2. Talk to me about some things that made it difficult for YOU to stay on (or start) ART BEFORE joining the study?

Section 2: Post-Trial Experiences

3. As a part of the study, you received [ONLY MENTION THE SERVICE THAT ALIGNS WITH THE CLIENTS STUDY ARM: counseling, mentorship, counseling + home-based 1 month, counseling + home-based 3 months].
    a. How did you feel about this service?
    b. Was this helpful to you? How?
4. Since you enrolled in the study did you restart ARVS?
    a. IF NEVER RE-INITIATED ART IN THE STUDY: Why have you not re-started ART since joining the study?

5. Think about the challenges you experienced BEFORE joining the study. [REFER BACK TO THE CHALLENGES MENTIONED IN QUESTION 1 and 2]. Have these challenges gone away or been reduced since you joined the study?
   a. How?
   b. Why has it changed/not changed?

6. Have you had any **new or different** challenges since joining the study (either to attend refill appointments or taking your ART)? What are they?
   a. Why do you think you experience them now?

7. Are there things about attending refill appointments or swallowing ART that are now easier since you joined the study?
   a. What are they? Why?

8. Think about what you discussed with the study HCW who met with you [he may or may not have used this flip chart]. When he chatted with you, he might have told you some things that were **new** to you about how to keep taking ARTs. Have you tried doing any of those things AFTER you met with the HCW?
   a. What was it?
   b. Did it help you? How?
   c. If it did not help you, what challenges did you face?

9. Have you continued to talk to the study HCW since you first met with him?
   a. Describe your relationship.
      i. How frequently do you connect? In person, on the phone, both?
      ii. What do you discuss?
      iii. How is this relationship helpful for you and your life?
      iv. Do you wish for your relationship with the HCW to continue [or continued in the case of the HCW being fired]? Why? Why not?

10. [GENERAL NOT JUST STUDY HCWS] What additional services/interventions [not materials/monitary] do you wish you could have to help you consistently access or swallow ART?
   a. Would you need this for a short period of time (one time, or for a month) or would you need it ALL the time (for years)? Why?

11. Do you have other health concerns outside of HIV? What are they?
   a. Is it easier or harder for you to get care for these concerns as compared to getting ART services? Why?

Section 3: Repeat Treatment Interruption [ONLY FOR THOSE WHO REINTIATED]
Now I want to talk to you about your use of ART services since joining the study.

12. Have you missed an ART appointment or missed swallowing your medication on any day since you restarted ART?

If **NO:**

13. How have you managed to stay on ART? What strategies do you think are most useful to help you stay on ART? Are they different from the one’s you mentioned before? Does someone or something specifically help you?

If **YES:**

14. Can you tell me what happened?
   a. For how long did you stop taking ART?
   b. How many times has it happened?
   c. Why did you miss ART appointment/swallowing ART?

15. What do you think would make it easier for men like you to not miss doses of ART or ART appointments?

16. Have you started taking ARVS **again** after you missed doses or gone back to the facility after you missed a refill appointment?

If **NO** – skip to question 18

If **YES**

17. Did anything made it easy for you to (re)start ART AGAIN?

18. We know that restarting ART can be difficult. What fears/worries/concerns did you have to overcome in order to restart (again)?

19. What would make it easier for men like you to come back to the clinic after missing an appointment?

Section 4: Client centered care choice experiment

20. We know there is no such thing as a 'perfect' healthcare worker. We are all human. But today we want to give you the opportunity to think about key things you want if you had the perfect interaction with a health care worker. Here is a picture of a health care worker. Around him we have characteristics on different cards [McDaphton to read through the cards]

1. He treats me with respect
2. His counseling message is easy to understand
3. His counseling addresses my specific concerns
4. He maintains privacy/keeps my secrets
5. He keeps in contact with me (not just a one time counseling)
6. He comes to the community to find me
7. He treats me like family and cares for me as a person
8. He asks me questions about my life/circumstances
9. He takes his time with me (the counseling session is not rushed)
10. He shares his own experiences openly as a fellow man
11. He provides fast services

21. Id like you to choose the 6 most important characteristics you think are essential for how you want to interact with health care workers. There is no right or wrong answers. This is about what matters to you.

a. Why have you chosen these? What makes them very important to you?

22. Now you can only choose 3 of these characteristics. Look at your 6 characteristics in front of you. What THREE are the most important/essential characteristics for your health care worker to still provide you with the services you would like.

a. Why have you chosen these? Why did you NOT choose the other three?

Section 5: Income

23. What things do you do to earn a living? Think about all the work you do – this may include several different things.

a. How do you earn money (what kind of work do you do)?
b. Where do you work?
c. Do you travel for work?
d. How often do you work?
e. How do you usually find this work? How do employers/bosses or customers find you or how do you find markets for your products?

24. What would happen to you and/or your family if you spent a whole day at the clinic and did not earn any money that day?

a. Is it possible for you to attend your ART appoint and not miss out on any income/money? How is this possible for you?
b. In the WORST case scenario: how much missed opportunities to earn money would you experience? What would be the biggest impact on your financial situation be if you attend a clinic appointment and miss work/work opportunities (exp: do you miss a client, did your shop not open, did you miss the chance to get a long term job?)
c. Does missing a day from making money impact your family? How?
