# Supplementary material for: Identifying efficient linkage strategies for men (IDEaL): a study protocol for an individually randomised control trial
Source: BMJ Open. 2023 Jul 12;13(7):e070896. doi: 10.1136/bmjopen-2022-070896 (PMC10347494; doi:10.1136/bmjopen-2022-070896)
Supplement: Author's manuscript [file bmjopen-2022-070896.draft_revisions.pdf]

BMJ Open

BMJ Open is committed to open peer review. As part of this commitment we make the peer review history of every article we publish publicly available.

When an article is published we post the peer reviewers' comments and the authors' responses online. We also post the versions of the paper that were used during peer review. These are the versions that the peer review comments apply to.

The versions of the paper that follow are the versions that were submitted during the peer review process. They are not the versions of record or the final published versions. They should not be cited or distributed as the published version of this manuscript.

BMJ Open is an open access journal and the full, final, typeset and author-corrected version of record of the manuscript is available on our site with no access controls, subscription charges or pay-per-view fees (http://bmjopen.bmj.com).

If you have any questions on BMJ Open's open peer review process please email info.bmjopen@bmj.com

BMJ Open

Identifying efficient linkage strategies for HIV self-testing (IDEaL): a study protocol for an individually randomized control trial

Journal:	*BMJ Open*
Manuscript ID	bmjopen-2022-070896
Article Type:	Protocol
Date Submitted by the Author:	13-Dec-2022

Complete List of Authors:	Dovel , Kathryn ; University of California Los Angeles, Division of Infectious Diseases ; Partners in Hope Medical Center, Implementation Science Department<br>Balakasi, Kelvin; Partners in Hope Medical Center, Implementation Science Department<br>Hubbard, Julie; University of California Los Angeles, Division of Infectious Diseases ; Partners in Hope Medical Center, Implementation Science Department<br>Phiri, Khumbo; Partners in Hope Medical Center, Implementation Science Department<br>Nichols, Brooke ; Boston University School of Public Health, Department of Global Health; Faculty of Health Sciences University of the Witwatersrand, Department of Internal Medicine<br>Coates, Thomas; University of California Los Angeles, Division of Infectious Diseases<br>Kulich, Michal; Charles University, Department of Probability and Math Statistics<br>Chikuse, Elijah; Partners in Hope Medical Center, Implementation Science Department<br>Phiri, Sam; Partners in Hope Medical Center, Implementation Science Department<br>Long, Lawrence; Boston University School of Public Health, Department of Global Health; Faculty of Health Sciences University of the Witwatersrand, Department of Internal Medicine<br>Hoffman, Risa; University of California Los Angeles, Division of Infectious Diseases<br>Choko, Augustine; University of Malawi, College of Medicine

Keywords:	HIV & AIDS < INFECTIOUS DISEASES, Infection control < INFECTIOUS DISEASES, Clinical trials < THERAPEUTICS

SCHOLARONE™
Manuscripts

Identifying efficient linkage strategies for HIV self-testing (IDEaL): a study protocol for an individually randomized control trial

Kathryn Dovel[1,2§], Kelvin Balakasi[1], Julie Hubbard[1], Khumbo Phiri[1], Brooke E. Nichols[3,4], Thomas J. Coates[1], Michal Kulich[5], Elijah Chikuse[2], Sam Phiri[2], Lawrence Long[3,4], Risa Hoffman[1], Augustine Choko[6].

Author affiliations:

[1] Division of Infectious Diseases, David Geffen School of Medicine, University of California Los Angeles, Los Angeles, CA, USA
[2] Partners in Hope, Lilongwe, Malawi
[3] Department of Global Health, Boston University School of Public Health, Boston, MA, USA
[4] Department of Internal Medicine, Faculty of Health Sciences, University of the Witwatersrand, Johannesburg, South Africa
[5] Department of Probability and Math. Statistics, Faculty of Mathematics and Physics, Charles University, Prague, Czech Republic
[6] College of Medicine, University of Malawi, Blantyre, Malawi

[§] **Corresponding Author:** Kathryn Dovel
Division of Infectious Diseases, David Geffen School of Medicine
10833 Le Conte Ave, Los Angeles, CA 90095
Phone: +13108838179
Email: kdovel@mednet.ucla.edu / kldovel@gmail.com

E-mail addresses of authors:

KD: KDovel@mednet.ucla.edu
KB: kelvin@pihmalawi.com
JH: jhubbard@mednet.ucla.edu
KP: khumbo@pihmalawi.com
BN: brooken@bu.edu
TC: TCoates@mednet.ucla.edu
MK: kulich@karlin.mff.cuni.cz
EC: elijah@pihmalawi.com
SP: samphiri@pihmalawi.com
LL: lclong@bu.edu
RH: RHoffman@mednet.ucla.edu
AC: augutc@gmail.com

Word Count: Abstract: 252; Manuscript: 3,967

Trial registration number: NCT05137210

For peer review only

Keywords: HIV, men, ART initiation, ART retention, Stepped interventions, sub-Saharan Africa

Date and version: 30 November, 2022: Version 1

ABSTRACT

Introduction

Men in sub-Saharan Africa are less likely than women to initiate antiretroviral therapy (ART) and are more likely to have longer cycles of disengagement from ART programs. Treatment interventions that meet the unique needs of men are needed, but they must be scalable. We will conduct a study to test the impact of various interventions on six-month retention in ART programs among men living with HIV who are not currently engaged in care.

Methods and Analysis

We will conduct a programmatic, individually randomized, non-blinded, non-inferiority controlled trial. "Non-engaged" men will be randomized 1:1:1 to either a Stepped, Low-Intensity, or High-Intensity arm. In the Stepped arm, intervention activities build in intensity over time for those who do not reengage in care with the following steps: 1) one-time male-specific counseling + facility navigation → 2) ongoing male mentorship + facility navigation → 3) outside-facility ART initiation + male-specific counseling + facility navigation for follow-up ART visits. The Low-Intensity Intervention includes one-time male-specific counseling + facility navigation only and the High-Intensity Intervention offers immediate outside-facility ART initiation + male-specific counseling + facility navigation for follow-up ART visits. Our primary outcome is 6-month retention in care. Secondary outcomes include cost-effectiveness and rates of adverse events.

Ethics and Dissemination

The Institutional Review Board of the University of California, Los Angeles and the National Health Sciences Research Council in Malawi have approved the trial protocol. Findings will be disseminated rapidly in national and international forums, as well as in peer-reviewed journals and are expected to provide urgently needed information to other countries and donors.

ARTICLE SUMMARY

For peer review only - http://bmjopen.bmj.com/site/about/guidelines.xhtml

Strengths and limitations

- IDEAL provides male-specific differentiated models of care aimed to improve men's ART outcomes. We specifically focus on building trusting relationships with health care workers and developing client-led, individualized strategies to overcome barriers to care.
- IDEAL will test the impact of a stepped intervention for men. This approach promises to improve the efficiency and reach of HIV programs for men as the highest-resource interventions will only be received by the minority of men who are most in need.
- IDEAL develops and tests male-specific counseling curriculum that, if effective, could easily be taken to scale. Findings from the study will identify critical components for male-specific counseling, especially among men who struggle to be retained in HIV care.

For peer review only

For peer review only - http://bmjopen.bmj.com/site/about/guidelines.xhtml

INTRODUCTION

Men in sub-Saharan Africa (SSA) are underrepresented in HIV programs.[1] Men are less likely than women to know their HIV status and to initiate antiretroviral therapy (ART), and more likely to face treatment interruptions once in care.[2] Men are particularly prone to cycling through ART programs, with more frequent stop-start instances and longer periods outside of care as compared to women.[5-8] Engagement in ART programs is not static – many men and women cycle through care, starting and stopping HIV care multiple times throughout their lifetime.[3,4] Men who disengage from HIV programs (either after testing HIV-positive or after enrolling in HIV care services) are frequently described as a difficult and 'hard-to-reach' population.[9,10] However, growing evidence suggests that men desire HIV services[11,12] but encounter multiple health systems barriers to care that make it impossible to stay in care long-term.[13] There is an urgent need to develop client-centered strategies tailored to men that facilitate men's engagement and re-engagement in HIV treatment programs.

Some men may require male-specific interventions to facilitate engagement in HIV care. Men have less exposure to HIV services than women[13,14] and work demands may conflict with ART clinic schedules.[15,16] Difficult interactions with health care workers (HCWs) can also prevent men from engaging or re-engaging in care.[17,18] Furthermore, most ART counseling curricula do not target men and often lack the client-centered counseling needed to develop internal motivation to engage and stay engaged in care.

Differentiated service delivery models (DSD) are now being developed to improve men's ART engagement throughout SSA.[19-21] As DSDs for men are developed, it is critical that strategies be feasible and cost-effective to allow scale-up. A "one size fits all" model is not as effective as more nuanced approaches.[22-24] Stepped interventions increase in intensity over time and are purposively designed to address prevailing barriers in the target population in order to positively affect the desired outcome.[25,26] An incremental, stepped approach may be the most appropriate and scalable way to improve men's care in low-resource settings. Men are not homogeneous: some men may require minimal support to engage in care, while others may require extensive

For peer review only - http://bmjopen.bmj.com/site/about/guidelines.xhtml

support. Stepped interventions allow programs to target the highest-resource interventions to the minority of men who need them most.

The *Identifying efficient linkage strategies for HIV self-testing (IDEaL) Trial* is an individually randomized control trial aimed to test the impact of various interventions on ART (re-) engagement and six-month retention among men living with HIV who are not currently engaged in HIV care in Malawi. We will compare a Stepped intervention against Low-Intensity and High-Intensity interventions to assess the impact of the Stepped intervention on men's use of ART services over time (see Supporting Information S1).

METHODS AND ANALYSIS

Objectives

Our primary objective is to test the effect of a male-specific, Stepped intervention on men's 6-month retention in ART care compared to male-specific Low-Intensity and High-Intensity interventions (retention is defined as <28-days late for their ART appointment). Secondary objectives are to understand the impact of a Stepped intervention on: (1) ART initiation; (2) the presence of adverse events (i.e., unwanted disclosure, end of relationship, or intimate partner violence (IPV)); (3) intervention acceptability; and (4) cost-effectiveness.

Trial Design

IDEaL is a programmatic, individually randomized, non-blinded, non-inferiority controlled trial design. We will recruit men from 15 high-burden health facilities in Malawi using medical chart reviews to identify men who are living with HIV but not engaged in HIV care.

Randomization

Individual men will be block randomized by a biostatistician using a 1:1:1 ratio to either the Stepped, Low-Intensity, or High-Intensity study arm using a computer-generated program. Participants will be randomized in blocks of 3 and 6, depending on the number of men available for recruitment at each facility. After enrolling in the trial and completing a baseline survey, men will be assigned to a study ID based on the randomization list. Study ID's will be linked with the pre-assigned blocked randomization and pre-loaded into the tablet device, but will be unknown

For peer review only - http://bmjopen.bmj.com/site/about/guidelines.xhtml

to the study staff until survey and randomization modules are completed and saved, ensuring randomization cannot be manipulated by the study staff. Once finalized, the randomization results will appear on the tablet device as a picture, and will be shown to the participant to maximize transparency and study buy-in.

Interventions

The effectiveness of the Stepped Intervention will be compared to a Low-Intensity Intervention (one time male-specific counseling + facility navigation) and to a High-Intensity Intervention (outside-facility ART initiation + male-specific counseling + facility navigation for follow-up ART visits). Across all arms, men who do not (re-)engage in ART will continue receiving follow-up visits for up to three months, depending on preferences of the client. The number of intervention visits delivered for each participant will be documented.

Arm 1: Stepped Arm

The Stepped arm will build in intensity over time for those who have not (re-)engaged in care 14-days after enrollment, or who do not return for their first ART follow-up appointment after (re-)engagement (see Fig 1). Individuals will move to the next "step" every 2-weeks, moving from the lightest to the most intensive interventions over the course of X weeks until (re-)engagement has been achieved (as defined by starting or restarting ART). The Stepped arm includes the following steps:

Step 1: Male-Specific Counseling + Facility Navigation: Participants will be traced in the community and receive a one-time, one-on-one, intensive counseling session in the community by a lower-level cadre male HIV counselor (called Patient Supporter in Malawi) using a male-specific counseling curriculum developed specifically for this trial.

Ministry of Health counseling materials will be adapted to develop the male-specific counseling curriculum, based on formative in-depth interviews, focus group discussions, and a systematic literature review. Adaptations will include exploring topics of most concern to men in Malawi (i.e., earning money while HIV-positive, side effects and concerns regarding lifelong medication, ART as a tool to provide and care for family, etc.). The materials will also include language and pictures

For peer review only - http://bmjopen.bmj.com/site/about/guidelines.xhtml

that resonate with men (i.e., emphasizing how HIV and HIV services interact with men's strength, responsibility, planning for the future), and male-specific case studies of challenges men face and how they overcome them. The adapted male-specific counseling curriculum will be developed into a standardized counseling flip chart to promote consistent and accurate implementation of the intervention.

Men who wish to (re-)engage in care will be provided with facility navigation, including being escorted to the facility (if desired) and being oriented to the ART clinic within the health facility by the same counselor who delivers the intervention. Men will access all ART clinical services at a health facility of their choice (but counseling described above will be provided in the community).

Step 2: Ongoing Motivational Interviewing + Facility Navigation: Men who do not (re-)engage in care (either have not engaged ART within 14 days of enrollment or do (re-)engage ART but are ≥7-days late for a follow-up appointment) will move to the next 'step' of the intervention, which adds ongoing motivational interviewing to their package of activities. Motivational interviewing is a validated form of counseling aimed to help individuals identify barriers to a desired outcome and develop personalized solutions.[27] Mentors will work with participants to: (1) build self-efficacy, (2) identify internal motivations for the desired behavior, and (3) establish strategies and short- and long-term goals needed to reach ART initiation and retention. A male mentor specifically trained in motivational interviewing will provide ongoing, one-on-one in-depth counseling, motivational interviewing, and general "check-ins" approximately twice within a two-week period. The mentor will not necessarily be HIV-positive (unlike other mentorship models) as the Malawi Ministry of Health has moved away from HIV-positive peer mentor cadres. However, they will be experienced in HIV counseling and trained on male-specific needs. Motivational interviewing will take place in a location preferred by the participant, likely in the community. Participants who choose to (re)engage in care will be given facility navigation.

Step 3: Outside-facility ART initiation + male-specific counseling + facility navigation: Men who are not engaged after Step 2 (either have not engaged in ART care within 14 days of receiving the first motivational interviewing or have engaged but are ≥7-days late for a follow-up ART

For peer review only - http://bmjopen.bmj.com/site/about/guidelines.xhtml

appointment) will be offered outside-facility ART initiation by a male nurse affiliated with the nearest public health facility. The nurse will meet the participant at a convenient and private location of his choice. Outside-facility ART will be male-friendly, including components like early hours and convenient locations.

The nurse will provide client-centered counseling by reviewing key points from male-specific counseling that are most relevant to the individual, and conduct WHO staging. Individuals classified as WHO Stage 3 or 4 will not be eligible for outside-facility ART and will be escorted to the nearest public health facility for additional services. Participants classified as WHO Stage 1 or 2 will be given ART for same day initiation. The nurse will schedule a 4-week follow-up ART refill appointment at the health facility of the man's choice. At the time of the follow-up appointment, the nurse will provide facility navigation to facilitate a positive experience engaging in or returning to care and serve as the ART provider for this appointment to facilitate service continuity and familiarity.

Arm 2: Low-Intensity Arm: Male-specific counseling + facility navigation

Participants randomized to the Low-Intensity Arm will be traced in the community and receive a one-time, one-on-one, intensive counseling session in the community by a lower-level cadre male HIV counselor. The counseling session will use the same male-specific counseling curriculum described in Step 1 of the Stepped Arm. For men who do not (re-)engage in care, follow-up counseling will be offered approximately every two weeks until individuals engage in care or inform the counselor that they do not wish to be contacted. Those who choose to engage in ART following male-specific counseling will be given facility navigation for their first ART appointment.

Arm 3: High-Intensity Arm: Outside-facility ART initiation + male-specific counseling + facility navigation

The High-Intensity Arm will offer the male-specific counseling described above, outside-facility ART initiation, and facility navigation for their 4-week follow-up appointment. After receiving male-specific counseling, participants interested in outside-facility ART initiation will be referred to a male study nurse who will meet men one-on-one at convenient times and locations. Those

For peer review only - http://bmjopen.bmj.com/site/about/guidelines.xhtml

who decline outside facility ART initiation will be referred immediately to the nearest health facility and receive facility navigation on a day and time that is convenient for them. For outside-facility ART, the nurse will provide client-centered counseling, reviewing key points from male-specific counseling that are most relevant and/or of greatest concern to the individual, conduct WHO staging, and for those in WHO Stage 1 or 2, provide same day ART initiation (as described in the Stepped Arm). The nurse will provide facility navigation for the 4-week ART refill appointment. Men who do not (re-)engage in care will be offered biweekly follow-up counseling at times and intervals determined by participants' preferences until they engage in care or inform the nurse that they no longer wish to be contacted.

[Figure 1 here]

Trial setting

The study will take place in central and southern Malawi. Malawi has an HIV prevalence of 9.6%[28] and, of the estimated 330,000 men living with HIV in the country, 54,500 are not in care.[29] Men in Malawi live in primarily rural settings, are self-employed, subsistence farmers, the minority have regular access to a private phone, and most are highly mobile.[30,31]

Population

We will recruit men from 15 high-burden health facilities in Malawi, using medical chart reviews to identify men living with HIV who are not engaged in HIV care. Study facilities will vary by facility type (hospital/health center), management (public/mission), location (rural/urban), and region (central/southern Malawi).

Eligibility criteria for men include: (1) ≥15years of age; (2) live in facility catchment area; and (3) tested HIV-positive and either (a) self-report having not yet initiated ART within 7-days of testing HIV-positive, (b) initiated ART but are at risk of immediate default (i.e., ≥7-days late for their 30-day ART refill appointment), or (c) initiated ART and attended their first refill appointment but later defaulted (i.e. ≥28-days late to care). For those who never initiated ART and do not have proof of a confirmatory HIV test, study staff will offer an HIV self-test kit prior to enrollment, to

For peer review only - http://bmjopen.bmj.com/site/about/guidelines.xhtml

confirm a positive HIV status. Those who choose to initiate ART will receive the standard Determine and Unigold confirmatory tests prior to ART initiation, following routine care.

Study outcomes

The primary outcome is the proportion of men who are retained in ART care 6-months after (re-) engagement. Secondary outcomes include: (1) ART initiation; (2) adverse events experienced by men or their female partners (i.e., unwanted disclosure, end of relationship, or intimate partner violence(IPV); (3) intervention acceptability; and (4) cost-effectiveness. ART retention outcomes will be measured through medical chart reviews, while secondary outcomes will be measured through self-reports. Process outcomes include: (1) the proportion of men who were successfully traced; (2) the proportion of eligible men who consented to participate; (3) men's experience with the intervention; and (4) the quality of the intervention.

Sample size considerations

We powered the study to detect differences in 6-month retention between Stepped and Low-Intensity Arms, and the Stepped and High-Intensity Arms. Based on previous trials, we assumed that 40% of men in the Low-Intensity Arm, 60% in the Stepped Arm, and 80% in the High-Intensity Arm will engage in ART and be retained at 6-months. Any man lost to follow-up will be treated as a failure for the outcome evaluation. With 181 men per arm and 20% loss to follow-up from the study in all arms, the power for detecting the specified difference between Stepped and Low Intensity arms will be 0.8 and the power to detect the specified difference between Stepped and High-Intensity arms will be >0.99, taking into account that two comparisons are made. The calculation is based on asymptotic normality of log odds ratio.[32] We need to enroll and randomize 181 men per arm (a total of 543 men living with HIV).

Data Collection

Study recruitment, enrollment, and data collection will be conducted by study staff, who are distinct from local HCWs implementing the interventions.

Recruitment

For peer review only - http://bmjopen.bmj.com/site/about/guidelines.xhtml

Men will be identified through both medical register reviews and in-person recruitment at participating health facilities. Various medical charts will be reviewed to identify different types of eligible men: HIV testing and counseling (HTC) to identify men who tested HIV positive but never initiated ART; client follow-up registers to identify those who initiated but never returned for their first ART appointment, or those who defaulted from care; and index counseling and testing (ICT) registers to identify male partners of female ART clients (Figure 2). In-person recruitment will involve screening men at outpatient departments (OPD) because our previous research has found that men in Malawi frequent OPD settings for health needs,[12] and our formative work suggests that men who disengage from ART services still frequent the OPD for care. In-person recruitment will be used for all client types.

[Figure 2 here]

Tracing and Eligibility Screening

Study staff will trace potential participants identified through medical chart reviews via phone (if available) or home visits based on tracing data provided in medical documentation. All potential participants will be traced up to three times before being considered lost to follow-up. All screening and enrollment processes will take place in-person.

Consent, Enrollment, and Baseline Survey

Men who are eligible for the study will complete written informed consent and complete a baseline survey immediately following enrollment. The baseline survey will collect data on key demographic variables (marital status, number of children, employment, self-rated health) and previous engagement with HIV and non-HIV health services. All surveys will be conducted in the local language (Chichewa) by trained study staff using electronic tablets. Surveys will be programmed using SurveyCTO software (http://www.surveycto.com).

Follow-Up Data

Study staff will administer follow-up surveys at 2- and 4-months after enrollment. Follow-up surveys will measure exposure to (and acceptability of) the interventions, changes in key demographics since enrollment (i.e., marital status, number of children, employment, self-rated

For peer review only - http://bmjopen.bmj.com/site/about/guidelines.xhtml

health), any adverse events since enrollment (i.e., unwanted status disclosure, termination of relationship due to the intervention), and use of ART services. The location and specific time of the follow-up survey will be based on participant preference.

Medical chart reviews will be conducted to assess men's engagement with ART services 6-months after study enrollment. Individuals without a medical chart outcome will be followed-up in person and their health passport, a pocket medical record where providers record data during health visits, will be reviewed to collect the ART outcome. Men who cannot be reached or are lost to follow-up in any arm will be counted as failures for that specific ART outcome of interest: (re-)engagement or 6-month retention).

Patient and Public Involvement

Extensive formative work informed the development of the study protocol including in-depth interviews, focus group discussions, and a systematic literature review. The study protocol and tools were presented to Ministry of Health, national stakeholders and implementing partners (see Supporting Information S2).

Cost data

The average cost per successful outcome (6-month retention) will be calculated and compared across arms incrementally. We will use micro-costing methods by creating an inventory of the resources used to achieve the observed study outcomes including: (1) standard counseling interactions (staff cadre, training received, duration of interaction and distance from facility travelled where applicable); (2) motivational interviewing interactions (staff cadre, training received, duration of interaction and distance from facility travelled where applicable); (3) provider interactions (staff cadre, training received, duration of interaction and distance from facility travelled where applicable); and (4) cost of reminder messages sent, when messages are delivered telephonically instead of in person. For each study patient, the quantity (number of units) of resources used will be determined. Costs will be measured from the health care provider perspective. Unit costs of resources, which are not human subject data, will be obtained from external suppliers and the health facilities' finance and procurement records and multiplied by the resource usage data to provide an average cost per study patient in each study arm. A cost-

For peer review only - http://bmjopen.bmj.com/site/about/guidelines.xhtml

effectiveness analysis will be conducted by dividing the incremental cost between two arms by the incremental effectiveness (number of people retained at 6-months) in the respective arms.

Analysis plan

Data analysis will be conducted in R: A Language and Environment for Statistical Computing (R Foundation for Statistical Computing). We will use the Consolidated Standards of Reporting Trials (CONSORT) standards for reporting trial outcomes.[33] Using an intention-to-treat analysis, all randomized men will be included in the analysis of the primary outcome; men with missing outcome assessment due to loss to follow-up will be treated as outcome failures. We will calculate descriptive statistics, including mean, standard deviation, range, and frequency distributions for the demographic characteristics and study outcomes by study arm. The primary outcome and all other binary outcomes will be analyzed by logistic regression models with key sociodemographic variables included as covariates (i.e., age and marital status). The intervention effects will be tested by pairwise Wald tests with Bonferroni adjustment. Confidence intervals for intervention effects will be calculated by profile likelihood methods, also with Bonferroni adjustment. To address the secondary objectives, more elaborate logistic regression models will be built for each of the binary outcomes with available individual-, community-, and facility-level factors included as covariates in addition to the intervention status.

Nested studies

A series of nested, mixed methods studies will be conducted to identify factors associated with ART engagement within each intervention arm, and to explore the implementation and acceptability of interventions.

Qualitative data collection

We will conduct in-depth interviews with a random subset of 120 male participants throughout the study period in order to assess characteristics of men who fail to engage in care, contextualize decisions around ART initiation and retention, and identify additional strategies that may be needed for men to successfully engage and be retained in ART programs.

For peer review only - http://bmjopen.bmj.com/site/about/guidelines.xhtml

Interviews will be conducted by a trained male interviewer. Interviews will be digitally recorded, transcribed, and translated into English for analysis. Investigators will pilot a codebook by independently reading and coding a randomly-selected subset of transcripts. Through an iterative consultative process, each investigator will revise their respective codebook until there is high interrater reliability among the group. All transcripts will be coded in Atlas.ti v8.3 using constant comparison, and coding disagreements will be resolved by consensus.

Implementation Log Sheet

During the course of the intervention, HCWs will keep daily logs as one of the study monitoring and evaluation tools to assess the implementation of the intervention for each participant. Primary events to be recorded in the daily logs are: (1) unable to reach participant (and reason); (2) contacted participant; (3) intervention provided (and notes about the challenges and successes of the interaction); and (4) other comments relevant to intervention implementation. Each event will be recorded with a corresponding date. Logs will be digitized in English. Findings may influence how similar interventions are implemented in the future.

Ethics and Dissemination

The IDEaL trial is registered with ClinicalTrials.gov as NCT05137210. The protocol was approved by the Institutional Review Board of the University of California, Los Angeles and the National Health Sciences Research Council in Malawi. Study findings will be disseminated through peer-reviewed journal articles, national and international conference presentations, and meetings with Malawi Ministry of Health, facility, and community stakeholders.

DISCUSSION

Studies have reported poorer outcomes for HIV testing, treatment initiation, and treatment adherence in men compared to women[2] for over a decade.[34,35] Men are often portrayed as difficult, hard-to-reach, and actively avoiding health facilities. In IDEaL, we aim to investigate whether men really are hard-to-reach or, will men engage in care when services are offered in ways that are accessible to them and resonate with their needs, as growing evidence suggests.[12] We propose to test a Stepped intervention that increases in intensity over time against Low- and

For peer review only - http://bmjopen.bmj.com/site/about/guidelines.xhtml

High-intensity interventions – all tailored to men – to identify the most cost effective strategy to (re-)engage men in HIV treatment services in Malawi.

IDEaL is different from other ART engagement and re-engagement interventions in several important ways. First, we will enroll men living with HIV across the treatment cascade, including those who have never initiated ART, those who are at risk of immediate default after initiation, and those who have been in care but subsequently default. Formative research suggests that barriers to ART initiation and re-initiation may be similar,[36] however most interventions focus specifically on either first-time initiation or re-engagement, but not both. Our study will assess if one overarching program can improve men's engagement across the treatment cascade, regardless of whether they are starting ART for the first time or returning to care after a period of disengagement. One overarching intervention may be more scalable than multiple, separate interventions across the cascade. Second, we tailor interventions to men's unique needs and motivations, based on extensive formative work. While innovative interventions for men are underway,[19-21] few have rigorously tested the impact of male-tailored interventions on ART engagement.[37]

Our study will measure adverse events including unwanted disclosure, end of a relationship, and/or intimate partner violence (IPV). We will also measure female partner perceptions of the feasibility and acceptability of a male-only intervention, and any unintended or adverse events such as IPV. Understanding these events will be critical to evaluating the feasibility of scale-up should one or more of the approaches prove effective.

Finally, we will test a Stepped intervention that builds in intensity over time until men (re-) engage in care. This approach allows men who are ready to (re-)engage to do so at minimal cost to the health system, while those who need additional support can receive more resource-intensive interventions to support their ART engagement.[25] Stepped interventions have been effective in other settings and can address multiple barriers faced by the target population with minimal cost.[25,26] Findings from IDEaL will provide crucial knowledge to how best men can be reached and can inform intervention scale-up.

For peer review only - http://bmjopen.bmj.com/site/about/guidelines.xhtml

Contributorship statement: KD and AC conceptualized the study. KD is responsible for funding acquisition. KD, KB, JH, KP, BN, RH and AC developed study protocol and materials. JH, KB, KP, and EC will implement the study. KD, MK, KB, TC, BN, LL, TC, and AC developed the analysis plan and KD, MK, KB, and AC will analyze the data. KD and EC wrote the first draft and KB, JH, KP, BN, LL, RH, SP, EC, RH, TC, and AC edited following drafts. All authors have read and approved the final manuscript.

Competing interests: The authors declare that they have no competing interests.

Funding: The work was supported by the Bill and Melinda Gates Foundation grant number INV-001423. KD was supported by National Institute of Mental Health of the National Institutes of Health grant number R01-MH122308, Fogarty International grant number K01-TW011484-01 and UCLA GSTTP (grant number N/A). LL was supported by the National Institute of Mental Health of the National Institutes of Health under grant number K01MH119923. The content is solely the responsibility of the authors and does not necessarily represent the official views of the National Institutes of Health.

Acknowledgements: We wish to thank the Malawi Ministry of Health for their support of this trial. We would also like to acknowledge Joep van Oosterhout, Misheck Mphande, Isabella Robson, Thoko Banda, Peter Mwamlima, and Eric Lungu for their contributions to protocol development and for their support of study implementation, and to Hannah Whitehead for assistance with manuscript development and submission.

For peer review only

REFERENCES

1. Cornell M, Majola M, Johnson LF, Dubula-Majola V. HIV services in sub-Saharan Africa: the greatest gap is men. *The Lancet.* 2021;397(10290):2130-2132.
2. UNAIDS 'AIDSinfo' (accessed September 2021).
3. Ehrenkranz P, Rosen S, Boulle A, et al. The revolving door of HIV care: Revising the service delivery cascade to achieve the UNAIDS 95-95-95 goals. *PLoS Medicine.* 2021;18(5):e1003651.
4. Grimsrud A, Wilkinson L, Eshun-Wilson I, Holmes C, Sikazwe I, Katz IT. Understanding engagement in HIV programmes: how health services can adapt to ensure no one is left behind. *Current HIV/AIDS Reports.* 2020:1-9.

For peer review only - http://bmjopen.bmj.com/site/about/guidelines.xhtml

5. Lopez-Varela E, Augusto O, Fuente-Soro L, et al. Quantifying the gender gap in the HIV care cascade in southern Mozambique: We are missing the men. *PLOS ONE.* 2021;16(2):e0245461.
6. Kranzer K, Lewis JJ, Ford N, et al. Treatment interruption in a primary care antiretroviral therapy program in South Africa: cohort analysis of trends and risk factors. *Journal of acquired immune deficiency syndromes (1999).* 2010;55(3):e17-e23.
7. Mody A, Eshun-Wilson I, Sikombe K, et al. Longitudinal engagement trajectories and risk of death among new ART starters in Zambia: A group-based multi-trajectory analysis. *PLoS medicine.* 2019;16(10):e1002959-e1002959.
8. Gosset A, Protopopescu C, Larmarange J, et al. Retention in Care Trajectories of HIV-Positive Individuals Participating in a Universal Test-and-Treat Program in Rural South Africa (ANRS 12249 TasP Trial). *J Acquir Immune Defic Syndr.* 2019;80(4):375-385.
9. Esacove AW. Good sex/bad sex: the individualised focus of US HIV prevention policy in sub-Saharan Africa, 1995–2005. *Sociology of health & illness.* 2013;35(1):33-48.
10. Poulin M, Dovel K, Watkins SC. Men with money and the "vulnerable women" client category in an AIDS epidemic. *World Development.* 2016;85:16-30.
11. Chamberlin S, Mphande M, Phiri K, Kalande P, Dovel K. How HIV Clients Find Their Way Back to the ART Clinic: A Qualitative Study of Disengagement and Re-engagement with HIV Care in Malawi. *AIDS and Behavior.* 2021:1-12.
12. Dovel K, Balakasi K, Gupta S, et al. Frequency of visits to health facilities and HIV services offered to men, Malawi. *Bulletin of the World Health Organization.* 2021;99(9):618.
13. Dovel K, Dworkin SL, Cornell M, Coates TJ, Yeatman S. Gendered health institutions: examining the organization of health services and men's use of HIV testing in Malawi. *Journal of the International AIDS Society.* 2020;23:e25517.
14. Yeatman S, Chamberlin S, Dovel K. Women's (health) work: A population-based, cross-sectional study of gender differences in time spent seeking health care in Malawi. *PLoS One.* 2018;13(12):e0209586.
15. Colvin CJ. Strategies for engaging men in HIV services. *The Lancet HIV.* 2019;6(3):e191-e200.
16. Phiri K, McBride K, Moucheraud C, et al. Community and health system factors associated with antiretroviral therapy initiation among men and women in Malawi: a mixed methods study exploring gender-specific barriers to care. *International health.* 2021;13(3):253-261.
17. Ware NC, Wyatt MA, Geng EH, et al. Toward an understanding of disengagement from HIV treatment and care in sub-Saharan Africa: a qualitative study. *PLoS medicine.* 2013;10(1):e1001369.
18. Layer EH, Brahmbhatt H, Beckham SW, et al. "I Pray That They Accept Me Without Scolding:" Experiences with Disengagement and Re-Engagement in HIV Care and Treatment Services in Tanzania. *AIDS Patient Care and STDs.* 2014;28(9):483-488.
19. Bell J, Sharma S, Malone S, et al. Targeting interventions for HIV testing and treatment uptake: An attitudinal and behavioural segmentation of men aged 20–34 in KwaZulu-Natal and Mpumalanga, South Africa. *PloS one.* 2021;16(3):e0247483.
20. van Rooyen H, Makusha T, Joseph P, et al. Zwakala Ndoda: a cluster and individually randomized trial aimed at improving testing, linkage, and adherence to treatment for hard-to reach men in KwaZulu-Natal, South Africa. *Trials.* 2019;20(1):1-14.

For peer review only - http://bmjopen.bmj.com/site/about/guidelines.xhtml

21. Coalition M. *MenStar Strategy.* 2020.
22. Grimsrud A, Bygrave H, Doherty M, et al. Reimagining HIV service delivery: the role of differentiated care from prevention to suppression. *Journal of the International AIDS Society.* 2016;19(1):21484-21484.
23. Duncombe C, Rosenblum S, Hellmann N, et al. Reframing HIV care: putting people at the centre of antiretroviral delivery. *Tropical Medicine & International Health.* 2015;20(4):430-447.
24. Hagey JM, Li X, Barr-Walker J, et al. Differentiated HIV care in sub-Saharan Africa: a scoping review to inform antiretroviral therapy provision for stable HIV-infected individuals in Kenya. *AIDS Care.* 2018;30(12):1477-1487.
25. Bower P, Gilbody S. Stepped care in psychological therapies: access, effectiveness and efficiency: narrative literature review. *The British Journal of Psychiatry.* 2005;186(1):11-17.
26. McNairy ML, Lamb MR, Gachuhi AB, et al. Effectiveness of a combination strategy for linkage and retention in adult HIV care in Swaziland: The Link4Health cluster randomized trial. *PLoS medicine.* 2017;14(11):e1002420-e1002420.
27. Rollnick S, Miller WR. What is Motivational Interviewing? *Behavioural and Cognitive Psychotherapy.* 1995;23(4):325-334.
28. UNAIDS. Focus on: Malawi. 2017; https://www.unaids.org/en/20190402_country_focus_Malawi. Accessed October 12, 2021.
29. UNAIDS. Country factsheets: Malawi 2020. 2020; https://www.unaids.org/en/regionscountries/countries/malawi. Accessed October, 2021.
30. Feldacker C, Emch M, Ennett S. The who and where of HIV in rural Malawi: Exploring the effects of person and place on individual HIV status. *Health Place.* 2010;16(5):996-1006.
31. Mandiwa C, Namondwe B. Uptake and correlates of HIV testing among men in Malawi: evidence from a national population–based household survey. *BMC Health Services Research.* 2019;19(1):203.
32. Jewell NP. Section 7.1. In: *Statistics for Epidemiology.* Chapman and Hall/CRC; 2004.
33. Schulz KF, Altman DG, Moher D. CONSORT 2010 Statement: updated guidelines for reporting parallel group randomised trials. *BMJ.* 2010;340:c332.
34. Higgins JA, Hoffman S, Dworkin SL. Rethinking gender, heterosexual men, and women's vulnerability to HIV/AIDS. *American journal of public health.* 2010;100(3):435-445.
35. Braitstein P, Boulle A, Nash D, et al. Gender and the use of antiretroviral treatment in resource-constrained settings: findings from a multicenter collaboration. *Journal of women's health.* 2008;17(1):47-55.
36. Coursey K, Kalande P, Phiri K, Chamberlin S, Coates T, Dovel K. Developing intervention strategies to improve ART program initiation and retention among HIV-positive men in Malawi.
37. Kusemererwa S, Akena D, Nakanjako D, et al. Strategies for retention of heterosexual men in HIV care in sub-Saharan Africa: A systematic review. *PloS one.* 2021;16(2):e0246471.

For peer review only

FIGURE LEGEND

Figure 1: Trial Design

Figure 2: Recruitment sources and ART disengagement criteria by recruitment type

For peer review only

For peer review only - http://bmjopen.bmj.com/site/about/guidelines.xhtml

Figure 1: Trial Design

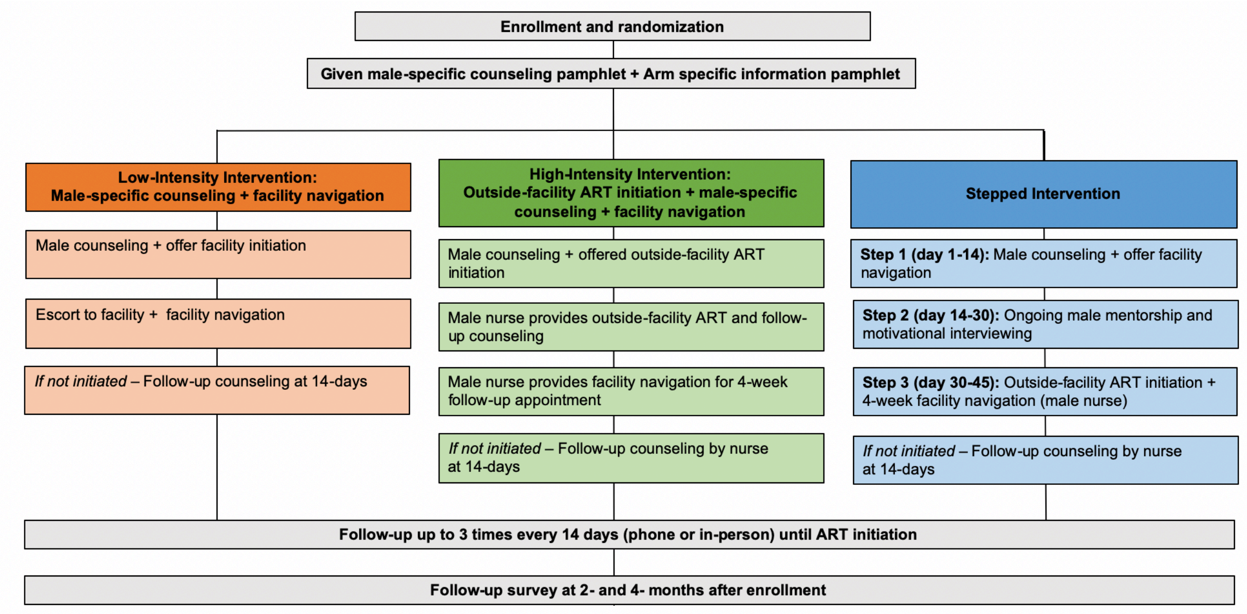

For peer review only

Fig 2. Recruitment sources and ART disengagement criteria by recruitment type

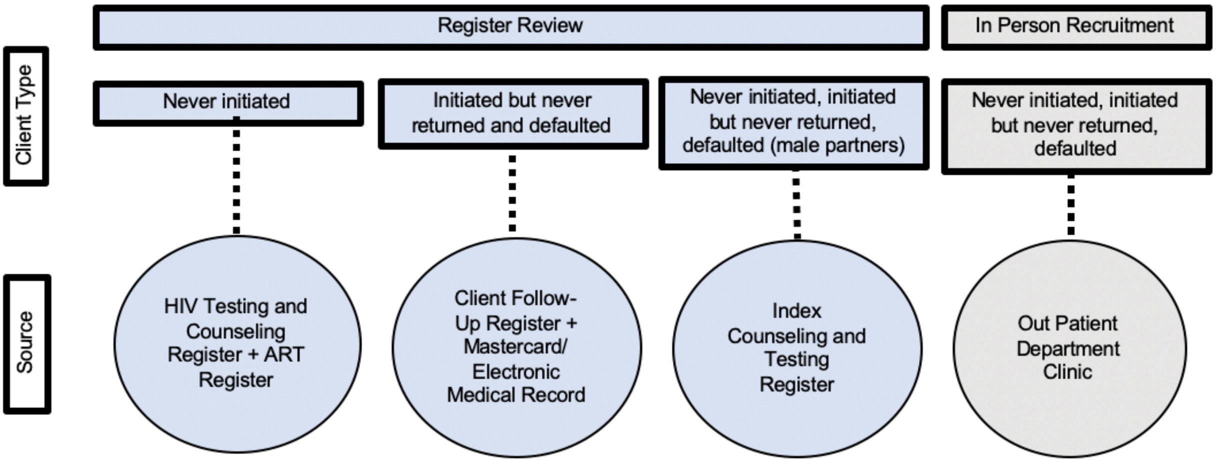

For peer review only

SPIRIT
STANDARD PROTOCOL ITEMS: RECOMMENDATIONS FOR INTERVENTIONAL TRIALS

SPIRIT 2013 Checklist: Recommended items to address in a clinical trial protocol and related documents*

Section/item	Item No	Description	Addressed on page number
**Administrative information**			
Title	1	Descriptive title identifying the study design, population, interventions, and, if applicable, trial acronym	______1_______
Trial registration	2a	Trial identifier and registry name. If not yet registered, name of intended registry	______1, 15_____
	2b	All items from the World Health Organization Trial Registration Data Set	______n/a______
Protocol version	3	Date and version identifier	______2_______
Funding	4	Sources and types of financial, material, and other support	______19______
Roles and responsibilities	5a	Names, affiliations, and roles of protocol contributors	_____18-19____
	5b	Name and contact information for the trial sponsor	______19______
	5c	Role of study sponsor and funders, if any, in study design; collection, management, analysis, and interpretation of data; writing of the report; and the decision to submit the report for publication, including whether they will have ultimate authority over any of these activities	______19_______
	5d	Composition, roles, and responsibilities of the coordinating centre, steering committee, endpoint adjudication committee, data management team, and other individuals or groups overseeing the trial, if applicable (see Item 21a for data monitoring committee)	_____n/a_______

For peer review only

Introduction

Background and rationale	6a	Description of research question and justification for undertaking the trial, including summary of relevant studies (published and unpublished) examining benefits and harms for each intervention	____4-5_______
	6b	Explanation for choice of comparators	_____ ________
Objectives	7	Specific objectives or hypotheses	_____5_______
Trial design	8	Description of trial design including type of trial (eg, parallel group, crossover, factorial, single group), allocation ratio, and framework (eg, superiority, equivalence, noninferiority, exploratory)	_______5_______

Methods: Participants, interventions, and outcomes

Study setting	9	Description of study settings (eg, community clinic, academic hospital) and list of countries where data will be collected. Reference to where list of study sites can be obtained	____9-10_______
Eligibility criteria	10	Inclusion and exclusion criteria for participants. If applicable, eligibility criteria for study centres and individuals who will perform the interventions (eg, surgeons, psychotherapists)	____10______
Interventions	11a	Interventions for each group with sufficient detail to allow replication, including how and when they will be administered	____6-9______
	11b	Criteria for discontinuing or modifying allocated interventions for a given trial participant (eg, drug dose change in response to harms, participant request, or improving/worsening disease)	_______n/a______
	11c	Strategies to improve adherence to intervention protocols, and any procedures for monitoring adherence (eg, drug tablet return, laboratory tests)	_______n/a______
	11d	Relevant concomitant care and interventions that are permitted or prohibited during the trial	_______n/a______
Outcomes	12	Primary, secondary, and other outcomes, including the specific measurement variable (eg, systolic blood pressure), analysis metric (eg, change from baseline, final value, time to event), method of aggregation (eg, median, proportion), and time point for each outcome. Explanation of the clinical relevance of chosen efficacy and harm outcomes is strongly recommended	______10_____
Participant timeline	13	Time schedule of enrolment, interventions (including any run-ins and washouts), assessments, and visits for participants. A schematic diagram is highly recommended (see Figure)	__6,8-9, 11-12__

For peer review only - http://bmjopen.bmj.com/site/about/guidelines.xhtml

Sample size	14	Estimated number of participants needed to achieve study objectives and how it was determined, including clinical and statistical assumptions supporting any sample size calculations	____10-11_____
Recruitment	15	Strategies for achieving adequate participant enrolment to reach target sample size	____11-12______

Methods: Assignment of interventions (for controlled trials)

Allocation:

Sequence generation	16a	Method of generating the allocation sequence (eg, computer-generated random numbers), and list of any factors for stratification. To reduce predictability of a random sequence, details of any planned restriction (eg, blocking) should be provided in a separate document that is unavailable to those who enrol participants or assign interventions	_____5-6_______
Allocation concealment mechanism	16b	Mechanism of implementing the allocation sequence (eg, central telephone; sequentially numbered, opaque, sealed envelopes), describing any steps to conceal the sequence until interventions are assigned	_____5-6_______
Implementation	16c	Who will generate the allocation sequence, who will enrol participants, and who will assign participants to interventions	______5_______
Blinding (masking)	17a	Who will be blinded after assignment to interventions (eg, trial participants, care providers, outcome assessors, data analysts), and how	______6_______
	17b	If blinded, circumstances under which unblinding is permissible, and procedure for revealing a participant's allocated intervention during the trial	______n/a______

Methods: Data collection, management, and analysis

Data collection methods	18a	Plans for assessment and collection of outcome, baseline, and other trial data, including any related processes to promote data quality (eg, duplicate measurements, training of assessors) and a description of study instruments (eg, questionnaires, laboratory tests) along with their reliability and validity, if known. Reference to where data collection forms can be found, if not in the protocol	_____12_____
	18b	Plans to promote participant retention and complete follow-up, including list of any outcome data to be collected for participants who discontinue or deviate from intervention protocols	_____12_____

Data management	19	Plans for data entry, coding, security, and storage, including any related processes to promote data quality (eg, double data entry; range checks for data values). Reference to where details of data management procedures can be found, if not in the protocol	_______n/a_______
Statistical methods	20a	Statistical methods for analysing primary and secondary outcomes. Reference to where other details of the statistical analysis plan can be found, if not in the protocol	_____13-14_______
	20b	Methods for any additional analyses (eg, subgroup and adjusted analyses)	___13-14____
	20c	Definition of analysis population relating to protocol non-adherence (eg, as randomised analysis), and any statistical methods to handle missing data (eg, multiple imputation)	______13-14______
**Methods: Monitoring**			
Data monitoring	21a	Composition of data monitoring committee (DMC); summary of its role and reporting structure; statement of whether it is independent from the sponsor and competing interests; and reference to where further details about its charter can be found, if not in the protocol. Alternatively, an explanation of why a DMC is not needed	_______n/a_______
	21b	Description of any interim analyses and stopping guidelines, including who will have access to these interim results and make the final decision to terminate the trial	_______n/a_______
Harms	22	Plans for collecting, assessing, reporting, and managing solicited and spontaneously reported adverse events and other unintended effects of trial interventions or trial conduct	_____12 _______
Auditing	23	Frequency and procedures for auditing trial conduct, if any, and whether the process will be independent from investigators and the sponsor	_______n/a_______
**Ethics and dissemination**			
Research ethics approval	24	Plans for seeking research ethics committee/institutional review board (REC/IRB) approval	_______15________ _
Protocol amendments	25	Plans for communicating important protocol modifications (eg, changes to eligibility criteria, outcomes, analyses) to relevant parties (eg, investigators, REC/IRBs, trial participants, trial registries, journals, regulators)	______n/a_______

Consent or assent	26a	Who will obtain informed consent or assent from potential trial participants or authorised surrogates, and how (see Item 32)	______12_______
	26b	Additional consent provisions for collection and use of participant data and biological specimens in ancillary studies, if applicable	______n/a______
Confidentiality	27	How personal information about potential and enrolled participants will be collected, shared, and maintained in order to protect confidentiality before, during, and after the trial	______5______
Declaration of interests	28	Financial and other competing interests for principal investigators for the overall trial and each study site	______19_______
Access to data	29	Statement of who will have access to the final trial dataset, and disclosure of contractual agreements that limit such access for investigators	______n/a______
Ancillary and post-trial care	30	Provisions, if any, for ancillary and post-trial care, and for compensation to those who suffer harm from trial participation	______n/a______
Dissemination policy	31a	Plans for investigators and sponsor to communicate trial results to participants, healthcare professionals, the public, and other relevant groups (eg, via publication, reporting in results databases, or other data sharing arrangements), including any publication restrictions	____2,15______
	31b	Authorship eligibility guidelines and any intended use of professional writers	______n/a______
	31c	Plans, if any, for granting public access to the full protocol, participant-level dataset, and statistical code	______n/a______
**Appendices**			
Informed consent materials	32	Model consent form and other related documentation given to participants and authorised surrogates	_____ n/a ______
Biological specimens	33	Plans for collection, laboratory evaluation, and storage of biological specimens for genetic or molecular analysis in the current trial and for future use in ancillary studies, if applicable	______n/a______

*It is strongly recommended that this checklist be read in conjunction with the SPIRIT 2013 Explanation & Elaboration for important clarification on the items. Amendments to the protocol should be tracked and dated. The SPIRIT checklist is copyrighted by the SPIRIT Group under the Creative Commons "Attribution-NonCommercial-NoDerivs 3.0 Unported" license.

Partners in Hope Medical Center

Identifying efficient linkage strategies for HIV self-testing (IDEaL)

For peer review only

Kathyrn Dovel, Principle Investigator
Partners in Hope
PO Box 302
3-6-2019

ABSTRACT

Background: HIV self-testing (HIVST) has been found to be a highly acceptable approach for men to learn of their HIV status and has resulted in increased testing uptake (Dovel 2019, cite Augustines stuff). However, rates of antiretroviral therapy (ART) initiation among those tested with HIVST are difficult to capture and some studies have suggested that linkage rates are low (Ortblad 2017, MacPherson 2014), particularly amongst men. We propose a clinical trial to test varying approaches to ART initiation among men who test HIV-positive through HIVST. We will test three interventions:

Lightest Touch Intervention (Arm 1): simple reminders to visit the health facility (given every two weeks);

Staged Intervention (Arm 2): a staged intervention that consecutively increases in intensity every month that a participant does not initiate ART (intervals include reminders, motivational interviewing, and home-based ART initiation);

Intensive Intervention (Arm 3): home-based ART initiation followed by linkage to the health facility of their choice the following month.

Objective: Our primary objective is to identify a cost-effective package for ART initiation among men identified as HIV-positive through HIVST in Malawi. Our specific objectives are:

Objective 1. Evaluate the effectiveness of the Staged ART Intervention vs Lightest Touch Intervention (primary analysis) and the effectiveness of the Staged ART Intervention vs Intensive Intervention (secondary analysis) on ART initiation within 4-months after enrolment in the trial.

Objective 2. Identify individual-, community-, and facility-level factors associated with ART initiation within each intervention arm (Lightest Touch; Staged; and Intensive Interventions).

Objective 3. Determine the cost and scalability of each intervention (Lightest Touch; Staged; and Intensive Interventions).

Methods: We will preform an individually randomized control trial with 543 HIV-positive men identified through HIVST and their female partners. Men will be individually randomized 1:1:1 to one of the three intervention arms described above. The study will be preformed at 10 health facilities suppored by Partners in Hope (PIH). Data collection will include baseline and follow-up surveys and interviews with men and women; medical charter reviews at four-months after study enrollment; qualitative interviews; and a cost analysis of costs associated with each arm. Participants will be enrolled in the study for a total of 4 months with approximately 2 or 3 study visits throughout that period.

Anticipated results: We anticipate learning about the most effective stragty to engage men in ART. We also anticipate learning about the type and degree of followup necessary to support men's engagement in ART services. Finally, we anticipate learning about the cost-effectiveness of intervention, with the goal of improving cost-effectiveness for the Ministry of Health. Results from this study could be used to define best practices and to further scale ART-focused programs for men in Malawi.

For peer review only

TABLE OF CONTENTS

1. INTRODUCTION 4

1.1. Background 4
1.2. Problem statement 4
1.3. Justification 4

2. OBJECTIVES 4

2.1. Primary objective........ 4
2.2. Secondary objectives........ 4

3. LITERATURE REVIEW........ 5
4. METHODOLOGY 8

4.1. Intervention description 9
4.2. Place of study 11
4.3. OBJECTIVE 1........ 12

4.3.1. Study design........ 12
4.3.2. Target population 12
4.3.3. Sampling techniques and enrollment........ 13
4.3.4. Study Activites........ 13
4.3.5. Data collection techniques and tools 15
4.3.6. Sample size determination........ 17
4.3.7. Data analysis 17

4.4. OBJECTIVE 2........ 17

4.4.1. Study design........ 17
4.4.2. Target population 17
4.4.3. Sampling techniques and tools........ 18
4.4.4. Data collection techniques and tools 18
4.4.5. Sample size determination........ 19
4.4.6. Data analysis 19

4.5. OBJECTIVE 3........ 20

4.5.1. Study design........ 20
4.5.2. Data collection techniques and tools 20
4.5.3. Data analysis 20

5. ETHICAL CONSIDERATIONS 21
6. DISSMINATION OF RESULTS 22
7. PERSONNEL ROLES AND INSTITUTIONS........ 23
8. REGULATORY OVERSIGHT 24
9. STUDY IMPLEMENTATION 24
10. PROTOCOL DEVIATION REPORTING........ 25
11. WORK PLAN TIMELINE........ 25
12. BUDGET AND JUSTIFICATION........ 25
13. REFERENCES 27
14. APPENDIX 30

14.1. APPENDIX A – Study Invitation Cards 30

For peer review only

14.2. APPENDIX B – Recruitment and Screening Script 32
14.3. APPENDIX C – Written Informed Consent- Female 36
14.4. APPENDIX D: Written Informed Consent – Male 46
14.5. APPENDIX E: Baseline Survey – Male 55
14.6. APPENDIX F: Baseline survey – Female 100
14.7. APPENDIX G: Follow-up Survey - Male 129
14.8. APPENDIX H: Follow-up Survey – Female 137
14.9. APPENDIX I: Data Extraction Tool 144
14.10. APPENDIX J: In-Depth Interview Guide – Female 147
14.11. APPENDIX K: In-Depth Interview Guide – Male 155
14.12. APPENDIX L: Personel CV's 165

For peer review only

1. INTRODUCTION

1.1. Background

HIV self-testing (HIVST) is an effective strategy to improve HIV testing coverage, especially among hard-to- reach populations such as men and youth. Index testing, whereby a HIV positive client gives an HIVST kit to their sexual partner to use at home, is considered beneficial for its ability to maintain a testers privacy. The method is now recommended by the World Health Organization (WHO) and is being adopted as policy throughout sub-Saharan Africa (SSA). However, uptake of antiretroviral therapy (ART) and adherence after utilizing HIVST remains sub-optimal among certain population, specifically men. Innovative ART initiation and early retention strategies are urgently needed for Index HIVST to be successful.

1.2. Problem statement

We must better understand how to engage men in HIV care. Specifically, there is limited literature on feasible differentiated models to support men to start and stay on treatment that can be taken to scale. Further, there is increased recognition that individuals are at greatest risk of loss-to-follow-up during transition periods across the cascade (i.e. when starting ART). ART initiation and early retention must be improved if HIVST is to become a viable option for high-risk groups in SSA. To address this gap, we propose to conduct a study to test and evaluate varying strategies for ART initiation and retention amongst men.

1.3. Justification

This study will combine HIVST with a second-level intervention focused on ART initiation to address the urgent gap in ART initiation and early retention among HIVST users. Additionlly, Objective 3 will allow us to develop the lowest cost intervention package while reaching the highest number of male partners.

2. OBJECTIVES

2.1. Primary objective

Objective 1. Test the impact of a staged ART intervention vs simple reminders and the effectiveness of a staged ART intervention vs home-based ART on ART initiation within 3-months of an HIV-positive diagnosis

2.2. Secondary objectives

Objective 2. Identify individual-, community-, and facility-level factors associated with ART initiation within each intervention.

Objective 3. Determine the cost and scalability of each intervention.

For peer review only

3. LITERATURE REVIEW

Background

Men in sub-Saharan Africa are less likely than women to use HIV services.[1] Men's absence from care is concerning not only for their own health, but also for the health of girls and young women who continue to be infected at unacceptably high rates.[2] HIV prevention and treatment programs have not traditionally been directed at men. Men are notably absent from international guidelines, national policies, and local HIV interventions. Research shows that women are 322% more likely to be mentioned in international HIV guidelines than men.[3] In the context of Malawi, national guidelines expect women of reproductive age to attend a health facility 5-17 times per year (equivalent to 19-63 hours)[4], and 180-472 times in their reproductive lifespan (15-44 years). There are no such expectations for men (see Table 1). The justification for the global attention of HIV program thus far on women and girls is without dispute. Gender inequality is a key driver which impacts women's health and access to HIV services and creates specific vulnerabilities for women to HIV infection.[5] However, framing HIV as a woman's concern means we have failed to understand how gender affects and drives the burden of ill health for men, and inadvertently perpetuates the epidemic for young women and girls. Targeted strategies specific to men are urgently needed if we are to engage them in care.

Table 1: *Malawi ministry of health recommended health services and estimated visits required across the reproductive life span (15-44 years)*

			Estimated number of visits between 15-44 years			
Service	Frequency	Target Population	Women: 5-year FP (Implant; 9%*)	Women: quarterly FG (injectables; 23%*)	Women: monthly FP (pills, 2%*)	Men
ANC	17.6	Women	18	18	18	-
Delivery	4.4	Women	4	4	4	-
Post-natal	4.4	Women	4	4	4	-
Family Planning	88	Women	7	88	264	-
Under five	120	Women	120	120	120	-
HIV testing	22.6	Women and men	23	23	23	29
Circumcision	3	Men	-	-	-	3
**Total**			**176**	**257**	**433**	**32**

Male partners of women who are already identified as HIV-positive (index partners) are still a major concern for epidemic control due to high rates of multiple and concurrent partnerships among men[6] and the fact that index male partners have two times the risk of being HIV-positive as compared to the general male population.[7] Data from a recent HIVST study show that there is a high need for index testing among men in Malawi: across 3 high-burden district hospitals in Malawi, men represented 73% of all index partners in need of testing. Among those who did test, male partners were 4 times more likely to test HIV-positive than female partners (23% versus 3%), representing urgent unmet need among men.[8]

A recent Index HIV Trial in Malawi found that HIV testing among men increased dramatically when HIV-positive clients give HIVST kits to their sexual partners to use at home.[9] The study found that 66% of male partners in the HIVST arm tested for HIV compared to only 22% of men in the standard partner referral slip arm. Within the HIVST arm, men who tested for HIV had an HIV-positivity rate of 23%, with no adverse events reported (see Fig 1).[2] Index HIVST is highly acceptable and allows men to test at times and locations convenient for them, with complete privacy in their own homes.[10,11]

However, innovative ART initiation and early retention strategies are urgently needed for Index HIVST

to be successful. The aforementioned study showed that ART initiation was unacceptably low, with only 22% of HIV-positive men in the HIVST arm initiating ART at 6-months versus 75% of men in standard partner referral slip arm).[3] (See Figure 1). Poor rates of ART initiation are commonly reported across most HIVST studies, with ART initiation rates ~20-45%[7,12–14], although ART initiation is notoriously difficult to measure within HIVST strategies. A cost analysis for national scale-up of Index HIVST in Malawi showed that 76% of men tested must initiate ART for Index HIVST to be cost-neutral at the national level as compared to using partner referral slips.

Figure 1: *Male partner use of HIV services Index HIVST vs. referral slips (n=285) from HIVST trial (PI: Dovel)*

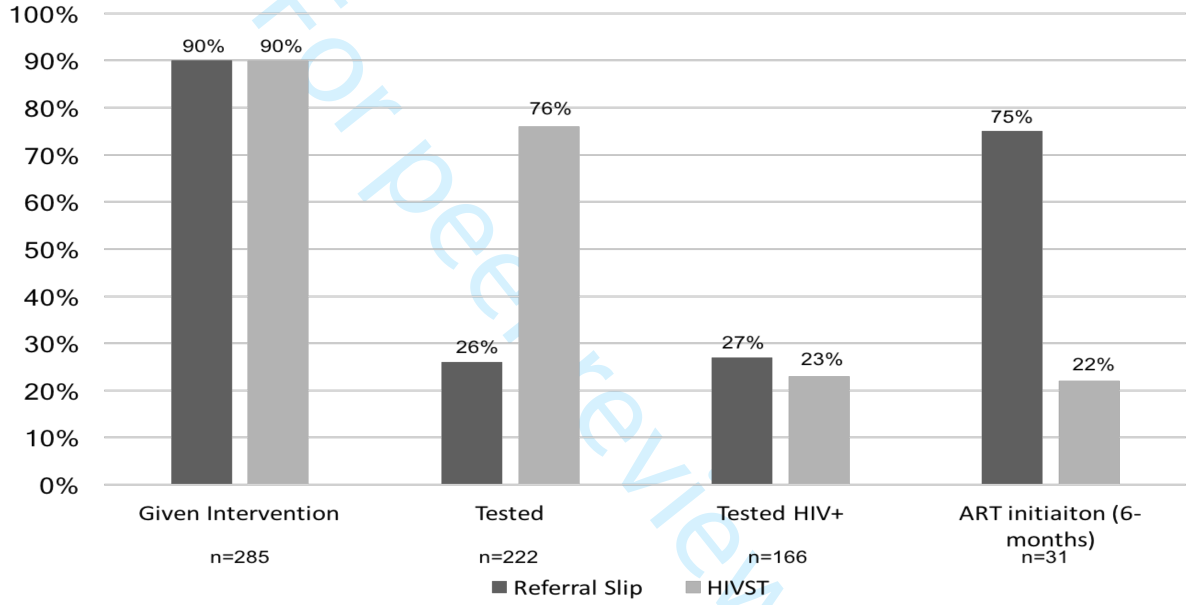

Two overarching barriers keep HIV-positive men from accessing ART services: (1) lack of male-friendly services;[15–17] and (2) harmful gender norms.[18–20] Male friendly services are private and convenient (requiring minimal time), and offered by health workers who understand the unique needs of men.[21] In addition, men are often unfamiliar with the health system, and are unsure how to navigate facility-based services. Gender norms that prioritize men as strong and self-reliant perpetuate fear of unwanted disclosure and stigma, and discourage men's engagement in ART.[18,21] Our research in Malawi found similar barriers to ART initiation for men who tested HIV-positive: men avoided ART services due to (1) fear of unwanted disclosure and stigma due to lack of privacy; (2) time/cost required to access care; (3) poor knowledge about the benefits of early ART initiation; and (4) beliefs that require men be strong, in control, and focused on short-term benefits such as daily financial earnings and respect from their male friends. Index HIVST must be combined with innovative ART interventions that address these barriers.

Evidence based for interventions that increase ART initiation

We have conducted a thorough search of the literature and have identified several intervention strategies that may increase ART initiation among men who use HIVST: (1) reminders + peer navigation; (2) motivational interviewing; and (3) home-based ART.

Reminders + Peer Navigation is shown to help clients overcome fears about facility-based services and

provide peer modeling how to live successfully with HIV.[22] While the strategy has been primarily tested within traditional HIV testing strategies, we hypothesize that the same mechanisms will work for men who test through Index HIVST. Reminders are usually done over the phone via phone calls or SMS and can vary in frequency based on the health care workers disgression. Peer Navigation is assisted guidance to the health clinic as well as overviews of where to go/what to do when at the facility once there to ensure men feel more comfortable in the clinic environment.

Motivational Interviewing is becoming widely recognized as a key strategy to help clients navigate barriers to the desired outcome by building client's self-efficacy, identifying internal motivation for the desired behavior, and establishing strategies and short- and long-term goals needed to reach a desired outcome.[8,23] Motivational interviewing is seen as particularly effective when clients need to make difficult decisions and overcome multi-level barriers to behavior change.[24,25] The strategy has been used to improve ART adherence[8,25] and reduce sexual risk behavior.[15]

In contrast, traditional counselling efforts are largely informational and directive, whereby health care workers deliver a pre-determined counseling package that is not responsive to a client's individual situation.[16,17] Such methods have been proven largely ineffective,[18] particularly with hard-to-reach populations such as men.[19] Motivational interviewing differs from traditional strategies by adopting a client-centered approach is based on collaboration, evocation and respect for autonomy. We hypothesize that these counseling techniques will encourage HIV status acceptance and disclosure, promote health seeking behavior, provide coping strategies men need to overcome barriers related to facility-based care, and ultimately, facilitate ART initiation.

Furthermore, motivational interviewing and client-centered care should resonate with and address the needs of men. Partners in Hope Malawi conducted 25 interviews with men and 6 focus group discussions with health care workers and female partners (n=42) to assess what health services men desired. Overwhelmingly, men reported wanting increased counseling on sexual health (including HIV) and marital concerns. Exit surveys with male ART clients (n=180) show that only 38% of men were aware of Treatment as Prevention and 65% aware of the benefits of early ART initiation, highlighting major knowledge gaps that may influence engagement in care. Motivational interviewing and client-centered counseling will be able to address both gaps in ART treatment and sexual health knowledge.

Home-based ART initiation has improved ART initiation across the region. A systematic review found that home-based ART is associated with ART retention, decreased mortality,[26] and in some cases, reduced stigma and increased privacy.[27,28] We conducted one of the only studies to examine home-based ART initiation within a community HIVST distribution strategy (Co-I: Choko). We found that home-based ART initiation alongside home-based HIVST significantly increased ART initiation as compared to standard facility-based initiation (RR 2.94; p-value<0.001).[13]

Home-based ART may be particularly attractive to hard-to-reach men because it reduces client time required to access services and provides an easy, opt-out entry point for men who otherwise may have never engaged with the health care system, or know how to navigate complicated, busy health facilities. Home-based ART has been associated with a three-fold reduction in financial costs to clients.[29] Further, home-based ART facilitates client-centered, one-on-one care that is often not feasible in busy clinic settings.

The Malawi Ministry of Health is in the process of rolling out community-based ART distribution strategies, and may consider home-based ART initiation for hard-to-reach populations. However, home-based ART is considered a resource intensive strategy, and therefore should (1) only be offered to the hardest-to-reach populations, and (2) requires that clients who initiate ART at home-based eventually link into facility-based care. Additionally, findings from a recent Index HIVST Trial show that home-based services for men is acceptable to female ART clients in the Malawian context, with minimal risk of adverse events. Over 90% of female ART clients had disclosed their HIV-status to their partner and were willing to have their male partners traced in their homes for additional services.

For peer review only

Finally, increased privacy and decreased wait-times are essential if men are to engage in HIV services. As part of a study on new Universal Treatment policies in Malawi, 15 in-depth interviews and 208 surveys were conducted with newly diagnosed HIV-positive men. Fear of unwanted disclosure due to limited privacy and a lack of trust in the health facility were the primary barriers to men's ART initiation. Home-based ART initiation can help address these barriers for ART initiation, and motivational interviewing can help provide men the skills needed to navigate these barriers within the health system in order to promote ART retention. Further, the vast majority (>95%) of men who used HIVST in the Index HIVST trial disclosed their HIV status to their female partner[8], meaning that home visits (i.e., reminders, peer navigation, motivational interviewing, or home-based ART) will not increase risk of unwanted disclosure to one's sexual partner. Table 2 below outlines how the proposed interventions will address barriers to ART initiaiton identified in the literature.

Table 2. *Levels and specific barriers to men's ART initiation, and intervention components*

Barriers to ART Initiation	Intervention Components	Level of Intervention
Unfamiliar with health system	Reminders and Peer Navigation	Health System
Belief about gender norms and focus on short-term benefits	Motivational Interviewing	Community/Individual
Poor knowledge		
Time/cost requirements	Home-Based ART Initiation	Health System
Lack of privacy/fear of disclosure		
Unfamiliar with health system		

4. METHODOLOGY

This study will be an individually randomized trial comparing three dfferent strategies to improve ART initiation and early retention among men who test HIV-positive with HIVST. Study staff will utilize the Minsitry of Health Index Testing Register and trace HIV positive women and their male partners to be screened and enrolled if they meet the inclusion criteria. Enrolled men will be randomized to one of three arms and will receive follow up and varying degrees of support based on the arm assigned. Outcomes will be assessed after 90 days after enrollment. Survey data, qualitative data, medical chart data (Objectives 1 and 2) and costing data (Objective 3) will be collected over years 1-3. A study flow chart is illustrated below (Figure 2).

Figure 2: *Study flow chart*

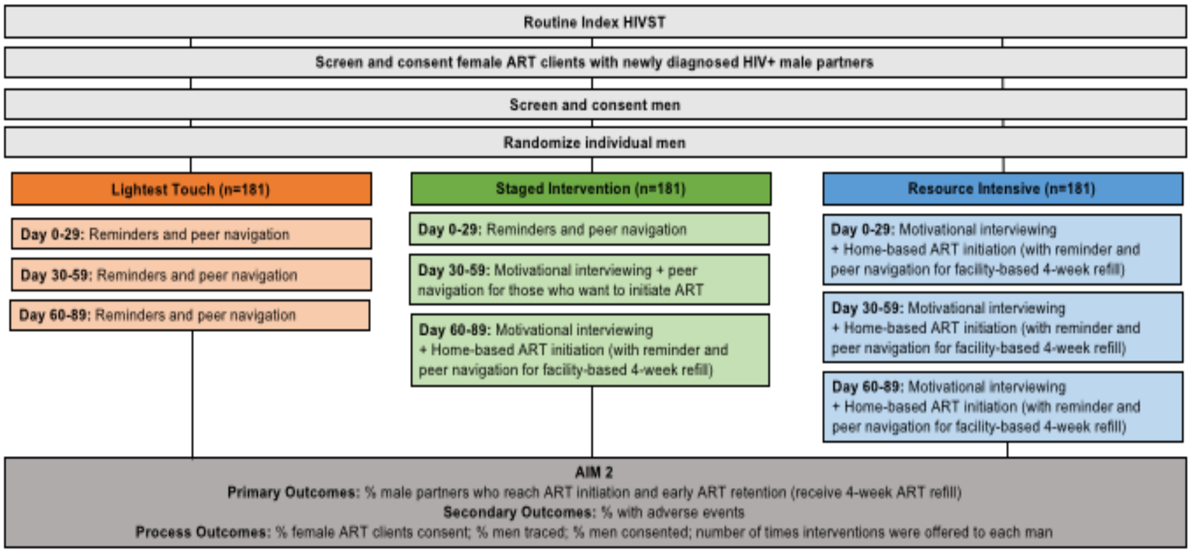

4.1. Intervention description

Each arm will offer an intervention immediately after study enrollment (that same day; day 0). Follow-up interventions will be offered every 14 days after that until 76 days or ART initiation is reached, whichever comes first.

Description of study arms:

Arm 1: "Lightest Touch" Intervention, whereby facility staff provide reminders and peer navigation (SMS and home visits) for men to encourage enrolment in facility-based ART programs. One reminder on the day of enrollment and every 14 days thereafter, until 76 days or ART initiation, whichever comes first. If initiation is not reached at 90 days, the patient will be classified as not initiated for study purposes. See Arm 1 diagram below:

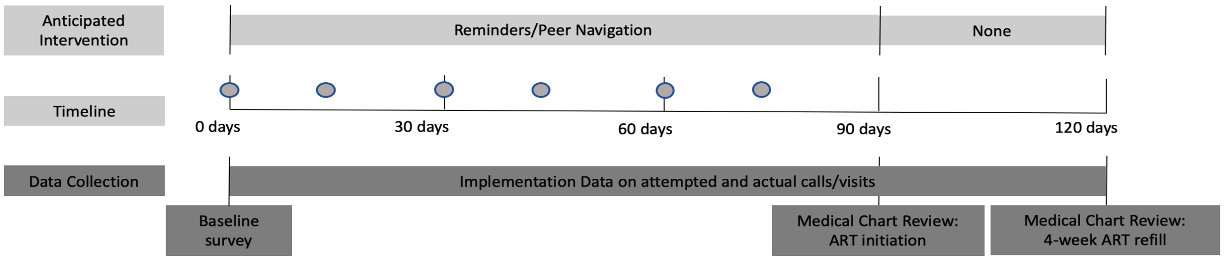

Fig 2. Timing of interventions to be offered in Aim 1

Legend:

Approximate time the intervention will be offered (approximately every 2 weeks until 76 days, or until ART initiation, whichever comes first

Arm 2: "Staged" Intervention, whereby the intervention will <u>build in intensity each month for those</u>

who have not initiated ART in the previous month, or for a maximum of 3 months, whichever comes first. The following intervention components that will be added each month (incrementally) until the first ART distribution is completed:

- Day 0-29: Reminders and, for those who agree to initiate, peer navigation;
- Day 30-59: Motivational interviewing and, for those who agree to initiate, peer navigation;
- Day 60-79: Motivational interviewing + home-based ART initiation and, for those who initiate, reminders and peer navigation for the facility-based 4-week ART refill appointment).

See Arm 2 diagram delow:

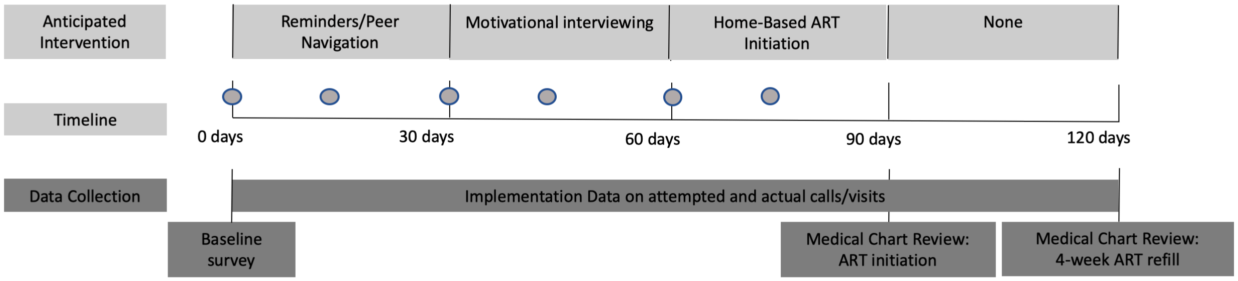

Fig 3. Timing of interventions to be offered in Aim 2

Legend:
Approximate time the intervention will be offered (approximately every 2 weeks until 76 days, or until ART initiation, whichever comes first

Arm 3: "Intensive" Intervention, whereby the most resource intensive intervention is offered immediately to all HIV-positive male partners. See Fig 4 for timeline. Components include:

- Motivational interviewing
- Home-based ART initiation
- 4-weeks after ART initiation: Reminders and peer navigation to facility for 4-week ART refill appointment

Home-based ART initiation will be scheduled at times convenient for men, including evening and weekend hours. Men who prefer to initiate in another private location in the community (besides their home) will be able to do so. The first home-visit will be conducted by a trained nurse and will include confirmatory HIV testing using Ministry of Health standard algorithm (Determine + Unigold), pre-ART counseling and motivational interviewing, a basic health evaluation, and ART initiation with a 30-day supply of first-line ART in Malawi – dolutegravir, tenofovir, and lamivudine as a single tablet. Clients will also be given a 30-day supply of cotrimoxazole, which is standard of care for all HIV-positive individuals.

Prior to ART initiation, a basic health evaluation will be performed by the nurse, including screening for tuberculosis with routine questions.[66] Any individual identified by the study nurse with concerns for an active opportunistic infection or other health problem(s) that could complicate home-based ART will be immediately referred and escorted to the facility.

At the same visit, motivational interviewing will be performed in preparation for men to engage in facility-based ART services. This includes counseling on the benefits of early ART, strategies for disclosure and positive living, strategies to overcome facility-based barriers to ART services, and addressing harmful gender norms that may discourage men from using care. Counseling will be adaptive to the needs and concerns of male clients. At 4-weeks after ART initiation, an expert client will escort the

review only

man to a nearby facility of his choice to join the facility-based ART cohort. Peer navigation will be provided to ensure men become familiar with the facility-based program. Men who wish to attend a facility that is not nearby will be linked with a male counselor from the selected facility. After completing all facility-based ART services for that day, the male partner will receive additional client-centered counseling with the same counselor to discuss the experience, benefits and challenges associated with facility-based ART, and strategies to overcome barriers. See Arm 3 diagram below:

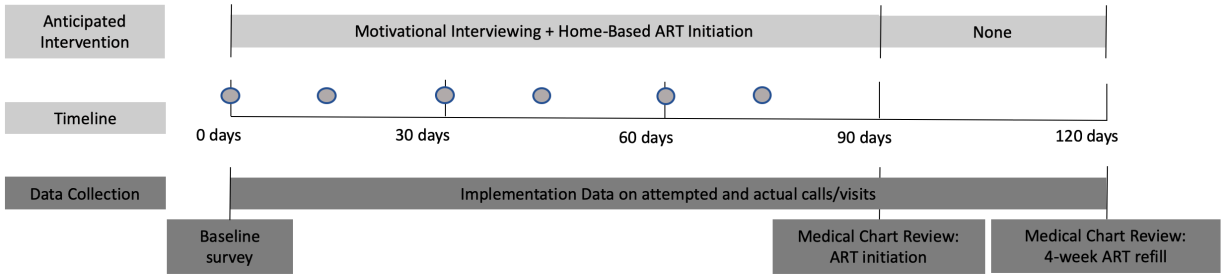

Fig 4. Timing of interventions to be offered in Aim 3

Legend:
Approximate time the intervention will be offered (approximately every 2 weeks until 76 days, or until ART initiation, whichever comes first

4.2. Place of study

All study activities will take place in 10 Partners in Hope supported facilities within the Lilongwe and Chikwawa districts, representing 19,198 adult female ART clients across all sites. These districts were chosen because they are priority districts for the Presidents Emergency Plan for AIDS Relief (PEPFAR) and have the highest HIV prevalence and unmet need of all Partners in Hope supported districts. See Table 3 for the selected 10 health facilities.

Table 3: *Selected study sites*

District	Facility name	ART Cohort Size
Chickwawa	St. Monftord Mission Hospital	3533
Chickwawa	Kalemba Community Hospital	2087
Chikwawa	Chickwawa District Hospital	4898
Kasungu	Kasungu District Hospital	5942
Lilongwe	Nkhoma Community Hospital	2166
Lilongwe	Mponela Rural Hospital	1099
Lilongwe	Daeyang Luke Hospital	1594
Nkhotakota	Nkhotakota District Hospital	5361

For peer review only - http://bmjopen.bmj.com/site/about/guidelines.xhtml

Nsanje	Nsanje District Hospital	3904
Nsanje	Ngabu Rural Hospital	1237

Specific methodology for each Objective is described below:

4.3. OBJECTIVE 1

Evaluate the effectiveness of the Staged ART Intervention vs Lightest Touch Intervention (primary analysis) and the effectiveness of the Staged ART Intervention vs Intensive Intervention (secondary analysis) on ART initiation within 4-months after enrolment in the trial.

- **Hypothesis 1.1:** 25% of men will initiate ART with simple reminders compared to 45% with motivational interviewing
- **Hypothesis 1.2:** 65% of men will initiate ART with home-based ART initiation compared to 45% with motivational interviewing

4.3.1. Study design

We will conduct an individually randomized controlled trial at 10 high-burden facilities in Malawi.

4.3.2. Target population

We will enroll 543 HIV-positive men identified through routine Index HIVST strategies and their female partners (1,086 participants total). While men are the primary focus of the study, female partners will be enrolled in order to understand their perception of their male partners use of ART services, acceptability of the intervention, and any unintended outcomes or adverse events.

Eligibility Critiera

Female ART clients will be enrolled in order to conduct baseline and follow-up surveys to understand their perception of their male partners use of ART services, acceptability of the intervention, and any unintended outcomes or adverse events.

Female Partner

- Inclusion criteria include: (1) client and partner are ≥15years of age; (2) partner lives in facility catchment area; (3) partner tested HIV-positive and has not initiated ART; and (4) ART client reports no interpersonal violence (IPV) as defined by WHO with their current sexual partner in the past 12 months.

- Exclusion criteria include: (1) client and partner are <15years of age; (2) partner does not live in facility catchment area; (3) partner has not tested HIV-positive or has testing positive and has initiated ART; and (4) ART client has reported interpersonal violence (IPV) as defined by WHO with their current sexual partner in the past 12 months.

Men will be enrolled as the primary recipient of the intervention.

Male partner

- Inclusion criteria include: (1) self and partner ≥15 years of age; (2) live in the facility catchment area (i.e., in the past 30 days, has spent ≥50% of all nights in the village); (3) has tested HIV-positive and has not initiated ART;

- Exclusion criteria include: (1) self and partner <15 years of age; (2) does not live in the facility catchment area (i.e., in the past 30 days, has spent <50% of all nights in the village); (3) has not tested HIV-positive or has tested HIV-positive and has initiated ART

4.3.3. *Sampling techniques and enrollment*

Sampling, screening, and enrolling male partners will be embedded within routine Index HIVST strategies.

Brief description of Routine MOH HIVST Guidelines

Briefly, routine Index HIVST includes three steps:

- *Identify ART clients with sexual partners in need of Index HIVST:* as outlined in the Malawi guidelines within the health care facility.
- *Distribute Index HIVST:* ART clients with a partner of unknown status will receive standard Index HIVST kit. They will be provided a demonstration, counseling, and an overview of risks and benefits.
- *Follow-Up on Index HIVST Use:* During their next ART appointments, ART clients are asked about HIVST distribution, use, and result of the HIVST kit, along with male partner linkage to a health facility for those who tested HIV-positive (i.e., confirmatory HIV testing and ART initiation). Data are documented in the Index HIVST register.

Once routine Index HIVST activities are completed, study recruitment and enrollment will commence. See Figure 3 for a complete description of particiant enrollment and study activities.

Figure 3: *Study screening and enrollment procedures flow chart*

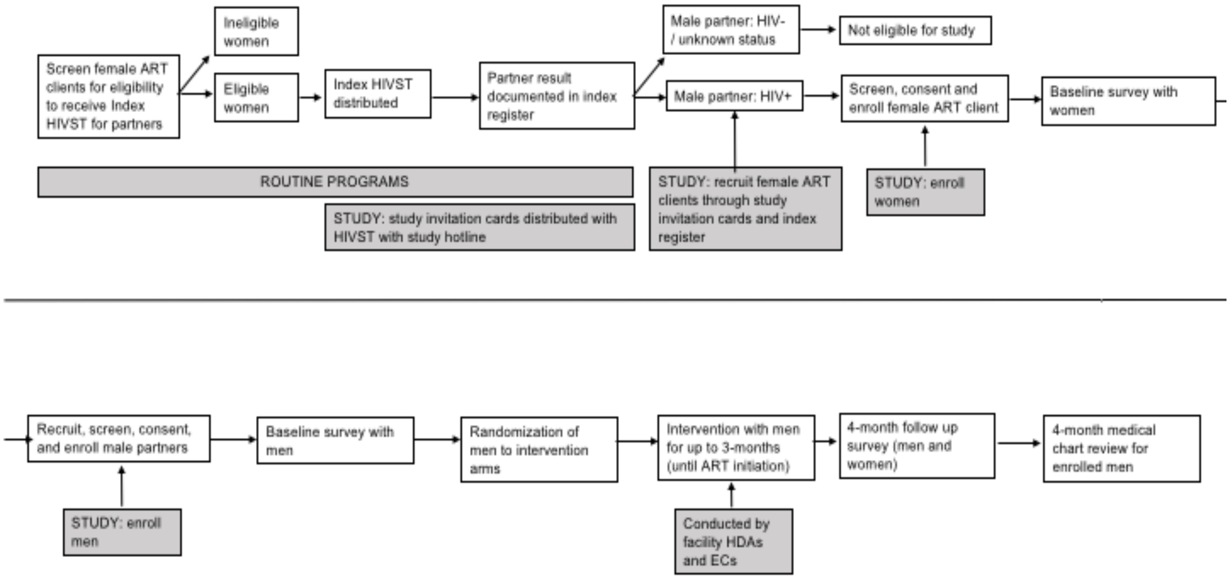

4.3.4. *Study Activites*

Female ART client recruitment

To maximize the number of male partners available for enrollment, the study team will introduce study invitation cards as part of Index HIVST activities and include them with HIVST kits distributed as a part of routine Index HIVST procedures (described above). Study invitation cards will include a 'hotline' phone number and will not provide any HIV specific information to ensure confidentiality. (see Appendix A for study invitation card). Female partners will be informed that should their male partner be found HIV-positive, they and their partner may be eligible for a study and should call the hotline number of the invitation card to receive more information.

In addition, the study team will review the Index Testing Register on a regular basis to identify female ART clients who reported HIV-positive male partners through routine Index HIVST procedures. Female ART clients identified will be contact by routine facility staff to be informed about the study and refered to study staff if interested in enrollment.

Female ART client enrollment

Female partners interested in the study will provide oral consent to participate in study screening and be screened for eligibility for the study (Appendix B). Study staff will conduct all consent and screening activities. If eligibile and willing to participant, written informed consent will be obtained (Appendix C). Female partners will be enrolled even if their male partner (1) does not consent to study participation (2) cannot be traced.

Upon enrollment in the study, female ART clients will work with study staff to make a plan for inviting male partners to enroll in the study. Baseline surveys with female ART clients will be conducted that same day or on a day convenient for the female ART client. Women whose partners agree to participate in the study will be followed up after 4-months for a follow up survey regarless of the outcome of their partner (ie loss-to-follow-up).

Male partner recruitment

Once female ART clients are enrolled, male partners will be recruited, screened, and consented. Study staff will work with the female ART client to establish a recruitment plan that is acceptable and feasible for the women. This may include women referring male partners to the study staff, study staff actively recruiting male partners based on information provided by the female client, or a joint approach where the study staff approaches male partners with the female ART client. Each female ART client enrollled in the study will be allowed to choose the recruitment strategy that best fits her individual situation and the needs of her partner.

Male partner enrollment

Men found eligibile and willing to participante, will provide written informed consent (Appendix D) and will be randomized to one of the three study arms. Randomization will occur using an electronic randomization system on tablet devices. A baseline survey will be concuted immediately following consent and the intervention will be carried out over the course of 3 months. Men will be contacted by the study staff 4-months after enrollment for a follow-up survey.

Randomization

Randomization will be conducted after completion of the baseline survey with male participants. Study staff will show randomization results as a picture on a pre-programmed tablet, allowing the participant to view the results themselves in order to maximize transparency and study buy-in. Male participants will be randomized to 1 of three arms and will be randomizied 1:1:1.

For peer review only

Intervention

Interventions will be offered immediately after study enrollment (that same day or the closest day that is convenient for the male participant). Follow-up interventions will be offered every 14 days after enrollment until 76 days after enrollment in the study or ARTinitiation is reached, whichever comes first. See a full description of the intervention in the Intervention Description section (4.1).

Intervention arms include:

1. ***Lightest touch arm:*** reminders and peer navigation to facility-based ART services
2. ***Staged arm:*** intervention builds in intensity each month for those who have not initiated ART in the previous month. Strategies include reminders and peer navigation, motivational interviewing, and home-based ART initiation
3. ***Intensive arm:*** home-based ART initiation + motivational interviewing + peer navigation to facility-based ART services for their 4-week follow-up appointment

Male expert clients and male nurses will complete all intervention activities to ensure it is as close to real-world implementation as possible (not implemented by Research Assistants). Research Assistants will support facility staff to ensure men are given the appropriate intervention (based on randomization arm), and that all staff activities related to the intervention are documented (i.e., how many reminders were given to each client, ect.). Intervention monitoring and evaluation tools will be developed and incorporated into the facility staff daily routine. Weekly reviews of all intervention monitoring tools and planning for the following week will be completed with study expert clients, nurses, and Research Assistant to ensure adherence to the study protocol. The Study coordinators and PI's will be highly involved throughout the implementation process to ensure protocol adherence.

4.3.5. Data collection techniques and tools

Data collection tools will include:

- *Baseline Survey:* Research assistants will administer baseline surveys with both female ART clients and male partners immediately following enrollment (before randomization). Surveys will collect data on male and female demographics, sexual partnerships and couple dynamics, and men's history with health services, and HIV services specifically. (Appendix E & F)

- *Follow-up Survey:* Research assistants will administer follow-up surveys with female ART clients and male participants 4-months after enrolment in the study. Follow-up surveys with men will assess the primary outcome of interest (ART initiation and completion of 4-week follow-up appointment), acceptability of the intervention, and any adverse events (ie., unwanted status disclosure). Men who cannot be reached will be counted as failures for true ART initiation. Follow-up survyes with female ART clients will assess acceptability of the intervention and any adverse events (i.e., IPV, end of the relationship, or unwanted disclosure) associated with intervention procedures. (Appendix G & H)

- *Medical Chart Reviews:* Identifiers will be collected for all men enrolled in the study, including name, age, village and address, and phone number. Identifiers will be used to conduct medical chart reviews at 4-months after enrollment as another measure of ART initiation (attendance to the 4-week follow-up ART appointment). Facility staff (established data clerks employed by Partners in Hope) will review medical records at study facilities and all other Partners in Hope

For peer review only

supported facilities within participating districts (61 facilities in total) to account for men who engage in ART outside study facilities. We successfully used this method in other HIVST studies to capture ART initiation.[3] Male partners who are not found in medical chart reviews will receive a follow-up home-visit to confirm ART outcomes through review of their individual medical record book (health passport) and self-reporting in the event that there are gaps in the record. Men who cannot be reached will be counted as failures for true ART initiation. (Appendix I)

- *Process Implementation Data:* Expert clients, nurses, and research assistants will keep daily logs as part of study monitoring and evaluation tools in order to assess the implementation of the intervention for each participant. Primary events to be recorded in the daily logs are: (1) unable to reach participant (and reason); (2) contacted participant; (3) intervention provided (and notes about the challenges and successes of the interaction; and (4) other comments relevant to intervention implementation. Each event will be recorded with a corresponding date.

Primary and secondary outcomes are measured through medical chart reviews and follow-up surveys (see Table 4).

Table 4: *Study Measures for Objective 1*

Outcome	Measurement	Source
Primary Outcomes		
Early ART Retention	Proportion of men who initiate ART at 3-months and attended their 4-week ART refill appointment at 4-months after enrollment	Medical chart review at 4-months
Secondary Outcomes		
ART initiation	Proportion of men who initiate ART at 3-months after enrollment	Medical chart review at 3-months
Adverse events by female ART client (IPV, unwanted disclosure, end of relationship) or male partner (unwanted disclosure)	Self-report from female ART client and their male partners who were identified as HIV-positive	Follow-up surveys at 4-months
Process Outcomes		
Proportion of female ART clients who consent	Proportion of eligible ART clients who consent to the participate in the study	Process implementation data
Proportion of men traced	Proportion of men who were successfully traced within 3-months after female ART client is enrolled	Process implementation data
Proportion of men who consented	Proportion of eligible men who consent to participate in the study	Process implementation data

- *Sensitivity analyses for men excluded from the trial***:** We recognize that men's consent to participate in the study may bias the sample enrolled in the study. There are two groups that we may not be able to include in the main study: (1) men we are unable to trace/contact (herein referred to as "unreachable men") and (2) men we are able to reach but who refuse to participate in the main trial (herein referred to as "male refusers"). We will take two approaches to address this potential bias
    - Unreachable Men*:* We will collect data on men who are unreachable via their female partner. Female partners for these men will complete a brief survey regarding Surveys will collect data on male and female demographics, sexual partnerships and couple dynamics, and men's history with health services, and HIV services specifically (as reported by female partners). We successfully used similar methods in the Index HIVST Trial.
    - Male Refusers*:* Men who are contacted but do not consent to the trial will be consented for a one-time survey immediately following refusal for the larger study. The same data will be collected, as described above.

4.3.6. *Sample size determination*

We powered the study to detect differences in ART initiation between Lightest Touch and Staged Interventions at 4-months after enrollment (primary outcome). We also assured we were powered to detect differences in ART initiation between Staged Interventions and the Intensive Intervention. We Assume that 25% of men in the Lightest Touch arm, 55% in the Staged Intervention arm, and 75% in the Resource Intensive arm initiate ART at 3-months and attend their 4-week follow-up appointment at 4-months. Any man lost to follow-up in any arm will be treated as failures for the outcome evaluation. The sample size needed to detect this difference with the power of 0.8 is 181 men per arm. The calculation is based on asymptotic normality of log odds ratio. We need to enroll and randomize a total of 543 HIV-positive men. Assuming that 25% of women have partners of unknown status, 65% of male partners will use the HIVST kit, 25% of them will be HIV-positive, and 80% of them will enroll in the study, we will need to screen over 3,000 women who were given index HIVST to reach the required sample size.

4.3.7. *Data analysis*

All randomized men will be included in the analysis of primary outcomes; men with missing outcome assessment due to loss to follow-up will be treated as outcome failures. All primary outcomes are binary; they will be analyzed by logistic regression models with intervention as a predictor, adjusted for baseline socioeconomic and demographic variables. We will conduct sensitivity analyses to account for men who we were never able to contact (unreachable men) and men who refused to participate in the full trial (refusers). We will run several analyses whereby the denominator includes (1) unreachable men and refusers; and (2) refusers.

4.4. OBJECTIVE 2

Identify individual-, community-, and facility-level factors associated with ART initiation within each intervention arm (Lightest Touch; Staged; and Intensive Interventions).

- **Hypothesis 2.1***:* In quanitative data, older men, men without strong social support networks, men with high levels of internalized and perceived HIV-related stigma, and men who hold rigid beliefs of gender norms and men's role as the provider and decision maker of the home will be less likely to initate ART.
- **Hypothesis 2.2***:* In qualitative data, primary factors influencing men's decision to start ART will be perceptions of feeling healthy, perceptions of one's ability to continue working and providing for their family without ART initiation, and perceptions of HIV-related stigma within one's community.

4.4.1. *Study design*

We will use baseline survey data from the randomized trial (Objective 1) to identify factors associated with ART initiation among men. We will also conduct 200 semi-structured in-depth qualitative interviews with a random sub-set of enrolled men (n=100) and their female partners (n=100) to assess in-depth characteristics of men who fail to engage in care, contextualize decisions around ART initiation and retention, and understand additional strategies that may be needed for male partners to successfully initiate and be retained in ART programs.

4.4.2. *Target population*

All men and women enrolled in the overarching trial will compelte a baseline survey. Eligibility criteria for study enrollment is described in detail under Objective 1.

A subset of men and women enrolled in the overarching trial will be randomly selected to complete an in-depth interview. Eligibility criteria for in-depth interview are as follows:

For peer review only

Male partners

- Inclusion criteria include: (1) randomly selected using electronic random selection techniques; (2) linked to care within 4-months after enrolling in the study (defined as completing the 4-week ART refill appointment) (n=50 respondetns); or (3) did not link to care within 4 months (n=50 respondents)
- Exclusion criteria include: (1) not randomly selected using electronic random selection techniques

Female partners

- Inclusion criteria include: (1) randomly selected using electronic random selection techniques; (2) partners could never be traced for study enrollment; or (3) partners were enrolled but were lost to follow up and unable to be reached again
- Exclusion criteria include: (1) not randomly selected using electronic random selection techniques; (2) partners could be traced for study enrollment; or (3) partners were enrolled and were not lost to follow up and were able to be reached again

4.4.3. Sampling techniques and tools

Survey data will include all men and women enrolled in the study (n=1,086; described in detail in Objective 1). 100 men (~ 33 per study arm) and 100 women (~ 33 per study arm) enrolled in the study will be randomly be selected for in-depth interviews.

4.4.4. Data collection techniques and tools

Surveys: Baseline and 4-month follow-up surveys will be conducted with men and women enrolled in the trial. They will focus on:

- *Health care system*: perceptions regarding the following aspects of ART services (i) privacy and confidentiality; (ii) availability of services; (iii) wait-time and distance to facility; (iv) quality of care and rude behavior from health care providers, using validated measures.
- *Sociodemographics*: (i) age; (ii) household assets; (iii) work; and (iv) substance use
- *Couple characteristics*: (i) relationship type and length; (ii) sexual activity and risk; (iii) frequency of communication; (iv) disclosure; (v) joint decision making using standard measures from Demographic Health Survey (DHS);[17] (vi) gender norms using validated Gender Equitable Men (GEM) Scale,[67] and (v) Revised Conflict Tactics Scale.[68]
- *Knowledge/perceptions and biomedical factors:* (i) knowledge about HIV and ART (treatment as prevention, benefits of early ART); (ii) risk perception (morbidity and mortality); standard DHS measures on (iii) previous use of HIV services and (iv) self-rated health;[17] and (v) WHO staging at enrollment.

In-depth interviews: Table 5 below describes in-depth interview participants and justification. In-depth interview guides for men and women are developed based on existing literature and our extensive experience conducting in-depth interviews with this population (Appendix J and K). Female partners will provide important insight into the circumstances of these men, and potential strategies to more effectively reach them. Men's qualitative feedback is particularly important for the staged intervention and intensive intervention since these are fairly novel and under explored.

For peer review only

Table 5. *Description of in-depth interview participants and justification*

Participant type	Number of interviews	Justification
Women whose male partners were **unreachable** during the study enrollment	50 (~ 16 per arm)	To understand the couple dynamics and characheristics of men who were never treaced and additional strategies to reaching these men
Women whose male partners were **loss-to-follow-up after** study enrollment	50 (~ 16 per arm)	To understand the couple dynamics and charactersitcs of men who were loss-to-follow-up, and additional strategies to better engage these men in care
Male particiapnts who **did not** complete 4-week ART refill appointments by 4-months	50 (~ 16 per arm)	To understand what they liked and did not like about the intervention, why they did not link to ART, and suggestions on how to improve the intervention
Male participants who completed 4-week ART refill appointments by 4-months	50 (~ 16 per arm)	To understand what they liked and did not like about the intervention, why they linked to ART, and suggestions on how to improve the intervention

4.4.5. Sample size determination

Survey sample size details provided in Objective 1. The number of interviews required for qualitative data can be challenging to predict. Data should be collected until saturation is reached, meaning that no new themes or relevant information is emerging. The exact number of interviews required to reach saturation differs based on the aim of the study, the diversity in respondents, and the theoretical framework used for analysis.[30] However, a basic rule of thumb is that no sample size should be under 25 participants in order to reach saturation and identify all relevant themes or new information important to the study.

4.4.6. Data analysis

Surveys: Subjects with complete data in outcomes as well as predictors will be included in the analysis. We will calculate descriptive statistics, including mean/median, variation (standard deviation, kurtosis), range, and frequency distributions for the demographic and clinical characteristics, overall and by study arm. Logistic models will be developed for the probability of a positive outcome, with sociodemographic factors included as covariates in a suitable form (linear/spline/factor). Differences in the prevalence of each of the outcomes of interest will be examined by study arm as well as by other factors of interest including demographic characteristics (e.g., age), couple chararisterics, and knowledge/perceptions and biomedical knowledge. The differences will be evaluated using t-tests, Mann-Whitney U test (or other non-parametric tests), chi-square methods, and Fisher's exact test as appropriate.

In-depth interviews: Audio recordings of in-depth interviews will be transcribed and translated to English. A preliminary codebook will be developed for both interview types (male and female). Selected investigators will piloted a codebook by independently reading and coding a randomly-selected subset of transcripts. Through an iterative consultative process, each investigator will revised their respective codebook and repeated this process until there was high interrater reliability among the group. All

For peer review only

transcripts will be coded in Atlas.ti v8.3 using constant comparison, and coding disagreements were resolved by consensus.

4.5. OBJECTIVE 3

Determine the cost-effectiveness and scalability of the intervention arms through costing and mathematical modeling.

- **Hypothesis 3.1:** The staged intervention will be more cost effective at having men initiate ART than both the lightest touch intervention and the intensive intervention.

4.5.1. Study design

We will conduct an incremental cost-effectiveness analysis and mathematical modelling to determine national scale-up potential. The average cost per successful outcome (early ART retention) will be calculated and compared across arms incrementally.

4.5.2. Data collection techniques and tools

Costs will be measured from the health care provider. We will use micro-costing methods by first creating an inventory of all the resources used to achieve the observed study outcomes including:

- Standard counseling interactions (staff cadre, training received, duration of interaction and distance from facility travelled where applicable)
- Motivational interviewing interactions (staff cadre, training received, duration of interaction and distance from facility travelled where applicable)
- Provider interactions (staff cadre, training received, duration of interaction and distance from facility travelled where applicable)
- Cost of reminder messages sent (when messages delivered telephonically instead of in person)

For each study patient, the quantity (number of units) of resources used will be determined. Unit costs of resources, which are not human subject data, will be obtained from external suppliers and the site's finance and procurement records and multiplied by the resource usage data to provide an average cost per study patient across centers in each study arm.

4.5.3. Data analysis

Cost-Effectiveness: Using the average cost per patient as described above, we will then estimate the cost per outcome achieved in each arm. The main measure of effectiveness for the cost-effectiveness analysis will be both the primary study outcome (early ART retention). We will calculate the difference in cost divided by the difference in effectiveness among study arms. Costs will be reported as means (standard deviations) and medians (IQRs) in USD, using the exchange rate prevailing during the follow up period.

National scale-up modeling: To determine the budget impact and affordability of the intervention arms, we will parameterize a national scale-up model using the study output. To determine the total cost and impact of the three intervention arms, as well as combinations of interventions, we will model cost and impact out to early ART retention (ART initiation and completion of the 4-week ART refill appointment). The following parameters to be estimated from this trial include:

- Percent of men not linking after HIVST (and thus eligible for this trial)

For peer review only

- Proportion of men that have not linked that could be reached
- Proportion of men that are known HIV-positive and on ART (not disclosed to their partner)
- Proportion of men that initiate ART
- Proportion of men that complete the 4-week ART refill appointment

We will then estimate the expected increase in the number of men linked to ART after index HIVST, adjusted by facility type where possible, by each intervention arm. The number of facility-level HIV tests conducted through index testing at all 652 public healthcare facilities in Malawi from Oct 2019-Sept 2020 will be used for these national calculations. Each intervention will be tested separately in this model, as well as different combination of interventions. Different scenarios will be explored where interventions are used at different facilities (urban versus rural targeting of interventions, geospatial targeting of interventions), or different groups of men within the same facility (where data suggest that different demographics of men resnd differently to the different interventions).

The national-level costs and expected number of men linked to ART, by each intervention and combinations of interventions, will be reported from this model. We will then contextualize the national cost of each intervention with a short-term 3-year budget impact: percent increase (or decrease) of the national HIV treatment budget with the inclusion of one of these interventions

5. ETHICAL CONSIDERATIONS

There is minimal risk associated with the above-mentioned procedures. We have extensive experience measuring ART initiation within HIVST studies. We conducted the first trials in the region to objectively measure ART initiation among men after receiving HIVST through the Index HIVST Trial (PI: Dovel) and PASTAL Trial (male partners of antenatal clients; PI: Choko). We draw from lessons learned from our previous trials.

Informed Consent

Informed consent will be obtained before any study-specific procedures are performed. The informed consent process will include information exchange, detailed discussion, and assessment of understanding of all required elements of informed consent, including the potential risks, benefits, and alternatives to study participation. The process will emphasize the randomized nature of the study and the differences that participants may experience as part of the study relative to current local standards of care. The study will include children 15 years of age and older. Following Malawian protocol, adolescents <18 years of age will be required to attain assent before completing the survey. Based on prior studies, we anticipate <10% of participants to be under 18 years of age, providing a small sample size to explore the potential impact of facility-based testing for youth.

Potential Benefits

Men who participate in the study may have access to additional HIV services not usually provided through routine care, such as appointment reminders, peer navigation, motivational interviewing, and home-based ART initiation. Men can refuse these additional services at any point. Further, both men and women will have the opportunity to discuss their use of HIV services and any concerns with HIV as individuals or as a couple. Information learned in this study may be of benefit to participants and others in the future, particularly information that may lead to optimized testing guidelines.

Potential risks and discomforts

Study procedures have minimal risk to the client. For men, maintaining privacy and confidentiality is a potential risk, particularly with home-based ART initiation. In our prior work delivering routine Index

For peer review only

HIVST, we have had health workers visit a cluster of homes (not just one) to avoid unwanted questions about the individual's serostatus. This has worked quite well, with no reports of unwanted disclosure, and we will use this approach in our proposed study to minimize risk of unwanted disclosure. Further, men may refuse any ART service at anypoint if they are uncomfortable.

For women, increased intimate partner violence (IPV) may be a potential risk, particularly if their male partner is prone to violence. To reduse these risks, women who report IPV with their current partner in the past 12 months will be excluded from the study. Female ART clients who report IPV at anypoint of the intervention will be withdrawn from the study, along with their male partner, counseled, and referred to community-based resources for IPV. We also will provide extensive counseling on status disclosure and an IPV hotline to all female participants. Further, Our PASTAL and Index HIVST Trials show no sign of increased IPV and we have published extensively on risk factors for IPV in other settings.[71-73]

Finally, Participation includes completion of a survey that will assess previous use of health services, perceptions of health services received, and sociodemographic and biomedical factors that may be associated with health service utilization. Participants may feel some psychological stress or discomfort from some of the questions, although most questions are not sensitive in nature. Participants may decline to answer any questions that make them uncomfortable and may end participation at any time.

Reimbursement/compensation

Participants will be provided MK 7,500 (equivalent to 10USD) for each survey completed (MK 15,000 / 20USD across the duration of the study). They will receive the above compensation regardless if they use HIV services or not. Those who complete the additional in-depth interview 6-months after study enrollment will receive an additional MK 7,500 (equivalent to 10USD) for their time.

Privacy and confidentiality

All study procedures will be conducted in private, and every effort will be made to protect participant privacy and confidentiality to the extent possible. Participant information will not be released without written permission to do so except as necessary for review, monitoring, and/or auditing. All study-related information will be stored securely. Participant research records will be stored in locked areas with access limited to study staff. All study data will be identified by participant ID (PID) only. Likewise, communications between study staff and protocol team members regarding individual participants will identify participants by PID only. Process evaluation documents, such as intervention monitoring and evaluation tools, will only include PID and will not store PID and identifiers together. All local databases will be encrypted and secured with password-protected access systems. Lists, logbooks, appointment books, and any other documents that link PID numbers to personal identifying information will be stored in a separate, locked location in an area with limited access. For the intervention, home visits will be conducted by health workers who visit a cluster of homes (not just one) at one time in order to avoid unwanted questions about the individual's serostatus. This has been used in other interventions focused on partner testing and treatment with high success of removing unwanted disclosure to community members.

6. DISSMINATION OF RESULTS

This study will set the stage for interventions that combine HIVST with differentiated models for early ART retention in low-resource settings. The study is timely and of high-impact. Findings will establish the effectiveness of home-based ART among male HIVST users, and can directly inform HIV programs throughout the region. The dissemination plan was developed to achieve the most impact while still ensuring dissemination among local stakeholders who may immediately benefit from study findings.

Partners in Hope is already integrated into national technical working groups, so dissemination will follow standard meeting schedules and draw upon Partners in Hope's longstanding history with the

For peer review only

Ministry of Health. Additionally, we will disseminate results through presentations at international scientific meetings and through high-impact peer-reviewed journals. The mentorship team has extensive experience publishing in high-impact journals (e.g., *AJPH, AIDS, BMJ, Lancet HIV, JAIDS, PLOS Med*)

7. PERSONNEL ROLES AND INSTITUTIONS

The proposed research team includes clinical researchers and implimentation science professionals with substantial experience in HIV testing, HIV prevention and treatment, cost effectiveness, differentiated care model studies, and male-focused studies and programs in Malawi and Sub-Saharan Africa. The study will be implemented in partnership with Partners in Hope Medical Center in Lilongwe, which has years of experience collaborating with Ministry of Health and local health facilities on similar studies, mentoring staff, and running studies embedded within routine clinical care.

- Kathryn Dovel, MPH, PhD, Principle Investigator, Division of Infectious Disease University of California Los Angeles (UCLA) and Research Director for Partners in Hope
- Thomas Coates, PhD, Co-Investigator, Division of Infectious Disease UCLA
- Risa Hoffman, MPH, MD, Co-Investigator, Division of Infectious Disease UCLA
- Brooke Nichols, Co-Investigator, School of Global Health, Boston Univeristy
- Lawrence Long, Co-Investigator, School of Global Health, Boston Univeristy
- Alemayehu Amberbir, Co-Investigator, Partners in Hope
- Augustine Choko, PhD, Site Co-Investigator, Malawi Liverpool Wellcome Trust
- Michal Kulich, Biostatistician, Charles Univeristy in Prauge
- Julie Hubabrd, MSc, Study Coordinator, Partners in Hope
- Kelvin Balakasi, Study Data Manager, Parners in Hope
- Khumbo Phiri, Implimentation Science Manager, Partners in Hope

Dr. Kathryn Dovel, the Principle Investigator, is the Science Director at Partners in Hope and an Assitant Assistant Professor in the Division of Infectious Diseases at UCLA. Dr. Dovel has over ten years of experience in Malawi and collaborating with the study team. She is regularly involved in UNAIDS and WHO workshops and meetings regarding strategies for male engagement, and has been a consultant on two Ministry of Health guidelines on the topic in Malawi.

Dr. Augustine Choko will be responsible with Dr. Dovel for overall adherence to the study protocol and serve as the primary liaison with the local IRB and key stakeholders in Malawi. Dr. Thomas Coates will serve as the community-based trials specialist, with over two decades of experience conducting individual- and cluster-randomized trials in communities with the end goal of engagement in HIV services. Dr. Risa Hoffman is an established clinical investigator and will serve as the MD specializing in differentiated models of ART treatment delivery and HIV care, and ensuring client safety. Brooke Nichols and Lawrence Long will be responsible for reviewing all modeling data, making an analysis plan for the proposed models, and providing modeling for publications. Dr. Michal Kulich is the Chair of the Probability and Statistics Department at Charles University and has extensive experience with the design, conduct, and analysis of clinical trials in the context of HIV prevention research.

Partners in Hope's staff Kelvin Balakasi (Data Manager) Julie Hubbard (Research Coordinator), Khumbo Phiri (Implimentation Science Manager) and Alemayehu Amberbir (Science Director) will be responsible implimentation and oversight inlcuidng data collection, data management, quality control, and training and certification of data entry personnel. They will also be responsible for ensuring the intervention promotes client safety, meets Ministry of Health guidelines, and is implemented in such a way to promote sustainability and scalability.

CV's for participating personelle are provided in the Appendix L.

For peer review only

8. REGULATORY OVERSIGHT

This study is sponsored by the Bill and Melinda Gates Foundation and implemented through Partners in Hope (PIH), Malawi. PIH staff will perform monitoring visits. As part of these visits, monitors will inspect study-related documentation to ensure compliance with all applicable regulatory requirements.
All health facilities will receive an Initial Registration Notification from PIH that indicates successful completion of the protocol registration process. A copy of the Initial Registration Notification will be retained in the site's regulatory files.

We have developed a trial advisory group. See Table 6 for details about the group members. The group will meet every quarter to review progress, and challenges with study implementation, and provide input on the final interventions to be tested, based on qualitative findings in Aim 1.

Table 6. *Description of trial advisory group*

Name	Affiliation	Expertise
Dr. Morna Cornell	University of Cape Town	Epidemiologist, health system barriers to men's care, men's HIV services, advocacy and policy change
Dr. Heidi van Rooyen	SA Human Sciences Research Council	Social scientist, HIV vulnerability and inequality, interventions for men's ART initiation
Dr. Deborah Donnell	University of Washington, Fred Hutch Vaccine and Infectious Disease Division	Biostatistician, international HIV trials, PI of the HPTN Statistical and Data Management Center
Dr. Connie Celum	University of Washington	Infectious disease physician and epidemiologist, implementation science in Africa, HIV prevention trials
Dr. Thoko Kalua	Malawi Ministry of Health, Deputy Director at Department of HIV and AIDS	Epidemiologist. Extensive experience in national HIV programs, M&E, and scale-up of interventions on the ground
Dr. Sergio Chicumbe	Mozambique National Health Institute (INS), Health System Research Cluster	Clinical trials and implementation science. Extensive experience in national public health programs, methodology for health services research and quality care improvement.

For any future protocol amendments, upon receiving final IRB/EC and any other applicable regulatory entity approvals, sites should implement the amendment immediately. Sites are required to submit an amendment registration packet to the PIH Protocol Team. PIH key personnel will review the submitted protocol registration packet to ensure that all the required documents have been received.

9. STUDY IMPLEMENTATION

Study implementation at each site will be guided site-specific standard operating procedures (SOPs). These SOPs will be updated and/or supplemented as needed to describe roles, responsibilities, and procedures for this study.

10. PROTOCOL DEVIATION REPORTING

All protocol deviations will be documented in participant research records. Reasons for the deviations and corrective and preventive actions taken in response to the deviations will also be documented. Deviations will be reported to site IRBs/ECs and other applicable review bodies in accordance with the policies and procedures of these review bodies. Serious deviations that are associated with increased risk to one or more study participants and/or significant impacts on the integrity of study data must also be reported to the Protocol Team as soon as possible.

11. WORK PLAN TIMELINE

Table 7: *Anticipated workplan timeline of study activities, by year*

IdeAL Study Timeline																
**Activities**	**Year 1**				**Year 2**				**Year 3**				**Year 4**			
Quarter	1	2	3	4	1	2	3	4	1	2	3	4	1	2	3	4
Preparation	X	X	X	X												
Objective 1:3. Test the effectiveness of models of varying intensity to link patients who use HIVST to care																
Enrollment					X	X	X	X								
Intervention and follow-up					X	X	X	X	X	X						
Follow-up analyses & writing										X	X	X	X			
Objective 2: 4. Identify factors associated with early ART retention																
Baseline sociodemographic survey					X	X	X	X								
Qualitative assessment- rolling					X	X	X	X	X	X						
Analyses & writing						X	X			X	X	X	X			
Objective 3: 5. Determine the cost and scalability of the intervention																
Data collection					X	X	X	X	X	X						
Synthesis of results and parameter estimation										X						
Model development										X	X	X				
Analyses & writing										X	X	X	X			

12. BUDGET AND JUSTIFICATION

Table 8: *Study budget*

Description	USD	Justification
Study Coordinator	5000	25% LOE to coordinate RAs
Research Assistant for data collection	15000	5 RAs for 6 months at 500 USD per month
Incentive for participants	21720	10USD per study visit, 2 study visits per participant, 1086 participants (543 men and 543 female partners)

For peer review only

Expert Client/Nurse lunch allowance	720	6USD lunch allowance for 10 Expert Clients and 10 nurses on a monthly basis to hear from them how the intervention is going
Telecomunications	1000	Mobile data collection processing by RAs and communication with coordinator
NHSRC application fee	150	Application fee
Sub Total	*43,590*	
NHSRC 10% fee	4,359	10% contribution fee of study budget
**Grand total**	**47,949**	

For peer review only

13. REFERENCES

1. Dovel K, Yeatman S, Watkins S, Poulin M. Men's heightened risk of AIDS-related death: the legacy of gendered HIV testing and treatment strategies. *AIDS (London, England)*. 2015;29(10):1123.

2. UNAIDS. *Ending AIDS: Progress towards 90-90-90 Targets*.; 2017.

3. Choko AT, Kumwenda MK, Johnson CC, et al. Acceptability of woman-delivered HIV self-testing to the male partner, and additional interventions: a qualitative study of antenatal care participants in Malawi. *Journal of the International AIDS Society*. 2017;20(1):21610.

4. Yeatman S, Chamberlin S, Dovel K. Women's (health) work: A population-based, cross-sectional study of gender differences in time spent seeking health care in Malawi. *PloS one*. 2018;13(12).

5. Mills EJ, Beyrer C, Birungi J, Dybul MR. Engaging men in prevention and care for HIV/AIDS in Africa. *PLoS medicine*. 2012;9(2).

6. Malawi National Statistics Office. *Demographic Health Survey: Malawi*.; 2016.

7. Sharma M, Ying R, Tarr G, Barnabas R. Systematic review and meta-analysis of community and facility-based HIV testing to address linkage to care gaps in sub-Saharan Africa. *Nature*. 2015;528(7580):S77–S85.

8. Partners in Hope. Update on index partner testing in Partners in Hope supported sites: quarterly review. Presented at the: Malawi Ministry of Health Technical Working Group: HIV Self-Testing; 2019.

9. Dovel K, Balakasi K, Shaba F, Phiri K, Offorjebe O, Gupta S. A Randomized trial on index HIV self-testing for partners for ART clients in Malawi. In: *Conference on Retroviruses and Opportunistic Infections, Seattle*. ; 2019.

10. Johnson CC, Kennedy C, Fonner V, et al. Examining the effects of HIV self-testing compared to standard HIV testing services: a systematic review and meta-analysis. *Journal of the International AIDS Society*. 2017;20(1):21594.

11. Masters SH, Agot K, Obonyo B, Mavedzenge SN, Maman S, Thirumurthy H. Promoting partner testing and couples testing through secondary distribution of HIV self-tests: a randomized clinical trial. *PLoS medicine*. 2016;13(11).

12. Ortblad K, Musoke DK, Ngabirano T, et al. Direct provision versus facility collection of HIV self-tests among female sex workers in Uganda: a cluster-randomized controlled health systems trial. *PLoS medicine*. 2017;14(11).

13. MacPherson P, Lalloo DG, Webb EL, et al. Effect of optional home initiation of HIV care following HIV self-testing on antiretroviral therapy initiation among adults in Malawi: a randomized clinical trial. *Jama*. 2014;312(4):372–379.

14. Sibanda EL, Weller IV, Hakim JG, Cowan FM. The magnitude of loss to follow-up of HIV-exposed infants along the prevention of mother-to-child HIV transmission continuum of care: a systematic review and meta-analysis. *AIDS (London, England)*. 2013;27(17):2787.

For peer review only - http://bmjopen.bmj.com/site/about/guidelines.xhtml

15. Larsson EC, Thorson A, Nsabagasani X, Namusoko S, Popenoe R, Ekström AM. Mistrust in marriage-Reasons why men do not accept couple HIV testing during antenatal care-a qualitative study in eastern Uganda. *BMC public health*. 2010;10(1):769.

16. Koo K, Makin JD, Forsyth BW. Barriers to male-partner participation in programs to prevent mother-to-child HIV transmission in South Africa. *AIDS Education and Prevention*. 2013;25(1):14–24.

17. Theuring S, Nchimbi P, Jordan-Harder B, Harms G. Partner involvement in perinatal care and PMTCT services in Mbeya Region, Tanzania: the providers' perspective. *AIDS care*. 2010;22(12):1562–1568.

18. Sileo KM, Fielding-Miller R, Dworkin SL, Fleming PJ. What role do masculine norms play in men's HIV testing in sub-Saharan Africa?: a scoping review. *AIDS and Behavior*. 2018;22(8):2468–2479.

19. van den Berg W, Brittain K, Mercer G, et al. Improving men's participation in preventing mother-to-child transmission of HIV as a maternal, neonatal, and child health priority in South Africa. *PLoS medicine*. 2015;12(4).

20. Unaids HIV. *Addressing a Blind Spot in the Response to HIV—Reaching out to Men and Boys*. Joint United Nations Programme on HIV/AIDS Geneva; 2017.

21. Cornell M, Cox V, Wilkinson L. Public health blindness towards men in HIV programmes in Africa. *Tropical Medicine & International Health*. 2015;20(12):1634–1635.

22. Poulin M, Dovel K, Watkins SC. Men with money and the "vulnerable women" client category in an AIDS epidemic. *World Development*. 2016;85:16–30.

23. Pai NP, Sharma J, Shivkumar S, et al. Supervised and unsupervised self-testing for HIV in high-and low-risk populations: a systematic review. *PLOS medicine*. 2013;10(4).

24. Sharma M, Barnabas RV, Celum C. Community-based strategies to strengthen men's engagement in the HIV care cascade in sub-Saharan Africa. *PLoS medicine*. 2017;14(4).

25. Choko AT, MacPherson P, Webb EL, et al. Uptake, accuracy, safety, and linkage into care over two years of promoting annual self-testing for HIV in Blantyre, Malawi: a community-based prospective study. *PLoS medicine*. 2015;12(9).

26. Decroo T, Rasschaert F, Telfer B, Remartinez D, Laga M, Ford N. Community-based antiretroviral therapy programs can overcome barriers to retention of patients and decongest health services in sub-Saharan Africa: a systematic review. *International health*. 2013;5(3):169–179.

27. Wools-Kaloustian KK, Sidle JE, Selke HM, et al. A model for extending antiretroviral care beyond the rural health centre. *Journal of the International AIDS Society*. 2009;12(1):22–22.

28. Apondi R, Bunnell R, Awor A, et al. Home-based antiretroviral care is associated with positive social outcomes in a prospective cohort in Uganda. *JAIDS Journal of Acquired Immune Deficiency Syndromes*. 2007;44(1):71–76.

29. Jaffar S, Amuron B, Foster S, et al. Rates of virological failure in patients treated in a home-based versus a facility-based HIV-care model in Jinja, southeast Uganda: a cluster-randomised equivalence trial. *The Lancet*. 2009;374(9707):2080–2089.

For peer review only

30. Malterud K, Siersma VD, Guassora AD. Sample size in qualitative interview studies: guided by information power. *Qualitative health research*. 2016;26(13):1753–1760.

For peer review only

14. APPENDIX

14.1. APPENDIX A – Study Invitation Cards

STUDY INVITATION CARD
Identifying efficient linkage strategies for HIV self-testing (IDEaL)
English

Study invitation Card

Date: ……/……/2020

Dear: ____

At __________________________________, we are conducting a study. The study will generate information that will help the Malawi government, Partners in Hope and other stakeholders develop programs that will help your community lead a healthy life. We are therefore inviting you and your partner to come to ___________________________ on___________________________ at_________, so that we can assess if you are eligible for the study. If eligible, you and your partner will each receive MK 7,500 (equivalent to 10USD).

Thank you for your kind attention to this request and we look forward to seeing you. When coming call or flash this number…………………… so that you are met by study personnel.

Sincerely,

[District Health Officer]

For peer review only

STUDY INVITATION CARD
Identifying efficient linkage strategies for HIV self-testing (IDEaL)
Chichewa

Khadi Lokuyitanani Ku Kafukufuku

Tsiku: ……/……/2020

Wokondedwa: ____

Ku chipatala cha, _______________________________________, tikupanga kafukufuku. Kafukufukuyu adzathandiza kupeza mfundo zomwe zidzathandize boma la Malawi, bungwe la Partners In Hope ndi mabungwe/magulu ena kukhazikitsa ndondomeko zomwe zidzathandizire anthu a mdela lanu kukhala ndi umoyo wanthanzi. Choncho mukuyitanidwa pamodzi ndi okondedwa anu kuti mubwere ku_________________ tsiku nthawi ya _____________, ndicholinga choti tidzaone ngati muli oyenera kutenga nawo mbali mu kafukufukuyu. Mukadzapezeka kuti ndinu oyenera kutenga nawo mbali mu kafukufukuyu, inuyo komanso bwenzi lanu mudzalandira K7,500 (yokwana 10USD) aliyense.

Zikomo kwambiri chifukwa cha chidwi chanu pa pempholi ndipo ife tikuyembekezera kuzaonana nanu pa tsikuli. Mukamadzabwera, muzaimbe kapena kufulasha pa nambala iyi…………………………………kuti muzakumane ndi anthu a kafukufukuyu.

Ndine wanu,

[District Health Officer]

For peer review only

14.2. APPENDIX B – Recruitment and Screening Script

RECRUITMENT AND SCREENING SCRIPT
Identifying efficient linkage strategies for HIV self-testing (IDEaL)
Female

Thank you for speaking with me about the study entitled, *"Identifying efficient linkages strategies for HIVST"* conducted by Partners in Hope and the University of California Los Angeles in the United States. You are being approached because you recently reported that your male partner tested HIV-positive using a HIV self-test kit.

The purpose of this is to determine what are the best interventions that can help men who are diagnosed with HIV use other health services, if desired. The study will offer several different strategies for HIV services to see what works best for men who use HIV self-testing kits.

Would you be interested in participating in the eligibility screening to see if you are eligible to participate in the study? Your participation is voluntary and you will not be penalized if you choose not to participate in the screening or the project.

[*If no, thank the person and end the session*]

[*If yes, continue to the screening questions below or make an appointment to complete the screening questions*]

Before we continue with the study, we first need to determine if you are eligible to participate.

1. Are you and your partner 15 years old or older?
2. To the best of your knowledge, did your partner recently test HIV-positive using a HIV self-test kit?
3. To the best of your knowledge, your partner currently NOT taking ART?
4. Does your partner live in the facility catchment area?
5. In the last 30 days, your current partner has NEVER hit, slapped, or kicked you, or forced you to have sexual intercourse with them?

If you answered yes to all these questions, then you are eligible to participate in the study. You may stay here to continue with the study consent, and we will explain how the study will be conducted.

For peer review only

RECRUITMENT AND SCREENING SCRIPT
Identifying efficient linkage strategies for HIV self-testing (IDEaL)
Female (Chichewa)

Zikomo kwambiri povemera kucheza nane zokhudza study yotchedwa "Kupeza njira zabwino zothandizira anthu omwe akulandira ma ARV" amene akupangidwa ndi bungwe la Partners In Hope-EQUIP polojekiti ndi sukulu ya ukachenjede ya University of California Los Angeles yaku Amerika. Mukufunsidwa kuti mutenge nawo mbali mu gawo lakafukufukuyu chifukwa bwenzi lanu lalimuna lili ndi kachirombo ka HIV ndipo linadziwa zotsatirazi pogwiritsa ntchito ka chida koziyezera wekha.

Cholinga cha kafukufukuyu ndi kufuna kupeza njira zabwino zomwe zingathandize azibambo omwe ali ndi kachirombo ka HIV kugwiritsa ntchito thandizo lina la zaumoyo, ngati akonda kutero. Studyyi idzapeleka njira zingapo zosiyanasiyana za thandizo la HIV kuti awone njira yomwe ikugwira bwino kwa azibambo omwe amagwiritsa ncthito ka chida koziyezera wekha HIV.

Kodi muli okondwa kutenga nawo mbali mu mayele ofuna kuwona ngati muli oyenela kutenga nawo mbali mu study? Kutenga nawo mbali kwanu ndi kosakakamiza ndipo palibe chilango chilichonse ngati mungasankhe kusatenga nawo mbali mu mayele a study.

[Ngati ayi, thokozani munthuyo ndipo malizani session]

[Ngati eya, pitilizani kufunsa mafunso a mayele omwe ali munsiwa kapena sankhani tsiku loti muzamalize kufunsa mafunso]

Tisanapitilize ndi study, choyamba tifuna tidziwe ngati muli oyenera kutenga nawo mbali.

1. *Muli ndi zaka khumi ndi zisanu kapena kuposela apo?*
2. *Monga mukudziwira kodi bwenzi lanu laziyezera kachida koziyezera wekha ndikupezeka ndi kachirombo ka HIV?*
3. *Monga mene mukudziwira, kodi pakadali pano bwenzi lanu likumwa mankhwala a ma ARV?*
4. *Kodi bwenzi lanu limakhala mu dela lozungulira chipatala?*
5. *M'masiku makumi atatu apitawa, bwenzi lanu SILINAPANGE izi kumenyani kapena kukukakamizani kuti mugonane nalo?*

Ngati mwayankha eya pa mafunso onse mwafunsidwawa, zikusonyeza kuti ndinu oyenera kutenga nawo mbali mu study. Muli omasuka kukhala ndikupitiliza chilolezo cha study, ndipo ndikufotokozerani za momwe study ichitikire.

For peer review only

RECRUITMENT AND SCREENING SCRIPT
Identifying efficient linkage strategies for HIV self-testing (IDEaL)
Male

Thank you for speaking with me about the study entitled, *"Identifying efficient linkages strategies for HIVST"* conducted by Partners in Hope and the University of California Los Angeles in the United States. You are being approached because you recently reported testing HIV-positive using a HIV self-test kit.

The purpose of this is to determine what are the best interventions that can help men who are diagnosed with HIV use other health services, if desired. The study will offer several different strategies for HIV services to see what works best for men who use HIV self-testing kits.

Would you be interested in participating in the eligibility screening to see if you are eligible to participate in the study? Your participation is voluntary and you will not be penalized if you choose not to participate in the screening or the project.

[*If no, thank the person and end the session*]

[*If yes, continue to the screening questions below or make an appointment to complete the screening questions*]

Before we continue with the study, we first need to determine if you are eligible to participate.

1. Are you 15 years old or older?
2. You recently test HIV-positive using a HIV self-test kit?
3. You are NOT currently taking ART?
4. Do you live in the facility catchment area?

If you answered yes to all these questions, then you are eligible to participate in the study. You may stay here to continue with the study consent, and we will explain how the study will be conducted.

For peer review only

RECRUITMENT AND SCREENING SCRIPT
Identifying efficient linkage strategies for HIV self-testing (IDEaL)
Male (Chichewa)

Zikomo kwambiri povemera kucheza nane zokhudza study yotchedwa "Kupeza njira zabwino zothandizira anthu omwe akulandira ma ARV" amene akupangidwa ndi bungwe la Partners In Hope-EQUIP polojekiti ndi sukulu ya ukachenjede ya University of California Los Angeles yaku Amerika. Mukufunsidwa kuti mutenge nawo mbali mu gawo lakafukufukuyu chifukwa muli ndi kachirombo ka HIV ndipo munadziwa zotsatirazi pogwiritsa ntchito ka chida koziyezera wekha.

Cholinga cha kafukufukuyu ndi kufuna kuoeza njira zabwiino zomwe zingathandize azibambo omwe ali ndi kachirombo ka HIV kugwiritsa ntchito thandizo lina la zaumoyo, ngati akonda kutero. Studyyi idzapeleka njira zingapo zosiyanasiyana za thandizo la HIV kuti awone njira yomwe ikugwira bwino kwa azibambo omwe amagwiritsa ncthito ka chida koziyezera wekha HIV.

Kodi muli okondwa kutenga nawo mbali mu mayele ofuna kuwona ngati muli oyenela kutenga nawo mbali mu study? Kutenga nawo mbali kwanu ndi kosakakamiza ndipo palibe chilango chilichonse ngati mungasankhe kusatenga nawo mbali mu mayele a study.

[Ngati ayi, thokozani munthuyo ndipo malizani session]

[Ngati eya, pitilizani kufunsa mafunso a mayele omwe ali munsiwa kapena sankhani tsiku loti muzamalize kufunsa mafunso]

Tisanapitilize ndi study, choyamba tifuna tidziwe ngati muli oyenera kutenga nawo mbali.

1. *Muli ndi zaka khumi ndi zisanu kapena kuposela apo?*
2. *Kodi mwaziyezera kachida koziyezera wekha ndikupezeka ndi kachirombo ka HIV?*
3. *Kodi pakadali pano mukumwa mankhwala a ma ARV?*
4. *Kodi mumakhala mu dela lozungulira chipatala?*

Ngati mwayankha eya pa mafunso onse mwafunsidwawa, zikusonyeza kuti ndinu oyenera kutenga nawo mbali mu study. Muli omasuka kukhala ndikupitiliza chilolezo cha study, ndipo ndikufotokozerani za momwe study ichitikire.

For peer review only

14.3. APPENDIX C – Written Informed Consent- Female

WRITTEN INFORMED CONSENT
Identifying efficient linkage strategies for HIV self-testing (IDEaL)
Female

You are asked to participate in a research study entitled *"Identifying efficient linkages strategies for HIVST"* conducted by Partners in Hope and the University of California Los Angeles in the United States. You are being requested to take part in the study because you recently reported that your male partner tested HIV-positive using a HIV self-test kit. Your participation in this study is entirely voluntary. You will be read the information below, and you are free to ask questions about anything you do not understand, before deciding whether or not to participate. ***I as the field assistant for this study will take you through this consenting process.***

- **Why is this study being done?**

HIV self-testing is very helpful for people who want to know their status but do not usually go to the health facility. However, it can be hard for individuals who test HIV-positive with HIV self-testing to be able to access other health services. Researchers want to determine what are the best interventions that can help men who are diagnosed with HIV use other health services, if desired. The study will offer several different strategies for HIV services to see what works best for men who use HIV self-testing kits.

- **What will happen if you take part in this research study?**

There are several steps to this study. if you volunteer to participate in this study you will have the opportunity to participate in the following components:

1. Allow me to trace your male partner, or take me to your male partner in order to invite him to participate in the study as well. Note, you can choose to participate in the study even if your partner refuses or you think your partner would refuse.

2. Complete one or two study visits where a research assistant like myself will interview you and ask you information about yourself, including whether you are married, number of sexual partners, your level of education, information about your experiences with HIV services, and how you feel about HIV testing and treatment services. We will ask you questions today (or a day nearby that is convenient for you) and, if your partner enrolls in the study, we will ask you similar questions again in four months in order to see if anything has changed. Each interview will last about 45minutes. You can refuse a follow-up survey at any point

For peer review only

3. If your partner agrees to participate in the study, he will be randomized to one of three interventions. We will do the randomization together with him so he can see exactly what intervention he will be offered. The potential interventions are:

    1) Standard of care where providers may send him reminders about the benefits of health services.
    2) Motivational Interviewing where he can talk to someone about his life, challenges he faces, and strategies to make his life better and additional services as needed.
    3) Home-based health services whereby a provider will offer him HIV services and NCD screening at your home as a one-time event. He will then be visited after 4-weeks to be escorted to the clinic if desired.

    Regardless of what arm your partner is randomized to, he can always refuse health services or refuse talking to a health care provider and still remain in the study. You can remain in the study regardless of what your partner does.

4. Finally, you may be randomly selected to within 6-months of the study to complete a 1-hour in-depth interview so we can learn more about your experiences in the study. Not all participants will be contacted for the interview and you always have the right to decline an in-depth interview – refusal will not affect your participation in the larger study.

- **How long will you be in the research study?**

All study activities will be completed within 6-months of today.

- **Are there any potential risks or discomforts that you can expect from this study?**

You will be asked a series of questions by a research assistant about your sexual relationship and your perceptions of your partners use of HIV and other health services. We will NEVER disclose your HIV status to your partner. We will NEVER disclose to your partner that you told us he had tested HIV+. However, you may feel uncomfortable answering some questions asked during the interview or you may feel comfortable having your partner in the study. You are able to withdraw from the study at any time. During an interview you can say "I don't want to answer" to any questions that make you uncomfortable. All questions will be asked in a private place so that no one else will hear your answers.

If you experience distress or adverse events as a result of the study, we will provide you with counseling resources or refer you to resources for assistance.

- **Are there any potential benefits to participating?**

You will have the opportunity to discuss information about your well-being, your relationship with your partner, and HIV services for men with a Research Assistant in a confidential, private manner.

- **Are there any potential benefits to society?**

Information obtained as part of this work may be of benefit to the larger Malawi program, or similar programs in sub-Saharan Africa, since the work aims to determine if there are better ways to offer HIV services to men who use HIV self-test kits. If researchers better understand what

For peer review only

type of programs work better for men, the program in Malawi can be scaled up and strengthened to provide these specific types of increased support.

- **Will you receive payment for being part of this study?**

Your participation is entirely voluntary. You will be provided MK 7,500 (equivalent to 10USD) for each survey completed (MK 15,000 / 20USD across the duration of the study). You will receive the above compensation regardless if you use HIV services or not. Those who complete the additional in-depth interview 6-months after study enrollment will receive an additional MK 7,500 (equivalent to 10USD) for their time.

- **What is the cost of participating in this study?**

There is no cost to participate in this study.

- **Will information about me be kept confidential?**

The study team are the only people who will know about you or any information that you provide in this study. If necessary to protect your rights or welfare (for example, if you are injured and need emergency care) or if required by Malawian law, specific information about you may be made available to providers or officials.

Authorized representatives of the Malawi National Health Sciences Research Council who are responsible for ensuring the rules related to research are followed, may need to review records of study participants. As a result, they may see your name; but they will not to reveal your identity to others.

When the results of the research are published or discussed in meetings, no information will be included that would reveal your identity. Any paperwork related to the study which contains information about you will be kept in a locked cabinet in a locked office. Only staff members of the study will have access to this information. A code will be assigned to each individual participating in the study. This code will be stored on a computer in a locked file. The key to unlock the information will only be known by the research staff. All data entered into a computer will be entered using this code so information will no longer have any information that can identify you such as your name. Forms containing any identifying information will be destroyed two years after the study is finished.

- **Participation and Withdrawal**

Your participation in this research is VOLUNTARY. If you choose not to participate, that will not affect your relationship with the hospital, your health provider or health centre you usually get your medical care from, or your right to health care. If you decide to participate, you are free to withdraw your consent and stop your participation at any time and can still receive future health care at the hospital or health center you go to.

- **Withdrawal of Participation by the Investigator**

The research investigator may stop your participation in this research if he or she feels this is best for you. The investigators will make the decision and let you know if it is not possible for you to continue. The decision may be made to protect your health and safety.

For peer review only

- **Who can answer questions I might have about this study?**

In the event of a research related injury or if you experience a problem, please immediately return to the hospital or health centre you go to or contact Khumbo Phiri. The NHSRC Ministry of Health information (Dr. Mitambo) is also provided in case you have questions about your rights as a research participant.

Kusiyitsidwa kutenga nawo mbali mu kafukufuku ndi wakafukufuku

Anthu opangitsa kafukufukuyu akhonza kukuletsani kutenga nawo mbali mukafukufukuyu akaona kuti ndi bwino kuti mutero. Anthu akafukufukuwa azapanga chiganizochi ndikukudziwitsani kuti sizitheka kuti mupitirize. Chiganizochi chitha kupangidwa kuti ateteze thanzi ndi chitetezo chanu.

Investigator

Khumbo Phiri
Mobile: +265999840946
Partners in Hope Clinic
Area 36, Plot8
M1 Road South
Lilongwe, Malawi

OR

Dr.C. Mitambo
The Secretariate, NHSRC
Ministry of Health
P.O Box 30377
Lilongwe 3
Cell +265888344 443

SIGNATURE OF RESEARCH SUBJECT [OR LEGAL REPRESENTATIVE]

I have read (or someone has read to me) the information provided above. I have been given an opportunity to ask questions and all of my questions have been answered to my satisfaction. I have been given a copy of this form.

Ndawerenga (kapena munthu wina wandiwerengera) zonse zalembedwa mwambamu. Ndapatsidwa mwayi wofunsa mafunso ndi mafunso onse ndinafunsa ayankhidwa ndipo ndakhutusidwa. Ndapasidwa pepala ina yangati yomweyi.

BY SIGNING THIS FORM, I WILLINGLY AGREE TO PARTICIPATE IN THE RESEARCH:

____

Name of Subject

____

Name of Legal Representative (if applicable)

Fingerprint

For peer review only

_____________________________________ DATE (DAY/MO/YR): _________________

Signature of Subject or Legal Representative
(may place an X OR fingerprint if unable to sign)

SIGNATURE OF INVESTIGATOR OR DESIGNEE

I have explained the research to the subject or his/her legal representative and answered all of his/her questions. I believe that he/she understands the information described in this document and freely consents to participate.

____

Name of Investigator or Designee

______________________________ ___________________________

Signature of Investigator or Designee Date (must be the same as subject's)

For peer review only

WRITTEN INFORMED CONSENT
Identifying efficient linkage strategies for HIV self-testing (IDEaL)
Female (Chichewa)

Mukufunsidwa kuti mutenge nawo mbali mu gawo lino la kafukufuku wotchedwa"kupeza njira zoyenera zobwezeletsa anthu pa thandizo la ma ARV" amene akupangidwa ndi bungwe la Partners In Hope-EQUIP polojekiti ndi sukulu ya ukachenjede ya University of California Los Angeles yaku Amerika. Mukufunsidwa kuti mutenge nawo mbali mu gawo lakafukufukuyu chifukwa bwenzi lanu lalimuna linapezeka ndi kachirombo ka HIV pogwiritsa ntchito kachida koziyezela wekha HIV. Kutenga nawo mbali mukafukufuku ameneyi sikokakamiza. Tikuwerengerani uthenga omwe walembedwa pansipa, ndipo muli ololedwa kufunsa mafunso aliwonse pa zomwe simukumvetsetsa,musanapange chiganizo chotenga nawo mbali kapena ayi. ***Ine ngati othandizira mukafufuku ameneyu ndikuthandizani pa ndondomeko yotenga chilolezo***.

- **Chifukwa chiyani kafukufukuyu akuchitika?**

Kuziyeza wekha HIV ndi kofunika kwa anthu omwe akufuna kudziwa za momwe mthupi mwawo mulili ku mbali ya HIV koma sapita kuchipatala, kotelo, ndikovuta kwa anthu omwe apezeka ndi HIV kudzela chipangizo choziyezela wekha kuti apeze thandizo la zaumoyo. Akafukufuku akufuna apeze njira zabwino zomwe zingathe kuthandiza azibambo omwe apezeka ndi HIV kuti agwiritse ntchito thandizo lina lazaumoyo ngati angakonde kutero. Kafukufukuyu apeleka mwayi wa njira zosiyana siyana zothandizira HIV kuti aone zomwe zingagwire ntchito bwino kwa azibambo omwe amagwiritsa ntchito ka chida koziyezela wekha HIV.

- **Chichitike ndi chani mukatenga nawo mbali mu kafukufukuyu?**

Pali ma gawo angapo omwe adzachitike mukafukuku, Ngati mungazipereke kutenga nawo mbali mu kafukufukuyu mudzatenga nawo mbali mu magawo otsatirawa:

1. Mundilole kuti ndifufuze bwenzi lanu lalimuna kapena ndipelekezeni kwa bwenzi lanu lalimuna ndi cholinga choti nalonso litenge nawo gawo mu kafukufuku. Chidziwitso: muli omasuka kutenga nawo mbali mu kafukufu ngati bwenzi lanu lakana kutenga nawo mbali kapena mukuganiza kuti bwenzi lanu likana.

2. Pamapepo pa kucheza koyamba komanso kachiwiri othandiza kafukufuku ngati ine ndidzacheza nanu ndikukufunsani mafunso okhuza inuyo, kuphatikizapo ngati muli pa banja, muli ndi abwenzi ogonana nawo angati, maphunziro anu komanso zomwe munakumana nazo polandira thandizo la HIV komanso momwe mumnvera kumbali ya thandizo la HIV angakhalenso kuyezedwa HIV. Tikufunsani mafunso lelo (Ngati lelo muli okonzeka kuyankha mafuns)komanso miyezi inayi ikudzayi kuti tiwone ngati pali chomwe chasintha, kucheza kuli konse kuzitenga nthawi yosachepela makumi anayi ndi isanu. Muli ololedwa kukana kutenga nawo mbali mu kucheza kotsatira nthawi iliyonse.

3. Ngati bwenzi lanu lidzatenge nawo mbali mu study, adzaikidwa mu gulu limodzi mwa magulu atatu mwa mayere. Tidzachita mayere limodzi ndi bwenzi lanu kuti awone kuti ali 'gulu liti mwa magulu atatuwa. Maguluwa ali motere:

1) Chikumbutso cha ubwino wa thandizo la zaumoyo
2) Kucheza kwa chilimbitso komwe mungathe ndi mwayi ocheza ndi anthu ena ndi kuwafotokozela za umoyo wanu, zofuta zomwe mumakumana nazo komanso njira zomwe mumagwiritsa ntchito kuti moyo wanu ukhale wosavuta.
3) Thandizo la zaumoyo lomwe mumatha kulandira pakhomo monga thandizo la HIV komanso NCD lomwe mumalandila kamodzi.Pakatha masaba anayi mudzayendeledwa ndi wa zaumoyo yemwe adzakupelekezeni ku chipatala komwe mukapitilize kulandira thandizo ngati mwakonda kutelo.

Posatengera njira yomwe mwapatsidwa mongathe kukana kulandira thandizo la zaumoyo koma ndikupitiliza kutenga nawo mbali mu kafukufuku ndipo tingathe kupitilizabe kucheza komwe tatchula m'mwambamu.

4. Pamapeto tidzakuyendelani pakutha kwa miyezi isanu ndi umodzi ya kafukufuku kuti tidzacheze nanu komanso kuti tidzanve za momwe mukunvera za kafukufuku, maganizo anu okhudza njira zina za mtsogolo komanso, thandizo lina lowonjezera ngati ilipo. Si onse otenga nawo mbali omwe adzaonedwe ndipo muli ololedwa kukana kutenga nawo mbali mukucheza ndipo kukana kwanu sikudzaononga mwayi wanu otenga nawo mbali mu study.

- **Mutenga nthawi yaitali bwanji muli mukafukufuku?**

Zochitika zonse zakafukufuku zizamalizidwa mu miyezi isanu ndi imodzi.

- **Pali zinthu zosowetsa mtendere kapena zodetsa nkhawa zomwe mungayembekezere kuchokera mu kafukufukuyu?**

Mufunsidwa mafunso angapo ndi opangitsa kafukufuku okhudza maubwenzi anu ogonana komanso maganizo anu pa momwe abwenzi anu amagwiritsira thandizo la HIV ndi mathandizo ena a zaumoyo. SITIDZAULULA momwe mthupi mwanu muliri kumbali ya kachilombo ka HIV kwa bwenzi lanu. SITIDZAULURA kwa bwenzi lanu kuti munatiuza kuti ali ndi kachilombo ka HIV. Ngakhale zili choncho, mukhoza kusamasuka kuyankha mafunso ena mu kafukufuku kapenanso mukhoza kufuna kuti bwenzi lanu likhale nanu poyankha mafunsowa. Muli ndi ufulu osiya kutenga nawo gawo mu kafukufuku nthawi iliyonse. Mkati mwa kucheza kwathu, mukhoza kunena kuti "Sindikufuna kuyankha" ku funso lililonse lomwe sindinu omasuka kuyankha. Mafunso onse afunsidwa malo oduka mphepo kuti munthu wina aliyense asamve mayankho anu.

Ngati mungapeze mavuto kapena nkhawa mu mtima kamba kotenga nawo gawo mu kafukufukuyu, tikupatsani uphungu oyenera kapena kukulozerani koyenera kupeza thandizo.

- **Pali cholowa chilichonse potenga nawo mbali mukafukufukuyu?**

Mudzakhala ndi mwayi okambilana ndikunva zambiri zokhudza moyo wanu komanso thandizo la HIV ndi othandiza kafukufuku komanso dotolo munjira yachinsisi. Mudzakhalanso ndi mwayi oyamba mankhwala a ma ARV ku chipatala cha kufuna kwanu.

- **Pali cholowa chilichonse kwa anthu a mudera?**

Uthenga womwe udzatengedwe ngati mbali imodzi yakafukufukuyu uzakhala othandiza mu mapologalamu a dziko la Malawi, kapena ma pologalamu ena ofananirapo a kum'mwera kwa Africa, chifukwa choti ntchito imeneyi ikufuna kuona ngati pali njira yabwino yopeleka thandizo la HIV mwa

For peer review only

azibambo, ma pologalamu aku Malawi azapita patsogolo ndikulimbikitsa njira zopititsira patsogolo.

- **Kodi mulandira malipiro potenga nawo mbali mukafukufukuyu?**

Kutenga nawo mbali kosakakamiza. Mudzalandira chiongola dzanja cha ndalama zokwana 7,500(Pafupifupi MK 15,000/20USD). Mudzalandila ndalamazi olo mutakhala kuti simunatenge nawo kapena mwatenga nawo mbali mu thandizo la HIV. Kwa omwe adzamalize nawo kucheza kowonjezera patatha miyezi isanu ndi umodzi adzalandira ndalama yowonjezera yokwana MK 7,500 (yokwana pafupi fupi 10 USD0 chifukwa cha nthawi yawo.

- **Kodi pali kulipira kulikonse chifukwa chotenga nawo mbali mukafukufukuyu?**

Kutenga nawo mbali mukafukufukuyu ndi kwaulere.

- **Kodi uthenga wanga uzasungidwa mwa chinsinsi?**

Anthu ogwira nawo ntchito mu kafukufuku okhawo ndi amene adziwe za uthenga wanu kapena chilichonse chomwe mutiuze pa kafukufukuyu.

Anthu ovomerezeka oyimirira bungwe la National Health Sciences Research Council ndi UCLA office for Protection Of Research Subjects ndi amene ali ndi udindo woonetsetsa kuti malamulo a kafukufuku akutsatidwa, akhoza kufuna kuona nawo kaundula wa anthu amene akutenga nawo mbali mukafukufukuyu. Kutanthauza kuti akhonza kuzaona dzina lanu; koma sangaulure zokhuza inu kwa anthu ena.

Pa nthawi imene zotsatira za kafukufukuyu zidzatsindikidwa kapena kukambidwa mu misonkhano, palibe uthenga womwe udzayikidwe wokuzindikiritsani. Uthenga uliwonse wolembedwa pa pepala wokhunza inu uzasungidwa mu kabati yokhoma mu ofesi yokhomanso. Anthu ogwira nawo ntchito mukafukufukuyu okhawo ndi amene azathe kuona uthenga umenewu. Njira yotanthauzira uthenga umenewu izadziwika ndi anthu akafukufukuyu basi. Uthenga onse olowetsedwa pa makina a kompyuta uzalowetsedwa kugwiritsa ntchito nambala, ndekuti uthenga onse sudzakhalanso ndi zokuzindikiritsani monga dzina lanu. Ma pepala amene pali uthenga okuzindikitsani adzawonongedwa pakapita zaka ziwiri chimalizireni kafukufukuyu.

- **Kutenga mbali ndi kusiya kutenga mbali**

Kutenga nawo mbali mu kafukufukuyu SIKOKAKAMIZA. Ngati musankhe kuti simutenga nawo mbali, izi sizingasokoneze ubale wanu ndi chipatala chino, anthu ogwira ntchito kuchipatala,kapena chipatala chomwe mumalandilirako chithandizo, kapena ufulu wanu wolandira thandizo la chipatala. Ngati mwapanga chisankho chotenga nawo mbali, mukhoza kuchosa chilolezo chanu ndikusiya kutenga nawo mbali nthawi ina iliyonse ndipo mungathe kuzalandirabe chithandizo pa chipatala pano mtsogolo.

- **Kusiyitsidwa kutenga nawo mbali mu kafukufuku ndi wakafukufuku**

Anthu opangitsa kafukufukuyu akhonza kukuletsani kutenga nawo mbali mukafukufukuyu akaona kuti ndi bwino kuti mutero. Anthu akafukufukuwa azapanga chiganizochi ndikukudziwitsani kuti sizitheka kuti mupitirize. Chiganizochi chitha kupangidwa kuti ateteze thanzi ndi chitetezo chanu.

For peer review only

- **Angayankhe ndi ndani mafunso amene ndingakhale nawo wokhuza kafukufukuyu?**

Zitachitika kuti mwavulala kamba ka kafukufukuyu kapena mukukumana ndi vuto,chonde bwelerani mwachangu kuchipatala chomwe munapitako kapena lumikizanani ndi Mike Nyirenda. Bungwe la NHSRC Ministry of Health information kudzera mwa Dr Kathyola liliponso ngati mungakhale ndi mafunso okhudza ufulu wanu ngati munthu wotenga nawo mbali mu kafukufuku.

Investigator
Khumbo Phiri
Mobile: +265999840946
Partners in Hope Clinic
Area 36, Plot8
M1 Road South
Lilongwe, Malawi

OR

Dr.C. Mitambo
The Secretariate, NHSRC
Ministry of Health
P.O Box 30377
Lilongwe 3
Cell +265888344 443

SAYINI YA OTENGA NAWO MBALI [KAPENA YA OYIMIRA OTENGA NAWO MBALI]

Ndawerenga (kapena munthu wina wandiwerengera) zonse zalembedwa mwambamu. Ndapatsidwa mwayi wofunsa mafunso ndi mafunso onse ndinafunsa ayankhidwa ndipo ndakhutusidwa. Ndapasidwa pepala ina yangati yomweyi.

POSAYINA PA PEPALALI, NDIKUVOMERA MOSAKAKAMIZIDWA KUTENGA NAWO MBARI MU KAFUKUFUKU:

____

Name of Subject

____

Name of Legal Representative (if applicable)

Fingerprint

______________________________ DATE (DAY/MO/YR): ________________

Signature of Subject or Legal Representative

(may place an X OR fingerprint if unable to sign)

For peer review only

SAYINI YA OFUNSA MAFUNSO

Ndamufotokozera otenga nawo mbali/oyimirira otenga nawo mbali za kafukufukuyu ndipo ndayankha mafunso onse amene anali nawo. Ndikukhulupirira kuti amvetsetsa uthenga onse omwe uli chikalata cha uthengawu ndipo avomera kutenga nawo mbali mwa kufuna kwao.

____

Dzina la ofunsa mafunso kapena oyimira akafukufuku

________________________________ ____________________________

Sayini ya ofusa mafunso kapena oyimira akafukufuku Date (must be the same as subject's)

For peer review only

14.4.APPENDIX D: Written Informed Consent – Male

WRITTEN INFORMED CONSENT
Identifying efficient linkage strategies for HIV self-testing (IDEaL)
Male

You are asked to participate in a research study entitled *"Identifying efficient linkages strategies for HIVST"* conducted by Partners in Hope and the University of California Los Angeles in the United States. You are being requested to take part in the study because you recently received a self-test kit and reported recently testing HIV-positive. Your participation in this study is entirely voluntary. You will be read the information below, and you are free to ask questions about anything you do not understand, before deciding whether or not to participate. ***I as the field assistant for this study will take you through this consenting process.***

- **Why is this study being done?**

HIV self-testing is very helpful for people who want to know their status but do not usually go to the health facility. However, it can be hard for individuals who test HIV-positive with HIV self-testing to be able to access other health services. Researchers want to determine what are the best interventions that can help men who are diagnosed with HIV use other health services, if desired. The study will offer several different strategies for HIV services to see what works best for men who use HIV self-testing kits.

- **What will happen if you take part in this research study?**

There are several steps to this study. If you volunteer to participate in this study you will have the opportunity to participate in the following components:

1. Complete two study visits where a research assistant like myself will interview you and ask you information about yourself, including whether you are married, number of sexual partners, your level of education, information about your experiences with HIV services, and how you feel about HIV testing and treatment services. We will ask you questions today (or a day that is convenient for you) and in four months in order to see if anything has changed. Each interview will last about 45minutes. You can refuse a follow-up survey at any point.
2. After completing the first interview you will be randomized to one of three interventions. We will do the randomization together so you can see exactly what you will be offered. Based on what intervention you are randomly selected for, you will also be offered a variety of health services from health facility staff. The potential interventions are:

    a) Reminders about the benefits of health services;

    b) Motivational Interviewing where you can talk to someone about your life, challenges you face, and strategies to make your life and better, and additional services as needed

    c) Home-based health services whereby a provider will offer you HIV services and NCD screening at your home as a one-time event. After four weeks, you will be visited by a health care worker who will escort you to the clinic for continued health services if desired.

Regardless of what arm you are randomized to, you can always refuse health services or refuse talking to a health care provider and still remain in the study. Even if you do not plan to use any additional

health services, don't worry, you can still be in the study and we can still complete the interviews we discussed above.

3. In the first 6 months of the study, we will also review medical records at your local health facility to see if you visited the health facility since enrolling in the study. This does not require interaction with you and will be completely confidential.

4. Finally, we may contact you within 6-months of the study to complete another in-depth interview so we can learn more about your experience with the study, recommendations for future interventions, and what additional services, if any, you would like. Not all participants will be contacted for the interview and you always have the right to decline an in-depth interview – refusal will not affect your participation in the larger study.

- **How long will you be in the research study?**

All study activities will be completed within 6-months of today.

- **Are there any potential risks or discomforts that you can expect from this study?**

You will be asked a series of questions by a research assistant about your sexual history and your experience receiving and using a self-test kit from your sexual partner. You may feel uncomfortable answering some of the questions asked by the interviewer. You can say "I don't want to answer" to any questions that make you uncomfortable. All questions will be asked in a private place so that no other patients or staff will hear your answers.

If you agree to participate in the assigned intervention, providers may ask to reach you at home or in the community. As with any health service, if you choose to initiate ART you may be at risk of unwanted status disclosure.

If you experience distress or adverse events, we will provide you with counseling resources or refer you to resources for assistance.

- **Are there any potential benefits to participating?**

You will have the opportunity to discuss information about your well-being and HIV services with a Research Assistant and possibly a health care provider and in a confidential, private manner. You will also have the chance to link to HIV care services at the facility of your choosing.

- **Are there any potential benefits to society?**

Information obtained as part of this work may be of benefit to the larger Malawi program, or similar programs in sub-Saharan Africa, since the work aims to determine if there are better ways to offer HIV services to men who use HIV self-test kits. If researchers better understand what type of programs work better for men, the program in Malawi can be scaled up and strengthened to provide these specific types of increased support.

- **Will you receive payment for being part of this study?**

Your participation is entirely voluntary. You will be provided MK 7,500 (equivalent to 10USD) for each survey completed (MK 15,000 / 20USD across the duration of the study). You will receive the above compensation regardless if you use HIV services or not. Those who complete the additional in-depth interview 6-months after study enrollment will receive an additional MK 7,500 (equivalent to 10USD) for their time.

- **What is the cost of participating in this study?**

There is no cost to participate in this study.

For peer review only

- **Will information about me be kept confidential?**

Authorized representatives of the Malawi National Health Sciences Research Council who are responsible for ensuring the rules related to research are followed, may need to review records of study participants. As a result, they may see your name; but they will not to reveal your identity to others.

When the results of the research are published or discussed in meetings, no information will be included that would reveal your identity. Any paperwork related to the study which contains information about you will be kept in a locked cabinet in a locked office. Only staff members of the study will have access to this information. A code will be assigned to each individual participating in the study. This code will be stored on a computer in a locked file. The key to unlock the information will only be known by the research staff. All data entered into a computer will be entered using this code so information will no longer have any information that can identify you such as your name. Forms containing any identifying information will be destroyed two years after the study is finished.

- **Participation and Withdrawal**

Your participation in this research is VOLUNTARY. If you choose not to participate, that will not affect your relationship with the hospital, your health provider or health centre you usually get your medical care from, or your right to health care. If you decide to participate, you are free to withdraw your consent and stop your participation at any time and can still receive future health care at the hospital or health center you go to.

- **Withdrawal of Participation by the Investigator**

The research investigator may stop your participation in this research if he or she feels this is best for you. The investigators will make the decision and let you know if it is not possible for you to continue. The decision may be made to protect your health and safety.

- **Who can answer questions I might have about this study?**

In the event of a research related injury or if you experience a problem, please immediately return to the hospital or health centre you go to or contact Mike Nyirenda. The NHSRC Ministry of Health information (Dr. Kathyola) is also provided in case you have questions about your rights as a research participant.

Investigator:

Khumbo Phiri
Mobile: +265999840946
Partners in Hope Clinic
Area 36, Plot8
M1 Road South
Lilongwe, Malawi

OR

Dr.C. Mitambo
The Secretariate, NHSRC
Ministry of Health
P.O Box 30377
Lilongwe 3
Cell +265888344 443

For peer review only

SIGNATURE OF RESEARCH SUBJECT [OR LEGAL REPRESENTATIVE]

I have read (or someone has read to me) the information provided above. I have been given an opportunity to ask questions and all of my questions have been answered to my satisfaction. I have been given a copy of this form.

BY SIGNING THIS FORM, I WILLINGLY AGREE TO PARTICIPATE IN THE RESEARCH:

____

Name of Subject

____

Name of Legal Representative (if applicable)

______________________________________ DATE (DAY/MO/YR): __________________

Signature of Subject or Legal Representative
(may place an X OR fingerprint if unable to sign)

SIGNATURE OF INVESTIGATOR OR DESIGNEE

I have explained the research to the subject or his/her legal representative and answered all of his/her questions. I believe that he/she understands the information described in this document and freely consents to participate.

____

Name of Investigator or Designee

_______________________________ ____________________________

Signature of Investigator or Designee Date (must be the same as subject's)

For peer review only

WRITTEN INFORMED CONSENT
Identifying efficient linkage strategies for HIV self-testing (IDEaL)
Male (Chichewa)

Mukufunsidwa kuti mutenge nawo mbali mu gawo lino la kafukufuku wotchedwa"kupeza njira zoyenera zobwezeletsa anthu pa thandizo la ma ARV" amene akupangidwa ndi bungwe la Partners In Hope-EQUIP polojekiti ndi sukulu ya ukachenjede ya University of California Los Angeles yaku Amerika. Mukufunsidwa kuti mutenge nawo mbali mu gawo lakafukufukuyu chifukwa muli ndi kachirombo ka HIV komanso mwalandila kachipangizo koziyezela wekha HIVi. Kutenga nawo mbali mukafukufuku ameneyi sikokakamiza. Tikuwerengerani uthenga omwe walembedwa pansipa, ndipo muli ololedwa kufunsa mafunso aliwonse pa zomwe simukumvetsetsa,musanapange chiganizo chotenga nawo mbali kapena ayi. Ine ngati othandizira mukafufuku ameneyu ndikuthandizani pa ndondomeko yotenga chilolezo.*

Chifukwa chiyani kafukufukuyu akuchitika?

Kuziyeza wekha HIV ndi kofunika kwa anthu omwe akufuna kudziwa za momwe mthupi mwawo mulili ku mbali ya HIV koma sapita kuchipatala, kotelo, ndikovuta kwa anthu omwe apezeka ndi HIV kudzela chipangizo choziyezela wekha kuti apeze thandizo la zaumoyo. Akafukufuku akufuna apeze njira zabwino zomwe zingathe kuthandiza azibambo omwe apezeka ndi HIV kuti agwiritse ntchito thandizo lina lazaumoyo ngati angakonde kutero. Kafukufukuyu apeleka mwayi wa njira zosiyana siyana zothandizira HIV kuti aone zomwe zingagwire ntchito bwino kwa azibambo omwe amagwiritsa ntchito ka chida koziyezela wekha HIV.

Chichitike ndi chani mukatenga nawo mbali mu kafukufukuyu?

Pali ma gawo angapo omwe adzachitike mukafukuku, Ngati mungazipereke kutenga nawo mbali mu kafukufukuyu mudzatenga nawo mbali mu magawo otsatirawa:

Akafukufuku adzakuyendera kawiri. Ulendo woyamba tidzapemphani kuti mutenge nawo mbali mu kafukufuku yemwe tidzakufunseni zokhuza inuyo, kuphatikizapo ngati muli pa banja, muli ndi abwenzi ogonana nawo angati, maphunziro anu komanso zomwe munakumana nazo polandira thandizo la HIV komanso momwe mumnvera kumbali ya thandizo la HIV angakhalenso kuyezedwa HIV. Tikufunsani mafunso lelo (Ngati lelo muli okonzeka kuyankha mafuns)komanso miyezi inayi ikudzayi kuti tiwone ngati pali chomwe chasintha, kucheza kuli konse kuzitenga nthawi yosachepela makumi anayi ndi isanu. Muli ololedwa kukana kutenga nawo mbali mu kucheza kotsatira nthawi iliyonse.

2. Pamapepo pa kucheza koyamba mudzayikidwa mu imodzi mwa njira zitatu popanda ndondomeko iliyonse,pa nthawi yomwe mudzakhale mukuyikidwa mu njirayi mudzakhala muli pomwepo kuti muone njira yomwe mwapatsidwa.Potengera njira yomwe mwayikidwa mudzapatsidwanso mwayi wa mathandizo a zaumoyo angapo ochoka kwa opeleka thandizo la zaumoyo pa chiptala, zina mwa njira ndi:

a) Chikumbutso cha ubwino wa thandizo la zaumoyo

b) Kucheza kwa chilimbitso komwe mungathe ndi mwayi ocheza ndi anthu ena ndi kuwafotokozela za umoyo wanu, zofuta zomwe mumakumana nazo komanso njira zomwe mumagwiritsa ntchito kuti moyo wanu ukhale wosavuta.

c) Thandizo la zaumoyo lomwe mumatha kulandira pakhomo monga thandizo la HIV komanso NCD lomwe mumalandila kamodzi.Pakatha masaba anayi mudzayendeledwa ndi wa zaumoyo yemwe adzakupelekezeni ku chipatala komwe mukapitilize kulandira thandizo ngati mwakonda kutelo.

Posatengera njira yomwe mwapatsidwa mongathe kukana kulandira thandizo la zaumoyo koma ndikupitiliza kutenga nawo mbali mu kafukufuku ndipo tingathe kupitilizabe kucheza komwe tatchula m'mwambamu.

3. Miyezi isanu ndi umodzi yoyambilira ya kafukufuku tidzaona zambiri ya umoyo wanu ku chipatala cha m'dela lanu ngati munapitako mutalowa kale mu kafukufuku, zimenezi sizidzafuika kulankhula nanu ndipo zidzachitika mwachinsinsi.

4.Pamapeto tidzakuyendelani pakutha kwa miyezi isanu ndi umodzi ya kafukufuku kuti tidzacheze nanu komanso kuti tidzanve za momwe mukunvera za kafukufuku, maganizo anu okhudza njira zina za mtsogolo komanso, thandizo lina lowonjezera ngati ilipo. Si onse otenga nawo mbali omwe adzaonedwe ndipo muli ololedwa kukana kutenga nawo mbali mukucheza ndipo kukana kwanu sikudzaononga mwayi wanu otenga nawo mbali mu study.

Mutenga nthawi yaitali bwanji muli mukafukufuku?

Zochitika zonse zakafukufuku zizamalizidwa pa miyezi isanu ndi umodzi (6) kuchokera lero.

Pali zinthu zosowetsa mtendere kapena zodetsa nkhawa zomwe mungayembekezere kuchokera mu kafukufukuyu?

Muzafunsidwa mndandanda wa mafunso ndi othandizira mukafukufuku ameneyi zokhuza mbiri yanu pankhani zogonana ndi zochitika mutalandira ka chida koziyeza wekha kuchokera kwa bwezi wanu ogonana naye komanso mmene munagwiritsira ntchito. Mutha kukhala osamasuka poyankha mafunso ena omwe wofunsa mafunso angafunse. Mutha kunena kuti "sindikufuna kuyankha" kufunso lilironse lomwe simukumasuka nalo. Mafunso onse azafunsidwa pa malo achinsinsi pomwe odwala anzanu kapena ogwira ntchito pachipatala sadzamva nawo mayankho anu.

Ngati mudzavomere kutenga nawo mbali mu imodzi mwa njira, a zaumoyo adzakufunsani kuti akupezeni kunyumba kwanu kapena mu dela lanu. Monga mwa thandizo lililonse la zaumoyo, ngati mungavomere kuyamba kumwa mankhwala a ma ARV mungathe kukhala pa chiospyezo choulula za momwe mulili m'mthupi mwano mosafuna.

Ngati mungakumane ndi masautso aliwonse, tidzakupatsani uphungu kapena kukutumizani koti mukathandizidwe ndi uphungu.

Pali cholowa chilichonse potenga nawo mbali mukafukufukuyu?

Mudzakhala ndi mwayi okambilana ndikunva zambiri zokhudza moyo wanu komanso thandizo la HIV ndi othandiza kafukufuku komanso dotolo munjira yachinsisi. Mudzakhalanso ndi mwayi oyamba mankhwala a ma ARV ku chipatala cha kufuna kwanu.

Pali cholowa chilichonse kwa anthu a mudera?

Uthenga womwe udzatengedwe ngati mbali imodzi yakafukufukuyu uzakhala othandiza mu mapologalamu a dziko la Malawi, kapena ma pologalamu ena ofananirapo a kum'mwera kwa Africa, chifukwa choti ntchito imeneyi ikufuna kuona ngati pali njira yabwino yopeleka thandizo la HIV mwa azibambo, ma pologalamu aku Malawi azapita patsogolo ndikulimbikitsa njira zopititsira patsogolo.

Kodi mulandira malipiro potenga nawo mbali mukafukufukuyu?

Kutenga nawo mbali kosakakamiza. Mudzalandira chiongola dzanja cha ndalama zokwana 7,500(Pafupifupi MK 15,000/20USD). Mudzalandila ndalamazi olo mutakhala kuti simunatenge nawo kapena mwatenga nawo mbali mu thandizo la HIV. Kwa omwe adzamalize nawo kucheza kowonjezera patatha miyezi isanu ndi umodzi adzalandira ndalama yowonjezera yokwana MK 7,500 (yokwana pafupi fupi 10 USD0 chifukwa cha nthawi yawo.

Kodi pali kulipira kulikonse chifukwa chotenga nawo mbali mukafukufukuyu?

Kutenga nawo mbali mukafukufukuyu ndi kwaulere.

Kodi uthenga wanga uzasungidwa mwa chinsinsi?

Anthu ogwira nawo ntchito mu kafukufuku okhawo ndi amene adziwe za uthenga wanu kapena chilichonse chomwe mutiuze pa kafukufukuyu.

Anthu ovomerezeka oyimirira bungwe la National Health Sciences Research Council ndi UCLA office for Protection Of Research Subjects ndi amene ali ndi udindo woonetsetsa kuti malamulo a kafukufuku akutsatidwa, akhoza kufuna kuona nawo kaundula wa anthu amene akutenga nawo mbali mukafukufukuyu. Kutanthauza kuti akhonza kuzaona dzina lanu; koma sangaulure zokhuza inu kwa anthu ena.

Pa nthawi imene zotsatira za kafukufukuyu zidzatsindikidwa kapena kukambidwa mu misonkhano, palibe uthenga womwe udzayikidwe wokuzindikiritsani. Uthenga uliwonse wolembedwa pa pepala wokhunza inu uzasungidwa mu kabati yokhoma mu ofesi yokhomanso. Anthu ogwira nawo ntchito mukafukufukuyu okhawo ndi amene azathe kuona uthenga umenewu. Njira yotanthauzira uthenga umenewu izadziwika ndi anthu akafukufukuyu basi. Uthenga onse olowetsedwa pa makina a kompyuta uzalowetsedwa kugwiritsa ntchito nambala, ndekuti uthenga onse sudzakhalanso ndi zokuzindikiritsani monga dzina lanu. Ma pepala amene pali uthenga okuzindikitsani adzawonongedwa pakapita zaka ziwiri chimalizireni kafukufukuyu.

Kutenga nawo mbali mu kafukufukuyu SIKOKAKAMIZA. Ngati musankhe kuti simutenga nawo mbali, izi sizingasokoneze ubale wanu ndi chipatala chino, anthu ogwira ntchito kuchipatala,kapena chipatala chomwe mumalandilirako chithandizo, kapena ufulu wanu wolandira thandizo la chipatala. Ngati mwapanga chisankho chotenga nawo mbali, mukhoza kuchosa chilolezo chanu ndikusiya kutenga nawo mbali nthawi ina iliyonse ndipo mungathe kuzalandirabe chithandizo pa chipatala pano mtsogolo.

Kusiyitsidwa kutenga nawo mbali mu kafukufuku ndi wakafukufuku

Anthu opangitsa kafukufukuyu akhonza kukuletsani kutenga nawo mbali mukafukufukuyu akaona kuti ndi bwino kuti mutero. Anthu akafukufukuwa azapanga chiganizochi ndikukudziwitsani kuti sizitheka kuti mupitirize. Chiganizochi chitha kupangidwa kuti ateteze thanzi ndi chitetezo chanu.

Kusiyitsidwa kutenga nawo mbali mu kafukufuku ndi wakafukufuku

Anthu opangitsa kafukufukuyu akhonza kukuletsani kutenga nawo mbali mukafukufukuyu akaona kuti ndi bwino kuti mutero. Anthu akafukufukuwa azapanga chiganizochi ndikukudziwitsani kuti sizitheka kuti mupitirize. Chiganizochi chitha kupangidwa kuti ateteze thanzi ndi chitetezo chanu.

Investigator:

Khumbo Phiri
Mobile: +265999840946
Partners in Hope Clinic
Area 36, Plot8
M1 Road South
Lilongwe, Malawi

OR

Dr.C. Mitambo
The Secretariate, NHSRC
Ministry of Health
P.O Box 30377
Lilongwe 3
Cell +265888344 443

Wotenga mbali kapena omuyimira asayine

Ndawerenga (kapena munthu wina wandiwerengera) zonse zalembedwa mwambamu. Ndapatsidwa mwayi wofunsa mafunso ndi mafunso onse ndinafunsa ayankhidwa ndipo ndakhutitsidwa. Ndapatsidwa pepala lina langati lomweyi.

____

Dzina la otenga mbali

____

Dzina la oyimola malamulo

______________________________________ Tsiku: __________________

Wotenga mbali kapena oyimira malamulo asayine

(Mutha kuyika X ngati simungathe kusayinila)

Ofufuza asayinire/Kapena othandizila Kafukufuku

Ndafotokoza za kafukufuku uyu kwa otenga nawo mbali kapena owayimila, ndayankhanso mafunso awo onse. Ndikukhulupilira kuti amvetsetsa zomwe zananedwa mu chikalata ichi

For peer review only

ndiponso avomereza mwakufuna kwawo kutenga nawo mbali.

____

Dzina la ofufuza kapena othandizila kafukufuku

__________________________________ ______________________________

Ofufuza kapena othandizila kafukufuku asayine apa Tsiku

For peer review only

14.5. APPENDIX E: Baseline Survey – Male

BASELINE SURVEY
Identifying efficient linkage strategies for HIV self-testing (IDEaL)
Male

**Question Name**	**Label**	**Responses**
INTRODUCTION SECTION		
interviewer	Full Name of Interviewer	
Interview date	Interview date	
Time start	Time survey started	
District	District	
TA	TA	
village	Village	
SECTION A: DEMOGRAPHICS		
Intro Note	Thank you for agreeing to participate. Now I will ask you a few questions about yourself and who you are. Please feel free to answer honestly. There are no right or wrong answers.	
a7	What is your tribe?	1. Lomwe<br>2. Sena<br>3. Chewa<br>4. Mang'anja/Nyanja<br>5. Ngoni<br>6. Tumbuka<br>7. Tonga<br>8. Yao

For peer review only

		99. Other, specify
a3	What is the highest level of school you attended?	1. Primary<br>2. Secondary<br>3. Higher
a3b	What class did you complete in your highest level of school?	____________________
a4	Please think of the past 12months, how would you describe your primary occupation?	1. Working formally (employed full time)<br>2. Working informally (ganyu, farming, business)<br>3. Not working
a5	Are you currently married?	1. Married<br>2. Live-in partner<br>3. Steady Girlfriend/Boyfriend<br>4. Separated<br>5. Divorced<br>99. Other, specify
a6	How many living children do you have?	____________________
a6b	What is the age of your youngest child?	____________________
a6bc	What age is the child (in years or months)	____________________
a4b	How many children currently live with you?	____________________
a7	How many sexual partners have you had in the past 12 months?	____________________
a8	Have you had sex with someone besides your wife/husband without a condom in the past 12 months?	1. Yes<br>0. No<br>88. Don't know/ Not sure<br>89. Refused to answer

For peer review only

a8b	Have you had sex without a condom in the past 12 months?	1. Yes<br>0. No<br>88. Don’t know/ Not sure<br>89. Refused to answer
SECTION B: INCOME QUESTIONS		
Intro Note	I will now discuss with you about the valuable items that you or your household possesses. As I will be chatting with you I will also some questions about money you have and activities that you indulge in to find money.	
b1	Please think of the past 12 months, how would you describe your primary occupation?	1. Working formally (employed full time)<br>2. Working informally (ganyu, farming, business)<br>3. Not working
b1b	Think about all the work you have done in the past month. How many days did you normally work this month that gave you pay?	________________
b2	Do you have any savings for the future, such as a bank account, savings group or cash?	1. Yes<br>0. No
Household Assets		
b3	Does your household have:<br>*The respondent said that his/her household doesn't have any of the household assets. Please probe and ensure that this is correct before you proceed.*	
b3_1	Metal Roof?	1. Yes<br>0. No
b3_2	Electricity?	1. Yes

For peer review only

		0. No
b3_3	Paraffin lamp with no glass?	1. Yes<br>0. No
b3_4	A paraffin lamp?	1. Yes<br>0. No
b3_5	A radio?	1. Yes<br>0. No
b3_6	A television?	1. Yes<br>0. No
b3_7	A cellular phone?	1. Yes<br>0. No
b3_8	A bed?	1. Yes<br>0. No
b3_9	A sofa set?	1. Yes<br>0. No
b3_10	A table?	1. Yes<br>0. No
b3_11	A refrigerator	1. Yes<br>0. No
b3_12	Mattress?	1. Yes<br>0. No
b3_13	Chair(s)?	1. Yes<br>0. No
b3_14	Cattle?	1. Yes<br>0. No
b3_15	Goat?	1. Yes<br>0. No
b3_16	Sheep?	1. Yes<br>0. No
b3_17	Pigs?	1. Yes<br>0. No

For peer review only

b3_18	Donkey?	1. Yes<br>0. No
b3_19	Chickens?	1. Yes<br>0. No
b3_20	Other poultry?	1. Yes<br>0. No
b4	In the past 30 days, have you drank beer?	1. Yes<br>0. No
b4b	How many days in the past 30 days have you drank beer?	______________________
b4c	How much money did you spend on beer the last time you went?	MWK:
b4d	In total, approximately how much money did you spend on beer in the past 30 days?	MWK:
Relationship		
Intro Note	Now I'd like to talk to you about your current sexual relationship	
f8	How long have you been/were you in a sexual relationship with your partner?	Days______________<br>Months_____________<br>Years_______________
f9	Do you have children with your partner? How many children?	______________________
f10	How often do you currently talk to your partner?	1. Everyday<br>2. A couple times a week<br>3. Once a week<br>4. A couple times a month<br>5. Once a month<br>6. Less than once a month<br>7. Not at all (never)
f10b	In a typical month, who earns more money? You, or your partner?	1. Myself<br>2. This partner<br>3. We earn the same amount<br>88. Don't know

For peer review only

Decision Making		
Intro Note	Now I would like to talk to you about how you and your partner make decisions.	
f11	Who usually decides how the money you earn will be used?	1. Yourself (Respondent)<br>2. Jointly (This partner and you together)<br>3. Mainly this partner<br>4. Someone else<br>5. Do not earn money<br>88. Refuse to say
f11b	(if above question=4) Who decides?	________________________
f12	Who usually decides how your partner's earnings will be used?	1. Yourself (Respondent)<br>2. Jointly (This partner and you together)<br>3. Mainly this partner<br>4. Someone else<br>5. Do not earn money<br>88. Refuse to say
f12b	(if above question=4) Who decides?	________________________
f13	Who usually makes decisions about health care for yourself?	1. Yourself (Respondent)<br>2. Jointly (This partner and you together)<br>3. Mainly this partner<br>4. Someone else<br>5. Not applicable/ Don't have children<br>88. Refuse to say
f13b	(if above question=4) Who decides?	________________________
f14	Who usually makes decisions about health care for your child with this partner?	1. Yourself (Respondent)<br>2. Jointly (This partner and you together)<br>3. Mainly this partner<br>4. Someone else

For peer review only

		5. Not applicable/ Don't have children<br>88. Refuse to say
f14b	(if above question=4) Who decides?	___________________
f15	Who usually makes decisions about health care for your partner?	1. Yourself (Respondent)<br>2. Jointly (This partner and you together)<br>3. Mainly this partner<br>4. Someone else<br>5. Not applicable/ Don't have children<br>88. Refuse to say
f15b	(if above question=4) Who decides?	___________________
f16	Who usually makes decisions about making major household purchases?	1. Yourself (Respondent)<br>2. Jointly (This partner and you together)<br>3. Mainly this partner<br>4. Someone else<br>5. Not applicable/ Don't have children<br>88. Refuse to say
f16b	(if above question=4) Who decides?	
Note	I would like to ask you questions about [probability/chance/likelihood] that certain things will happen. There are ten beans in this cup. I will ask you to pick some of the beans and put them in the plate. The number of beans that you are going to put in the plate will reflect the probability that something will happen. One bean means there is very little chance that something will happen. If you do not put any bean in the plate it means you are certain that there is no likelihood that something will happen.	

For peer review only

note2	If you put additional beans in the plate it means the chance that something will happen will also increase. For example, if you put one or two beans in the plate, it means there is little chance that something will happen. Even though there is little chance but it can happen. If you put ten beans it means there is equal chance of something happening or not. If you put six beans it means the chance that something will happen is slightly greater than not happening. If you put all ten beans, it means you are certain that whatever the case something will really happen. There is no wrong or right answer I just want to know what you think.	
note3	INTERVIEWER: Report for each question the NUMBER OF BEANS put in the PLATE. After each question, replace the beans on the table (unless otherwise noted).	
Practice		
pr1	Pick the number of beans that reflects how likely you think it is that:	____________________
pr1b	You will go to the market at least once within the next 2 days.	____________________
pr1c	You will go to the market at least once within the next 2 weeks.	____________________
Practice		
pr2	INTERVIEWER: Did Respondent add any beans between pr1b and pr1c?	1. Yes<br>0. No

For peer review only

pr3	Remember, as time goes by, you may find more time to go to the market. Therefore, you should have added beans to the plate. Let me ask you again. Now, add beans in the plate so that the number of beans in the plate reflects how likely you think it is that you will go to the market at least once within 2 weeks.<br><br>How likely you think it is that you will go to the market at least once within 2 weeks?	________________
f17	Pick the number of beans that reflects how likely you think:	
f17b	You will still be married/with [partner one year from now.	________________
f17c	You are currently infected with HIV/AIDS	________________
f17d	You will become infected with HIV/AIDS during the next 12 months	________________
f17e	You will become infected with HIV/AIDS during their lifetime	________________
f17c	partner is infected with HIV/AIDS now.	________________
f17d	partner will become infected with HIV/AIDS during the next 12 months.	________________
SELF REPORTED HEALTH AND HAPPINESS		
Intro Note	Now I'd like to talk to you about how healthy and happy you feel.	
e1	I am interested in your general level of well-being or satisfaction with life. How satisfied are you with your life, all things considered?	1. Very satisfied<br>2. Somewhat satisfied<br>3. Neutral<br>4. Somewhat unsatisfied<br>5. Very unsatisfied

For peer review only

e2	Do you think that you are more, equally or less satisfied than other persons your age and sex living in your village?	1. More satisfied<br>2. Equally satisfied<br>3. Less satisfied
e3	In general, would you say your health now is: very good, good, poor or very poor?	1. Very good<br>2. Good<br>3. Poor<br>4. Very poor
e4	How would you compare your health to other people of the same age and sex in your village?	1. More healthy<br>2. Equally healthy<br>3. Less healthy
e5	In the past month, how many days were you too sick to work/go to school/complete household chores?	____________________
Happiness		
e6	How true are the following statements for you in the last month?	
e6_1	I have felt depressed	1. Strongly Agree<br>2. Agree<br>3. Disagree<br>4. Strongly Disagree
e6_2	I have felt life was not worth living	1. Strongly Agree<br>2. Agree<br>3. Disagree<br>4. Strongly Disagree
e6_3	I have felt content.	1. Strongly Agree<br>2. Agree<br>3. Disagree<br>4. Strongly Disagree
e6_4	I have felt lonely	1. Strongly Agree<br>2. Agree<br>3. Disagree<br>4. Strongly Disagree
GENDER EQUITABLE MEN SCALE		
Note	Please tell me if you strongly agree, agree, disagree, or strongly disagree with the following statements:	

For peer review only

j1	Woman's most important role is to take care of her home and cook (take care of home is about housekeeping)	1. Strongly Agree<br>2. Agree<br>3. Unsure<br>4. Disagree<br>5. Strongly Disagree<br>88. Refuse to say
j2	Men need sex more than women	1. Strongly Agree<br>2. Agree<br>3. Unsure<br>4. Disagree<br>5. Strongly Disagree<br>88. Refuse to say
j3	Men don't talk about sex, they just do it.	1. Strongly Agree<br>2. Agree<br>3. Unsure<br>4. Disagree<br>5. Strongly Disagree<br>88. Refuse to say
j4	There are times when a woman deserves to be beaten	1. Strongly Agree<br>2. Agree<br>3. Unsure<br>4. Disagree<br>5. Strongly Disagree<br>88. Refuse to say
j5	Changing diapers, giving kids a bath & feeding kids are mother's responsibility	1. Strongly Agree<br>2. Agree<br>3. Unsure<br>4. Disagree<br>5. Strongly Disagree<br>88. Refuse to say
j6	It is a woman's responsibility to avoid getting pregnant	1. Strongly Agree<br>2. Agree<br>3. Unsure<br>4. Disagree<br>5. Strongly Disagree<br>88. Refuse to say
j7	A man should have the final word about decisions in his home	1. Strongly Agree<br>2. Agree<br>3. Unsure<br>4. Disagree

For peer review only

		5. Strongly Disagree<br><br>88. Refuse to say
j8	Men are always ready to have sex	1. Strongly Agree<br>2. Agree<br>3. Unsure<br>4. Disagree<br>5. Strongly Disagree<br><br>88. Refuse to say
j9	A woman should tolerate violence in order to keep her family together	1. Strongly Agree<br>2. Agree<br>3. Unsure<br>4. Disagree<br>5. Strongly Disagree<br><br>88. Refuse to say
j10a	I would be outraged if my wife asked me to use a condom.	1. Strongly Agree<br>2. Agree<br>3. Unsure<br>4. Disagree<br>5. Strongly Disagree<br><br>88. Refuse to say
j10b	Men would be outraged if their wife asked them to use a condom	1. Strongly Agree<br>2. Agree<br>3. Unsure<br>4. Disagree<br>5. Strongly Disagree<br><br>88. Refuse to say
j11	A man and a woman should decide together what type of contraceptive to use	1. Strongly Agree<br>2. Agree<br>3. Unsure<br>4. Disagree<br>5. Strongly Disagree<br><br>88. Refuse to say
j12	I would never have a homosexual friend	1. Strongly Agree<br>2. Agree<br>3. Unsure<br>4. Disagree<br>5. Strongly Disagree<br><br>88. Refuse to say

For peer review only

j13a	If someone insults me, I will defend my reputation, with force if I have to.	1. Strongly Agree<br>2. Agree<br>3. Unsure<br>4. Disagree<br>5. Strongly Disagree<br><br>88. Refuse to say
j13b	If someone insults a man, he should defend his reputation, with force if he has to	1. Strongly Agree<br>2. Agree<br>3. Unsure<br>4. Disagree<br>5. Strongly Disagree<br><br>88. Refuse to say
j14	To be a man you need to be tough.	1. Strongly Agree<br>2. Agree<br>3. Unsure<br>4. Disagree<br>5. Strongly Disagree<br><br>88. Refuse to say
j15	Men should be embarrassed if unable to get an erection	1. Strongly Agree<br>2. Agree<br>3. Unsure<br>4. Disagree<br>5. Strongly Disagree<br><br>88. Refuse to say
j16	If a guy gets a woman pregnant, child is the responsibility of both the man and woman	1. Strongly Agree<br>2. Agree<br>3. Unsure<br>4. Disagree<br>5. Strongly Disagree<br><br>88. Refuse to say
j17	A man should know what his partner likes during sex	1. Strongly Agree<br>2. Agree<br>3. Unsure<br>4. Disagree<br>5. Strongly Disagree<br><br>88. Refuse to say
j18	The participation of the father is important in raising children	1. Strongly Agree<br>2. Agree<br>3. Unsure<br>4. Disagree

For peer review only

		5. Strongly Disagree<br>88. Refuse to say
j19	It's important for men to have friends to talk about their problems	1. Strongly Agree<br>2. Agree<br>3. Unsure<br>4. Disagree<br>5. Strongly Disagree<br>88. Refuse to say
j20	A couple should decide together if they want to have children.	1. Strongly Agree<br>2. Agree<br>3. Unsure<br>4. Disagree<br>5. Strongly Disagree<br>88. Refuse to say
STIGMA		
Note	In this next section I'd like to discuss your thoughts about people living with HIV in your community. Please feel free to talk openly, there is no right or wrong answer. I am interested in your own thoughts.	
i3	I would buy fresh vegetables from a shopkeeper or vendor if I knew that this person had HIV	1. Strongly Agree<br>2. Agree<br>3. Neutral<br>4. Disagree<br>5. Strongly Disagree
i4	If a member of my family became sick with AIDS, I would be willing to care for her or him in our own household	1. Strongly Agree<br>2. Agree<br>3. Neutral<br>4. Disagree<br>5. Strongly Disagree
i5	In my opinion, if a female teacher has HIV but is not sick, she should be allowed to continue teaching in the school	1. Strongly Agree<br>2. Agree<br>3. Neutral<br>4. Disagree<br>5. Strongly Disagree
EXPECTATIONS		

For peer review only

Intro Note	I would like to ask you questions about [probability/chance/likelihood] that certain things will happen. There are ten beans in this cup. I will ask you to pick some of the beans and put them in the plate. The number of beans that you are going to put in the place will reflect the probability that something will happen. One beans means there is very little chance that something will happen. If you do not put any bean in the plate it means you are certain that there is no likelihood that something will happen.	
h4	Pick the number of beans that reflects how likely you think it is that:	______________________
h4b	You will have to rely on family members for financial assistance in the next 12 months.	______________________
h4c	You will have to provide some family members with financial assistance in the next 12 months.	______________________
Note	Next, I would like to ask you a few questions about what you expect in the future. I know that nobody knows for sure what the future may bring, but let`s just talk about your best guess.	
h5	In the next year how likely is it that you will:	
h5a	You will be enrolled in school one year from now	______________________
h5b	Start a new business?	______________________
h5c	Open a bank account?	______________________
h5d	Buy land?	______________________

For peer review only

h5e	Save money?	______________________
h5f	Experience shortage of food?	______________________
h5g	Have steady work?	______________________
	Tested for HIV	
pd2	Approximately how many times have you ever been tested for HIV? *Enter "-99" if client doesn't remember*	______________________
pd3	When was the last time you were tested for HIV?	a. Year______<br>b. Month________
pd5	Think about the very first time you received an HIV+ test result. Since that very first HIV+ test result, have you ever tested for HIV again (excluding a confirmatory test)?	1. Yes<br>0. No
pd6	Have you ever initiated ART?	1. Yes<br>0. No
	First Initiated ART	
pd6b	When did you first initiate ART?	a. Year_______<br>b. Month____
pd7	Have you ever been >14 days late for an ART appointment?	1. Yes<br>0. No
pd7b	How many times?	______________________
pd8	Do you know anyone who is on ART?	1. Yes<br>0. No
pd8b	Now think about the person on ART who you are closest with.<br><br>How often do you talk with them	1. Everyday<br>2. A couple times a week<br>3. Once a week<br>4. A couple times a month

	about ART?	5. Once a month<br>6. Less than once a month<br>7. Not at all (never)
pd9	Have you disclosed your HIV status to anyone besides your partner?	1. Yes<br>0. No
pd9b	Who else did you disclose to?<br>*Mark all that apply*	1. Sister<br>2. Brother<br>3. Father<br>4. Mother<br>5. Uncle<br>6. Aunt<br>7. Friend<br>8. Mother-in-Law<br>9. Father-in-Law<br>10. My children<br>11. Employee<br>12. Other sexual partner<br>99. Other, specify_____
pd10	Of those people you disclosed to, who do you talk to most often?	1. Sister<br>2. Brother<br>3. Father<br>4. Mother<br>5. Uncle<br>6. Aunt<br>7. Friend<br>8. Mother-in-Law<br>9. Father-in-Law<br>10. My children<br>11. Employee<br>12. Other sexual Partner<br>99. Other, specify_____
pd10c	How often do you talk to that person?	1. Everyday<br>2. A couple times a week<br>3. Once a week<br>4. A couple times a month<br>5. Once a month<br>6. Less than once a month<br>7. Not at all (never)

For peer review only

PREVIOUS USE OF HEALTH SERVICES		
Intro Note	Now I'd like to talk to you about your experience with using health services at health facilities.	
h1	Have you gone to a health facility in the past 12 months (either for yourself or someone else - AKA as a guardian)?	1. Yes<br>0. No
h2	How many times have you gone to the health facility in the past 12 months?	______________________
h3	Now think about yourself specifically. How many times have you gone to a health facility in the past 12 months for your own health care?	______________________
h_a1	When was the last time (the YEAR) you went to a health facility for YOUR OWN health?<br>NOTE: PUT WHAT YEAR. (i.e., 2015). If DO NOT REMEMBER, help them estimate. IF NEVER GONE, put -99	______________________
h4	What services did you receive at your last health facility visit for your own health?	1. ANC<br>2. Family Planning<br>3. Delivery<br>4. Post-natal<br>5. Under Five<br>6. HTC<br>7. ART<br>8. Feeling sick (OPD)<br>9. Injury (OPD)<br>10. Dentist<br>11. None<br>99. Other specify_____
h_a2	Now think about the SECOND most recent time you went to a health facility for YOUR OWN health. What year did you go to the health facility?	______________________
c3	Now please think about your MOST RECENT visit to a health facility, excluding today. When did you go?	a. Year___________<br>b. Month_________

For peer review only

c4	Which facility did you go to?	1. Current facility<br>0. Other facility, specify________________
c5	What was the main service you went for?	1. ANC<br>2. Family Planning<br>3. Delivery<br>4. Post-natal<br>5. Under Five<br>6. HTC<br>7. ART<br>8. Feeling sick (OPD)<br>9. Injury (OPD)<br>10. Dentist<br>11. None<br>99. Other specify_____
c5b	Who received services?	1. Myself<br>2. My child<br>3. My partner<br>4. Another family member<br>5. A friend<br>99. Other, specify_______
c6a	Did you (or the person you came with) receive another service?	1. Yes<br>0. No
c6	What was the second service you went for?	1. ANC<br>2. Family Planning<br>3. Delivery<br>4. Post-natal<br>5. Under Five<br>6. HTC<br>7. ART<br>8. Feeling sick (OPD)<br>9. Injury (OPD)<br>10. Dentist<br>11. None<br>99. Other specify_____
c6b	Who received services?	1. Myself<br>2. My child<br>3. My partner<br>4. Another family member<br>5. A friend<br>99. Other, specify_______

For peer review only

Service Satisfaction		
c10	Now I would like to talk to you about your satisfaction with the services you received that day. Please tell me whether any of these were problems for you at the VISIT YOU ARE THINKING ABOUT NOW, and if so, whether they were major or minor problems for you.	
c10_1	Time you waited to see a provider	1. Major<br>2. Minor<br>0. No-problem<br>88. Not applicable<br>89. Don't know
c10_2	Ability to discuss problems or concerns about your pregnancy	1. Major<br>2. Minor<br>0. No-problem<br>88. Not applicable<br>89. Don't know
c10_3	Amount of explanation you received about the problem or treatment	1. Major<br>2. Minor<br>0. No-problem<br>88. Not applicable<br>89. Don't know
c10_4	Privacy from having others see the examination	1. Major<br>2. Minor<br>0. No-problem<br>88. Not applicable<br>89. Don't know
c10_5	Privacy from having others hear your consultation discussion	1. Major<br>2. Minor<br>0. No-problem<br>88. Not applicable<br>89. Don't know
c10_6	Availability of medicines at this facility	1. Major<br>2. Minor

For peer review only

		0. No-problem<br>88. Not applicable<br>89. Don't know
c10_7	The hours of service at this facility, i.e., when they open and close	1. Major<br>2. Minor<br>0. No-problem<br>88. Not applicable<br>89. Don't know
c10_8	The number of days services are available to you	1. Major<br>2. Minor<br>0. No-problem<br>88. Not applicable<br>89. Don't know
c10_9	The cleanliness of the facility	1. Major<br>2. Minor<br>0. No-problem<br>88. Not applicable<br>89. Don't know
c10_10	How the staff treated you	1. Major<br>2. Minor<br>0. No-problem<br>88. Not applicable<br>89. Don't know
c10_11	Cost for services or treatments	1. Major<br>2. Minor<br>0. No-problem<br>88. Not applicable<br>89. Don't know
Satisfaction		
c11	In general, which of the following statements best describes your opinion of the services you either received or were provided at the facility	1. I am very satisfied with the services I received<br>2. I am satisfied with the services I received<br>3. I am not satisfied with the services I received

For peer review only

		4. I am very dissatisfied with the services I received
c12	Did you recommend this health facility to a friend or family member?	1. Yes<br>0. No
Comment	We have reached the end of the chat. Thank you for your time. Do you have anything else you would like to say?	
End Note	Thank the participant for their time and give them transport reimbursement, if they did not come for an ART appointment	
Comments	Enumerator comments	
End of the survey!		

For peer review only

BASELINE SURVEY

Identifying efficient linkage strategies for HIV self-testing (IDEaL)

Male (Chichewa)

**Question Name**	**Label**	**Responses**
INTRODUCTION SECTION		
interviewer	Full Name of Interviewer	
Interview date	Interview date	
Time start	Time survey started	
District	District	
Facility	Facility	
ID	ID	
SECTION A: DEMOGRAPHICS		
Intro Note	Zikomo povomera kutenga nawo mbali. Pa nthawi ino ndikufunsani ma funso ochepa okhudza za inu komanso kuti ndinu ndani. Chonde khalani omasuka kuyankha moona. Palibe yankho lokhoza komanso lolakwa.	
a7	Ndinu mtundu wanji wa munthu?	9. Lomwe<br>10. Sena<br>11. Chewa<br>12. Mang'anja/Nyanja<br>13. Ngoni<br>14. Tumbuka

For peer review only

		15. Tonga<br>16. Yao<br>99. Other, specify
a3	Kodi maphunziro anu mudapita nawo patali bwanji?	1. Pulayimale<br>2. Sekondale<br>3. Koleji
a3b	Ndi kalasi liti munamaliza ya maphunziro anu apamwamba?	___________________
a4	Chonde ganizani za masabata khumi ndi awiri apitawa, mungafotokoze bwanji za ntchito yomwe mumagwira?	1. Ntchito yokhazikika (ya nthawi yayitali)<br>2. Ntchito yosakhazikika (ganyu, ulimi, bizinesi)<br>3. Sindikugwira ntchito
a5	Kodi pakadali pano muli pa banja?	1. Pa banja<br>2. Kukhala limodzi ngati banja<br>3. Chibwezi Chokhazikika<br>4. Tinasiyana<br>5. Banja linatha<br>99. Zina
a6	Muli ndi ana angati amoyo?	___________________
a6b	Mwana wanu wang'ono ali ndi zaka zingati?	___________________
a6bc	Mwana wanu wang'ono ali ndi zaka zingati?	___________________
a4b	Pakadali pano ana omwe mumakhala nawo ndi angati?	___________________
a7	Pa miyezi khumi ndi iwiri yapitayi mwakhala ndi abwenzi ogonana nawo angati?	___________________
a8	Mwakhalapo ndi bwenzi logonana kupatula akazi anu?amuna anu osagwiritsa ntchito kondomu mu miyezi khumi ndi awiri yapitayi?	1. Eya<br>2. Ayi<br>88. Sindikudziwa<br>89. Akana kuyankha

For peer review only

a8b	Mwagonanapo ndi munthu osagwiritsa ntchito kondomu mu miyezi khumi ndi awiri yapitayi?	1. Eya<br>2. Ayi<br>88. Sindikudziwa<br>89. Akana kuyankha
SECTION B: INCOME QUESTIONS		
Intro Note	Pa nthawi ino ndikufunsani za zipangizo zomwe inu komanso apabanja panu alinazo. Mkati mwakucheza kwathu ndikufunsaninso za ndalama zomwe mulinazo komanso komanso zomwe mumachita kuti mupeze ndalama.	
b1	Chonde ganizirani za miyezi Khumi ndi iwiri yapitayi, mungafotokoze bwanji za ntchito yomwe mumagwira?	4. Ntchito yokhazikika<br>5. Ganyu/bisinesi<br>6. Sindikugwira ntchito
b1b	Ganizani za ntchito zonse mwagwira mwezi watha. Mwagwira masiku angati olipidwa?	__________________
b2	Muli ndi ndalama zilizonse zomwe mukusungira za mtsogolo monga, ku banki, gulu losugira ndalama kapena ndalama zosunga kunyumba?	2. Eya<br>3. Ayi
Household Assets		
b3	Does your household have:<br>*The respondent said that his/her household doesn't have any of the household assets. Please probe and ensure that this is correct before you proceed.*	
b3_1	Denga la malata?	1. Eya<br>2. Ayi
b3_2	Magetsi ?	1. Eya<br>2. Ayi
b3_3	Koloboyi?	1. Eya<br>2. Ayi
b3_4	Nyali?	1. Eya<br>2. Ayi

For peer review only

b3_5	Wailesi?	1. Eya<br>2. Ayi
b3_6	Kanema?	1. Eya<br>2. Ayi
b3_7	Lamya ya M'manja?	1. Eya<br>2. Ayi
b3_8	Kama?	1. Eya<br>2. Ayi
b3_9	Sofa?	1. Eya<br>2. Ayi
b3_10	Tebulo?	1. Eya<br>2. Ayi
b3_11	FIliji?	1. Eya<br>2. Ayi
b3_12	Matilesi?	1. Eya<br>2. Ayi
b3_13	Mipando	1. Eya<br>2. Ayi
b3_14	Ng'ombe?	1. Eya<br>2. Ayi
b3_15	Mbuzi?	1. Eya<br>2. Ayi
b3_16	Nkhosa?	1. Eya<br>2. Ayi
b3_17	Nkhumba?	1. Eya<br>2. Ayi
b3_18	Bulu?	1. Eya<br>2. Ayi
b3_19	Nkhuku?	1. Eya<br>2. Ayi
b3_20	Zoweta zina	1. Eya<br>2. Ayi
b4	Mu masiku makumi atatu apitawa mwamako mowa?	1. Eya<br>2. Ayi
b4b	Pa masiku makumi atatu apitawa mwamwa mowa masiku angati?	______________________
b4c	Mwataya ndalama zingati masiku omaliza omwe munapita ku mowa?	MWK:
b4d	Zonse pamodzi, mwataya ndalama zingati pa mowa mu masiku makumi atatu apitawa?	MWK:
Relationship		

For peer review only

Intro Note	Panthawi ino ndikufunsani za abwezi ogonana nawo?	
f8	Mwakhala pa ubwenzi ogonana ndi bwenzi lanu kwa nthawi yayitali bwanji?	Masiku ___________<br>Miyezi _____________<br>Zaka________________
f9	Muli ndi ana ndi bwenzi lanu logonana nalo? Ana angati?	______________________
f10	Mumayankhulana mowirikiza bwanji ndi bwenzi lanu pakadali pano?	1. Tsiku ndi tsiku<br>2. Masiku angapo pasabata<br>3. Kamodzi pa sabata<br>4. Kangapo pa mwezi<br>5. Kamodzi pa mwezi<br>6. Kosakwana mwezi<br>7. Sitiyankhulani
f10b	Pa mwezi amalandila ndalama zambiri ndi ndani? Inu kapena bwenzi lanu?	1. Ine<br>2. Bwenzi langa<br>3. Timalandira ndalama zofanana<br>4. Sindikudziwa
Decision Making		
Intro Note	Pa nthawi ino ndikufunsani za momwe mumapangira maganizo ndi bwenzi lanu	
f11	Nthawi zambiri ndi ndani amene amapanga chiganizo cha momwe ndalama mumapeze zigwiritsidwe ntchito?	1. Ine<br>2. Timagwirizana<br>3. Nthawi zambiri bwenzi langa<br>4. Munthu wina<br>5. Sindipeza ndalama<br>88. Sindikufuna kuyankha
f11b	(Ngati yankho ndi 4) Amapanga chiganizo ndi ndani?	______________________
f12	Amapanga chiganizo cha momwe ndalama za bwenzi lanu zigwiritsidwe ntchito ndi ndani?	1. Ine<br>2. Timagwirizana<br>3. Nthawi zambiri bwenzi langa<br>4. Munthu wina<br>5. Sindipeza ndalama

For peer review only

		88. Sindikufuna kuyankha
f12b	(Ngati yankho ndi 4)	____________________
f13	Nthawi zambiri amapanga chiganizo chokhudza thandizo la zaumoyo wanu ndi ndani?	1. Ine<br>2. Timagwirizana<br>3. Nthawi zambiri bwenzi langa<br>4. Munthu wina<br>5. Sindipeza ndalama<br>88. Sindikufuna kuyankha
f13b	(Ngati yankho ndi 4) Amapanga chiganizo ndi ndani?	____________________
f14	Nthawi zambiri amapanga chiganizo cha thandizo la zaumoyo la mwana yemwe muli naye ndi bwenzi lanu ndi ndani?	6. Ine<br>7. Mogwirizana<br>8. Nthawi zambiri bwenzi langa<br>9. Munthu wina<br>10. Ndilibe mwana<br>88. Sindikufuna kuyankha
f14b	(if above question=4) Who decides?	____________________
f15	Nthawi zambiri amapanga chiganizo chokhudza thandizo la zaumoyo la bwenzi lanu ndi ndani?	1. Ine<br>2. Mogwirizana<br>3. Nthawi zambiri bwenzi langa<br>4. Munthu wina<br>5. Ndilibe ana<br>88.Sindikufuna kuyankha
f15b	(Ngati yankho ndi 4) Amapanga chiganizo ndi ndani?	____________________
f16	Kodi amapanga ziganizo zogula katundu mkulumkulu wapakhomo panu ndi ndani?	1. Ine<br>2. Mogwirizana<br>3. Nthawi zambiri bwenzi langa

For peer review only

		4. Munthu wina<br>5. Ndilibe ana<br>88.Sindikufuna kuyankha
f16b	(Ngati yankho ndi 4) Amapanga chiganizo ndani?	
Note	Ndikufuna ndikufunseni zokhudzana za (Kuthekela/mwayi) kuti zinthu zina zichitike. Muli nyemba mu kapu. Ndikufunsani kuti musankhe zina mwa nyemba ndipo muyike mu mbale. Mulingo wa nyemba omwe muyike mu mbale udzafanizira kuti chinachake chichitika. Nyemba imodzi ikusonyeza kuti mwayi ndiochepa kuti chinachake chichitika. Ngati simuika nyemba mm'bale ndekuti mukutsimikiza kuti palibe mwayi oti chinachake chichitika	
note2	Ngati muyike nyemba zowonjezera mu mbale, zikutanthauza kuti mwayi oti chinachake chichitika uchuluka, mwachitsanzo ngati muyika nyemba imodzi kapena ziwiri mwayi oti chinachake chichitika. Ngakhale pali mwayi ochepa koma chinachake chichitika. Ngati muyike nyemba nkhumi zikutanthauza kuti pali mwayi ofanana oti chinachake chichika kapena ayi. Ngati muyike nyemba zisanu ndi imodzi zikutanthauza kuti mwayi woti chinachake chichitika uli ochulukilapo kuposa mwayi oti chinachake sichichitika. Ngati muyike nyemba zonse khumi ndekuti muli ndi chikhulupiliro chonse kuti chinachake chichitikadi pavute pasavute. Palibe yankho lokhonza kapena lolakwa ndikungofuna kudziwa zomwe mukuganiza.	

For peer review only

note3	Ofunsa: Pelekani yankho lanu pa funso lililonse nambala ya nyemba zomwe lili m'bale. Pakutha pa funso lililonse, bwezeletsani nyemba pa tebulo.	
Practice		
pr1	Sankhani mulingo wa nyemba omwe ukhale ndi kuthekela kumene mukuganiza kuti:	________________
pr1b	Mupita ku msika mosachepera kamodzi m'masiku awiri akudzawa	________________
pr1c	Mupita ku msika mosachepera kamodzi m'masabata awiri akudzawa.	________________
Practice		
pr2	Ofunsa: kodi oyankha anawonjezera nyamba pakati pa pr1b ndi pr1c	2. Eya<br>0. Ayi
pr3	Kumbukurani kuti pamene nthawi ikupita muzipeza mpata wambiri opita kunsika. Choncho, munayenela kuti mwaika nyemba zambiri m'bale<br><br>Kodi mukuganiza kuti kumsika mupita mosachepela kamodzi bwanji mu nyengo ya ma sabata awiriwa?	________________
f17	Sankhani mlingo wa nyemba umene ufanizile kaganidwe kanu:	
f17b	Mukhalabe mukanali pa banja/ndi (bwenzi oposela chaka chimodzi kuchoka pano	________________
f17c	Mudzadwala mu miyezi khumi ndi iwiri ikubwelayi	________________
f17d	Mudzayamba kumwa ma ARV mu miyezi itatu ikubwelayi	________________

For peer review only

f17e	Mudzadziwitsa achibale ndi anzanu za zotsatira zanu za HIV m'miyezi itatu ikubwelayi	______________
f17c	Mukusafuna kwanu, anzanu ndi abale azadziwa kuti muli ndi HIV, mu miyezi itatu yapitayi.	______________
SELF REPORTED HEALTH AND HAPPINESS		
Intro Note	Pa nthawi ino ndikufuna ndikambe nanu zokhudza umoyo ndi chisangalalo chanu.	
e1	Ndili ndi chidwi ndi mukudziwa za umoyo ndi kukhutitsidwa kwanu. Kodi muli okhutitsidwa bwanji ndi moyo wanu, pakutengela zonse.	6. Okhutitsidwa kwamburi<br>7. Okhutitsidwa pang'ono<br>8. Pakatikati<br>9. Okhutitsidwa pang'ono<br>10. Osakhutitsidwa olo pang'ono
e2	Kodi mukuona ngati muli okhutitsidwa mofanana kapena osakhutitsidwa pang'ono mosaposela anthu ena a muna kapena akazi a msinkhu wanu ndi opezeka m'mudzi mwanu?	1. Okhutitsidwa kwambiri<br>2. Okhutiotsidwa<br>3. Okhutitsidwa pang'ono
e3	Kutengela zonse, Kodi munganene kuti umoyo wanu tsopano uli bwino kwambiri, ulibwino, sulibwino, suli bwino olo pang'ono	1. Bwino kwambiri<br>2. Bwino<br>3. Silibwino<br>4. SIlibwino olo pang'ono
e4	Kodi mungazifananizile bwanji za thanzi lanu ndi la anthu ena a msikhu ofanana ndi wanu, amuna kapena akazi a m'mudzi mwanu.	1. A thanzi kwambiri<br>2. A thanzi<br>3. Nthanzi lochepekela
e5	Kodi mwezi wathawu, ndi masiku angati omwe munadwara kwambiri ofika kukulepheretsani kugwira ncthito/kupita ku sukulu/kugwira ntchito za pa khomo	______________
Happiness		
e6	Kodi ziganizo izi ndi zowona bwanji kwa inu?	
e6_1	Ndinali ndi nkhawa	1. Ndikugwirizana nazo kwambiri

For peer review only

		2. Ndikugwirizana nazo<br>3. Sindikugwirizana nazo<br>4. Sindikugwirizana nazo kwambiri
e6_2	Ndimanva ngati moyo wafika	1. Ndikugwirizana nazo kwambiri<br>2. Ndikugwirizana nazo<br>3. Sindikugwirizana nazo<br>4. Sindikugwirizana nazo kwambiri
e6_3	Ndimanva kukhutitsidwa	1. Ndikugwirizana nazo kwambiri<br>2. Ndikugwirizana nazo<br>3. Sindikugwirizana nazo<br>4. Sindikugwirizana nazo kwambiri
e6_4	Ndimanva kusalidwa	1. Ndikugwirizana nazo kwambiri<br>2. Ndikugwirizana nazo<br>3. Sindikugwirizana nazo<br>4. Sindikugwirizana nazo kwambiri
GENDER EQUITABLE MEN SCALE		
Note	Chonde ndiuzeni ngati mukugwirizana nazo kwabiri, mukugwirizana nazo, simukugwirizana nazo, simukugwirizana nazo olo pang'ono ziganizo izi:	
j1	Udindo ofunikila wa mzimayi ndi kusamala khomo lake ndi kuphika.	1. Ndikugwirizana nazo kwambiri<br>2. Ndikugwirizana nazo<br>3. Sindikukhulupilira<br>4. Sindikugwirizana nazo<br>5. Sindikugwirizana nazo olo pang'ono<br>88. Sindikufuna kuyankha
j2	Abambo amafuna kugonana kuposa amayi	1. Ndikugwirizana nazo kwambiri<br>2. Ndikugwirizana nazo<br>3. Sindikukhulupilira<br>4. Sindikugwirizana nazo

For peer review only

		5. Sindikugwirizana nazo olo pang'ono<br>88. Sindikufuna kuyankha
j3	Azibambo sakonda kukambirana za kugonana amangochita	1. Ndikugwirizana nazo kwambiri<br>2. Ndikugwirizana nazo<br>3. Sindikukhulupilira<br>4. Sindikugwirizana nazo<br>5. Sindikugwirizana nazo olo pang'ono<br>88. Sindikufuna kuyankha
j4	Nthawi zina mzimayi amayenela kumenyedwa.	1. Ndikugwirizana nazo kwambiri<br>2. Ndikugwirizana nazo<br>3. Sindikukhulupilira<br>4. Sindikugwirizana nazo<br>5. Sindikugwirizana nazo olo pang'ono<br>88. Sindikufuna kuyankha
j5	Kusinta matewela, kusambitsa mwana, kudyetsa mwana ndi udindo wa mzimayi	1. Ndikugwirizana nazo kwambiri<br>2. Ndikugwirizana nazo<br>3. Sindikukhulupilira<br>4. Sindikugwirizana nazo<br>5. Sindikugwirizana nazo olo pang'ono<br>88. Sindikufuna kuyankha
j6	Ndi udindi wa mzimayi kupewa kutenga mimba.	1. Ndikugwirizana nazo kwambiri<br>2. Ndikugwirizana nazo<br>3. Sindikukhulupilira<br>4. Sindikugwirizana nazo<br>5. Sindikugwirizana nazo olo pang'ono<br>88. Sindikufuna kuyankha
j7	Mzibambo ayenela kukhala ndi chiganizo chomaliza cha mnyumba mwake.	1. Ndikugwirizana nazo kwambiri<br>2. Ndikugwirizana nazo<br>3. Sindikukhulupilira<br>4. Sindikugwirizana nazo

For peer review only

		5. Sindikugwirizana nazo olo pang'ono<br>88. Sindikufuna kuyankha
j8	Azibambo amakhala okonzeka ku gonana	1. Ndikugwirizana nazo kwambiri<br>2. Ndikugwirizana nazo<br>3. Sindikukhulupilira<br>4. Sindikugwirizana nazo<br>5. Sindikugwirizana nazo olo pang'ono<br>Sindikufuna kuyankha
j9	Mzimayi akuyenela kulekelela nkhaza kuti asunge banja lake.	1. Ndikugwirizana nazo kwambiri<br>2. Ndikugwirizana nazo<br>3. Sindikukhulupilira<br>4. Sindikugwirizana nazo<br>5. Sindikugwirizana nazo olo pang'ono<br>Sindikufuna kuyankha
j10a	Ndikhoza kukwiya akaza anga atati andiuze kuti ndigwiritse ntchito kondumu.	1. Ndikugwirizana nazo kwambiri<br>2. Ndikugwirizana nazo<br>3. Sindikukhulupilira<br>4. Sindikugwirizana nazo<br>5. Sindikugwirizana nazo olo pang'ono<br>88. Sindikufuna kuyankha
j10b	Azibambo akhoza kukwiya akazi awo atawauza kuti agwiritse ntchito kondomu	1. Ndikugwirizana nazo kwambiri<br>2. Ndikugwirizana nazo<br>3. Sindikukhulupilira<br>4. Sindikugwirizana nazo<br>5. Sindikugwirizana nazo olo pang'ono<br>88. Sindikufuna kuyankha
j11	Mzimayi komanso nzibambo agwirizane limodzi kuti agwiritse ntchito njira yanji yakulela	1. Ndikugwirizana nazo kwambiri<br>2. Ndikugwirizana nazo<br>3. Sindikukhulupilira<br>4. Sindikugwirizana nazo

For peer review only

		5. Sindikugwirizana nazo olo pang'ono<br><br>88. Sindikufuna kuyankha
j12	Sindingakhale ndi nzanga wopanga zamathanyula	1. Ndikugwirizana nazo kwambiri<br>2. Ndikugwirizana nazo<br>3. Sindikukhulupilira<br>4. Sindikugwirizana nazo<br>5. Sindikugwirizana nazo olo pang'ono<br><br>88. Sindikufuna kuyankha
j13a	Ngati munthu angandinyoze, ndiziteteza pogwiritsa ntchito mphanvu, ngati ndikufunika kutero.	1. Ndikugwirizana nazo kwambiri<br>2. Ndikugwirizana nazo<br>3. Sindikukhulupilira<br>4. Sindikugwirizana nazo<br>5. Sindikugwirizana nazo olo pang'ono<br><br>88. Sindikufuna kuyankha
j13b	Ngati munthu anganyoze mzibambo, aziteteze pogwiritsa ntchito mphanvu ngati akufunika kutelo.	1. Ndikugwirizana nazo kwambiri<br>2. Ndikugwirizana nazo<br>3. Sindikukhulupilira<br>4. Sindikugwirizana nazo<br>5. Sindikugwirizana nazo olo pang'ono<br><br>Sindikufuna kuyankha
j14	Kuti ukhale mzibambo ukufunika kukhala ovuta.	1. Ndikugwirizana nazo kwambiri<br>2. Ndikugwirizana nazo<br>3. Sindikukhulupilira<br>4. Sindikugwirizana nazo<br>5. Sindikugwirizana nazo olo pang'ono<br>Sindikufuna kuyankha<br>Sindikugwirizana nazo kwambiri<br><br>88. Refuse to say<br><br>Sindikufuna kuyankha
j15	Azibambo akuyenela kunva manyazi ngati akukanika kutota	1. Ndikugwirizana nazo kwambiri<br>2. Ndikugwirizana nazo

		3. Sindikukhulupilira<br>4. Sindikugwirizana nazo<br>5. Sindikugwirizana nazo olo pang'ono<br><br>88. Sindikufuna kuyankha
j16	Ngati mzibambo wapeleka mimba kwa mzimayi, mwanayo ndi udindo wa anthu onse a wiri.	1. Ndikugwirizana nazo kwambiri<br>2. Ndikugwirizana nazo<br>3. Sindikukhulupilira<br>4. Sindikugwirizana nazo<br>5. Sindikugwirizana nazo olo pang'ono<br><br>88. Sindikufuna kuyankha
j17	Nzibambo akuyenela kudziwa zomwe bwenzi lake limakonda pogonana	1. Ndikugwirizana nazo kwambiri<br>2. Ndikugwirizana nazo<br>3. Sindikukhulupilira<br>4. Sindikugwirizana nazo<br>5. Sindikugwirizana nazo olo pang'ono<br><br>88. Sindikufuna kuyankha
j18	Kutenga nawo mbali kwa a bambo ndi kofunika polela mwana	1. Ndikugwirizana nazo kwambiri<br>2. Ndikugwirizana nazo<br>3. Sindikukhulupilira<br>4. Sindikugwirizana nazo<br>5. Sindikugwirizana nazo olo pang'ono<br><br>88. Sindikufuna kuyankha
j19	Ndi zofunika kuti abambo azikhala ndi anzawo okambilana nawo mavuto awo.	1. Ndikugwirizana nazo kwambiri<br>2. Ndikugwirizana nazo<br>3. Sindikukhulupilira<br>4. Sindikugwirizana nazo<br>5. Sindikugwirizana nazo olo pang'ono<br><br>88. Sindikufuna kuyankha
j20	Banja lizigwirizana limodzi ngati likufuna kukhala ndi mwana	1. Ndikugwirizana nazo kwambiri<br>2. Ndikugwirizana nazo<br>3. Sindikukhulupilira<br>4. Sindikugwirizana nazo

For peer review only

		5. Sindikugwirizana nazo olo pang'ono<br>88. Sindikufuna kuyankha
STIGMA		
Note	Mugawo lotsatila ndifuna tikambilane zokhudza maganizo anu a anthu omwe ali ndi HIV komanso akukhala mu dela lanu. Chonde khalani omasuka kuyankhula momasuka, palibe yankho lohoza kapena lolakwa. Ndikufuna ndinve maganizo anu.	
i3	Ndikhoza kugula ndiwo zamasamba kwa munthu oti ndikudziwa kuti ali ndi HIV.	1. Ndikugwirizana zano kwambiri<br>2. Ndikugwirizana nazo<br>3. Pakatikati<br>4. Sindikugwurizana nazo<br>5. Sindikugwirizana nazo kwambiri
i4	Ngati wachibale wanga angadwale AIDS, ndingavomele kumusamala pakhomo panga.	1. Ndikugwirizana zano kwambiri<br>2. Ndikugwirizana nazo<br>3. Pakatikati<br>4. Sindikugwurizana nazo<br>5. Sindikugwirizana nazo kwambiri
i5	M'maganizo mwanga, ngati mphunzitsi wa mkazi ali ndi HIV koma sakudwala, aloledwe kupitiliza kuphunzitsa.	1. Ndikugwirizana zano kwambiri<br>2. Ndikugwirizana nazo<br>3. Pakatikati<br>4. Sindikugwurizana nazo<br>5. Sindikugwirizana nazo kwambiri
EXPECTATIONS		

For peer review only

Intro Note	Ndikufuna ndikufunseni zokhudzana za (Kuthekela/mwayi) kuti zinthu zina zichitike. Muli nyemba mu kapu. Ndikufunsani kuti musankhe zina mwa nyemba ndipo muyike mu mbale. Mulingo wa nyemba omwe muyike mu mbale udzafanizira kuti chinachake chichitika. Nyemba imodzi ikusonyeza kuti mwayi ndiochepa kuti chinachake chichitika. Ngati simuika nyemba mm'bale ndekuti mukutsimikiza kuti palibe mwayi oti chinachake chichitika	
h4	Sankhani mulingo wa nyemba omwe ukhale ndi kuthekela kumene mukuganiza kuti:	______________
h4b	Mudzakhala mukudalila apabanja panu pa nkhani ya zachuma mu miyezi itatu ikubwelayi.	______________
h4c	Mukhala mukuthandiza achibale ena pa nkhani za chuma miyezi itatu ikubweyi.	______________
Note	Kotsatira ndikufuna ndikufunseni mafunse ochepa okhudza chiyembekezo chanu cha mtsogolo. Ndikudziwa palibe yemmwe amadziwa za mtsogolo koma tiyeni tikambe mongoyelezeka.	
h5	Mu chaka chamawa chiyembekezero choti mudzakhala mu:	
h5a	Mudzakhala mutayamba sukulukuchoka lelo chaka chamawa	______________
h5b	Kuyamba buzinesi	______________
h5c	Kutsegula akaunti ku banki	______________
h5d	Ku gula malo?	______________

For peer review only

h5e	Kusunga ndalama?	____________________
h5f	Kukhala ndi chakudya chochepa?	____________________
h5g	Kukhala pa ntchito yokhazikika	____________________
Tested for HIV		
pd2	Mwayezetsapo HIV kokwana kangati? *Enter "-99" if client doesn't remember*	____________________
pd3	Komaliza munayezetsa HIV kanali liti?	a) Chaka ______ b) Mwezi ________
pd5	Ganizani za ulendo wanu ayamba olandira zotsatira zakuyezadwa kwa HIV. Koyamba kulandira zotsatira zoti akupezani ndi HIV kanali liti?	a) Chaka ______ b) Mwezi ________
pd6	Have you ever initiated ART? Munayamba mwamapo ma ARV?	2. Eya 0. Ayi
First Initiated ART		
pd6b	Koyamba kumwa ma ARV kanali liti?	c. Chaka ______ d. Mwezi ________
pd7	Munayamba mwachedwako kukatenga mwankhwala masiku ochepela khumi ndi folo?	1. Eya 0. Ayi
pd7b	Masiku angati?	____________________
Pd7c	Munasiya kumwa ma ARV chifukwa chani?	____________________ ____________________
pd8	Mukudziwa munthu wina aliyense	

	yenwe akumwa ma ARV?	1. Eya<br>0. Ayi
pd8b	Pano ganizani za munthu yemwe akumwa ma ARV amene mulinali pafupi<br><br>Mumayankhula naye mowilikiza bwanji?	1. Siku ndi tsiku<br>2. Kangapo pa sabata<br>3. Kamodzi pa sabata<br>4. Kangapo pa sabata<br>5. Kamodzi kamwezi<br>6. Kochepera kamodzi pa mwezi<br>7. Sitiyankhulana
pd9	Munayamba mwaululapo za momwe nthupi mwanu mulili za HIV kwa anthu ena kupatura bwenzi lanu?	1. Eya<br>1. Ayi
pd9b	Wina munamuuza anali ndani?<br>*Mark all that apply*	1. Ntcemwali<br>2. Ntchimwene<br>3. Bambo anga<br>4. Mayi anga<br>5. Malume<br>6. Azakhali anga<br>7. Nzanga<br>8. Apongozi akazi<br>9. Apongozi amuna<br>10. Ana anga<br>11. Ogwira naye ntchito<br>12. Bwezi logonanalo<br>13. Zina, fotokozani
pd10	Pa munthu yemwe munamuwuza, ndi ndani yemwe mumayankhula naye kawirikawiri?	1. Ntcemwali<br>2. Ntchimwene<br>3. Bambo anga<br>4. Mayi anga<br>5. Malume<br>6. Azakhali anga<br>7. Nzanga<br>8. Apongozi akazi<br>9. Apongozi amuna<br>10. Ana anga<br>11. Ogwira naye ntchito<br>12. Bwezi logonanalo<br>Zina, fotokozani

For peer review only

pd10c	Muntjuyi mumayankhula naye mowirikiza bwanji?	1. Tsiku ndi tsiku<br>2. Kangapo pa sabata<br>3. Kamodzi pa sabata<br>4. Kangapo pa mwezi<br>5. Kamodzi pa mwezi<br>6. Kochepera kamodzi pa mwezi<br>7. Sitiyankhulana
PREVIOUS USE OF HEALTH SERVICES		
Intro Note	Pa ntahwi ino ndifuna tikambilane zokhudza za zomwe mwadutsamo pogwiritsa ntchito thandizo la za umoyo pa chipatala.	
h1	Munayamba mwapitako ku chipatala miyezi khumi ndi iwiri yapitayi.(chifukwa cha inu kapena kupelekeza munthu wina)?	1. Eya<br>0. Ayi
h2	Mwapita ku chipatala kangati miyezi khumi ndi iwiri yapitayi?	________________
h3	Pa nthawi ino ganizani za inu. Mwapita kangati ku chipatala miyezi khumi ndi awiri yapitayi panokha kukalandira thandizo la zaumoyo?	________________
h_a1	Komaliza kupita kuchipatala(chaka) chifukwa mwadwala ndinu kanali liti?<br>NOTE: PUT WHAT YEAR. (i.e., 2015). If DO NOT REMEMBER, help them estimate. IF NEVER GONE, put -99	________________
h4	Munalandira thandizo lanji ulendo umaliza munapita kuchipatala?	12. ANC<br>13. Family Planning<br>14. Delivery<br>15. Post-natal<br>16. Under Five Ku ana<br>17. Kukayezetsa HIV<br>18. ARV<br>19. (OPD) Kudwala<br>20. (OPD)Kuvulala<br>21. Kukonana ndi dotolo wa manu

For peer review only

		22. Palibe
h_a2	Tsopano ganizani za kachiwiri komwe munapita kuchipatala nokha cha posachedwapa. Munapita ku chiptala chaka chanji?	______________________
c3	Chonde ganizani za ulendo wanu munapita kuchipatala chaposachedwa, kuphatikizapo lelo. Munapita lelo?	c. Chaka___________<br>d. Mwezi_________<br>Mwezi
c4	Munapita ku chipatala chiti	1. Chipatala chomwe mumapita pakali pano<br>2. Chipatala china, tchulani
c5	Munapita kukalandila thandizo lanji ku chipatala?	1. ANC<br>2. Family Planning<br>3. Delivery<br>4. Post-natal<br>5. Under Five Ku ana<br>6. Kukayezetsa HIV<br>7. ARV<br>8. (OPD) Kudwala<br>9. (OPD)Kuvulala<br>10. Kukonana ndi dotolo wa manu<br>11. Palibe
c5b	Amene analandira thandizo la zaumoyo anali ndani?	1. Ineyo<br>2. Mwana wanga<br>3. Bwenzi langa<br>4. Wapabanja panga<br>5. Mzanga<br>99. Zina, fotokozani_______
c6a	Inu kapena munthu yemwe munapita naye kuchipatala analandila munthu wina wathandizo la zaumoyo?	2. Eya<br>0. Ayi
c6	Thandizo lachiwiri la zaumyo lomwe manalandira linali lanji?	1. ANC<br>2. Family Planning<br>3. Delivery<br>4. Post-natal<br>5. Under Five Ku ana<br>6. Kukayezetsa HIV<br>7. ARV<br>8. (OPD) Kudwala

For peer review only

		9. (OPD)Kuvulala<br>10. Kukonana ndi dotolo wa manu<br>11. Palibe
c6b	Analandira thandizo la zaumoyo anali ndani?	1. Ine<br>2. Mwana wanga<br>3. Bwezi langa<br>4. Wapabanja panga<br>5. Nzanga Zina, fotokozani
Service Satisfaction		
c10	Pa nthawi ino ndikufuna ndikufunseni za kukhutila kwanu ndi thandizo la zaumoyo munalandila pa tsikulo. Chinde nduuzeni ngai zina mwazotsatirazi zinli vito kwa inu patsiku lomwe munapita kuchipatala, ngati eya, ngati vutolo linali lalikulu kapena ayi.	
c10_1	Nthawi yomwe mumafuna kuonana ndi dotolo	1. Vuto kwambiri<br>2. Vuto pang'ono<br>3. Silinali vuto<br>88. Not applicable<br>98. Sindikudziwa
c10_2	Kuthekela kokambilana za mavuto anu a pakati.	1. Vuto kwambiri<br>2. Vuto pang'ono<br>0. Silinali vuto<br>88. Not applicable<br>98. Sindikudziwa
c10_3	Mulingo waku Kufotokozeledwa munalandira okhudza vuto lanu kapena thandizo.	1. Vuto kwambiri<br>2. Vuto pang'ono<br>0. Silinali vuto<br>88. Not applicable<br>98. Sindikudziwa
c10_4	Chinsinsi kuopetsa ena kuwona zotsatila za umoyo	1. Vuto kwambiri<br>2. Vuto pang'ono<br>0. Silinali vuto<br>88. Not applicable<br>98. Sindikudziwa

For peer review only

c10_5	Chinsinsi kuopa ena kunva za zokambilana zanu	1. Vuto kwambiri<br>2. Vuto pang'ono<br>0. Silinali vuto<br>88. Not applicable<br>98. Sindikudziwa
c10_6	Kupezeka kwa mankwala mzipatala	1. Vuto kwambiri<br>2. Vuto pang'ono<br>0. Silinali vuto<br>88. Not applicable<br>98. Sindikudziwa
c10_7	Ma ola omwe thandizo limapelekedwa pa chipatala i.e thawi yotsegulila ndi yotsekela	1. Vuto kwambiri<br>2. Vuto pang'ono<br>0. Silinali vuto<br>88. Not applicable<br>98. Sindikudziwa
c10_8	Masiku omwe mumalandila thandizo	1. Vuto kwambiri<br>2. Vuto pang'ono<br>0. Silinali vuto<br>88. Not applicable<br>98. Sindikudziwa Sindikudziwa
c10_9	Ukhondo wa pa chipatala	1. Vuto kwambiri<br>2. Vuto pang'ono<br>0. Silinali vuto<br>88. Not applicable<br>98. Sindikudziwa
c10_10	Momwe ogwira ntchito amakusamalilani	1. Vuto kwambiri<br>2. Vuto pang'ono<br>0. Silinali vuto<br>88. Not applicable<br>98. Sindikudziwa
c10_11	Mtengo wa thandizo ndi mankwala	1. Vuto kwambiri<br>2. Vuto pang'ono<br>1. Silinali vuto<br>88. Not applicable<br>98. Sindikudziwa

For peer review only

Satisfaction		
c11	Ndi chiganizo chiti chomwe chikufotokoza za maganizo anu okhudza thandizo lomwe munalandila pa chipatala?	1. Ndili okhutitsidwa kwabiri ndi thandizo ndinalandila.<br>2. Ndili okhutitsidwa ndi thandizo ndinalandila.<br>3. Sindili okhutitsidwa ndi thandizo ndinalandila<br>4. Sindili okhutitsidwa kwambiri ndi thandizo ndinalandila
c12	Munatchulako za chipatalachi kwa nzanu kapena wachibale?	2. Eya<br>0. Ayi
Comment	Tafika pamapeto a kucheza kwathu. Zikomo chifukwa cha nthawi yanu. Pali chili chonse mukufuna kuwonjezera?	
End Note	Thank the participant for their time and give them transport reimbursement, if they did not come for an ART appointment	
Comments	Enumerator comments	
End of the survey!		

For peer review only

14.6. APPENDIX F: Baseline survey – Female

BASELINE SURVEY
Identifying efficient linkage strategies for HIV self-testing (IDEaL)
Female

INTRODUCTION SECTION		
interviewer	Full Name of Interviewer	
Interview date	Interview date	
Time start	Time survey started	
District	District	
Facility	Facility	
ID	ID	

For peer review only

SECTION A: DEMOGRAPHICS		
Intro Note	Thank you for agreeing to participate. Now I will ask you a few questions about yourself and who you are. Please feel free to answer honestly. There are no right or wrong answers.	
a7	What is your tribe?	1. Lomwe<br>2. Sena<br>3. Chewa<br>4. Mang'anja/Nyanja<br>5. Ngoni<br>6. Tumbuka<br>7. Tonga<br>8. Yao<br>99. Other, specify
a3	What is the highest level of school you attended?	1. Primary<br>2. Secondary<br>3. Higher
a3b	What class did you complete in your highest level of school?	________________
a4	Please think of the past 12months, how would you describe your primary occupation?	1. Working formally (employed full time)<br>2. Working informally (ganyu, farming, business)<br>3. Not working
a5	Are you currently married?	1. Married<br>2. Live-in partner<br>3. Steady Boyfriend<br>4. Separated<br>5. Divorced

For peer review only

		99. Other, specify
a6	How many living children do you have?	______________________
a6b	What is the age of your youngest child?	______________________
a6bc	What age is the child (in years or months)	______________________
a4b	How many children currently live with you?	______________________
a7	How many sexual partners have you had in the past 12 months?	______________________
a8	Have you had sex with someone besides your husband without a condom in the past 12 months?	2. Yes<br>0. No<br>88. Don't know/ Not sure<br>89. Refused to answer
a8b	Have you had sex without a condom in the past 12 months?	2. Yes<br>0. No<br>88. Don't know/ Not sure<br>89. Refused to answer
SECTION B: INCOME QUESTIONS		
Intro Note	I will now discuss with you about the valuable items that you or your household possesses. As I will be chatting with you I will also some questions about money you have and activities that you indulge in to find money.	
b1	Please think of the past 12 months, how would you describe your primary occupation?	1. Working formally (employed full time)<br>2. Working informally (ganyu, farming, business)<br>3. Not working

b1b	Think about all the work you have done in the past month. How many days did you normally work this month that gave you pay?	________________
b2	Do you have any savings for the future, such as a bank account, savings group or cash?	2. Yes<br>0. No
	Household Assets	
b3	Does your household have:<br>*The respondent said that his/her household doesn't have any of the household assets. Please probe and ensure that this is correct before you proceed.*	
b3_1	Metal Roof?	2. Yes<br>0. No
b3_2	Electricity?	2. Yes<br>0. No
b3_3	Paraffin lamp with no glass?	2. Yes<br>0. No
b3_4	A paraffin lamp?	2. Yes<br>0. No
b3_5	A radio?	2. Yes<br>0. No
b3_6	A television?	2. Yes<br>0. No
b3_7	A cellular phone?	2. Yes<br>0. No
b3_8	A bed?	2. Yes<br>0. No
b3_9	A sofa set?	2. Yes<br>0. No
b3_10	A table?	2. Yes

For peer review only

		0. No
b3_11	A refrigerator	2. Yes<br>0. No
b3_12	Mattress?	2. Yes<br>0. No
b3_13	Chair(s)?	2. Yes<br>0. No
b3_14	Cattle?	2. Yes<br>0. No
b3_15	Goat?	2. Yes<br>0. No
b3_16	Sheep?	2. Yes<br>0. No
b3_17	Pigs?	2. Yes<br>0. No
b3_18	Donkey?	2. Yes<br>0. No
b3_19	Chickens?	2. Yes<br>0. No
b3_20	Other poultry?	2. Yes<br>0. No
b4	In the past 30 days, have you drank beer?	2. Yes<br>0. No
b4b	How many days in the past 30 days have you drank beer?	______________
b4c	How much money did you spend on beer the last time you went?	MWK:
b4d	In total, approximately how much money did you spend on beer in the past 30 days?	MWK:
Relationship		

For peer review only

Intro Note	Now I'd like to talk to you about your current sexual relationship	
f8	How long have you been/were you in a sexual relationship with your partner?	Days_______________<br>Months_____________<br>Years________________
f9	Do you have children with your partner? How many children?	________________________
f10	How often do you currently talk to your partner?	1. Everyday<br>2. A couple times a week<br>3. Once a week<br>4. A couple times a month<br>5. Once a month<br>6. Less than once a month<br>7. Not at all (never)
f10b	In a typical month, who earns more money? You, or your partner?	1. Myself<br>2. This partner<br>3. We earn the same amount<br>88. Don't know
	Decision Making	
Intro Note	Now I would like to talk to you about how you and your partner make decisions.	
f11	Who usually decides how the money you earn will be used?	1. Yourself (Respondent)<br>2. Jointly (This partner and you together)<br>3. Mainly this partner<br>4. Someone else<br>5. Do not earn money<br>88. Refuse to say
f11b	(if above question=4) Who decides?	________________________
f12	Who usually decides how your partner's earnings will be used?	1. Yourself (Respondent)<br>2. Jointly (This partner and you together)<br>3. Mainly this partner<br>4. Someone else<br>5. Do not earn money<br>88. Refuse to say

For peer review only

f12b	(if above question=4) Who decides?	____________________
f13	Who usually makes decisions about health care for yourself?	1. Yourself (Respondent)<br>2. Jointly (This partner and you together)<br>3. Mainly this partner<br>4. Someone else<br>5. Not applicable/ Don't have children<br>88. Refuse to say
f13b	(if above question=4) Who decides?	____________________
f14	Who usually makes decisions about health care for your child with this partner?	1. Yourself (Respondent)<br>2. Jointly (This partner and you together)<br>3. Mainly this partner<br>4. Someone else<br>5. Not applicable/ Don't have children<br>88. Refuse to say
f14b	(if above question=4) Who decides?	____________________
f15	Who usually makes decisions about health care for your partner?	1. Yourself (Respondent)<br>2. Jointly (This partner and you together)<br>3. Mainly this partner<br>4. Someone else<br>5. Not applicable/ Don't have children<br>88. Refuse to say
f15b	(if above question=4) Who decides?	____________________
f16	Who usually makes decisions about making major household purchases?	1. Yourself (Respondent)<br>2. Jointly (This partner and you together)<br>3. Mainly this partner<br>4. Someone else

For peer review only

		5. Not applicable/ Don't have children<br><br>88. Refuse to say
f16b	(if above question=4) Who decides?	
Note	I would like to ask you questions about [probability/chance/likelihood] that certain things will happen. There are ten beans in this cup. I will ask you to pick some of the beans and put them in the plate. The number of beans that you are going to put in the plate will reflect the probability that something will happen. One bean means there is very little chance that something will happen. If you do not put any bean in the plate it means you are certain that there is no likelihood that something will happen.	
note2	If you put additional beans in the plate it means the chance that something will happen will also increase. For example, if you put one or two beans in the plate, it means there is little chance that something will happen. Even though there is little chance but it can happen. If you put ten beans it means there is equal chance of something happening or not. If you put six beans it means the chance that something will happen is slightly greater than not happening. If you put all ten beans, it means you are certain that whatever the case something will really happen. There is no wrong or right answer I just want to know what you think.	
note3	INTERVIEWER: Report for each question the NUMBER OF BEANS put in the PLATE. After each question, replace the beans on the table (unless otherwise noted).	
	Practice	
pr1	Pick the number of beans that reflects how likely you think it is that:	___________________

For peer review only

pr1b	You will go to the market at least once within the next 2 days.	________________
pr1c	You will go to the market at least once within the next 2 weeks.	________________
Practice		
pr2	INTERVIEWER: Did Respondent add any beans between pr1b and pr1c?	2. Yes<br>0. No
pr3	Remember, as time goes by, you may find more time to go to the market. Therefore, you should have added beans to the plate. Let me ask you again. Now, add beans in the plate so that the number of beans in the plate reflects how likely you think it is that you will go to the market at least once within 2 weeks.<br><br>How likely you think it is that you will go to the market at least once within 2 weeks?	________________
f17	Pick the number of beans that reflects how likely you think:	
f17a	You will still be married/with [partner one year from now.	________________
f17b	Your partner will become sick during the next 12 months	________________
f17c	Your partner will start ART treatment in the next 3 months	________________
f17d	Your partner will disclose your HIV status to your close friends/family in the next 3 months	________________
SELF REPORTED HEALTH AND HAPPINESS		
Intro Note	Now I'd like to talk to you about how healthy and happy you feel.	

For peer review only

e1	I am interested in your general level of well-being or satisfaction with life. How satisfied are you with your life, all things considered?	1. Very satisfied<br>2. Somewhat satisfied<br>3. Neutral<br>4. Somewhat unsatisfied<br>5. Very unsatisfied
e2	Do you think that you are more, equally or less satisfied than other persons your age and sex living in your village?	1. More satisfied<br>2. Equally satisfied<br>3. Less satisfied
e3	In general, would you say your health now is: very good, good, poor or very poor?	1. Very good<br>2. Good<br>3. Poor<br>4. Very poor
e4	How would you compare your health to other people of the same age and sex in your village?	1. More healthy<br>2. Equally healthy<br>3. Less healthy
e5	In the past month, how many days were you too sick to work/go to school/complete household chores?	____________________
Happiness		
e6	How true are the following statements for you in the last month?	
e6_1	I have felt depressed	1. Strongly Agree<br>2. Agree<br>3. Disagree<br>4. Strongly Disagree
e6_2	I have felt life was not worth living	1. Strongly Agree<br>2. Agree<br>3. Disagree<br>4. Strongly Disagree
e6_3	I have felt content.	1. Strongly Agree<br>2. Agree<br>3. Disagree<br>4. Strongly Disagree
e6_4	I have felt lonely	1. Strongly Agree<br>2. Agree<br>3. Disagree<br>4. Strongly Disagree
GENDER EQUITABLE MEN SCALE		

For peer review only

Note	Please tell me if you strongly agree, agree, disagree, or strongly disagree with the following statements:	
j1	Woman's most important role is to take care of her home and cook (take care of home is about housekeeping)	1. Strongly Agree<br>2. Agree<br>3. Unsure<br>4. Disagree<br>5. Strongly Disagree<br><br>88. Refuse to say
j2	Men need sex more than women	1. Strongly Agree<br>2. Agree<br>3. Unsure<br>4. Disagree<br>5. Strongly Disagree<br><br>88. Refuse to say
j3	Men don't talk about sex, they just do it.	1. Strongly Agree<br>2. Agree<br>3. Unsure<br>4. Disagree<br>5. Strongly Disagree<br><br>88. Refuse to say
j4	There are times when a woman deserves to be beaten	1. Strongly Agree<br>2. Agree<br>3. Unsure<br>4. Disagree<br>5. Strongly Disagree<br><br>88. Refuse to say
j5	Changing diapers, giving kids a bath & feeding kids are mother's responsibility	1. Strongly Agree<br>2. Agree<br>3. Unsure<br>4. Disagree<br>5. Strongly Disagree<br><br>88. Refuse to say
j6	It is a woman's responsibility to avoid getting pregnant	1. Strongly Agree<br>2. Agree<br>3. Unsure<br>4. Disagree<br>5. Strongly Disagree<br><br>88. Refuse to say

For peer review only

j7	A man should have the final word about decisions in his home	1. Strongly Agree<br>2. Agree<br>3. Unsure<br>4. Disagree<br>5. Strongly Disagree<br>88. Refuse to say
j8	Men are always ready to have sex	1. Strongly Agree<br>2. Agree<br>3. Unsure<br>4. Disagree<br>5. Strongly Disagree<br>88. Refuse to say
j9	A woman should tolerate violence in order to keep her family together	1. Strongly Agree<br>2. Agree<br>3. Unsure<br>4. Disagree<br>5. Strongly Disagree<br>88. Refuse to say
j10a	I would be outraged if my wife asked me to use a condom.	1. Strongly Agree<br>2. Agree<br>3. Unsure<br>4. Disagree<br>5. Strongly Disagree<br>88. Refuse to say
j10b	Men would be outraged if their wife asked them to use a condom	1. Strongly Agree<br>2. Agree<br>3. Unsure<br>4. Disagree<br>5. Strongly Disagree<br>88. Refuse to say
j11	A man and a woman should decide together what type of contraceptive to use	1. Strongly Agree<br>2. Agree<br>3. Unsure<br>4. Disagree<br>5. Strongly Disagree<br>88. Refuse to say
j12	I would never have a homosexual friend	1. Strongly Agree<br>2. Agree<br>3. Unsure<br>4. Disagree

For peer review only

		5. Strongly Disagree<br>88. Refuse to say
j13a	If someone insults me, I will defend my reputation, with force if I have to.	1. Strongly Agree<br>2. Agree<br>3. Unsure<br>4. Disagree<br>5. Strongly Disagree<br>88. Refuse to say
j13b	If someone insults a man, he should defend his reputation, with force if he has to	1. Strongly Agree<br>2. Agree<br>3. Unsure<br>4. Disagree<br>5. Strongly Disagree<br>88. Refuse to say
j14	To be a man you need to be tough.	1. Strongly Agree<br>2. Agree<br>3. Unsure<br>4. Disagree<br>5. Strongly Disagree<br>88. Refuse to say
j15	Men should be embarrassed if unable to get an erection	1. Strongly Agree<br>2. Agree<br>3. Unsure<br>4. Disagree<br>5. Strongly Disagree<br>88. Refuse to say
j16	If a guy gets a woman pregnant, child is the responsibility of both the man and woman	1. Strongly Agree<br>2. Agree<br>3. Unsure<br>4. Disagree<br>5. Strongly Disagree<br>88. Refuse to say
j17	A man should know what his partner likes during sex	1. Strongly Agree<br>2. Agree<br>3. Unsure<br>4. Disagree<br>5. Strongly Disagree<br>88. Refuse to say

For peer review only

j18	The participation of the father is important in raising children	1. Strongly Agree<br>2. Agree<br>3. Unsure<br>4. Disagree<br>5. Strongly Disagree<br><br>88. Refuse to say
j19	It's important for men to have friends to talk about their problems	1. Strongly Agree<br>2. Agree<br>3. Unsure<br>4. Disagree<br>5. Strongly Disagree<br><br>88. Refuse to say
j20	A couple should decide together if they want to have children.	1. Strongly Agree<br>2. Agree<br>3. Unsure<br>4. Disagree<br>5. Strongly Disagree<br><br>88. Refuse to say
Comment	We have reached the end of the chat. Thank you for your time. Do you have anything else you would like to say?	
End Note	Thank the participant for their time and give them transport reimbursement, if they did not come for an ART appointment	
Comments	Enumerator comments	
End of the survey!		

For peer review only

BASELINE SURVEY
Identifying efficient linkages strategies for HIVST (IDEaL)
Female (Chichewa)

**Question Name**	**Label**	**Responses**
INTRODUCTION SECTION		
interviewer	Full Name of Interviewer	
Interview date	Interview date	
Time start	Time survey started	
District	District	
Facility	Facility	
ID	ID	
SECTION A: DEMOGRAPHICS		
Intro Note	Zikomo povomera kutenga nawo mbali. Pa nthawi ino ndikufunsani ma funso ochepa okhudza za inu komanso kuti ndinu ndani. Chonde khalani omasuka kuyankha moona. Palibe yankho lokhoza komanso lolakwa.	
a7	Ndinu mtundu wanji wa munthu?	1. Lomwe<br>2. Sena<br>3. Chewa<br>4. Mang'anja/Nyanja<br>5. Ngoni<br>6. Tumbuka<br>7. Tonga<br>8. Yao<br>99. Other, specify

For peer review only - http://bmjopen.bmj.com/site/about/guidelines.xhtml

a3	Kodi maphunziro anu mudapita nawo patali bwanji?	1. Pulayimale<br>2. Sekondale<br>3. Koleji
a3b	Ndi kalasi liti munamaliza ya maphunziro anu apamwamba?	______________________
a4	Chonde ganizani za masabata khumi ndi awiri apitawa, mungafotokoze bwanji za ntchito yomwe mumagwira?	1. Ntchito yokhazikika<br>2. Ganyu/bisinesi<br>3. Sindikugwira ntchito
a5	Kodi pakadali pano muli pa banja?	1. Pa banja<br>2. Kukhala limodzi ngati banja<br>3. Chibwezi Chokhazikika<br>4. Tinasiyana<br>5. Banja linatha<br>99. Zina
a6	Muli ndi ana angati amoyo?	________________________
a6b	Mwana wanu wang'ono ali ndi zaka zingati?	_________________________
a6bc	Mwana wanu wang'ono ali ndi zaka zingati?	_________________________
a4b	Pakadali pano ana omwe mumakhala nawo ndi angati?	_________________________
a7	Pa miyezi khumi ndi iwiri yapitayi mwakhala ndi abwenzi ogonana nawo angati?	_________________________
a8	Mwakhalapo ndi bwenzi logonana kupatula akazi anu?amuna anu osagwiritsa ntchito kondomu mu miyezi khumi ndi awiri yapitayi?	3. Eya<br>4. Ayi<br>88. Sindikudziwa<br>89. Akana kuyankha
a8b	Mwagonanapo ndi munthu osagwiritsa ntchito kondomu mu miyezi khumi ndi awiri yapitayi?	3. Eya<br>4. Ayi<br>88. Sindikudziwa<br>89. Akana kuyankha

For peer review only

SECTION B: INCOME QUESTIONS		
Intro Note	Pa nthawi ino ndikufunsani za zipangizo zomwe inu komanso apabanja panu alinazo. Mkati mwakucheza kwathu ndikufunsaninso za ndalama zomwe mulinazo komanso komanso zomwe mumachita kuti mupeze ndalama.	
b1	Chonde ganizirani za miyezi Khumi ndi iwiri yapitayi, mungafotokoze bwanji za ntchito yomwe mumagwira?	1. Ntchito yokhazikika<br>2. Ganyu/bisinesi<br>3. Sindikugwira ntchito
b1b	Ganizani za ntchito zonse mwagwira mwezi watha. Mwagwira masiku angati olipidwa?	____________________
b2	Muli ndi ndalama zilizonse zomwe mukusungira za mtsogolo monga, ku banki, gulu losugira ndalama kapena ndalama zosunga kunyumba?	2. Eya<br>3. Ayi
Household Assets		
b3	Does your household have:<br>*The respondent said that his/her household doesn't have any of the household assets. Please probe and ensure that this is correct before you proceed.*	
b3_1	Denga la malata?	1. Eya<br>2. Ayi
b3_2	Magetsi ?	3. Eya<br>4. Ayi
b3_3	Koloboyi?	3. Eya<br>4. Ayi
b3_4	Nyali?	3. Eya<br>4. Ayi
b3_5	Wailesi?	3. Eya<br>4. Ayi
b3_6	Kanema?	3. Eya<br>4. Ayi
b3_7	Lamya ya M'manja?	3. Eya<br>4. Ayi
b3_8	Kama?	3. Eya

For peer review only

		4. Ayi
b3_9	Sofa?	3. Eya<br>4. Ayi
b3_10	Tebulo?	3. Eya<br>4. Ayi
b3_11	FIliji?	3. Eya<br>4. Ayi
b3_12	Matilesi?	3. Eya<br>4. Ayi
b3_13	Mipando	3. Eya<br>4. Ayi
b3_14	Ng'ombe?	3. Eya<br>4. Ayi
b3_15	Mbuzi?	3. Eya<br>4. Ayi
b3_16	Nkhosa?	3. Eya<br>4. Ayi
b3_17	Nkhumba?	3. Eya<br>4. Ayi
b3_18	Bulu?	3. Eya<br>4. Ayi
b3_19	Nkhuku?	3. Eya<br>4. Ayi
b3_20	Zoweta zina	3. Eya<br>4. Ayi
b4	Mu masiku makumi atatu apitawa mwamako mowa?	3. Eya<br>4. Ayi
b4b	Pa masiku makumi atatu apitawa mwamwa mowa masiku angati?	______________________
b4c	Mwataya ndalama zingati masiku omaliza omwe munapita ku mowa?	MWK:
b4d	Zonse pamodzi, mwataya ndalama zingati pa mowa mu masiku makumi atatu apitawa?	MWK:
Relationship		
Intro Note	Panthawi ino ndikufunsani za abwezi ogonana nawo?	
f8	Mwakhala pa ubwenzi ogonana ndi bwenzi lanu kwa nthawi yayitali bwanji?	Masiku ___________<br>Miyezi _____________

For peer review only

		Zaka________________
f9	Muli ndi ana ndi bwenzi lanu logonana nalo? Ana angati?	______________________
f10	Mumayankhulana mowirikiza bwanji ndi bwenzi lanu pakadali pano?	8. Tsiku ndi tsiku<br>9. Masiku angapo pasabata<br>10. Kamodzi pa sabata<br>11. Kangapo pa mwezi<br>12. Kamodzi pa mwezi<br>13. Kosakwana mwezi<br>14. Sitiyankhulani
f10b	Pa mwezi amalandila ndalama zambiri ndi ndani? Inu kapena bwenzi lanu?	5. Ine<br>6. Bwenzi langa<br>7. Timalandira ndalama zofanana<br>8. Sindikudziwa
	Decision Making	
Intro Note	Pa nthawi ino ndikufunsani za momwe mumapangira maganizo ndi bwenzi lanu	
f11	Nthawi zambiri ndi ndani amene amapanga chiganizo cha momwe ndalama mumapeze zigwiritsidwe ntchito?	6. Ine<br>7. Timagwirizana<br>8. Nthawi zambiri bwenzi langa<br>9. Munthu wina<br>10. Sindipeza ndalama<br>89. Sindikufuna kuyankha
f11b	(Ngati yankho ndi 4) Amapanga chiganizo ndi ndani?	______________________
f12	Amapanga chiganizo cha momwe ndalama za bwenzi lanu zigwiritsidwe ntchito ndi ndani?	6. Ine<br>7. Timagwirizana<br>8. Nthawi zambiri bwenzi langa<br>9. Munthu wina<br>10. Sindipeza ndalama<br>89. Sindikufuna kuyankha
f12b	(Ngati yankho ndi 4)	______________________

For peer review only

f13	Nthawi zambiri amapanga chiganizo chokhudza thandizo la zaumoyo wanu ndi ndani?	6. Ine<br>7. Timagwirizana<br>8. Nthawi zambiri bwenzi langa<br>9. Munthu wina<br>10. Sindipeza ndalama<br>89. Sindikufuna kuyankha
f13b	(Ngati yankho ndi 4) Amapanga chiganizo ndi ndani?	______________________
f14	Nthawi zambiri amapanga chiganizo cha thandizo la zaumoyo la mwana yemwe muli naye ndi bwenzi lanu ndi ndani?	6. Ine<br>7. Mogwirizana<br>8. Nthawi zambiri bwenzi langa<br>9. Munthu wina<br>10. Ndilibe mwana<br>88. Sindikufuna kuyankha
f14b	(if above question=4) Who decides?	______________________
f15	Nthawi zambiri amapanga chiganizo chokhudza thandizo la zaumoyo la bwenzi lanu ndi ndani?	1. Ine<br>2. Mogwirizana<br>3. Nthawi zambiri bwenzi langa<br>4. Munthu wina<br>5. Ndilibe ana<br>88.Sindikufuna kuyankha
f15b	(Ngati yankho ndi 4) Amapanga chiganizo ndi ndani?	______________________
f16	Kodi ndi ndani amene amapanga ziganizo zogula katundu mkulumkulu wapa khomo panu?	1. Ine<br>2. Mogwirizana<br>3. Nthawi zambiri bwenzi langa<br>4. Munthu wina<br>5. Ndilibe ana<br>88.Sindikufuna kuyankha
f16b	(Ngati yankho ndi 4) Amapanga	

For peer review only

	chiganizo ndani?	
Note	Ndikufuna ndikufunseni zokhudzana za (Kuthekela/mwayi) kuti zinthu zina zichitike. Muli nyemba mu kapu. Ndikufunsani kuti musankhe zina mwa nyemba ndipo muyike mu mbale. Mulingo wa nyemba omwe muyike mu mbale udzafanizira kuti chinachake chichitika. Nyemba imodzi ikusonyeza kuti mwayi ndiochepa kuti chinachake chichitika. Ngati simuika nyemba mm'bale ndekuti mukutsimikiza kuti palibe mwayi oti chinachake chichitika	
note2	Ngati muyike nyemba zowonjezera mu mbale, zikutanthauza kuti mwayi oti chinachake chichitika uchuluka, mwachitsanzo ngati muyika nyemba imodzi kapena ziwiri mwayi oti chinachake chichitika. Ngakhale pali mwayi ochepa koma chinachake chichitika. Ngati muyike nyemba nkhumi zikutanthauza kuti pali mwayi ofanana oti chinachake chichika kapena ayi. Ngati muyike nyemba zisanu ndi imodzi zikutanthauza kuti mwayi woti chinachake chichitika uli ochulukilapo kuposa mwayi oti chinachake sichichitika. Ngati muyike nyemba zonse khumi ndekuti muli ndi chikhulupiliro chonse kuti chinachake chichitikadi pavute pasavute. Palibe yankho lokhonza kapena lolakwa ndikungofuna kudziwa zomwe mukuganiza.	
note3	Ofunsa: Pelekani yankho lanu pa funso lililonse nambala ya nyemba zomwe lili m'bale. Pakutha pa funso lililonse, bwezeletsani nyemba pa tebulo.	
Practice		

For peer review only

pr1	Sankhani mulingo wa nyemba omwe ukhale ndi kuthekela kumene mukuganiza kuti:	________________
pr1b	Mupita ku msika mosachepera kamodzi m'masiku awiri akudzawa	________________
pr1c	Mupita ku msika mosachepera kamodzi m'masabata awiri akudzawa.	________________
Practice		
pr2	Ofunsa: kodi oyankha anawonjezera nyamba pakati pa pr1b ndi pr1c	3. Eya<br>1. Ayi
pr3	Kumbukurani kuti pamene nthawi ikupita muzipeza mpata wambiri opita kunsika. Choncho, munayenela kuti mwaika nyemba zambiri m'bale<br><br>Kodi mukuganiza kuti kumsika mupita mosachepela kamodzi bwanji mu nyengo ya ma sabata awiriwa?	________________
f17	Sankhani mlingo wa nyemba umene ufanizile kaganidwe kanu:	

f17a	Mudzakhalabe pabanja ndi bwenzi lanu m'chaka chimodzi chikubwerachi	________________
f17b	Bwenzi lanu lidzapezeka ndi matenda a HIV m'miyezi khumi ndi iwiri ikubwerayi	________________
f17c	Bwenzi lanu lidzayamba kumwa ma ARV m'miyezi itatu ikubwerayi	________________
f17d	Bwenzi lanu lidzaulula kuti inuyo muli ndi kachilombo ka HIV kwa anzanu apamtima/abale anu m'miyezi itatu ikubwerayi	________________
SELF REPORTED HEALTH AND HAPPINESS		
Intro Note	Pa nthawi ino ndikufuna ndikambe nanu zokhudza umoyo ndi chisangalalo chanu.	
e1	Ndili ndi chidwi ndi mukudziwa za umoyo ndi kukhutitsidwa kwanu. Kodi muli okhutitsidwa bwanji ndi moyo wanu, pakutengela zonse.	6. Okhutitsidwa kwamburi<br>7. Okhutitsidwa pang'ono<br>8. Pakatikati<br>9. Okhutitsidwa pang'ono<br>10. Osakhutitsidwa olo pang'ono
e2	Kodi mukuona ngati muli okhutitsidwa mofanana kapena osakhutitsidwa pang'ono mosaposela anthu ena a muna kapena akazi a msinkhu wanu ndi opezeka m'mudzi mwanu?	4. Okhutitsidwa kwambiri<br>5. Okhutiotsidwa<br>6. Okhutitsidwa pang'ono
e3	Kutengela zonse, Kodi munganene kuti umoyo wanu tsopano uli bwino kwambiri, ulibwino, sulibwino, suli bwino olo pang'ono	5. Bwino kwambiri<br>6. Bwino<br>7. Silibwino<br>8. SIlibwino olo pang'ono
e4	Kodi mungazifananizile bwanji za thanzi lanu ndi la anthu ena a msikhu ofanana ndi wanu, amuna kapena	4. A thanzi kwambiri<br>5. A thanzi<br>6. Nthanzi lochepekela

For peer review only

	akazi a m'mudzi mwanu.	
e5	Kodi mwezi wathawu, ndi masiku angati omwe munadwara kwambiri ofika kukulepheretsani kugwira ncthito/kupita ku sukulu/kugwira ntchito za pa khomo	____________________
Happiness		
e6	Kodi ziganizo izi ndi zowona bwanji kwa inu?	
e6_1	Ndinali ndi nkhawa	5. Ndikugwirizana nazo kwambiri<br>6. Ndikugwirizana nazo<br>7. Sindikugwirizana nazo<br>8. Sindikugwirizana nazo kwambiri
e6_2	Ndimanva ngati moyo wafika	5. Ndikugwirizana nazo kwambiri<br>6. Ndikugwirizana nazo<br>7. Sindikugwirizana nazo<br>8. Sindikugwirizana nazo kwambiri
e6_3	Ndimanva kukhutitsidwa	5. Ndikugwirizana nazo kwambiri<br>6. Ndikugwirizana nazo<br>7. Sindikugwirizana nazo<br>8. Sindikugwirizana nazo kwambiri
e6_4	Ndimanva kusalidwa	5. Ndikugwirizana nazo kwambiri<br>6. Ndikugwirizana nazo<br>7. Sindikugwirizana nazo<br>8. Sindikugwirizana nazo kwambiri
GENDER EQUITABLE MEN SCALE		
Note	Chonde ndiuzeni ngati mukugwirizana nazo kwabiri, mukugwirizana nazo, simukugwirizana nazo, simukugwirizana nazo olo pang'ono ziganizo izi:	

For peer review only

j1	Udindo ofunikila wa mzimayi ndi kusamala khomo lake ndi kuphika.	6. Ndikugwirizana nazo kwambiri<br>7. Ndikugwirizana nazo<br>8. Sindikukhulupilira<br>9. Sindikugwirizana nazo<br>10. Sindikugwirizana nazo olo pang'ono<br>89. Sindikufuna kuyankha
j2	Abambo amafuna kugonana kuposa amayi	6. Ndikugwirizana nazo kwambiri<br>7. Ndikugwirizana nazo<br>8. Sindikukhulupilira<br>9. Sindikugwirizana nazo<br>10. Sindikugwirizana nazo olo pang'ono<br>88. Sindikufuna kuyankha
j3	Azibambo sakonda kukambirana za kugonana amangochita	6. Ndikugwirizana nazo kwambiri<br>7. Ndikugwirizana nazo<br>8. Sindikukhulupilira<br>9. Sindikugwirizana nazo<br>10. Sindikugwirizana nazo olo pang'ono<br>88. Sindikufuna kuyankha
j4	Nthawi zina mzimayi amayenela kumenyedwa.	6. Ndikugwirizana nazo kwambiri<br>7. Ndikugwirizana nazo<br>8. Sindikukhulupilira<br>9. Sindikugwirizana nazo<br>10. Sindikugwirizana nazo olo pang'ono<br>88. Sindikufuna kuyankha
j5	Kusinta matewela, kusambitsa mwana, kudyetsa mwana ndi udindo wa mzimayi	6. Ndikugwirizana nazo kwambiri<br>7. Ndikugwirizana nazo<br>8. Sindikukhulupilira<br>9. Sindikugwirizana nazo<br>10. Sindikugwirizana nazo olo pang'ono<br>88. Sindikufuna kuyankha
j6	Ndi udindi wa mzimayi kupewa	6. Ndikugwirizana nazo kwambiri

For peer review only

	kutenga mimba.	7. Ndikugwirizana nazo<br>8. Sindikukhulupilira<br>9. Sindikugwirizana nazo<br>10. Sindikugwirizana nazo olo pang'ono<br>88. Sindikufuna kuyankha
j7	Mzibambo ayenela kukhala ndi chiganizo chomaliza cha mnyumba mwake.	6. Ndikugwirizana nazo kwambiri<br>7. Ndikugwirizana nazo<br>8. Sindikukhulupilira<br>9. Sindikugwirizana nazo<br>10. Sindikugwirizana nazo olo pang'ono<br>88. Sindikufuna kuyankha
j8	Azibambo amakhala okonzeka ku gonana	6. Ndikugwirizana nazo kwambiri<br>7. Ndikugwirizana nazo<br>8. Sindikukhulupilira<br>9. Sindikugwirizana nazo<br>10. Sindikugwirizana nazo olo pang'ono<br>Sindikufuna kuyankha
j9	Mzimayi akuyenela kulekelela nkhaza kuti asunge banja lake.	6. Ndikugwirizana nazo kwambiri<br>7. Ndikugwirizana nazo<br>8. Sindikukhulupilira<br>9. Sindikugwirizana nazo<br>10. Sindikugwirizana nazo olo pang'ono<br>Sindikufuna kuyankha
j10a	Ndikhoza kukwiya akaza anga atati andiuze kuti ndigwiritse ntchito kondumu.	6. Ndikugwirizana nazo kwambiri<br>7. Ndikugwirizana nazo<br>8. Sindikukhulupilira<br>9. Sindikugwirizana nazo<br>10. Sindikugwirizana nazo olo pang'ono<br>88. Sindikufuna kuyankha
j10b	Azibambo akhoza kukwiya akazi awo atawauza kuti agwiritse ntchito	6. Ndikugwirizana nazo kwambiri

For peer review only

	kondomu	7. Ndikugwirizana nazo<br>8. Sindikukhulupilira<br>9. Sindikugwirizana nazo<br>10. Sindikugwirizana nazo olo pang'ono<br>88. Sindikufuna kuyankha
j11	Mzimayi komanso nzibambo agwirizane limodzi kuti agwiritse ntchito njira yanji yakulela	6. Ndikugwirizana nazo kwambiri<br>7. Ndikugwirizana nazo<br>8. Sindikukhulupilira<br>9. Sindikugwirizana nazo<br>10. Sindikugwirizana nazo olo pang'ono<br>88. Sindikufuna kuyankha
j12	Sindingakhale ndi nzanga wopanga zamathanyula	6. Ndikugwirizana nazo kwambiri<br>7. Ndikugwirizana nazo<br>8. Sindikukhulupilira<br>9. Sindikugwirizana nazo<br>10. Sindikugwirizana nazo olo pang'ono<br>88. Sindikufuna kuyankha
j13a	Ngati munthu angandinyoze, ndiziteteza pogwiritsa ntchito mphanvu, ngati ndikufunika kutero.	6. Ndikugwirizana nazo kwambiri<br>7. Ndikugwirizana nazo<br>8. Sindikukhulupilira<br>9. Sindikugwirizana nazo<br>10. Sindikugwirizana nazo olo pang'ono<br>88. Sindikufuna kuyankha
j13b	Ngati munthu anganyoze mzibambo, aziteteze pogwiritsa ntchito mphanvu ngati akufunika kutelo.	6. Ndikugwirizana nazo kwambiri<br>7. Ndikugwirizana nazo<br>8. Sindikukhulupilira<br>9. Sindikugwirizana nazo<br>10. Sindikugwirizana nazo olo pang'ono<br>Sindikufuna kuyankha
j14	Kuti ukhale mzibambo ukufunika kukhala ovuta.	6. Ndikugwirizana nazo kwambiri<br>7. Ndikugwirizana nazo<br>8. Sindikukhulupilira

For peer review only

		9. Sindikugwirizana nazo<br>10. Sindikugwirizana nazo olo pang'ono<br>Sindikufuna kuyankha<br>Sindikugwirizana nazo kwambiri<br>88. Refuse to say<br>Sindikufuna kuyankha
j15	Azibambo akuyenela kunva manyazi ngati akukanika kutota	6. Ndikugwirizana nazo kwambiri<br>7. Ndikugwirizana nazo<br>8. Sindikukhulupilira<br>9. Sindikugwirizana nazo<br>10. Sindikugwirizana nazo olo pang'ono<br>88. Sindikufuna kuyankha
j16	Ngati mzibambo wapeleka mimba kwa mzimayi, mwanayo ndi udindo wa anthu onse a wiri.	6. Ndikugwirizana nazo kwambiri<br>7. Ndikugwirizana nazo<br>8. Sindikukhulupilira<br>9. Sindikugwirizana nazo<br>10. Sindikugwirizana nazo olo pang'ono<br>88. Sindikufuna kuyankha
j17	Nzibambo akuyenela kudziwa zomwe bwenzi lake limakonda pogonana	6. Ndikugwirizana nazo kwambiri<br>7. Ndikugwirizana nazo<br>8. Sindikukhulupilira<br>9. Sindikugwirizana nazo<br>10. Sindikugwirizana nazo olo pang'ono<br>88. Sindikufuna kuyankha
j18	Kutenga nawo mbali kwa a bambo ndi kofunika polela mwana	6. Ndikugwirizana nazo kwambiri<br>7. Ndikugwirizana nazo<br>8. Sindikukhulupilira<br>9. Sindikugwirizana nazo<br>10. Sindikugwirizana nazo olo pang'ono<br>88. Sindikufuna kuyankha

For peer review only

j19	Ndi zofunika kuti abambo azikhala ndi anzawo okambilana nawo mavuto awo.	6. Ndikugwirizana nazo kwambiri<br>7. Ndikugwirizana nazo<br>8. Sindikukhulupilira<br>9. Sindikugwirizana nazo<br>10. Sindikugwirizana nazo olo pang'ono<br>88. Sindikufuna kuyankha
j20	Banja lizigwirizana limodzi ngati likufuna kukhala ndi mwana	6. Ndikugwirizana nazo kwambiri<br>7. Ndikugwirizana nazo<br>8. Sindikukhulupilira<br>9. Sindikugwirizana nazo<br>10. Sindikugwirizana nazo olo pang'ono<br>88. Sindikufuna kuyankha
Comment	Tafika pamapeto a kucheza kwathu. Zikomo chifukwa cha nthawi yanu. Pali chili chonse mukufuna kuwonjezera?	
End Note	Thank the participant for their time and give them transport reimbursement, if they did not come for an ART appointment	
Comments	Enumerator comments	
End of the survey!		

For peer review only

14.7. APPENDIX G: Follow-up Survey - Male

FOLLOW-UP SURVEY
Identifying efficient linkages strategies for HIVST (IDEaL)
Male

Complete this form for men who enrolled in the study 4-months ago

Date of Interview: ____________________
Site Code: ____________________________

Full Name of Interviewer: ______________________________

Participant Study ID#: ___________

#	Question	Response
c1a	Please think about your primary partner in the last 4 months.<br><br>Has your relationship changed in the last 4 months? How?	☐ Nothing changed (1)<br>☐ Married (2)<br>☐ Steady Girlfriend (3)<br>☐ Moved out of the house (4)<br>☐ Became an infrequent partner (5)<br>☐ Separated (6)<br>☐ Divorced (7)<br>☐ Other (8)
c4a	Have you disclosed your HIV status to this partner?	☐ Yes (1)<br>☐ No (0)
c5a	Have you had any new children with this partner since we last spoke?	☐ Yes (1)<br>☐ No (0)
c18a	Have you started ART in the past 4months?	☐ Yes (1)<br>☐ No (0)<br>☐ No, but I plan to link

For peer review only

c18b	IF YES: When did you start ART?	______________<br>Day/Month/Year
	**Unintended Consequences**	
c19a	Were unwantedly pressured to initiate ART?	☐ Yes (1)<br>☐ No (0)
c20a	After enrolling in the study, did anyone find out your HIV status against your will (unwanted disclosure)?	☐ Yes (1)<br>☐ No (0)
C20b	After enrolling in the study, did anyone find out your partners' HIV status against her will (unwanted disclosure)?	☐ Yes (1)<br>☐ No (0)
c21a	After enrolling in the study, did your partner….<br><br>Threaten to hurt or harm you or someone you cared about.	☐ Yes (1)<br>☐ No (0)<br>☐ Refused to respond (99)
c22a	Insulted you or made you feel bad about yourself.	☐ Yes (1)<br>☐ No (0)<br>☐ Refused to respond (99)
c23a	Hit, slapped, kicked or did anything else meant to physically hurt you.	☐ Yes (1)<br>☐ No (0)<br>☐ Refused to respond (99)
c21a	After enrolling in the study, did you ever do the following to your partner….<br><br>Threaten to hurt or harm her or someone she cared about.	☐ Yes (1)<br>☐ No (0)<br>☐ Refused to respond (99)

For peer review only

c22a	Insulted her or made her feel bad about herself.	☐ Yes (1)<br>☐ No (0)<br>☐ Refused to respond (99)
c23a	Hit, slapped, kicked or did anything else meant to physically hurt her.	☐ Yes (1)<br>☐ No (0)<br>☐ Refused to respond (99)
c24	Slept with another woman	☐ Yes (1)<br>☐ No (0)<br>☐ Refused to respond (99)
c25a	*Now, I am going to ask you a series of questions about who makes within this relationship. Please think about the last 4 months*<br><br>Who usually decides how the money you earn will be used?	☐ Yourself (respondent)<br>☐ Jointly (This partner and you together)<br>☐ Mainly this partner<br>☐ Someone else<br>☐ Do not earn money<br>☐ Refuse to say
c26a	Who usually decides how your partner's earnings will be used?	☐ Yourself (respondent)<br>☐ Jointly (This partner and you together)<br>☐ Mainly this partner<br>☐ Someone else<br>☐ Refuse to say

For peer review only

c27a	Who usually makes decisions about health care for yourself?	☐ Yourself (respondent)<br>☐ Jointly (This partner and you together)<br>☐ Mainly this partner<br>☐ Someone else<br>☐ Refuse to say

	**D. Additional Questions**	
#	**Question**	**Response**
d1	Would you recommend the ART intervention you were part of to other male friends or family?	☐ Yes (1)<br>☐ No (0)
d2	Are you happy that you participated in the ART intervention?	☐ Yes (1)<br>☐ No (0)

Thank the participant for their time and end the survey

For peer review only

BASELINE SURVEY
Identifying efficient linkages strategies for HIVST (IDEaL)
Male (Chichewa)

Complete this form for men who enrolled in the study 4-months ago

Date of Interview: ____________________
Site Code: _________________________
Full Name of Interviewer: ____________________________
Participant Study ID#: ___________

#	Question	Response
c1a	Taganizani za bwenzi lanu logonana nalo lomwe lakhala lodalilika kwa miyesi folo yapitayi.<br><br>Kodi ubwenzi wanu wasintha munjira ina iliyonse mu miyezi folo yapitayi?	☐ Palibe chasintha (1)<br>☐ Married (2)<br>☐ Chibwenzi chokhazikika (3)<br>☐ Chibwenzi Chinachoka pakhomo (4)<br>☐ Chibwenzi cha apo ndi apo (5)<br>☐ Separated (6)<br>☐ Banja linatha (7)<br>☐ Other (8)
c4a	Kodi munawauza abwenzi anuwa za mmene mulili mnthupi mwanu ku mbali ya HIV?	☐ Eya(1)<br>☐ Ayi(0)
c5a	Kodi mwakhala ndi ana ndi abwenzi anuwa kuchokera ulendo watha tinayankhulana?	☐ Eya(1)<br>☐ Ayi(0)
c18a	Kodi munayamba kumwa mankhwala a ma ARV mu miyezi folo yapitayi?	☐ Eya(1)<br>☐ Ayi(2)<br>☐Ayi, ndikulingalira zoyamba (3)

For peer review only

c18b	Ngati eya: Munayamba liti mankwala a ma ARV?	________ Tsiku/Mwezi/Chaka
**Unintended Consequences**		
c19a	Munayamba kumwa mankhwala a ma ARV mokakamizidwa?	☐ Eya(1) ☐ Ayi(0)
c20a	Chilowereni mu study, alipo omwe anadziwa za m'mene mulili mnthupi mwanu kumbari ya HIV inu musakufuna? (Kuwulula za HIV mosafuna)	☐ Eya(1) ☐ Ayi(0)
C20b	Chilowereni mu study, alipo omwe anadziwa za m'mene mulili mnthupi mwa bwenzi lanu kumbari ya HIV eni asakufuna? (Kuwulula za HIV mosafuna)	☐ Eya(1) ☐ Ayi(0)
c21a	Chilowereni mu study, kodi bwenzi lanu….. Linaospyeza kuvulaza inu kapena wina aliyense amane mumamukonda?	☐ Eya(1) ☐ Ayi(0) ☐ Refused to respond (99)
c22a	Linakunyozani kapena kukunyogodolani	☐ Eya(1) ☐ Ayi(0) ☐ Refused to respond (99)
c23a	Anakumenyani ndikukupwetekani.	☐ Eya(1) ☐ Ayi(0) ☐ Refused to respond (99)
c21a	Chilowereni mu study kodi munayamba mwapangapo zotsatirazi kwa bwenzi anu…. Kuosyeza kuti muvulaza bwezi lanu kapena wina aliyense yemwe amamukonda.	☐ Eya(1) ☐ Ayi(0) ☐ Refused to respond (99)

c22a	Kunyoza kapenanso kumupangitsa kuti azizikayikila.	☐ Eya(1)<br>☐ Ayi(0)<br>☐ Refused to respond (99)
c23a	Kumumenya ndikumupweteka.	☐ Eya(1)<br>☐ Ayi(0)<br>☐ Refused to respond (99)
c24	Kuchita mchitidwe ogonana ndi mzimayi wina	☐ Eya(1)<br>☐ Ayi(0)<br>☐ Refused to respond (99)
c25a	Tsopano ndikufunsani mafunso okhuzana ndi omwe amalamula pakhomo panu.<br><br>Kodi amalamula za mmene ndalama zomwe mwapeza zitagwilitsidwile ntchito ndi ndani?	☐ Amene akuyankha<br>☐ Mogwirizana<br>☐ Nthawi zambiri bwenzi<br>☐ Munthu wina<br>☐ Sapeza ndalama<br>☐ Refuse to say
c26a	Kodi amalamulila za mmene ndalama za abwenzi anu zingagwilitsidwile ntchito ndi ndani?	☐ Amene akuyankha<br>☐ Mogwirizana<br>☐ Nthawi zambiri bwenzi<br>☐ Munthu wina<br>☐ Refuse to say
c27a	Kodi amene amakhala ndi ulamulilo pa chisamalilo cha moyo wanu ndi ndani?	☐ Amene akuyankha<br>☐ Mogwirizana<br>☐ Nthawi zambiri bwenzi<br>☐ Munthu wina<br>☐ Refuse to say

For peer review only

	Additional Questions	
d1	Kodi mungalimbikitse anzanu ena achizibambo kapena apabanja panu kutenga nawo mbali mu ART intervention munaliyi?	☐ Eya(1)<br>☐ Ayi(0)
d2	Kodi muli okondwa kuti munatenga nawo mbali mu ART intervention?	☐ Eya(1)<br>☐ Ayi(0)

For peer review only

14.8. APPENDIX H: Follow-up Survey – Female

FOLLOW-UP SURVEY
Identifying efficient linkages strategies for HIVST (IDEaL)
Female

Complete this form for women whose:

(1) Partners consented to be in the study 4-months ago

Date of Interview: ____________________
Site Code: _________________________

Full Name of Interviewer: ______________________________

Participant Study ID#: ___________

#	Question	Response
c1a	Please think about your primary partner in the last 4 months.<br><br>Has your relationship changed in the last 4 months? How?	☐ Nothing changed (1)<br>☐ Married (2)<br>☐ Steady Boyfriend (3)<br>☐ Moved out of the house (4)<br>☐ Became an infrequent partner (5)<br>☐ Separated (6)<br>☐ Divorced (7)<br>☐ Other (8)
c4a	Have you disclosed your HIV status to this partner?	☐ Yes (1)<br>☐ No (0)
c5a	Have you had any new children with this partner since we last spoke?	☐ Yes (1)<br>☐ No (0)
c18a	To your knowledge, did your partner start ART?	☐ Yes (1)<br>☐ No, they do not plan to link (2)<br>☐ No, but they plan to link (3)

For peer review only - http://bmjopen.bmj.com/site/about/guidelines.xhtml

		☐ Unsure (88)
**Unintended Consequences**		
c19a	Did you pressure your partner to initiate ART?	☐ Yes (1)<br>☐ No (0)
c20a	After enrolling in the study, did anyone find out your HIV status against your will (unwanted disclosure)?	☐ Yes (1)<br>☐ No (0)
C20b	After enrolling in the study, did anyone find out your partners' HIV status against his will (unwanted disclosure)?	☐ Yes (1)<br>☐ No (0)
c21a	After enrolling in the study, did your partner….<br><br>Threaten to hurt or harm you or someone you cared about.	☐ Yes (1)<br>☐ No (0)<br>☐ Refused to respond (99)
c22a	Insulted you or made you feel bad about yourself.	☐ Yes (1)<br>☐ No (0)<br>☐ Refused to respond (99)
c23a	Hit, slapped, kicked or did anything else meant to physically hurt you.	☐ Yes (1)<br>☐ No (0)<br>☐ Refused to respond (99)
C23b	Forced sexual intercourse and other forms of sexual coercion	☐ Yes (1)<br>☐ No (0)<br>☐ Refused to respond (99)

For peer review only

C23c	Slept with another woman.	☐ Yes (1)<br>☐ No (0)<br>☐ Refused to respond (99)
c24a	Ended the relationship	☐ Yes (1)<br>☐ No (0)<br>☐ Refused to respond (99)
c25a	*Now, I am going to ask you a series of questions about who makes within this relationship*<br><br>Who usually decides how the money you earn will be used?	☐ Yourself (respondent)<br>☐ Jointly (This partner and you together)<br>☐ Mainly this partner<br>☐ Someone else<br>☐ Do not earn money<br>☐ Refuse to say
c26a	Who usually decides how your partner's earnings will be used?	☐ Yourself (respondent)<br>☐ Jointly (This partner and you together)<br>☐ Mainly this partner<br>☐ Someone else<br>☐ Refuse to say
c27a	Who usually makes decisions about health care for yourself?	☐ Yourself (respondent)<br>☐ Jointly (This partner and you together)<br>☐ Mainly this partner<br>☐ Someone else<br>☐ Refuse to say

For peer review only

	Additional Questions	
d1	Would you recommend the ART intervention to other male friends or family?	☐ Yes (1)<br>☐ No (0)
d2	Are you happy your partner was in the ART intervention?	☐ Yes (1)<br>☐ No (0)

Thank the participant for their time and end the survey

For peer review only

FOLLOW-UP SURVEY
Identifying efficient linkages strategies for HIVST (IDEaL)
Female (Chichewa)

Complete this form for women whose:

(1) Partners consented to be in the study 4-months ago

Date of Interview: ______________________
Site Code: ____________________________

Full Name of Interviewer: _________________________________

Participant Study ID#: ____________

#	Question	Response
c1a	Taganizani za bwenzi lanu logonana nalo lomwe lakhala lodalilika kwa miyesi folo yapitayi.<br><br>Kodi ubwenzi wanu wasintha munjira ina iliyonse mu miyezi folo yapitayi?<br><br>Bwenzi lokhazikika	☐ Palibe chasintha (1)<br>☐ Married (2)<br>☐ Bwenzi lokhazikika (3)<br>☐ Chibwenzi Chinachoka pakhomo (4)<br>☐ Chibwenzi cha apo ndi apo (5)<br>☐ Separated (6)<br>☐ Banja linatha (7)<br>☐ Other (8)
c4a	Kodi munawauza abwenzi anuwa za mmene mulili mnthupi mwanu ku mbali ya HIV?	☐ Eya(1)<br>☐ Ayi(0)
c5a	Have you had any new children with this partner since we last spoke?<br>Kodi mwakhala ndi ana ndi abwenzi anuwa kuchokera ulendo watha tinayankhulana?	☐ Eya(1)<br>☐ Ayi(0)
c18a	Mongamukudziwira, kodi bwenzi lanu linayamba kumwa mwankhala a ama ARV?	☐ Eya<br>☐ Ayi, sakulingalira zoyamba kumwa mwankhwala a ma ARV<br>☐ Ayi, koma

		akulingalira zoyamba<br>☐ Unsure (88)
**Unintended Consequences**		
c19a	Kodi munawakakamiza a bwenzi anu kuti ayambe kumwa mankhwala a ama ARV?	☐ Eya(1)<br>☐ Ayi(0)
c20a	Chilowereni mu study, alipo omwe anadziwa za m'mene mulili mnthupi mwanu kumbari ya HIV inu musakufuna? (Kuwulula za HIV mosafuna)	☐ Eya(1)<br>☐ Ayi(0)
C20b	Chilowereni mu study, alipo omwe anadziwa za m'mene mulili mnthupi mwa bwenzi lanu kumbari ya HIV eni asakufuna? (Kuwulula za HIV mosafuna)	☐ Eya(1)<br>☐ Ayi(0)
c21a	Chilowereni mu study, kodi bwenzi lanu…..<br><br>Linaospyeza kuvulaza inu kapena wina aliyense amane mumamukonda?	☐ Eya(1)<br>☐ Ayi(0)<br>☐ Refused to respond (99)
c22a	Anakunyozani kapenanso kukupangitsani kuti muzizikayikila.	☐ Eya(1)<br>☐ Ayi(0)<br>☐ Refused to respond (99)
c23a	Anakumenyani ndikukupwetekani.	☐ Eya(1)<br>☐ Ayi(0)<br>☐ Refused to respond (99)
C23b	Kukukakamizani kugonana ndi zinthu zina?	☐ Eya(1)<br>☐ Ayi(0)<br>☐ Refused to respond (99)
C23c	Kuchita mtchitidwe ogonana ndi mzimayi wina	☐ Eya(1)<br>☐ Ayi(0)

For peer review only

		☐ Refused to respond (99)
c24a	Kuthetsa Chibwenzi	☐ Eya(1)<br>☐ Ayi(0)<br>☐ Refused to respond (99)
c25a	Tsopano ndikufunsani mafunso okhuzana ndi omwe amalamula pakhomo panu.<br><br>Kodi amalamula za mmene ndalama zomwe mwapeza zitagwilitsidwile ntchito ndi ndani?	☐ Amene akuyankha<br>☐ Mogwirizana<br>☐ Nthawi zambiri bwenzi<br>☐ Munthu wina<br>☐ Refuse to say
c26a	Kodi amalamulila za mmene ndalama za abwenzi anu zingagwilitsidwile ntchito ndi ndani?	☐ Amene akuyankha<br>☐ Mogwirizana<br>☐ Nthawi zambiri bwenzi<br>☐ Munthu wina<br>☐ Refuse to say
c27a	Kodi amene amakhala ndi ulamulilo pa chisamalilo cha moyo wanu ndi ndani?	☐ Amene akuyankha<br>☐ Mogwirizana<br>☐ Nthawi zambiri bwenzi<br>☐ Munthu wina<br>☐ Refuse to say

	**Additional Questions**	
d1	Kodi mungalimbikitse anzanu ena achizibambo kapena apabanja panu kutenga nawo mbali mu ART intervention munaliyi?	☐ Eya(1)<br>☐ Ayi(0)
d2	Kodi muli okondwa kuti munatenga nawo mbali mu ART intervention?	☐ Eya(1)<br>☐ Ayi(0)

For peer review only

14.9. APPENDIX I: Data Extraction Tool

DATA EXTRACTION TOOL
Identifying efficient linkages strategies for HIVST (IDEaL)
English only

INSTRUCTIONS:

The Medical Chart Review will be used to link the male study participant with the facilities ART records and to document their facility visits over the 4-months of study participation. Please follow the instructions to prepare for data collection (1) gather all ART registers that were active between DAY MONTH YEAR up to today (2) enter and re-enter the participant ID's who have reached the 4-month follow up period into the tablet (3) once you have re-entered, the tablet will provide you with identifying information about the male study participant. (5) match the participants information with information provided by the ART register to see if the participant initiated ART or not. If a participant did not initiate care (i.e. you cannot find him in the ART register), still enter the initial data points and indicate that the participant did not

For peer review only

Code	Question	Responses
pid	Please enter the Participant ID	
district	District	☐ Chickwawa<br>☐ Nkhotakota<br>☐ Lilongwe<br>☐ Kasungu
site	Facility name	☐ Chickwawa District Hospital<br>☐ St. Montford Mission Hospital<br>☐ Kalemba Community Hospital<br>☐ Kasungu District Hospital<br>☐ Nkhoma Community Hospital<br>☐ Mponela Rural Hospital<br>☐ Deayang Luke Hospital<br>☐ Nkhotakota District Hospital

		☐ Nsanje District Hospital<br>☐ Ngabu Rural Hospital
	We want to know if this participant initiated ART. Please look at the ART register used at the clinic between DATE MONTH YEAR up to today<br><br>*Instructions: Look for the below information in the ART register, matching the below participant with a name in the ART register.*<br><br>*Sometimes it is hard to find an exact match in the ART register. Consider it a match if 3 of the 4 data points match. For example, someone's name may be different, but the age, and village/residence matches. Consider this the same person.*<br><br>*CLIENT NAME:*<br>*AGE:*<br>*Ta:*<br>*Village:*	
found_art	Was the participant found in the ART register?	☐ Yes (1) – proceed to next question<br>☐ No (0) – end survey
art_number	What is the participant assigned ART number?	
art_date	What is the clients ART start date?	___ ___/___ ___/___ ___<br>Day Month Year

For peer review only - http://bmjopen.bmj.com/site/about/guidelines.xhtml

	Use the ART number to find the paper Mastercard OR look up the participant in the Baobab system	
mastercard_found	Did you find the mastercard/baobab record?	☐ Yes (1) – proceed to next question<br>☐ No (0) – end survey
tb	At initiation: TB	☐ Yes (1)<br>☐ No (0)
ks	At initiation: KS	☐ Yes (1)<br>☐ No (0)
pillcount	Number of pills given	
nextapp_date	Date of next appointment	___ ___/___ ___/___ ___<br>Day Month Year
nextconsult_date	Date of next consultation visit	___ ___/___ ___/___ ___<br>Day Month Year

END OF SURVEY

For peer review only

14.10. APPENDIX J: In-Depth Interview Guide – Female

IN-DEPTH INTERVIEW GUIDE
Identifying efficient linkages strategies for HIVST (IDEaL)
Female

BEGIN RECORDING

Original Study ID: ____________________

Repeat Original Study ID:

State if male or female respondent ____________________

Health Facility: ____________________

Date of Interview: ____________________

Full Name of Interviewer: ______________________________

District where respondent Lives: ______________________________

Open- Ended Questions

Note: The in-depth interview will be open-ended and guided by the respondent's answers. This outline reflects a general guide for the in-depth interviews.

The interviews are meant to help us understand barriers and facilitators to ART initiation. We are also interested in their thoughts on new interventions we are developing to help men start ART. The following questions are meant to guide interviewers. Actual questions asked during the interview will vary based on participant responses.

DEMOGRAPHICS:

Demographics on the female participant not collected. Already in the original study. Just make sure the Original Study ID is documented correctly.

1. What type of job/work does your partner do?
    a. If he does not work, why?
    b. Does he do anything else to earn money?
2. What time of day/week is your partner usually busy?
3. Where does he spend most of his time when he is not working?
    a. *Probe: At the bar, watching football, at church, at home…*
4. How long have you been in a relationship with your sexual partner (whom you gave the HIVST kit to)

For peer review only

HIV TESTING:

Now I'd like to talk to you about you and your partner's experience with HIV services

1) To your knowledge, has your partner tested for HIV before you gave him the HIV-self testing kit?
   a) IF YES, did he tell you the result? What was his result?
   b) IF HIV+: At that time, did he start ART? IF NO: Why not?
2) Think about when you gave the HIV self-test kit to your sexual partner (show the kit).
   a) How did your partner feel about his HIV positive test result? How did the result affect him?
   b) How did you feel after your partner received an HIV positive status on the HIV self-test kit? How did the result affect you?
   c) Did your relationship change at all after he received an HIV-positive status? How?
   d) Did he talk to anyone about it?/ who?
   e) Did he disclose to anyone besides you? Why/why not?

ART UPTAKE:

1) After he tested HIV-positive, did he initiate ART? Why/Why not?
   a) IF INITIATED ART: How long did it take him to initiate ART? (several weeks, several months?). Why did you initiate ART so quickly/slowly? /

We know starting ART is difficult.

1) What do you think is the most difficult thing about starting ART for your partner right now?
   a) Do you think this is the same for men and for women? How is it different?
   b) How does taking ART affect one's daily activities? (schedule/routine) In bad ways? In good ways? Is it different for men? How?
   c) How does taking ART affect relationships? In bad ways? In good ways? Is it different for men? How?
   d) Out of all these things you mentioned, what do you think is the biggest concern/problem for your partner?

<u>FOR MEN WHO ARE CONSIDERED LOSS-TO-FOLLOW UP</u>

1. Since we saw you last, we haven't been able to follow up with your partner. From your perspective, why do you think this is?
2. Since your partner was enrolled in our study, what kind of things did you notice or did he mention to you related to HIV health services?
   a. *Probe: phone calls/SMS or speak one-on-one to a health care worker? Was your partner visited by a health care professional within your home or in the community?*
3. Has your partner mentioned any of these interactions to anyone?
   a. *Probe: friends, family, community members*
4. How did your partner react to these things? Did he like/dislike them? What did he do when they happened?
   a. *Probe: phone call, SMS, in-person visit, home-based ART*
5. How did you react to these things? Did you like/dislike them?
   a. *Probe: Was there any reaction amongst your family, friends or the community to these things?*
   b. *If so, how did this affect your partner and/or you?*

SUGGESTIONS FOR ART SERVICES

Thank you for all the information. We would like to develop ART services that meet the needs of individuals in your community. We understand that men may face different challenges than women. I would like to know your opinion about what is needed in order to make ART services easy to access and use.

1) Could you describe the ideal way ART services would be given to your partner? If the clinic could do anything …
   a) When would he want to pick up ART?
      i) Probe: day of week, time of day?
      ii) *Why do you say this?*

   b) Where would he want to pick up ART*?*
      i) Probe: clinic near you, clinic far from you, somewhere in the community (WHERE SPECIFICALLY), at your home?
      ii) *Why do you say this?*

2) Do you think your partner needs more HIV-related information? About the benefits of ART, how to keep his status a secret, or how to disclose his status, about other aspects of his health?
   a) IF YES: How would he want to get this information? In person (one on one, pamphlet, radio, phone call, …)
      i) Probe: IF YES: WHO would he like to talk to about this information? (Provider, expert client, other community member)

3) Does he need support? For example, reminders to go to the health facility, someone to talk to regularly about what he is going through with ART, help disclosing his status, anything else).
   a) Probe: IF YES: How would he want to get this support? In person (one on one, pamphlet, radio, phone call)
   b) Probe: IF YES: WHO would he like to talk to about this support? (Provider, expert client, other community member)

4) Out of time of pick up, location, more information, or more support, what are the most important factors for your partner to use ART services?
   a) *PROBE: Think about other males. What do you think they would say is the most important factor for men to use ART services?*

SPECIFIC SUGGESTIONS ON CURRENT INTERVENTIONS

FOR MEN WHO ARE CONSIDERED LOSS-TO-FOLLOW UP

1. As discussed earlier, your partner received [phone calls, texts, in person counseling, home-based ART].
   a. *Do you feel that this was enough to encourage him to start ART?*
      i. *If NO, what would you have done differently? (frequency, content, location)*
      ii. *If YES, why do you think they were sufficient?*
   b. Are there any other ideas/services we should think about doing beside the ones we just talked about (appointment reminders, in-depth counseling, community/home ART)?/
      i. PROBE: What is it?
      ii. Why do you think this could work?

For peer review only

CONCLUSION

Thank you for your time.

1. Is there anything else you would like to say about men's use of ART?
2. Is there anything else you would like to say about your own use of ART and how we can help make your experience better?

STOP THE RECORDER AND MAKE SURE RECORDING IS SAVED.

THANK YOU FOR YOUR PARTICIPATION IN THIS INTERVIEW. LET ME ENCOURAGE YOU THAT ARVS CAN HELP YOU LIVE LONG AND HEALTHY.

The following general education messages should be conveyed to all male and female participants:

- All people who have been tested HIV positive should start ART as soon as possible for their own health and to prevent passing the virus on to others.
- Serious diseases can occur even in patients with high CD4 count (>500), without any previous symptoms. Immediate ART greatly reduces this risk.
- People that start ART and continue lifelong without interruptions can remain healthy and live as long as people without HIV.
- Even though you may not feel sick, ART is still important to keep you healthy for the rest of your life.
- ART reduces the amount of virus in your body and therefore can reduce the chance that HIV is passed to your sex partners.
- Current ART regimens are easy to take and rarely cause serious side-effects. Some people have side effects in the first few weeks of treatment and these almost always go away. IF there are persistent side effects, an alternative HIV regimen can be given.

NOTE: Be careful not to give any specific medical advice but rather refer respondents back to the clinic to speak to a provider.

IN-DEPTH INTERVIEW GUIDE
Identifying efficient linkages strategies for HIVST (IDEaL)
Female (Chichewa)

BEGIN RECORDING

Original Study ID: ______________________

Repeat Original Study ID:

State if male or female respondent ______________________

Health Facility: ______________________

Date of Interview: ______________________

Full Name of Interviewer: ________________________________

District where respondent Lives: ________________________________

Demographics

1) Kodi bwenzi lanu limagwira ntchito yanji kuti apeze ndalama?
   a) Ngati sagwira ntchito ndi chifukwa chani?
   b) Kodi amapanga zinthu zina zothandizira kupeza ndalama?
2) Kodi ntha nd nthawi iti ya tsiku/sabata lomwe bwenzi lanu limakhala lotanganidwa?
3) Kodi nthawi yawo yambiri amakhala ali kuti ngati sakugwira ntchito?
4) Kodi mwakhala pa ubwenzi kwa nthawi yayitali bwanji ndi bwenzi lanu logonana nalo (lomwe munalipatsa ka chida koziyezela wekha HIV)

HIV-Testing

Pano ndimafuna ndikufunseni mafunso okhudzana ndi zomwe mwakumanapo nazo zokhudza thandizo la HIV.

1. Momwe mukudziwira kodi bwenzi lanu linayamba layezetsapo HIV musanawapatse kachida koziyeza wekha HIV?
   a. Ngati eya, anakuwuzani zotsatira? Zotsatira zawo zinali zotani?
   b. Ngati anapezeka ndi HIV nthawi imeneyo, anayamba kumwa ma ARV? Ngati ayi chifukwa chani?
2. Ganizirani nthawi yomwe mudapereka kachipangizo kodziyezera wekha HIV (awonetseni) kwa bwenzi lanu.
   a. Kodi bwenzi lanu linanva bwanji atadziwa za zotsatira zawo za HIV? Zotsatirazo anazilandira bwanji?
   b. Kodi munamva bwanji bwenzi lanu litalandira zotsatira zoti lili ndi kachilombo ka HIV

pa kachipangizoka? Izi zinakukhudzani bwanji?

c. Kodi ubwenzi wanu udasintha atalandira zotsatira zoti ali ndi kachilombo? Motani?
d. Kodi analankhulapo ndi wina aliyense? Mosatchula dzina, ndani?
e. Kodi anawuzapo wina aliyense za mthupi mwake kupatula inu? Chifukwa chani?

ART Uptake

1) Atadziyeza ndi kupeza kuti ali ndi kachilombo, kodi adakayamba kumwa mankhwala? Chifukwa?
   a) Kodi zinatenga nthawi yaitali bwanji asanayambe kumwa mankhwala a ma ARV? (masabata angapo, miyezi ingapo?) Kodi anayamba kumwa mankhwala mnsanga/mochedwa chifukwa chani?
2) Tikudziwa kuti kuyamba kumwa mankhwala a ARV kumavutirapo. Kodi mukuona kuti chovuta chachikulu pa kuyamba kumwa mankhwala ndi chani?
   a) Kodi mukuganiza kuti izi ndi zofanana kwa amuna ndi akazi? Nzosiyana chifukwa chani?
   b) Kodi kumwa mankhwala a ma ARV kumakhudza bwanji kagwiridwe ka ntchito ka munthu tsiku ndi tsiku (ntchito zomwe amagwiragwira) Mu njira yabwino kapena yobwera mmbuyo? Ndi zosiyana kwa amuna? Motani?
   c) Kodi kumwa mankhwala a ma ARV kumakhudza bwanji mwaubwenzi? Mu njira yabwino kapena yobwera mmbuyo? Ndi zosiyana kwa amuna? Motani?
   d) Pa zinthu zonse zomwe tambambikalana, mukuona kuti vuto lalikuru kwa bwenzi lanu ndi chani?

<u>*FOR MEN WHO ARE CONSIDERED LOSS-TO-FOLLOW UP*</u>

1) Chiwonelaneni ulendo watha, talephela kuonananso ndi bwenzi lanu. Mumaganizo anu mukuona kuti zili motere chifukwa chani?
2) Chilowereni cha bwenzi lanu mu study, mwaonako zinthu zotani kapena zomwe atchulako zokhudzana ndi thandizo la HIV?
   a) Funsitsani: Lamya ya m'manja/uthenga wa pa lamya kapena kuyankhula ndi wazaumoyo? Kodi bwenzi lanu linayendeleledwapo ndi wazaumoyo mu dela lanu kapena pafupi ndi nyumba yanu?
3) Kodi bwenzi lanu lathculako mikumano iyi kwa wina aliyense?
   a) Funsitsani: Anzawo,achibale, anthu a mudela
4) Kodi bwenzi lanu linapangapo chani pa zinthu izi? Anazikonda/sana zikonde? Anapanga chani zitachitika?
   a) Funsitsani: Lamya ya m'manja/uthenga wa pa lamya, kupita okha , thandizo la ARV lapakhomo
5) Munanva bwanji kuzinthuzi? Munazikonda/simunazikonde?
   a) *Achibale anapanga chilichonse, anzanu,anthu a mudela ku zinthu zimenezi?*
   b) Ngati zili choncho, izi zinawatani a bwenzi anu /inuyo

SUGGESTIONS FOR ART SERVICES

Zikomo chifukwa cha mayankho anu. Timafuna titakhazikitsa ndondomeko zokomera anthu mu dera lanu pokhudza thandizo la ma ARV. Tikudziwa kuti amuna amakumana ndi zophinja/mavuto osiyana

For peer review only

ndi amayi. Ndimafuna ndimve maganizo anu pa zofunika kuti tikonze bwino thandizoli kuti likhale losavuta kufikira ndi kugwiritsa ntchito.

1) Momwe mukuganizira, kodi mungandiuzeko njira yabwino kwambiri yomwe mukuganiza kuti thandizo la ARV lingamaperekedwere kwa bwenzi lanu? Chipatala chitati chichitepo kanthu….
    a) Kodi bwenzi lanu lingafune lidzipita nthawi zotani ku chipatala kukatenga mankhwala
        i) Tsiku la mu sabata, nthawi?
        ii) *Ndi chifukwa chani?*
    b) Kodi ndi malo ati omwe bwenzi lanu lingakonde kumakatengerako ARV?
        i) Pa chipatala cha pafupi nanu, kutali nanu, kwina mu dela lino (MALO ATI KWENIKWENI) pakhomo panu
        ii) *Ndi chifukwa chani?*
2) Kodi mukuganiza kwanu, mukuona kuti bwenzi lanu likufunika uthenga wina owonjezera? Pa zokhudza ubwino wa ARV, momwe lingasungire chinsinsi cha momwe mthupi mwawo mulili kapena kuwauza ena za mthupi mwawo, zina zikhuzana ndi thanzi lawo?
    a) Bwenzi lanu lingakonde litalandira mu njira yotani uthengawu? Kukawapeza paokha (paokha, kulembedwa mu bukhu, pa wailesi, kuimbiridwa foni)
    b) Ndi NDANI amene bwenzi lanu lingakonde kulankhula naye za uthengawu? (Dokotala, Expert Client, anthu ena a mu dera)
3) Kodi mukuganiza kuti bwenzi lanu likufunika thandizo lapadera? Mwachitsanzo, kukumbutsidwa kuti lipite ku chipatala, kulankhula ndi munthu wina mowirikiza pa zomwe akukumana nazo pakumwa ARV, kuthandizidwa kuuza ena za mmomwe mthupi mwawo mulili ndi zina)
    a) *Bwenzi lanu lingakonde litalandira mu njira yotani thandizoli? Kukawapeza pawokha (pawokha, kulembedwa mu bukhu, pa wailesi, kuimbiridwa foni)*
    b) *Ndi NDANI amene bwenzi lanu lingakonde kulankhula naye za thandizoli? (Dokotala, Expert Client, anthu ena a mu dera)*
4) Potengera thandizo ndi chisamaliro cha ma ARV, pakati pa nthawi yokatengera mankhwala, malo ake, uthenga owonjezera ndi thandizo la padera, kodi mukuganiza kuti chofunikira kwambiri ndi chiti kwa bwenzi lanu?
    a) *Ganizirani amuna ena a mdera lanu. Kodi mukuganiza kuti iwo angatchule chani ngati chinthu chofunikira kwambiri kwa amuna pa zomwe tachula zija?*

SPECIFIC SUGGESTIONS ON CURRENT INTERVENTIONS

<u>FOR MEN WHO ARE CONSIDERED LOSS-TO-FOLLOW UP</u>

1) Monga takambirana kale poyamba,bwenzi lanu linalandira (Kuyimbilidwa lanya, uthenga wa pa lamya, malangizo, thandizo la ma ARV la pakhomo)
    a) Mukuwona ngati izi zinali zokwanira kulimbikitsa bwezi lanu kuyamba ma ARV?
        i) Ngati ayi ndi chani mukanapanga mosiyana? (malo)
        ii) Ngati eya mukuwona ngati zili zofunika chifukwa chani?
2) Kodi pali maganizo ena omwe mukuona kuti angathandizepo kupatula omwe tatchulawa (kukumbutsana, uphungu wapadera, kugawa mankhwala kumudzi) ndi chani?

For peer review only

a) Ndi chani?
b) Ndi chifukwa chani mukuona kuti zikhoza kuyenda bwino

CONCLUSION

1) Zikomo chifukwa cha nthawi yanu. Pali choonjezera china chilichonse chomwe mungalankhulepo chokhudza kagwiritsidwe ntchito ka thandizo la ARV ndi amuna?
2) Pali chilichonse chokhudza kagwiritsidwe ntchito kanu ka thandizo la ma ARV ndi momwe tingakonzere thandizoli?

STOP THE RECORDER AND MAKE SURE RECORDING IS SAVED.

ZIKOMO CHIFUKWA CHOTENGA NAWO MBALI MU KAFUKUFUKUYU. NDIMAFUNA NDIKULIMBIKITSENI KUTI MA ARV AKHOZA KUTHANDIZA INU KUKHALA MOYO WAUTALI NDINSO WATHANZI.

The following general education messages should be conveyed to all male and female participants:

- Anthu onse omwe ayezetsa ndi kupezeka ndi kachilombo ka HIV akuyenera kuyamba kumwa mankhwala a ma ARV mwamsangamsanga kuti zipundulire thanzi lawo komanso apewe kupatsira ena kachilombo.

- Matenda oopsa akhoza kugwira munthu ngakhale amene chiwerengero cha asilikali a mnthupi ndi ochuluka (kuposera 500) posaonetsa zizindikiro zoyamba. Mankhwala a ma ARV amachepetsa chiopsezochi.

- Anthu omwe ayamba kumwa ma ARV ndipo akupitiliza moyo wawo onse osalekalekeza akhoza kukhala moyo wa thanzi ndi wautali chimodzimodzi anthu omwe alibe kachilombo ka HIV.

- Ngakhale simukumva kudwala, mankhwala a ARV ndi ofunikirabe kuti mukhale ndi thanzi moyo wanu onse.

- Makhwala a ma ARV amachepetsa mlingo wa tidzilombo mthupi mwanu kotero amachepetsa chiposezo chopatsira kachilombo kwa ena.

- Mankhwala omwe alipo pakadali pano a ma ARV ndiosavuta kumwa komanso sakhala ndi mavuto ambiri. Anthu ena amakhala ndi mavuto obwera kamba komwa mankhwala masabata oyambirira koma izi zimatha. Ngati zikupitilirabe pali mtundu wina wa mankhwala omwe akhoza kukupatsani.

14.11. APPENDIX K: In-Depth Interview Guide – Male

IN-DEPTH INTERVIEW GUIDE
Identifying efficient linkages strategies for HIVST (IDEaL)
Male

BEGIN RECORDING

Original Study ID: ____________________

Repeat Original Study ID:

State if male or female respondent ______________________

Health Facility: ______________________

Date of Interview: ______________________

Full Name of Interviewer: ________________________________

District where respondent Lives: ________________________________

DEMOGRAPHICS:

1. What is your current age in years?	Age in [ ][ ]<br>Completed Years
2. How would you rate your health today on a scale from 1-5 with 1 being excellent health and 5 being the very poor health?	☐ Excellent (1)<br>☐ Very good (2)<br>☐ Good (3)<br>☐ Fair (4)<br>☐ Poor (5)
3. Now I'd like to ask about your relationships. Are you currently in a sexual relationship?	☐ Yes (1)<br>☐ No (0)<br>**If NO, skip to QUALITATIVE**
4. Does your partner know <u>your</u> HIV status?	☐ Yes (1)<br>☐ No (0)

For peer review only - http://bmjopen.bmj.com/site/about/guidelines.xhtml

5. How many times have you tested for HIV?	
6. When was the first time you tested HIV-positive?	

Open- Ended Questions

Note: The in-depth interview will be open-ended and guided by the respondent's answers. This outline reflects a general guide for the in-depth interviews.

The interviews are meant to help us understand barriers and facilitators to ART initiation. We are also interested in their thoughts on new interventions we are developing to help men start ART. The following questions are meant to guide interviewers. Actual questions asked during the interview will vary based on participant responses.

SCRIPT: Now I'd like to talk to you about your experience with HIV services.

HIV TESTING

1. Think about when you used an HIV self-test kit (show the kit). When did you use it?
2. How did you feel after receiving the HIV positive status?
3. Can you talk to me about what happened after you tested HIV positive? Walk me through it so I can see the picture in detail?.
    a. Did you talk to anyone about it?
    b. Has your HIV status changed your daily activities at all? (Your schedule/routine)
    c. Has your HIV status impacted your relationships? How?
    d. Have you disclosed your status to anyone besides your partner? Why/why not

INTERVENTION

1. After you tested positive, we approached you to be a part of our study. Since you were enrolled in the study (ie in the last 3 months), can you walk me through what has happened related to HIV health services?
    a. Probe: What kinds of interactions have you had with expert clients or health personelle (phone call, SMS, in-person visits, home based ART).
    b. What was the frequency of these interactions (weekly, every other week, monthly)

ART INITIATION

2. Since you have been enrolled in the study, have you initiated ART?

INITIATED

3. How long did it take you to initiate ART? (several weeks, several months?). Where did you initiate?
    a. Why did you initiate ART so quickly/slowly?
4. Since you initiated treatment, have you continued to take you medication?

For peer review only

a. Have you returned to the clinic for another refill of ART? Have there been challenges to staying on treatment – how have you overcome them?

NOT INITIATED

1) Since you tested HIV-positive, have you been to a health facility?
   a) When did you attend? (year)
   b) Why did you attend? (guardian vs client; HIV vs OPD)
   c) Why did you not initiate ART during this visit?

ART UPTAKE

2) We know starting ART is difficult. What do you think is most the most difficult thing about starting ART?
   a) PROBE: Think about your male friends. What do you think they would say is the most difficult part about starting ART for men in your village?

INITITATED ART

1) How has taking ART affected your daily activities at all? (your schedule/routine) In bad ways? In good ways?
2) How has taking ART affected your relationships? In bad ways? In good ways?
3) Does being on ART change if you are able to hide/keep your HIV status from other people/ If you are able to hide/keep your status other people, does that make it easier to be on ART? How?

NOT INITITATED ART

1) How do you think taking ART would affect your daily activities at all? (your schedule/routine) In bad ways? In good ways?
2) How do you think taking ART would affect your relationships? In bad ways? In good ways?
3) Would being able to hide/keep your HIV status from other people make it easier for you to be on ART? How?

4) What do you think is most the SECOND most difficult thing about starting ART?

   a) PROBE: Think about your male friends. What do you think they would say is the SECOND most difficult part about starting ART for men in your village?

SUGGESTIONS FOR ART SERVICES

Thank you for all the information. We would like to develop ART services that meet the needs of men in your community. We understand that men are busy and may face different challenges than women. I would like to know your opinion about what is needed in order to make ART services easy to use for men in your community

1) Could you describe the ideal way ART services would be given to you? If the clinic could do anything
   a) When would you want to pick up ART?
   b) Probe: day of week, time of day?
   c) Why do you say this?
2) Where would you want to pick up ART?
   a) Clinic near you, clinic far from you, somewhere in the community (WHERE SPECIFICALLY), at your home?
   b) Why do you say this?

For peer review only

I would like to learn about how you felt about each of the interactions we talked about earlier (remind participant of what they mentioned – ex: phone calles, texts, in person visits, home based ART)

1) What did you like about them?
   a) Why? (ex: individual follow-up, sense of support, not having to travel to the clinic for homebased ART)
2) What did you dislike about them? What were challenges?
   a) Why? (ex: difficulty maintaining privacy with contact or visits, doesn't want to start ART for other reasons)
3) Do you feel like these things helped encourage you to seek health services?
   a) If YES, why?
   b) If NO, why?
4) Do you think these things would help other men in your community if they were to test positive for HIV?

<u>INITIATED</u>

1) Do you feel that these things helped to encourage you to initiate and stay on ART?
2) Do you think you would have started treatment without them?

<u>NOT INITIATED</u>

1) Why do you think these things failed to help you start/stay on treatment?
   a) Why?
2) If you could change anything about these interactions that you have listed, what would you change?
   a) Probe: Type of contact, frequency of contact, personelle, location, topics covered

3) We understand that everyone is different. Beyond what you have experienced, do you still have problems related to seeking health services for HIV? (i.e. are there still things that you need?)
   a) What are these unmet needs?
   b) What do you feel would be the best solution to meet those needs?
4) Are there any other ideas/services we should think about doing beside the ones we just talked about (appointment reminders, in-depth counseling, community/home ART)?
   a) What is it?
   b) Why do you think this could work?
5) Is there anything else that you would like to add as we are towards the end of the interview?

STOP THE RECORDER AND MAKE SURE RECORDING IS SAVED.

THANK YOU FOR YOUR PARTICIPATION IN THIS INTERVIEW. LET ME ENCOURAGE YOU THAT ARVS CAN HELP YOU LIVE LONG AND HEALTHY.

The following general education messages should be conveyed to all male and female participants:

- All people who have been tested HIV positive should start ART as soon as possible for their own health and to prevent passing the virus on to others.

For peer review only

- Serious diseases can occur even in patients with high CD4 count (>500), without any previous symptoms. Immediate ART greatly reduces this risk.
- People that start ART and continue lifelong without interruptions can remain healthy and live as long as people without HIV.
- Even though you may not feel sick, ART is still important to keep you healthy for the rest of your life.
- ART reduces the amount of virus in your body and therefore can reduce the chance that HIV is passed to your sex partners.
- Current ART regimens are easy to take and rarely cause serious side-effects. Some people have side effects in the first few weeks of treatment and these almost always go away. IF there are persistent side effects, an alternative HIV regimen can be given.

Be careful not to give any specific medical advice but rather refer respondents back to the clinic to speak to a provider.

For peer review only

IN-DEPTH INTERVIEW GUIDE
Identifying efficient linkages strategies for HIVST (IDEaL)
Male (Chichewa)

BEGIN RECORDING

Original Study ID: ______________________

Repeat Original Study ID:

State if male or female respondent ______________________

Health Facility: ______________________

Date of Interview: ______________________

Full Name of Interviewer: __________________________________

District where respondent Lives: __________________________________

1. Kodi pakadali pano muli ndi zaka zingati zakubadwa?	
2. Kodi thanzi lanu mungati lilibwanji lero pa mlingo wa 1 mpaka 5, pamene 1 akuyimira thanzi labwino kwambiri ndi 5 akuyimira kuti thanzi lanu silili bwino konse?	☐ Lili bwino kwambiri (1)<br>☐ Lilibwino (2)<br>☐ Pakati mpakati (3)<br>☐ Silili bwino (4)<br>☐ Silili bwino ngakhale pang'ono (5)
3. Pano ndimafuna ndikufunseni za maubwenzi. Kodi pakadali pano muli pa ubwenzi ogonana?	☐ Eya (1)<br>☐ Ayi (0)<br>**If NO, skip to QUALITATIVE**
4. Kodi bwenzi lanu limadziwa kuti mthupi mwanu muli bwanji kumbali ya HIV?	

For peer review only - http://bmjopen.bmj.com/site/about/guidelines.xhtml

5. Mwayezetsa kokwana kangati HIV?	
6. Kodi ulendo oyamba omwe mudayezetsa ndi kukupezani ndi kachilombo ka HIV ndi liti? (chaka)	

Pano ndimafuna ndikufunseni mafunso okhudzana ndi zomwe mwakumana nazo zokhudza ndi thandizo la HIV.

HIV TESTING

1) Ganizirani nthawi yomwe mudagwiritsa ntchito kachipangizo kodziyezera wekha HIV (awonetseni), kodi liti mudagwiritsa kachipangizoka?
2) Kodi munamva bwanji mutaona kuti zikuonetsa kuti muli ndi kachilombo ka HIV?
3) Mungandifotokozele zomwe zinachitika mutayezetsa ndikupezeka ndi HIV? Mugafotokoze mwatsatanetsatane kuti ndione chithunzithunzi cha momwe zinalili?
   a) Munalankhula ndi aliyense?
   b) Kodi kudziwa kuti muli ndi kachilombo ka HIV kunasintha ntchito zanu za tsiku ndi tsiku?
   c) Kodi kudziwa kuti muli ndi kachilombo ka HIV kunakhudza maubwenzi anu? Motani?
   d) Kodi munayamba mwauzapo za momwe mthupi mwanu mulili kupatula kwa okondedwa anu? Chifukwa chani?

INTERVENTION

1) Chifukwa choti munapezeka ndi HIV, tinakupezani kuti mutenge nawo mbali mu study yathu. Poti munatenga nawo mbali mu study (miyezi itatu yapitayi),mungandifotokozere zomwe zakhala zikuchitika zokhudza ndi thandizo la HIV?
   a) Mwakhala ndi mikumano yotani ndi omwe ali kale pa ma ARV kapena a zaumoyo(pa lamya,uthenga wa lamya yam'manja, kuyankhulana pamaso, kupita nokha kukakumana nawo,kulandira thandizo la ma ARV pakhomo).
   b) Kodi mikumanoyi imachitika pafupipafupi bwanji?

ART INITATION

1) Chiloweleni mu study, mwayamba kulandira ma ARV?

OYAMBA MANKHWALA

2) Kodi zinatenga nthawi yaitali bwanji musanayambe kumwa mankhwala a ARV? (masabata angapo, miyezi ingapo?) Kodi mukanayambira kuti? Kodi munayamba kumwa mankhwala mnsanga/mochedwa chifukwa chani?
3) Chiyambileni kulandira thandizo la ARV, kodi mukupitiliza kumwa mankwala anu?
   a) Mwapitako ulend wina kuchipatala kukalandira mankwala ena a ama ARV? Mwakhala mukukumana ndi zovuta zina chifukwa chokumwa mankhwala ama ARV, kodi mavutowa mwawathetsa bwanji?

For peer review only

OSAYAMBA MANKHWALA

4) Mutayezetsa nkupezeka ndi kachilombo ka HIV, munayamba mwapitapo ku chipatala?
   a) Munapita liti? (chaka)
   b) Munapita chifukwa chani? (kuperekeza odwala wina kapena munadwala?; munapitila HIV kapena ku OPD)
   c) Kodi simunayambe kumwa mankhwala a ma ARV nthawi imeneyi chifukwa chani?

ART UPTAKTE

1) Tikudziwa kuti kuyamba kumwa mankhwala a ARV kumavutirapo. Kodi mukuona kuti chovuta chachikulu pa kuyamba kumwa mankhwala ndi chani?
   a) Ganizirani anzanu aamuna mmudzi mwanu. Kodi mukuona kuti iwo angatchule chani ngati chovuta chachikulu pa kuyamba kumwa mankhwala a ART?

OYAMBA MANKHWALA

2) Kodi kumwa mankhwala kwakhudza bwanji ntchito zanu za tsiku ndi tsiku? Mu njira yabwino kapena yobwerera mmbuyo?
3) Kodi kumwa mankhwala kwakhudza bwanji maubwenzi anu? Mu njira yabwino kapena yobwerera mmbuyo?
4) Kodi kumwa mankhwala a ARV kumaphweka mukabisa momwe mthupi mulili kwa anthu ena? Motani?

OSAYAMBA MANKHWALA

5) Kodi mukuganiza kuti kumwa ma ARV kungakhudze bwanji ntchito zanu za tsiku ndi tsiku? Mu njira yabwino kapena yobwerera mmbuyo?
6) Kodi mukuganiza kuti kumwa mankhwala a ma ARV kungakhudze bwanji maubwenzi anu? Mu njira yabwino kapena yobwerera mmbuyo?
7) Kodi kumwa mankhwala a ARV kungaphweke/kumaphweka kuti mukabisa momwe mthupi mulili kwa anthu ena? Motani?

8) Kodi chinthu chachiwiri chovuta kwambiri pa kuyamba kumwa mankhwala ndi chani?
   a) Ganizirani amuna ena a mmudzi mwanu. Kodi mukuganiza kuti anganene kuti chovuta CHACHIWIRI pa kuyamba kumwa a ma ARV ndi chani mmudzi mwanu?

SUGGESTIONS FOR ART SERVICES

Zikomo chifukwa cha mayankho anu. Timafuna titakhazikitsa ndondomeko zokomera amuna mu dera lanu zokhudza thandizo la ma ARV. Tikumvetsetsa kuti amuna amakhala otangwanidwa ndipo amakumana ndi mavuto osiyana ndi amayi. Ndimafuna ndimve maganizo anu pa zofunika kuti tikonze bwino thandizoli maka kwa amuna mu dera lino.

For peer review only

1) Kodi mungandiuzeko njira yabwino kwambiri yomwe mukuganiza kuti thandizo la ma ARV lingamaperekedwere kwa inu, chipatala chitati chichitepo kanthu….
   a) Kodi mungafune mudzipita nthawi zotani ku chipatala kukatenga mankhwala?
      i) Tsiku la mu sabata, nthawi?
      ii) Ndi chifukwa chani?
   b) Kodi ndi malo ati omwe mungakonde kumakatengerako ARV?
      i) Pa chipatala cha pafupi nanu, kutali nanu, kwina mu dela lino (MALO ATI KWENIKWENI) pakhomo panu
      ii) Ndi chifukwa chani?
2) Ndimafuna ndinve za momwe mukunvera za kucheza konse tinali nako poyamba(Kumbutsani otenga nawo mbali zomwe anatchula-kuyimbilidwa lamya, uthenga wa pa lamya, kupitako okha, thandizo la pakhomo la ARV)
   a) Chomwe munakondapo chinali chani?
      i) Chifukwa?(Kuyendeledwa, kunva kuthandizidwa, osafunika kupita ku chipatala kukalandira ma AR olandilira pakhomo)
   b) Chomwe simunaonde ndi chani? Zovuta zinali chani?
      i) Chifukwa? (chitsanzo: kuvutika kusunga chinsinsi ndi owadziwa kapena mikumano,simukufuna kuyamba kumwa ma ARV pazifukwa zina)
   c) Mukuganiza kuti zinthu zimennezi zakulimbikitsani kupeza thandizo la zaumoyo?
      i) Ngati eya , chifukwa?
      ii) Ngati ayi, chifukwa chani?
   d) Kodi mukuona ngati zinthu zimenezi zingathandize anthu ena mu dela lanu ngati angakhale ndi HIV?

OYAMBA MANKHWALA

1) Kodi mukuona ngati zinthu zimenezi zinakuthandizani kulimbikitsika kuti muyambe komanso kupitiliza kumwa mankhwala a ma ARV?
   a) Kodi mukuganiza kuti mukanatha kuyamba thandizo la ma ARV popanda zimenezi?

OSAYAMBA MANKHWALA

1) Kodi mukuganiza kuti zinthu zimenezi zinalephera kukuthandizani kuyamba/kukhala mankhwala a ma ARV?
   a) Chifukwa?
2) Ngati mungathe kusintha chilichonse cha mikumano yomwe mwatchula,chingakhale chani?
   a) Mtundu wa mkumano, muligo wa mkumano, muthu, malo,mitu yokambilana
3) Tikunvetsa kuti anthu ndife osiyana. Kuposa zomwe mwakumana nazo, pakadali pano mukukumanabe ndi mavuto okhudzana ndi kupeza thandizo la zaumoyo la HIV? (pali zinthu zina zomwe mumafunabe?)
   a) Ndi zinthu ziti?
   b) Mukuganiza kuti ndi njira yanji yabwino yothandiza kupeza zofunikazi?

For peer review only

4) Kodi pali maganizo ena omwe mukuona kuti angathandizepo kupatula omwe tachulawa (kukumbutsana, uphungu wapadera, kugawa mankhwala kumudzi) ndi chani? Mukuona kuti maganizo anuwa angatheke chifukwa chani?\
5) Apa tikufunakumaliza kucheza kwathu, pali china chiwinjezera chomwe mungalankhulepo pa zomwe takambirana?

STOP THE RECORDER AND MAKE SURE RECORDING IS SAVED.

ZIKOMO CHIFUKWA CHOTENGA NAWO MBALI MU KAFUKUFUKUYU. NDIMAFUNA NDIKULIMBIKITSENI KUTI MA ARV AKHOZA KUTHANDIZA INU KUKHALA MOYO WAUTALI NDINSO WATHANZI.

The following general education messages should be conveyed to all male and female participants:

- Anthu onse omwe ayezetsa ndi kupezeka ndi kachilombo ka HIV akuyenera kuyamba kumwa mankhwala a ma ARV mwamsangamsanga kuti zipundulire thanzi lawo komanso apewe kupatsira ena kachilombo.
- Matenda oopsa akhoza kugwira munthu ngakhale amene chiwerengero cha asilikali a mnthupi ndi ochuluka (kuposera 500) posaonetsa zizindikiro zoyamba. Mankhwala a ma ARV amachepetsa chiopsezochi.
- Anthu omwe ayamba kumwa ma ARV ndipo akupitiliza moyo wawo onse osalekalekeza akhoza kukhala moyo wa thanzi ndi wautali chimodzimodzi anthu omwe alibe kachilombo ka HIV.
- Ngakhale simukumva kudwala, mankhwala a ARV ndi ofunikirabe kuti mukhale ndi thanzi moyo wanu onse.
- Makhwala a ma ARV amachepetsa mlingo wa tidzilombo mthupi mwanu kotero amachepetsa chiposezo chopatsira kachilombo kwa ena.
- Mankhwala omwe alipo pakadali pano a ma ARV ndiosavuta kumwa komanso sakhala ndi mavuto ambiri. Anthu ena amakhala ndi mavuto obwera kamba komwa mankhwala masabata oyambirira koma izi zimatha. Ngati zikupitilirabe pali mtundu wina wa mankhwala omwe akhoza kukupatsani.

Be careful not to give any specific medical advice but rather refer respondents back to the clinic to speak to a provider.

14.12. APPENDIX L: Personel CV's

Kathryn L. Dovel

Department of Medicine, Division of Infectious Diseases Email: kdovel@mednet.ucla.edu
David Geffen School of Medicine at UCLA Office: +1 (310) 825-7225
10833 Le Conte Ave 37-121 CHS Fax: +1 (310) 825-3632
Los Angeles, CA 90095

POSITIONS HELD

2017- Adjunct Assistant Professor, Division of Infectious Disease
Department of Medicine, David Geffen School of Medicine - UCLA

2017- Research Director - Partners in Hope
Lilongwe, Malawi

2016-17 Postdoctoral Fellow in Global HIV Prevention Research
David Geffen School of Medicine - UCLA, 2016

2012-15 International Programs Director - 31Bits International
Gulu, Uganda

EDUCATION

PhD Health and Behavioral Sciences - University of Colorado Denver, 2016

Outstanding CLAS Ph.D. Student for the University of Colorado Denver

Outstanding Dissertation Award for the University of Colorado Denver

Dissertation: "Shifting Focus from Individuals to Institutions: The Role of Gendered Health Institutions on Men's use of HIV Services"

MPH Community Health Sciences - UCLA, 2010

Certificate in Global Health

BA Sociology & Anthropology (dual major), minor in Biology - Vanguard University, 2007

Magna Cum Laude

HONORS AND AWARDS

2019 5th place in the 2019 Department of Medicine Research Day poster competition, Department of Medicine, University of California Los Angeles

2019 Invited panelist, IAS 2019, Sticky and durable linkage: Latest evidence and new strategies

2019 Panel Chair, IAS Pre-Conference 2019, Men and HIV: What we know and what we don't know

2019 Top 5 abstracts at CROI, 2019

For peer review only

2019 Invited participant, Technical Consultation on HIV Linkage, International AIDS Society

2019 Invited participant, Differentiated Service Delivery Think Tank, Gates Foundation

2018 Joep Lange Award (best abstract at INTEREST, 2018)

2018 Female Global Scholar, The Women in Global Health Research Initiative (Weill Cornell Medicine, Cornell University)

2016 Outstanding Dissertation Award (UCDenver)

2016 Outstanding CLAS Ph.D. Student (UCDenver)

2012-16 Deans Travel Grant (UCDenver)

2007 Delta Kappa Honor Society (Vanguard University)

2007 Alpha Kappa Delta Honor Society (Vanguard University)

2007 Lambda Alpha Honor Society (Vanguard University)

2007 Anthropology Student of the Year (Vanguard University)

EXTERNAL GRANTS

2019-2023 Principle Investigator, Bill and Melinda Gates Foundation. (001423) "Identifying Effective Linkage Strategies for HIVST (IDEaL)"

2019-2024 Principle Investigator, Fogarty International Center. International Research Scientist Development Award (K01), K01TW011484. "Innovative strategies to increase ART Initiation and viral suppression among HIV+ men in Malawi".

2019-2020 Principle Investigator, Clinton Health Access Foundation. "The impact of facility HIV self-test scale up in Malawi: a mixed methods study"

2018-2021 Co-Investigator, The Conrad N. Hilton Foundation. Delivery of childhood development services as part of HIV treatment services in Malawi. Project implemented by UCLA and Partners in Hope.

2017-19 Principal Investigator, USAID. **Use of HIV self-test kits to increase identification of HIV-infected individuals and their partners: a Cluster Randomized Control Trial.** (sub-study within a large PEPFAR-USAID grant; PI: Risa Hoffman).

2017-19 Principle Investigator, Clinical Research Scholar, National Institutes of Health Loan Repayment Program.

2016-18 Co-Principle Investigaor, USAID. Test and Start: Tracking Uptake and Retention in Care using Standard Registry Data. (sub-study within a large PEPFAR-USAID grant; PI: Risa Hoffman).

2015 Principal Investigator, 31Bits International. "Evaluating the impact of a couple's livelihoods program on power dynamics and economic attainment among couples in northern Uganda." Project implemented by 31Bits International.

2014-16 Principal Investigator, NIMH National Research Service Award Predoctoral Individual Fellowship, F31-MH103078-01A1, "Gender Disparities in High-Risk PITC: The Role of Policy on Provider Practices", Impact Score: 14; Percentile: 2.0

2013-16 Principal Investigator, Stop AIDS Now!. "Evaluation of the 'Quality HIV and reproductive maternal and neonatal health services for women and young women in Africa through good

For peer review only

clinical governance and community-driven accountability'". Project implemented by the Clinton Health Access Initiative.

INTERNAL GRANTS

2016-18 Principal Investigator, UCLA Center for AIDS Research Seed Grant, University of California Los Angeles, "The Gendered Dynamics of ART Uptake and Retention under Universal Treatment Policies. Examining trends and ART barriers in Central Malawi"

2014 Principal Investigator, Calvin L Wilson Scholarship, University of Colorado Denver, "Gender and the provision of HIV testing: Examining how models of care influence men's use of testing services in southern Malawi"

2013-15 Principal Investigator, Dissertation Grant, University of Colorado Denver, "Gender Disparities in High-Risk PITC: The Role of Policy on Provider Practices"

2013-14 Principal Investigator, Robinson Durst Scholarship, University of Colorado Denver, "Gender disparities in high-risk PITC: Exploring the influence of feminized policy on provider practices in Malawi"

2009 Principal Investigator, Drabkin and Bixby International Scholarship, UCLA, "Evaluating barriers and facilitators of a nutrition program in the Bateyes of Dominican Republic"

2009 Principal Investigator, Global Health Grant, UCLA, "Evaluating a Nutrition Program in the Bateyes of Dominican Republic"

PUBLICATIONS

* represents MPH, PhD or medical students I mentored

2020 Cornell, Morna, Katherine Horton, Christopher Colvin, Andrew Medina-Marino, **Kathryn Dovel.** Raising the profile of men's health: the role of the research community: Letter to the editor. *Lancet*. Ahead of Print.

2020 **Dovel, Kathryn**, Mike Nyirenda, Frackson Shaba*, O. Agatha Offorjebe*, Kelvin Balakasi, Brooke Nichols, Khumbo Phiri*, Khumbo Ngona, Sundeep K Gupta, Risa Hoffman. "Facility-based HIV self-testing for outpatients dramatically increases HIV testing in Malawi: a cluster randomized trial." *Lancet Global Health*. Ahead of print

2020 **Dovel, Kathryn,** Khumbo Phii, Misheck Mphande, Deborah Mindry, Esnart Sanudi, McDaphton Bellos, Risa Hoffman. Optimizing Test and Treat in Malawi: Health care worker perspectives on barriers and facilitators to ART initiation among healthy clients. Global Health Action. Ahead of Print.

2020 Hubbard, Julie, Khumbo Phiri*, Corrina Moucheraud, Kaitlyn McBride, Ashley Bardon, Kelvin Balakasi, Eric Lungu, **Kathryn Dovel**, Gift Kakwesa, Risa Hoffman. A qualitative assessment of provider and client experiences with three- and six-month dispensing of antiretroviral therapy in Malawi. Global Health: Science and Practice. Ahead of Print.

For peer review only - http://bmjopen.bmj.com/site/about/guidelines.xhtml

2020 Hoffman, Risa M, Kelvin Balakasi, Ashley Bardon, Zumbe Siwale, Julie Hubbard, Gift Kakwesa, Mwiza Haambokoma, Thoko Kalua, Pedro Pisa, Crispin Moyo, **Kathryn Dovel**, Thembi Xulu, Ian Sanne, Matt Fox, Sydney Rosen. Eligibility for differentiated models of HIV treatment service delivery: an estimate from Malawi and Zambia. *AIDS.* 1;34(3):475-9.

2019 McBride, Kaitlyn, Julie Parent, Kondwani Mmanga, Mackenzie Chivwala, Mike H. Nyirenda, Alan Schooley, James B. Mwambene, **Kathryn Dovel**, Eric Lungu, Kelvin Balakasi, Risa M. Hoffman, Corrina Moucheraud. "ART Adherence Among Malawian Youth Enrolled in Teen Clubs: A Retrospective Chart Review." *AIDS Behav.* (2019): 1-5.

2019 Frackson Shaba*, Ogechukwu Offorjebe*, Phiri Khumbo, Lungu Eric, Kalande Pericles, Nyirenda Mike, Hoffman M Risa, Gupta Sundeep, **Dovel Kathryn**. Perceived Acceptability of a Facility-Based HIV Self-Test Intervention in Outpatient Waiting Spaces Among Adult Outpatients in Malawi: A Formative Study. *JAIDS*. 1;81(3):e92-4.

2019 Magaço Amílcar, **Dovel Kathryn**, Cataldo Fabian, Nhassengo Pedroso, Nuera Lucas, Tique José, Saide Mohomed, Couto Aleny, Mbofana Francisco, Gudo E Eduardo, Cuco Rosa Marlene, Chicumbe Sérgio. “Good health as a barrier and facilitator to ART initiation: a qualitative study in the era of Test and Treat in Mozambique.” *Cult Health Sex. 11:1-5.*

2018 Cornell M, **Dovel K**. Reaching key adolescent populations. *Cur Opinion HIV AIDS.* 1;13(3):274-80.

2018 Sara, Yeatman, Stephanie Chamberlin*, **Kathryn Dovel.** Women’s (health) work: A population-based, cross-sectional study of gender differences in time spent seeking health care in Malawi. *PLoS ONE.* 13(12): e0209586

2018 Nhassengo, Pedroso Fabian Cataldo, Amílcar Magaço, Risa Hoffman, Lucas Nuera, José Tique, Mohomed Saide, Aleny Couto, Francisco Mbofana, Eduardo Gudo, Rosa Marlene Cuco, Sérgio Chicumbe, **Kathryn Dovel**. “Barriers and facilitators to the uptake of universal treatment in Mozambique: a qualitative study on patient and provider perceptions.” *PLoS ONE.* 13(12): e0205919

2018 Hubbard, Julie, Gift Kakwesa, Mike Nyirenda, James Mwambeneb, Ashley Bardona, Kelvin Balakasi, **Kathryn Dovel**, Thokozani Kaluac, Risa Hoffman. Towards the third 90: improving viral load testing with a simple quality improvement program in health facilities in Malawi. *International Public Health.* Ahead of print.

2018 **Dovel, Kathryn,** Frackson Shaba*, Ogechukwu Offorjebe*, Kelvin Balakasi, Khumbo Phiri*, Brooke Nichols, Chi-Hong Tseng, Ashley Bardon, Khumbo Ngona, Risa Hoffman. “Evaluating the integration of HIV self-testing into low-resource health systems: study protocol for a cluster randomized trial from EQUIP Innovations” *Trials.* 19:498.

2018 Moucheraud, Corrina, Dennis Chasweka, Mike Nyirenda, Alan Schooley, **Kathryn Dovel**, Risa Hoffman. “A simple screening tool may help identify high-risk children for targeted HIV testing in Malawian inpatient wards.” *JAIDS.* 79:352-7.

2018 Cornell Morna, **Dovel Kathryn**. “Reaching key adolescent populations.” *Current opinion*

For peer review only

in HIV and AIDS. 13(3):274-80.

2016 **Dovel, Kathryn**, Sara Yeatman, Joep Vanoosterhout, Adrienne Chan, Alfred Matengeni, Megan Landes, Richard Bedell, and Sumeet Sodhi. "Trends in ART Initiation among Men and Non-Pregnant/Non-Breastfeeding Women before and after Option B+ in Southern Malawi." *PLoS ONE.* (12): e0165025.

2016 Poulin, Michelle, **Kathryn Dovel** and Susan Watkins. "Men with money and the 'vulnerable women' client category in an AIDS epidemic." *World Development*. 85; 16-30.

2016 **Dovel, Kathryn**, Susan Watkins, Sara Yeatman, and Michelle Poulin. "Prioritizing strategies to reduce AIDS-related mortality for men in sub-Saharan Africa: Author's reply." *AIDS*. 30(1); 158-9.

2015 **Dovel, Kathryn,** Sara Yeatman, Susan Watkins, and Michelle Poulin. "Men's heightened risk of AIDS-related death: the legacy of gendered HIV testing and treatment strategies." *AIDS.* 29; 1123–5.

2015 **Dovel, Kathryn** and Kallie Thomson. "Financial obligations and economic barriers to antiretroviral therapy experienced by HIV positive women participating in a job-creation program in northern Uganda." *Culture, Health, and Sexuality*. 18(6).

2015 Krueger, Patrick, **Kathryn Dovel** and Justin Denney. "Democracy and self-rated health across 67 countries: A multilevel analysis.*" Social Science and Medicine.* 143; 137-44.

2013 Conroy, Amy, Sara Yeatman and **Kathryn Dovel**. "The social construction of HIV/AIDS during a time of evolving access to antiretroviral therapy in rural Malawi." *Culture, Health and Sexuality.* 15(8); 924-37.

2012 Yeatman, Sara, **Kathryn Dovel**, Amy Conroy and Hazel Namadingo. "The predictors of HIV treatment optimism and its relationship with sexual risk behavior among a population-based sample of young adults in southern Malawi." *AIDS Care.* 25(8);1018-25.

TECHNICAL MANUSCRIPTS

2019 Hopkins, John, Laura Pascoe, Dean Peacock and Kathryn Dovel. "Accelerating Men's HIV service delivery and uptake in Eastern and Southern Africa UNAIDS Literature Review, Eastern and Southern Africa Regional Focus." UNAIDS, Johannesburg, South Africa.

2018 Masina, Tobias, **Kathryn Dovel**, Reuben Mwenda on behalf of the Malawi Ministry of Health. "National Guidelines for HIV self-testing." Malawi Ministry of Health Lilongwe Malawi.

2017 Pascoe, Laura, Dean Peacock and **Kathryn Dovel**. "To Get to Zero, We Must Also Get to Men – UNAIDS Literature Review, Eastern and Southern Africa Regional Focus." UNAIDS, Geneva.

2016 Macharia, Faith, Job Akuno, Faith Wanji, Julius Nguku, **Kathryn Dovel**, Caroline Ngare, Fred Nyagah, and Daniel Mwisunji. "National Guidelines for Male Engagement in HIV Services." Kenya Ministry of Health. Nairobi, Kenya.

2016 **Kathryn Dovel**, James Mkandawire, Susan Watkins, Nancy Mulauzi and Sydney Rodney Lungu. "Evaluation of the Good Clinical Governance Project: improving HIV and reproductive health services in Lilongwe, Malawi." Stop AIDS Now!. Lilongwe, Malawi.

For peer review only

OTHER PUBLICATIONS

2019 Kathryn Dovel, Stephanie Chamberlin, Sara, Yeatman. Malawi's Health System Puts Women First. This Isn't Always a Good Thing. *The Conversation: Africa.* Published February 19, 2019. Found at https://theconversation.com/malawis-health-system-puts-women-first-this-isnt-always-a-good-thing-111277

2016 **Dovel, Kathryn**, Sara Yeatman, and Susan Watkins. **Dying from a treatable disease: HIV and the men we neglect. *Huffington Post.* Published February 23, 2016. Found at** http://www.huffingtonpost.com/the-conversation-africa/dying-from-a-treatable-di_b_9295620.html

WORK IN PREPARATION

Dovel, Kathryn. "The gendered organization of HIV services and men's poor use of testing in southern Malawi: consequences of hegemonic masculinity within health institutions." (Revise & Resubmit, JIAS)

Offorjebe , Ogechukwu*, Frackson Shaba*, Kelvin Balakasi, Mike Nyrienda, Risa Hoffman, **Kathryn Dovel**. "Partner-delivered HIV self-testing increases the perceived acceptability of index partner testing among HIV-positive clients in Malawi." (Revise & Resubmit, PLoS ONE)

Dovel, Kathryn, Kelvin Balakasi, Khumbo Phiri*, Frackson Shaba*, O. Agatha Offorjebe*, Sundeep K Gupta, Vincent Wong, Eric Lungu, Brooke Nichols, Mike Nyirenda, Ngona K, Anteneh Worku, Risa Hoffman. "A randomized trial on index HIV self-testing for sexual partners of ART clients in Malawi." (Under Review)

Nichols, Brooke; Offorjebe, O. Agatha; Cele, Refiloe; Shaba , Frackson ; Balakasi, Kelvin; Chivwara, Mackenzie; Hoffman, Risa; Long, Lawrence; Rosen, Sydney; **Dovel, Kathryn**. "Economic evaluation of facility-based HIV self-testing among adult outpatients in Malawi. " (Under Review)

Dovel, Kathryn, Gladies Orobmi, Melanie Beagly*, Kallie Thomson. "Including men without sidelining women: the feasibility of male involvement within resource-strained gender equality programs in sub Saharan Africa." (Under Review)

Dovel, Kathryn and Kallie Thomson. "Evaluating the impact of a couple's livelihoods program on power dynamics and economic attainment among couples in northern Uganda." (In preparation)

Dovel, Kathryn. "Men in global HIV policy: examining discourses of blame and vulnerability." (In preparation)

SELECT PEER-REVIEWED PRESENTATIONS

2020 Moucheraud, Corrina, Samuel W. Lewis, Misheck Mphande, Ben Allan Banda, Hitler Sigauke, Paul Kawale, Aubrey Dkangoma, **Kathryn Dovel**, Alemayehu Amberbir, Agnes Moses, Sundeep Gupta, Risa M. Hoffman. Cervical cancer knowledge and attitudes among HIV-positive men in Malawi." Paper accepted for poster presentation. Conference on Retroviruses and Opportunistic Infections (CROI). Boston, Massachusetts, USA

2019 **Dovel, Kathryn**, Kelvin Balakasi, Khumbo Phiri*, Frackson Shaba*, O. Agatha Offorjebe*, Sundeep K Gupta, Vincent Wong, Eric Lungu, Brooke Nichols, Mike Nyirenda, Ngona K, Anteneh Worku, Risa Hoffman. "Index HIV self-testing among male partners in Malawi:

For peer review only - http://bmjopen.bmj.com/site/about/guidelines.xhtml

predictors of self-testing within a randomized controlled trial". Paper accepted for poster presentation. International AIDS Society. Mexico City, Mexico

2019 **Dovel Kathryn**, Salem Ejigu, Pericles Kalande, Evelyn Udedi, Chipawiru Mbalanga, Lauri Bruns, Thomas Coates. "Beyond the Caregiver: Diffusion of early childhood development knowledge and practices within the social networks of HIV-positive mothers in Malawi". Paper accepted for poster discussion. International AIDS Society. Mexico City, Mexico

2019 **Dovel, Kathryn**, Kelvin Balakasi, Khumbo Phiri*, Frackson Shaba*, O. Agatha Offorjebe*, Sundeep K Gupta, Vincent Wong, Eric Lungu, Brooke Nichols, Mike Nyirenda, Ngona K, Anteneh Worku, Risa Hoffman. "A randomized trial on index HIV self-testing for sexual partners of ART clients in Malawi." Paper accepted for oral presentation. Conference on Retroviruses and Opportunistic Infections (CROI). Seattle, Washington, USA

2019 Ogechukwu Offorjebe, **Kathryn Dovel**, Frackson Shaba, Kelvin Balakasi, Risa Hoffman, Sydney Rosen, Brooke Nichols, for the EQUIP Health team. Cost-effectiveness and national impact of index HIV self-testing in Malawi. Paper accepted for poster presentation. Conference on Retroviruses and Opportunistic Infections (CROI). Seattle, Washington, USA

2018 **Dovel, Kathryn**, Mike Nyirenda, Frackson Shaba*, Ogechukwu Offorjebe*, Kelvin Balakasi, Brooke Nichols, Khumbo Phiri*, Khumbo Ngona, Alan Schooley, Risa Hoffman on behalf of EQUIP Innovation for Health. "Facility-based HIV self-testing for outpatients dramatically increases HIV testing in Malawi: a cluster randomized trial." Paper accepted for oral presentation. International AIDS Society. Amsterdam, Netherlands

2018 Shaba, Frackson*, Kelvin Balakasi, Ogechukwu Offorjebe*, Mike Nyirenda, Risa Hoffman, **Kathryn Dovel** on behalf of EQUIP Innovation for Health. "Facility-based HIV self-testing in Malawi: an assessment of characteristics and concerns among clients who opt-out of testing." Paper accepted for poster presentation. International AIDS Society. Amsterdam, Netherlands

2018 **Dovel, Kathryn**, Mike Nyirenda, Frackson Shaba, Ogechukwu Offorjebe*, Kelvin Balakasi, Brooke Nichols, Khumbo Phiri*, Khumbo Ngona, Alan Schooley, Risa Hoffman on behalf of EQUIP Innovation for Health. "Facility-based HIV self-testing for outpatients dramatically increases HIV testing in Malawi: a cluster randomized trial." Paper accepted for oral presentation. INTEREST. Kigali, Rwanda - awarded the Joep Lange INTEREST award

2018 Offorjebe , Ogechukwu*, Frackson Shaba, Kelvin Balakasi, Mike Nyrienda, Risa Hoffman, **Kathryn Dovel** on behalf of EQUIP Innovation for Health. "Partner-delivered HIV self-testing increases the perceived acceptability of index partner testing among HIV-positive clients in Malawi." Paper accepted for mini-oral presentation. INTEREST. Kigali, Rwanda

2018 Stephanie Chamberlin*, Misheck Mphande, Pericles Kalande, **Kathryn Dovel** on behalf of EQUIP Innovation for Health. "Barriers and facilitators to consistent engagement in HIV care under Test and Treat in Malawi." Paper accepted for poster presentation. INTEREST. Kigali, Rwanda

2017 **Dovel Kathryn,** Khumbo Phiri*, Alan Schooley, Misheck Mphande, Mackenzie Chivwara, Risa Hoffman. "Facility-level barriers to antiretroviral therapy experienced by men in Malawi." Paper accepted for poster presentation. International AIDS Society. Paris, France

2017 Misheck Mphande, Khumbo Phiri*, Mackenzie Chivwara, Mike Nyirenda, Alan Schooley, Rachel Thomas, Risa Hoffman, **Kathryn Dovel**. "Examining Malawi's Rollout of Universal Treatment: Policy Implementation and Provider Perceptions." Paper accepted for poster presentation. International AIDS Society. Paris, France

2016 **Dovel, Kathryn**. "Factors influencing the implementation of provider-initiated testing and counseling (PITC) among STI clients in southern Malawi: A mixed methods study." Paper accepted for poster presentation. International AIDS Society. Durbin, South Africa

2016 Westerhof, Nienke, Dzowela M, **Kathryn Dovel**, E. Banda, J. Chikonda. "Community-driven accountability through advocacy committees: a vehicle for improving HIV and reproductive health services for women living with HIV." Paper accepted for poster presentation. International AIDS Society. Durbin, South Africa

2016 **Dovel, Kathryn**, Patrick Krueger, Shari Dworkin. "Predictors of men's use of HIV testing services in low-income countries: the role of masculinity." Paper accepted for poster presentation. Population Association of America. Washington D.C.

2014 **Dovel, Kathryn**. "Gender in HIV Policy: Examining how gender shapes the dissemination of HIV policies in southern Malawi." Paper accepted for roundtable presentation. American Public Health Association. New Orleans.

2014 **Dovel, Kathryn**. "Gendered care: examining how clinic experiences influence HIV testing decisions among STI patients in southern Malawi." Paper accepted for oral presentation. National Women's Studies Association, San Juan, Puerto Rico.

2013 **Dovel, Kathryn**. "HIV policies and their influence on men's use of care." Paper accepted for oral presentation. International HIV Social Science and Humanities Conference, Paris, France.

2007 **Dovel, Kathryn**. "Social and structural impediments that limit proper healthcare in rural southern Kurdistan." Paper accepted for oral presentation. The Anthropology and Sociology Research Conference, Santa Clara, CA.

INVITED PRESENTATIONS

2019 "Index HIV Self-Testing in Malawi". World Health Organization webinar

2019 "Men's (lack of) access to the health system". UNAIDS. Regional meeting on Accelerating Men's HIV service delivery and uptake in Eastern and Southern Africa.

2019 "Index HIVST in Malawi: a Randomized Control Trial. World Health Organization. Webinar

2019 "*Reaching men and engaging them in HIV care – lessons from Malawi*". Men and HIV forum. International AIDS Society. Mexico City, Mexico

2018 "The impact of HIV self-testing on HIV testing among outpatients in high burden facilities in Malawi: preliminary findings from a cluster randomized control trial" USAID Washington. Washington D.C

2017 "Who benefits from Test and Treat? Understanding gender dimensions of universal treatment policies and gender-specific barriers to care" Malawi Ministry of Health, HIV Treatment Technical Working Group. Lilongwe, Malawi.

2016 "Facility-based barriers to HIV testing among men in Malawi: a systems approach" Malawi Ministry of Health, HIV Treatment Technical Working Group. Lilongwe, Malawi.

2015 "Men's heightened risk of AIDS-related death: the legacy of gendered HIV testing and treatment strategies" United Nations Meeting on Male Engagement. Geneva, Switzerland

2015 "Facility-based barriers to men's use of HIV testing: recommendations for male engagement guidelines." National AIDS Control Council Meeting for the Development of the Male

For peer review only

Engagement Guidelines. Nairobi, Kenya

2014 "Gendered care: examining who 'does gender' in clinical settings and its influence on HIV services for men in southern Malawi." Health Working Group, UCLA. Los Angeles, CA

2013 "From questions to methods: mixed methods approach to disparities research." Course in Qualitative Methods (Doctoral Students). University of Colorado Denver. Denver, C

2010 "Lost in translation: examples of why best-practice nutrition programs fail in rural Dominican Republic." Drabkin and Bixby International Conference, UCLA. Los Angeles, CA

RELEVANT EMPLOYMENT ACTIVITIES

2015- Consultant for Mixed Methods, Invest in Knowledge, Zomba, Malawi

Activities: Oversaw data analysis and write-up for studies implemented by Invest in Knowledge. I focused on qualitative and mixed methods analysis and write-up.

2009-10 Research Specialist, Korean Resource Center, Los Angeles, CA

Activities: Managed data entry and data cleaning and led in data analysis and write-up of a study assessing use of non-communicable disease services among first- and second-generation Korean populations in Los Angeles.

2009 Program Evaluation Fellow, Bataye Relief Alliance, Santo Domingo, Dominican Republic

Activities: Led the assessment of a nutritional program aimed to improve child health outcomes in Haitian populated bateyes in Dominican Republic. I led tool development, training enumerators, data analysis, and write-up

2007-08 Program Coordinator, Orange County Department of Public Health, Santa Ana, CA

Activities: Conducted literature reviews and assisting in the development of interventions to address Alcohol and Drug abuse among young adults in Orange County. Assisted in the protocol development and implementation of interventions.

SERVICE

2019 Committee Member of the Men's HIV Forum at the International AIDS Conference, Mexico City

2018- Member of the Malawi Ministry of Health HIV Self-Testing Guidelines Task Force

2017- Member of the Malawi Ministry of Health HIV Testing Services Technical Working Group

2017- Member of the EQUIP HIV Self-Testing Technical Working Group

2016- Member of the UNAIDS Working Group "Engaging men in solutions for the HIV epidemic: Health systems."

2016- Member of the "Men and HIV Global Working Group"

2016 Reviewer for the APHA 2016 Annual Meeting & Expo

2012-13 Editor of the Health and Behavioral Sciences Peer-Reviewed Journal, University of Colorado, Denver

2011-12 Student Advisory Council Member, University of Colorado, Denver

For peer review only

COURSES TAUGHT

Adjunct Professor

Social determinants of health in the context of HIV services in sub-Saharan Africa – Field Rotation Series (UCLA)

Health, Disease & Globalization: Foundations of Epidemiology (Vanguard University)

Human Sexuality (co-taught, Vanguard University)

Cultural Anthropology (Vanguard University)

Applied Anthropology (Vanguard University)

Qualitative Methods (Vanguard University)

Teaching Assistant

AIDS and Other Sexually Transmitted Diseases (UCLA)

Global Health Issues (UCLA)

Social Determinants of Health (University of Colorado Denver)

Statistical Analysis (University of Colorado Denver)

MENTORSHIP

University of California Los Angeles. David Geffen School of Medicine. Medical Student. Kate Coursey. "Examining characteristics of women who engage in an integrated Early Childhood Development and PMTCT program in Malawi: endline evaluation." 2019-

University of California Los Angeles. David Geffen School of Medicine. Medical Student. "Provider acceptability of interventions to increase ART initiation among men who test HIV-positive through index HIV self-testing." 2019-

University of California Los Angeles. David Geffen School of Medicine. Medical Student. Tijana Temelkovska. "Examining the successes and challenges of implementing an early childhood development intervention with HIV-positive women in Malawi: a process evaluation." 2018-

University of California Los Angeles. Internal Medicine Residency, Global Health Track. Resident Physician. Marguerite Thorp. "Can a brief screening tool identify ART clients at risk of defaulting from treatment? a prospective study in Malawi." 2018-

University of California Los Angeles. Internal Medicine Residency, Global Health Track. Resident Physician. Adrian Mayo. "Predictors of early ART retention among adults who initiated under Universal Treatment policies in Malawi." 2018-

University of Colorado Denver. Health and Behavioral Sciences. Doctoral Student. Stephanie Chamberlin. "Exploring the association between education and ART retention in rural Malawi." 2017-

University of California Los Angeles. Fogarty GloCal Fellow. Medical Student. Ogechukwu Offorjebe. "Examining the feasibility and acceptability of HIV self-test kits for index testing among HIV+ clients and their partners in Malawi: A mixed methods study." 2017-18

College of Medicine, Malawi. MPH Student. Khumbo Phiri. "The role of lay cadre in ART initiation and retention under Test and Treat in Malawi." 2017-18

For peer review only

Brandeis University. Elisa Morales, Becca Sliwosk, and Melanie Morris (capstone project). "Developing a funding proposal for Men-to-Men, a gender-transformation and income-generating program for men in northern Uganda." 2015 (with 31Bits International)

Vanguard University. Medical Anthropology Honors Thesis. Joanna Takegami. "Barriers to Women's use of Antiretroviral Therapy in Northern Uganda: Exploring the Role of Structural Violence." 2011

AD HOC REVIEWER

AIDS, JAIDS, JIAS, BMC Public Health, Global Health Action, Culture, Health and Sexuality

PROFESSIONAL MEMBERSHIPS

Member, American Public Health Association (APHA), Present

Member, American Sociology Association (ASA), Present

Member, American Anthropological Association (AAA), Present

For peer review only

OMB No. 0925-0001 and 0925-0002 (Rev. 10/15 Approved Through 10/31/2018)

THOMAS J. COATES

eRA COMMONS USER NAME (credential, e.g., agency login): **TCOATS**

POSITION TITLE: **Professor Emeritus, Division of Infectious Diseases, Department of Medicine UCLA David Geffen School of Medicine**

EDUCATION/TRAINING *(Begin with baccalaureate or other initial professional education, such as nursing, include postdoctoral training and residency training if applicable. Add/delete rows as necessary.)*

INSTITUTION AND LOCATION	DEGREE *(if applicable)*	Completion Date MM/YYYY	FIELD OF STUDY
San Luis Rey College, San Luis Rey, California	BA	06/1968	Philosophy
San Jose State University, San Jose, California	MA	01/1971	Psychology
Stanford University, Stanford, California	PhD	06/1977	Counseling Psychology

A. Personal Statement

I am Director of the system-wide University of California Global Health Institute (founded in 2008) and was the Founding Director of the UCLA Center for World Health (founded in 2012) until 2018. In 1986 I co-founded the Center for AIDS Prevention Studies (CAPS) at UCSF and directed it from 1991 to 2003. I was also the founding Director of the UCSF AIDS Research Institute, leading it from 1996 to 2003.

I have substantial expertise in research on HIV prevention among heterosexual men and women in the HIV epidemic in sub-Saharan Africa and in the HIV testing and treatment trials in sub-Saharan Africa, especially Malawi through PEPFAR funding. As Distinguished Research Professor of Medicine, I continue with two NIH and two foundation grants focused in southern Africa. I also continue as a co-investigator on the UCLA-based Center for HIV Identification, Prevention and Treatment Studies (CHIPTS).

I have had extensive experience with large-scale, community-based, multi-site research and implementation projects spanning HIV prevention, care and treatment, and policy. I currently have funding to test and evaluate innovative strategies for bring men in South Africa into HIV testing and treatment, as well as for providing early childhood development training for HIV-infected mothers and their babies in Malawi through support from the Conrad N. Hilton Foundation. We are also in the first year of a 5-year NIH-funded grant to study pre-exposure prophylaxis for pregnant and post-partum women in South Africa.

B. Positions and Honors

For peer review only

1984 - 2003	Member, Medical Attending Staff, UCSF Hospitals and Clinics
1990 - 2003	Professor, Department of Medicine, UCSF
1991 - 2003	Director, Center for AIDS Prevention Studies, UCSF
1996 - 2003	Director, AIDS Research Institute, UCSF
2000	Elected to the Institute of Medicine (now the National Academy of Medicine)
2010 - 2014	Member, Institute of Medicine Board on Global Health
2003 - 2006	Professor Step VII, Division of Infectious Diseases, Department of Medicine, David Geffen School of Medicine, UCLA
2003 - Present	Joint Appointment, Department of Medicine, UCSF; Member, Executive Committee, UCLA AIDS Institute
2003 - 2011	Director, UCLA Program in Global Health
2004 - Present	Joint Appointment, Department of Epidemiology, UCLA School of Public Health
2006 – 2009	Professor Step IX, Division of Infectious Diseases, Department of Medicine, David Geffen School of Medicine, UCLA
2006 - 2018	Michael & Sue Steinberg Endowed Professor of Global AIDS Research, Division of Infectious Diseases, Department of Medicine, David Geffen School of Medicine, UCLA
2006 - 2018	Director, Global Capacity Building Core Center for HIV Identification, Prevention, and Treatment Services, UCLA Semel Neurosciences Institute
2006 - 2018	Associate Director for International and Policy Research UCLA AIDS Institute
2009 - 2016	Co-director, University of California Global Health Institute
2009 –2018	Distinguished Professor, Division of Infectious Diseases, Department of Medicine, David Geffen School of Medicine, University of California, Los Angeles
2011 – 2018	Director, UCLA Center for World Health at the David Geffen School of Medicine and UCLA Health
2016-Present	Director, University of California Global Health Institute
2018-Present	Distinguished Research Professor, Division of Infectious Diseases, UCLA David Geffen School of Medicine

C. Contribution to Science

1. **Combination HIV Prevention including Pre-Exposure Prophylaxis:** I have written extensively and conducted research on combination HIV prevention for MSM in the United States and Latin America and with a variety of populations in sub-Saharan Africa. My writing and research have been influential in shaping thinking about combination prevention, and in demonstrating the importance of considering HIV prevention as a combination of factors, as opposed to any single kind of program.

Joseph Davey D, Bekker LG, Gorbach P, **Coates T**, Myer L. Delivering PrEP to pregnant and breastfeeding women in sub-Saharan africa: The implementation science frontier. AIDS. 2017 Jul 18. doi: 10.1097/QAD.0000000000001604. PubMed PMID: 28723709.

For peer review only

Richter L, Komárek A, Desmond C, Celentano D, Morin S, Sweat M, Chariyalertsak S, Chingono A, Gray G, Mbwambo J, **Coates T**; Reported physical and sexual abuse in childhood and adult HIV risk behaviour in three African countries: findings from Project Accept (HPTN-043). AIDS and behavior. 2014; 18(2):381-9. PMCID: PMC3796176

Coates TJ; An expanded behavioral paradigm for prevention and treatment of HIV-1 infection. Journal of acquired immune deficiency syndromes (1999). 2013; 63 Suppl 2:S179-82. PMCID: PMC3943341

Coates TJ, Richter L, Caceres C. Behavioural strategies to reduce HIV transmission: how to make them work better. Lancet. 2008; 372(9639):669-84. PMCID: PMC2702246

2. **HIV Counseling and Testing (HTC):** I have conducted many significant and influential studies in HTC, beginning first with observational studies of the effect of HTC on risk behavior among men who have sex with men (MSM) in San Francisco. I was Principal Investigator for the first randomized controlled trial of HTC in Eastern Africa and the Caribbean, examining the effect of HTC on individual males and females, as well as couples presenting for HTC in Kenya, Tanzania, and Trinidad and Tobago, and these results were reported in *The Lancet* in 2000. I was the Principal Investigator for Project Accept, a cluster randomized trial conducted in South Africa, Zimbabwe, Tanzania, and Thailand, and these results were reported in *Lancet Global Health* in 2015. I also was the Principal Investigator of a randomized trial at Mulago Hospital in Uganda examining the effect of short vs. elaborated counseling on males and females presenting for care, and these results were reported in *Lancet Global Health* in 2014.

Coates TJ, Kulich M, Celentano DD, Zelaya CE, Chariyalertsak S, Chingono A, Gray G, Mbwambo JK, Morin SF, Richter L, Sweat M, van Rooyen H, McGrath N, Fiamma A, Laeyendecker O, Piwowar-Manning E, Szekeres G, Donnell D, Eshleman SH; NIMH Project Accept (HPTN 043) study team; Effect of community-based voluntary counselling and testing on HIV incidence and social and behavioural outcomes (NIMH Project Accept; HPTN 043): a cluster-randomised trial. The Lancet. Global Health. 2014; 2(5):e267-77. PMCID: PMC4131207

van Rooyen H1, McGrath N, Chirowodza A, Joseph P, Fiamma A, Gray G, Richter L, **Coates T**. Mobile VCT: reaching men and young people in urban and rural South African pilot studies (NIMH Project Accept, HPTN 043). AIDS and behavior. 2013; 17(9):2946-53. PMCID: PMC3597746

Wanyenze RK, Kamya MR, Fatch R, Mayanja-Kizza H, Baveewo S, Szekeres G, Bangsberg DR, **Coates T**, Hahn JA; Abbreviated HIV counselling and testing and enhanced referral to care in Uganda: a factorial randomised controlled trial. The Lancet. Global Health. 2013; 1(3):e137-45. PMCID: PMC4129546

Mhlongo S, Dietrich J, Otwombe KN, Robertson G, **Coates TJ**, Gray G.Factors associated with not testing for HIV and consistent condom use among men in Soweto, South Africa. PloS one. 2013; 8(5):e62637. PMCID: PMC3656000

3. **Global Health:** I have contributed to the literature on global health, especially from the perspective of engaging multiple disciplinary perspectives to attend to a variety of global health issues around the world.

For peer review only

Debas HT, **Coates TJ**; The University of California Global Health Institute opportunities and challenges. Infectious disease clinics of North America. 2011; 25(3):499-509, vii. PubMed [journal]PMID: 21896355

Duber HC, **Coates TJ**, Szekeras G, Kaji AH, Lewis RJ; Is there an association between PEPFAR funding and improvement in national health indicators in Africa? A retrospective study. Journal of the International AIDS Society. 2010; 13:21. PMCID: PMC2895577

Maman S, Abler L, Parker L, Lane T, Chirowodza A, Ntogwisangu J, Srirak N, Modiba P, Murima O, Fritz K.A comparison of HIV stigma and discrimination in five international sites: the influence of care and treatment resources in high prevalence settings. Social science & medicine (1982). 2009; 68(12):2271-8. PMCID: PMC2696587

Collins C, Coates TJ, Szekeres G; Accountability in the global response to HIV: measuring progress, driving change. AIDS (London, England). 2008; 22 Suppl 2:S105-111. PMCID: PMC2879260

Complete List of Published Work in MyBibliography:
http://www.ncbi.nlm.nih.gov/sites/myncbi/thomas.coates.1/bibliography/40839346/public/?sort=date&direction=descending

D. Research Support

Ongoing Research Support

R01MH105534-01A1 (Coates) 07/07/15 – 04/30/20
NIH/NIMH
Bringing South African Men into HIV Counseling and Testing (HCT) and Care
The objective of this project is to provide evidence-based strategies to improve treatment of HIV+ men through a three-step process: (1) Testing a significant proportion of the population, (2) linkage to care, and (3) maintaining in care a significant proportion of HIV+ individuals to the point of viral suppression. My role is as the Principal Investigator.

UM1 AI068619 (El Sadr) 07/01/14 – 11/30/20
Family Health International
NIH-NIAID
HIV Prevention Trials Network (HPTN) Leadership Group
The goals of this project are: 1) to develop the HPTN research agenda; 2) to review SWG research plans; 3) to review and approve concept plans; 4) to oversee the discretionary fund; 5) to review and revise HPTN policies and procedures; and 6) to evaluate the performance of the HPTN. My role is as Chair of the Manuscript Review Committee

P30 MH058107 (Shoptaw) 03/01/2017-02/28/2022
NIMH/NIH

For peer review only

Center for HIV Identification, Prevention, and Treatment Services

This project is a P30 and provides center grant services to HIV investigators at UCLA. I am a Co-Investigator in this center.

The Conrad N. Hilton Foundation 01/01/2018-12/31/2020

Delivery of Childhood Development Services as Part of HIV Treatment Services in Malawi

This grant supports the integration of early childhood development services within pre- and post-natal care for HIV+ mothers in Malawi.

R01 MH116771-01A1 09/30/2018-09-29-2023

NIMH/NIH

Evaluating the Prep-PP Cascade in HIV-negative Pregnant and Breastfeeding Women in South Africa.

The goal of this project is to test innovative models for delivering PrEP to pregnant and breastfeeding women age 16 and above in South Africa.

Entertainment Industry Foundation-Charlize Theron Africa Outreach Project 06/01/2018-05/30/2021

The goal of this project is to create a Youth Leaders Scholarship Fund to support promising young South Africans to attend South African tertiary education institutions.

Bill and Melinda Gates Foundation **(**Dovel) 12/4/2019-12/3/2023

Identifying Effective Linkage Strategies for HIVST (IDEaL)

This grant tests the effect of a staged intervention for ART initiation among men in Malawi, whereby additional interventions are added each month for individuals who have not yet initiated ART.

Completed Research Support

20150025 (Coates) 09/01/15 – 08/31/17
Conrad N. Hilton Foundation
Delivery of Early Childhood Development Services as a Part of HIV Treatment Services in Malawi
Pilot grant to assess the feasibility and acceptability, as well as initial outcomes, of supporting Option B+ mothers in Malawi to increase their responsiveness to their children and have positive impacts on early childhood development (ECD).

P30 MH58107 (Rotheram-Borus) 02/01/07 - 01/31/17

NIH/NIMH

Center for HIV Identification, Prevention, and Treatment Services (CHIPTS)

The mission of the Center for HIV Identification, Prevention, and Treatment Services (CHIPTS) is to promote collaborative research and education on effective HIV detection, prevention, and treatment programs for HIV at the societal, community, provider, and individual levels. My role is as the Director for International Care.

For peer review only

For peer review only

RISA MICHELLE HOFFMAN

CURRICULUM VITAE

PERSONAL HISTORY

David Geffen School of Medicine at UCLA
Division of Infectious Diseases
10833 Le Conte Ave 37-121 CHS
Los Angeles, CA 90095
rhoffman@mednet.ucla.edu
Tele: (310) 825-7225
Fax: (310) 825-3632

EDUCATION

Stanford University 1994, BA
University of California Los Angeles 2000, MD
Harvard School of Public Health 2000, MPH
Internship 2000-2001: Harvard Combined Medicine/Pediatrics Residency Program
Residency 2001-2004: Harvard Combined Medicine/Pediatrics Residency Program
Fellowship Infectious Diseases: 2005-2008: University of California, Los Angeles

LICENSURE

California, A85173, 01/31/2021

BOARD CERTIFICATION/OTHER CERTIFICATION

2004 & 2014 American Board of Internal Medicine
2007 & 2017 American Board of Internal Medicine, Infectious Diseases
2005 Certification in Travel Medicine from the London School of Hygiene and Tropical Medicine

PROFESSIONAL EXPERIENCE

Present Position

2016-present	Associate Clinical Professor, Division of Infectious Diseases, UCLA
2010-2016	Assistant Clinical Professor, Division of Infectious Diseases, UCLA Medical Center, Los Angeles, California
2008-2010	Clinical Instructor, Division of Infectious Diseases, UCLA Medical Center, Los Angeles, California

Previous Positions

2005-2008	Fellow in Infectious Diseases, UCLA Medical Center, Los Angeles, California
2001-2004	Resident Physician, Internal Medicine, Brigham and Women's Hospital, Boston, Massachusetts
2001-2004	Resident Physician, Pediatrics, Boston Children's Hospital and Massachusetts General Hospital, Boston, Massachusetts
2000-2001	Intern, Internal Medicine, Brigham and Women's Hospital, Boston, Massachusetts
2000-2001	Intern, Pediatrics, Boston Children's Hospital and Massachusetts General Hospital, Boston, Massachusetts

For peer review only

PROFESSIONAL ACTIVITIES & MEMBERSHIPS

2018-present	Interim Director, Global Health Education and Research Program, David Geffen School of Medicine at UCLA
2016-present	Co-Director UCLA AIDS Institute/CFAR International Health Services and Policy Research Program Section
2015-present	Associate Program Director, UCLA Infectious Diseases Fellowship Training Program
2013-present	Advisory Board Member for the University of California Global Health Institute GloCal Health Fellowship
2009-present	Research Co-Director, Partners in Hope Malawi and UCLA Research Collaboration
2009-present	Investigator, AIDS Clinical Trials Group (ACTG) and Maternal Child Adolescent Network (IMPAACT)
2009-present	HIV Clinical Consultant, To Help Everyone Clinic in Los Angeles, California
2009-present	Ad hoc Peer Reviewer (AIDS Care, International Journal of STD and AIDS, BMC Women's Health, American Society of Tropical Medicine and Hygiene, Journal of Infectious Diseases, International Health, JIAS)
2007-present	Member, Infectious Diseases Society of America (IDSA)
2016-2018	Committee Member, Antiretroviral Therapy Strategies (ARTs), AIDS Clinical Trials Group
2008-2016	Founder/Program Director, Sustainable Nutrition for Orphans and Vulnerable Children in Malawi, Central Africa: Provides education on nutrition and sustainable food sources for families caring for orphans in northern Malawi
2014-2016	Committee Lead, Infectious Diseases Quality Improvement M&M Program
2011-2016	Committee Member, AIDS Clinical Trials Group *Women's Health Inter-network Scientific Committee (WHISC)*
2007-2013	Founder/Program Co-Director, UCLA resident physician elective training program in Malawi, Africa
2010-2013	Co-Director, UCLA Program in Global Health and Global Health Education Program for the David Geffen School of Medicine at UCLA
2008-2012	Faculty for 'Multidisciplinary Approach to Global Health' elective course for first and second year medical students at UCLA
2007-2012	Committee Member, American Society of Tropical Medicine and Hygiene Education Committee
2005-2012	Advisory Board Member, UCLA Medicine/Pediatrics Residency Training Advisory Board
2005-2011	Interviewer, UCLA Medicine/Pediatrics Residency Training Program
2006-2008	Faculty Group Leader, Problem Based Learning Microbiology Block for second year medical students at UCLA
2006-2008	Creator/Organizer, UCLA Infectious Diseases Core Curriculum Program

HONORS AND AWARDS

2012	David Geffen School of Medicine Award for Excellence in Education
2011	Nomination for the Consortium of Universities for Global Health Early Career Award
2009	Nomination for UCLA Faculty Teaching Award

For peer review only

2007 Nomination for UCLA Fellow Teaching Award
2006 Nomination for UCLA Fellow Teaching Award

2000 Elected to the UCLA chapter of the Alpha Omega Alpha Honor Society
2000 Janet M. Glasgow Memorial Achievement Citation for Academic Achievement at the UCLA School of Medicine
2000 John M. Adams Award for Excellence in Pediatrics, UCLA School of Medicine
2000 Edith and Carl Lasky Memorial Award for Outstanding Research Achievement, UCLA School of Medicine
1999 Longmire Surgical Medal for outstanding performance in surgical clerkships, awarded by the Department of Surgery, UCLA School of Medicine
1999 Charles Wacker Summer Research Fellowship, UCLA School of Medicine
1994 Elected to the Stanford Chapter of Phi Beta Kappa
1994 Elected to the Stanford Cap and Gown Women's Honor Society
1994 Recipient of the Joshua Lederberg Award for Outstanding Academic Achievement in Human Biology

LECTURES AND PRESENTATIONS

"Management of Febrile Neutropenia" Presented as part of the UCLA Division of Infectious Diseases Core Curriculum Program. Los Angeles, California, November 2013

"Challenges and Successes of EQUIP Malawi" Presented at UCLA Infectious Diseases Grand Rounds. Los Angeles, California, April 2014

"Primary Care Issues in HIV Care" Presented at the UCLA Department of Medicine housestaff curriculum. Los Angeles, California, May 2014

"A Case of Multi-Class HIV Resistance" Presented at the UCLA HIV/Hepatitis C Case Conference Series, Los Angeles, California, May 2014

"Evidence Based Managed of Osteomyelitis" Presented at the UCLA Division of Infectious Diseases Case Conference Series, Los Angeles, California, June 2014

"Responding to Viral Load: A Primer for Malawi Clinical Mentors" Presented at a PEPFAR EQUIP Clinical Training Meeting in Malawi, Africa, January 2015

"Clinical Pearls in the Management of HIV/AIDS". Presented at the UCLA Internal Medicine Resident Core Curriculum Conference Series, Los Angeles, California, February 2015

"Quality Improvement in ID Care at UCLA: Lessons Learned from M&M" Presented at the UCLA Division of Infectious Diseases Core Conference Series, Los Angeles, California, April 2015

For peer review only

“Health & Safety Overseas: An orientation for medical students” Presented at the UCLA Global Health Education Medical Student Orientation Program, Los Angeles, California, April 2015

“ID Mimics”. Presented at the UCLA ID Fellow Core Curriculum Series, Los Angeles, California, May 2015

“Quality Improvement on the Infectious Diseases Service: Transition of Care.” Presented at the UCLA Division of Infectious Diseases Case Conference, Los Angeles, California, June 2015

“Quality Improvement on the Infectious Diseases Service: Notes and Documentation.” Presented at the UCLA Division of Infectious Diseases Case Conference, Los Angeles, California, December 2015

“Quality Improvement on the Infectious Diseases Service: HIV Care”. Presented at the UCLA Division of Infectious Diseases Case Conference, Los Angeles, California, February 2016

“Multi-month scripting to achieve improved outcomes in EQUIP”. Presented at the EQUIP annual meeting, Johannesburg, South Africa March 2016

“Clinical Management of HIV/AIDS for the Primary Care Resident”. Presented at the UCLA Internal Medicine Resident Core Curriculum Conference Series, Los Angeles, California, April 2016

“Update on Option B+ in Malawi”. Presented to the Women’s Health Committee of the AIDS Clinical Trials Group, Los Angeles, California, April 2016

EQUIP Malawi: A Partnership for HIV Care in Malawi. Presented at Harbor UCLA Infectious Diseases Grand Rounds, Los Angeles, California, July 2016

Speaker, Infectious Diseases Career Panel for Medical Students at the David Geffen School of Medicine. Los Angeles, September 2016

Systemwide Case Conference Faculty Discussant for the MultiCampus Infectious Diseases Fellowship Program. Presented at the VA Hospital, Los Angeles, California, December 2016

“Introduction to Global HIV Treatment in Resource Poor Settings,” Lecturer for the UCLA School of Public Health, February 2018, Los Angeles

UCLA Division of Infectious Diseases, Journal Club Faculty Discussant, MDR TB Treatment, March 2018, Los Angeles

For peer review only

Faculty Panelist. Global Health Career Night for the David Geffen School of Medicine. November 2018, Los Angeles

West LA VA Internal Medicine Grand Rounds Speaker: "The Intersection of HIV and Non-Communicable Diseases in Resource-Limited Settings" April 2019, Los Angeles

"Qualitative Client and Provider Experiences with Multi-Dispensing for HIV in Malawi and Zambia". Presented as part of the CQUIN Consortium. Webinar, April 2019

"Introduction to the Global Health Program". Presented as part of the DGSOM Global Health Selective, September 2019, Los Angeles

PUBLICATION/BIBLIOGRAPHY

RESEARCH PAPERS

RESEARCH PAPERS (PEER REVIEWED)

Hoffman RM, Umeh OC, Garris C, Givens N, Currier JS. Evaluation of Sex Differences of Fosamprenavir (With and Without Ritonavir) in HIV-infected Men and Women. HIV Clin Trials. 2007;8(6):371-380.

Hoffman RM, AboulHosn J, Child JS, Pegues DA. Bartonella Endocarditis in Complex Congenital Heart Disease. Congenit Heart Dis. 2007;2(1):79-84.

Black V, **Hoffman RM**, Sugar CA, Menon P, Venter FWD, Currier JS, Rees H. Safety and Efficacy of Initiating Highly Active Antiretroviral Therapy in an Integrated Antenatal and HIV Clinic in Johannesburg, South Africa. J Acquir Immune Defic Syndr. 2008;49(3):276-81. PMC2893046.

Hoffman RM, Black V, Technau K, van der Merwe KJ, Currier JS, Coovadia A, Chersich M. Effects of Highly Active Antiretroviral Therapy Duration and Regimen on Risk for Mother-to-Child Transmission of HIV in Johannesburg, South Africa. J Acquir Immune Defic Syndr. 2010;54(1):35-41. PMC2880466.

Pilotto JH, Velasque L, Khalili R, Ismerio R, Veloso VG, Grinsztejn B, Morgado MG, Watts DH, Currier JS, **Hoffman RM**. Maternal Outcomes after HAART for Prevention of Mother-to-Child Transmission in HIV-infected Women in Brazil. Antivir Ther. 2011;16(3):349-56. PMC3437753.

Hoffman RM, Jamieson BD, Bosch RJ, Currier JS, Kitchen CMR, Schmid I, Zhu Y, Bennett K, Mitsuyasu R. Baseline Immune Phenotypes and CD4+ T Lymphocyte Responses to Antiretroviral

For peer review only

Therapy in Younger versus Older HIV-infected Individuals. J Clin Immunol. 2011;31(5):873-81. PMC3194061.

Van der Merwe J, Hoffman RM, Black V, Chersich M, Coovadia A, Rees H. Birth outcomes in South African Women Receiving Highly Active Antiretroviral Therapy: a Retrospective Observational Study. J Int AIDS Soc. 2011;14:42. PMC3163172.

Mindry D, Wagner G, Lake JE, Smith A, Linnemayr S, Quinn M, **Hoffman RM**. Fertility Desires Among HIV-infected Men and Women in Los Angeles County: Client Needs and Provider Perspectives; Matern Child Health J. 2013 May;17(4):593-600. PMC N/A.

Burke Z, Chen J, Conceicao C, **Hoffman R**, Miller L, Taela A, DeUgarte DA. Evaluation of Preoperative and Intraoperative RBC Transfusion Practices in Maputo Central Hospital, Mozambique. Transfusion. 2013 May 21. doi: 10.1111/trf.12252. PMC3751985.

Hoffman RM, Leister E, Kacanek D, Shapiro DE, Read JS, Bryson Y, Currier JS. Biomarkers from late pregnancy to six weeks postpartum in HIV-infected women who continue versus discontinue antiretroviral therapy after delivery. JAIDS. 2013 May 8. PMC3868443.

Jaganath D, Mulenga C, **Hoffman R**, Hamilton J, Boneh G. This is My Story: Participatory Performance for HIV and AIDS Education at the University of Malawi. Health Education Research. 2013 Sep 18. PMC4155417.

Kawale P, Mindry D, Stramotas S, Chilikoh P, Phoya A, Henry K, Elashoff D, Jansen P, **Hoffman R**. Factors associated with desire for children among HIV-infected women and men: A quantitative and qualitative analysis from Malawi and implications for the delivery of safer conception counseling. AIDS Care. 2013 Jun;26(6). PMC3943633.

1. Iroezi N, Mindry D, Kawale P, Chikowi G, Jansen P, **Hoffman R**. A qualitative analysis of the barriers and facilitators to receiving care in a prevention of mother-to-child program in Nkhoma, Malawi. Afr JReprod Health. 2013 Dec;17(4). PMC4361063.

2. Russell E, Mohammed T, Smeaton L, Jorowe B, MacLeod I, **Hoffman R**, Currier JS, Moyo S, Essex M, Lockman S. Immune activation markers in peripartum women in Botswana: association with feeding strategy and maternal morbidity. PLoS One. 2014 Mar 21. PMC3962339.

3. Reddy D, Njala J, Stocker P, Schooley A, Flores M, Tseng C-H, Pfaff C, Jansen P, Mitsuyasu RT, **Hoffman RM**. High-risk human papillomavirus in HIV-infected women undergoing cervical cancer screening in Lilongwe, Malawi: A pilot study. International Journal of STDS and AIDS, 2014 Jun 13. PMC4363075.

4. Kamuyango A, Hirschhorn L, Wang W, Jansen P, **Hoffman R**. One-Year Outcomes of Women Started on Antiretroviral Therapy during Pregnancy before and after the Implementation of Option B+ in Malawi: A Retrospective Chart Review from Three Facilities. World Journal of AIDS. 2014 Sept;4(3). PMC4356991.

5. Shull H, Tymchuk C, Grogan T, Hamilton J, Friedman J, **Hoffman RM**. Evaluation of the UCLA Department of Medicine Malawi Global Health Clinical Elective: Lessons from the First Five Years. Am J Trop Med Hyg. 2014 Sep 15. PMC4228879.

For peer review only

6. Hoffman JC, Anton PA, Baldwin GC, Elliott J, Anisman-Posner D, Tanner K, Grogan T, Elashoff D, Sugar C, Yang OO, **Hoffman RM**. Seminal Plasma HIV-1 RNA Concentration is Strongly Associated with Altered Levels of Seminal Plasma Interferon Gamma, Interleukin-17, and Interleukin-5. AIDS Res Hum Retroviruses. 2014 Oct 2. PMC4208556.

7. Menon P, **Hoffman R**, Black V. Characteristics of HIV-infected women on antiretroviral therapy who develop preeclampsia in South Africa: A Case Series. The Journal of Global Health. 2014;4(2). PMC N/A.

8. Cheng Q, Engelage E, Grogan T, Currier JS, and **Hoffman RM**. Who provides primary care? A cross-sectional survey of HIV patients and providers in a Los Angeles clinic. AIDS Clinical Research. 27 Oct 2014;5(11). PMC4409003.

9. Kawale P, Mindry D, Phoya A, Jansen P**, Hoffman RM**. Provider attitudes about childbearing and knowledge of safer conception at two HIV clinics in Malawi. Reproductive Health. 2015 Mar 7;12(1):17. PMC4355153.

10. Yeatman S, **Hoffman RM,** Chilungo A, Lungu S, Namadingo H, Chimwaza A, and Trinitapoli JA. Health-seeking behavior and symptoms associated with early HIV infection: Results from a population-based cohort in southern Malawi. JAIDS. 2015 May 1;69(1):126-30. PMC4422188.

11. **Hoffman RM**, Jaycocks A, Vardavas V, Wagner G, Lake JE, Mindry D, Currier JS, Landovitz R. Benefits of PrEP as an adjunctive method of HIV prevention during attempted conception between HIV-uninfected women and HIV-infected male partners. Journal of Infectious Diseases, J Infect Dis. 2015 Jun 19. PMC4621256.

12. Lake JE, **Hoffman RM**, Tseng CH, Wilhalme HM, Currier JS. Success of Standard Dose Vitamin D Supplementation in Treated HIV Infection. Open Forum Infect Dis. 2015 May 15;2(2). PMC4462892.

13. Chipungu C, Veltman JA, Jansen P, Chiliko P, Lossa C, Namarika D, Benner B, **Hoffman RM**, Bristow CC, Klausner JD. Feasibility and Acceptability of Cryptococcal Antigen Screening and Prevalence of Cryptocococcemia in Patients Attending a Resource-limited HIV/AIDS clinic in Malawi. Journal of the International Association of Providers of AIDS Care, J Int Assoc Provid AIDS Care. 2015 Jul 2. PMC N/A.

14. Coelho L, Cardoso SW, Luz PM, **Hoffman RM**, Mendonca L, Veloso VG, Currier JS, Grinsztejn B, Lake JE. Vitamin D3 supplementation in HIV infection: effectiveness and associations with antiretroviral therapy. Nutr J. 2015 Aug 18: 14. PMC4538921.

15. **Hoffman RM**, Lake JE, Wilhame H, Tseng CH, Currier JD. Vitamin D levels and markers of inflammation and metabolism in HIV-infected individuals on suppressive antiretroviral therapy. AIDS Res and Human Retroviruses 2016 March;32(3). PMC4779972.

16. Herbst de Cortina S, Arora G, Wells T, **Hoffman RM**. Evaluation of a Structured Predeparture Orientation at the David Geffen School of Medicine's Global Health Education Programs. Am J Trop Med Hyg. 2016 Mar 2;94(3). PMC4775891.

For peer review only

17. Jordan J, **Hoffman R**, Arora G, Coates W. Activated learning: providing structure in global health education at the David Geffen School of Medicine at the University of California, Los Angeles – a pilot study. BMC Medical Education. 2016 Feb 16. PMC4755030.

18. Wagner GJ, Linnemayr S, Ghosh-Dastidar B, Currier JD, **Hoffman R**, Schneider S. Supporting Treatment Adherence Readiness through Training (START) for patients with HIV on antiretroviral therapy: a study protocol for a randomized controlled trial. Trials. 2016 March 24;17(1). PMC4806419.

19. Arora G, Perkins K, **Hoffman RM**. Optimizing global health electives through partnerships: A pilot study of pediatric residents. Academic Pediatrics. 2015 Sept-Oct;15(5). PMC N/A.

20. Chien E, Phiri K, Schooley S, Chivwala M, Hamilton J, **Hoffman RM**. Successes and Challenges of HIV Mentoring in Malawi: The Mentee Perspective. PLoS One. 2016 Jun 18;11(6). PMC4924818.

21. Schooley A, Kamudumuli P, Vangala S, Tseng CH, Soko C, Parent J, Phiri K, Jahn A, Namarika D, **Hoffman RM**. CD4 Variability in Malawi: Implications for Use of a CD4 Threshold of 500 Cells/mm3 Versus Universal Eligibility for Antiretroviral Therapy. Open Forum Infectious Diseases. 2016 Aug 29;3(3). PMC5047419.

22. Hemarajata P, Baghdadi JD, **Hoffman R**, Humphries RM. Burkholderia pseudomallei: Challenges for the Clinical Microbiology Laboratory. J Clin Microbiol 2016 Dec;54(12):2866-2873. PMC5121373.

23. Pfaff C, Scott V, **Hoffman R**, Mwagomba B. You can treat my HIV – But can you treat my blood pressure? Availability of integrated HIV and non-communicable disease care in northern Malawi. Afr J Prim Health Care Fam Med. 2017 Feb 15;9(1):e1-e8. PMC5320467.

24. Gibb J, Chitsulo J, Chipungu C, Chivwara M, Schooley A, **Hoffman RM**. Supporting Quality Data Systems: Lessons Learned from Early Implementation of Routine Viral Load Monitoring at a Large Clinic in Lilongwe, Malawi. Clinical Research in HIV AIDS and Prevention. 2017 Mar 14;2(4). PMC5502771

25. Arora G and **Hoffman RM**. Development of an HIV Postexposure Prophylaxis (PEP) Protocol for Trainees Engaging in Academic Global Health Experiences. Acad Med. 2017 Apr 25. PMC28445222.

26. **Hoffman RM**, Phiri K, Parent J, Grotts J, Elashoff D, Kawale P, Yeatman S, Currier JS, Schooley A. Factors associated with retention in Option B+ in Malawi: a case control study. J Int AIDS Soc. 2017 Apr 27;20(1):1-8. PMC28453243.

27. Currier JS, Britto P, **Hoffman RM**, Brummel S, Masheto G, Joao E, Santos B, Aurpibul L, Losso M, Pierre MF, Weinberg A, Gnanashanmugam D, Chakhtoura N, Klingman K, Browning R, Coletti A, Mofenson L, Shapiro D, Pilotto J. Randomized Trial of Stopping or Continuing ART among Postpartum Women with Pre-ART CD4 ≥ 400 cells/mm3 (PROMISE 1077HS). PLoS One. 2017 May 10;12(5):e0176009. PMC28489856.

28. **Hoffman R**, Bardon A, Rosen S, Fox M, Kalua T, Xulu T, Taylor A, Sanne I. Varying intervals of antiretroviral medication dispensing to improve outcomes for HIV patients (The INTERVAL

For peer review only

Study): study protocol for a randomized controlled trial. Trials. 2017 Oct 13;18(1):476. PMC29029644.

29. Judson SD, LeBreton M, Fuller T, **Hoffman RM**, Njabo K, Brewer TF, Dibongue E, Diffo J, Kameni JF, Loul S, Nchinda GW, Njouom R, Nwobegahay J, Takuo JM, Torimiro JN, Wade A, Smith TB. Translating Predictions of Zoonotic Viruses for Policymakers. Ecohealth. 2017 Dec 11. PMC29230614.

30. Fatti G, Ngorima-Mabhena N, Chirowa F, Chirwa B, Takarinda K, Tafuma TA, Mahachi N, Chikodzore R, Nyadundu S, Ajayi CA, Mutasa-Apollo T, Mugurungi O, Mothibi E, **Hoffman RM**, Grimwood A. The effectiveness and cost-effectiveness of 3- vs. 6-monthly dispensing of antiretroviral treatment (ART) for stable HIV patients in community ART-refill groups in Zimbabwe: study protocol for a pragmatic, cluster-randomized trial. Trials. 2018 Jan 29;19(1):79. PMC29378662.

31. Caplan MR, Phiri K, Parent J, Phoya A, Schooley A, **Hoffman RM**. Provider Perspectives on Barriers to Reproductive Health Services for HIV-Infected Clients in Central Malawi. Clinical Obstetrics, Gynecology and Reproductive Medicine. 2018 Feb 12;4(1). PMC6391881.

32. Canan T, **Hoffman RM**, Schooley A, Boas Z, Schwab K, Kahn D, Shih R, Phiri K, Parent J, Banda BA, Chagoma R, Chipungu C, Pool K-L. Training Course in Focused Assessment with Sonography for HIV/TB in HIV Prevalent Medical Centers in Malawi. Journal of Global Radiology. 2018 March;4(1). PMC N/A.

33. Rough K, Seage GR, Williams PL, Hernandez-Diaz S, Huo Y, Chadwick EG, Currier JS, **Hoffman RM**, Barr E, Shapiro DE, Patel K; PHACS and the IMPAACT P1025 Study Teams. Birth Outcomes for Pregnant Women with HIV Using Tenofovir–Emtricitabine. New England Journal of Medicine. 2018 April 26;378(17):1593-1603. PMC5984044

34. Dovel K, Shaba F, Nyirenda M, Offorjebe OA, Balakasi K, Phiri K, Nichols B, Tseng C-H, Bardon A, Ngona K, **Hoffman R**. Evaluating the Integration of HIV Self-Testing Into Low-Resource Health Systems: Study Protocol for a Cluster-Randomized Control Trial from EQUIP Innovations. Trials. 2018 Sept 17;19(1). PMC6142354

35. Schwab K, **Hoffman RM**, Phiri L, Kahn D, Longwe L, Banda BA, Gama K, Chimombo M, Shih R, Schooley A, Pool K-L. Remote Training and Oversight of Sonography for Human Immunodeficiency Virus–Associated Tuberculosis in Malawi. Journal of the American College of Radiology. 2018 Sept 26. PMC6540803

36. Hubbard J, Kakwesa G, Nyirenda M, Mwambene J, Bardon A, Balakasi K, Dovel K, Kalua T, **Hoffman RM**. Towards the Third 90: Improving Viral Load Testing with a Simple Quality Improvement Program in Health Facilities in Malawi. Int Health. 2018 Nov 1. PMC Pending

37. Moucheraud C, Chasweka D, Nyirenda M, Schooley A, Dovel K, **Hoffman RM**. Simple Screening Tool to Help Identify High-Risk Children for Targeted HIV Testing in Malawian Inpatient Wards. J Acquir Immune Def Syndr. 2018 Nov 1; 79(3). PMC Pending

38. **Hoffman RM**, Brummel SS, Britto P, Pilotto JH, Masheto G, Aurpibul L, Joao E, Purswani MU, Buschur S, Flore Pierre M, Coletti A, Chakhtoura N, Klingman KL, Currier JS for the 1077HS

PROMISE Team. Adverse Pregnancy Outcomes among Women who Conceive on Antiretroviral Therapy. Clin Infect Dis 2018 June 1. PMC6321847

39. Nhassengo P, Cataldo F, Magaco A, **Hoffman RM**, Nerua L, Saide M, Cuco R, Hoek R, Mbofana F, Couto A, Gudo E, Chicumbe S, Dovel K. Barriers and facilitators to the uptake of Test and Treat in Mozambique: a qualitative study on patient and provider perceptions. PLOS One. 2018 Dec 26; 13(12). PMC6306230

40. Magaço A, Dovel K, Cataldo F, Nhassengo P, **Hoffman RM**, Nerua L, Tique J, Saide M, Couto A, Mbofana F, Gudo E, Cuco R, Chicumbe S. 'Good health' as a barrier and facilitator to ART initiation: a qualitative study in the era of test-and-treat in Mozambique. Culture, Health and Sexuality. 2018. PMC Pending

41. **Hoffman RM**, Konstantia A, Brummel S, Saidi F, Violari A, Dula D, Mave V, Fairlie L, Gerhard T, Kamateeka M, Chipato T, Chi B, Stranix-Chibanda L, Nematadzira T, Moodley D, Bhattacharya D, Gupta A, Coletti A, McIntyre JA, Klingman KL, Chakhtoura N, Shapiro DE, Folwer MG, Currier JS. Maternal health outcomes among HIV-infected breastfeeding women with high CD4 counts: results of a treatment strategy trial. HIV Clinical Trials 2018 Dec;19 (6). PMC6428202 [Available on 2019-12-01]

42. Murnane PM, Bacchetti P, Currier JS, Brummel S, Okochi H, Phung N, Louie A, Kuncze K, **Hoffman RM**, Nematadzira T, Soko DK, Owor M, Saidi F, Flynn PM, Fowler MG, Gandhi M. Tenofovir concentrations in hair strongly predict virologic suppression in breastfeeding women. AIDS 2019 Apr 22. [Epub ahead of print]. PMC Pending

43. Shaba F, Offorjebe OA, Phiri K, Lungu E, Kalande, P, Nyirenda M, Gupta S, **Hoffman RM**, Dovel K. Perceived acceptability of a facility-based HIV self-test intervention in outpatient waiting spaces among adult outpatients in Malawi: A formative study. J Acquir Immune Defic Syndr. 2019 Jul 1;81(3):e92-e94. PMC Pending

44. Weinberg A, Huo Y, Kacanek D, Patel K, Watts DH, Wara D, **Hoffman RM**, Klawitter J, Christians U; for IMPAACT P1025 Team. Markers of spontaneous preterm delivery in women living with HIV: relationship with protease inhibitors and Vitamin D. J Acquir Immune Defic Syndr. 2019 May 28. [Epub ahead of print]. PMC Pending

45. McBride K, Parent J, Mmanga K, Chivwala M, Nyirenda MH, Schooley A, Mwambene JB, Dovel K, Lungu E, Balakasi K, **Hoffman RM**, Moucheraud C. ART Adherence Among Malawian Youth Enrolled in Teen Clubs: A Retrospective Chart Review. AIDS Behav. 2019 July 10. PMC Pending

46. Le C, Britto P, Brummel SS, **Hoffman RM**, Li JZ, Flynn PM, Taha TE, Coletti A, Fowler MG, Bosch RJ, Gandhi RT, Klingman KL, McIntyre JA, Currier JS. Time to Viral Rebound and Safety after Antiretroviral Treatment Interruption in Postpartum Women Compared to Men. In Press AIDS 2019, August 1. PMC Pending

For peer review only

OMB No. 0925-0001 and 0925-0002 (Rev. 09/17 Approved Through 03/31/2020)

MICHAL KULICH

eRA COMMONS USER NAME (credential, e.g., agency login):

POSITION TITLE: Associate Professor of Statistics

EDUCATION/TRAINING *(Begin with baccalaureate or other initial professional education, such as nursing, include postdoctoral training and residency training if applicable. Add/delete rows as necessary.)*

INSTITUTION AND LOCATION	DEGREE *(if applicable)*	Completion Date MM/YYYY	FIELD OF STUDY
Charles University, Prague	M.S.	9/1991	Math. Statistics
Limburgs Universitair Centrum, Diepenbeek	M.S.	9/1992	Biostatistics
University of Washington, Seattle	M.S.	9/1995	Biostatistics
University of Washington, Seattle	Ph.D.	10/1997	Biostatistics

A. Personal Statement

I have an extensive past experience with design, conduct and analysis of clinical trials, especially community randomized trials, in the context of HIV prevention research. I served as the Lead Statistician for the Behavioral Working Group within the HPTN in 2000–2003 and as the Protocol Statistician and Steering Committee member for Project ACCEPT (HPTN043) in 2003-2013. I have been also participating in protocol review groups in the HPTN. I was involved in the design of HPTN043, development of data collection procedures, and development and application of data quality control measures. I am a coauthor of 7 research papers on methodology and results of HPTN043. Since 2015, I am a protocol statstician on another community-randomized trial, Zwakala Ndoda Study: Diagnosing, Linking and Maintaining Men in Antiretroviral Treatment in Vulindlela and Greater Edendale Area, KwaZulu-Natal. The current application builds on my past experience with HIV prevention trials.

B. Positions and Honors

Professional Positions

For peer review only

1998–2000 Dept. of Probability and Statistics, Charles University, Prague, Czech Rep., Assistant Professor
2000–2003 Dept. of Biostatistics, University of Washington, Seattle, Research Assistant Professor
2004–2009 Dept. of Probability and Statistics, Charles University, Prague, Czech Rep., Assistant Professor
2010–2013 Dept. of Probability and Statistics, Charles University, Prague, Czech Rep., Associate Professor
2014– Dept. of Probability and Statistics, Charles University, Prague, Czech Rep., Chair

Professional Memberships

1995- Member, American Statistical Association

1998- Member, Czech Statistical Society

2004- Member, International Biometric Society

2005- Member, International Society for Clinical Biostatistics

Honors

1995 Donovan J. Thompson Award, University of Washington, Seattle, WA.

1997 Best Written Paper, International Biometric Society, Park City, UT.

2001 Bolzano Award, Bernard Bolzano Foundation, Prague, Czech Republic.

C. Contributions to Science

Design and conduct of HIV prevention trials

I have an expertise in design, conduct and analysis of large randomized HIV prevention trials. I was a protocol statistician in Project ACCEPT (HPTN043), a community-randomized trial conducted in five African and Asian sites, with HIV incidence calculated from cross-sectional blood samples as the primary endpoint. I designed methods for obtaining population samples by household-probability sampling, participated in data verification, performed analyses and collaborated on publications.

Genberg, B., **Kulich, M.,** Kawichai, S., Modiba, P., Chingono, A., Kilonzo, G., Richter, L., Pettifor, A., Sweat, M. & Celentano, D. HIV risk behaviors in Sub-Saharan Africa and Northern Thailand: Baseline behavioral data from Project Accept. *Journal of AIDS* 2008, 49(3):309-319. PMID: 18845954

Sweat, M., Morin, S., Celentano, D., Mulawa, M., Singh, B., Mbwambo, J., Kawichai, S., Chingono, A., Khumalo-Sakutukwa, G., Gray, G., Richter, L., **Kulich, M.,** Sadowski, A., Coates, T., and the Project Accept study team. Community-based intervention to increase HIV testing and case detection in people aged 16-32 years in Tanzania, Zimbabwe, and Thailand (NIMH Project Accept, HPTN 043): a randomised study. *The Lancet Infectious Diseases* 2011, 11(7), 525-532. PMID: 21546309

Coates, T.J., **Kulich, M.**, Celentano, D.D., Zelaya, C.E., Chariyalertsak, S., Chingono, A., Gray, G., Mbwambo, J.K.K., Morin, S.F., Richter, L., Sweat, M., van Rooyen, H., McGrath, N., Fiamma, A.,

For peer review only - http://bmjopen.bmj.com/site/about/guidelines.xhtml

Laeyendecker, O., Piwowar-Manning, E., Szekeres, G., Donnell, D., Eshleman, S.H. (2014) Effect of community-based voluntary counselling and testing on HIV incidence and social and behavioural outcomes (NIMH Project Accept; HPTN 043): A cluster-randomised trial. *The Lancet Global Health* 2014, 2 (5), e267-e277. PMID: 25103167

Salazar-Austin, N., **Kulich, M.**, Chingono, A., Chariyalertsak, S., Srithanaviboonchai, K., Gray, G., Richter, L., van Rooyen, H., Morin, S., Sweat, M., Mbwambo, J., Szekeres, G., Coates, T., Celentano, D. (2017) Age-Related Differences in Socio-Demographic and Behavioral Determinants of HIV Testing and Counseling in HPTN 043/NIMH Project Accept. *AIDS and Behavior* 2018, 22(2) 569-579. PMID: 28589504

Methods for cross-sectional incidence estimation

I participated in the development of laboratory and statistical methods for estimating HIV incidence from cross-sectional blood samples. These methods were needed for successful evaluation of the primary outcome in Project ACCEPT.

Laeyendecker, O., Piwowar-Manning, E., Fiamma, A., **Kulich, M.**, Donnell, D., Bassuk, D., Mullis, C. E., Chin, C., Swanson, P., Hackett, Jr, J., Clarke, W., Marzinke, M., Szekeres, G., Gray, G., Richter, L., Alexandre, M. W., Chariyalertsak, S., Chingono, A., Celentano, D. D., Morin, S. F., Sweat, M., Coates, T., Eshleman, S. H. Estimation of HIV Incidence in a Large, Community-Based, Randomized Clinical Trial: NIMH Project Accept (HIV Prevention Trials Network 043), *PLoS ONE* 2013, 8:7, e68349. PMID: 23874597

Laeyendecker, O., **Kulich, M.**, Donnell, D., Komárek, A., Omelka, M., Mullis, C. E., Szekeres, G., Piwowar-Manning, E., Fiamma, A., Gray, R. H., Lutalo, T., Morrison, C. S., Salata, R. A., Chipato, T., Celum, C., Kahle, E. M., Taha, T. E., Kumwenda, N. I., Karim, Q. A., Naranbhai, V., Lingappa, J. R., Sweat, M. D., Coates, T., Eshleman, S. H. Development of Methods for Cross-Sectional HIV Incidence Estimation in a Large, Community Randomized Trial. *PLoS ONE* 2013, 8:11, e78818. PMID: 2423605

Fogel, J.M., Piwowar-Manning, E., Donohue, K., Cummings, V., Marzinke, M.A., Clarke, W., Breaud, A., Fiamma, A., Donnell, D., **Kulich, M.**, Mbwambo, J., Richter, L., Gray, G., Sweat, M., Coates, T., Eshleman, S. Determination of HIV status in African adults with discordant HIV rapid tests. *Journal of Acquired Immune Deficiency Syndromes* 2015, 69, 430-438. PMID: 25835607

Fogel, J.M., Clarke, W., **Kulich, M.**, Piwowar-Manning, E., Breaud, A., Olson, M.T., Marzinke, M.A., Laeyendecker O., Fiamma, A., Donnell, D., Mbwambo, J., Richter, L., Gray, G., Sweat, M., Coates, T.J., Eshleman, S.H. Antiretroviral drug use in a cross-sectional population survey in Africa: NIMH Project Accept (HPTN 043). *Journal of Acquired Immune Deficiency Syndromes* 2017, 74, 158-165. PMID: 27828875

For peer review only

AUGUSTINE T CHOKO

Institution: Malawi Liverpool Wellcome Trust Clinical Research Programme

General Medical Council (or equivalent) registration number N/A

Do you currently have personal medical malpractice insurance? (if so, name of insurer)

N/A

Project role

Principal Investigator

Qualifications

Degree	Year	Subject	Awarding Institution
PhD	2018	Epidemiology	LSHTM
MSc	2012	Epidemiology	LSHTM
BSc	2009	Statistics & Computing	University of Malawi

Positions held (last ten years)

Start	End	Organisation	Position title, brief description of responsibilities
**2020**	**2024**	Malawi Liverpool Wellcome Trust (MLW)	Wellcome Trust & National Institute for Health Research International Intermediate Fellow
2019	2020	MLW	Protocol Lead; leading design, implementation and write up of a complex primary health clinic randomized trial.
2015	2018	MLW	Wellcome Trust Fellow in Public Health and Tropical Medicine<br>▪ PhD student

For peer review only

2013	2015	MLW	Research Assistant (Epidemiology)<br>▪ Data analysis and publication
2012	2013	MLW	Trial Manager<br>▪ Leading implementation of a community-based cluster randomized trial (HIV/TB)
2009	2012	MLW	Data Manager/Statistician<br>▪ Designing and administering study databases<br>▪ Preparing data for analysis and data analysis

GCP training: course provider, date

NIDA Clinical Trials Network, Obtained 11 November 2016, expires 2019

Please list any institutions with which you are affiliated to

Malawi Liverpool Wellcome Trust Clinical Research Programme, London School of Hygiene & Tropical Medicine

Ethics training: details of training, course provider, date

Clinical trials experience: title of trial, role, dates (if multiple, restrict to most recent and most relevant)

Secondary distribution of HIV self-tests through antenatal and HIV testing services: a pragmatic cluster-randomized trial (STAR-ANC); 2018-2019.

Partner-Provided Self-Testing and Linkage (PASTAL) adaptive multi-arm multi-stage cluster randomized trial; 2016-2017.

Intensified HIV / TB prevention linking home-based HIV testing, including the option of self-testing, with HIV care: a cluster-randomised trial in Blantyre, Malaw, Research Fellow, 2011-2015.

Other professional experience relevant to role in project

Experience in handling and analyzing large epidemiological datasets.

Relevant recent publications (maximum 5)

1. **Choko AT**, Corbett EL, Stallard N, Maheswaran H, Lepine A, Johnson CC, Sakala D, Kalua T, Kumwenda M, Hayes R, Fielding K. Effect of HIV self-testing alone or with additional

For peer review only

interventions including financial incentives on linkage to care or prevention among male partners of antenatal care attendees in Malawi: An adaptive multi-arm multi-stage cluster randomised trial. *PLoS Med* 2019 Jan 2;16(1):e1002719.

2. **Choko AT**, Fielding K, Stallard N, et al. Investigating interventions to increase uptake of HIV testing and linkage into care or prevention for male partners of pregnant women in antenatal clinics in Blantyre, Malawi: study protocol for a cluster randomised trial. *Trials.* 2017;18(1):349.
3. **Choko AT**, Kumwenda MK, Johnson CC, et al. Acceptability of woman-delivered HIV self-testing to the male partner, and additional interventions: a qualitative study of antenatal care participants in Malawi. *Journal of the International AIDS Society.* 2017;20(1):21610.
4. **Choko AT**, MacPherson P, Webb EL, et al. Uptake, Accuracy, Safety, and Linkage into Care over Two Years of Promoting Annual Self-Testing for HIV in Blantyre, Malawi: A Community-Based Prospective Study. *PLoS medicine.* 2015;12(9):e1001873.
5. **Choko AT**, Desmond N, Webb EL, et al. The uptake and accuracy of oral kits for HIV self-testing in high HIV prevalence setting: a cross-sectional feasibility study in Blantyre, Malawi. *PLoS medicine.* 2011;8(10):e1001102.

For peer review only

Khumbo Phiri Nyirenda

CONTACTS
Partners in Hope
PO Box 302, Lilongwe**.**
Cell: 265882400721/ 265999 840 946
Email: khumbophiri@gmail.com

ACADEMIC QUALIFICATIONS

I. MPH, University of Malawi, College of medicine, anticipating graduation in 2020
II. BSOC, Economics, University of Malawi, Chancellor College- February, 2006
III. Malawi School Certificate of Education (MSCE) Phwezi Girls Sec School-June, 2000

COURSES/ TRAININGS

- Qualitative research synthesis, university of cape town, faculty of health sciences February 2020
- Qualitative data analysis university of cape town, faculty of health sciences January 2020
- Biomedical Researchers and Staff modules (CITI program _May 2016)
- Certificate of Attendance in Value chain analysis training by Ron Black from CNFA's farmer to farmer USAID funded program, Washington DC (February 23-27, 2009)
- Output marketing training in grain grading by CNFA/RUMARK facilitated by a North Carolina Agriculture Department officer, held at Natural Resources College (August 25 -28, 2009).
- Certificate of Attendance in a Leadership workshop facilitated by Engineers without Boarders (October 26 – 28, 2009).
- Training of trainers course in Business management and technical knowledge by COMESA's ACTESA and IFDC in Lusaka, Zambia (September 6-15, 2010)

WORK EXPERIENCE

PARTNERS IN HOPE
Position: Implementation Science Manager
Period: September 2017 to date
Summary:
The Projects Research Coordinator is responsible for overseeing and implementing all research related activities at Partners in Hope (PIH) and in all program-supported sites. He/she is in charge of monitoring and evaluating projects and ensuring that PIH is accountable to research donors. This person works hand-in-hand with the University of California in Los Angeles (UCLA), Partners in Hope (PIH), the Ministry of Health (MoH) and other partners.
Responsibilities

For peer review only

1. Overseeing and implementing all research projects at Partners in Hope and all EQUIP-supported sites

2. Research applications, Reviews and Reports.

- Oversee applications for ethical review for the Malawi NHSRC and/or COMREC, including initial and renewal applications, as well as closeout of completed projects.
- Serve as the first line of direct communication with the NHSRC and/or COMREC to advocate for submitted applications.
- Work with UCLA, PIH, MoH and other partners to ensure all research is performed to the highest ethical standards and that data is securely managed.
- Make sure appropriate reporting is provided to the governing bodies (final reports, publications, etc.).
- Oversee submission of abstracts to research meetings.

3. Monitoring, Evaluation and Accountability to Donors

- Monitor all research projects and develop donor communications in collaboration with senior leadership, especially the M&E Team.
- Ensure timely production and submission of donor experts.
- Participate in development of strategies for expansion of research.
- Ensure continuous evaluation of projects and staff, including hiring and regular appraisals.

Period: December 2012 to March 2016
Organization: Partners in Hope
Position: Research coordinator

Description

- Coordinates and administers research study associated activities. Assists in project planning and ensures that pre-established work scope, study protocol and regulatory (ethical review in Malawi and at UCLA) requirements are followed. Oversees and coordinates research staff. Develops and maintains record keeping systems and procedures. My job as Research Coordinator involves these main tasks
- Assistance developing research proposals, data collection forms, and spreadsheets for organization of data. Develops and maintains record keeping systems and procedures.

- Ensures the smooth and efficient day to day operation of research and data collection
- activities; acts as the primary administrative point of contact for EQUIP research staff.
- Supervision of team of research assistants

- Assistance with recruitment and coordination of research subjects as appropriate.

- Supervision and assistance with quality control Data.

- Monitors the progress of research activities; develops and maintains records of research activities and prepares periodic and ad hoc reports as required by investigators, administrators and funding agencies(USAID quarterly reports) and regulatory bodies (NHSRC,UCLA,IRB)

For peer review only

- Assistance with preparing ethical review applications (HSRC) including frequent communication with NHSRC about status of pending applications.

I. CARANA COOPERATION

Position: M&E/MIS Assistant
Period: November 2010–September 2011

Description
Market Linkages Initiative was a project funded by **USAID** and implemented by ACDI/VOCA and CARANA Corporation. The two key objectives of the project are to strengthen and expand grain bulking systems and to integrate farmers to national and regional markets. My job as an M&E/MIS Specialist involved these main tasks:

- Assisting the M&E specialist in tracking MLI indicators, collecting and verifying information and maintaining PMP reports, work plans and reports(weekly, monthly updates, quarterly and annual) for Malawi activities

- Administering data collection tools to GBC/VACs and capacity building of grantees to keep relevant records and generate M&E reports as stipulated by the Grant agreement.

- Collating , analyzing and reporting in usable forms all data collected form GBC/VAC

- Supporting in the coming up of GIS map for MLI supported GBCs and its associated VACs in Malawi

- Administering M&E data collection tools and supervising M&E data collectors and ensure quality data collection

- Maintaining records of all source documents from grantees and other sources including filled questionnaires and interview reports
- Keeping records of field trip reports and monitor and updating field trip tracker for Malawi based MLI staff
- Undertaking case studies and documenting most significant change stories for selected GBCs/VACs/Farmers to monitor impacts of MLI work

- Maintaining an up to date filing system including project photos

- Ensuring that quality control procedures are met in terms of market data.

- Facilitating dissemination of market information to farmers on a timely and reliable basis using the E-platform

- Providing technical assistance on the E- platform to strategic partners

- Working alongside the new company and assisting/participating in development and deployment team to design and roll out a web to phone MIS platform

For peer review only

- Conducting weekly data checks on approved prices inside E-platform's price flagging module

- Manage a user, market and commodities database

II. **CNFA/RUMARK**

Position: Monitoring and Evaluation Coordinator
Period: January 2009 to October 2010

CNFA/RUMARK implemented the Malawi Agrodealer Strengthening Program funded by AGRA. Its main objective was to develop rural-based, commercially-viable agrodealer networks and to work with agrodealers to improve the management, technical and financial capacity of their enterprises, thereby creating a rural market driven economic environment specifically designed to meet the unique needs of smallholder farmers. My position of as M&E coordinator involved the following tasks

- Monitoring progress of the project activities by doing surveys which included development of survey tools which mostly use participatory methods.

- Monitoring and evaluating the agrodealers performance in terms of sales as well as their financial status.

- Analysis of information on Agrodealer performance

- Verifying and identifying operational and potential agrodealers and recommending them for training to enable them get registered with CNFA

- Organizing promotional activities i.e. lottery competitions with the intention of creating customer database for surveys
- Playing a facilitating role in managing relationships between RUMARK and input supply companies to ensure cordial relationships and partnerships.
- Consolidating and analyzing results across CNFA's programs.

- Conducting training needs assessment for different categories of agrodealers to ensure equal treatment so that their specific needs are taken on board.

- Organizing the Agrodealers Annual Convention.

- Production of monthly as well as interim semi-annual reports for the Project

- Involved in advocating for policies which are conducive for agrodealers' business growth and sustainability through Private–public partnerships which involves working with various stakeholders including Government and civil society organizations.

Research Abstracts
Provider perspectives on barriers to reproductive health services for HIV-infected clients

For peer review only

in Central Malawi: Khumbo Phiri, Margaret R Caplan , Julie Parent , Ann Phoya , Alan Schooley, and Risa M. Hoffman, Poster presentation at Interest 2017, Malawi

Barriers to ART uptake experienced by healthy clients in Malawi under Test and Treat: Dovel, Kathryn, Khumbo Phiri, Alan Schooley, McDaphton Bellos, Esnart Sanudi, Denis Chasweka, Risa Hoffman, poster exhibition at the 9th IAS Conference on HIV Science (IAS 2017, in Paris, France, 23-26 July 2017 and Interest 2017 in Malawi).

Facility-level barriers to antiretroviral therapy experienced by men in Malawi: Dovel, Kathryn, Khumbo Phiri, Alan Schooley, Misheck Mphande, Mackenzie Chivwara, Risa Hoffman(poster presentation at interest 2017, Malawi)

Examining Malawi's Rollout of Universal Treatment: Policy Implementation and Provider Perceptions: Misheck Mphande, Khumbo Phiri, Mackenzie Chivwara, Mike Nyirenda, Alan Schooley, Rachel Thomas, Risa Hoffman, Kathryn Dovel, (Poster presentation at IAS 2017 in Paris, France)

Low rates of successful defaulter tracing and re-engagement in care in Option B+ women in Central Malawi. K. Phiri, J. Parent, T. Mulitswa, A. Schooley, R. Hoffman. Poster presentation at the International AIDS Society (IAS) conference (Durban, 2016).

The successes and Challenges of collaborating with Health Surveillance Assistants (HSAs) to trace Option B+ defaulters. Khumbo Phiri Nyirenda, Julie Parent, Risa Hoffman, Alan Schooley, Temwanani Mulitswa

The Option B+ cascade: Characterizing uptake and retention in a USAID-PEPFAR program in rural Malawi. Khumbo Phiri, Alan Schooley, Mackenzie Chivwala, Joseph Njala, Judy Currier, Andreas Jahn, Anteneh Worku, Perry Jansen, Risa Hoffman

Improvements and on-going challenges in exposed infants care at rural sites in Malawi. Alan Schooley, Khumbo Phiri, Mackenzie Chivwala, Peter Chilikoh, Antenneh worku, Risa Hoffman

Health Surveillance Assistants Can Successfully Perform Defaulter Tracing In Rural Malawi. Mackenzie Chivwala, Khumbo Phiri, Risa Hoffman, Jimmy Chitsulo, Alan Schooley

Assessing the Potential Impact of Health Surveillance Assistants on HIV Care At The Facility And Community Level. Mackenzie Chivwala, Khumbo Phiri, Weston Njamwaha, Peter Chilikoh, Risa Hoffman, Alan Schooley

Mentee Perspectives on Factors Associated with a Successful HIV Mentorship Program Mike Nyrienda, Chiulemu Kussen, Savior Mwandira, Khumbo Phiri, Chiukepo Longwe, Peter Chilikoh, Risa Hoffman, Weston Njamwaha, Alan Schooley

Rapid Rollout of Viral Load Testing at Rural Health Facilities in Malawi. Alan Schooley, Risa Hoffman, Mike Nyirenda, Savior Mwandira, Weston Njamwaha, Khumbo Phiri, Chifundo Chipungu, Mackenzie Chivwala, James Kandulu

Increased HIV testing after implementation of an innovative CD4 results reporting system in rural Malawi. Alan Schooley, Mackenzie Chivwala, Reynier Ter Haar, George Mtonga,

For peer review only

Doreen Suwande, Kelvin Rambiki, Chiulemu Kussen, John Hamilton, Khumbo Phiri, Peter Chilikoh, Risa Hoffman, Perry Jansen. Accepted for poster presentation at the 6th South African AIDS Conference, Durban, South Africa, 18-21 June 2013.

Barriers to Adherence to ART in the Prevention of Mother-to-Child Transmission of HIV: Option B+ in Nkhoma, Malawi. Paul Kawale, Alan Schooley, Virginia Tancioco, Danielle Wickman, Khumbo Phiri, Ella Bwanausi, Risa Hoffman. Accepted for poster presentation at the 6th South African AIDS Conference, Durban, South Africa, 18-21 June 2013.

MANUSCRIPTS ACCEPTED/PUBLISHED

Successes and Challenges of HIV Mentoring in Malawi: The Mentee Perspective. E. Chien, K. Phiri, A. Schooley, M. Chivwala, J. Hamilton, R. Hoffman. PLoS One. 2016 Jun;11(6).

CD4 variability in Malawian adults and implications for universal eligibility. A.L. Schooley, P.S. Kamudumuli, S. Vangala, C.H. Tseng, C. Soko, J. Parent, K. Phiri, A. Jahn, D. Namarika, R. Hoffman. Open Forum Infect Dis. 2016 Aug;3(3).

Provider perspectives on barriers to reproductive health services for HIV-infected clients in Central Malawi: Margaret R Caplan, Khumbo Phiri, Julie Parent, Ann Phoya , Alan Schooley, and Risa M. Hoffman, PLOS ONE.

Factors Associated with Retention in Option B+ in Malawi: A Case Control Study: Risa M. Hoffman, khumbo phiri, Julie parent, J Grotts D Elashoff, Paul Kawale, Sara Yeatman, J S Currier, A Schooley, JIAS.

Training Course in Focused Assessment with Sonography for HIV/TB in HIV Prevalent Medical Centers in Malawi: Timothy Canan, R Hoffman, Alan Schooley, Zachary Boas, Kristin Schwab, Daniel Kahn, Roger Shih, Khumbo Phiri, Julie Parent, Ben Allan Banda, Ronald Chagoma, Chifundo Chipungu. Kara-Lee Pool, Journal of Global Radiology

REFEREES

Risa Hoffman (MD), Assistant Clinical Professor**,** David Gaffen school of medicine, UCLA**,** RHoffman@mednet.ucla.edu

Rachel Sibande (PhD), Program Director, United Nations Foundation rsibande@unfoundation.org, +27670236497

Godfrey Chapola (PhD), Managing Director, RUMARK, P.O Box 31290, Lilongwe.

Cel: 0999792 070, gchapola@rumark.org

For peer review only

Julie Hubbard

94 Culford Road London N1 4HN
+44 7845 445338
jhubbard@mednet.ucla.edu

EDUCATION

London School of Hygiene and Tropical Medicine
MSc Control of Infectious Diseases

Seattle Pacific University
Bachelor of Arts: Sociology & Women's Studies
Cum Laude GPA: 3.74
Graduated July 2012

PROFESSIONAL EXPERIENCE

University of California Los Angeles (UCLA), March 2017- Current
Research Coordinator – 'INTERVAL' Study
Lilongwe, Malawi and Lusaka, Zambia

- Supervise data collection by study personnel across 15 health facilities in southern and central Malawi. Coordinate field supervision to ensure data quality. Work with Principle Investigator (PI) to develop operating procedures for study implementation. Provide leadership and technical support to Zambia study team.

Harvest India USA, January 2016 – March 2017
Director of Operations
Costa Mesa, California and Andhrah Pradesh, India

- Managed all aspects of operations to support, fundraise, and raise awareness for education and poverty alleviation initiatives amongst the Dalit, or 'untouchable', caste. Drafted and executed marketing campaigns to meet fundraising goals.

31 Bits International, December 2012 – February 2015
Director of Operations
Gulu, Northern Uganda

- Directed 160 beneficiaries and 6 Ugandan counselors in income generating projects. Developed and implemented in-depth monthly reports to evaluate income. Used data to identify hindrances to livelihood, such as domestic violence and HIV health complications. Organized necessary support through internal management or accessing external resources.

One Days Wages, March 2011- December 2012
Chief Grant Analyst
Seattle, Washington

- Generated extensive research on project proposals pertaining to the UN Millennium Development Goals and presented analyses for grant decisions.

Seattle Pacific University, September 2011- July 2012
Research Assistant
Seattle, Washington

- Edited, reviewed, and prepared research documents for Assistant Director of Women's Studies Program.

PUBLICATIONS AND PRESENTATIONS

For peer review only

Publications
Julie Hubbard, Gift Kakwesa, Mike Nyirenda, James Mwambene, Ashley Bardon, Kelvin Balakasi, Kathryn Dovel, Thokozani Kalua, Risa M Hoffman; Towards the third 90: improving viral load testing with a simple quality improvement program in health facilities in Malawi, International Health, , ihy083, https://doi.org/10.1093/inthealth/ihy083

Hubbard J, Moucheraud C, Lungu E, Bardon A, Balakasi K, Kakwesa G, Hoffman R ""I forget that I am a patient": A qualitative assessment of 6 month dispensing of ART" (Under review)

Dovel K, Beagley M, Hubbard J, Orombi G, Thompson K "Including men without sidelining women: the feasibility of male involvement within a women's empowerment program in northern Uganda" (Under review)

Dovel K, Hubbard J, Phiri K. "Gender and HIV services: The role of gender norms on ART initiation among men and women in Malawi." (In preparation)

Peer reviewed poster presentations
"Gender and HIV services: The role of gender norms on ART initiation among men and women in Malawi." Women in Global Health Scientific Conference. New York, New York. April 2018

"Towards the third 90: improving viral load testing with a simple quality improvement program in health facilities in Malawi" International Aids Society (IAS) Conference, Amsterdam, Netherlands. July 2018

Presentations
"Innovations in differentiated service delivery: Six-month scripting lessons from Ethiopia, Malawi and Zambia" Colombia University Mailman School of Public Health. Webinar, April 2019

CERTIFICATIONS
Confronting Gender Based Violence: Global Lessons with Case Studies from India Certification Course
Coursera (Johns Hopkins University) - Online
October 2015

- Epidemiology of gender-based violence, clinical care issues and how to provide psychosocial support for victims.

FELLOWSHIP
Mennonite Central Committee
Community Development Associate, July-August 2011

- Rural and urban poverty field study in Recife, Brazil association under the direction of the Chair of the Sociology Department at Seattle Pacific University.

HONORS
Seattle Pacific University Deans Scholar, 2008-2012

For peer review only

BROOKE E. NICHOLS, PHD, MS

OFFICE ADDRESS:

801 Massachusetts Avenue

Crosstown Center, 3rd Floor, Room 304

Boston, MA, USA 02118

+1 617 358 2403

Email: brooken@bu.edu

ACADEMIC TRAINING:

2015	Ph.D.	Department of Viroscience, Erasmus Medical Center (Rotterdam, the Netherlands)<br>*Mathematical Modeling and Cost-Effectiveness of Antiretroviral-Based HIV-1 Prevention Strategies.*
2011	M.S.	School of Public Health & Health Science, University of Massachusetts, Amherst<br>(Amherst, MA, USA), Epidemiology
2009	B.A.	Mount Holyoke College (South Hadley, MA, USA), International Relations, *cum laude*

RESEARCH APPOINTMENTS:

2019 – Present	Assistant Professor	Department of Global Health, School of Public Health,<br>Boston University, Boston, MA
2018 – 2019	Instructor	Department of Global Health, School of Public Health,<br>Boston University, Boston, MA
2017 – 2018	Research Scientist	Department of Global Health, School of Public Health,

For peer review only

		Boston University, Boston, MA
2017 – Present	Principal Researcher	Health Economics & Epidemiology Research Office, Wits
		Health Consortium, Faculty of Health Sciences, University of
		Witwatersrand, Johannesburg, South Africa
2017 – Present	Researcher	Joint Faculty Appointment, School of Clinical Medicine,
		Faculty of Health Sciences, University of the Witwatersrand,
		Johannesburg, South Africa
2015 – 2016	Postdoctoral Fellow	Department of Viroscience, Erasmus Medical Center,
		Rotterdam, the Netherlands
2009 – 2010	Research Assistant	University of Massachusetts Amherst, School of Public Health
		& Health Sciences, Amherst, MA

OTHER RESEARCH EXPERIENCE:

2012 – 2014	Epidemiologist	Médicins Sans Frontières
		Amsterdam, the Netherlands.
		Project: *Spinal cord injury outcomes in Sri Lanka*
2008 – 2009	Researcher	Ministry of Health and Social Services, Lüderitz, Namibia
		Project: *Ecologic study on alcohol establishments and HIV prevalence*

PROFESSIONAL APPOINTMENTS:

2008 – 2010	Research Associate: Epidemiology	Environ Corporation, Amherst, MA, USA.

AWARDS AND HONOURS:

2019	**Mary Lyon Award.** Mount Holyoke College Alumni Association. Award honors an alumna who

For peer review only

has demonstrated sustained achievement in her life and career consistent with the humane values

that Mary Lyon exemplified and inspired in others.

CONFERENCE ORAL PRESENTATIONS:

*Denotes graduate student or mentee

1. Popping S*, Kall M, Stempher E, Versteegh L, **Nichols B**, van Sighem A, van de Vijver D, Boucher C, Verbon A, Delpech V. Country specific factors determine the quality of life among people with HIV in two western European countries.: 4th European Workshop on Health Living with HIV, Barcelona, Spain, September 2019.

2. Dovel K, Balakasi K, Shaba F, Offorjebe O, Gupta S, Wong S, Phiri K, Lungu E, Nyirenda M, **Nichols B**, Ngona K, Hoffman R. A randomized trial on index HIV self-testing for partners of ART clients in Malawi. Conference on Retroviruses and Opportunistic Infections (CROI), Seattle, USA, March 2019.

3. **Nichols BE**, Girdwood SJ*, Crompton T, Stewart-Isherwood L, Berrie L, Chimhamhiwa D, Moyo C, Kuehnle J, Rosen S. Monitoring viral load for the last mile: what will it cost? AIDS, Amsterdam, Netherlands, July 2018.

4. Girdwood SJ*, **Nichols BE**, Moyo C, Crompton T, Chimhamhiwa D, Rosen S. Optimizing access for the last mile: Geospatial cost model for point of care viral load instrument placement in Zambia. AIDS, Amsterdam, Netherlands, July 2018.

5. Dovel K, Nyirenda M, Shaba F, Offorjebe OA, Balakasi K, **Nichols BE**, Phiri K, Schooley A, Hoffman RM. Facility-based HIV self-testing for outpatients dramatically increases HIV testing in Malawi: a cluster randomized trial. AIDS, Amsterdam, Netherlands, July 2018.

6. **Nichols BE**, Hendrickson C, Sigwebela N, Moyo C, Fox MP, Rosen S. Prioritizing healthcare facilities for on-site mentorship to increase HIV treatment uptake: results from EQUIP. International AIDS Economics Network (IAEN) Conference, Amsterdam, Netherlands, July 2018.

7. van de Vijver DA, Richter A-K, Boucher CA, Gunsenheimer-Bartmeyer B, Kollan C, **Nichols BE**, Spinner C, Wasem J, Schewe K, Neumann A. Cost-effectiveness of pre-exposure prophylaxis in Germany (Kosteneffektivität der HIV-Präexpositionsprophylaxe in Deutschland). DGGÖ (German Society for health economics) Annual Meeting, Hamburg, Germay, March 2018.

8. van de Vijver DA, Richter A-K, Boucher CA, Gunsenheimer-Bartmeyer B, Kollan C, **Nichols BE**, Spinner C, Wasem J, Schewe K, Neumann A. Cost-effectiveness of pre-exposure prophylaxis for HIV-1 prevention in Germany. European AIDS Conference (EACS), Milan, Italy, October 2017.

9. Smit M, van Zoest RA, **Nichols BE**, Vaartjes I, Smit C, van der Valk M, van Sighem A, Wit FW, Hallett TB, Reiss P. Cardiovascular prevention policy in HIV: recommendations from a modeling study. Conference on Retroviruses and Opportunistic Infections (CROI), Seattle, WA. February 2017.

10. Popping S*, **Nichols BE**, van Kampen JJA, Verbon A, Boucher CAB, van de Vijver DA. Intensive hepatitis C monitoring in previously HCV infected HIV-positive MSM is a cost saving method to

For peer review only

reduce the HCV epidemic. Netherlands Conference on HIV Pathogenesis, Epidemiology, Prevention and Treatment (NCHIV), Amsterdam, the Netherlands, November 2016.

11. **Nichols BE**, Boucher CAB, van der Valk M, Rijnders BJA, van de Vijver DA. PrEP is Only Cost-Effective Among MSM in the Netherlands When Used on Demand. Conference on Retroviruses and Opportunistic Infections (CROI), Boston, MA. February 2016.

12. **Nichols BE**, Boucher CAB, van der Valk M, Rijnders BJA, van de Vijver DA. On demand PrEP among MSM in the Netherlands: a cost-effective approach for preventing HIV-1 infections. Netherlands Conference on HIV

Peer reviewed publications:

*Authors contributed equally

**Denotes graduate student or mentee

1. Dovel K, Nyirenda M, Shaba F, Offorjebe OA, Balakaksi K, **Nichols BE**, Cele R. Phiri K, Wong V, Gupta S, Hoffman RM. Effect of facility-based HIV self-testing on uptake of testing among adult outpatients in Malawi: a cluster-randomized trial. *The Lancet Global Health. In press.*

2. van Vliet MM**, Hendrickson C**, **Nichols BE**, Boucher CAB, Peters RPH, Polis CB, van de Vijver DAMC. Epidemiological impact and cost-effectiveness of long-acting pre-exposure prophylaxis combined with injectable contraceptives for HIV prevention in South Africa: a modelling study. *JIAS*. 2019, 22:e25427.

3. Long, L., Kuchukhidze, S., Pascoe, S., **Nichols, B.,** Cele R., Govathson, C., Flynn, D., Rosen, S. Differentiated Models of Service Delivery for Antiretroviral Treatment of HIV in sub-Saharan Africa: A Rapid Review Protocol. *Systematic Reviews*. 2019, 8:314.

4. Masuku S**, Berhanu R, van Rensburg C, Ndjeka N, Rosen S, Long L, Evans D, **Nichols BE**. The costs of managing multi drug-resistant tuberculosis in South Africa: an economic evaluation of moving to a short-course treatment regimen containing bedaquiline. *International Journal of Tuberculosis and Lung Disease. In press.*

5. Hendrickson C*,**, Long L*, van de Vijver DA, Boucher CA, O'Bra H, Claassen CW, Njelesani M, Moyo C, Mumba DB, Subedar H, Mulenga L, Rosen S, **Nichols BE**. Novel metric for evaluating PrEP program effectiveness in real-world settings. *Lancet HIV. In press.*

6. Girdwood SJ**, **Nichols BE,** Moyo C, Crompton T, Chimhamhiwa D, Rosen S. Optimizing access for the last mile: Geospatial cost model for point of care viral load instrument placement. *PLoS ONE*. 14(8):e0221586.

7. **Nichols BE**, Girdwood SJ**, Crompton T, Stewart-Isherwood L, Berrie L, Chimhamhiwa D, Moyo C, Kuehnle J, Stevens W, Rosen S. Monitoring viral load for the last mile: what will it cost? *JIAS*. 2019, 22:e25337.

8. Popping S**, **Nichols BE**, van Kampen JJA, Verbon A, Boucher CAB, van de Vijver DA. Targeted HCV core antigen monitoring among HIV-positive men-who-have-sex-with-men is cost-saving. *Journal of Virus Eradication*. 2019; 5:179-190.

For peer review only

9. **Nichols BE**, Girdwood SJ**, Shibemba A, Sikota S, Gill C, Mwananyanda L, Scott L, Noble L, Carmona S, Rosen S, Stevens W. Cost and impact of dried blood spot versus plasma separation card for viral load testing in resource limited settings. *Clinical Infectious Diseases.* Advance article: 10.1093/cid/ciz338.

10. van de Vijver DA, Richter A-K, Boucher CA, Gusenheimer-Bartmeyer B, Kollan C, **Nichols BE**, Spinner CD, Wasem J, Schewe K, Neumann A. Cost-effectiveness and budget impact of generic pre-exposure prophylaxis for HIV-1 prevention in Germany. *Eurosurveillance.* 2019 Feb; 24(7).

11. Popping S**, Hulligie SJ, Boerekamps A, Rijnders BJA, de Knegt RJ, Rockstroh JK, Verbon A, Boucher CAB, **Nichols BE**, van de Vijver DA. Early treatment of acute HCV infection is cost-effective in HIV-infected men-who-have-sex-with-men. *PLoS One*, 2019. 14(1):e0210179.

12. **Nichols BE**, Girdwood SJ**, Crompton T, Stewart-Isherwood L, Berrie L, Chimhamhiwa D, Moyo C, Kuehnle J, Stevens W, Rosen S. Impact of a borderless sample transport network for scaling up viral load monitoring: results of a geospatial optimization model for Zambia. *JIAS.* 2018, 21:e25206.

13. Dovel K, Shaba F, Nyirenda M, Ogechukwu AO, Balakasi K, Phiri K, **Nichols BE**, Tseng C-H, Bardon A, Namachapa KN, Hoffman RM. Evaluating the integration of HIV self-testing into low-resource health systems: a study protocol for a cluster randomized control trial from EQUIP Innovations. *Trials,* 2018. 19:498.

14. Phillips AN, Cambiano V, Nakagawa F, Revill P, Jordan MR, Hallett TB, Doherty M, De Luca A, Lundgren JD, Mhangara M, Apollo T, Mellors J, **Nichols B**, Parikh U, Pillay D, Rinke de Wit T, Sigaloff K, Havlir D, Kuritzkes DR, Pozniak A, van de Vijver D, Vitoria M, Wainberg MA, Raizes E, Bertagnolio S, Working Group on Modelling Potential Responses to High Levels of Pre-ART Drug Resistance in Sub-Saharan Africa. Cost-effectiveness of public-health policy options in the presence of pretreatment NNRTI drug resistance in sub-Saharan: a modelling study. *Lancet HIV*, 2018. 5(3):e146-e154.

15. Smit M, van Zoest RA, **Nichols BE**, Vaartjes I, Smit C, van der Valk M, van Sighem A, Wit FW, Hallett TB, Reiss P; Netherlands ATHENA observational HIV cohort. Cardiovascular disease prevention policy in HIV: recommendations from a modelling study. *Clinical Infectious Diseases*, 2018. 66(5):743-750.

16. Luiken GPM, Joore IK, Taselaar A, Schuit SCE, Geerlings SE, Govers A, Rood PPM, Prins JM, **Nichols BE**, Verbon A, de Vries-Sluijs TEMS. Non-targeted HIV screening in emergency departments in the Netherlands. *The Netherlands Journal of Medicine*, 2017. 75(9):386-393.

17. **Nichols BE**, Boucher CAB, van der Valk M, Rijnders BJA, van de Vijver DAMC. Cost-effectiveness analysis of pre-exposure prophylaxis for HIV-1 prevention in the Netherlands: a mathematical modelling study. *Lancet Infectious Diseases*, 2016. 16(12):1423-1429.

18. **Working Group on Modelling of ART Monitoring Strategies in Sub-Saharan Africa**. Sustainable HIV Treatment in Africa through Viral Load-Informed Differentiated Care. *Nature*, 2015. 528(7580):S68-76.

19. **Nichols BE**, Gotz HM, van Gorp ECM, Verbon A, Rokx C, Boucher CAB, van de Vijver DAMC. Partner notification for reduction of HIV-1 transmission and related costs among men who have sex with men: a mathematical modeling study. *PLoS One,* 2015. 10(11):e0142576.

For peer review only

20. **Nichols BE**, Sigaloff KC, Kityo C, Hamers RL, Baltussen R, Bertagnolio S, Jordan MR, Hallett TB, Boucher CA, Rinke de Wit TF, van de Vijver DA. Increasing the use of second-line therapy is a cost-effective approach to prevent the spread of drug-resistant HIV: a mathematical modelling study. *J Int AIDS Soc*, 2014. 17:19164.

21. **Nichols BE**, Baltussen R, van Dijk JH, Thuma PE, Nouwen JL, Boucher CA, van de Vijver DA. Cost-effectiveness of PrEP in HIV/AIDS control in Zambia: a stochastic league approach. *JAIDS*, 2014. 66(2):221-8.

22. Eaton JW, Menzies NA, Stover J, Cambiano V, Chindelevitch L, Cori A, Hontelez JAC, Humair S, Kerr CC, Klein DJ, Mishra S, Mitchell KM, **Nichols BE**, Vickerman P, Bakker R, Barnighausen T… Hallett TB. How should HIV programmes respond to evidence for the benefit of earlier treatment initiation? A combined analysis of twelve mathematical models. *Lancet Global Health*, 2014. 2:e23-34.

23. Armstrong JC*, **Nichols BE***, Wilson JM, Cosico RA, Shanks L. Spinal cord injury in the emergency context: review of program outcomes of a spinal cord injury rehabilitation program in Sri Lanka. *Conflict and Health*, 2014. 8(1):4.

24. **Nichols BE,** Sigaloff KC, Kityo C, Mandaliya K, Hamers RL, Bertagnolio S, Jordan MR, Boucher CA, Rinke de Wit TF, van de Vijver DA. Averted HIV infections due to expanded antiretroviral treatment eligibility offsets risk of transmitted drug resistance: A modeling study. *AIDS*, 2014. 28(1):73-83.

25. van de Vijver DA, **Nichols BE,** Abbas UL, Boucher CA, Cambiano V, Eaton JW, Glaubius R, Lythgoe K, Mellors J, Phillips A, Sigaloff KC, Hallett TB. Pre-Exposure Prophylaxis will have a limited impact on HIV-1 drug resistance in sub-Saharan Africa: A comparison of mathematical models. *AIDS*, 2013. 27(18):2943-2951.

For peer review only

Alemayehu Amberbir

POSITION TITLE: Epidemiologist
eRA Commons User Name: A.AMBERBIR_DI

EDUCATION/TRAINING

INSTITUTION AND LOCATION	DEGREE *(if applicable)*	YEAR(s)	FIELD OF STUDY
Haramaya University, Ethiopia	BSc	2004	Health Officer
Jimma University, Ethiopia	MPH	2007	Public Health
University of Nottingham, UK	PhD	2012	Epidemiology

Positions and Honors

Sept 2003 – Jun 2004 Intern (Clinical/Public Health), Haramaya University & Hiwot Fana Hospital, Ethiopia Oct 2004 – Sept 2005 HIV/AIDS Prevention and Care Program Officer, Menschen Für Menschen, Ethiopia
Aug 2007 - Mar 2008 Lecturer, Department of Epidemiology and Biostatistics, Jimma University, Ethiopia
Feb 2008 – Mar 2011 Honorary Lecturer, Addis Ababa University, Ethiopia
Feb 2008 – Feb 2012 Research Fellow, University of Nottingham, UK
Mar 2012 – Sep 2013 Research Fellow, London School of Hygiene and Tropical Medicine, UK
Oct 2013 – Jan 2016 Lecturer, London School of Hygiene and Tropical Medicine, UK
Jan 2016 – Jun 2019 Epidemiologist, Dignitas International
Jan 2018 – Jun 2019 Adjunct Lecturer, Dalla Lana School of Public Health, University of Toronto
Jan 2018 – Dec 2019 Postdoctoral Fellow; CIHR Canadian HIV Trials Network (CTN), Canada
Aug 2019 – present Science Director, University of California Los Angles; David Geffen School of Medicine

Contribution to Science

Investigating non-communicable diseases (hypertension, diabetes and asthma) in Africa (selected)

1. Soares ALG, Banda L, **Amberbir A**, Jaffar S, Musicha C, Price A, Nyirenda MJ, Lawlor DA, Crampin A. Sex and area differences in the association between adiposity and lipid profile in Malawi. *BMJ Glob Health*. 2019 Sep 11;4(5):e001542. doi: 10.1136/bmjgh-2019-001542.

2. **Amberbir A.** The challenge of worldwide tuberculosis control: and then came diabetes. Lancet GlobHealth. 2019 Apr;7(4):e390-e391. doi: 10.1016/S2214-109X(19)30053-1.

3. **Amberbir A**, S Lin, J Berman, et al A Muula, D Jacoby, E Wroe et al. Systematic review of hypertension and diabetes burden and risk factors and interventions for prevention and control in

Malawi: The NCD BRITE Consortium. Glob Heart. 2019 Jun;14(2):149-154. doi: 10.1016/j.gheart.2019.05.004. Review.

4. **Amberbir A**, Banda V, Singano V, Matengeni A, Pfaff C, Ismail Z, Allain TJ, Chan AK, Sodhi SK, van Oosterhout JJ. Effect of cardio-metabolic risk factors on all-cause mortality among HIV patients on antiretroviral therapy in Malawi: A prospective cohort study. ***PLoS ONE***. 2019 Jan 17;14(1):e0210629. doi: 10.1371/journal.pone.0210629. PMID: 30653539

5. **Amberbir A**, Singano V, Matengeni A, Ismail Z, Kawalazira G, Chan AK, et al. Dyslipidemia among rural and urban HIV patients in south-east Malawi. PLoS ONE 2018; 13(5): e0197728. https://doi.org/10.1371/journal.pone.0197728

6. Price AJ, Crampin AC, **Amberbir A**, et al. Prevalence of obesity, hypertension and diabetes, and cascade of care in sub-Saharan Africa: a cross-sectional population-based study in rural and urban Malawi. ***Lancet Diabetes Endocrinol 2018***; published online Jan 19. http://dx.doi.org/10.1016/S2213-8587 (17)30432-1.

7. *The GBD 2015 Obesity Collaborators et al **[inc Amberbir A].** Health Effects of Overweight and Obesity in 195 Countries over 25 Years. *N Engl J Med 2017*;377(1):13-27.

8. Divala OH, **Amberbir A**, Ismail Z, Beyene T, Garone D, Pfaff C, Singano V, Akello H, Joshua M, Nyirenda MJ, Matengeni A, Berman J, Mallewa J, Chinomba GS, Kayange N, Allain TJ, Chan AK, Sodhi SK, van Oosterhout JJ. The burden of hypertension, diabetes mellitus, and cardiovascular risk factors among adult Malawians in HIV care: consequences. *BMC Public Health*. 2016 Dec 12;16(1):1243. PMID:27955664

9. Crampin AC, Kayuni N, **Amberbir A**, Musicha C, Koole O, Tafatatha T, Branson K, Saul J, Mwaiyeghele E, Nkhwazi L, Phiri A, Price AJ, Mwagomba B, Mwansambo C, Jaffar S, Nyirenda MJ. Hypertension and Diabetes in Africa: Design and implementation of a large population-based study of burden and risk factors in rural and urban Malawi. *Emerging Themes in Epidemiology*. 2016, 13:3. DOI: 10.1186/s12982-015-0039-2

10. Jaffar S, **Amberbir A**, Kayuni N, Musicha C, Nyirenda M. Viewpoint: scaling up testing services for noncommunicable diseases in Africa: priorities for implementation research. Trop Med Int Health. 2013Nov;18(11):1353-6. doi: 10.1111/tmi.12180. Epub 2013 Sep

11. **Amberbir A**, Medhin G, Hanlon C, Britton J, Davey G, Venn A. Effects of early life paracetamol use on the incidence of allergic disease and sensitization: 5 year follow-up of an Ethiopian birth cohort. *PLoS ONE*. **2014** Apr 9;9(4):e93869. doi: 10.1371/journal.pone.0093869.

12. **Amberbir A**, Medhin G, Abegaz WE, Hanlon C, Robinson K, Fogarty A, Britton J, Venn A, Davey G. Exposure to Helicobacter pylori infection in early childhood and the risk of allergic disease and atopic sensitization: a longitudinal birth cohort study. Clin Exp Allergy. 2014 Apr;44(4):563-71. doi:

13. **Amberbir A**, Medhin G, Erku W, Alem A, Simms R, Robinson K, Fogarty A, Britton J, Venn A, Davey G. Effects of Helicobacter pylori, geohelminth infection and selected commensal bacteria on the risk of allergic disease and sensitization in 3-year-old Ethiopian children. *Clin Exp Allergy*. **2011** Oct;41(10):1422-30. doi: 10.1111/j.1365-2222.2011.03831.x. Epub 2011 Aug 10.

For peer review only

14. **Amberbir A**, Medhin G, Alem A, Britton J, Davey G, Venn A. The role of acetaminophen and geohelminth infection on the incidence of wheeze and eczema: a longitudinal birth-cohort study. *Am J Respir Crit Care Med*. **2011** Jan 15;183(2):165-70. doi: 10.1164/rccm.201006-0989OC. Epub 2010 Oct 8.

HIV risk behaviours and status disclosure in African settings (selected)

1) Dessalegn NG, Hailemichael RG, Shewa-Amare A, Sawleshwarkar S, Lodebo B, **Amberbir A**, Hillman RJ. HIV Disclosure: HIV-positive status disclosure to sexual partners among individuals receiving HIV care in Addis Ababa, Ethiopia. PLoS One**. 2019** Feb 15;14(2):e0211967. doi: 10.1371/journal.pone.0211967. eCollection 2019.

2) Deribe K, Woldemichael K, Wondafrash M, Haile A, **Amberbir A**. Disclosure experience and associated factors among HIV positive men and women clinical service users in Southwest Ethiopia. *BMC Public Health*. 2008 Feb 29;8:81. doi: 10.1186/1471-2458-8-81.

3) Biadgilign S, Deribew A, **Amberbir A**, Escudero HR, Deribe K. Factors associated with HIV/AIDS diagnostic disclosure to HIV infected children receiving HAART: a multi-center study in Addis Ababa, Ethiopia. *PLoS ONE*. 2011 Mar 21;6(3):e17572. doi: 10.1371/journal.pone.0017572.

Health system research in to infectious diseases – HIV – in Africa (selected)

1. Alhaj M[1,2¶],**Amberbir A**[1¶], Singogo E, Banda V, van Lettow M, Matengeni A, Kawalazira G, Theu J, Jagriti MR, Chan AK, van Oosterhout JJ. Retention on antiretroviral therapy during universal test and treat implementation in Zomba district, Malawi: a retrospective cohort study. J Int AIDS Soc. 2019 Feb;22(2):e25239. doi: 10.1002/jia2.25239.

2. Singano V[¶], **Amberbir A**[¶], Garone D, Kandionamaso C, Msonko J; van Lettow M, Kalima K, Mataka, Kawalazira G, Mateyu G, Kwekwesa A, Matengeni A; van Oosterhout JJ. The burden of gynecomastia among men on Antiretroviral Therapy in Zomba, Malawi. *PLoS ONE 12(11): e0188379: doi.org/10.1371/journal.pone.0188379*

3. Mpawa H, Kwekwesa A, **Amberbir A**, Garone D, Divala OH, Kawalazira G, van Schoor V , Ndindi H, van Oosterhout JJ. Virological outcomes of Antiretroviral Therapy in Zomba Central Prison, Malawi: *J Int AIDS Soc* 2017, 20:21623

4. **Amberbir A**, Woldemichael K, Getachew S, Girma B, Deribe K. Predictors of adherence to antiretroviral therapy among HIV-infected persons: a prospective study in Southwest Ethiopia. *BMC Public Health***. 2008** Jul 30;8:265. doi: 10.1186/1471-2458-8-265.

5. Biadgilign S, Deribew A, **Amberbir A**, Deribe K. Barriers and facilitators to antiretroviral medication adherence among HIV-infected paediatric patients in Ethiopia: A qualitative study. *SAHARA J***.** 2009 Dec;6(4):148-54.

For peer review only

6. Deribe K, Hailekiros F, Biadgilign S, **Amberbir A**, Beyene BK. Defaulters from antiretroviral treatment in Jimma University Specialized Hospital, Southwest Ethiopia. *Trop Med Int Health*. 2008 Mar;13(3):328-33. doi: 10.1111/j.1365-3156.2008.02006.x. Epub 2008 Feb 21.

For peer review only

LAWRENCE C. LONG, PHD, MCOM

OFFICE ADDRESS: 801 Massachusetts Avenue, Crosstown Center, 3rd Floor, Room 369
Boston, MA, 02119, USA.
+1 (617) 358-3122
Email: lclong@bu.edu

ACADEMIC TRAINING:

2016	Ph.D.	School of Public Health, University of Witwatersrand (Johannesburg, South Africa)
2009	M.Com.	School of Economic and Business Sciences, University of Witwatersrand (Johannesburg, South Africa), Economics, *cum laude*
2001	B.Bus.Sci.	School of Economics, University of Cape Town (Cape Town, South Africa), Economics and Finance

ACADEMIC LEADERSHIP:

2011 - 2017	Deputy Division Head	Health Economics & Epidemiology Research Office<br>Wits Health Consortium<br>Faculty of Health Sciences, University of Witwatersrand
2008 – 2011	Research Coordinator	Health Economics & Epidemiology Research Office<br>Wits Health Consortium<br>Faculty of Health Sciences, University of Witwatersrand

RESEARCH APPOINTMENTS:

2017 – Present	Research Assistant Professor	Department of Global Health, School of Public Health,<br>Boston University
2008 – Present	Associate Researcher	Joint Faculty Appointment, School of Clinical Medicine,<br>Faculty of Health Sciences, University of the Witwatersrand
2008 – Present	Principal Researcher (Visiting)	Health Economics & Epidemiology Research Office<br>Wits Health Consortium<br>Faculty of Health Sciences, University of

For peer review only

2006 – 2007	Research Associate	Witwatersrand<br>Health Economics & Epidemiology Research Office<br>Wits Health Consortium<br>Faculty of Health Sciences, University of Witwatersrand
2005	Study Coordinator	Health Economics & Epidemiology Research Office<br>Wits Health Consortium<br>Faculty of Health Sciences, University of Witwatersrand

OTHER RESEARCH EXPERIENCE:

2007 – 2008	Consultant	Center for International Health and Development,<br>Boston University School of Public Health<br>Project: *Cost and Outcomes of Models for Delivering Antiretroviral Therapy for HIV/AIDS in Zambia*
2007 – 2008	Consultant	Center for International Health and Development,<br>Boston University School of Public Health<br>Project: *Cost and Outcomes of Models for Delivering Antiretroviral Therapy for HIV/AIDS in Kenya*

TEACHING EXPERIENCE:

2019	**Structural factors affecting HIV – an economists perspective.** Audience: MPH Students. Guest Lecture in Individual, Community, and Population Health SPH GH 720 Boston University, Boston, USA. Presented in two classes (Profs Monica Onyango & Jennifer Schlezinger).
2019	**Economic evaluation.** Audience: MPH Students. Guest Lecture in Monitoring and Evaluation of Global Health Programs SPH GH 745, Boston University, Boston, USA.
2019	**Using economics to influence policy.** Audience: MPH Students. Guest Lecture in Essential of Economics and Finance for Global Health SPH GH 762, Boston University, Boston, USA.
2017	**From cost to clinic – Economics changing health policy.** Audience: MPH Students. Guest Lecture in Essentials of Economics and Finance for Global Health SPH GH 762, Boston University, Boston, USA.
2016	**Data collection and analysis for economic evaluations.** Audience: Technical implementing partners. EQUIP Partners Meeting, Johannesburg, South Africa.

For peer review only

2016 **From patient to policy – Ensuring that your clinical practice is positioned to inform evidenced based policy.** Audience: HIV Clinicians. Chair of Research Skills Building Session, Southern African HIV Clinicians Society Conference, Johannesburg, South Africa.

2014 **Introduction to Health Programme Evaluation**. Audience: MSc students – Module of Epidemiology for Health Researchers II. University of Witwatersrand, Johannesburg.

INVITED PRESENTATION:

2019 **Costs and resources needed to provide HIV services to key populations over the next 10 years.** Idea creation meeting – Defining and addressing HIV treatment and prevention needs of underserved and high-risk populations. BMGF & Journal of International AIDS Society, New York, USA.

2019 **Direct action to achieve a result.** Spotlight on "Think, Teach, Do", School of Public Health, Boston University, Boston, USA.

2019 **Learning Community – Take home.** Academy for Faculty Advancement, School of Medicine, Boston University, Boston, USA.

2018 **Urban public health issues – the transition to Boston.** Health Economics and Epidemiology Research Office, Johannesburg, South Africa.

2017 **Partner's Area of Expertise – Health Economics.** USAID South Africa, Partners Meeting, Pretoria, South Africa. Presented in absentia by Denise Evans.

2017 **Innovations Research on AIDS (INROADS).** Director Doug Arbuckle, Office HIV AIDS (OHA, USA). USAID, Johannesburg, South Africa. Presented in absentia by Denise Evans.

2016 **Test and Start – Research supporting evidence based policy change.** Zambian Department of Health & USAID, EQUIP Project, Johannesburg, South Africa.

2016 **Initiating ART at a patients first clinic visit: the RapIT randomised trial.** Faculty of Health Sciences Research Day, University of Witwatersrand, Johannesburg, South Africa.

CONFERENCE ORAL PRESENTATIONS:

1. Van Rensburg C, Berhanu R, Hirasen K, Evans D, Rosen S, **Long L**. Cost outcome analysis of decentralised care for drug-resistant tuberculosis in Johannesburg, South Africa. 49th Union World Conference on Lung Health, 24-27 October, The Hague, The Netherlands. 2018.

For peer review only

2. Evans D, Musakwa N, Nattey C, Bor J, Lonnermark E, Larshans C, Andreasson S, Nyasulu P, **Long L**. Barriers to accessing care for HIV and Tuberculosis among adolescents and young adults aged 18 – 25 years in Johannesburg, South Africa. 5th SA TB Conference, 12-15 June, Durban, South Africa. 2018.

3. Lince-Deroche N, Leuner R, **Long L**. When Donor Funding Leaves: The immediate impact on resources of withdrawal of support for direct HIV care and treatment at public health facilities in Johannesburg. 9th International AIDS Society (IAS) Conference, 23-26 July, Paris, France. 2017.

4. Musakwa N, Evans D, Feeley A, Magwete M, Patz S, McNamara L, **Long L**, Sanne I. Acute malnutrition and dietary intake among paediatric HIV-positive patients initiating antiretroviral therapy in Johannesburg, South Africa. 8th SA AIDS Conference, 13-15 June, Durban, South Africa. 2017.

5. Rosen S, Maskew M, Fox MP, Nyoni C, Mongwenyana C, Malete G, Sanne I, Bokaba D, Sauls C, Rohr J, **Long L**. Initiating ART at a Patient's First Clinic Visit: The RapIT Randomized Trial. Conference on Retroviruses and Opportunistic Infections (CROI) 2016, 22-25 February, Boston, USA.

6. Hirasen K, Berhanu R, Schnippel K, **Long L**, Rosen S, Sanne I. Twelve-month outcomes of patients initiating drug-resistant tuberculosis treatment at a decentralized outpatient clinic in Johannesburg, South Africa. 46th World Conference on Lung Health of the International Union Against Tuberculosis and Lung Disease (The Union), Cape Town, South Africa, 2-6 December 2015.

7. Evans D, Schnippel K, Budgell E, Shearer K, Berhanu R, **Long L**, Rosen S. Predictors of mortality in patients diagnosed with preXDR- and XDR-TB: results from the South African National TB Programme, 2009-2010. 46th World Conference on Lung Health of the International Union Against Tuberculosis and Lung Disease (The Union), Cape Town, South Africa, 2-6 December 2015.

BIBLIOGRAPHY:

ORCID ID: 0000-0003-4986-4988
Google Scholar: https://scholar.google.co.za/citations?user=fao8zDgAAAAJ&hl=en&oi=ao

Peer reviewed publications:

1. **Long L**, Kuchukhidze S, Pascoe S, Nichols B, Cele R, Govathson C, Huber A, Flynn D, Rosen S. Differentiated models of service delivery for antiretroviral treatment of HIV in sub-Saharan Africa: a rapid review protocol. *Systematic Reviews*. DOI: 10.1186/s13643-019-1210-6. 2019.

For peer review only

2. Jamieson L, Evans D, Berhanu R, Ismail N, Aucock S, Wallengren K, **Long L**. Data quality of drug-resistant tuberculosis and antiretroviral therapy electronic registers in South Africa. *BMC Public Health*. DOI: 10.1186/s12889-019-7965-9. 2019.

3. Mokhele I, Mashamaite S, Majuba P, Xulu T, **Long L**, Onoya D. Effective public-private partnerships for sustainable antiretroviral therapy: outcomes of the Right to Care health services GP down-referral program. *BMC Public Health*. DOI: 10.1186/s12889-019-7660-x. 2019.

4. Lince-Deroche N, Leuner R, Meyer-Rath G, Pillay Y, **Long L**. When Donor Funding Leaves: A retrospective review of the impact of integrating direct HIV care and treatment into public health services in a region of Johannesburg. *Cost Effectiveness and Resource Allocation*. DOI: 10.1186/s12962-019-0192-5. 2019.

5. Musakwa N, Feeley A, Magwete M, Patz S, McNamara L, Sanne I, **Long L**, Evans D. Dietary intake among paediatric HIV-positive patients initiating antiretroviral therapy in Johannesburg, South Africa. *Vulnerable Children and Youth Studies (RVCH)*. DOI: 10.1080/17450128.2019.166858. 2019.

6. Brennan A, Bonawitz R, Gill CJ, Thea DM, Kleinman M, **Long L**, McCallum C, Fox MP. A Meta-analysis Assessing Diarrhea and Pneumonia in HIV-Exposed Uninfected Compared With HIV-Unexposed Uninfected Infants and Children. *Journal of Acquired Immune Deficiency Syndrome (JAIDS)*. DOI: 10.1097/QAI.0000000000002097. 2019

7. Berry KM, Rodriguez CA, Berhanu R, Ismail N, Rosen S, **Long L**, Evans D. Treatment outcomes among pediatrics, adolescents, and adults on treatment for drug-sensitive TB in two metropolitan municipalities in Gauteng Province, South Africa. *BMC Public Health*. DOI: 10.1186/s12889-019-7257-4. 2019.

For peer review only

Identifying efficient linkage strategies for HIV self-testing (IDEaL): a study protocol for an individually randomized control trial

Journal:	*BMJ Open*
Manuscript ID	bmjopen-2022-070896.R1
Article Type:	Protocol
Date Submitted by the Author:	28-Mar-2023
Complete List of Authors:	Dovel , Kathryn ; University of California Los Angeles, Division of Infectious Diseases ; Partners in Hope Medical Center, Implementation Science Department<br>Balakasi, Kelvin; Partners in Hope Medical Center, Implementation Science Department<br>Hubbard, Julie; University of California Los Angeles, Division of Infectious Diseases ; Partners in Hope Medical Center, Implementation Science Department<br>Phiri, Khumbo; Partners in Hope Medical Center, Implementation Science Department<br>Nichols, Brooke ; Boston University, Department of Global Health; Health Economics and Epidemiology Research Office,<br>Coates, Thomas; University of California Los Angeles, Division of Infectious Diseases, School of Medicine<br>Kulich, Michal; Charles University, Department of Probability and Math Statistics<br>Chikuse, Elijah; Partners in Hope Medical Center, Implementation Science Department<br>Phiri, Sam; Partners in Hope Medical Center, Implementation Science Department<br>Long, Lawrence; Boston University School of Public Health, Department of Global Health; Faculty of Health Sciences University of the Witwatersrand, Department of Internal Medicine<br>Hoffman, Risa; University of California Los Angeles, Division of Infectious Diseases<br>Choko, Augustine; University of Malawi, College of Medicine
**Primary Subject Heading**:	HIV/AIDS
Secondary Subject Heading:	Global health, Health services research, Infectious diseases, Public health
Keywords:	HIV & AIDS < INFECTIOUS DISEASES, Infection control < INFECTIOUS DISEASES, Clinical trials < THERAPEUTICS

SCHOLARONE™
Manuscripts

Identifying efficient linkage strategies for HIV self-testing (IDEaL): a study protocol for an individually randomized control trial

Kathryn Dovel[1,2§], Kelvin Balakasi[1], Julie Hubbard[1], Khumbo Phiri[1], Brooke E. Nichols[3,4], Thomas J. Coates[1], Michal Kulich[5], Elijah Chikuse[2], Sam Phiri[2], Lawrence Long[3,4], Risa Hoffman[1], Augustine Choko[6].

Author affiliations:

[1] Division of Infectious Diseases, David Geffen School of Medicine, University of California Los Angeles, Los Angeles, CA, USA
[2] Partners in Hope, Lilongwe, Malawi
[3] Department of Global Health, Boston University School of Public Health, Boston, MA, USA
[4] Department of Internal Medicine, Faculty of Health Sciences, University of the Witwatersrand, Johannesburg, South Africa
[5] Department of Probability and Math. Statistics, Faculty of Mathematics and Physics, Charles University, Prague, Czech Republic
[6] College of Medicine, University of Malawi, Blantyre, Malawi

[§] **Corresponding Author:** Kathryn Dovel
Division of Infectious Diseases, David Geffen School of Medicine
10833 Le Conte Ave, Los Angeles, CA 90095
Phone: +13108838179
Email: kdovel@mednet.ucla.edu / kldovel@gmail.com

E-mail addresses of authors:

KD: KDovel@mednet.ucla.edu
KB: kelvin@pihmalawi.com
JH: jhubbard@mednet.ucla.edu
KP: khumbo@pihmalawi.com
BN: brooken@bu.edu
TC: TCoates@mednet.ucla.edu
MK: kulich@karlin.mff.cuni.cz
EC: elijah@pihmalawi.com
SP: samphiri@pihmalawi.com
LL: lclong@bu.edu
RH: RHoffman@mednet.ucla.edu
AC: augutc@gmail.com

Word Count: Abstract: 252; Manuscript: 3,967

Trial registration number: NCT05137210

Keywords: HIV, men, ART initiation, ART retention, Stepped interventions, sub-Saharan Africa

Date and version: 13 March 2023: Version 2

ABSTRACT

For peer review only - http://bmjopen.bmj.com/site/about/guidelines.xhtml

Introduction

Men in sub-Saharan Africa are less likely than women to initiate antiretroviral therapy (ART) and more likely to have longer cycles of disengagement from ART programs. Treatment interventions that meet the unique needs of men are needed, but they must be scalable. We will test the impact of various interventions on six-month retention in ART programs among men living with HIV who are not currently engaged in care (never initiated ART and ART clients with treatment interruption).

Methods and Analysis

We will conduct a programmatic, individually randomized, non-blinded, controlled trial. “Non-engaged” men will be randomized 1:1:1 to either a Low-Intensity, High-Intensity, or Stepped arm. The Low-Intensity Intervention includes one-time male-specific counseling + facility navigation only. The High-Intensity Intervention offers immediate outside-facility ART initiation + male-specific counseling + facility navigation for follow-up ART visits. In the Stepped arm, intervention activities build in intensity over time for those who do not reengage in care with the following steps: 1) one-time male-specific counseling + facility navigation→2) ongoing male mentorship + facility navigation→3) outside-facility ART initiation + male-specific counseling + facility navigation for follow-up ART visits. Our primary outcome is 6-month retention in care. Secondary outcomes include cost-effectiveness and rates of adverse events. The primary analysis will be intention to treat with all eligible men in the denominator and all men retain in care at 6 months in the numerator. The proportions achieving the primary outcome will be compared with a risk ratio, corresponding 95% confidence interval and p-value computed using binomial regression accounting for clustering at facility level.

Ethics and Dissemination

The Institutional Review Board of the University of California, Los Angeles and the National Health Sciences Research Council in Malawi have approved the trial protocol. Findings will be disseminated rapidly in national and international forums and in peer-reviewed journals and are expected to provide urgently needed information to other countries and donors.

Trial registration number: NCT05137210.

ARTICLE SUMMARY

Strengths and limitations

- IDEaL provides male-specific differentiated models of care aimed to improve men’s ART outcomes. We specifically focus on building trusting relationships with health care workers and

For peer review only - http://bmjopen.bmj.com/site/about/guidelines.xhtml

developing client-led, individualized strategies to overcome barriers to care.

- IDEaL will test the impact of a stepped intervention for men. This approach promises to improve the efficiency and reach of HIV programs for men as the highest-resource interventions will only be received by the minority of men who are most in need.
- IDEaL develops and tests male-specific counseling curriculum that, if effective, could easily be taken to scale. Findings from the study will identify critical components for male-specific counseling, especially among men who struggle to be retained in HIV care.
- IDEaL interventions do not change facility characteristics that may act as barriers to men's use of facility-based services. IDEaL focuses on providing outside-facility services for reaching men.

For peer review only

INTRODUCTION

Men in sub-Saharan Africa (SSA) are underrepresented in HIV programs.[1] Men are less likely than women to know their HIV status and to initiate antiretroviral therapy (ART), and more likely to face treatment interruptions once in care.[2] Only 69% of men who start ART reach viral suppression compared to 77% of women.[2] As a result, men in the region are 37% more likely to die from AIDS-related causes as compared to women.[3]

One contributor to men's poor HIV outcomes is an increased risk of disengagement from care. Engagement in ART programs is not static – many ART clients cycle through care, starting and stopping HIV care multiple times throughout their lifetime.[4,5] Up to 46% of ART clients experience treatment interruption,[6–8] and between 30-40% of those who return experience repeat treatment interruption within 6 months.[9,10] Men are particularly prone to cycling through ART programs, with more frequent stop-start instances and longer periods outside of care as compared to women.[6,11–13] Improving men's long-term engagement in HIV care is critical for men's health and reducing HIV transmission.[14]

Men who disengage from HIV programs (either after testing HIV-positive or after enrolling in HIV care services) are frequently described as a difficult and 'hard-to-reach' population.[15,16] However, growing evidence suggests that men desire HIV services[17,18] but encounter multiple health systems barriers to care that make it impossible to stay in care long-term.[19] There is an urgent need to develop client-centered strategies tailored to men that facilitate men's engagement and re-engagement in HIV treatment programs.

Some men may require male-specific interventions to facilitate engagement in HIV care. Men have less exposure to HIV services than women[19,20] and work demands may conflict with ART clinic schedules.[21,22] Difficult interactions with health care workers (HCWs) can also prevent men from engaging or re-engaging in care.[23,24] Furthermore, most ART counseling curricula do not target men and often lack the client-centered counseling needed to develop internal motivation to engage and stay engaged in care.

Differentiated service delivery models (DSD) are now being developed to improve men's ART engagement throughout SSA.[25–27] As DSDs for men are developed, it is critical that strategies be feasible and cost-effective to allow scale-up. A "one size fits all" model is not as effective as more nuanced approaches.[28–30] Stepped interventions increase in intensity over time and are purposively designed to address prevailing barriers in the target population in order to positively affect the desired outcome.[31,32] An incremental, stepped approach may be the most appropriate and scalable way to improve men's care in low-resource settings. Men are not homogeneous: some men may require minimal support to engage in

For peer review only - http://bmjopen.bmj.com/site/about/guidelines.xhtml

care, while others may require extensive support. Stepped interventions allow programs to target the highest-resource interventions to the minority of men who need them most.

The *Identifying efficient linkage strategies for HIV self-testing (IDEaL) trial* is an individually randomized control trial aimed to test the impact of various interventions on ART (re-) initiation and six-month retention among men living with HIV who are not currently engaged in HIV care in Malawi. We will compare a Stepped intervention against Low-Intensity and High-Intensity interventions to assess the impact of the Stepped intervention on men's use of ART services over time (see Supporting Information S1). The trial contributes to existing literature by testing male-specific, client-centered strategies to re-engage men in care. This is one of the first trials specifically designed with men's re-engagement in care in mind. If effective, such interventions may decrease repeat treatment interruption and duration of treatment interruptions among men, which can improve viral suppression and reduce onward HIV transmission.[14]

METHODS AND ANALYSIS

Objectives

Our primary objective is to test the effect of a male-specific, Stepped intervention on men's 6-month retention in ART care compared to male-specific Low-Intensity and High-Intensity interventions (retention is defined as <28-days late for their ART appointment). Secondary objectives are to understand the effect of a Stepped intervention on: (1) ART initiation; (2) the presence of adverse events (i.e., unwanted disclosure, end of relationship, or intimate partner violence (IPV)); (3) intervention acceptability; and (4) cost-effectiveness.

Trial Design

IDEaL is a programmatic, individually randomized, non-blinded controlled trial design. We will recruit men from 15 high-burden health facilities in Malawi using medical chart reviews to identify men who are living with HIV but not engaged in HIV care.

Randomization

Individual men will be block randomized using R by a biostatistician using a 1:1:1 ratio to either the Stepped, Low-Intensity, or High-Intensity study arm using a computer-generated program. Participants will be randomized in blocks of 3 and 6, depending on the number of men available for recruitment at each facility. After enrolling in the trial and completing a baseline survey, men will be assigned to a study ID based on the randomization list. Study ID's will be linked with the pre-assigned blocked

For peer review only

randomization and pre-loaded into the tablet device, but will be unknown to the study staff until survey and randomization modules are completed and saved, ensuring randomization cannot be manipulated by the study staff. Once finalized, the randomization results will appear on the tablet device as a picture, and will be shown to the participant to maximize transparency and study buy-in.

Interventions

The effectiveness of the Stepped Intervention will be compared to a Low-Intensity Intervention (one time male-specific counseling + facility navigation defined as escort to the facility (if desired) and orientation to the ART clinic and procedures) and to a High-Intensity Intervention (outside-facility ART initiation + male-specific counseling + facility navigation for follow-up ART visits). Across all arms, men who do not (re-)initiate in ART will continue receiving follow-up visits for up to three months, depending on preferences of the client. The number of intervention visits delivered for each participant will be documented.

Arm 1: Low-Intensity Arm: Male-specific counseling + facility navigation

Participants randomized to the Low-Intensity Arm will be traced in the community and receive a one-time, one-on-one male-specific counseling session, using client-centered service techniques.[33] All counseling sessions will be completed by a lay cadre male HIV counselor (called Patient Supporter in Malawi) trained in the study counseling curriculum. Patient supporters are responsible for routine tracing, linkage support medical record documentation and counseling.

The male-specific curriculum is developed specifically for this trial. Ministry of Health counseling materials is adapted to meet the specific needs of men, based on formative in-depth interviews, focus group discussions, and a systematic literature review. Adaptations will include exploring topics of most concern to men in Malawi (i.e., earning money while HIV-positive, side effects and concerns regarding lifelong medication, ART as a tool to provide and care for family, etc.). The materials will also include language and pictures that resonate with men (i.e., emphasizing how HIV and HIV services interact with men's strength, responsibility, planning for the future), and male-specific case studies of challenges men face and how they overcome them. The adapted male-specific counseling curriculum will be developed into a standardized counseling flip chart (i.e., job aid).

Men who wish to (re-)initiate ART will be offered facility navigation and facility-based services at the facility of their choice. Participants will be escorted to the facility (if desired), orientated to the ART clinic

For peer review only - http://bmjopen.bmj.com/site/about/guidelines.xhtml

procedures, and introduced to other HCWs who routinely work at the facility. Facility navigation is intended to facilitate a positive experience for men by helping them feel comfortable and confident navigating clinic spaces. Men may access all ART clinical services at the health facility of their choice (but counseling described above will be provided in the community). The client will be responsible for all transport costs related to return to the facility in all study arms.

Participants who do not (re-)initiate in care within 14 days will be offered followed-up counseling every two weeks until participants (re-)initiate in care or inform the counselor that they do not wish to be contacted.

Arm 2: High-Intensity Arm: Male-specific counseling + outside-facility ART initiation + facility navigation

Participants in the High-Intensity Arm will be offered community-based male-specific counseling by HIV counselors (described above), and offered ART (re-)initiation outside-facility (either at home or another location in the community of their choice). Those who choose outside-facility ART initiation will be referred to a male study nurse who will meet participants one-on-one at times and locations that are convenient for participants. The nurse will offer a brief counseling session, reviewing key topics from male-specific counseling curriculum that is most relevant to the individual participant. Nurses will then conduct WHO staging. Individuals classified as WHO Stage 3 or 4 will be referred (and escorted, if desired) to the nearest public health facility for additional services. Participants classified as WHO Stage 1 or 2 will be given same day ART. Prior to their 4-week follow-up ART appointment participants will receive facility navigation by the same nurse and afterward access ART services.

Participants who choose facility-based ART (re-)initiation will be referred (and escorted, if desired) by the HIV counselor (lay cadre) to the nearest facility of their choice and receive facility navigation on a day and time that is convenient for them.

Men who do not (re-)initiate in care will be offered biweekly follow-up counseling at times and intervals determined by participants' preferences until they engage in care or inform the nurse that they no longer wish to be contacted.

Arm 3: Stepped Arm

The Stepped arm will build in intensity over time for those who have not (re-)initiate in care 14-days after study enrollment, or who do not return for their first ART follow-up appointment after (re-)initiation (see

For peer review only - http://bmjopen.bmj.com/site/about/guidelines.xhtml

Fig 1). Individuals will move to the next "step" every 2-weeks, moving from the lightest to the most intensive interventions over the course of 6 weeks until (re-)initiation has been achieved. The Stepped arm includes the following steps:

Step 1: Male-Specific Counseling + Facility Navigation and Facility-Based ART Services: Step 1 includes the same components described in the Low-Intensity Arm. Briefly, participants will be traced in the community and receive a one-time male-specific counseling session, with repeat counseling sessions if participants do not (re-)initiate within 14 days after the first counseling session. Men who wish to (re-) initiate in care will be provided facility navigation and standard of care facility-based services.

Step 2: Ongoing Motivational Interviewing + Facility Navigation and Facility-Based ART Services: Men who do not (re-)initiate in care (either have not engaged ART within 14 days of enrollment or do (re-)initiate ART but are ≥7-days late for a follow-up appointment) will move to the next 'step' of the intervention, which adds ongoing motivational interviewing to their package of activities. Motivational interviewing is a client-centered, client-led method for counseling that helps participants identify barriers to a desired outcome and develop personalized solutions.[34,35] The strategy has successfully been used with ART clients.[36] Mentors will work with participants to: (1) build self-efficacy, (2) identify internal motivations for the desired behavior, and (3) establish strategies and short- and long-term goals needed to reach ART initiation and retention. A male mentor specifically trained in motivational interviewing adapted to the local context and male population will provide ongoing, one-on-one in-depth counseling, motivational interviewing, and general "check-ins" approximately twice within a two-week period. The mentor will not necessarily be HIV-positive (unlike other mentorship models) as the Malawi Ministry of Health has moved away from HIV-positive peer mentor cadres. However, they will be experienced in HIV counseling and trained on male-specific needs. Motivational interviewing will take place in a location preferred by the participant, likely in the community. Participants who choose to (re) initiate ART can access ART services at the facility of their choice and will be given facility navigation as described in Arm 1.

Step 3: Outside-facility ART initiation + Male-Specific Counseling: Men who are not engaged after Step 2 (either have not (re-)initiated ART ≤ 14 days after moving to Step 2 or did (re-)initiate but are ≥7-days late for a follow-up ART appointment) will be offered outside-facility ART by a male nurse certified in HIV counseling. Steps will follow those outlined in the High Intensity Arm, with a brief counseling session, WHO staging, same-day ART re-initiation for those WHO stage 1 or 2, and facility navigation for their 4-week follow-up appointment.

For peer review only - http://bmjopen.bmj.com/site/about/guidelines.xhtml

[Figure 1 here]

Trial setting

The study will take place in central and southern Malawi. Malawi has an HIV prevalence of 9.6%[37] and, of the estimated 330,000 men living with HIV in the country, 54,500 are not in care.[38] Men in Malawi live in primarily rural settings, are self-employed, subsistence farmers, the minority have regular access to a private phone, and most are highly mobile.[39,40]

Population

We will recruit men from 13 high-burden health facilities in Malawi, using medical chart reviews to identify men living with HIV who are not engaged in HIV care. Study facilities will vary by facility type (hospital/health center), management (public/mission), location (rural/urban), and region (central/southern Malawi).

Eligibility criteria for men include: (1) ≥15years of age; (2) live in facility catchment area; and (3) tested HIV-positive and either (a) self-report having not yet initiated ART within 7-days of testing HIV-positive, (b) initiated ART but are at risk of immediate default (i.e., ≥7-days late for their 30-day ART refill appointment), or (c) initiated ART and attended their first refill appointment but later defaulted (i.e. ≥28-days late to care). For those who never initiated ART and do not have proof of a confirmatory HIV test, study staff will offer an HIV self-test kit prior to enrollment, to confirm a positive HIV status. Those who choose to initiate ART will receive the standard Determine and Unigold confirmatory tests prior to ART initiation, following routine care.

Study outcomes

The primary outcome is the proportion of men who are retained in ART care 6-months after (re-) engagement. Secondary outcomes include: (1) ART initiation; (2) adverse events experienced by men or their female partners (i.e., unwanted disclosure, end of relationship, or intimate partner violence (IPV); (3) intervention acceptability; and (4) cost-effectiveness. ART retention outcomes will be measured through medical chart reviews, while secondary outcomes will be measured through self-reports. Process outcomes include: (1) the proportion of men who were successfully traced; (2) the proportion of eligible men who consented to participate; (3) men's experience with the intervention; and (4) the quality of the intervention.

Sample size considerations

For peer review only - http://bmjopen.bmj.com/site/about/guidelines.xhtml

We powered the study to detect differences in 6-month retention between Stepped and Low-Intensity Arms, and the Stepped and High-Intensity Arms. Based on pilot data, we assumed that 40% of men in the Low-Intensity Arm, 60% in the Stepped Arm, and 80% in the High-Intensity Arm will engage in ART and be retained at 6-months. Any man lost to follow-up will be treated as a failure for the outcome evaluation. With 181 men per arm and 20% loss to follow-up from the study in all arms, the power for detecting the specified differences between Stepped and Low Intensity arms and between Stepped and High-Intensity arms will be 0.8, with test level 0.025 after Bonferroni adjustment for two comparisons. We will have 0.99 power to detect specified differences between Low Intensity and High Intensity arms. The calculation is based on asymptotic normality of log odds ratio.[41] We need to enroll and randomize 181 men per arm (a total of 543 men living with HIV).

Data Collection

Study recruitment, enrollment, and data collection will be conducted by study staff, who are distinct from local HCWs implementing the interventions.

Recruitment

Men will be identified through both medical register reviews and in-person recruitment at participating health facilities. Various medical charts will be reviewed to identify different types of eligible men: HIV testing and counseling (HTC) to identify men who tested HIV positive but never initiated ART; client follow-up registers to identify those who initiated but never returned for their first ART appointment, or those who defaulted from care; and index counseling and testing (ICT) registers to identify male partners of female ART clients (Figure 2). In-person recruitment will involve screening men at outpatient departments (OPD) because our previous research has found that men in Malawi frequent OPD settings for health needs,[17] and our formative work suggests that men who disengage from ART services still frequent the OPD for care. In-person recruitment will be used for all client types.

[Figure 2 here]

Tracing and Eligibility Screening

Study staff will trace potential participants identified through medical chart reviews via phone (if available) or home visits based on tracing data provided in medical documentation. All potential participants will be traced up to three times before being considered lost to follow-up. All screening and enrollment processes will take place in-person.

For peer review only

Consent, Enrollment, and Baseline Survey

Men who are eligible for the study will complete written informed consent and complete a baseline survey immediately following enrollment. The baseline survey will collect data on key demographic variables (marital status, number of children, employment, self-rated health) and previous engagement with HIV and non-HIV health services. All surveys will be conducted in the local language (Chichewa) by trained study staff using electronic tablets. Surveys will be programmed using SurveyCTO software (http://www.surveycto.com).

Follow-Up Data

Study staff will administer follow-up surveys at 2- and 4-months after enrollment. Follow-up surveys will measure exposure to (and acceptability of) the interventions, changes in key demographics since enrollment (i.e., marital status, number of children, employment, self-rated health), any adverse events since enrollment (i.e., unwanted status disclosure, termination of relationship due to the intervention), and use of ART services. The location and specific time of the follow-up survey will be based on participant preference.

Medical chart reviews will be conducted to assess men's engagement with ART services 6-months after study enrollment. Individuals without a medical chart outcome will be followed-up in person and their health passport, a pocket medical record where providers record data during health visits, will be reviewed to collect the ART outcome. Men who cannot be reached or are lost to follow-up in any arm will be counted as failures for that specific ART outcome of interest: (re-)initiation or 6-month retention).

Patient and Public Involvement

Extensive formative work informed the development of the study protocol including in-depth interviews, focus group discussions, and a systematic literature review. The study protocol and tools were presented to Ministry of Health, national stakeholders and implementing partners (see Supporting Information S2).

Cost data

The average cost per successful outcome (6-month retention) will be calculated and compared across arms incrementally. We will use micro-costing methods by creating an inventory of the resources used to achieve the observed study outcomes including: (1) standard counseling interactions (staff cadre, training received, duration of interaction and distance from facility travelled where applicable); (2) motivational interviewing interactions (staff cadre, training received, duration of interaction and distance from facility travelled where applicable); (3) provider interactions (staff cadre, training received, duration of

For peer review only - http://bmjopen.bmj.com/site/about/guidelines.xhtml

interaction and distance from facility travelled where applicable); and (4) cost of reminder messages sent, when messages are delivered telephonically instead of in person. For each study patient, the quantity (number of units) of resources used will be determined. Costs will be measured from the health care provider perspective. Unit costs of resources, which are not human subject data, will be obtained from external suppliers and the health facilities' finance and procurement records and multiplied by the resource usage data to provide an average cost per study patient in each study arm. A cost-effectiveness analysis will be conducted by dividing the incremental cost between two arms by the incremental effectiveness (number of people retained at 6-months) in the respective arms.

Analysis plan

Data analysis will be conducted in R: A Language and Environment for Statistical Computing (R Foundation for Statistical Computing). We will use the Consolidated Standards of Reporting Trials (CONSORT) standards for reporting trial outcomes.[42] Using an intention-to-treat analysis, all randomized men will be included in the analysis of the primary outcome; men with missing outcomes due to loss to follow-up will be treated as outcome failures. We will calculate descriptive statistics, including mean, standard deviation, range, and frequency distributions for the demographic characteristics and study outcomes by study arm. The primary outcome and all other binary outcomes will be analyzed by logistic regression models with age, marital status, health care facility and other key sociodemographic variables included as covariates. The intervention effects will be tested by Wald tests of the relevant regression parameters. The hypotheses of no difference between the stepped arm and the low/high intensity arm will be rejected if the p-value is smaller than 0.025 (Bonferroni adjustment). Confidence intervals for odds ratios comparing the stepped arm to the low intensity and high intensity arms with coverage probabilities 0.975 will be calculated by profile likelihood methods. Due to the Bonferroni adjustment, the simultaneous coverage probability of both intervals will be at least 0.95. To address the secondary objectives, more elaborate logistic regression models will be built for each of the binary outcomes with available individual-, community-, and facility-level factors included as covariates in addition to the intervention status.

Nested studies

A series of nested, mixed methods studies will be conducted to identify factors associated with ART engagement within each intervention arm, and to explore the implementation and acceptability of interventions.

Qualitative data collection

For peer review only

We will conduct in-depth interviews with a random subset of 40 male participants per arm (120 total) throughout the study period. Clients will be randomly selected at various times of the study using computer-generated randomization, stratifying the sample by arm and successful trial outcomes (i.e., did clients re-initiate ART and/or reach 6-month retention). Data will assess characteristics of men who fail to engage in care, contextualize decisions around ART initiation and retention, and identify additional strategies that may be needed for men to successfully engage and be retained in ART programs (see Supporting Information 3). Data collection tools and analysis plans will be informed by the Andersen's Emerging Model of Health Services Use, phase 4[43] that examines multi-level factors that influence health outcomes. Specifically, it examines the interaction of: 1) environment and structure of health services; 2) clients' enabling resources; and 3) clients' perceived need/motivation to access services. Qualitatively understanding how the IDEaL interventions influence these levels, and what barriers still remain, will help refine future interventions.

Interviews will be conducted by a trained male interviewer in the local language. Interviews will be digitally recorded, transcribed, and translated into English for analysis. Investigators will pilot a codebook by independently reading and coding a randomly-selected subset of transcripts. Through an iterative consultative process, each investigator will revise their respective codebook until there is high interrater reliability among the group. All transcripts will be coded in Atlas.ti v8.3[44] and text analyzed using constant comparison methods[45] to compare and contrast themes that arise within and between interventions and trial outcomes.

Implementation Log Sheet

During the course of the intervention, HCWs will keep daily logs as one of the study monitoring and evaluation tools to assess the implementation of the intervention for each participant. Primary events to be recorded in the daily logs are: (1) unable to reach participant (and reason); (2) contacted participant; (3) intervention provided (and notes about the challenges and successes of the interaction); and (4) other comments relevant to intervention implementation. Each event will be recorded with a corresponding date. Logs will be digitized in English. Findings may influence how similar interventions are implemented in the future.

Ethics and Dissemination

The IDEaL trial is registered with ClinicalTrials.gov as NCT05137210. The protocol was approved by the Institutional Review Board of the University of California, Los Angeles and the National Health Sciences Research Council in Malawi. Study findings will be disseminated through peer-reviewed journal articles,

For peer review only - http://bmjopen.bmj.com/site/about/guidelines.xhtml

For peer review only

national and international conference presentations, and meetings with Malawi Ministry of Health, facility, and community stakeholders.

DISCUSSION

Studies have reported poorer outcomes for HIV testing, treatment initiation, and treatment adherence in men compared to women[2] for over a decade.[46,47] Men are often portrayed as difficult, hard-to-reach, and actively avoiding health facilities. In IDEaL, we aim to investigate whether men really are hard-to-reach or, will men engage in care when services are offered in ways that are accessible to them and resonate with their needs, as growing evidence suggests.[17] We propose to test a Stepped intervention that increases in intensity over time against Low- and High-intensity interventions – all tailored to men – to identify the most cost effective strategy to (re-)initiate men in HIV treatment services in Malawi.

IDEaL is different from other ART engagement and re-engagement interventions in several important ways. First, we will enroll men living with HIV across the treatment cascade, including those who have never initiated ART, those who are at risk of immediate default after initiation, and those who have been in care but subsequently default. Formative research suggests that barriers to ART initiation and re-initiation may be similar,[48] however most interventions focus specifically on either first-time initiation or re-engagement, but not both. Our study will assess if one overarching program can improve men's engagement across the treatment cascade, regardless of whether they are starting ART for the first time or returning to care after a period of disengagement. One overarching intervention may be more scalable than multiple, separate interventions across the cascade. Second, we tailor interventions to men's unique needs and motivations, based on extensive formative work. While innovative interventions for men are underway,[25–27] few have rigorously tested the impact of male-tailored interventions on ART engagement.[49]

Finally, we will test a Stepped intervention that builds in intensity over time until men (re-)initiate in care. This approach allows men who are ready to (re-)initiate to do so at minimal cost to the health system, while those who need additional support can receive more resource-intensive interventions to support their ART engagement.[31] Stepped interventions have been effective in other settings and can address multiple barriers faced by the target population with minimal cost.[31,32] Findings from IDEaL will provide crucial knowledge to how best men can be reached and can inform intervention scale-up.

Contributorship statement: KD and AC conceptualized the study. KD is responsible for funding acquisition. KD, KB, JH, KP, BN, RH and AC developed study protocol and materials. JH, KB, KP, and

For peer review only - http://bmjopen.bmj.com/site/about/guidelines.xhtml

EC will implement the study. KD, MK, KB, TC, BN, LL, TC, and AC developed the analysis plan and KD, MK, KB, and AC will analyze the data. KD and EC wrote the first draft and KB, JH, KP, BN, LL, RH, SP, EC, RH, TC, and AC edited following drafts. All authors have read and approved the final manuscript.

Competing interests: The authors declare that they have no competing interests.

Funding: The work was supported by the Bill and Melinda Gates Foundation grant number INV-001423. KD was supported by National Institute of Mental Health of the National Institutes of Health grant number R01-MH122308, Fogarty International grant number K01-TW011484-01 and UCLA GSTTP (grant number N/A). LL was supported by the National Institute of Mental Health of the National Institutes of Health under grant number K01MH119923. The content is solely the responsibility of the authors and does not necessarily represent the official views of the National Institutes of Health.

Acknowledgements: We wish to thank the Malawi Ministry of Health for their support of this trial. We would also like to acknowledge Joep van Oosterhout, Misheck Mphande, Isabella Robson, Thoko Banda, Peter Mwamlima, and Eric Lungu for their contributions to protocol development and for their support of study implementation.

For peer review only

REFERENCES

1. Cornell M, Majola M, Johnson LF, Dubula-Majola V. HIV services in sub-Saharan Africa: the greatest gap is men. *The Lancet*. 2021;397(10290):2130-2132.
2. AIDSinfo | UNAIDS. Accessed February 21, 2023. https://aidsinfo.unaids.org/
3. Druyts E, Dybul M, Kanters S, et al. Male sex and the risk of mortality among individuals enrolled in antiretroviral therapy programs in Africa: a systematic review and meta-analysis. *Aids*. 2013;27(3):417-425.
4. Ehrenkranz P, Rosen S, Boulle A, et al. The revolving door of HIV care: Revising the service delivery cascade to achieve the UNAIDS 95-95-95 goals. *PLoS Medicine*. 2021;18(5):e1003651.
5. Grimsrud A, Wilkinson L, Eshun-Wilson I, Holmes C, Sikazwe I, Katz IT. Understanding engagement in HIV programmes: how health services can adapt to ensure no one is left behind. *Current HIV/AIDS Reports*. 2020;17:458-466.
6. Kranzer K, Lewis JJ, Ford N, et al. Treatment interruption in a primary care antiretroviral therapy programme in South Africa: cohort analysis of trends and risk factors. *Journal of acquired immune deficiency syndromes (1999)*. 2010;55(3):e17.
7. Mee P, Rice B, Lemsalu L, et al. Changes in patterns of retention in HIV care and antiretroviral treatment in Tanzania between 2008 and 2016: an analysis of routinely collected national programme data. *Journal of global health*. 2019;9(1).
8. Tomescu S, Crompton T, Adebayo J, et al. Factors associated with an interruption in treatment of people living with HIV in USAID-supported states in Nigeria: a retrospective study from 2000–2020. *BMC public health*. 2021;21:1-8.
9. Nakiwogga-Muwanga A, Musaazi J, Katabira E, Worodria W, Talisuna SA, Colebunders R. Patients who return to care after tracking remain at high risk of attrition: experience from a large HIV clinic, Uganda. *International journal of STD & AIDS*. 2015;26(1):42-47.
10. Teklu AM, Yirdaw KD. Patients who restart antiretroviral medication after interruption remain at high risk of unfavorable outcomes in Ethiopia. *BMC Health Services Research*. 2017;17(1):1-8.
11. Lopez-Varela E, Augusto O, Fuente-Soro L, et al. Quantifying the gender gap in the HIV care cascade in southern Mozambique: We are missing the men. *Plos one*. 2021;16(2):e0245461.
12. Mody A, Eshun-Wilson I, Sikombe K, et al. Longitudinal engagement trajectories and risk of death among new ART starters in Zambia: A group-based multi-trajectory analysis. *PLOS medicine*. 2019;16(10):e1002959.
13. Gosset A, Protopopescu C, Larmarange J, et al. Retention in care trajectories of HIV-positive individuals participating in a universal test-and-treat program in rural South Africa (ANRS 12249 TasP Trial). *Journal of Acquired Immune Deficiency Syndromes (1999)*. 2019;80(4):375.
14. Monod M, Brizzi A, Galiwango RM, et al. Growing gender disparity in HIV infection in Africa: sources and policy implications. *medRxiv*. Published online 2023:2023-03.
15. Esacove AW. Good sex/bad sex: the individualised focus of US HIV prevention policy in sub-Saharan Africa, 1995–2005. *Sociology of health & illness*. 2013;35(1):33-48.
16. Poulin M, Dovel K, Watkins SC. Men with money and the "vulnerable women" client category in an AIDS epidemic. *World Development*. 2016;85:16-30.
17. Dovel K, Balakasi K, Gupta S, et al. Frequency of visits to health facilities and HIV services offered to men, Malawi. *Bulletin of the World Health Organization*. 2021;99(9):618.
18. Chamberlin S, Mphande M, Phiri K, Kalande P, Dovel K. How HIV Clients Find Their Way Back to the ART Clinic: A Qualitative Study of Disengagement and Re-engagement with HIV Care in Malawi. *AIDS and Behavior*. 2022;26(3):674-685.
19. Dovel K, Dworkin SL, Cornell M, Coates TJ, Yeatman S. Gendered health institutions: examining the organization of health services and men's use of HIV testing in Malawi. *Journal of the International AIDS Society*. 2020;23:e25517.

For peer review only - http://bmjopen.bmj.com/site/about/guidelines.xhtml

20. Yeatman S, Chamberlin S, Dovel K. Women's (health) work: a population-based, cross-sectional study of gender differences in time spent seeking health care in Malawi. *PLoS One*. 2018;13(12):e0209586.
21. Colvin CJ. Strategies for engaging men in HIV services. *The Lancet HIV*. 2019;6(3):e191-e200.
22. Phiri K, McBride K, Moucheraud C, et al. Community and health system factors associated with antiretroviral therapy initiation among men and women in Malawi: a mixed methods study exploring gender-specific barriers to care. *International health*. 2021;13(3):253-261.
23. Ware NC, Wyatt MA, Geng EH, et al. Toward an understanding of disengagement from HIV treatment and care in sub-Saharan Africa: a qualitative study. *PLoS medicine*. 2013;10(1):e1001369.
24. Layer EH, Brahmbhatt H, Beckham SW, et al. "I pray that they accept me without scolding:" Experiences with disengagement and re-engagement in HIV care and treatment services in Tanzania. *AIDS patient care and STDs*. 2014;28(9):483-488.
25. Bell J, Sharma S, Malone S, et al. Targeting interventions for HIV testing and treatment uptake: an attitudinal and behavioural segmentation of men aged 20–34 in KwaZulu-Natal and Mpumalanga, South Africa. *PloS one*. 2021;16(3):e0247483.
26. van Rooyen H, Makusha T, Joseph P, et al. Zwakala Ndoda: a cluster and individually randomized trial aimed at improving testing, linkage, and adherence to treatment for hard-to reach men in KwaZulu-Natal, South Africa. *Trials*. 2019;20(1):1-14.
27. MenStar Coalition | Ending AIDS for all. MenStar Coalition. Accessed March 15, 2023. https://www.menstarcoalition.org/
28. Grimsrud A, Bygrave H, Doherty M, et al. Reimagining HIV service delivery: the role of differentiated care from prevention to suppression. *Journal of the International AIDS Society*. 2016;19(1).
29. Duncombe C, Rosenblum S, Hellmann N, et al. Reframing HIV care: putting people at the centre of antiretroviral delivery. *Tropical Medicine & International Health*. 2015;20(4):430-447.
30. Hagey JM, Li X, Barr-Walker J, et al. Differentiated HIV care in sub-Saharan Africa: a scoping review to inform antiretroviral therapy provision for stable HIV-infected individuals in Kenya. *AIDS care*. 2018;30(12):1477-1487.
31. Bower P, Gilbody S. Stepped care in psychological therapies: access, effectiveness and efficiency: narrative literature review. *The British Journal of Psychiatry*. 2005;186(1):11-17.
32. McNairy ML, Lamb MR, Gachuhi AB, et al. Effectiveness of a combination strategy for linkage and retention in adult HIV care in Swaziland: The Link4Health cluster randomized trial. *PLoS medicine*. 2017;14(11):e1002420.
33. Hubbard J, Mphande M, Robson I, Balakasi K, Phiri K. Core components of male-specific person-centered care: a qualitative analysis from client and healthcare worker perspectives in Malawi. In: ; 2023.
34. Miller WR, Rose GS. Toward a theory of motivational interviewing. *American psychologist*. 2009;64(6):527.
35. Rollnick S, Miller WR, Butler CC, Aloia MS. Motivational interviewing in health care: helping patients change behavior. Published online 2008.
36. Hill S, Kavookjian J. Motivational interviewing as a behavioral intervention to increase HAART adherence in patients who are HIV-positive: a systematic review of the literature. *AIDS care*. 2012;24(5):583-592.
37. Focus on: Malawi | UNAIDS. Accessed March 15, 2023. https://www.unaids.org/en/20190402_country_focus_Malawi
38. Malawi | UNAIDS. Accessed March 15, 2023. https://www.unaids.org/en/regionscountries/countries/malawi
39. Feldacker C, Emch M, Ennett S. The who and where of HIV in rural Malawi: Exploring the effects of person and place on individual HIV status. *Health & place*. 2010;16(5):996-1006.
40. Mandiwa C, Namondwe B. Uptake and correlates of HIV testing among men in Malawi: evidence from a national population–based household survey. *BMC health services research*. 2019;19:1-8.

For peer review only - http://bmjopen.bmj.com/site/about/guidelines.xhtml

41. Jewell NP. *Statistics for Epidemiology*. CRC press; 2003.
42. Schulz KF, Altman DG, Moher D. CONSORT 2010 statement: Updated guidelines for reporting parallel group randomized trials. *Annals of Internal Medicine*. 2011;154(4):291-292.
43. Andersen RM. National health surveys and the behavioral model of health services use. *Medical care*. Published online 2008:647-653.
44. ATLAS.ti 9 [computer program].
45. Glaser BG. The constant comparative method of qualitative analysis. *Social problems*. 1965;12(4):436-445.
46. Higgins JA, Hoffman S, Dworkin SL. Rethinking gender, heterosexual men, and women's vulnerability to HIV/AIDS. *American journal of public health*. 2010;100(3):435-445.
47. Braitstein P, Boulle A, Nash D, et al. Gender and the use of antiretroviral treatment in resource-constrained settings: findings from a multicenter collaboration. *Journal of women's health*. 2008;17(1):47-55.
48. Coursey K, Kalande P, Phiri K, Chamberlin S, Coates T, Dovel K. Developing intervention strategies to improve ART program initiation and retention among HIV-positive men in Malawi.
49. Kusemererwa S, Akena D, Nakanjako D, et al. Strategies for retention of heterosexual men in HIV care in sub-Saharan Africa: a systematic review. *PloS one*. 2021;16(2):e0246471.

FIGURE LEGEND

Figure 1: Trial Design

Figure 2: Recruitment sources and ART disengagement criteria by recruitment type

SUPPORTING INFORMATION

Supporting Information 1: Spirit Checklist

Supporting Information 2: Approved protocol

Supporting Information 3: In-depth interview guides

For peer review only

Figure 1: Trial Design

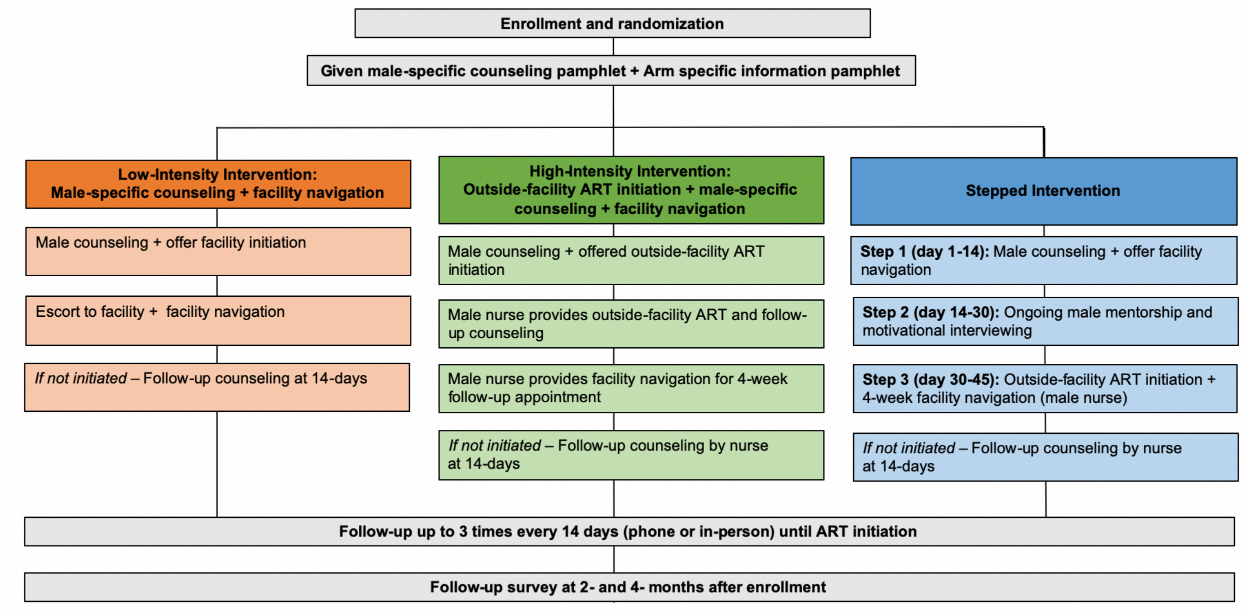

For peer review only

Fig 2. Recruitment sources and ART disengagement criteria by recruitment type

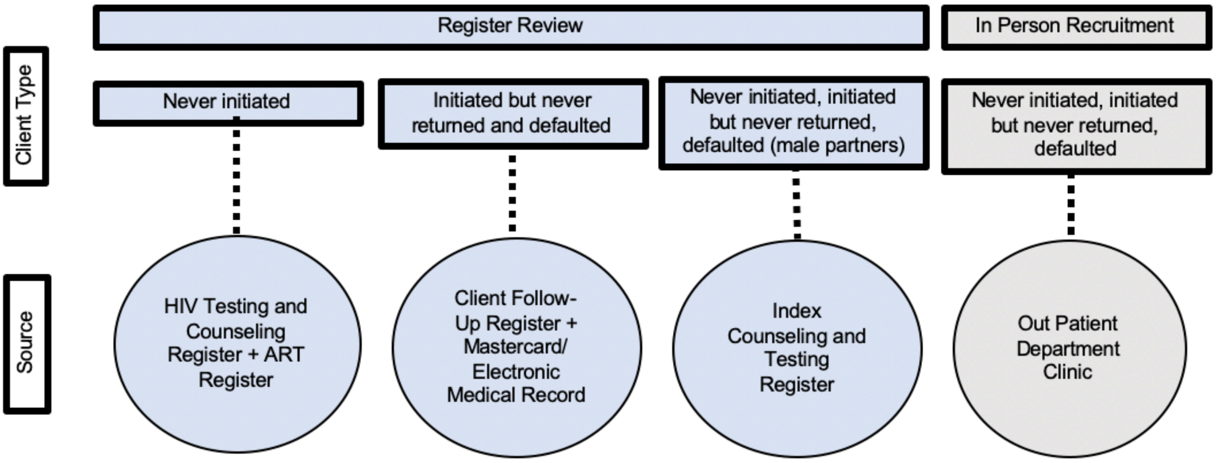

For peer review only

SPIRIT

STANDARD PROTOCOL ITEMS: RECOMMENDATIONS FOR INTERVENTIONAL TRIALS

SPIRIT 2013 Checklist: Recommended items to address in a clinical trial protocol and related documents*

Section/item	Item No	Description	Addressed on page number
**Administrative information**			
Title	1	Descriptive title identifying the study design, population, interventions, and, if applicable, trial acronym	______1_______
Trial registration	2a	Trial identifier and registry name. If not yet registered, name of intended registry	______1, 15_____
	2b	All items from the World Health Organization Trial Registration Data Set	______n/a______
Protocol version	3	Date and version identifier	______2_______
Funding	4	Sources and types of financial, material, and other support	______19______
Roles and responsibilities	5a	Names, affiliations, and roles of protocol contributors	_____18-19____
	5b	Name and contact information for the trial sponsor	______19______
	5c	Role of study sponsor and funders, if any, in study design; collection, management, analysis, and interpretation of data; writing of the report; and the decision to submit the report for publication, including whether they will have ultimate authority over any of these activities	______19_______
	5d	Composition, roles, and responsibilities of the coordinating centre, steering committee, endpoint adjudication committee, data management team, and other individuals or groups overseeing the trial, if applicable (see Item 21a for data monitoring committee)	_____n/a_______

For peer review only

Introduction

Background and rationale	6a	Description of research question and justification for undertaking the trial, including summary of relevant studies (published and unpublished) examining benefits and harms for each intervention	____4-5_______
	6b	Explanation for choice of comparators	_____ ________
Objectives	7	Specific objectives or hypotheses	_____5_______
Trial design	8	Description of trial design including type of trial (eg, parallel group, crossover, factorial, single group), allocation ratio, and framework (eg, superiority, equivalence, noninferiority, exploratory)	_______5_______

Methods: Participants, interventions, and outcomes

Study setting	9	Description of study settings (eg, community clinic, academic hospital) and list of countries where data will be collected. Reference to where list of study sites can be obtained	____9-10_______
Eligibility criteria	10	Inclusion and exclusion criteria for participants. If applicable, eligibility criteria for study centres and individuals who will perform the interventions (eg, surgeons, psychotherapists)	____10______
Interventions	11a	Interventions for each group with sufficient detail to allow replication, including how and when they will be administered	____6-9______
	11b	Criteria for discontinuing or modifying allocated interventions for a given trial participant (eg, drug dose change in response to harms, participant request, or improving/worsening disease)	_______n/a______
	11c	Strategies to improve adherence to intervention protocols, and any procedures for monitoring adherence (eg, drug tablet return, laboratory tests)	_______n/a______
	11d	Relevant concomitant care and interventions that are permitted or prohibited during the trial	_______n/a______
Outcomes	12	Primary, secondary, and other outcomes, including the specific measurement variable (eg, systolic blood pressure), analysis metric (eg, change from baseline, final value, time to event), method of aggregation (eg, median, proportion), and time point for each outcome. Explanation of the clinical relevance of chosen efficacy and harm outcomes is strongly recommended	_____10_____
Participant timeline	13	Time schedule of enrolment, interventions (including any run-ins and washouts), assessments, and visits for participants. A schematic diagram is highly recommended (see Figure)	__6,8-9, 11-12__

For peer review only - http://bmjopen.bmj.com/site/about/guidelines.xhtml

Sample size	14	Estimated number of participants needed to achieve study objectives and how it was determined, including clinical and statistical assumptions supporting any sample size calculations	____10-11_____
Recruitment	15	Strategies for achieving adequate participant enrolment to reach target sample size	____11-12______

Methods: Assignment of interventions (for controlled trials)

Allocation:

Sequence generation	16a	Method of generating the allocation sequence (eg, computer-generated random numbers), and list of any factors for stratification. To reduce predictability of a random sequence, details of any planned restriction (eg, blocking) should be provided in a separate document that is unavailable to those who enrol participants or assign interventions	_____5-6_______
Allocation concealment mechanism	16b	Mechanism of implementing the allocation sequence (eg, central telephone; sequentially numbered, opaque, sealed envelopes), describing any steps to conceal the sequence until interventions are assigned	_____5-6_______
Implementation	16c	Who will generate the allocation sequence, who will enrol participants, and who will assign participants to interventions	______5_______
Blinding (masking)	17a	Who will be blinded after assignment to interventions (eg, trial participants, care providers, outcome assessors, data analysts), and how	______6_______
	17b	If blinded, circumstances under which unblinding is permissible, and procedure for revealing a participant's allocated intervention during the trial	______n/a______

Methods: Data collection, management, and analysis

Data collection methods	18a	Plans for assessment and collection of outcome, baseline, and other trial data, including any related processes to promote data quality (eg, duplicate measurements, training of assessors) and a description of study instruments (eg, questionnaires, laboratory tests) along with their reliability and validity, if known. Reference to where data collection forms can be found, if not in the protocol	_____12_____
	18b	Plans to promote participant retention and complete follow-up, including list of any outcome data to be collected for participants who discontinue or deviate from intervention protocols	_____12_____

Data management	19	Plans for data entry, coding, security, and storage, including any related processes to promote data quality (eg, double data entry; range checks for data values). Reference to where details of data management procedures can be found, if not in the protocol	_______n/a_______
Statistical methods	20a	Statistical methods for analysing primary and secondary outcomes. Reference to where other details of the statistical analysis plan can be found, if not in the protocol	_____13-14_______
	20b	Methods for any additional analyses (eg, subgroup and adjusted analyses)	___13-14____
	20c	Definition of analysis population relating to protocol non-adherence (eg, as randomised analysis), and any statistical methods to handle missing data (eg, multiple imputation)	______13-14______
**Methods: Monitoring**			
Data monitoring	21a	Composition of data monitoring committee (DMC); summary of its role and reporting structure; statement of whether it is independent from the sponsor and competing interests; and reference to where further details about its charter can be found, if not in the protocol. Alternatively, an explanation of why a DMC is not needed	_______n/a_______
	21b	Description of any interim analyses and stopping guidelines, including who will have access to these interim results and make the final decision to terminate the trial	_______n/a_______
Harms	22	Plans for collecting, assessing, reporting, and managing solicited and spontaneously reported adverse events and other unintended effects of trial interventions or trial conduct	_____12 _______
Auditing	23	Frequency and procedures for auditing trial conduct, if any, and whether the process will be independent from investigators and the sponsor	_______n/a_______
**Ethics and dissemination**			
Research ethics approval	24	Plans for seeking research ethics committee/institutional review board (REC/IRB) approval	_______15________
Protocol amendments	25	Plans for communicating important protocol modifications (eg, changes to eligibility criteria, outcomes, analyses) to relevant parties (eg, investigators, REC/IRBs, trial participants, trial registries, journals, regulators)	______n/a_______

Consent or assent	26a	Who will obtain informed consent or assent from potential trial participants or authorised surrogates, and how (see Item 32)	______12_______
	26b	Additional consent provisions for collection and use of participant data and biological specimens in ancillary studies, if applicable	______n/a______
Confidentiality	27	How personal information about potential and enrolled participants will be collected, shared, and maintained in order to protect confidentiality before, during, and after the trial	______5______
Declaration of interests	28	Financial and other competing interests for principal investigators for the overall trial and each study site	______19_______
Access to data	29	Statement of who will have access to the final trial dataset, and disclosure of contractual agreements that limit such access for investigators	______n/a______
Ancillary and post-trial care	30	Provisions, if any, for ancillary and post-trial care, and for compensation to those who suffer harm from trial participation	______n/a______
Dissemination policy	31a	Plans for investigators and sponsor to communicate trial results to participants, healthcare professionals, the public, and other relevant groups (eg, via publication, reporting in results databases, or other data sharing arrangements), including any publication restrictions	____2,15______
	31b	Authorship eligibility guidelines and any intended use of professional writers	______n/a______
	31c	Plans, if any, for granting public access to the full protocol, participant-level dataset, and statistical code	______n/a______
**Appendices**			
Informed consent materials	32	Model consent form and other related documentation given to participants and authorised surrogates	_____ n/a ______
Biological specimens	33	Plans for collection, laboratory evaluation, and storage of biological specimens for genetic or molecular analysis in the current trial and for future use in ancillary studies, if applicable	______n/a______

*It is strongly recommended that this checklist be read in conjunction with the SPIRIT 2013 Explanation & Elaboration for important clarification on the items. Amendments to the protocol should be tracked and dated. The SPIRIT checklist is copyrighted by the SPIRIT Group under the Creative Commons "Attribution-NonCommercial-NoDerivs 3.0 Unported" license.

Partners in Hope Medical Center

Identifying efficient linkage strategies for HIV self-testing (IDEaL)

For peer review only

Kathyrn Dovel, Principle Investigator
Partners in Hope
PO Box 302
3-6-2019

ABSTRACT

Background: HIV self-testing (HIVST) has been found to be a highly acceptable approach for men to learn of their HIV status and has resulted in increased testing uptake (Dovel 2019, cite Augustines stuff). However, rates of antiretroviral therapy (ART) initiation among those tested with HIVST are difficult to capture and some studies have suggested that linkage rates are low (Ortblad 2017, MacPherson 2014), particularly amongst men. We propose a clinical trial to test varying approaches to ART initiation among men who test HIV-positive through HIVST. We will test three interventions:

Lightest Touch Intervention (Arm 1): simple reminders to visit the health facility (given every two weeks);

Staged Intervention (Arm 2): a staged intervention that consecutively increases in intensity every month that a participant does not initiate ART (intervals include reminders, motivational interviewing, and home-based ART initiation);

Intensive Intervention (Arm 3): home-based ART initiation followed by linkage to the health facility of their choice the following month.

Objective: Our primary objective is to identify a cost-effective package for ART initiation among men identified as HIV-positive through HIVST in Malawi. Our specific objectives are:

Objective 1. Evaluate the effectiveness of the Staged ART Intervention vs Lightest Touch Intervention (primary analysis) and the effectiveness of the Staged ART Intervention vs Intensive Intervention (secondary analysis) on ART initiation within 4-months after enrolment in the trial.

Objective 2. Identify individual-, community-, and facility-level factors associated with ART initiation within each intervention arm (Lightest Touch; Staged; and Intensive Interventions).

Objective 3. Determine the cost and scalability of each intervention (Lightest Touch; Staged; and Intensive Interventions).

Methods: We will preform an individually randomized control trial with 543 HIV-positive men identified through HIVST and their female partners. Men will be individually randomized 1:1:1 to one of the three intervention arms described above. The study will be preformed at 10 health facilities suppored by Partners in Hope (PIH). Data collection will include baseline and follow-up surveys and interviews with men and women; medical charter reviews at four-months after study enrollment; qualitative interviews; and a cost analysis of costs associated with each arm. Participants will be enrolled in the study for a total of 4 months with approximately 2 or 3 study visits throughout that period.

Anticipated results: We anticipate learning about the most effective stragty to engage men in ART. We also anticipate learning about the type and degree of followup necessary to support men's engagement in ART services. Finally, we anticipate learning about the cost-effectiveness of intervention, with the goal of improving cost-effectiveness for the Ministry of Health. Results from this study could be used to define best practices and to further scale ART-focused programs for men in Malawi.

For peer review only

TABLE OF CONTENTS

1. INTRODUCTION 4

1.1. Background 4
1.2. Problem statement 4
1.3. Justification 4

2. OBJECTIVES 4

2.1. Primary objective 4
2.2. Secondary objectives 4

3. LITERATURE REVIEW 5
4. METHODOLOGY 8

4.1. Intervention description 9
4.2. Place of study 11
4.3. OBJECTIVE 1 12

4.3.1. Study design 12
4.3.2. Target population 12
4.3.3. Sampling techniques and enrollment 13
4.3.4. Study Activites 13
4.3.5. Data collection techniques and tools 15
4.3.6. Sample size determination 17
4.3.7. Data analysis 17

4.4. OBJECTIVE 2 17

4.4.1. Study design 17
4.4.2. Target population 17
4.4.3. Sampling techniques and tools 18
4.4.4. Data collection techniques and tools 18
4.4.5. Sample size determination 19
4.4.6. Data analysis 19

4.5. OBJECTIVE 3 20

4.5.1. Study design 20
4.5.2. Data collection techniques and tools 20
4.5.3. Data analysis 20

5. ETHICAL CONSIDERATIONS 21
6. DISSMINATION OF RESULTS 22
7. PERSONNEL ROLES AND INSTITUTIONS 23
8. REGULATORY OVERSIGHT 24
9. STUDY IMPLEMENTATION 24
10. PROTOCOL DEVIATION REPORTING 25
11. WORK PLAN TIMELINE 25
12. BUDGET AND JUSTIFICATION 25
13. REFERENCES 27
14. APPENDIX 30

14.1. APPENDIX A – Study Invitation Cards 30

For peer review only

14.2. APPENDIX B – Recruitment and Screening Script 32
14.3. APPENDIX C – Written Informed Consent- Female 36
14.4. APPENDIX D: Written Informed Consent – Male 46
14.5. APPENDIX E: Baseline Survey – Male 55
14.6. APPENDIX F: Baseline survey – Female 100
14.7. APPENDIX G: Follow-up Survey - Male............ 129
14.8. APPENDIX H: Follow-up Survey – Female 137
14.9. APPENDIX I: Data Extraction Tool............ 144
14.10. APPENDIX J: In-Depth Interview Guide – Female............ 147
14.11. APPENDIX K: In-Depth Interview Guide – Male 155
14.12. APPENDIX L: Personel CV's............ 165

For peer review only

1. INTRODUCTION

1.1. Background

HIV self-testing (HIVST) is an effective strategy to improve HIV testing coverage, especially among hard-to- reach populations such as men and youth. Index testing, whereby a HIV positive client gives an HIVST kit to their sexual partner to use at home, is considered beneficial for its ability to maintain a testers privacy. The method is now recommended by the World Health Organization (WHO) and is being adopted as policy throughout sub-Saharan Africa (SSA). However, uptake of antiretroviral therapy (ART) and adherence after utilizing HIVST remains sub-optimal among certain population, specifically men. Innovative ART initiation and early retention strategies are urgently needed for Index HIVST to be successful.

1.2. Problem statement

We must better understand how to engage men in HIV care. Specifically, there is limited literature on feasible differentiated models to support men to start and stay on treatment that can be taken to scale. Further, there is increased recognition that individuals are at greatest risk of loss-to-follow-up during transition periods across the cascade (i.e. when starting ART). ART initiation and early retention must be improved if HIVST is to become a viable option for high-risk groups in SSA. To address this gap, we propose to conduct a study to test and evaluate varying strategies for ART initiation and retention amongst men.

1.3. Justification

This study will combine HIVST with a second-level intervention focused on ART initiation to address the urgent gap in ART initiation and early retention among HIVST users. Additionlly, Objective 3 will allow us to develop the lowest cost intervention package while reaching the highest number of male partners.

2. OBJECTIVES

2.1. Primary objective

Objective 1. Test the impact of a staged ART intervention vs simple reminders and the effectiveness of a staged ART intervention vs home-based ART on ART initiation within 3-months of an HIV-positive diagnosis

2.2. Secondary objectives

Objective 2. Identify individual-, community-, and facility-level factors associated with ART initiation within each intervention.

Objective 3. Determine the cost and scalability of each intervention.

For peer review only

3. LITERATURE REVIEW

Background

Men in sub-Saharan Africa are less likely than women to use HIV services.[1] Men's absence from care is concerning not only for their own health, but also for the health of girls and young women who continue to be infected at unacceptably high rates.[2] HIV prevention and treatment programs have not traditionally been directed at men. Men are notably absent from international guidelines, national policies, and local HIV interventions. Research shows that women are 322% more likely to be mentioned in international HIV guidelines than men.[3] In the context of Malawi, national guidelines expect women of reproductive age to attend a health facility 5-17 times per year (equivalent to 19-63 hours)[4], and 180-472 times in their reproductive lifespan (15-44 years). There are no such expectations for men (see Table 1). The justification for the global attention of HIV program thus far on women and girls is without dispute. Gender inequality is a key driver which impacts women's health and access to HIV services and creates specific vulnerabilities for women to HIV infection.[5] However, framing HIV as a woman's concern means we have failed to understand how gender affects and drives the burden of ill health for men, and inadvertently perpetuates the epidemic for young women and girls. Targeted strategies specific to men are urgently needed if we are to engage them in care.

Table 1: *Malawi ministry of health recommended health services and estimated visits required across the reproductive life span (15-44 years)*

			Estimated number of visits between 15-44 years			
Service	Frequency	Target Population	Women: 5-year FP (Implant; 9%*)	Women: quarterly FG (injectables; 23%*)	Women: monthly FP (pills, 2%*)	Men
ANC	17.6	Women	18	18	18	-
Delivery	4.4	Women	4	4	4	-
Post-natal	4.4	Women	4	4	4	-
Family Planning	88	Women	7	88	264	-
Under five	120	Women	120	120	120	-
HIV testing	22.6	Women and men	23	23	23	29
Circumcision	3	Men	-	-	-	3
**Total**			**176**	**257**	**433**	**32**

Male partners of women who are already identified as HIV-positive (index partners) are still a major concern for epidemic control due to high rates of multiple and concurrent partnerships among men[6] and the fact that index male partners have two times the risk of being HIV-positive as compared to the general male population.[7] Data from a recent HIVST study show that there is a high need for index testing among men in Malawi: across 3 high-burden district hospitals in Malawi, men represented 73% of all index partners in need of testing. Among those who did test, male partners were 4 times more likely to test HIV-positive than female partners (23% versus 3%), representing urgent unmet need among men.[8]

A recent Index HIV Trial in Malawi found that HIV testing among men increased dramatically when HIV-positive clients give HIVST kits to their sexual partners to use at home.[9] The study found that 66% of male partners in the HIVST arm tested for HIV compared to only 22% of men in the standard partner referral slip arm. Within the HIVST arm, men who tested for HIV had an HIV-positivity rate of 23%, with no adverse events reported (see Fig 1).[2] Index HIVST is highly acceptable and allows men to test at times and locations convenient for them, with complete privacy in their own homes.[10,11]

However, innovative ART initiation and early retention strategies are urgently needed for Index HIVST

For peer review only - http://bmjopen.bmj.com/site/about/guidelines.xhtml

to be successful. The aforementioned study showed that ART initiation was unacceptably low, with only 22% of HIV-positive men in the HIVST arm initiating ART at 6-months versus 75% of men in standard partner referral slip arm).[3] (See Figure 1). Poor rates of ART initiation are commonly reported across most HIVST studies, with ART initiation rates ~20-45%[7,12–14], although ART initiation is notoriously difficult to measure within HIVST strategies. A cost analysis for national scale-up of Index HIVST in Malawi showed that 76% of men tested must initiate ART for Index HIVST to be cost-neutral at the national level as compared to using partner referral slips.

Figure 1: *Male partner use of HIV services Index HIVST vs. referral slips (n=285) from HIVST trial (PI: Dovel)*

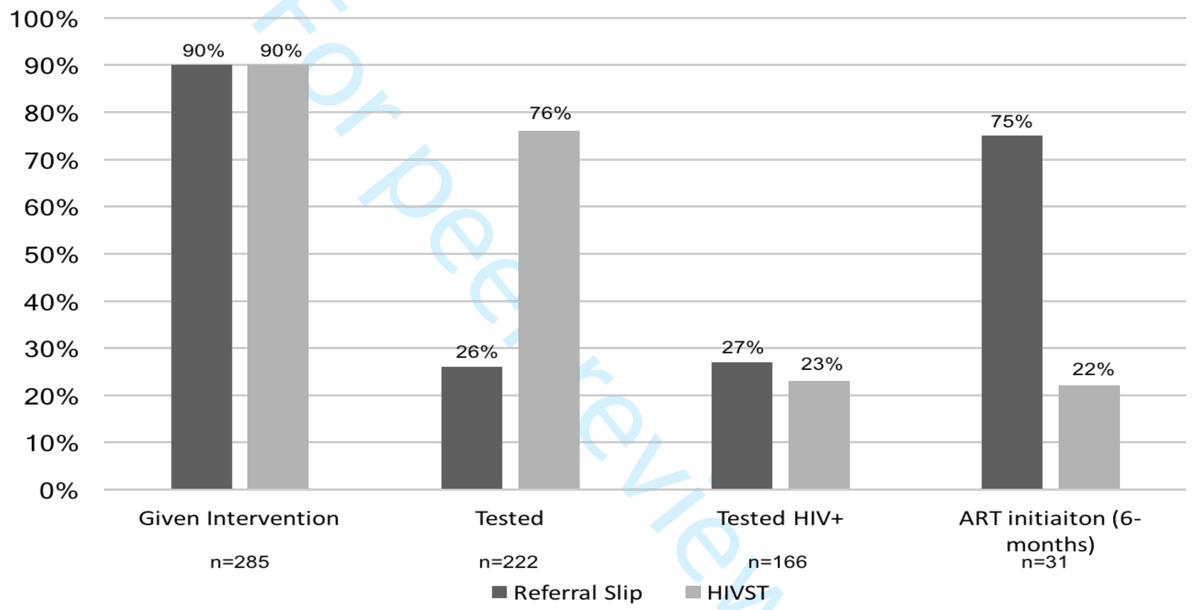

Two overarching barriers keep HIV-positive men from accessing ART services: (1) lack of male-friendly services;[15–17] and (2) harmful gender norms.[18–20] Male friendly services are private and convenient (requiring minimal time), and offered by health workers who understand the unique needs of men.[21] In addition, men are often unfamiliar with the health system, and are unsure how to navigate facility-based services. Gender norms that prioritize men as strong and self-reliant perpetuate fear of unwanted disclosure and stigma, and discourage men's engagement in ART.[18,21] Our research in Malawi found similar barriers to ART initiation for men who tested HIV-positive: men avoided ART services due to (1) fear of unwanted disclosure and stigma due to lack of privacy; (2) time/cost required to access care; (3) poor knowledge about the benefits of early ART initiation; and (4) beliefs that require men be strong, in control, and focused on short-term benefits such as daily financial earnings and respect from their male friends. Index HIVST must be combined with innovative ART interventions that address these barriers.

Evidence based for interventions that increase ART initiation

We have conducted a thorough search of the literature and have identified several intervention strategies that may increase ART initiation among men who use HIVST: (1) reminders + peer navigation; (2) motivational interviewing; and (3) home-based ART.

Reminders + Peer Navigation is shown to help clients overcome fears about facility-based services and

For peer review only

provide peer modeling how to live successfully with HIV.[22] While the strategy has been primarily tested within traditional HIV testing strategies, we hypothesize that the same mechanisms will work for men who test through Index HIVST. Reminders are usually done over the phone via phone calls or SMS and can vary in frequency based on the health care workers disgression. Peer Navigation is assisted guidance to the health clinic as well as overviews of where to go/what to do when at the facility once there to ensure men feel more comfortable in the clinic environment.

Motivational Interviewing is becoming widely recognized as a key strategy to help clients navigate barriers to the desired outcome by building client's self-efficacy, identifying internal motivation for the desired behavior, and establishing strategies and short- and long-term goals needed to reach a desired outcome.[8,23] Motivational interviewing is seen as particularly effective when clients need to make difficult decisions and overcome multi-level barriers to behavior change.[24,25] The strategy has been used to improve ART adherence[8,25] and reduce sexual risk behavior.[15]

In contrast, traditional counselling efforts are largely informational and directive, whereby health care workers deliver a pre-determined counseling package that is not responsive to a client's individual situation.[16,17] Such methods have been proven largely ineffective,[18] particularly with hard-to-reach populations such as men.[19] Motivational interviewing differs from traditional strategies by adopting a client-centered approach is based on collaboration, evocation and respect for autonomy. We hypothesize that these counseling techniques will encourage HIV status acceptance and disclosure, promote health seeking behavior, provide coping strategies men need to overcome barriers related to facility-based care, and ultimately, facilitate ART initiation.

Furthermore, motivational interviewing and client-centered care should resonate with and address the needs of men. Partners in Hope Malawi conducted 25 interviews with men and 6 focus group discussions with health care workers and female partners (n=42) to assess what health services men desired. Overwhelmingly, men reported wanting increased counseling on sexual health (including HIV) and marital concerns. Exit surveys with male ART clients (n=180) show that only 38% of men were aware of Treatment as Prevention and 65% aware of the benefits of early ART initiation, highlighting major knowledge gaps that may influence engagement in care. Motivational interviewing and client-centered counseling will be able to address both gaps in ART treatment and sexual health knowledge.

Home-based ART initiation has improved ART initiation across the region. A systematic review found that home-based ART is associated with ART retention, decreased mortality,[26] and in some cases, reduced stigma and increased privacy.[27,28] We conducted one of the only studies to examine home-based ART initiation within a community HIVST distribution strategy (Co-I: Choko). We found that home-based ART initiation alongside home-based HIVST significantly increased ART initiation as compared to standard facility-based initiation (RR 2.94; p-value<0.001).[13]

Home-based ART may be particularly attractive to hard-to-reach men because it reduces client time required to access services and provides an easy, opt-out entry point for men who otherwise may have never engaged with the health care system, or know how to navigate complicated, busy health facilities. Home-based ART has been associated with a three-fold reduction in financial costs to clients.[29] Further, home-based ART facilitates client-centered, one-on-one care that is often not feasible in busy clinic settings.

The Malawi Ministry of Health is in the process of rolling out community-based ART distribution strategies, and may consider home-based ART initiation for hard-to-reach populations. However, home-based ART is considered a resource intensive strategy, and therefore should (1) only be offered to the hardest-to-reach populations, and (2) requires that clients who initiate ART at home-based eventually link into facility-based care. Additionally, findings from a recent Index HIVST Trial show that home-based services for men is acceptable to female ART clients in the Malawian context, with minimal risk of adverse events. Over 90% of female ART clients had disclosed their HIV-status to their partner and were willing to have their male partners traced in their homes for additional services.

For peer review only - http://bmjopen.bmj.com/site/about/guidelines.xhtml

Finally, increased privacy and decreased wait-times are essential if men are to engage in HIV services. As part of a study on new Universal Treatment policies in Malawi, 15 in-depth interviews and 208 surveys were conducted with newly diagnosed HIV-positive men. Fear of unwanted disclosure due to limited privacy and a lack of trust in the health facility were the primary barriers to men's ART initiation. Home-based ART initiation can help address these barriers for ART initiation, and motivational interviewing can help provide men the skills needed to navigate these barriers within the health system in order to promote ART retention. Further, the vast majority (>95%) of men who used HIVST in the Index HIVST trial disclosed their HIV status to their female partner[8], meaning that home visits (i.e., reminders, peer navigation, motivational interviewing, or home-based ART) will not increase risk of unwanted disclosure to one's sexual partner. Table 2 below outlines how the proposed interventions will address barriers to ART initiaiton identified in the literature.

Table 2. *Levels and specific barriers to men's ART initiation, and intervention components*

Barriers to ART Initiation	Intervention Components	Level of Intervention
Unfamiliar with health system	Reminders and Peer Navigation	Health System
Belief about gender norms and focus on short-term benefits	Motivational Interviewing	Community/Individual
Poor knowledge		
Time/cost requirements	Home-Based ART Initiation	Health System
Lack of privacy/fear of disclosure		
Unfamiliar with health system		

4. METHODOLOGY

This study will be an individually randomized trial comparing three dfferent strategies to improve ART initiation and early retention among men who test HIV-positive with HIVST. Study staff will utilize the Minsitry of Health Index Testing Register and trace HIV positive women and their male partners to be screened and enrolled if they meet the inclusion criteria. Enrolled men will be randomized to one of three arms and will receive follow up and varying degrees of support based on the arm assigned. Outcomes will be assessed after 90 days after enrollment. Survey data, qualitative data, medical chart data (Objectives 1 and 2) and costing data (Objective 3) will be collected over years 1-3. A study flow chart is illustrated below (Figure 2).

Figure 2: *Study flow chart*

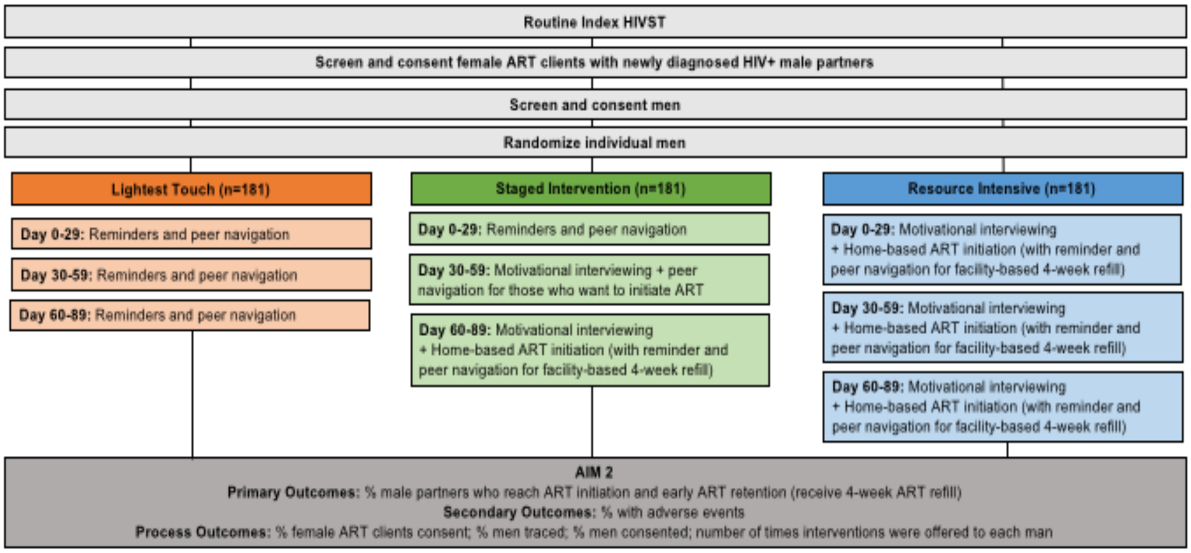

4.1. Intervention description

Each arm will offer an intervention immediately after study enrollment (that same day; day 0). Follow-up interventions will be offered every 14 days after that until 76 days or ART initiation is reached, whichever comes first.

Description of study arms:

Arm 1: "Lightest Touch" Intervention, whereby facility staff provide reminders and peer navigation (SMS and home visits) for men to encourage enrolment in facility-based ART programs. One reminder on the day of enrollment and every 14 days thereafter, until 76 days or ART initiation, whichever comes first. If initiation is not reached at 90 days, the patient will be classified as not initiated for study purposes. See Arm 1 diagram below:

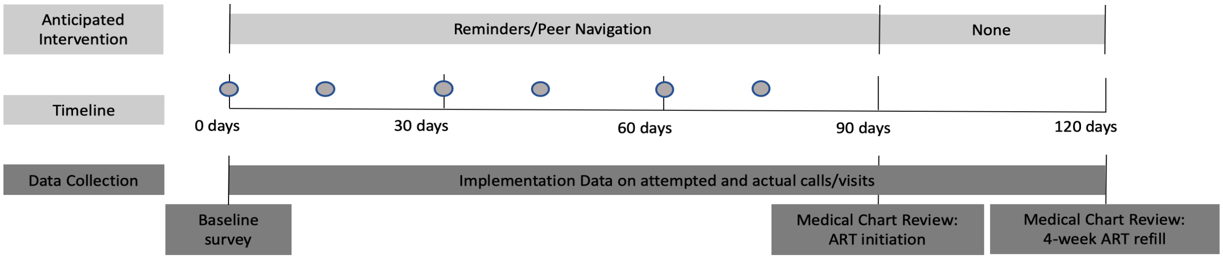

Fig 2. Timing of interventions to be offered in Aim 1

Legend:
Approximate time the intervention will be offered (approximately every 2 weeks until 76 days, or until ART initiation, whichever comes first

Arm 2: "Staged" Intervention, whereby the intervention will <u>build in intensity each month for those</u>

who have not initiated ART in the previous month, or for a maximum of 3 months, whichever comes first. The following intervention components that will be added each month (incrementally) until the first ART distribution is completed:

- Day 0-29: Reminders and, for those who agree to initiate, peer navigation;
- Day 30-59: Motivational interviewing and, for those who agree to initiate, peer navigation;
- Day 60-79: Motivational interviewing + home-based ART initiation and, for those who initiate, reminders and peer navigation for the facility-based 4-week ART refill appointment).

See Arm 2 diagram delow:

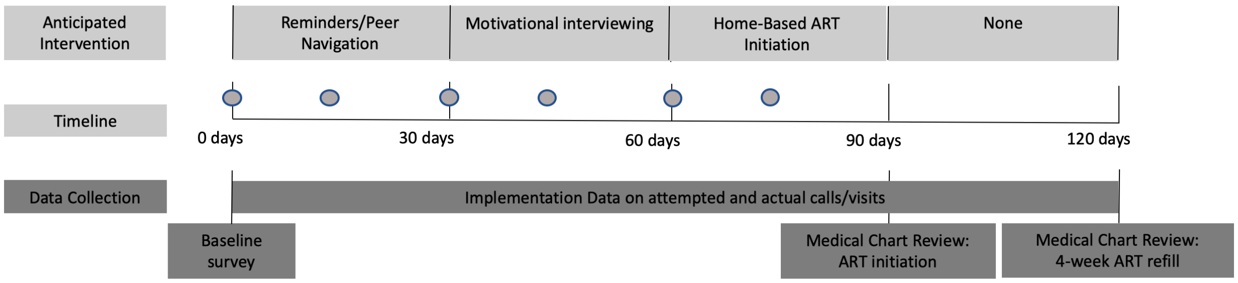

Fig 3. Timing of interventions to be offered in Aim 2

Legend:

Approximate time the intervention will be offered (approximately every 2 weeks until 76 days, or until ART initiation, whichever comes first

Arm 3: "Intensive" Intervention, whereby the most resource intensive intervention is offered immediately to all HIV-positive male partners. See Fig 4 for timeline. Components include:

- Motivational interviewing
- Home-based ART initiation
- 4-weeks after ART initiation: Reminders and peer navigation to facility for 4-week ART refill appointment

Home-based ART initiation will be scheduled at times convenient for men, including evening and weekend hours. Men who prefer to initiate in another private location in the community (besides their home) will be able to do so. The first home-visit will be conducted by a trained nurse and will include confirmatory HIV testing using Ministry of Health standard algorithm (Determine + Unigold), pre-ART counseling and motivational interviewing, a basic health evaluation, and ART initiation with a 30-day supply of first-line ART in Malawi – dolutegravir, tenofovir, and lamivudine as a single tablet. Clients will also be given a 30-day supply of cotrimoxazole, which is standard of care for all HIV-positive individuals.

Prior to ART initiation, a basic health evaluation will be performed by the nurse, including screening for tuberculosis with routine questions.[66] Any individual identified by the study nurse with concerns for an active opportunistic infection or other health problem(s) that could complicate home-based ART will be immediately referred and escorted to the facility.

At the same visit, motivational interviewing will be performed in preparation for men to engage in facility-based ART services. This includes counseling on the benefits of early ART, strategies for disclosure and positive living, strategies to overcome facility-based barriers to ART services, and addressing harmful gender norms that may discourage men from using care. Counseling will be adaptive to the needs and concerns of male clients. At 4-weeks after ART initiation, an expert client will escort the

review only

man to a nearby facility of his choice to join the facility-based ART cohort. Peer navigation will be provided to ensure men become familiar with the facility-based program. Men who wish to attend a facility that is not nearby will be linked with a male counselor from the selected facility. After completing all facility-based ART services for that day, the male partner will receive additional client-centered counseling with the same counselor to discuss the experience, benefits and challenges associated with facility-based ART, and strategies to overcome barriers. See Arm 3 diagram below:

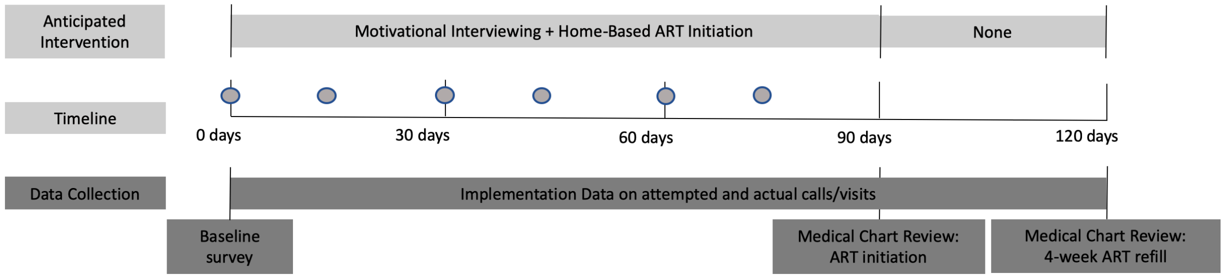

Fig 4. Timing of interventions to be offered in Aim 3

Legend:
Approximate time the intervention will be offered (approximately every 2 weeks until 76 days, or until ART initiation, whichever comes first

4.2. Place of study

All study activities will take place in 10 Partners in Hope supported facilities within the Lilongwe and Chikwawa districts, representing 19,198 adult female ART clients across all sites. These districts were chosen because they are priority districts for the Presidents Emergency Plan for AIDS Relief (PEPFAR) and have the highest HIV prevalence and unmet need of all Partners in Hope supported districts. See Table 3 for the selected 10 health facilities.

Table 3: *Selected study sites*

District	Facility name	ART Cohort Size
Chickwawa	St. Monftord Mission Hospital	3533
Chickwawa	Kalemba Community Hospital	2087
Chikwawa	Chickwawa District Hospital	4898
Kasungu	Kasungu District Hospital	5942
Lilongwe	Nkhoma Community Hospital	2166
Lilongwe	Mponela Rural Hospital	1099
Lilongwe	Daeyang Luke Hospital	1594
Nkhotakota	Nkhotakota District Hospital	5361

For peer review only - http://bmjopen.bmj.com/site/about/guidelines.xhtml

Nsanje	Nsanje District Hospital	3904
Nsanje	Ngabu Rural Hospital	1237

Specific methodology for each Objective is described below:

4.3. OBJECTIVE 1

Evaluate the effectiveness of the Staged ART Intervention vs Lightest Touch Intervention (primary analysis) and the effectiveness of the Staged ART Intervention vs Intensive Intervention (secondary analysis) on ART initiation within 4-months after enrolment in the trial.

- **Hypothesis 1.1:** 25% of men will initiate ART with simple reminders compared to 45% with motivational interviewing
- **Hypothesis 1.2:** 65% of men will initiate ART with home-based ART initiation compared to 45% with motivational interviewing

4.3.1. Study design

We will conduct an individually randomized controlled trial at 10 high-burden facilities in Malawi.

4.3.2. Target population

We will enroll 543 HIV-positive men identified through routine Index HIVST strategies and their female partners (1,086 participants total). While men are the primary focus of the study, female partners will be enrolled in order to understand their perception of their male partners use of ART services, acceptability of the intervention, and any unintended outcomes or adverse events.

Eligibility Critiera

Female ART clients will be enrolled in order to conduct baseline and follow-up surveys to understand their perception of their male partners use of ART services, acceptability of the intervention, and any unintended outcomes or adverse events.

Female Partner

- Inclusion criteria include: (1) client and partner are ≥15years of age; (2) partner lives in facility catchment area; (3) partner tested HIV-positive and has not initiated ART; and (4) ART client reports no interpersonal violence (IPV) as defined by WHO with their current sexual partner in the past 12 months.

- Exclusion criteria include: (1) client and partner are <15years of age; (2) partner does not live in facility catchment area; (3) partner has not tested HIV-positive or has testing positive and has initiated ART; and (4) ART client has reported interpersonal violence (IPV) as defined by WHO with their current sexual partner in the past 12 months.

Men will be enrolled as the primary recipient of the intervention.

Male partner

- Inclusion criteria include: (1) self and partner ≥15 years of age; (2) live in the facility catchment area (i.e., in the past 30 days, has spent ≥50% of all nights in the village); (3) has tested HIV-positive and has not initiated ART;

- Exclusion criteria include: (1) self and partner <15 years of age; (2) does not live in the facility catchment area (i.e., in the past 30 days, has spent <50% of all nights in the village); (3) has not tested HIV-positive or has tested HIV-positive and has initiated ART

4.3.3. Sampling techniques and enrollment

Sampling, screening, and enrolling male partners will be embedded within routine Index HIVST strategies.

Brief description of Routine MOH HIVST Guidelines

Briefly, routine Index HIVST includes three steps:

- *Identify ART clients with sexual partners in need of Index HIVST:* as outlined in the Malawi guidelines within the health care facility.
- *Distribute Index HIVST:* ART clients with a partner of unknown status will receive standard Index HIVST kit. They will be provided a demonstration, counseling, and an overview of risks and benefits.
- *Follow-Up on Index HIVST Use:* During their next ART appointments, ART clients are asked about HIVST distribution, use, and result of the HIVST kit, along with male partner linkage to a health facility for those who tested HIV-positive (i.e., confirmatory HIV testing and ART initiation). Data are documented in the Index HIVST register.

Once routine Index HIVST activities are completed, study recruitment and enrollment will commence. See Figure 3 for a complete description of particiant enrollment and study activities.

Figure 3: *Study screening and enrollment procedures flow chart*

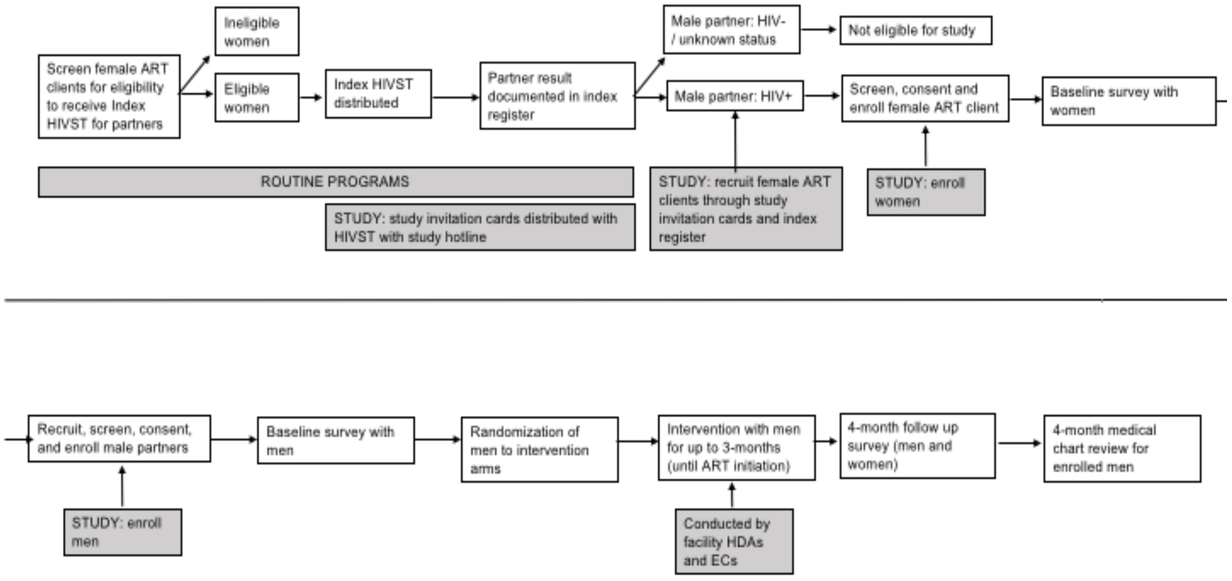

4.3.4. Study Activites

Female ART client recruitment

To maximize the number of male partners available for enrollment, the study team will introduce study invitation cards as part of Index HIVST activities and include them with HIVST kits distributed as a part of routine Index HIVST procedures (described above). Study invitation cards will include a 'hotline' phone number and will not provide any HIV specific information to ensure confidentiality. (see Appendix A for study invitation card). Female partners will be informed that should their male partner be found HIV-positive, they and their partner may be eligible for a study and should call the hotline number of the invitation card to receive more information.

In addition, the study team will review the Index Testing Register on a regular basis to identify female ART clients who reported HIV-positive male partners through routine Index HIVST procedures. Female ART clients identified will be contact by routine facility staff to be informed about the study and refered to study staff if interested in enrollment.

Female ART client enrollment

Female partners interested in the study will provide oral consent to participate in study screening and be screened for eligibility for the study (Appendix B). Study staff will conduct all consent and screening activities. If eligibile and willing to participant, written informed consent will be obtained (Appendix C). Female partners will be enrolled even if their male partner (1) does not consent to study participation (2) cannot be traced.

Upon enrollment in the study, female ART clients will work with study staff to make a plan for inviting male partners to enroll in the study. Baseline surveys with female ART clients will be conducted that same day or on a day convenient for the female ART client. Women whose partners agree to participate in the study will be followed up after 4-months for a follow up survey regarless of the outcome of their partner (ie loss-to-follow-up).

Male partner recruitment

Once female ART clients are enrolled, male partners will be recruited, screened, and consented. Study staff will work with the female ART client to establish a recruitment plan that is acceptable and feasible for the women. This may include women referring male partners to the study staff, study staff actively recruiting male partners based on information provided by the female client, or a joint approach where the study staff approaches male partners with the female ART client. Each female ART client enrollled in the study will be allowed to choose the recruitment strategy that best fits her individual situation and the needs of her partner.

Male partner enrollment

Men found eligibile and willing to participante, will provide written informed consent (Appendix D) and will be randomized to one of the three study arms. Randomization will occur using an electronic randomization system on tablet devices. A baseline survey will be concuted immediately following consent and the intervention will be carried out over the course of 3 months. Men will be contacted by the study staff 4-months after enrollment for a follow-up survey.

Randomization

Randomization will be conducted after completion of the baseline survey with male participants. Study staff will show randomization results as a picture on a pre-programmed tablet, allowing the participant to view the results themselves in order to maximize transparency and study buy-in. Male participants will be randomized to 1 of three arms and will be randomizied 1:1:1.

For peer review only

Intervention

Interventions will be offered immediately after study enrollment (that same day or the closest day that is convenient for the male participant). Follow-up interventions will be offered every 14 days after enrollment until 76 days after enrollment in the study or ARTinitiation is reached, whichever comes first. See a full description of the intervention in the Intervention Description section (4.1).

Intervention arms include:

1. ***Lightest touch arm:*** reminders and peer navigation to facility-based ART services
2. ***Staged arm:*** intervention builds in intensity each month for those who have not initiated ART in the previous month. Strategies include reminders and peer navigation, motivational interviewing, and home-based ART initiation
3. ***Intensive arm:*** home-based ART initiation + motivational interviewing + peer navigation to facility-based ART services for their 4-week follow-up appointment

Male expert clients and male nurses will complete all intervention activities to ensure it is as close to real-world implementation as possible (not implemented by Research Assistants). Research Assistants will support facility staff to ensure men are given the appropriate intervention (based on randomization arm), and that all staff activities related to the intervention are documented (i.e., how many reminders were given to each client, ect.). Intervention monitoring and evaluation tools will be developed and incorporated into the facility staff daily routine. Weekly reviews of all intervention monitoring tools and planning for the following week will be completed with study expert clients, nurses, and Research Assistant to ensure adherence to the study protocol. The Study coordinators and PI's will be highly involved throughout the implementation process to ensure protocol adherence.

4.3.5. Data collection techniques and tools

Data collection tools will include:

- *Baseline Survey:* Research assistants will administer baseline surveys with both female ART clients and male partners immediately following enrollment (before randomization). Surveys will collect data on male and female demographics, sexual partnerships and couple dynamics, and men's history with health services, and HIV services specifically. (Appendix E & F)

- *Follow-up Survey:* Research assistants will administer follow-up surveys with female ART clients and male participants 4-months after enrolment in the study. Follow-up surveys with men will assess the primary outcome of interest (ART initiation and completion of 4-week follow-up appointment), acceptability of the intervention, and any adverse events (ie., unwanted status disclosure). Men who cannot be reached will be counted as failures for true ART initiation. Follow-up survyes with female ART clients will assess acceptability of the intervention and any adverse events (i.e., IPV, end of the relationship, or unwanted disclosure) associated with intervention procedures. (Appendix G & H)

- *Medical Chart Reviews:* Identifiers will be collected for all men enrolled in the study, including name, age, village and address, and phone number. Identifiers will be used to conduct medical chart reviews at 4-months after enrollment as another measure of ART initiation (attendance to the 4-week follow-up ART appointment). Facility staff (established data clerks employed by Partners in Hope) will review medical records at study facilities and all other Partners in Hope

For peer review only - http://bmjopen.bmj.com/site/about/guidelines.xhtml

supported facilities within participating districts (61 facilities in total) to account for men who engage in ART outside study facilities. We successfully used this method in other HIVST studies to capture ART initiation.[3] Male partners who are not found in medical chart reviews will receive a follow-up home-visit to confirm ART outcomes through review of their individual medical record book (health passport) and self-reporting in the event that there are gaps in the record. Men who cannot be reached will be counted as failures for true ART initiation. (Appendix I)

- *Process Implementation Data:* Expert clients, nurses, and research assistants will keep daily logs as part of study monitoring and evaluation tools in order to assess the implementation of the intervention for each participant. Primary events to be recorded in the daily logs are: (1) unable to reach participant (and reason); (2) contacted participant; (3) intervention provided (and notes about the challenges and successes of the interaction; and (4) other comments relevant to intervention implementation. Each event will be recorded with a corresponding date.

Primary and secondary outcomes are measured through medical chart reviews and follow-up surveys (see Table 4).

Table 4: *Study Measures for Objective 1*

Outcome	Measurement	Source
Primary Outcomes		
Early ART Retention	Proportion of men who initiate ART at 3-months <u>and</u> attended their 4-week ART refill appointment at 4-months after enrollment	Medical chart review at 4-months
Secondary Outcomes		
ART initiation	Proportion of men who initiate ART at 3-months after enrollment	Medical chart review at 3-months
Adverse events by female ART client (IPV, unwanted disclosure, end of relationship) or male partner (unwanted disclosure)	Self-report from female ART client and their male partners who were identified as HIV-positive	Follow-up surveys at 4-months
Process Outcomes		
Proportion of female ART clients who consent	Proportion of eligible ART clients who consent to the participate in the study	Process implementation data
Proportion of men traced	Proportion of men who were successfully traced within 3-months after female ART client is enrolled	Process implementation data
Proportion of men who consented	Proportion of eligible men who consent to participate in the study	Process implementation data

- *Sensitivity analyses for men excluded from the trial*: We recognize that men's consent to participate in the study may bias the sample enrolled in the study. There are two groups that we may not be able to include in the main study: (1) men we are unable to trace/contact (herein referred to as "unreachable men") and (2) men we are able to reach but who refuse to participate in the main trial (herein referred to as "male refusers"). We will take two approaches to address this potential bias
    - <u>Unreachable Men</u>*:* We will collect data on men who are unreachable via their female partner. Female partners for these men will complete a brief survey regarding Surveys will collect data on male and female demographics, sexual partnerships and couple dynamics, and men's history with health services, and HIV services specifically (as reported by female partners). We successfully used similar methods in the Index HIVST Trial.
    - <u>Male Refusers</u>*:* Men who are contacted but do not consent to the trial will be consented for a one-time survey immediately following refusal for the larger study. The same data will be collected, as described above.

4.3.6. Sample size determination

We powered the study to detect differences in ART initiation between Lightest Touch and Staged Interventions at 4-months after enrollment (primary outcome). We also assured we were powered to detect differences in ART initiation between Staged Interventions and the Intensive Intervention. We Assume that 25% of men in the Lightest Touch arm, 55% in the Staged Intervention arm, and 75% in the Resource Intensive arm initiate ART at 3-months and attend their 4-week follow-up appointment at 4-months. Any man lost to follow-up in any arm will be treated as failures for the outcome evaluation. The sample size needed to detect this difference with the power of 0.8 is 181 men per arm. The calculation is based on asymptotic normality of log odds ratio. We need to enroll and randomize a total of 543 HIV-positive men. Assuming that 25% of women have partners of unknown status, 65% of male partners will use the HIVST kit, 25% of them will be HIV-positive, and 80% of them will enroll in the study, we will need to screen over 3,000 women who were given index HIVST to reach the required sample size.

4.3.7. Data analysis

All randomized men will be included in the analysis of primary outcomes; men with missing outcome assessment due to loss to follow-up will be treated as outcome failures. All primary outcomes are binary; they will be analyzed by logistic regression models with intervention as a predictor, adjusted for baseline socioeconomic and demographic variables. We will conduct sensitivity analyses to account for men who we were never able to contact (unreachable men) and men who refused to participate in the full trial (refusers). We will run several analyses whereby the denominator includes (1) unreachable men and refusers; and (2) refusers.

4.4. OBJECTIVE 2

Identify individual-, community-, and facility-level factors associated with ART initiation within each intervention arm (Lightest Touch; Staged; and Intensive Interventions).

- **Hypothesis 2.1***:* In quanitative data, older men, men without strong social support networks, men with high levels of internalized and perceived HIV-related stigma, and men who hold rigid beliefs of gender norms and men's role as the provider and decision maker of the home will be less likely to initate ART.
- **Hypothesis 2.2***:* In qualitative data, primary factors influencing men's decision to start ART will be perceptions of feeling healthy, perceptions of one's ability to continue working and providing for their family without ART initiation, and perceptions of HIV-related stigma within one's community.

4.4.1. Study design

We will use baseline survey data from the randomized trial (Objective 1) to identify factors associated with ART initiation among men. We will also conduct 200 semi-structured in-depth qualitative interviews with a random sub-set of enrolled men (n=100) and their female partners (n=100) to assess in-depth characteristics of men who fail to engage in care, contextualize decisions around ART initiation and retention, and understand additional strategies that may be needed for male partners to successfully initiate and be retained in ART programs.

4.4.2. Target population

All men and women enrolled in the overarching trial will compelte a baseline survey. Eligibility criteria for study enrollment is described in detail under Objective 1.

A subset of men and women enrolled in the overarching trial will be randomly selected to complete an in-depth interview. Eligibility criteria for in-depth interview are as follows:

For peer review only

Male partners

- Inclusion criteria include: (1) randomly selected using electronic random selection techniques; (2) linked to care within 4-months after enrolling in the study (defined as completing the 4-week ART refill appointment) (n=50 respondetns); or (3) did not link to care within 4 months (n=50 respondents)
- Exclusion criteria include: (1) not randomly selected using electronic random selection techniques

Female partners

- Inclusion criteria include: (1) randomly selected using electronic random selection techniques; (2) partners could never be traced for study enrollment; or (3) partners were enrolled but were lost to follow up and unable to be reached again
- Exclusion criteria include: (1) not randomly selected using electronic random selection techniques; (2) partners could be traced for study enrollment; or (3) partners were enrolled and were not lost to follow up and were able to be reached again

4.4.3. *Sampling techniques and tools*

Survey data will include all men and women enrolled in the study (n=1,086; described in detail in Objective 1). 100 men (~ 33 per study arm) and 100 women (~ 33 per study arm) enrolled in the study will be randomly be selected for in-depth interviews.

4.4.4. *Data collection techniques and tools*

Surveys: Baseline and 4-month follow-up surveys will be conducted with men and women enrolled in the trial. They will focus on:

- *Health care system*: perceptions regarding the following aspects of ART services (i) privacy and confidentiality; (ii) availability of services; (iii) wait-time and distance to facility; (iv) quality of care and rude behavior from health care providers, using validated measures.
- *Sociodemographics*: (i) age; (ii) household assets; (iii) work; and (iv) substance use
- *Couple characteristics*: (i) relationship type and length; (ii) sexual activity and risk; (iii) frequency of communication; (iv) disclosure; (v) joint decision making using standard measures from Demographic Health Survey (DHS);[17] (vi) gender norms using validated Gender Equitable Men (GEM) Scale,[67] and (v) Revised Conflict Tactics Scale.[68]
- *Knowledge/perceptions and biomedical factors:* (i) knowledge about HIV and ART (treatment as prevention, benefits of early ART); (ii) risk perception (morbidity and mortality); standard DHS measures on (iii) previous use of HIV services and (iv) self-rated health;[17] and (v) WHO staging at enrollment.

In-depth interviews: Table 5 below describes in-depth interview participants and justification. In-depth interview guides for men and women are developed based on existing literature and our extensive experience conducting in-depth interviews with this population (Appendix J and K). Female partners will provide important insight into the circumstances of these men, and potential strategies to more effectively reach them. Men's qualitative feedback is particularly important for the staged intervention and intensive intervention since these are fairly novel and under explored.

For peer review only

Table 5. *Description of in-depth interview participants and justification*

Participant type	Number of interviews	Justification
Women whose male partners were **unreachable** during the study enrollment	50 (~ 16 per arm)	To understand the couple dynamics and characheristics of men who were never treaced and additional strategies to reaching these men
Women whose male partners were **loss-to-follow-up after** study enrollment	50 (~ 16 per arm)	To understand the couple dynamics and charactersitcs of men who were loss-to-follow-up, and additional strategies to better engage these men in care
Male particiapnts who **did not** complete 4-week ART refill appointments by 4-months	50 (~ 16 per arm)	To understand what they liked and did not like about the intervention, why they did not link to ART, and suggestions on how to improve the intervention
Male participants who completed 4-week ART refill appointments by 4-months	50 (~ 16 per arm)	To understand what they liked and did not like about the intervention, why they linked to ART, and suggestions on how to improve the intervention

4.4.5. Sample size determination

Survey sample size details provided in Objective 1. The number of interviews required for qualitative data can be challenging to predict. Data should be collected until saturation is reached, meaning that no new themes or relevant information is emerging. The exact number of interviews required to reach saturation differs based on the aim of the study, the diversity in respondents, and the theoretical framework used for analysis.[30] However, a basic rule of thumb is that no sample size should be under 25 participants in order to reach saturation and identify all relevant themes or new information important to the study.

4.4.6. Data analysis

Surveys: Subjects with complete data in outcomes as well as predictors will be included in the analysis. We will calculate descriptive statistics, including mean/median, variation (standard deviation, kurtosis), range, and frequency distributions for the demographic and clinical characteristics, overall and by study arm. Logistic models will be developed for the probability of a positive outcome, with sociodemographic factors included as covariates in a suitable form (linear/spline/factor). Differences in the prevalence of each of the outcomes of interest will be examined by study arm as well as by other factors of interest including demographic characteristics (e.g., age), couple chararisterics, and knowledge/perceptions and biomedical knowledge. The differences will be evaluated using t-tests, Mann-Whitney U test (or other non-parametric tests), chi-square methods, and Fisher's exact test as appropriate.

In-depth interviews: Audio recordings of in-depth interviews will be transcribed and translated to English. A preliminary codebook will be developed for both interview types (male and female). Selected investigators will piloted a codebook by independently reading and coding a randomly-selected subset of transcripts. Through an iterative consultative process, each investigator will revised their respective codebook and repeated this process until there was high interrater reliability among the group. All

For peer review only

transcripts will be coded in Atlas.ti v8.3 using constant comparison, and coding disagreements were resolved by consensus.

4.5. OBJECTIVE 3

Determine the cost-effectiveness and scalability of the intervention arms through costing and mathematical modeling.

- **Hypothesis 3.1:** The staged intervention will be more cost effective at having men initiate ART than both the lightest touch intervention and the intensive intervention.

4.5.1. Study design

We will conduct an incremental cost-effectiveness analysis and mathematical modelling to determine national scale-up potential. The average cost per successful outcome (early ART retention) will be calculated and compared across arms incrementally.

4.5.2. Data collection techniques and tools

Costs will be measured from the health care provider. We will use micro-costing methods by first creating an inventory of all the resources used to achieve the observed study outcomes including:

- Standard counseling interactions (staff cadre, training received, duration of interaction and distance from facility travelled where applicable)
- Motivational interviewing interactions (staff cadre, training received, duration of interaction and distance from facility travelled where applicable)
- Provider interactions (staff cadre, training received, duration of interaction and distance from facility travelled where applicable)
- Cost of reminder messages sent (when messages delivered telephonically instead of in person)

For each study patient, the quantity (number of units) of resources used will be determined. Unit costs of resources, which are not human subject data, will be obtained from external suppliers and the site's finance and procurement records and multiplied by the resource usage data to provide an average cost per study patient across centers in each study arm.

4.5.3. Data analysis

Cost-Effectiveness: Using the average cost per patient as described above, we will then estimate the cost per outcome achieved in each arm. The main measure of effectiveness for the cost-effectiveness analysis will be both the primary study outcome (early ART retention). We will calculate the difference in cost divided by the difference in effectiveness among study arms. Costs will be reported as means (standard deviations) and medians (IQRs) in USD, using the exchange rate prevailing during the follow up period.

National scale-up modeling: To determine the budget impact and affordability of the intervention arms, we will parameterize a national scale-up model using the study output. To determine the total cost and impact of the three intervention arms, as well as combinations of interventions, we will model cost and impact out to early ART retention (ART initiation and completion of the 4-week ART refill appointment). The following parameters to be estimated from this trial include:

- Percent of men not linking after HIVST (and thus eligible for this trial)

For peer review only

- Proportion of men that have not linked that could be reached
- Proportion of men that are known HIV-positive and on ART (not disclosed to their partner)
- Proportion of men that initiate ART
- Proportion of men that complete the 4-week ART refill appointment

We will then estimate the expected increase in the number of men linked to ART after index HIVST, adjusted by facility type where possible, by each intervention arm. The number of facility-level HIV tests conducted through index testing at all 652 public healthcare facilities in Malawi from Oct 2019-Sept 2020 will be used for these national calculations. Each intervention will be tested separately in this model, as well as different combination of interventions. Different scenarios will be explored where interventions are used at different facilities (urban versus rural targeting of interventions, geospatial targeting of interventions), or different groups of men within the same facility (where data suggest that different demographics of men resnd differently to the different interventions).

The national-level costs and expected number of men linked to ART, by each intervention and combinations of interventions, will be reported from this model. We will then contextualize the national cost of each intervention with a short-term 3-year budget impact: percent increase (or decrease) of the national HIV treatment budget with the inclusion of one of these interventions

5. ETHICAL CONSIDERATIONS

There is minimal risk associated with the above-mentioned procedures. We have extensive experience measuring ART initiation within HIVST studies. We conducted the first trials in the region to objectively measure ART initiation among men after receiving HIVST through the Index HIVST Trial (PI: Dovel) and PASTAL Trial (male partners of antenatal clients; PI: Choko). We draw from lessons learned from our previous trials.

Informed Consent

Informed consent will be obtained before any study-specific procedures are performed. The informed consent process will include information exchange, detailed discussion, and assessment of understanding of all required elements of informed consent, including the potential risks, benefits, and alternatives to study participation. The process will emphasize the randomized nature of the study and the differences that participants may experience as part of the study relative to current local standards of care. The study will include children 15 years of age and older. Following Malawian protocol, adolescents <18 years of age will be required to attain assent before completing the survey. Based on prior studies, we anticipate <10% of participants to be under 18 years of age, providing a small sample size to explore the potential impact of facility-based testing for youth.

Potential Benefits

Men who participate in the study may have access to additional HIV services not usually provided through routine care, such as appointment reminders, peer navigation, motivational interviewing, and home-based ART initiation. Men can refuse these additional services at any point. Further, both men and women will have the opportunity to discuss their use of HIV services and any concerns with HIV as individuals or as a couple. Information learned in this study may be of benefit to participants and others in the future, particularly information that may lead to optimized testing guidelines.

Potential risks and discomforts

Study procedures have minimal risk to the client. For men, maintaining privacy and confidentiality is a potential risk, particularly with home-based ART initiation. In our prior work delivering routine Index

For peer review only

HIVST, we have had health workers visit a cluster of homes (not just one) to avoid unwanted questions about the individual's serostatus. This has worked quite well, with no reports of unwanted disclosure, and we will use this approach in our proposed study to minimize risk of unwanted disclosure. Further, men may refuse any ART service at anypoint if they are uncomfortable.

For women, increased intimate partner violence (IPV) may be a potential risk, particularly if their male partner is prone to violence. To reduse these risks, women who report IPV with their current partner in the past 12 months will be excluded from the study. Female ART clients who report IPV at anypoint of the intervention will be withdrawn from the study, along with their male partner, counseled, and referred to community-based resources for IPV. We also will provide extensive counseling on status disclosure and an IPV hotline to all female participants. Further, Our PASTAL and Index HIVST Trials show no sign of increased IPV and we have published extensively on risk factors for IPV in other settings.[71-73]

Finally, Participation includes completion of a survey that will assess previous use of health services, perceptions of health services received, and sociodemographic and biomedical factors that may be associated with health service utilization. Participants may feel some psychological stress or discomfort from some of the questions, although most questions are not sensitive in nature. Participants may decline to answer any questions that make them uncomfortable and may end participation at any time.

Reimbursement/compensation

Participants will be provided MK 7,500 (equivalent to 10USD) for each survey completed (MK 15,000 / 20USD across the duration of the study). They will receive the above compensation regardless if they use HIV services or not. Those who complete the additional in-depth interview 6-months after study enrollment will receive an additional MK 7,500 (equivalent to 10USD) for their time.

Privacy and confidentiality

All study procedures will be conducted in private, and every effort will be made to protect participant privacy and confidentiality to the extent possible. Participant information will not be released without written permission to do so except as necessary for review, monitoring, and/or auditing. All study-related information will be stored securely. Participant research records will be stored in locked areas with access limited to study staff. All study data will be identified by participant ID (PID) only. Likewise, communications between study staff and protocol team members regarding individual participants will identify participants by PID only. Process evaluation documents, such as intervention monitoring and evaluation tools, will only include PID and will not store PID and identifiers together. All local databases will be encrypted and secured with password-protected access systems. Lists, logbooks, appointment books, and any other documents that link PID numbers to personal identifying information will be stored in a separate, locked location in an area with limited access. For the intervention, home visits will be conducted by health workers who visit a cluster of homes (not just one) at one time in order to avoid unwanted questions about the individual's serostatus. This has been used in other interventions focused on partner testing and treatment with high success of removing unwanted disclosure to community members.

6. DISSMINATION OF RESULTS

This study will set the stage for interventions that combine HIVST with differentiated models for early ART retention in low-resource settings. The study is timely and of high-impact. Findings will establish the effectiveness of home-based ART among male HIVST users, and can directly inform HIV programs throughout the region. The dissemination plan was developed to achieve the most impact while still ensuring dissemination among local stakeholders who may immediately benefit from study findings.

Partners in Hope is already integrated into national technical working groups, so dissemination will follow standard meeting schedules and draw upon Partners in Hope's longstanding history with the

Ministry of Health. Additionally, we will disseminate results through presentations at international scientific meetings and through high-impact peer-reviewed journals. The mentorship team has extensive experience publishing in high-impact journals (e.g., *AJPH, AIDS, BMJ, Lancet HIV, JAIDS*, *PLOS Med*)

7. PERSONNEL ROLES AND INSTITUTIONS

The proposed research team includes clinical researchers and implimentation science professionals with substantial experience in HIV testing, HIV prevention and treatment, cost effectiveness, differentiated care model studies, and male-focused studies and programs in Malawi and Sub-Saharan Africa. The study will be implemented in partnership with Partners in Hope Medical Center in Lilongwe, which has years of experience collaborating with Ministry of Health and local health facilities on similar studies, mentoring staff, and running studies embedded within routine clinical care.

- Kathryn Dovel, MPH, PhD, Principle Investigator, Division of Infectious Disease University of California Los Angeles (UCLA) and Research Director for Partners in Hope
- Thomas Coates, PhD, Co-Investigator, Division of Infectious Disease UCLA
- Risa Hoffman, MPH, MD, Co-Investigator, Division of Infectious Disease UCLA
- Brooke Nichols, Co-Investigator, School of Global Health, Boston Univeristy
- Lawrence Long, Co-Investigator, School of Global Health, Boston Univeristy
- Alemayehu Amberbir, Co-Investigator, Partners in Hope
- Augustine Choko, PhD, Site Co-Investigator, Malawi Liverpool Wellcome Trust
- Michal Kulich, Biostatistician, Charles Univeristy in Prauge
- Julie Hubabrd, MSc, Study Coordinator, Partners in Hope
- Kelvin Balakasi, Study Data Manager, Parners in Hope
- Khumbo Phiri, Implimentation Science Manager, Partners in Hope

Dr. Kathryn Dovel, the Principle Investigator, is the Science Director at Partners in Hope and an Assitant Assistant Professor in the Division of Infectious Diseases at UCLA. Dr. Dovel has over ten years of experience in Malawi and collaborating with the study team. She is regularly involved in UNAIDS and WHO workshops and meetings regarding strategies for male engagement, and has been a consultant on two Ministry of Health guidelines on the topic in Malawi.

Dr. Augustine Choko will be responsible with Dr. Dovel for overall adherence to the study protocol and serve as the primary liaison with the local IRB and key stakeholders in Malawi. Dr. Thomas Coates will serve as the community-based trials specialist, with over two decades of experience conducting individual- and cluster-randomized trials in communities with the end goal of engagement in HIV services. Dr. Risa Hoffman is an established clinical investigator and will serve as the MD specializing in differentiated models of ART treatment delivery and HIV care, and ensuring client safety. Brooke Nichols and Lawrence Long will be responsible for reviewing all modeling data, making an analysis plan for the proposed models, and providing modeling for publications. Dr. Michal Kulich is the Chair of the Probability and Statistics Department at Charles University and has extensive experience with the design, conduct, and analysis of clinical trials in the context of HIV prevention research.

Partners in Hope's staff Kelvin Balakasi (Data Manager) Julie Hubbard (Research Coordinator), Khumbo Phiri (Implimentation Science Manager) and Alemayehu Amberbir (Science Director) will be responsible implimentation and oversight inlcuidng data collection, data management, quality control, and training and certification of data entry personnel. They will also be responsible for ensuring the intervention promotes client safety, meets Ministry of Health guidelines, and is implemented in such a way to promote sustainability and scalability.

CV's for participating personelle are provided in the Appendix L.

For peer review only - http://bmjopen.bmj.com/site/about/guidelines.xhtml

8. REGULATORY OVERSIGHT

This study is sponsored by the Bill and Melinda Gates Foundation and implemented through Partners in Hope (PIH), Malawi. PIH staff will perform monitoring visits. As part of these visits, monitors will inspect study-related documentation to ensure compliance with all applicable regulatory requirements.
All health facilities will receive an Initial Registration Notification from PIH that indicates successful completion of the protocol registration process. A copy of the Initial Registration Notification will be retained in the site's regulatory files.

We have developed a trial advisory group. See Table 6 for details about the group members. The group will meet every quarter to review progress, and challenges with study implementation, and provide input on the final interventions to be tested, based on qualitative findings in Aim 1.

Table 6. *Description of trial advisory group*

Name	Affiliation	Expertise
Dr. Morna Cornell	University of Cape Town	Epidemiologist, health system barriers to men's care, men's HIV services, advocacy and policy change
Dr. Heidi van Rooyen	SA Human Sciences Research Council	Social scientist, HIV vulnerability and inequality, interventions for men's ART initiation
Dr. Deborah Donnell	University of Washington, Fred Hutch Vaccine and Infectious Disease Division	Biostatistician, international HIV trials, PI of the HPTN Statistical and Data Management Center
Dr. Connie Celum	University of Washington	Infectious disease physician and epidemiologist, implementation science in Africa, HIV prevention trials
Dr. Thoko Kalua	Malawi Ministry of Health, Deputy Director at Department of HIV and AIDS	Epidemiologist. Extensive experience in national HIV programs, M&E, and scale-up of interventions on the ground
Dr. Sergio Chicumbe	Mozambique National Health Institute (INS), Health System Research Cluster	Clinical trials and implementation science. Extensive experience in national public health programs, methodology for health services research and quality care improvement.

For any future protocol amendments, upon receiving final IRB/EC and any other applicable regulatory entity approvals, sites should implement the amendment immediately. Sites are required to submit an amendment registration packet to the PIH Protocol Team. PIH key personnel will review the submitted protocol registration packet to ensure that all the required documents have been received.

9. STUDY IMPLEMENTATION

For peer review only - http://bmjopen.bmj.com/site/about/guidelines.xhtml

Study implementation at each site will be guided site-specific standard operating procedures (SOPs). These SOPs will be updated and/or supplemented as needed to describe roles, responsibilities, and procedures for this study.

10. PROTOCOL DEVIATION REPORTING

All protocol deviations will be documented in participant research records. Reasons for the deviations and corrective and preventive actions taken in response to the deviations will also be documented. Deviations will be reported to site IRBs/ECs and other applicable review bodies in accordance with the policies and procedures of these review bodies. Serious deviations that are associated with increased risk to one or more study participants and/or significant impacts on the integrity of study data must also be reported to the Protocol Team as soon as possible.

11. WORK PLAN TIMELINE

Table 7: *Anticipated workplan timeline of study activities, by year*

IdeAL Study Timeline																
**Activities**	**Year 1**				**Year 2**				**Year 3**				**Year 4**			
Quarter	1	2	3	4	1	2	3	4	1	2	3	4	1	2	3	4
Preparation	X	X	X	X												
Objective 1:3. Test the effectiveness of models of varying intensity to link patients who use HIVST to care																
Enrollment					X	X	X	X								
Intervention and follow-up					X	X	X	X	X	X						
Follow-up analyses & writing										X	X	X	X			
Objective 2: 4. Identify factors associated with early ART retention																
Baseline sociodemographic survey					X	X	X	X								
Qualitative assessment- rolling					X	X	X	X	X	X						
Analyses & writing						X	X			X	X	X	X			
Objective 3: 5. Determine the cost and scalability of the intervention																
Data collection					X	X	X	X	X	X						
Synthesis of results and parameter estimation										X						
Model development										X	X	X				
Analyses & writing										X	X	X	X			

12. BUDGET AND JUSTIFICATION

Table 8: *Study budget*

Description	USD	Justification
Study Coordinator	5000	25% LOE to coordinate RAs
Research Assistant for data collection	15000	5 RAs for 6 months at 500 USD per month
Incentive for participants	21720	10USD per study visit, 2 study visits per participant, 1086 participants (543 men and 543 female partners)

For peer review only

Expert Client/Nurse lunch allowance	720	6USD lunch allowance for 10 Expert Clients and 10 nurses on a monthly basis to hear from them how the intervention is going
Telecomunications	1000	Mobile data collection processing by RAs and communication with coordinator
NHSRC application fee	150	Application fee
Sub Total	*43,590*	
NHSRC 10% fee	4,359	10% contribution fee of study budget
**Grand total**	**47,949**	

For peer review only

13. REFERENCES

1. Dovel K, Yeatman S, Watkins S, Poulin M. Men's heightened risk of AIDS-related death: the legacy of gendered HIV testing and treatment strategies. *AIDS (London, England)*. 2015;29(10):1123.

2. UNAIDS. *Ending AIDS: Progress towards 90-90-90 Targets*.; 2017.

3. Choko AT, Kumwenda MK, Johnson CC, et al. Acceptability of woman-delivered HIV self-testing to the male partner, and additional interventions: a qualitative study of antenatal care participants in Malawi. *Journal of the International AIDS Society*. 2017;20(1):21610.

4. Yeatman S, Chamberlin S, Dovel K. Women's (health) work: A population-based, cross-sectional study of gender differences in time spent seeking health care in Malawi. *PloS one*. 2018;13(12).

5. Mills EJ, Beyrer C, Birungi J, Dybul MR. Engaging men in prevention and care for HIV/AIDS in Africa. *PLoS medicine*. 2012;9(2).

6. Malawi National Statistics Office. *Demographic Health Survey: Malawi*.; 2016.

7. Sharma M, Ying R, Tarr G, Barnabas R. Systematic review and meta-analysis of community and facility-based HIV testing to address linkage to care gaps in sub-Saharan Africa. *Nature*. 2015;528(7580):S77–S85.

8. Partners in Hope. Update on index partner testing in Partners in Hope supported sites: quarterly review. Presented at the: Malawi Ministry of Health Technical Working Group: HIV Self-Testing; 2019.

9. Dovel K, Balakasi K, Shaba F, Phiri K, Offorjebe O, Gupta S. A Randomized trial on index HIV self-testing for partners for ART clients in Malawi. In: *Conference on Retroviruses and Opportunistic Infections, Seattle*. ; 2019.

10. Johnson CC, Kennedy C, Fonner V, et al. Examining the effects of HIV self-testing compared to standard HIV testing services: a systematic review and meta-analysis. *Journal of the International AIDS Society*. 2017;20(1):21594.

11. Masters SH, Agot K, Obonyo B, Mavedzenge SN, Maman S, Thirumurthy H. Promoting partner testing and couples testing through secondary distribution of HIV self-tests: a randomized clinical trial. *PLoS medicine*. 2016;13(11).

12. Ortblad K, Musoke DK, Ngabirano T, et al. Direct provision versus facility collection of HIV self-tests among female sex workers in Uganda: a cluster-randomized controlled health systems trial. *PLoS medicine*. 2017;14(11).

13. MacPherson P, Lalloo DG, Webb EL, et al. Effect of optional home initiation of HIV care following HIV self-testing on antiretroviral therapy initiation among adults in Malawi: a randomized clinical trial. *Jama*. 2014;312(4):372–379.

14. Sibanda EL, Weller IV, Hakim JG, Cowan FM. The magnitude of loss to follow-up of HIV-exposed infants along the prevention of mother-to-child HIV transmission continuum of care: a systematic review and meta-analysis. *AIDS (London, England)*. 2013;27(17):2787.

For peer review only

15. Larsson EC, Thorson A, Nsabagasani X, Namusoko S, Popenoe R, Ekström AM. Mistrust in marriage-Reasons why men do not accept couple HIV testing during antenatal care-a qualitative study in eastern Uganda. *BMC public health*. 2010;10(1):769.

16. Koo K, Makin JD, Forsyth BW. Barriers to male-partner participation in programs to prevent mother-to-child HIV transmission in South Africa. *AIDS Education and Prevention*. 2013;25(1):14–24.

17. Theuring S, Nchimbi P, Jordan-Harder B, Harms G. Partner involvement in perinatal care and PMTCT services in Mbeya Region, Tanzania: the providers' perspective. *AIDS care*. 2010;22(12):1562–1568.

18. Sileo KM, Fielding-Miller R, Dworkin SL, Fleming PJ. What role do masculine norms play in men's HIV testing in sub-Saharan Africa?: a scoping review. *AIDS and Behavior*. 2018;22(8):2468–2479.

19. van den Berg W, Brittain K, Mercer G, et al. Improving men's participation in preventing mother-to-child transmission of HIV as a maternal, neonatal, and child health priority in South Africa. *PLoS medicine*. 2015;12(4).

20. Unaids HIV. *Addressing a Blind Spot in the Response to HIV—Reaching out to Men and Boys*. Joint United Nations Programme on HIV/AIDS Geneva; 2017.

21. Cornell M, Cox V, Wilkinson L. Public health blindness towards men in HIV programmes in Africa. *Tropical Medicine & International Health*. 2015;20(12):1634–1635.

22. Poulin M, Dovel K, Watkins SC. Men with money and the "vulnerable women" client category in an AIDS epidemic. *World Development*. 2016;85:16–30.

23. Pai NP, Sharma J, Shivkumar S, et al. Supervised and unsupervised self-testing for HIV in high-and low-risk populations: a systematic review. *PLOS medicine*. 2013;10(4).

24. Sharma M, Barnabas RV, Celum C. Community-based strategies to strengthen men's engagement in the HIV care cascade in sub-Saharan Africa. *PLoS medicine*. 2017;14(4).

25. Choko AT, MacPherson P, Webb EL, et al. Uptake, accuracy, safety, and linkage into care over two years of promoting annual self-testing for HIV in Blantyre, Malawi: a community-based prospective study. *PLoS medicine*. 2015;12(9).

26. Decroo T, Rasschaert F, Telfer B, Remartinez D, Laga M, Ford N. Community-based antiretroviral therapy programs can overcome barriers to retention of patients and decongest health services in sub-Saharan Africa: a systematic review. *International health*. 2013;5(3):169–179.

27. Wools-Kaloustian KK, Sidle JE, Selke HM, et al. A model for extending antiretroviral care beyond the rural health centre. *Journal of the International AIDS Society*. 2009;12(1):22–22.

28. Apondi R, Bunnell R, Awor A, et al. Home-based antiretroviral care is associated with positive social outcomes in a prospective cohort in Uganda. *JAIDS Journal of Acquired Immune Deficiency Syndromes*. 2007;44(1):71–76.

29. Jaffar S, Amuron B, Foster S, et al. Rates of virological failure in patients treated in a home-based versus a facility-based HIV-care model in Jinja, southeast Uganda: a cluster-randomised equivalence trial. *The Lancet*. 2009;374(9707):2080–2089.

For peer review only

30. Malterud K, Siersma VD, Guassora AD. Sample size in qualitative interview studies: guided by information power. *Qualitative health research*. 2016;26(13):1753–1760.

For peer review only

14. APPENDIX

14.1. APPENDIX A – Study Invitation Cards

STUDY INVITATION CARD
Identifying efficient linkage strategies for HIV self-testing (IDEaL)
English

Study invitation Card

Date: ……/……/2020

Dear: ____

At __________________________________, we are conducting a study. The study will generate information that will help the Malawi government, Partners in Hope and other stakeholders develop programs that will help your community lead a healthy life. We are therefore inviting you and your partner to come to ___________________________ on__________________________ at_________, so that we can assess if you are eligible for the study. If eligible, you and your partner will each receive MK 7,500 (equivalent to 10USD).

Thank you for your kind attention to this request and we look forward to seeing you. When coming call or flash this number…………………… so that you are met by study personnel.

Sincerely,

[District Health Officer]

For peer review only

STUDY INVITATION CARD
Identifying efficient linkage strategies for HIV self-testing (IDEaL)
Chichewa

Khadi Lokuyitanani Ku Kafukufuku

Tsiku: ……/……/2020
Wokondedwa: ____

Ku chipatala cha, __________________________________, tikupanga kafukufuku. Kafukufukuyu adzathandiza kupeza mfundo zomwe zidzathandize boma la Malawi, bungwe la Partners In Hope ndi mabungwe/magulu ena kukhazikitsa ndondomeko zomwe zidzathandizire anthu a mdela lanu kukhala ndi umoyo wanthanzi. Choncho mukuyitanidwa pamodzi ndi okondedwa anu kuti mubwere ku_______________ tsiku nthawi ya ____________, ndicholinga choti tidzaone ngati muli oyenera kutenga nawo mbali mu kafukufukuyu. Mukadzapezeka kuti ndinu oyenera kutenga nawo mbali mu kafukufukuyu, inuyo komanso bwenzi lanu mudzalandira K7,500 (yokwana 10USD) aliyense.

Zikomo kwambiri chifukwa cha chidwi chanu pa pempholi ndipo ife tikuyembekezera kuzaonana nanu pa tsikuli. Mukamadzabwera, muzaimbe kapena kufulasha pa nambala iyi…………………………………kuti muzakumane ndi anthu a kafukufukuyu.

Ndine wanu,

[District Health Officer]

For peer review only - http://bmjopen.bmj.com/site/about/guidelines.xhtml

14.2. APPENDIX B – Recruitment and Screening Script

RECRUITMENT AND SCREENING SCRIPT
Identifying efficient linkage strategies for HIV self-testing (IDEaL)
Female

Thank you for speaking with me about the study entitled, *"Identifying efficient linkages strategies for HIVST"* conducted by Partners in Hope and the University of California Los Angeles in the United States. You are being approached because you recently reported that your male partner tested HIV-positive using a HIV self-test kit.

The purpose of this is to determine what are the best interventions that can help men who are diagnosed with HIV use other health services, if desired. The study will offer several different strategies for HIV services to see what works best for men who use HIV self-testing kits.

Would you be interested in participating in the eligibility screening to see if you are eligible to participate in the study? Your participation is voluntary and you will not be penalized if you choose not to participate in the screening or the project.

[*If no, thank the person and end the session*]

[*If yes, continue to the screening questions below or make an appointment to complete the screening questions*]

Before we continue with the study, we first need to determine if you are eligible to participate.

1. Are you and your partner 15 years old or older?
2. To the best of your knowledge, did your partner recently test HIV-positive using a HIV self-test kit?
3. To the best of your knowledge, your partner currently NOT taking ART?
4. Does your partner live in the facility catchment area?
5. In the last 30 days, your current partner has NEVER hit, slapped, or kicked you, or forced you to have sexual intercourse with them?

If you answered yes to all these questions, then you are eligible to participate in the study. You may stay here to continue with the study consent, and we will explain how the study will be conducted.

For peer review only

RECRUITMENT AND SCREENING SCRIPT
Identifying efficient linkage strategies for HIV self-testing (IDEaL)
Female (Chichewa)

Zikomo kwambiri povemera kucheza nane zokhudza study yotchedwa "Kupeza njira zabwino zothandizira anthu omwe akulandira ma ARV" amene akupangidwa ndi bungwe la Partners In Hope-EQUIP polojekiti ndi sukulu ya ukachenjede ya University of California Los Angeles yaku Amerika. Mukufunsidwa kuti mutenge nawo mbali mu gawo lakafukufukuyu chifukwa bwenzi lanu lalimuna lili ndi kachirombo ka HIV ndipo linadziwa zotsatirazi pogwiritsa ntchito ka chida koziyezera wekha.

Cholinga cha kafukufukuyu ndi kufuna kupeza njira zabwino zomwe zingathandize azibambo omwe ali ndi kachirombo ka HIV kugwiritsa ntchito thandizo lina la zaumoyo, ngati akonda kutero. Studyyi idzapeleka njira zingapo zosiyanasiyana za thandizo la HIV kuti awone njira yomwe ikugwira bwino kwa azibambo omwe amagwiritsa ncthito ka chida koziyezera wekha HIV.

Kodi muli okondwa kutenga nawo mbali mu mayele ofuna kuwona ngati muli oyenela kutenga nawo mbali mu study? Kutenga nawo mbali kwanu ndi kosakakamiza ndipo palibe chilango chilichonse ngati mungasankhe kusatenga nawo mbali mu mayele a study.

[Ngati ayi, thokozani munthuyo ndipo malizani session]

[Ngati eya, pitilizani kufunsa mafunso a mayele omwe ali munsiwa kapena sankhani tsiku loti muzamalize kufunsa mafunso]

Tisanapitilize ndi study, choyamba tifuna tidziwe ngati muli oyenera kutenga nawo mbali.

1. *Muli ndi zaka khumi ndi zisanu kapena kuposela apo?*
2. *Monga mukudziwira kodi bwenzi lanu laziyezera kachida koziyezera wekha ndikupezeka ndi kachirombo ka HIV?*
3. *Monga mene mukudziwira, kodi pakadali pano bwenzi lanu likumwa mankhwala a ma ARV?*
4. *Kodi bwenzi lanu limakhala mu dela lozungulira chipatala?*
5. *M'masiku makumi atatu apitawa, bwenzi lanu SILINAPANGE izi kumenyani kapena kukukakamizani kuti mugonane nalo?*

Ngati mwayankha eya pa mafunso onse mwafunsidwawa, zikusonyeza kuti ndinu oyenera kutenga nawo mbali mu study. Muli omasuka kukhala ndikupitiliza chilolezo cha study, ndipo ndikufotokozerani za momwe study ichitikire.

For peer review only - http://bmjopen.bmj.com/site/about/guidelines.xhtml

RECRUITMENT AND SCREENING SCRIPT
Identifying efficient linkage strategies for HIV self-testing (IDEaL)
Male

Thank you for speaking with me about the study entitled, *"Identifying efficient linkages strategies for HIVST"* conducted by Partners in Hope and the University of California Los Angeles in the United States. You are being approached because you recently reported testing HIV-positive using a HIV self-test kit.

The purpose of this is to determine what are the best interventions that can help men who are diagnosed with HIV use other health services, if desired. The study will offer several different strategies for HIV services to see what works best for men who use HIV self-testing kits.

Would you be interested in participating in the eligibility screening to see if you are eligible to participate in the study? Your participation is voluntary and you will not be penalized if you choose not to participate in the screening or the project.

[*If no, thank the person and end the session*]

[*If yes, continue to the screening questions below or make an appointment to complete the screening questions]*

Before we continue with the study, we first need to determine if you are eligible to participate.

1. Are you 15 years old or older?
2. You recently test HIV-positive using a HIV self-test kit?
3. You are NOT currently taking ART?
4. Do you live in the facility catchment area?

If you answered yes to all these questions, then you are eligible to participate in the study. You may stay here to continue with the study consent, and we will explain how the study will be conducted.

For peer review only

RECRUITMENT AND SCREENING SCRIPT
Identifying efficient linkage strategies for HIV self-testing (IDEaL)
Male (Chichewa)

Zikomo kwambiri povemera kucheza nane zokhudza study yotchedwa "Kupeza njira zabwino zothandizira anthu omwe akulandira ma ARV" amene akupangidwa ndi bungwe la Partners In Hope-EQUIP polojekiti ndi sukulu ya ukachenjede ya University of California Los Angeles yaku Amerika. Mukufunsidwa kuti mutenge nawo mbali mu gawo lakafukufukuyu chifukwa muli ndi kachirombo ka HIV ndipo munadziwa zotsatirazi pogwiritsa ntchito ka chida koziyezera wekha.

Cholinga cha kafukufukuyu ndi kufuna kuoeza njira zabwiino zomwe zingathandize azibambo omwe ali ndi kachirombo ka HIV kugwiritsa ntchito thandizo lina la zaumoyo, ngati akonda kutero. Studyyi idzapeleka njira zingapo zosiyanasiyana za thandizo la HIV kuti awone njira yomwe ikugwira bwino kwa azibambo omwe amagwiritsa ncthito ka chida koziyezera wekha HIV.

Kodi muli okondwa kutenga nawo mbali mu mayele ofuna kuwona ngati muli oyenela kutenga nawo mbali mu study? Kutenga nawo mbali kwanu ndi kosakakamiza ndipo palibe chilango chilichonse ngati mungasankhe kusatenga nawo mbali mu mayele a study.

[Ngati ayi, thokozani munthuyo ndipo malizani session]

[Ngati eya, pitilizani kufunsa mafunso a mayele omwe ali munsiwa kapena sankhani tsiku loti muzamalize kufunsa mafunso]

Tisanapitilize ndi study, choyamba tifuna tidziwe ngati muli oyenera kutenga nawo mbali.

1. *Muli ndi zaka khumi ndi zisanu kapena kuposela apo?*
2. *Kodi mwaziyezera kachida koziyezera wekha ndikupezeka ndi kachirombo ka HIV?*
3. *Kodi pakadali pano mukumwa mankhwala a ma ARV?*
4. *Kodi mumakhala mu dela lozungulira chipatala?*

Ngati mwayankha eya pa mafunso onse mwafunsidwawa, zikusonyeza kuti ndinu oyenera kutenga nawo mbali mu study. Muli omasuka kukhala ndikupitiliza chilolezo cha study, ndipo ndikufotokozerani za momwe study ichitikire.

For peer review only

14.3. APPENDIX C – Written Informed Consent- Female

WRITTEN INFORMED CONSENT
Identifying efficient linkage strategies for HIV self-testing (IDEaL)
Female

You are asked to participate in a research study entitled *"Identifying efficient linkages strategies for HIVST"* conducted by Partners in Hope and the University of California Los Angeles in the United States. You are being requested to take part in the study because you recently reported that your male partner tested HIV-positive using a HIV self-test kit. Your participation in this study is entirely voluntary. You will be read the information below, and you are free to ask questions about anything you do not understand, before deciding whether or not to participate. ***I as the field assistant for this study will take you through this consenting process.***

- **Why is this study being done?**

HIV self-testing is very helpful for people who want to know their status but do not usually go to the health facility. However, it can be hard for individuals who test HIV-positive with HIV self-testing to be able to access other health services. Researchers want to determine what are the best interventions that can help men who are diagnosed with HIV use other health services, if desired. The study will offer several different strategies for HIV services to see what works best for men who use HIV self-testing kits.

- **What will happen if you take part in this research study?**

There are several steps to this study. if you volunteer to participate in this study you will have the opportunity to participate in the following components:

1. Allow me to trace your male partner, or take me to your male partner in order to invite him to participate in the study as well. Note, you can choose to participate in the study even if your partner refuses or you think your partner would refuse.

2. Complete one or two study visits where a research assistant like myself will interview you and ask you information about yourself, including whether you are married, number of sexual partners, your level of education, information about your experiences with HIV services, and how you feel about HIV testing and treatment services. We will ask you questions today (or a day nearby that is convenient for you) and, if your partner enrolls in the study, we will ask you similar questions again in four months in order to see if anything has changed. Each interview will last about 45minutes. You can refuse a follow-up survey at any point

3. If your partner agrees to participate in the study, he will be randomized to one of three interventions. We will do the randomization together with him so he can see exactly what intervention he will be offered. The potential interventions are:

   1) Standard of care where providers may send him reminders about the benefits of health services.
   2) Motivational Interviewing where he can talk to someone about his life, challenges he faces, and strategies to make his life better and additional services as needed.
   3) Home-based health services whereby a provider will offer him HIV services and NCD screening at your home as a one-time event. He will then be visited after 4-weeks to be escorted to the clinic if desired.

   Regardless of what arm your partner is randomized to, he can always refuse health services or refuse talking to a health care provider and still remain in the study. You can remain in the study regardless of what your partner does.

4. Finally, you may be randomly selected to within 6-months of the study to complete a 1-hour in-depth interview so we can learn more about your experiences in the study. Not all participants will be contacted for the interview and you always have the right to decline an in-depth interview – refusal will not affect your participation in the larger study.

- **How long will you be in the research study?**

All study activities will be completed within 6-months of today.

- **Are there any potential risks or discomforts that you can expect from this study?**

You will be asked a series of questions by a research assistant about your sexual relationship and your perceptions of your partners use of HIV and other health services. We will NEVER disclose your HIV status to your partner. We will NEVER disclose to your partner that you told us he had tested HIV+. However, you may feel uncomfortable answering some questions asked during the interview or you may feel comfortable having your partner in the study. You are able to withdraw from the study at any time. During an interview you can say "I don't want to answer" to any questions that make you uncomfortable. All questions will be asked in a private place so that no one else will hear your answers.

If you experience distress or adverse events as a result of the study, we will provide you with counseling resources or refer you to resources for assistance.

- **Are there any potential benefits to participating?**

You will have the opportunity to discuss information about your well-being, your relationship with your partner, and HIV services for men with a Research Assistant in a confidential, private manner.

- **Are there any potential benefits to society?**

Information obtained as part of this work may be of benefit to the larger Malawi program, or similar programs in sub-Saharan Africa, since the work aims to determine if there are better ways to offer HIV services to men who use HIV self-test kits. If researchers better understand what

For peer review only

type of programs work better for men, the program in Malawi can be scaled up and strengthened to provide these specific types of increased support.

- **Will you receive payment for being part of this study?**

Your participation is entirely voluntary. You will be provided MK 7,500 (equivalent to 10USD) for each survey completed (MK 15,000 / 20USD across the duration of the study). You will receive the above compensation regardless if you use HIV services or not. Those who complete the additional in-depth interview 6-months after study enrollment will receive an additional MK 7,500 (equivalent to 10USD) for their time.

- **What is the cost of participating in this study?**

There is no cost to participate in this study.

- **Will information about me be kept confidential?**

The study team are the only people who will know about you or any information that you provide in this study. If necessary to protect your rights or welfare (for example, if you are injured and need emergency care) or if required by Malawian law, specific information about you may be made available to providers or officials.

Authorized representatives of the Malawi National Health Sciences Research Council who are responsible for ensuring the rules related to research are followed, may need to review records of study participants. As a result, they may see your name; but they will not to reveal your identity to others.

When the results of the research are published or discussed in meetings, no information will be included that would reveal your identity. Any paperwork related to the study which contains information about you will be kept in a locked cabinet in a locked office. Only staff members of the study will have access to this information. A code will be assigned to each individual participating in the study. This code will be stored on a computer in a locked file. The key to unlock the information will only be known by the research staff. All data entered into a computer will be entered using this code so information will no longer have any information that can identify you such as your name. Forms containing any identifying information will be destroyed two years after the study is finished.

- **Participation and Withdrawal**

Your participation in this research is VOLUNTARY. If you choose not to participate, that will not affect your relationship with the hospital, your health provider or health centre you usually get your medical care from, or your right to health care. If you decide to participate, you are free to withdraw your consent and stop your participation at any time and can still receive future health care at the hospital or health center you go to.

- **Withdrawal of Participation by the Investigator**

The research investigator may stop your participation in this research if he or she feels this is best for you. The investigators will make the decision and let you know if it is not possible for you to continue. The decision may be made to protect your health and safety.

For peer review only

- **Who can answer questions I might have about this study?**

In the event of a research related injury or if you experience a problem, please immediately return to the hospital or health centre you go to or contact Khumbo Phiri. The NHSRC Ministry of Health information (Dr. Mitambo) is also provided in case you have questions about your rights as a research participant.

Kusiyitsidwa kutenga nawo mbali mu kafukufuku ndi wakafukufuku

Anthu opangitsa kafukufukuyu akhonza kukuletsani kutenga nawo mbali mukafukufukuyu akaona kuti ndi bwino kuti mutero. Anthu akafukufukuwa azapanga chiganizochi ndikukudziwitsani kuti sizitheka kuti mupitirize. Chiganizochi chitha kupangidwa kuti ateteze thanzi ndi chitetezo chanu.

Investigator

Khumbo Phiri
Mobile: +265999840946
Partners in Hope Clinic
Area 36, Plot8
M1 Road South
Lilongwe, Malawi

OR

Dr.C. Mitambo
The Secretariate, NHSRC
Ministry of Health
P.O Box 30377
Lilongwe 3
Cell +265888344 443

SIGNATURE OF RESEARCH SUBJECT [OR LEGAL REPRESENTATIVE]

I have read (or someone has read to me) the information provided above. I have been given an opportunity to ask questions and all of my questions have been answered to my satisfaction. I have been given a copy of this form.

Ndawerenga (kapena munthu wina wandiwerengera) zonse zalembedwa mwambamu. Ndapatsidwa mwayi wofunsa mafunso ndi mafunso onse ndinafunsa ayankhidwa ndipo ndakhutusidwa. Ndapasidwa pepala ina yangati yomweyi.

BY SIGNING THIS FORM, I WILLINGLY AGREE TO PARTICIPATE IN THE RESEARCH:

____

Name of Subject

Fingerprint

____

Name of Legal Representative (if applicable)

For peer review only

_____________________________________ DATE (DAY/MO/YR): _________________

Signature of Subject or Legal Representative
(may place an X OR fingerprint if unable to sign)

SIGNATURE OF INVESTIGATOR OR DESIGNEE

I have explained the research to the subject or his/her legal representative and answered all of his/her questions. I believe that he/she understands the information described in this document and freely consents to participate.

____

Name of Investigator or Designee

______________________________ ___________________________

Signature of Investigator or Designee Date (must be the same as subject's)

For peer review only

WRITTEN INFORMED CONSENT
Identifying efficient linkage strategies for HIV self-testing (IDEaL)
Female (Chichewa)

Mukufunsidwa kuti mutenge nawo mbali mu gawo lino la kafukufuku wotchedwa"kupeza njira zoyenera zobwezeletsa anthu pa thandizo la ma ARV" amene akupangidwa ndi bungwe la Partners In Hope-EQUIP polojekiti ndi sukulu ya ukachenjede ya University of California Los Angeles yaku Amerika. Mukufunsidwa kuti mutenge nawo mbali mu gawo lakafukufukuyu chifukwa bwenzi lanu lalimuna linapezeka ndi kachirombo ka HIV pogwiritsa ntchito kachida koziyezela wekha HIV. Kutenga nawo mbali mukafukufuku ameneyi sikokakamiza. Tikuwerengerani uthenga omwe walembedwa pansipa, ndipo muli ololedwa kufunsa mafunso aliwonse pa zomwe simukumvetsetsa,musanapange chiganizo chotenga nawo mbali kapena ayi. ***Ine ngati othandizira mukafufuku ameneyu ndikuthandizani pa ndondomeko yotenga chilolezo***.

- **Chifukwa chiyani kafukufukuyu akuchitika?**

Kuziyeza wekha HIV ndi kofunika kwa anthu omwe akufuna kudziwa za momwe mthupi mwawo mulili ku mbali ya HIV koma sapita kuchipatala, kotelo, ndikovuta kwa anthu omwe apezeka ndi HIV kudzela chipangizo choziyezela wekha kuti apeze thandizo la zaumoyo. Akafukufuku akufuna apeze njira zabwino zomwe zingathe kuthandiza azibambo omwe apezeka ndi HIV kuti agwiritse ntchito thandizo lina lazaumoyo ngati angakonde kutero. Kafukufukuyu apeleka mwayi wa njira zosiyana siyana zothandizira HIV kuti aone zomwe zingagwire ntchito bwino kwa azibambo omwe amagwiritsa ntchito ka chida koziyezela wekha HIV.

- **Chichitike ndi chani mukatenga nawo mbali mu kafukufukuyu?**

Pali ma gawo angapo omwe adzachitike mukafukuku, Ngati mungazipereke kutenga nawo mbali mu kafukufukuyu mudzatenga nawo mbali mu magawo otsatirawa:

1. Mundilole kuti ndifufuze bwenzi lanu lalimuna kapena ndipelekezeni kwa bwenzi lanu lalimuna ndi cholinga choti nalonso litenge nawo gawo mu kafukufuku. Chidziwitso: muli omasuka kutenga nawo mbali mu kafukufu ngati bwenzi lanu lakana kutenga nawo mbali kapena mukuganiza kuti bwenzi lanu likana.

2. Pamapepo pa kucheza koyamba komanso kachiwiri othandiza kafukufuku ngati ine ndidzacheza nanu ndikukufunsani mafunso okhuza inuyo, kuphatikizapo ngati muli pa banja, muli ndi abwenzi ogonana nawo angati, maphunziro anu komanso zomwe munakumana nazo polandira thandizo la HIV komanso momwe mumnvera kumbali ya thandizo la HIV angakhalenso kuyezedwa HIV. Tikufunsani mafunso lelo (Ngati lelo muli okonzeka kuyankha mafuns)komanso miyezi inayi ikudzayi kuti tiwone ngati pali chomwe chasintha, kucheza kuli konse kuzitenga nthawi yosachepela makumi anayi ndi isanu. Muli ololedwa kukana kutenga nawo mbali mu kucheza kotsatira nthawi iliyonse.

3. Ngati bwenzi lanu lidzatenge nawo mbali mu study, adzaikidwa mu gulu limodzi mwa magulu atatu mwa mayere. Tidzachita mayere limodzi ndi bwenzi lanu kuti awone kuti ali 'gulu liti mwa magulu atatuwa. Maguluwa ali motere:

1) Chikumbutso cha ubwino wa thandizo la zaumoyo
2) Kucheza kwa chilimbitso komwe mungathe ndi mwayi ocheza ndi anthu ena ndi kuwafotokozela za umoyo wanu, zofuta zomwe mumakumana nazo komanso njira zomwe mumagwiritsa ntchito kuti moyo wanu ukhale wosavuta.
3) Thandizo la zaumoyo lomwe mumatha kulandira pakhomo monga thandizo la HIV komanso NCD lomwe mumalandila kamodzi.Pakatha masaba anayi mudzayendeledwa ndi wa zaumoyo yemwe adzakupelekezeni ku chipatala komwe mukapitilize kulandira thandizo ngati mwakonda kutelo.

Posatengera njira yomwe mwapatsidwa mongathe kukana kulandira thandizo la zaumoyo koma ndikupitiliza kutenga nawo mbali mu kafukufuku ndipo tingathe kupitilizabe kucheza komwe tatchula m'mwambamu.

4. Pamapeto tidzakuyendelani pakutha kwa miyezi isanu ndi umodzi ya kafukufuku kuti tidzacheze nanu komanso kuti tidzanve za momwe mukunvera za kafukufuku, maganizo anu okhudza njira zina za mtsogolo komanso, thandizo lina lowonjezera ngati ilipo. Si onse otenga nawo mbali omwe adzaonedwe ndipo muli ololedwa kukana kutenga nawo mbali mukucheza ndipo kukana kwanu sikudzaononga mwayi wanu otenga nawo mbali mu study.

- **Mutenga nthawi yaitali bwanji muli mukafukufuku?**

Zochitika zonse zakafukufuku zizamalizidwa mu miyezi isanu ndi imodzi.

- **Pali zinthu zosowetsa mtendere kapena zodetsa nkhawa zomwe mungayembekezere kuchokera mu kafukufukuyu?**

Mufunsidwa mafunso angapo ndi opangitsa kafukufuku okhudza maubwenzi anu ogonana komanso maganizo anu pa momwe abwenzi anu amagwiritsira thandizo la HIV ndi mathandizo ena a zaumoyo. SITIDZAULULA momwe mthupi mwanu muliri kumbali ya kachilombo ka HIV kwa bwenzi lanu. SITIDZAULURA kwa bwenzi lanu kuti munatiuza kuti ali ndi kachilombo ka HIV. Ngakhale zili choncho, mukhoza kusamasuka kuyankha mafunso ena mu kafukufuku kapenanso mukhoza kufuna kuti bwenzi lanu likhale nanu poyankha mafunsowa. Muli ndi ufulu osiya kutenga nawo gawo mu kafukufuku nthawi iliyonse. Mkati mwa kucheza kwathu, mukhoza kunena kuti "Sindikufuna kuyankha" ku funso lililonse lomwe sindinu omasuka kuyankha. Mafunso onse afunsidwa malo oduka mphepo kuti munthu wina aliyense asamve mayankho anu.

Ngati mungapeze mavuto kapena nkhawa mu mtima kamba kotenga nawo gawo mu kafukufukuyu, tikupatsani uphungu oyenera kapena kukulozerani koyenera kupeza thandizo.

- **Pali cholowa chilichonse potenga nawo mbali mukafukufukuyu?**

Mudzakhala ndi mwayi okambilana ndikunva zambiri zokhudza moyo wanu komanso thandizo la HIV ndi othandiza kafukufuku komanso dotolo munjira yachinsisi. Mudzakhalanso ndi mwayi oyamba mankhwala a ma ARV ku chipatala cha kufuna kwanu.

- **Pali cholowa chilichonse kwa anthu a mudera?**

Uthenga womwe udzatengedwe ngati mbali imodzi yakafukufukuyu uzakhala othandiza mu mapologalamu a dziko la Malawi, kapena ma pologalamu ena ofananirapo a kum'mwera kwa Africa, chifukwa choti ntchito imeneyi ikufuna kuona ngati pali njira yabwino yopeleka thandizo la HIV mwa

For peer review only

azibambo, ma pologalamu aku Malawi azapita patsogolo ndikulimbikitsa njira zopititsira patsogolo.

- **Kodi mulandira malipiro potenga nawo mbali mukafukufukuyu?**

Kutenga nawo mbali kosakakamiza. Mudzalandira chiongola dzanja cha ndalama zokwana 7,500(Pafupifupi MK 15,000/20USD). Mudzalandila ndalamazi olo mutakhala kuti simunatenge nawo kapena mwatenga nawo mbali mu thandizo la HIV. Kwa omwe adzamalize nawo kucheza kowonjezera patatha miyezi isanu ndi umodzi adzalandira ndalama yowonjezera yokwana MK 7,500 (yokwana pafupi fupi 10 USD0 chifukwa cha nthawi yawo.

- **Kodi pali kulipira kulikonse chifukwa chotenga nawo mbali mukafukufukuyu?**

Kutenga nawo mbali mukafukufukuyu ndi kwaulere.

- **Kodi uthenga wanga uzasungidwa mwa chinsinsi?**

Anthu ogwira nawo ntchito mu kafukufuku okhawo ndi amene adziwe za uthenga wanu kapena chilichonse chomwe mutiuze pa kafukufukuyu.

Anthu ovomerezeka oyimirira bungwe la National Health Sciences Research Council ndi UCLA office for Protection Of Research Subjects ndi amene ali ndi udindo woonetsetsa kuti malamulo a kafukufuku akutsatidwa, akhoza kufuna kuona nawo kaundula wa anthu amene akutenga nawo mbali mukafukufukuyu. Kutanthauza kuti akhonza kuzaona dzina lanu; koma sangaulure zokhuza inu kwa anthu ena.

Pa nthawi imene zotsatira za kafukufukuyu zidzatsindikidwa kapena kukambidwa mu misonkhano, palibe uthenga womwe udzayikidwe wokuzindikiritsani. Uthenga uliwonse wolembedwa pa pepala wokhunza inu uzasungidwa mu kabati yokhoma mu ofesi yokhomanso. Anthu ogwira nawo ntchito mukafukufukuyu okhawo ndi amene azathe kuona uthenga umenewu. Njira yotanthauzira uthenga umenewu izadziwika ndi anthu akafukufukuyu basi. Uthenga onse olowetsedwa pa makina a kompyuta uzalowetsedwa kugwiritsa ntchito nambala, ndekuti uthenga onse sudzakhalanso ndi zokuzindikiritsani monga dzina lanu. Ma pepala amene pali uthenga okuzindikitsani adzawonongedwa pakapita zaka ziwiri chimalizireni kafukufukuyu.

- **Kutenga mbali ndi kusiya kutenga mbali**

Kutenga nawo mbali mu kafukufukuyu SIKOKAKAMIZA. Ngati musankhe kuti simutenga nawo mbali, izi sizingasokoneze ubale wanu ndi chipatala chino, anthu ogwira ntchito kuchipatala,kapena chipatala chomwe mumalandilirako chithandizo, kapena ufulu wanu wolandira thandizo la chipatala. Ngati mwapanga chisankho chotenga nawo mbali, mukhoza kuchosa chilolezo chanu ndikusiya kutenga nawo mbali nthawi ina iliyonse ndipo mungathe kuzalandirabe chithandizo pa chipatala pano mtsogolo.

- **Kusiyitsidwa kutenga nawo mbali mu kafukufuku ndi wakafukufuku**

Anthu opangitsa kafukufukuyu akhonza kukuletsani kutenga nawo mbali mukafukufukuyu akaona kuti ndi bwino kuti mutero. Anthu akafukufukuwa azapanga chiganizochi ndikukudziwitsani kuti sizitheka kuti mupitirize. Chiganizochi chitha kupangidwa kuti ateteze thanzi ndi chitetezo chanu.

- **Angayankhe ndi ndani mafunso amene ndingakhale nawo wokhuza kafukufukuyu?**

Zitachitika kuti mwavulala kamba ka kafukufukuyu kapena mukukumana ndi vuto,chonde bwelerani mwachangu kuchipatala chomwe munapitako kapena lumikizanani ndi Mike Nyirenda. Bungwe la NHSRC Ministry of Health information kudzera mwa Dr Kathyola liliponso ngati mungakhale ndi mafunso okhudza ufulu wanu ngati munthu wotenga nawo mbali mu kafukufuku.

Investigator
Khumbo Phiri
Mobile: +265999840946
Partners in Hope Clinic
Area 36, Plot8
M1 Road South
Lilongwe, Malawi

OR

Dr.C. Mitambo
The Secretariate, NHSRC
Ministry of Health
P.O Box 30377
Lilongwe 3
Cell +265888344 443

SAYINI YA OTENGA NAWO MBALI [KAPENA YA OYIMIRA OTENGA NAWO MBALI]

Ndawerenga (kapena munthu wina wandiwerengera) zonse zalembedwa mwambamu. Ndapatsidwa mwayi wofunsa mafunso ndi mafunso onse ndinafunsa ayankhidwa ndipo ndakhutusidwa. Ndapasidwa pepala ina yangati yomweyi.

POSAYINA PA PEPALALI, NDIKUVOMERA MOSAKAKAMIZIDWA KUTENGA NAWO MBARI MU KAFUKUFUKU:

____

Name of Subject

____

Name of Legal Representative (if applicable)

Fingerprint

____________________________________ DATE (DAY/MO/YR): _________________

Signature of Subject or Legal Representative

(may place an X OR fingerprint if unable to sign)

For peer review only

SAYINI YA OFUNSA MAFUNSO

Ndamufotokozera otenga nawo mbali/oyimirira otenga nawo mbali za kafukufukuyu ndipo ndayankha mafunso onse amene anali nawo. Ndikukhulupirira kuti amvetsetsa uthenga onse omwe uli chikalata cha uthengawu ndipo avomera kutenga nawo mbali mwa kufuna kwao.

____

Dzina la ofunsa mafunso kapena oyimira akafukufuku

________________________________ ____________________________

Sayini ya ofusa mafunso kapena oyimira akafukufuku Date (must be the same as subject's)

For peer review only

14.4.APPENDIX D: Written Informed Consent – Male

WRITTEN INFORMED CONSENT
Identifying efficient linkage strategies for HIV self-testing (IDEaL)
Male

You are asked to participate in a research study entitled *"Identifying efficient linkages strategies for HIVST"* conducted by Partners in Hope and the University of California Los Angeles in the United States. You are being requested to take part in the study because you recently received a self-test kit and reported recently testing HIV-positive. Your participation in this study is entirely voluntary. You will be read the information below, and you are free to ask questions about anything you do not understand, before deciding whether or not to participate. ***I as the field assistant for this study will take you through this consenting process.***

- **Why is this study being done?**

HIV self-testing is very helpful for people who want to know their status but do not usually go to the health facility. However, it can be hard for individuals who test HIV-positive with HIV self-testing to be able to access other health services. Researchers want to determine what are the best interventions that can help men who are diagnosed with HIV use other health services, if desired. The study will offer several different strategies for HIV services to see what works best for men who use HIV self-testing kits.

- **What will happen if you take part in this research study?**

There are several steps to this study. If you volunteer to participate in this study you will have the opportunity to participate in the following components:

1. Complete two study visits where a research assistant like myself will interview you and ask you information about yourself, including whether you are married, number of sexual partners, your level of education, information about your experiences with HIV services, and how you feel about HIV testing and treatment services. We will ask you questions today (or a day that is convenient for you) and in four months in order to see if anything has changed. Each interview will last about 45minutes. You can refuse a follow-up survey at any point.
2. After completing the first interview you will be randomized to one of three interventions. We will do the randomization together so you can see exactly what you will be offered. Based on what intervention you are randomly selected for, you will also be offered a variety of health services from health facility staff. The potential interventions are:
   a) Reminders about the benefits of health services;
   b) Motivational Interviewing where you can talk to someone about your life, challenges you face, and strategies to make your life and better, and additional services as needed
   c) Home-based health services whereby a provider will offer you HIV services and NCD screening at your home as a one-time event. After four weeks, you will be visited by a health care worker who will escort you to the clinic for continued health services if desired.

Regardless of what arm you are randomized to, you can always refuse health services or refuse talking to a health care provider and still remain in the study. Even if you do not plan to use any additional

health services, don't worry, you can still be in the study and we can still complete the interviews we discussed above.

3. In the first 6 months of the study, we will also review medical records at your local health facility to see if you visited the health facility since enrolling in the study. This does not require interaction with you and will be completely confidential.

4. Finally, we may contact you within 6-months of the study to complete another in-depth interview so we can learn more about your experience with the study, recommendations for future interventions, and what additional services, if any, you would like. Not all participants will be contacted for the interview and you always have the right to decline an in-depth interview – refusal will not affect your participation in the larger study.

- **How long will you be in the research study?**

All study activities will be completed within 6-months of today.

- **Are there any potential risks or discomforts that you can expect from this study?**

You will be asked a series of questions by a research assistant about your sexual history and your experience receiving and using a self-test kit from your sexual partner. You may feel uncomfortable answering some of the questions asked by the interviewer. You can say "I don't want to answer" to any questions that make you uncomfortable. All questions will be asked in a private place so that no other patients or staff will hear your answers.

If you agree to participate in the assigned intervention, providers may ask to reach you at home or in the community. As with any health service, if you choose to initiate ART you may be at risk of unwanted status disclosure.

If you experience distress or adverse events, we will provide you with counseling resources or refer you to resources for assistance.

- **Are there any potential benefits to participating?**

You will have the opportunity to discuss information about your well-being and HIV services with a Research Assistant and possibly a health care provider and in a confidential, private manner. You will also have the chance to link to HIV care services at the facility of your choosing.

- **Are there any potential benefits to society?**

Information obtained as part of this work may be of benefit to the larger Malawi program, or similar programs in sub-Saharan Africa, since the work aims to determine if there are better ways to offer HIV services to men who use HIV self-test kits. If researchers better understand what type of programs work better for men, the program in Malawi can be scaled up and strengthened to provide these specific types of increased support.

- **Will you receive payment for being part of this study?**

Your participation is entirely voluntary. You will be provided MK 7,500 (equivalent to 10USD) for each survey completed (MK 15,000 / 20USD across the duration of the study). You will receive the above compensation regardless if you use HIV services or not. Those who complete the additional in-depth interview 6-months after study enrollment will receive an additional MK 7,500 (equivalent to 10USD) for their time.

- **What is the cost of participating in this study?**

There is no cost to participate in this study.

For peer review only

- **Will information about me be kept confidential?**

Authorized representatives of the Malawi National Health Sciences Research Council who are responsible for ensuring the rules related to research are followed, may need to review records of study participants. As a result, they may see your name; but they will not to reveal your identity to others.

When the results of the research are published or discussed in meetings, no information will be included that would reveal your identity. Any paperwork related to the study which contains information about you will be kept in a locked cabinet in a locked office. Only staff members of the study will have access to this information. A code will be assigned to each individual participating in the study. This code will be stored on a computer in a locked file. The key to unlock the information will only be known by the research staff. All data entered into a computer will be entered using this code so information will no longer have any information that can identify you such as your name. Forms containing any identifying information will be destroyed two years after the study is finished.

- **Participation and Withdrawal**

Your participation in this research is VOLUNTARY. If you choose not to participate, that will not affect your relationship with the hospital, your health provider or health centre you usually get your medical care from, or your right to health care. If you decide to participate, you are free to withdraw your consent and stop your participation at any time and can still receive future health care at the hospital or health center you go to.

- **Withdrawal of Participation by the Investigator**

The research investigator may stop your participation in this research if he or she feels this is best for you. The investigators will make the decision and let you know if it is not possible for you to continue. The decision may be made to protect your health and safety.

- **Who can answer questions I might have about this study?**

In the event of a research related injury or if you experience a problem, please immediately return to the hospital or health centre you go to or contact Mike Nyirenda. The NHSRC Ministry of Health information (Dr. Kathyola) is also provided in case you have questions about your rights as a research participant.

Investigator:

Khumbo Phiri
Mobile: +265999840946
Partners in Hope Clinic
Area 36, Plot8
M1 Road South
Lilongwe, Malawi

OR

Dr.C. Mitambo
The Secretariate, NHSRC
Ministry of Health
P.O Box 30377
Lilongwe 3
Cell +265888344 443

For peer review only

SIGNATURE OF RESEARCH SUBJECT [OR LEGAL REPRESENTATIVE]

I have read (or someone has read to me) the information provided above. I have been given an opportunity to ask questions and all of my questions have been answered to my satisfaction. I have been given a copy of this form.

BY SIGNING THIS FORM, I WILLINGLY AGREE TO PARTICIPATE IN THE RESEARCH:

____

Name of Subject

____

Name of Legal Representative (if applicable)

____________________________________ DATE (DAY/MO/YR): _________________

Signature of Subject or Legal Representative
(may place an X OR fingerprint if unable to sign)

SIGNATURE OF INVESTIGATOR OR DESIGNEE

I have explained the research to the subject or his/her legal representative and answered all of his/her questions. I believe that he/she understands the information described in this document and freely consents to participate.

____

Name of Investigator or Designee

______________________________ ___________________________

Signature of Investigator or Designee Date (must be the same as subject's)

For peer review only

WRITTEN INFORMED CONSENT
Identifying efficient linkage strategies for HIV self-testing (IDEaL)
Male (Chichewa)

Mukufunsidwa kuti mutenge nawo mbali mu gawo lino la kafukufuku wotchedwa"kupeza njira zoyenera zobwezeletsa anthu pa thandizo la ma ARV" amene akupangidwa ndi bungwe la Partners In Hope-EQUIP polojekiti ndi sukulu ya ukachenjede ya University of California Los Angeles yaku Amerika. Mukufunsidwa kuti mutenge nawo mbali mu gawo lakafukufukuyu chifukwa muli ndi kachirombo ka HIV komanso mwalandila kachipangizo koziyezela wekha HIVi. Kutenga nawo mbali mukafukufuku ameneyi sikokakamiza. Tikuwerengerani uthenga omwe walembedwa pansipa, ndipo muli ololedwa kufunsa mafunso aliwonse pa zomwe simukumvetsetsa,musanapange chiganizo chotenga nawo mbali kapena ayi. Ine ngati othandizira mukafufuku ameneyu ndikuthandizani pa ndondomeko yotenga chilolezo.*

Chifukwa chiyani kafukufukuyu akuchitika?

Kuziyeza wekha HIV ndi kofunika kwa anthu omwe akufuna kudziwa za momwe mthupi mwawo mulili ku mbali ya HIV koma sapita kuchipatala, kotelo, ndikovuta kwa anthu omwe apezeka ndi HIV kudzela chipangizo choziyezela wekha kuti apeze thandizo la zaumoyo. Akafukufuku akufuna apeze njira zabwino zomwe zingathe kuthandiza azibambo omwe apezeka ndi HIV kuti agwiritse ntchito thandizo lina lazaumoyo ngati angakonde kutero. Kafukufukuyu apeleka mwayi wa njira zosiyana siyana zothandizira HIV kuti aone zomwe zingagwire ntchito bwino kwa azibambo omwe amagwiritsa ntchito ka chida koziyezela wekha HIV.

Chichitike ndi chani mukatenga nawo mbali mu kafukufukuyu?

Pali ma gawo angapo omwe adzachitike mukafukuku, Ngati mungazipereke kutenga nawo mbali mu kafukufukuyu mudzatenga nawo mbali mu magawo otsatirawa:

Akafukufuku adzakuyendera kawiri. Ulendo woyamba tidzapemphani kuti mutenge nawo mbali mu kafukufuku yemwe tidzakufunseni zokhuza inuyo, kuphatikizapo ngati muli pa banja, muli ndi abwenzi ogonana nawo angati, maphunziro anu komanso zomwe munakumana nazo polandira thandizo la HIV komanso momwe mumnvera kumbali ya thandizo la HIV angakhalenso kuyezedwa HIV. Tikufunsani mafunso lelo (Ngati lelo muli okonzeka kuyankha mafuns)komanso miyezi inayi ikudzayi kuti tiwone ngati pali chomwe chasintha, kucheza kuli konse kuzitenga nthawi yosachepela makumi anayi ndi isanu. Muli ololedwa kukana kutenga nawo mbali mu kucheza kotsatira nthawi iliyonse.

2. Pamapepo pa kucheza koyamba mudzayikidwa mu imodzi mwa njira zitatu popanda ndondomeko iliyonse,pa nthawi yomwe mudzakhale mukuyikidwa mu njirayi mudzakhala muli pomwepo kuti muone njira yomwe mwapatsidwa.Potengera njira yomwe mwayikidwa mudzapatsidwanso mwayi wa mathandizo a zaumoyo angapo ochoka kwa opeleka thandizo la zaumoyo pa chiptala, zina mwa njira ndi:

a) Chikumbutso cha ubwino wa thandizo la zaumoyo

b) Kucheza kwa chilimbitso komwe mungathe ndi mwayi ocheza ndi anthu ena ndi kuwafotokozela za umoyo wanu, zofuta zomwe mumakumana nazo komanso njira zomwe mumagwiritsa ntchito kuti moyo wanu ukhale wosavuta.

c) Thandizo la zaumoyo lomwe mumatha kulandira pakhomo monga thandizo la HIV komanso NCD lomwe mumalandila kamodzi.Pakatha masaba anayi mudzayendeledwa ndi wa zaumoyo yemwe adzakupelekezeni ku chipatala komwe mukapitilize kulandira thandizo ngati mwakonda kutelo.

Posatengera njira yomwe mwapatsidwa mongathe kukana kulandira thandizo la zaumoyo koma ndikupitiliza kutenga nawo mbali mu kafukufuku ndipo tingathe kupitilizabe kucheza komwe tatchula m'mwambamu.

3. Miyezi isanu ndi umodzi yoyambilira ya kafukufuku tidzaona zambiri ya umoyo wanu ku chipatala cha m'dela lanu ngati munapitako mutalowa kale mu kafukufuku, zimenezi sizidzafuika kulankhula nanu ndipo zidzachitika mwachinsinsi.

4.Pamapeto tidzakuyendelani pakutha kwa miyezi isanu ndi umodzi ya kafukufuku kuti tidzacheze nanu komanso kuti tidzanve za momwe mukunvera za kafukufuku, maganizo anu okhudza njira zina za mtsogolo komanso, thandizo lina lowonjezera ngati ilipo. Si onse otenga nawo mbali omwe adzaonedwe ndipo muli ololedwa kukana kutenga nawo mbali mukucheza ndipo kukana kwanu sikudzaononga mwayi wanu otenga nawo mbali mu study.

Mutenga nthawi yaitali bwanji muli mukafukufuku?

Zochitika zonse zakafukufuku zizamalizidwa pa miyezi isanu ndi umodzi (6) kuchokera lero.

Pali zinthu zosowetsa mtendere kapena zodetsa nkhawa zomwe mungayembekezere kuchokera mu kafukufukuyu?

Muzafunsidwa mndandanda wa mafunso ndi othandizira mukafukufuku ameneyi zokhuza mbiri yanu pankhani zogonana ndi zochitika mutalandira ka chida koziyeza wekha kuchokera kwa bwezi wanu ogonana naye komanso mmene munagwiritsira ntchito. Mutha kukhala osamasuka poyankha mafunso ena omwe wofunsa mafunso angafunse. Mutha kunena kuti "sindikufuna kuyankha" kufunso lilironse lomwe simukumasuka nalo. Mafunso onse azafunsidwa pa malo achinsinsi pomwe odwala anzanu kapena ogwira ntchito pachipatala sadzamva nawo mayankho anu.

Ngati mudzavomere kutenga nawo mbali mu imodzi mwa njira, a zaumoyo adzakufunsani kuti akupezeni kunyumba kwanu kapena mu dela lanu. Monga mwa thandizo lililonse la zaumoyo, ngati mungavomere kuyamba kumwa mankhwala a ma ARV mungathe kukhala pa chiospyezo choulula za momwe mulili m'mthupi mwano mosafuna.

Ngati mungakumane ndi masautso aliwonse, tidzakupatsani uphungu kapena kukutumizani koti mukathandizidwe ndi uphungu.

Pali cholowa chilichonse potenga nawo mbali mukafukufukuyu?

Mudzakhala ndi mwayi okambilana ndikunva zambiri zokhudza moyo wanu komanso thandizo la HIV ndi othandiza kafukufuku komanso dotolo munjira yachinsisi. Mudzakhalanso ndi mwayi oyamba mankhwala a ma ARV ku chipatala cha kufuna kwanu.

Pali cholowa chilichonse kwa anthu a mudera?

For peer review only - http://bmjopen.bmj.com/site/about/guidelines.xhtml

Uthenga womwe udzatengedwe ngati mbali imodzi yakafukufukuyu uzakhala othandiza mu mapologalamu a dziko la Malawi, kapena ma pologalamu ena ofananirapo a kum'mwera kwa Africa, chifukwa choti ntchito imeneyi ikufuna kuona ngati pali njira yabwino yopeleka thandizo la HIV mwa azibambo, ma pologalamu aku Malawi azapita patsogolo ndikulimbikitsa njira zopititsira patsogolo.

Kodi mulandira malipiro potenga nawo mbali mukafukufukuyu?

Kutenga nawo mbali kosakakamiza. Mudzalandira chiongola dzanja cha ndalama zokwana 7,500(Pafupifupi MK 15,000/20USD). Mudzalandila ndalamazi olo mutakhala kuti simunatenge nawo kapena mwatenga nawo mbali mu thandizo la HIV. Kwa omwe adzamalize nawo kucheza kowonjezera patatha miyezi isanu ndi umodzi adzalandira ndalama yowonjezera yokwana MK 7,500 (yokwana pafupi fupi 10 USD0 chifukwa cha nthawi yawo.

Kodi pali kulipira kulikonse chifukwa chotenga nawo mbali mukafukufukuyu?

Kutenga nawo mbali mukafukufukuyu ndi kwaulere.

Kodi uthenga wanga uzasungidwa mwa chinsinsi?

Anthu ogwira nawo ntchito mu kafukufuku okhawo ndi amene adziwe za uthenga wanu kapena chilichonse chomwe mutiuze pa kafukufukuyu.

Anthu ovomerezeka oyimirira bungwe la National Health Sciences Research Council ndi UCLA office for Protection Of Research Subjects ndi amene ali ndi udindo woonetsetsa kuti malamulo a kafukufuku akutsatidwa, akhoza kufuna kuona nawo kaundula wa anthu amene akutenga nawo mbali mukafukufukuyu. Kutanthauza kuti akhonza kuzaona dzina lanu; koma sangaulure zokhuza inu kwa anthu ena.

Pa nthawi imene zotsatira za kafukufukuyu zidzatsindikidwa kapena kukambidwa mu misonkhano, palibe uthenga womwe udzayikidwe wokuzindikiritsani. Uthenga uliwonse wolembedwa pa pepala wokhunza inu uzasungidwa mu kabati yokhoma mu ofesi yokhomanso. Anthu ogwira nawo ntchito mukafukufukuyu okhawo ndi amene azathe kuona uthenga umenewu. Njira yotanthauzira uthenga umenewu izadziwika ndi anthu akafukufukuyu basi. Uthenga onse olowetsedwa pa makina a kompyuta uzalowetsedwa kugwiritsa ntchito nambala, ndekuti uthenga onse sudzakhalanso ndi zokuzindikiritsani monga dzina lanu. Ma pepala amene pali uthenga okuzindikitsani adzawonongedwa pakapita zaka ziwiri chimalizireni kafukufukuyu.

Kutenga nawo mbali mu kafukufukuyu SIKOKAKAMIZA. Ngati musankhe kuti simutenga nawo mbali, izi sizingasokoneze ubale wanu ndi chipatala chino, anthu ogwira ntchito kuchipatala,kapena chipatala chomwe mumalandilirako chithandizo, kapena ufulu wanu wolandira thandizo la chipatala. Ngati mwapanga chisankho chotenga nawo mbali, mukhoza kuchosa chilolezo chanu ndikusiya kutenga nawo mbali nthawi ina iliyonse ndipo mungathe kuzalandirabe chithandizo pa chipatala pano mtsogolo.

Kusiyitsidwa kutenga nawo mbali mu kafukufuku ndi wakafukufuku

Anthu opangitsa kafukufukuyu akhonza kukuletsani kutenga nawo mbali mukafukufukuyu akaona kuti ndi bwino kuti mutero. Anthu akafukufukuwa azapanga chiganizochi ndikukudziwitsani kuti sizitheka kuti mupitirize. Chiganizochi chitha kupangidwa kuti ateteze thanzi ndi chitetezo chanu.

For peer review only

Kusiyitsidwa kutenga nawo mbali mu kafukufuku ndi wakafukufuku

Anthu opangitsa kafukufukuyu akhonza kukuletsani kutenga nawo mbali mukafukufukuyu akaona kuti ndi bwino kuti mutero. Anthu akafukufukuwa azapanga chiganizochi ndikukudziwitsani kuti sizitheka kuti mupitirize. Chiganizochi chitha kupangidwa kuti ateteze thanzi ndi chitetezo chanu.

Investigator:

Khumbo Phiri
Mobile: +265999840946
Partners in Hope Clinic
Area 36, Plot8
M1 Road South
Lilongwe, Malawi

OR

Dr.C. Mitambo
The Secretariate, NHSRC
Ministry of Health
P.O Box 30377
Lilongwe 3
Cell +265888344 443

Wotenga mbali kapena omuyimira asayine

Ndawerenga (kapena munthu wina wandiwerengera) zonse zalembedwa mwambamu. Ndapatsidwa mwayi wofunsa mafunso ndi mafunso onse ndinafunsa ayankhidwa ndipo ndakhutitsidwa. Ndapatsidwa pepala lina langati lomweyi.

____

Dzina la otenga mbali

____

Dzina la oyimola malamulo

_______________________________________ Tsiku: __________________

Wotenga mbali kapena oyimira malamulo asayine

(Mutha kuyika X ngati simungathe kusayinila)

**Ofufuza asayinire/Kapena othandizila Kafukufuku**

Ndafotokoza za kafukufuku uyu kwa otenga nawo mbali kapena owayimila, ndayankhanso mafunso awo onse. Ndikukhulupilira kuti amvetsetsa zomwe zananedwa mu chikalata ichi

ndiponso avomereza mwakufuna kwawo kutenga nawo mbali.

____

Dzina la ofufuza kapena othandizila kafukufuku

__________________________________ ______________________________

Ofufuza kapena othandizila kafukufuku asayine apa Tsiku

For peer review only

14.5. APPENDIX E: Baseline Survey – Male

BASELINE SURVEY
Identifying efficient linkage strategies for HIV self-testing (IDEaL)
Male

**Question Name**	**Label**	**Responses**
INTRODUCTION SECTION		
interviewer	Full Name of Interviewer	
Interview date	Interview date	
Time start	Time survey started	
District	District	
TA	TA	
village	Village	
SECTION A: DEMOGRAPHICS		
Intro Note	Thank you for agreeing to participate. Now I will ask you a few questions about yourself and who you are. Please feel free to answer honestly. There are no right or wrong answers.	
a7	What is your tribe?	1. Lomwe<br>2. Sena<br>3. Chewa<br>4. Mang'anja/Nyanja<br>5. Ngoni<br>6. Tumbuka<br>7. Tonga<br>8. Yao

For peer review only

		99. Other, specify
a3	What is the highest level of school you attended?	1. Primary<br>2. Secondary<br>3. Higher
a3b	What class did you complete in your highest level of school?	____________________
a4	Please think of the past 12months, how would you describe your primary occupation?	1. Working formally (employed full time)<br>2. Working informally (ganyu, farming, business)<br>3. Not working
a5	Are you currently married?	1. Married<br>2. Live-in partner<br>3. Steady Girlfriend/Boyfriend<br>4. Separated<br>5. Divorced<br>99. Other, specify
a6	How many living children do you have?	____________________
a6b	What is the age of your youngest child?	____________________
a6bc	What age is the child (in years or months)	____________________
a4b	How many children currently live with you?	____________________
a7	How many sexual partners have you had in the past 12 months?	____________________
a8	Have you had sex with someone besides your wife/husband without a condom in the past 12 months?	1. Yes<br>0. No<br>88. Don't know/ Not sure<br>89. Refused to answer

For peer review only

a8b	Have you had sex without a condom in the past 12 months?	1. Yes<br>0. No<br>88. Don't know/ Not sure<br>89. Refused to answer
SECTION B: INCOME QUESTIONS		
Intro Note	I will now discuss with you about the valuable items that you or your household possesses. As I will be chatting with you I will also some questions about money you have and activities that you indulge in to find money.	
b1	Please think of the past 12 months, how would you describe your primary occupation?	1. Working formally (employed full time)<br>2. Working informally (ganyu, farming, business)<br>3. Not working
b1b	Think about all the work you have done in the past month. How many days did you normally work this month that gave you pay?	________________
b2	Do you have any savings for the future, such as a bank account, savings group or cash?	1. Yes<br>0. No
Household Assets		
b3	Does your household have:<br>*The respondent said that his/her household doesn't have any of the household assets. Please probe and ensure that this is correct before you proceed.*	
b3_1	Metal Roof?	1. Yes<br>0. No
b3_2	Electricity?	1. Yes

For peer review only

		0. No
b3_3	Paraffin lamp with no glass?	1. Yes<br>0. No
b3_4	A paraffin lamp?	1. Yes<br>0. No
b3_5	A radio?	1. Yes<br>0. No
b3_6	A television?	1. Yes<br>0. No
b3_7	A cellular phone?	1. Yes<br>0. No
b3_8	A bed?	1. Yes<br>0. No
b3_9	A sofa set?	1. Yes<br>0. No
b3_10	A table?	1. Yes<br>0. No
b3_11	A refrigerator	1. Yes<br>0. No
b3_12	Mattress?	1. Yes<br>0. No
b3_13	Chair(s)?	1. Yes<br>0. No
b3_14	Cattle?	1. Yes<br>0. No
b3_15	Goat?	1. Yes<br>0. No
b3_16	Sheep?	1. Yes<br>0. No
b3_17	Pigs?	1. Yes<br>0. No

For peer review only

b3_18	Donkey?	1. Yes<br>0. No
b3_19	Chickens?	1. Yes<br>0. No
b3_20	Other poultry?	1. Yes<br>0. No
b4	In the past 30 days, have you drank beer?	1. Yes<br>0. No
b4b	How many days in the past 30 days have you drank beer?	________________________
b4c	How much money did you spend on beer the last time you went?	MWK:
b4d	In total, approximately how much money did you spend on beer in the past 30 days?	MWK:
Relationship		
Intro Note	Now I'd like to talk to you about your current sexual relationship	
f8	How long have you been/were you in a sexual relationship with your partner?	Days________________<br>Months______________<br>Years________________
f9	Do you have children with your partner? How many children?	________________________
f10	How often do you currently talk to your partner?	1. Everyday<br>2. A couple times a week<br>3. Once a week<br>4. A couple times a month<br>5. Once a month<br>6. Less than once a month<br>7. Not at all (never)
f10b	In a typical month, who earns more money? You, or your partner?	1. Myself<br>2. This partner<br>3. We earn the same amount<br>88. Don't know

For peer review only

Decision Making		
Intro Note	Now I would like to talk to you about how you and your partner make decisions.	
f11	Who usually decides how the money you earn will be used?	1. Yourself (Respondent)<br>2. Jointly (This partner and you together)<br>3. Mainly this partner<br>4. Someone else<br>5. Do not earn money<br>88. Refuse to say
f11b	(if above question=4) Who decides?	______________________
f12	Who usually decides how your partner's earnings will be used?	1. Yourself (Respondent)<br>2. Jointly (This partner and you together)<br>3. Mainly this partner<br>4. Someone else<br>5. Do not earn money<br>88. Refuse to say
f12b	(if above question=4) Who decides?	______________________
f13	Who usually makes decisions about health care for yourself?	1. Yourself (Respondent)<br>2. Jointly (This partner and you together)<br>3. Mainly this partner<br>4. Someone else<br>5. Not applicable/ Don't have children<br>88. Refuse to say
f13b	(if above question=4) Who decides?	______________________
f14	Who usually makes decisions about health care for your child with this partner?	1. Yourself (Respondent)<br>2. Jointly (This partner and you together)<br>3. Mainly this partner<br>4. Someone else

For peer review only

		5. Not applicable/ Don't have children<br>88. Refuse to say
f14b	(if above question=4) Who decides?	___________________
f15	Who usually makes decisions about health care for your partner?	1. Yourself (Respondent)<br>2. Jointly (This partner and you together)<br>3. Mainly this partner<br>4. Someone else<br>5. Not applicable/ Don't have children<br>88. Refuse to say
f15b	(if above question=4) Who decides?	___________________
f16	Who usually makes decisions about making major household purchases?	1. Yourself (Respondent)<br>2. Jointly (This partner and you together)<br>3. Mainly this partner<br>4. Someone else<br>5. Not applicable/ Don't have children<br>88. Refuse to say
f16b	(if above question=4) Who decides?	
Note	I would like to ask you questions about [probability/chance/likelihood] that certain things will happen. There are ten beans in this cup. I will ask you to pick some of the beans and put them in the plate. The number of beans that you are going to put in the plate will reflect the probability that something will happen. One bean means there is very little chance that something will happen. If you do not put any bean in the plate it means you are certain that there is no likelihood that something will happen.	

For peer review only

note2	If you put additional beans in the plate it means the chance that something will happen will also increase. For example, if you put one or two beans in the plate, it means there is little chance that something will happen. Even though there is little chance but it can happen. If you put ten beans it means there is equal chance of something happening or not. If you put six beans it means the chance that something will happen is slightly greater than not happening. If you put all ten beans, it means you are certain that whatever the case something will really happen. There is no wrong or right answer I just want to know what you think.	
note3	INTERVIEWER: Report for each question the NUMBER OF BEANS put in the PLATE. After each question, replace the beans on the table (unless otherwise noted).	
	Practice	
pr1	Pick the number of beans that reflects how likely you think it is that:	____________
pr1b	You will go to the market at least once within the next 2 days.	____________
pr1c	You will go to the market at least once within the next 2 weeks.	____________
	Practice	
pr2	INTERVIEWER: Did Respondent add any beans between pr1b and pr1c?	1. Yes<br>0. No

For peer review only - http://bmjopen.bmj.com/site/about/guidelines.xhtml

pr3	Remember, as time goes by, you may find more time to go to the market. Therefore, you should have added beans to the plate. Let me ask you again. Now, add beans in the plate so that the number of beans in the plate reflects how likely you think it is that you will go to the market at least once within 2 weeks.<br><br>How likely you think it is that you will go to the market at least once within 2 weeks?	____________________
f17	Pick the number of beans that reflects how likely you think:	
f17b	You will still be married/with [partner one year from now.	____________________
f17c	You are currently infected with HIV/AIDS	____________________
f17d	You will become infected with HIV/AIDS during the next 12 months	____________________
f17e	You will become infected with HIV/AIDS during their lifetime	____________________
f17c	partner is infected with HIV/AIDS now.	____________________
f17d	partner will become infected with HIV/AIDS during the next 12 months.	____________________
SELF REPORTED HEALTH AND HAPPINESS		
Intro Note	Now I'd like to talk to you about how healthy and happy you feel.	
e1	I am interested in your general level of well-being or satisfaction with life. How satisfied are you with your life, all things considered?	1. Very satisfied<br>2. Somewhat satisfied<br>3. Neutral<br>4. Somewhat unsatisfied<br>5. Very unsatisfied

For peer review only

e2	Do you think that you are more, equally or less satisfied than other persons your age and sex living in your village?	1. More satisfied<br>2. Equally satisfied<br>3. Less satisfied
e3	In general, would you say your health now is: very good, good, poor or very poor?	1. Very good<br>2. Good<br>3. Poor<br>4. Very poor
e4	How would you compare your health to other people of the same age and sex in your village?	1. More healthy<br>2. Equally healthy<br>3. Less healthy
e5	In the past month, how many days were you too sick to work/go to school/complete household chores?	___________________
Happiness		
e6	How true are the following statements for you in the last month?	
e6_1	I have felt depressed	1. Strongly Agree<br>2. Agree<br>3. Disagree<br>4. Strongly Disagree
e6_2	I have felt life was not worth living	1. Strongly Agree<br>2. Agree<br>3. Disagree<br>4. Strongly Disagree
e6_3	I have felt content.	1. Strongly Agree<br>2. Agree<br>3. Disagree<br>4. Strongly Disagree
e6_4	I have felt lonely	1. Strongly Agree<br>2. Agree<br>3. Disagree<br>4. Strongly Disagree
GENDER EQUITABLE MEN SCALE		
Note	Please tell me if you strongly agree, agree, disagree, or strongly disagree with the following statements:	

For peer review only

j1	Woman's most important role is to take care of her home and cook (take care of home is about housekeeping)	1. Strongly Agree<br>2. Agree<br>3. Unsure<br>4. Disagree<br>5. Strongly Disagree<br>88. Refuse to say
j2	Men need sex more than women	1. Strongly Agree<br>2. Agree<br>3. Unsure<br>4. Disagree<br>5. Strongly Disagree<br>88. Refuse to say
j3	Men don't talk about sex, they just do it.	1. Strongly Agree<br>2. Agree<br>3. Unsure<br>4. Disagree<br>5. Strongly Disagree<br>88. Refuse to say
j4	There are times when a woman deserves to be beaten	1. Strongly Agree<br>2. Agree<br>3. Unsure<br>4. Disagree<br>5. Strongly Disagree<br>88. Refuse to say
j5	Changing diapers, giving kids a bath & feeding kids are mother's responsibility	1. Strongly Agree<br>2. Agree<br>3. Unsure<br>4. Disagree<br>5. Strongly Disagree<br>88. Refuse to say
j6	It is a woman's responsibility to avoid getting pregnant	1. Strongly Agree<br>2. Agree<br>3. Unsure<br>4. Disagree<br>5. Strongly Disagree<br>88. Refuse to say
j7	A man should have the final word about decisions in his home	1. Strongly Agree<br>2. Agree<br>3. Unsure<br>4. Disagree

For peer review only

		5. Strongly Disagree<br><br>88. Refuse to say
j8	Men are always ready to have sex	1. Strongly Agree<br>2. Agree<br>3. Unsure<br>4. Disagree<br>5. Strongly Disagree<br><br>88. Refuse to say
j9	A woman should tolerate violence in order to keep her family together	1. Strongly Agree<br>2. Agree<br>3. Unsure<br>4. Disagree<br>5. Strongly Disagree<br><br>88. Refuse to say
j10a	I would be outraged if my wife asked me to use a condom.	1. Strongly Agree<br>2. Agree<br>3. Unsure<br>4. Disagree<br>5. Strongly Disagree<br><br>88. Refuse to say
j10b	Men would be outraged if their wife asked them to use a condom	1. Strongly Agree<br>2. Agree<br>3. Unsure<br>4. Disagree<br>5. Strongly Disagree<br><br>88. Refuse to say
j11	A man and a woman should decide together what type of contraceptive to use	1. Strongly Agree<br>2. Agree<br>3. Unsure<br>4. Disagree<br>5. Strongly Disagree<br><br>88. Refuse to say
j12	I would never have a homosexual friend	1. Strongly Agree<br>2. Agree<br>3. Unsure<br>4. Disagree<br>5. Strongly Disagree<br><br>88. Refuse to say

For peer review only

j13a	If someone insults me, I will defend my reputation, with force if I have to.	1. Strongly Agree<br>2. Agree<br>3. Unsure<br>4. Disagree<br>5. Strongly Disagree<br><br>88. Refuse to say
j13b	If someone insults a man, he should defend his reputation, with force if he has to	1. Strongly Agree<br>2. Agree<br>3. Unsure<br>4. Disagree<br>5. Strongly Disagree<br><br>88. Refuse to say
j14	To be a man you need to be tough.	1. Strongly Agree<br>2. Agree<br>3. Unsure<br>4. Disagree<br>5. Strongly Disagree<br><br>88. Refuse to say
j15	Men should be embarrassed if unable to get an erection	1. Strongly Agree<br>2. Agree<br>3. Unsure<br>4. Disagree<br>5. Strongly Disagree<br><br>88. Refuse to say
j16	If a guy gets a woman pregnant, child is the responsibility of both the man and woman	1. Strongly Agree<br>2. Agree<br>3. Unsure<br>4. Disagree<br>5. Strongly Disagree<br><br>88. Refuse to say
j17	A man should know what his partner likes during sex	1. Strongly Agree<br>2. Agree<br>3. Unsure<br>4. Disagree<br>5. Strongly Disagree<br><br>88. Refuse to say
j18	The participation of the father is important in raising children	1. Strongly Agree<br>2. Agree<br>3. Unsure<br>4. Disagree

For peer review only

		5. Strongly Disagree<br>88. Refuse to say
j19	It's important for men to have friends to talk about their problems	1. Strongly Agree<br>2. Agree<br>3. Unsure<br>4. Disagree<br>5. Strongly Disagree<br>88. Refuse to say
j20	A couple should decide together if they want to have children.	1. Strongly Agree<br>2. Agree<br>3. Unsure<br>4. Disagree<br>5. Strongly Disagree<br>88. Refuse to say
STIGMA		
Note	In this next section I'd like to discuss your thoughts about people living with HIV in your community. Please feel free to talk openly, there is no right or wrong answer. I am interested in your own thoughts.	
i3	I would buy fresh vegetables from a shopkeeper or vendor if I knew that this person had HIV	1. Strongly Agree<br>2. Agree<br>3. Neutral<br>4. Disagree<br>5. Strongly Disagree
i4	If a member of my family became sick with AIDS, I would be willing to care for her or him in our own household	1. Strongly Agree<br>2. Agree<br>3. Neutral<br>4. Disagree<br>5. Strongly Disagree
i5	In my opinion, if a female teacher has HIV but is not sick, she should be allowed to continue teaching in the school	1. Strongly Agree<br>2. Agree<br>3. Neutral<br>4. Disagree<br>5. Strongly Disagree
EXPECTATIONS		

For peer review only

Intro Note	I would like to ask you questions about [probability/chance/likelihood] that certain things will happen. There are ten beans in this cup. I will ask you to pick some of the beans and put them in the plate. The number of beans that you are going to put in the place will reflect the probability that something will happen. One beans means there is very little chance that something will happen. If you do not put any bean in the plate it means you are certain that there is no likelihood that something will happen.	
h4	Pick the number of beans that reflects how likely you think it is that:	___________
h4b	You will have to rely on family members for financial assistance in the next 12 months.	___________
h4c	You will have to provide some family members with financial assistance in the next 12 months.	___________
Note	Next, I would like to ask you a few questions about what you expect in the future. I know that nobody knows for sure what the future may bring, but let`s just talk about your best guess.	
h5	In the next year how likely is it that you will:	
h5a	You will be enrolled in school one year from now	___________
h5b	Start a new business?	___________
h5c	Open a bank account?	___________
h5d	Buy land?	___________

For peer review only

h5e	Save money?	______________
h5f	Experience shortage of food?	______________
h5g	Have steady work?	______________
Tested for HIV		
pd2	Approximately how many times have you ever been tested for HIV?<br>*Enter "-99" if client doesn't remember*	______________
pd3	When was the last time you were tested for HIV?	a. Year______<br>b. Month________
pd5	Think about the very first time you received an HIV+ test result. Since that very first HIV+ test result, have you ever tested for HIV again (excluding a confirmatory test)?	1. Yes<br>0. No
pd6	Have you ever initiated ART?	1. Yes<br>0. No
First Initiated ART		
pd6b	When did you first initiate ART?	a. Year_______<br>b. Month____
pd7	Have you ever been >14 days late for an ART appointment?	1. Yes<br>0. No
pd7b	How many times?	______________
pd8	Do you know anyone who is on ART?	1. Yes<br>0. No
pd8b	Now think about the person on ART who you are closest with.<br><br>How often do you talk with them	1. Everyday<br>2. A couple times a week<br>3. Once a week<br>4. A couple times a month

For peer review only

	about ART?	5. Once a month<br>6. Less than once a month<br>7. Not at all (never)
pd9	Have you disclosed your HIV status to anyone besides your partner?	1. Yes<br>0. No
pd9b	Who else did you disclose to?<br>*Mark all that apply*	1. Sister<br>2. Brother<br>3. Father<br>4. Mother<br>5. Uncle<br>6. Aunt<br>7. Friend<br>8. Mother-in-Law<br>9. Father-in-Law<br>10. My children<br>11. Employee<br>12. Other sexual partner<br>99. Other, specify_____
pd10	Of those people you disclosed to, who do you talk to most often?	1. Sister<br>2. Brother<br>3. Father<br>4. Mother<br>5. Uncle<br>6. Aunt<br>7. Friend<br>8. Mother-in-Law<br>9. Father-in-Law<br>10. My children<br>11. Employee<br>12. Other sexual Partner<br>99. Other, specify_____
pd10c	How often do you talk to that person?	1. Everyday<br>2. A couple times a week<br>3. Once a week<br>4. A couple times a month<br>5. Once a month<br>6. Less than once a month<br>7. Not at all (never)

For peer review only

PREVIOUS USE OF HEALTH SERVICES		
Intro Note	Now I'd like to talk to you about your experience with using health services at health facilities.	
h1	Have you gone to a health facility in the past 12 months (either for yourself or someone else - AKA as a guardian)?	1. Yes<br>0. No
h2	How many times have you gone to the health facility in the past 12 months?	______________________
h3	Now think about yourself specifically. How many times have you gone to a health facility in the past 12 months for your own health care?	______________________
h_a1	When was the last time (the YEAR) you went to a health facility for YOUR OWN health?<br>NOTE: PUT WHAT YEAR. (i.e., 2015). If DO NOT REMEMBER, help them estimate. IF NEVER GONE, put -99	______________________
h4	What services did you receive at your last health facility visit for your own health?	1. ANC<br>2. Family Planning<br>3. Delivery<br>4. Post-natal<br>5. Under Five<br>6. HTC<br>7. ART<br>8. Feeling sick (OPD)<br>9. Injury (OPD)<br>10. Dentist<br>11. None<br>99. Other specify_____
h_a2	Now think about the SECOND most recent time you went to a health facility for YOUR OWN health. What year did you go to the health facility?	______________________
c3	Now please think about your MOST RECENT visit to a health facility, excluding today. When did you go?	a. Year___________<br>b. Month_________

For peer review only

c4	Which facility did you go to?	1. Current facility<br>0. Other facility, specify________________
c5	What was the main service you went for?	1. ANC<br>2. Family Planning<br>3. Delivery<br>4. Post-natal<br>5. Under Five<br>6. HTC<br>7. ART<br>8. Feeling sick (OPD)<br>9. Injury (OPD)<br>10. Dentist<br>11. None<br>99. Other specify_____
c5b	Who received services?	1. Myself<br>2. My child<br>3. My partner<br>4. Another family member<br>5. A friend<br>99. Other, specify_______
c6a	Did you (or the person you came with) receive another service?	1. Yes<br>0. No
c6	What was the second service you went for?	1. ANC<br>2. Family Planning<br>3. Delivery<br>4. Post-natal<br>5. Under Five<br>6. HTC<br>7. ART<br>8. Feeling sick (OPD)<br>9. Injury (OPD)<br>10. Dentist<br>11. None<br>99. Other specify_____
c6b	Who received services?	1. Myself<br>2. My child<br>3. My partner<br>4. Another family member<br>5. A friend<br>99. Other, specify_______

For peer review only

Service Satisfaction		
c10	Now I would like to talk to you about your satisfaction with the services you received that day. Please tell me whether any of these were problems for you at the VISIT YOU ARE THINKING ABOUT NOW, and if so, whether they were major or minor problems for you.	
c10_1	Time you waited to see a provider	1. Major<br>2. Minor<br>0. No-problem<br>88. Not applicable<br>89. Don't know
c10_2	Ability to discuss problems or concerns about your pregnancy	1. Major<br>2. Minor<br>0. No-problem<br>88. Not applicable<br>89. Don't know
c10_3	Amount of explanation you received about the problem or treatment	1. Major<br>2. Minor<br>0. No-problem<br>88. Not applicable<br>89. Don't know
c10_4	Privacy from having others see the examination	1. Major<br>2. Minor<br>0. No-problem<br>88. Not applicable<br>89. Don't know
c10_5	Privacy from having others hear your consultation discussion	1. Major<br>2. Minor<br>0. No-problem<br>88. Not applicable<br>89. Don't know
c10_6	Availability of medicines at this facility	1. Major<br>2. Minor

For peer review only

		0. No-problem<br>88. Not applicable<br>89. Don't know
c10_7	The hours of service at this facility, i.e., when they open and close	1. Major<br>2. Minor<br>0. No-problem<br>88. Not applicable<br>89. Don't know
c10_8	The number of days services are available to you	1. Major<br>2. Minor<br>0. No-problem<br>88. Not applicable<br>89. Don't know
c10_9	The cleanliness of the facility	1. Major<br>2. Minor<br>0. No-problem<br>88. Not applicable<br>89. Don't know
c10_10	How the staff treated you	1. Major<br>2. Minor<br>0. No-problem<br>88. Not applicable<br>89. Don't know
c10_11	Cost for services or treatments	1. Major<br>2. Minor<br>0. No-problem<br>88. Not applicable<br>89. Don't know
Satisfaction		
c11	In general, which of the following statements best describes your opinion of the services you either received or were provided at the facility	1. I am very satisfied with the services I received<br>2. I am satisfied with the services I received<br>3. I am not satisfied with the services I received

For peer review only

		4. I am very dissatisfied with the services I received
c12	Did you recommend this health facility to a friend or family member?	1. Yes<br>0. No
Comment	We have reached the end of the chat. Thank you for your time. Do you have anything else you would like to say?	
End Note	Thank the participant for their time and give them transport reimbursement, if they did not come for an ART appointment	
Comments	Enumerator comments	
End of the survey!		

For peer review only

BASELINE SURVEY
Identifying efficient linkage strategies for HIV self-testing (IDEaL)
Male (Chichewa)

Question Name	Label	Responses
INTRODUCTION SECTION		
interviewer	Full Name of Interviewer	
Interview date	Interview date	
Time start	Time survey started	
District	District	
Facility	Facility	
ID	ID	
SECTION A: DEMOGRAPHICS		
Intro Note	Zikomo povomera kutenga nawo mbali. Pa nthawi ino ndikufunsani ma funso ochepa okhudza za inu komanso kuti ndinu ndani. Chonde khalani omasuka kuyankha moona. Palibe yankho lokhoza komanso lolakwa.	
a7	Ndinu mtundu wanji wa munthu?	9. Lomwe<br>10. Sena<br>11. Chewa<br>12. Mang'anja/Nyanja<br>13. Ngoni<br>14. Tumbuka

For peer review only - http://bmjopen.bmj.com/site/about/guidelines.xhtml

		15. Tonga<br>16. Yao<br>99. Other, specify
a3	Kodi maphunziro anu mudapita nawo patali bwanji?	1. Pulayimale<br>2. Sekondale<br>3. Koleji
a3b	Ndi kalasi liti munamaliza ya maphunziro anu apamwamba?	____________________
a4	Chonde ganizani za masabata khumi ndi awiri apitawa, mungafotokoze bwanji za ntchito yomwe mumagwira?	1. Ntchito yokhazikika (ya nthawi yayitali)<br>2. Ntchito yosakhazikika (ganyu, ulimi, bizinesi)<br>3. Sindikugwira ntchito
a5	Kodi pakadali pano muli pa banja?	1. Pa banja<br>2. Kukhala limodzi ngati banja<br>3. Chibwezi Chokhazikika<br>4. Tinasiyana<br>5. Banja linatha<br>99. Zina
a6	Muli ndi ana angati amoyo?	____________________
a6b	Mwana wanu wang'ono ali ndi zaka zingati?	____________________
a6bc	Mwana wanu wang'ono ali ndi zaka zingati?	____________________
a4b	Pakadali pano ana omwe mumakhala nawo ndi angati?	____________________
a7	Pa miyezi khumi ndi iwiri yapitayi mwakhala ndi abwenzi ogonana nawo angati?	____________________
a8	Mwakhalapo ndi bwenzi logonana kupatula akazi anu?amuna anu osagwiritsa ntchito kondomu mu miyezi khumi ndi awiri yapitayi?	1. Eya<br>2. Ayi<br>88. Sindikudziwa<br>89. Akana kuyankha

For peer review only

a8b	Mwagonanapo ndi munthu osagwiritsa ntchito kondomu mu miyezi khumi ndi awiri yapitayi?	1. Eya<br>2. Ayi<br>88. Sindikudziwa<br>89. Akana kuyankha
SECTION B: INCOME QUESTIONS		
Intro Note	Pa nthawi ino ndikufunsani za zipangizo zomwe inu komanso apabanja panu alinazo. Mkati mwakucheza kwathu ndikufunsaninso za ndalama zomwe mulinazo komanso komanso zomwe mumachita kuti mupeze ndalama.	
b1	Chonde ganizirani za miyezi Khumi ndi iwiri yapitayi, mungafotokoze bwanji za ntchito yomwe mumagwira?	4. Ntchito yokhazikika<br>5. Ganyu/bisinesi<br>6. Sindikugwira ntchito
b1b	Ganizani za ntchito zonse mwagwira mwezi watha. Mwagwira masiku angati olipidwa?	________________
b2	Muli ndi ndalama zilizonse zomwe mukusungira za mtsogolo monga, ku banki, gulu losugira ndalama kapena ndalama zosunga kunyumba?	2. Eya<br>3. Ayi
Household Assets		
b3	Does your household have:<br>*The respondent said that his/her household doesn't have any of the household assets. Please probe and ensure that this is correct before you proceed.*	
b3_1	Denga la malata?	1. Eya<br>2. Ayi
b3_2	Magetsi ?	1. Eya<br>2. Ayi
b3_3	Koloboyi?	1. Eya<br>2. Ayi
b3_4	Nyali?	1. Eya<br>2. Ayi

For peer review only

b3_5	Wailesi?	1. Eya<br>2. Ayi
b3_6	Kanema?	1. Eya<br>2. Ayi
b3_7	Lamya ya M'manja?	1. Eya<br>2. Ayi
b3_8	Kama?	1. Eya<br>2. Ayi
b3_9	Sofa?	1. Eya<br>2. Ayi
b3_10	Tebulo?	1. Eya<br>2. Ayi
b3_11	FIliji?	1. Eya<br>2. Ayi
b3_12	Matilesi?	1. Eya<br>2. Ayi
b3_13	Mipando	1. Eya<br>2. Ayi
b3_14	Ng'ombe?	1. Eya<br>2. Ayi
b3_15	Mbuzi?	1. Eya<br>2. Ayi
b3_16	Nkhosa?	1. Eya<br>2. Ayi
b3_17	Nkhumba?	1. Eya<br>2. Ayi
b3_18	Bulu?	1. Eya<br>2. Ayi
b3_19	Nkhuku?	1. Eya<br>2. Ayi
b3_20	Zoweta zina	1. Eya<br>2. Ayi
b4	Mu masiku makumi atatu apitawa mwamako mowa?	1. Eya<br>2. Ayi
b4b	Pa masiku makumi atatu apitawa mwamwa mowa masiku angati?	____________________
b4c	Mwataya ndalama zingati masiku omaliza omwe munapita ku mowa?	MWK:
b4d	Zonse pamodzi, mwataya ndalama zingati pa mowa mu masiku makumi atatu apitawa?	MWK:
Relationship		

For peer review only

Intro Note	Panthawi ino ndikufunsani za abwezi ogonana nawo?	
f8	Mwakhala pa ubwenzi ogonana ndi bwenzi lanu kwa nthawi yayitali bwanji?	Masiku ___________<br>Miyezi _____________<br>Zaka________________
f9	Muli ndi ana ndi bwenzi lanu logonana nalo? Ana angati?	________________________
f10	Mumayankhulana mowirikiza bwanji ndi bwenzi lanu pakadali pano?	1. Tsiku ndi tsiku<br>2. Masiku angapo pasabata<br>3. Kamodzi pa sabata<br>4. Kangapo pa mwezi<br>5. Kamodzi pa mwezi<br>6. Kosakwana mwezi<br>7. Sitiyankhulani
f10b	Pa mwezi amalandila ndalama zambiri ndi ndani? Inu kapena bwenzi lanu?	1. Ine<br>2. Bwenzi langa<br>3. Timalandira ndalama zofanana<br>4. Sindikudziwa
Decision Making		
Intro Note	Pa nthawi ino ndikufunsani za momwe mumapangira maganizo ndi bwenzi lanu	
f11	Nthawi zambiri ndi ndani amene amapanga chiganizo cha momwe ndalama mumapeze zigwiritsidwe ntchito?	1. Ine<br>2. Timagwirizana<br>3. Nthawi zambiri bwenzi langa<br>4. Munthu wina<br>5. Sindipeza ndalama<br>88. Sindikufuna kuyankha
f11b	(Ngati yankho ndi 4) Amapanga chiganizo ndi ndani?	________________________
f12	Amapanga chiganizo cha momwe ndalama za bwenzi lanu zigwiritsidwe ntchito ndi ndani?	1. Ine<br>2. Timagwirizana<br>3. Nthawi zambiri bwenzi langa<br>4. Munthu wina<br>5. Sindipeza ndalama

For peer review only - http://bmjopen.bmj.com/site/about/guidelines.xhtml

		88. Sindikufuna kuyankha
f12b	(Ngati yankho ndi 4)	________________________
f13	Nthawi zambiri amapanga chiganizo chokhudza thandizo la zaumoyo wanu ndi ndani?	1. Ine<br>2. Timagwirizana<br>3. Nthawi zambiri bwenzi langa<br>4. Munthu wina<br>5. Sindipeza ndalama<br>88. Sindikufuna kuyankha
f13b	(Ngati yankho ndi 4) Amapanga chiganizo ndi ndani?	________________________
f14	Nthawi zambiri amapanga chiganizo cha thandizo la zaumoyo la mwana yemwe muli naye ndi bwenzi lanu ndi ndani?	6. Ine<br>7. Mogwirizana<br>8. Nthawi zambiri bwenzi langa<br>9. Munthu wina<br>10. Ndilibe mwana<br>88. Sindikufuna kuyankha
f14b	(if above question=4) Who decides?	________________________
f15	Nthawi zambiri amapanga chiganizo chokhudza thandizo la zaumoyo la bwenzi lanu ndi ndani?	1. Ine<br>2. Mogwirizana<br>3. Nthawi zambiri bwenzi langa<br>4. Munthu wina<br>5. Ndilibe ana<br>88.Sindikufuna kuyankha
f15b	(Ngati yankho ndi 4) Amapanga chiganizo ndi ndani?	________________________
f16	Kodi amapanga ziganizo zogula katundu mkulumkulu wapakhomo panu ndi ndani?	1. Ine<br>2. Mogwirizana<br>3. Nthawi zambiri bwenzi langa

For peer review only

		4. Munthu wina<br>5. Ndilibe ana<br>88.Sindikufuna kuyankha
f16b	(Ngati yankho ndi 4) Amapanga chiganizo ndani?	
Note	Ndikufuna ndikufunseni zokhudzana za (Kuthekela/mwayi) kuti zinthu zina zichitike. Muli nyemba mu kapu. Ndikufunsani kuti musankhe zina mwa nyemba ndipo muyike mu mbale. Mulingo wa nyemba omwe muyike mu mbale udzafanizira kuti chinachake chichitika. Nyemba imodzi ikusonyeza kuti mwayi ndiochepa kuti chinachake chichitika. Ngati simuika nyemba mm'bale ndekuti mukutsimikiza kuti palibe mwayi oti chinachake chichitika	
note2	Ngati muyike nyemba zowonjezera mu mbale, zikutanthauza kuti mwayi oti chinachake chichitika uchuluka, mwachitsanzo ngati muyika nyemba imodzi kapena ziwiri mwayi oti chinachake chichitika. Ngakhale pali mwayi ochepa koma chinachake chichitika. Ngati muyike nyemba nkhumi zikutanthauza kuti pali mwayi ofanana oti chinachake chichika kapena ayi. Ngati muyike nyemba zisanu ndi imodzi zikutanthauza kuti mwayi woti chinachake chichitika uli ochulukilapo kuposa mwayi oti chinachake sichichitika. Ngati muyike nyemba zonse khumi ndekuti muli ndi chikhulupiliro chonse kuti chinachake chichitikadi pavute pasavute. Palibe yankho lokhonza kapena lolakwa ndikungofuna kudziwa zomwe mukuganiza.	

For peer review only

note3	Ofunsa: Pelekani yankho lanu pa funso lililonse nambala ya nyemba zomwe lili m'bale. Pakutha pa funso lililonse, bwezeletsani nyemba pa tebulo.	
Practice		
pr1	Sankhani mulingo wa nyemba omwe ukhale ndi kuthekela kumene mukuganiza kuti:	________________
pr1b	Mupita ku msika mosachepera kamodzi m'masiku awiri akudzawa	________________
pr1c	Mupita ku msika mosachepera kamodzi m'masabata awiri akudzawa.	________________
Practice		
pr2	Ofunsa: kodi oyankha anawonjezera nyamba pakati pa pr1b ndi pr1c	2. Eya<br>0. Ayi
pr3	Kumbukurani kuti pamene nthawi ikupita muzipeza mpata wambiri opita kunsika. Choncho, munayenela kuti mwaika nyemba zambiri m'bale<br><br>Kodi mukuganiza kuti kumsika mupita mosachepela kamodzi bwanji mu nyengo ya ma sabata awiriwa?	________________
f17	Sankhani mlingo wa nyemba umene ufanizile kaganidwe kanu:	
f17b	Mukhalabe mukanali pa banja/ndi (bwenzi oposela chaka chimodzi kuchoka pano	________________
f17c	Mudzadwala mu miyezi khumi ndi iwiri ikubwelayi	________________
f17d	Mudzayamba kumwa ma ARV mu miyezi itatu ikubwelayi	________________

For peer review only

f17e	Mudzadziwitsa achibale ndi anzanu za zotsatira zanu za HIV m'miyezi itatu ikubwelayi	________________
f17c	Mukusafuna kwanu, anzanu ndi abale azadziwa kuti muli ndi HIV, mu miyezi itatu yapitayi.	________________
SELF REPORTED HEALTH AND HAPPINESS		
Intro Note	Pa nthawi ino ndikufuna ndikambe nanu zokhudza umoyo ndi chisangalalo chanu.	
e1	Ndili ndi chidwi ndi mukudziwa za umoyo ndi kukhutitsidwa kwanu. Kodi muli okhutitsidwa bwanji ndi moyo wanu, pakutengela zonse.	6. Okhutitsidwa kwamburi<br>7. Okhutitsidwa pang'ono<br>8. Pakatikati<br>9. Okhutitsidwa pang'ono<br>10. Osakhutitsidwa olo pang'ono
e2	Kodi mukuona ngati muli okhutitsidwa mofanana kapena osakhutitsidwa pang'ono mosaposela anthu ena a muna kapena akazi a msinkhu wanu ndi opezeka m'mudzi mwanu?	1. Okhutitsidwa kwambiri<br>2. Okhutiotsidwa<br>3. Okhutitsidwa pang'ono
e3	Kutengela zonse, Kodi munganene kuti umoyo wanu tsopano uli bwino kwambiri, ulibwino, sulibwino, suli bwino olo pang'ono	1. Bwino kwambiri<br>2. Bwino<br>3. Silibwino<br>4. SIlibwino olo pang'ono
e4	Kodi mungazifananizile bwanji za thanzi lanu ndi la anthu ena a msikhu ofanana ndi wanu, amuna kapena akazi a m'mudzi mwanu.	1. A thanzi kwambiri<br>2. A thanzi<br>3. Nthanzi lochepekela
e5	Kodi mwezi wathawu, ndi masiku angati omwe munadwara kwambiri ofika kukulepheretsani kugwira ncthito/kupita ku sukulu/kugwira ntchito za pa khomo	________________
Happiness		
e6	Kodi ziganizo izi ndi zowona bwanji kwa inu?	
e6_1	Ndinali ndi nkhawa	1. Ndikugwirizana nazo kwambiri

For peer review only

		2. Ndikugwirizana nazo<br>3. Sindikugwirizana nazo<br>4. Sindikugwirizana nazo kwambiri
e6_2	Ndimanva ngati moyo wafika	1. Ndikugwirizana nazo kwambiri<br>2. Ndikugwirizana nazo<br>3. Sindikugwirizana nazo<br>4. Sindikugwirizana nazo kwambiri
e6_3	Ndimanva kukhutitsidwa	1. Ndikugwirizana nazo kwambiri<br>2. Ndikugwirizana nazo<br>3. Sindikugwirizana nazo<br>4. Sindikugwirizana nazo kwambiri
e6_4	Ndimanva kusalidwa	1. Ndikugwirizana nazo kwambiri<br>2. Ndikugwirizana nazo<br>3. Sindikugwirizana nazo<br>4. Sindikugwirizana nazo kwambiri
GENDER EQUITABLE MEN SCALE		
Note	Chonde ndiuzeni ngati mukugwirizana nazo kwabiri, mukugwirizana nazo, simukugwirizana nazo, simukugwirizana nazo olo pang'ono ziganizo izi:	
j1	Udindo ofunikila wa mzimayi ndi kusamala khomo lake ndi kuphika.	1. Ndikugwirizana nazo kwambiri<br>2. Ndikugwirizana nazo<br>3. Sindikukhulupilira<br>4. Sindikugwirizana nazo<br>5. Sindikugwirizana nazo olo pang'ono<br>88. Sindikufuna kuyankha
j2	Abambo amafuna kugonana kuposa amayi	1. Ndikugwirizana nazo kwambiri<br>2. Ndikugwirizana nazo<br>3. Sindikukhulupilira<br>4. Sindikugwirizana nazo

For peer review only

		5. Sindikugwirizana nazo olo pang'ono<br>88. Sindikufuna kuyankha
j3	Azibambo sakonda kukambirana za kugonana amangochita	1. Ndikugwirizana nazo kwambiri<br>2. Ndikugwirizana nazo<br>3. Sindikukhulupilira<br>4. Sindikugwirizana nazo<br>5. Sindikugwirizana nazo olo pang'ono<br>88. Sindikufuna kuyankha
j4	Nthawi zina mzimayi amayenela kumenyedwa.	1. Ndikugwirizana nazo kwambiri<br>2. Ndikugwirizana nazo<br>3. Sindikukhulupilira<br>4. Sindikugwirizana nazo<br>5. Sindikugwirizana nazo olo pang'ono<br>88. Sindikufuna kuyankha
j5	Kusinta matewela, kusambitsa mwana, kudyetsa mwana ndi udindo wa mzimayi	1. Ndikugwirizana nazo kwambiri<br>2. Ndikugwirizana nazo<br>3. Sindikukhulupilira<br>4. Sindikugwirizana nazo<br>5. Sindikugwirizana nazo olo pang'ono<br>88. Sindikufuna kuyankha
j6	Ndi udindi wa mzimayi kupewa kutenga mimba.	1. Ndikugwirizana nazo kwambiri<br>2. Ndikugwirizana nazo<br>3. Sindikukhulupilira<br>4. Sindikugwirizana nazo<br>5. Sindikugwirizana nazo olo pang'ono<br>88. Sindikufuna kuyankha
j7	Mzibambo ayenela kukhala ndi chiganizo chomaliza cha mnyumba mwake.	1. Ndikugwirizana nazo kwambiri<br>2. Ndikugwirizana nazo<br>3. Sindikukhulupilira<br>4. Sindikugwirizana nazo

For peer review only

		5. Sindikugwirizana nazo olo pang'ono<br><br>88. Sindikufuna kuyankha
j8	Azibambo amakhala okonzeka ku gonana	1. Ndikugwirizana nazo kwambiri<br>2. Ndikugwirizana nazo<br>3. Sindikukhulupilira<br>4. Sindikugwirizana nazo<br>5. Sindikugwirizana nazo olo pang'ono<br><br>Sindikufuna kuyankha
j9	Mzimayi akuyenela kulekelela nkhaza kuti asunge banja lake.	1. Ndikugwirizana nazo kwambiri<br>2. Ndikugwirizana nazo<br>3. Sindikukhulupilira<br>4. Sindikugwirizana nazo<br>5. Sindikugwirizana nazo olo pang'ono<br><br>Sindikufuna kuyankha
j10a	Ndikhoza kukwiya akaza anga atati andiuze kuti ndigwiritse ntchito kondumu.	1. Ndikugwirizana nazo kwambiri<br>2. Ndikugwirizana nazo<br>3. Sindikukhulupilira<br>4. Sindikugwirizana nazo<br>5. Sindikugwirizana nazo olo pang'ono<br><br>88. Sindikufuna kuyankha
j10b	Azibambo akhoza kukwiya akazi awo atawauza kuti agwiritse ntchito kondomu	1. Ndikugwirizana nazo kwambiri<br>2. Ndikugwirizana nazo<br>3. Sindikukhulupilira<br>4. Sindikugwirizana nazo<br>5. Sindikugwirizana nazo olo pang'ono<br><br>88. Sindikufuna kuyankha
j11	Mzimayi komanso nzibambo agwirizane limodzi kuti agwiritse ntchito njira yanji yakulela	1. Ndikugwirizana nazo kwambiri<br>2. Ndikugwirizana nazo<br>3. Sindikukhulupilira<br>4. Sindikugwirizana nazo

For peer review only

		5. Sindikugwirizana nazo olo pang'ono<br>88. Sindikufuna kuyankha
j12	Sindingakhale ndi nzanga wopanga zamathanyula	1. Ndikugwirizana nazo kwambiri<br>2. Ndikugwirizana nazo<br>3. Sindikukhulupilira<br>4. Sindikugwirizana nazo<br>5. Sindikugwirizana nazo olo pang'ono<br>88. Sindikufuna kuyankha
j13a	Ngati munthu angandinyoze, ndiziteteza pogwiritsa ntchito mphanvu, ngati ndikufunika kutero.	1. Ndikugwirizana nazo kwambiri<br>2. Ndikugwirizana nazo<br>3. Sindikukhulupilira<br>4. Sindikugwirizana nazo<br>5. Sindikugwirizana nazo olo pang'ono<br>88. Sindikufuna kuyankha
j13b	Ngati munthu anganyoze mzibambo, aziteteze pogwiritsa ntchito mphanvu ngati akufunika kutelo.	1. Ndikugwirizana nazo kwambiri<br>2. Ndikugwirizana nazo<br>3. Sindikukhulupilira<br>4. Sindikugwirizana nazo<br>5. Sindikugwirizana nazo olo pang'ono<br>Sindikufuna kuyankha
j14	Kuti ukhale mzibambo ukufunika kukhala ovuta.	1. Ndikugwirizana nazo kwambiri<br>2. Ndikugwirizana nazo<br>3. Sindikukhulupilira<br>4. Sindikugwirizana nazo<br>5. Sindikugwirizana nazo olo pang'ono<br>Sindikufuna kuyankha<br>Sindikugwirizana nazo kwambiri<br>88. Refuse to say<br>Sindikufuna kuyankha
j15	Azibambo akuyenela kunva manyazi ngati akukanika kutota	1. Ndikugwirizana nazo kwambiri<br>2. Ndikugwirizana nazo

For peer review only

		3. Sindikukhulupilira<br>4. Sindikugwirizana nazo<br>5. Sindikugwirizana nazo olo pang'ono<br>88. Sindikufuna kuyankha
j16	Ngati mzibambo wapeleka mimba kwa mzimayi, mwanayo ndi udindo wa anthu onse a wiri.	1. Ndikugwirizana nazo kwambiri<br>2. Ndikugwirizana nazo<br>3. Sindikukhulupilira<br>4. Sindikugwirizana nazo<br>5. Sindikugwirizana nazo olo pang'ono<br>88. Sindikufuna kuyankha
j17	Nzibambo akuyenela kudziwa zomwe bwenzi lake limakonda pogonana	1. Ndikugwirizana nazo kwambiri<br>2. Ndikugwirizana nazo<br>3. Sindikukhulupilira<br>4. Sindikugwirizana nazo<br>5. Sindikugwirizana nazo olo pang'ono<br>88. Sindikufuna kuyankha
j18	Kutenga nawo mbali kwa a bambo ndi kofunika polela mwana	1. Ndikugwirizana nazo kwambiri<br>2. Ndikugwirizana nazo<br>3. Sindikukhulupilira<br>4. Sindikugwirizana nazo<br>5. Sindikugwirizana nazo olo pang'ono<br>88. Sindikufuna kuyankha
j19	Ndi zofunika kuti abambo azikhala ndi anzawo okambilana nawo mavuto awo.	1. Ndikugwirizana nazo kwambiri<br>2. Ndikugwirizana nazo<br>3. Sindikukhulupilira<br>4. Sindikugwirizana nazo<br>5. Sindikugwirizana nazo olo pang'ono<br>88. Sindikufuna kuyankha
j20	Banja lizigwirizana limodzi ngati likufuna kukhala ndi mwana	1. Ndikugwirizana nazo kwambiri<br>2. Ndikugwirizana nazo<br>3. Sindikukhulupilira<br>4. Sindikugwirizana nazo

For peer review only

		5. Sindikugwirizana nazo olo pang'ono<br>88. Sindikufuna kuyankha
STIGMA		
Note	Mugawo lotsatila ndifuna tikambilane zokhudza maganizo anu a anthu omwe ali ndi HIV komanso akukhala mu dela lanu. Chonde khalani omasuka kuyankhula momasuka, palibe yankho lohoza kapena lolakwa. Ndikufuna ndinve maganizo anu.	
i3	Ndikhoza kugula ndiwo zamasamba kwa munthu oti ndikudziwa kuti ali ndi HIV.	1. Ndikugwirizana zano kwambiri<br>2. Ndikugwirizana nazo<br>3. Pakatikati<br>4. Sindikugwurizana nazo<br>5. Sindikugwirizana nazo kwambiri
i4	Ngati wachibale wanga angadwale AIDS, ndingavomele kumusamala pakhomo panga.	1. Ndikugwirizana zano kwambiri<br>2. Ndikugwirizana nazo<br>3. Pakatikati<br>4. Sindikugwurizana nazo<br>5. Sindikugwirizana nazo kwambiri
i5	M'maganizo mwanga, ngati mphunzitsi wa mkazi ali ndi HIV koma sakudwala, aloledwe kupitiliza kuphunzitsa.	1. Ndikugwirizana zano kwambiri<br>2. Ndikugwirizana nazo<br>3. Pakatikati<br>4. Sindikugwurizana nazo<br>5. Sindikugwirizana nazo kwambiri
EXPECTATIONS		

For peer review only

Intro Note	Ndikufuna ndikufunseni zokhudzana za (Kuthekela/mwayi) kuti zinthu zina zichitike. Muli nyemba mu kapu. Ndikufunsani kuti musankhe zina mwa nyemba ndipo muyike mu mbale. Mulingo wa nyemba omwe muyike mu mbale udzafanizira kuti chinachake chichitika. Nyemba imodzi ikusonyeza kuti mwayi ndiochepa kuti chinachake chichitika. Ngati simuika nyemba mm'bale ndekuti mukutsimikiza kuti palibe mwayi oti chinachake chichitika	
h4	Sankhani mulingo wa nyemba omwe ukhale ndi kuthekela kumene mukuganiza kuti:	
h4b	Mudzakhala mukudalila apabanja panu pa nkhani ya zachuma mu miyezi itatu ikubwelayi.	
h4c	Mukhala mukuthandiza achibale ena pa nkhani za chuma miyezi itatu ikubweyi.	
Note	Kotsatira ndikufuna ndikufunseni mafunse ochepa okhudza chiyembekezo chanu cha mtsogolo. Ndikudziwa palibe yemmwe amadziwa za mtsogolo koma tiyeni tikambe mongoyelezeka.	
h5	Mu chaka chamawa chiyembekezero choti mudzakhala mu:	
h5a	Mudzakhala mutayamba sukulukuchoka lelo chaka chamawa	
h5b	Kuyamba buzinesi	
h5c	Kutsegula akaunti ku banki	
h5d	Ku gula malo?	

For peer review only

h5e	Kusunga ndalama?	____________________
h5f	Kukhala ndi chakudya chochepa?	____________________
h5g	Kukhala pa ntchito yokhazikika	____________________
Tested for HIV		
pd2	Mwayezetsapo HIV kokwana kangati?<br>*Enter "-99" if client doesn't remember*	____________________
pd3	Komaliza munayezetsa HIV kanali liti?	a) Chaka ______<br>b) Mwezi ________
pd5	Ganizani za ulendo wanu ayamba olandira zotsatira zakuyezadwa kwa HIV. Koyamba kulandira zotsatira zoti akupezani ndi HIV kanali liti?	a) Chaka ______<br>b) Mwezi ________
pd6	Have you ever initiated ART?<br>Munayamba mwamapo ma ARV?	2. Eya<br>0. Ayi
First Initiated ART		
pd6b	Koyamba kumwa ma ARV kanali liti?	c. Chaka ______<br>d. Mwezi ________
pd7	Munayamba mwachedwako kukatenga mwankhwala masiku ochepela khumi ndi folo?	1. Eya<br>0. Ayi
pd7b	Masiku angati?	____________________
Pd7c	Munasiya kumwa ma ARV chifukwa chani?	____________________<br>____________________
pd8	Mukudziwa munthu wina aliyense	

For peer review only

	yenwe akumwa ma ARV?	1. Eya<br>0. Ayi
pd8b	Pano ganizani za munthu yemwe akumwa ma ARV amene mulinali pafupi<br><br>Mumayankhula naye mowilikiza bwanji?	1. Siku ndi tsiku<br>2. Kangapo pa sabata<br>3. Kamodzi pa sabata<br>4. Kangapo pa sabata<br>5. Kamodzi kamwezi<br>6. Kochepera kamodzi pa mwezi<br>7. Sitiyankhulana
pd9	Munayamba mwaululapo za momwe nthupi mwanu mulili za HIV kwa anthu ena kupatura bwenzi lanu?	1. Eya<br>1. Ayi
pd9b	Wina munamuuza anali ndani?<br>*Mark all that apply*	1. Ntcemwali<br>2. Ntchimwene<br>3. Bambo anga<br>4. Mayi anga<br>5. Malume<br>6. Azakhali anga<br>7. Nzanga<br>8. Apongozi akazi<br>9. Apongozi amuna<br>10. Ana anga<br>11. Ogwira naye ntchito<br>12. Bwezi logonanalo<br>13. Zina, fotokozani
pd10	Pa munthu yemwe munamuwuza, ndi ndani yemwe mumayankhula naye kawirikawiri?	1. Ntcemwali<br>2. Ntchimwene<br>3. Bambo anga<br>4. Mayi anga<br>5. Malume<br>6. Azakhali anga<br>7. Nzanga<br>8. Apongozi akazi<br>9. Apongozi amuna<br>10. Ana anga<br>11. Ogwira naye ntchito<br>12. Bwezi logonanalo<br><br>Zina, fotokozani

pd10c	Muntjuyi mumayankhula naye mowirikiza bwanji?	1. Tsiku ndi tsiku<br>2. Kangapo pa sabata<br>3. Kamodzi pa sabata<br>4. Kangapo pa mwezi<br>5. Kamodzi pa mwezi<br>6. Kochepera kamodzi pa mwezi<br>7. Sitiyankhulana
PREVIOUS USE OF HEALTH SERVICES		
Intro Note	Pa ntahwi ino ndifuna tikambilane zokhudza za zomwe mwadutsamo pogwiritsa ntchito thandizo la za umoyo pa chipatala.	
h1	Munayamba mwapitako ku chipatala miyezi khumi ndi iwiri yapitayi.(chifukwa cha inu kapena kupelekeza munthu wina)?	1. Eya<br>0. Ayi
h2	Mwapita ku chipatala kangati miyezi khumi ndi iwiri yapitayi?	____________________
h3	Pa nthawi ino ganizani za inu. Mwapita kangati ku chipatala miyezi khumi ndi awiri yapitayi panokha kukalandira thandizo la zaumoyo?	____________________
h_a1	Komaliza kupita kuchipatala(chaka) chifukwa mwadwala ndinu kanali liti?<br>NOTE: PUT WHAT YEAR. (i.e., 2015). If DO NOT REMEMBER, help them estimate. IF NEVER GONE, put -99	____________________
h4	Munalandira thandizo lanji ulendo umaliza munapita kuchipatala?	12. ANC<br>13. Family Planning<br>14. Delivery<br>15. Post-natal<br>16. Under Five Ku ana<br>17. Kukayezetsa HIV<br>18. ARV<br>19. (OPD) Kudwala<br>20. (OPD)Kuvulala<br>21. Kukonana ndi dotolo wa manu

For peer review only

		22. Palibe
h_a2	Tsopano ganizani za kachiwiri komwe munapita kuchipatala nokha cha posachedwapa. Munapita ku chiptala chaka chanji?	________________________
c3	Chonde ganizani za ulendo wanu munapita kuchipatala chaposachedwa, kuphatikizapo lelo. Munapita lelo?	c. Chaka___________<br>d. Mwezi_________<br>Mwezi
c4	Munapita ku chipatala chiti	1. Chipatala chomwe mumapita pakali pano<br>2. Chipatala china, tchulani
c5	Munapita kukalandila thandizo lanji ku chipatala?	1. ANC<br>2. Family Planning<br>3. Delivery<br>4. Post-natal<br>5. Under Five Ku ana<br>6. Kukayezetsa HIV<br>7. ARV<br>8. (OPD) Kudwala<br>9. (OPD)Kuvulala<br>10. Kukonana ndi dotolo wa manu<br>11. Palibe
c5b	Amene analandira thandizo la zaumoyo anali ndani?	1. Ineyo<br>2. Mwana wanga<br>3. Bwenzi langa<br>4. Wapabanja panga<br>5. Mzanga<br>99. Zina, fotokozani_______
c6a	Inu kapena munthu yemwe munapita naye kuchipatala analandila munthu wina wathandizo la zaumoyo?	2. Eya<br>0. Ayi
c6	Thandizo lachiwiri la zaumyo lomwe manalandira linali lanji?	1. ANC<br>2. Family Planning<br>3. Delivery<br>4. Post-natal<br>5. Under Five Ku ana<br>6. Kukayezetsa HIV<br>7. ARV<br>8. (OPD) Kudwala

For peer review only

		9. (OPD)Kuvulala<br>10. Kukonana ndi dotolo wa manu<br>11. Palibe
c6b	Analandira thandizo la zaumoyo anali ndani?	1. Ine<br>2. Mwana wanga<br>3. Bwezi langa<br>4. Wapabanja panga<br>5. Nzanga Zina, fotokozani
Service Satisfaction		
c10	Pa nthawi ino ndikufuna ndikufunseni za kukhutila kwanu ndi thandizo la zaumoyo munalandila pa tsikulo. Chinde nduuzeni ngai zina mwazotsatirazi zinli vito kwa inu patsiku lomwe munapita kuchipatala, ngati eya, ngati vutolo linali lalikulu kapena ayi.	
c10_1	Nthawi yomwe mumafuna kuonana ndi dotolo	1. Vuto kwambiri<br>2. Vuto pang'ono<br>3. Silinali vuto<br>88. Not applicable<br>98. Sindikudziwa
c10_2	Kuthekela kokambilana za mavuto anu a pakati.	1. Vuto kwambiri<br>2. Vuto pang'ono<br>0. Silinali vuto<br>88. Not applicable<br>98. Sindikudziwa
c10_3	Mulingo waku Kufotokozeledwa munalandira okhudza vuto lanu kapena thandizo.	1. Vuto kwambiri<br>2. Vuto pang'ono<br>0. Silinali vuto<br>88. Not applicable<br>98. Sindikudziwa
c10_4	Chinsinsi kuopetsa ena kuwona zotsatila za umoyo	1. Vuto kwambiri<br>2. Vuto pang'ono<br>0. Silinali vuto<br>88. Not applicable<br>98. Sindikudziwa

For peer review only

c10_5	Chinsinsi kuopa ena kunva za zokambilana zanu	1. Vuto kwambiri<br>2. Vuto pang'ono<br>0. Silinali vuto<br>88. Not applicable<br>98. Sindikudziwa
c10_6	Kupezeka kwa mankwala mzipatala	1. Vuto kwambiri<br>2. Vuto pang'ono<br>0. Silinali vuto<br>88. Not applicable<br>98. Sindikudziwa
c10_7	Ma ola omwe thandizo limapelekedwa pa chipatala i.e thawi yotsegulila ndi yotsekela	1. Vuto kwambiri<br>2. Vuto pang'ono<br>0. Silinali vuto<br>88. Not applicable<br>98. Sindikudziwa
c10_8	Masiku omwe mumalandila thandizo	1. Vuto kwambiri<br>2. Vuto pang'ono<br>0. Silinali vuto<br>88. Not applicable<br>98. Sindikudziwa Sindikudziwa
c10_9	Ukhondo wa pa chipatala	1. Vuto kwambiri<br>2. Vuto pang'ono<br>0. Silinali vuto<br>88. Not applicable<br>98. Sindikudziwa
c10_10	Momwe ogwira ntchito amakusamalilani	1. Vuto kwambiri<br>2. Vuto pang'ono<br>0. Silinali vuto<br>88. Not applicable<br>98. Sindikudziwa
c10_11	Mtengo wa thandizo ndi mankwala	1. Vuto kwambiri<br>2. Vuto pang'ono<br>1. Silinali vuto<br>88. Not applicable<br>98. Sindikudziwa

For peer review only

Satisfaction		
c11	Ndi chiganizo chiti chomwe chikufotokoza za maganizo anu okhudza thandizo lomwe munalandila pa chipatala?	1. Ndili okhutitsidwa kwabiri ndi thandizo ndinalandila.<br>2. Ndili okhutitsidwa ndi thandizo ndinalandila.<br>3. Sindili okhutitsidwa ndi thandizo ndinalandila<br>4. Sindili okhutitsidwa kwambiri ndi thandizo ndinalandila
c12	Munatchulako za chipatalachi kwa nzanu kapena wachibale?	2. Eya<br>0. Ayi
Comment	Tafika pamapeto a kucheza kwathu. Zikomo chifukwa cha nthawi yanu. Pali chili chonse mukufuna kuwonjezera?	
End Note	Thank the participant for their time and give them transport reimbursement, if they did not come for an ART appointment	
Comments	Enumerator comments	
End of the survey!		

For peer review only

14.6. APPENDIX F: Baseline survey – Female

BASELINE SURVEY
Identifying efficient linkage strategies for HIV self-testing (IDEaL)
Female

INTRODUCTION SECTION		
interviewer	Full Name of Interviewer	
Interview date	Interview date	
Time start	Time survey started	
District	District	
Facility	Facility	
ID	ID	

For peer review only

SECTION A: DEMOGRAPHICS		
Intro Note	Thank you for agreeing to participate. Now I will ask you a few questions about yourself and who you are. Please feel free to answer honestly. There are no right or wrong answers.	
a7	What is your tribe?	1. Lomwe<br>2. Sena<br>3. Chewa<br>4. Mang'anja/Nyanja<br>5. Ngoni<br>6. Tumbuka<br>7. Tonga<br>8. Yao<br>99. Other, specify
a3	What is the highest level of school you attended?	1. Primary<br>2. Secondary<br>3. Higher
a3b	What class did you complete in your highest level of school?	________________
a4	Please think of the past 12months, how would you describe your primary occupation?	1. Working formally (employed full time)<br>2. Working informally (ganyu, farming, business)<br>3. Not working
a5	Are you currently married?	1. Married<br>2. Live-in partner<br>3. Steady Boyfriend<br>4. Separated<br>5. Divorced

For peer review only

		99. Other, specify
a6	How many living children do you have?	____________
a6b	What is the age of your youngest child?	____________
a6bc	What age is the child (in years or months)	____________
a4b	How many children currently live with you?	____________
a7	How many sexual partners have you had in the past 12 months?	____________
a8	Have you had sex with someone besides your husband without a condom in the past 12 months?	2. Yes<br>0. No<br>88. Don't know/ Not sure<br>89. Refused to answer
a8b	Have you had sex without a condom in the past 12 months?	2. Yes<br>0. No<br>88. Don't know/ Not sure<br>89. Refused to answer
SECTION B: INCOME QUESTIONS		
Intro Note	I will now discuss with you about the valuable items that you or your household possesses. As I will be chatting with you I will also some questions about money you have and activities that you indulge in to find money.	
b1	Please think of the past 12 months, how would you describe your primary occupation?	1. Working formally (employed full time)<br>2. Working informally (ganyu, farming, business)<br>3. Not working

For peer review only - http://bmjopen.bmj.com/site/about/guidelines.xhtml

b1b	Think about all the work you have done in the past month. How many days did you normally work this month that gave you pay?	________________
b2	Do you have any savings for the future, such as a bank account, savings group or cash?	2. Yes<br>0. No
	Household Assets	
b3	Does your household have:<br>*The respondent said that his/her household doesn't have any of the household assets. Please probe and ensure that this is correct before you proceed.*	
b3_1	Metal Roof?	2. Yes<br>0. No
b3_2	Electricity?	2. Yes<br>0. No
b3_3	Paraffin lamp with no glass?	2. Yes<br>0. No
b3_4	A paraffin lamp?	2. Yes<br>0. No
b3_5	A radio?	2. Yes<br>0. No
b3_6	A television?	2. Yes<br>0. No
b3_7	A cellular phone?	2. Yes<br>0. No
b3_8	A bed?	2. Yes<br>0. No
b3_9	A sofa set?	2. Yes<br>0. No
b3_10	A table?	2. Yes

For peer review only

		0. No
b3_11	A refrigerator	2. Yes<br>0. No
b3_12	Mattress?	2. Yes<br>0. No
b3_13	Chair(s)?	2. Yes<br>0. No
b3_14	Cattle?	2. Yes<br>0. No
b3_15	Goat?	2. Yes<br>0. No
b3_16	Sheep?	2. Yes<br>0. No
b3_17	Pigs?	2. Yes<br>0. No
b3_18	Donkey?	2. Yes<br>0. No
b3_19	Chickens?	2. Yes<br>0. No
b3_20	Other poultry?	2. Yes<br>0. No
b4	In the past 30 days, have you drank beer?	2. Yes<br>0. No
b4b	How many days in the past 30 days have you drank beer?	______________
b4c	How much money did you spend on beer the last time you went?	MWK:
b4d	In total, approximately how much money did you spend on beer in the past 30 days?	MWK:
Relationship		

For peer review only

Intro Note	Now I'd like to talk to you about your current sexual relationship	
f8	How long have you been/were you in a sexual relationship with your partner?	Days_______________<br>Months_____________<br>Years________________
f9	Do you have children with your partner? How many children?	______________________
f10	How often do you currently talk to your partner?	1. Everyday<br>2. A couple times a week<br>3. Once a week<br>4. A couple times a month<br>5. Once a month<br>6. Less than once a month<br>7. Not at all (never)
f10b	In a typical month, who earns more money? You, or your partner?	1. Myself<br>2. This partner<br>3. We earn the same amount<br>88. Don't know
	Decision Making	
Intro Note	Now I would like to talk to you about how you and your partner make decisions.	
f11	Who usually decides how the money you earn will be used?	1. Yourself (Respondent)<br>2. Jointly (This partner and you together)<br>3. Mainly this partner<br>4. Someone else<br>5. Do not earn money<br>88. Refuse to say
f11b	(if above question=4) Who decides?	______________________
f12	Who usually decides how your partner's earnings will be used?	1. Yourself (Respondent)<br>2. Jointly (This partner and you together)<br>3. Mainly this partner<br>4. Someone else<br>5. Do not earn money<br>88. Refuse to say

For peer review only

f12b	(if above question=4) Who decides?	___________________
f13	Who usually makes decisions about health care for yourself?	1. Yourself (Respondent)<br>2. Jointly (This partner and you together)<br>3. Mainly this partner<br>4. Someone else<br>5. Not applicable/ Don't have children<br>88. Refuse to say
f13b	(if above question=4) Who decides?	___________________
f14	Who usually makes decisions about health care for your child with this partner?	1. Yourself (Respondent)<br>2. Jointly (This partner and you together)<br>3. Mainly this partner<br>4. Someone else<br>5. Not applicable/ Don't have children<br>88. Refuse to say
f14b	(if above question=4) Who decides?	___________________
f15	Who usually makes decisions about health care for your partner?	1. Yourself (Respondent)<br>2. Jointly (This partner and you together)<br>3. Mainly this partner<br>4. Someone else<br>5. Not applicable/ Don't have children<br>88. Refuse to say
f15b	(if above question=4) Who decides?	___________________
f16	Who usually makes decisions about making major household purchases?	1. Yourself (Respondent)<br>2. Jointly (This partner and you together)<br>3. Mainly this partner<br>4. Someone else

For peer review only

		5. Not applicable/ Don't have children<br>88. Refuse to say
f16b	(if above question=4) Who decides?	
Note	I would like to ask you questions about [probability/chance/likelihood] that certain things will happen. There are ten beans in this cup. I will ask you to pick some of the beans and put them in the plate. The number of beans that you are going to put in the plate will reflect the probability that something will happen. One bean means there is very little chance that something will happen. If you do not put any bean in the plate it means you are certain that there is no likelihood that something will happen.	
note2	If you put additional beans in the plate it means the chance that something will happen will also increase. For example, if you put one or two beans in the plate, it means there is little chance that something will happen. Even though there is little chance but it can happen. If you put ten beans it means there is equal chance of something happening or not. If you put six beans it means the chance that something will happen is slightly greater than not happening. If you put all ten beans, it means you are certain that whatever the case something will really happen. There is no wrong or right answer I just want to know what you think.	
note3	INTERVIEWER: Report for each question the NUMBER OF BEANS put in the PLATE. After each question, replace the beans on the table (unless otherwise noted).	
	Practice	
pr1	Pick the number of beans that reflects how likely you think it is that:	____________________

For peer review only

pr1b	You will go to the market at least once within the next 2 days.	________________
pr1c	You will go to the market at least once within the next 2 weeks.	________________
Practice		
pr2	INTERVIEWER: Did Respondent add any beans between pr1b and pr1c?	2. Yes<br>0. No
pr3	Remember, as time goes by, you may find more time to go to the market. Therefore, you should have added beans to the plate. Let me ask you again. Now, add beans in the plate so that the number of beans in the plate reflects how likely you think it is that you will go to the market at least once within 2 weeks.<br><br>How likely you think it is that you will go to the market at least once within 2 weeks?	________________
f17	Pick the number of beans that reflects how likely you think:	
f17a	You will still be married/with [partner one year from now.	________________
f17b	Your partner will become sick during the next 12 months	________________
f17c	Your partner will start ART treatment in the next 3 months	________________
f17d	Your partner will disclose your HIV status to your close friends/family in the next 3 months	________________
SELF REPORTED HEALTH AND HAPPINESS		
Intro Note	Now I'd like to talk to you about how healthy and happy you feel.	

For peer review only

e1	I am interested in your general level of well-being or satisfaction with life. How satisfied are you with your life, all things considered?	1. Very satisfied<br>2. Somewhat satisfied<br>3. Neutral<br>4. Somewhat unsatisfied<br>5. Very unsatisfied
e2	Do you think that you are more, equally or less satisfied than other persons your age and sex living in your village?	1. More satisfied<br>2. Equally satisfied<br>3. Less satisfied
e3	In general, would you say your health now is: very good, good, poor or very poor?	1. Very good<br>2. Good<br>3. Poor<br>4. Very poor
e4	How would you compare your health to other people of the same age and sex in your village?	1. More healthy<br>2. Equally healthy<br>3. Less healthy
e5	In the past month, how many days were you too sick to work/go to school/complete household chores?	________________
Happiness		
e6	How true are the following statements for you in the last month?	
e6_1	I have felt depressed	1. Strongly Agree<br>2. Agree<br>3. Disagree<br>4. Strongly Disagree
e6_2	I have felt life was not worth living	1. Strongly Agree<br>2. Agree<br>3. Disagree<br>4. Strongly Disagree
e6_3	I have felt content.	1. Strongly Agree<br>2. Agree<br>3. Disagree<br>4. Strongly Disagree
e6_4	I have felt lonely	1. Strongly Agree<br>2. Agree<br>3. Disagree<br>4. Strongly Disagree
GENDER EQUITABLE MEN SCALE		

For peer review only

Note	Please tell me if you strongly agree, agree, disagree, or strongly disagree with the following statements:	
j1	Woman's most important role is to take care of her home and cook (take care of home is about housekeeping)	1. Strongly Agree<br>2. Agree<br>3. Unsure<br>4. Disagree<br>5. Strongly Disagree<br>88. Refuse to say
j2	Men need sex more than women	1. Strongly Agree<br>2. Agree<br>3. Unsure<br>4. Disagree<br>5. Strongly Disagree<br>88. Refuse to say
j3	Men don't talk about sex, they just do it.	1. Strongly Agree<br>2. Agree<br>3. Unsure<br>4. Disagree<br>5. Strongly Disagree<br>88. Refuse to say
j4	There are times when a woman deserves to be beaten	1. Strongly Agree<br>2. Agree<br>3. Unsure<br>4. Disagree<br>5. Strongly Disagree<br>88. Refuse to say
j5	Changing diapers, giving kids a bath & feeding kids are mother's responsibility	1. Strongly Agree<br>2. Agree<br>3. Unsure<br>4. Disagree<br>5. Strongly Disagree<br>88. Refuse to say
j6	It is a woman's responsibility to avoid getting pregnant	1. Strongly Agree<br>2. Agree<br>3. Unsure<br>4. Disagree<br>5. Strongly Disagree<br>88. Refuse to say

For peer review only

j7	A man should have the final word about decisions in his home	1. Strongly Agree<br>2. Agree<br>3. Unsure<br>4. Disagree<br>5. Strongly Disagree<br>88. Refuse to say
j8	Men are always ready to have sex	1. Strongly Agree<br>2. Agree<br>3. Unsure<br>4. Disagree<br>5. Strongly Disagree<br>88. Refuse to say
j9	A woman should tolerate violence in order to keep her family together	1. Strongly Agree<br>2. Agree<br>3. Unsure<br>4. Disagree<br>5. Strongly Disagree<br>88. Refuse to say
j10a	I would be outraged if my wife asked me to use a condom.	1. Strongly Agree<br>2. Agree<br>3. Unsure<br>4. Disagree<br>5. Strongly Disagree<br>88. Refuse to say
j10b	Men would be outraged if their wife asked them to use a condom	1. Strongly Agree<br>2. Agree<br>3. Unsure<br>4. Disagree<br>5. Strongly Disagree<br>88. Refuse to say
j11	A man and a woman should decide together what type of contraceptive to use	1. Strongly Agree<br>2. Agree<br>3. Unsure<br>4. Disagree<br>5. Strongly Disagree<br>88. Refuse to say
j12	I would never have a homosexual friend	1. Strongly Agree<br>2. Agree<br>3. Unsure<br>4. Disagree

For peer review only

		5. Strongly Disagree<br><br>88. Refuse to say
j13a	If someone insults me, I will defend my reputation, with force if I have to.	1. Strongly Agree<br>2. Agree<br>3. Unsure<br>4. Disagree<br>5. Strongly Disagree<br><br>88. Refuse to say
j13b	If someone insults a man, he should defend his reputation, with force if he has to	1. Strongly Agree<br>2. Agree<br>3. Unsure<br>4. Disagree<br>5. Strongly Disagree<br><br>88. Refuse to say
j14	To be a man you need to be tough.	1. Strongly Agree<br>2. Agree<br>3. Unsure<br>4. Disagree<br>5. Strongly Disagree<br><br>88. Refuse to say
j15	Men should be embarrassed if unable to get an erection	1. Strongly Agree<br>2. Agree<br>3. Unsure<br>4. Disagree<br>5. Strongly Disagree<br><br>88. Refuse to say
j16	If a guy gets a woman pregnant, child is the responsibility of both the man and woman	1. Strongly Agree<br>2. Agree<br>3. Unsure<br>4. Disagree<br>5. Strongly Disagree<br><br>88. Refuse to say
j17	A man should know what his partner likes during sex	1. Strongly Agree<br>2. Agree<br>3. Unsure<br>4. Disagree<br>5. Strongly Disagree<br><br>88. Refuse to say

For peer review only

j18	The participation of the father is important in raising children	1. Strongly Agree<br>2. Agree<br>3. Unsure<br>4. Disagree<br>5. Strongly Disagree<br><br>88. Refuse to say
j19	It's important for men to have friends to talk about their problems	1. Strongly Agree<br>2. Agree<br>3. Unsure<br>4. Disagree<br>5. Strongly Disagree<br><br>88. Refuse to say
j20	A couple should decide together if they want to have children.	1. Strongly Agree<br>2. Agree<br>3. Unsure<br>4. Disagree<br>5. Strongly Disagree<br><br>88. Refuse to say
Comment	We have reached the end of the chat. Thank you for your time. Do you have anything else you would like to say?	
End Note	Thank the participant for their time and give them transport reimbursement, if they did not come for an ART appointment	
Comments	Enumerator comments	
End of the survey!		

For peer review only

BASELINE SURVEY
Identifying efficient linkages strategies for HIVST (IDEaL)
Female (Chichewa)

**Question Name**	**Label**	**Responses**
INTRODUCTION SECTION		
interviewer	Full Name of Interviewer	
Interview date	Interview date	
Time start	Time survey started	
District	District	
Facility	Facility	
ID	ID	
SECTION A: DEMOGRAPHICS		
Intro Note	Zikomo povomera kutenga nawo mbali. Pa nthawi ino ndikufunsani ma funso ochepa okhudza za inu komanso kuti ndinu ndani. Chonde khalani omasuka kuyankha moona. Palibe yankho lokhoza komanso lolakwa.	
a7	Ndinu mtundu wanji wa munthu?	1. Lomwe<br>2. Sena<br>3. Chewa<br>4. Mang'anja/Nyanja<br>5. Ngoni<br>6. Tumbuka<br>7. Tonga<br>8. Yao<br>99. Other, specify

For peer review only - http://bmjopen.bmj.com/site/about/guidelines.xhtml

a3	Kodi maphunziro anu mudapita nawo patali bwanji?	1. Pulayimale<br>2. Sekondale<br>3. Koleji
a3b	Ndi kalasi liti munamaliza ya maphunziro anu apamwamba?	______________________
a4	Chonde ganizani za masabata khumi ndi awiri apitawa, mungafotokoze bwanji za ntchito yomwe mumagwira?	1. Ntchito yokhazikika<br>2. Ganyu/bisinesi<br>3. Sindikugwira ntchito
a5	Kodi pakadali pano muli pa banja?	1. Pa banja<br>2. Kukhala limodzi ngati banja<br>3. Chibwezi Chokhazikika<br>4. Tinasiyana<br>5. Banja linatha<br>99. Zina
a6	Muli ndi ana angati amoyo?	________________________
a6b	Mwana wanu wang'ono ali ndi zaka zingati?	_________________________
a6bc	Mwana wanu wang'ono ali ndi zaka zingati?	_________________________
a4b	Pakadali pano ana omwe mumakhala nawo ndi angati?	_________________________
a7	Pa miyezi khumi ndi iwiri yapitayi mwakhala ndi abwenzi ogonana nawo angati?	_________________________
a8	Mwakhalapo ndi bwenzi logonana kupatula akazi anu?amuna anu osagwiritsa ntchito kondomu mu miyezi khumi ndi awiri yapitayi?	3. Eya<br>4. Ayi<br>88. Sindikudziwa<br>89. Akana kuyankha
a8b	Mwagonanapo ndi munthu osagwiritsa ntchito kondomu mu miyezi khumi ndi awiri yapitayi?	3. Eya<br>4. Ayi<br>88. Sindikudziwa<br>89. Akana kuyankha

For peer review only

SECTION B: INCOME QUESTIONS		
Intro Note	Pa nthawi ino ndikufunsani za zipangizo zomwe inu komanso apabanja panu alinazo. Mkati mwakucheza kwathu ndikufunsaninso za ndalama zomwe mulinazo komanso komanso zomwe mumachita kuti mupeze ndalama.	
b1	Chonde ganizirani za miyezi Khumi ndi iwiri yapitayi, mungafotokoze bwanji za ntchito yomwe mumagwira?	1. Ntchito yokhazikika<br>2. Ganyu/bisinesi<br>3. Sindikugwira ntchito
b1b	Ganizani za ntchito zonse mwagwira mwezi watha. Mwagwira masiku angati olipidwa?	____________________
b2	Muli ndi ndalama zilizonse zomwe mukusungira za mtsogolo monga, ku banki, gulu losugira ndalama kapena ndalama zosunga kunyumba?	2. Eya<br>3. Ayi
Household Assets		
b3	Does your household have:<br>*The respondent said that his/her household doesn't have any of the household assets. Please probe and ensure that this is correct before you proceed.*	
b3_1	Denga la malata?	1. Eya<br>2. Ayi
b3_2	Magetsi ?	3. Eya<br>4. Ayi
b3_3	Koloboyi?	3. Eya<br>4. Ayi
b3_4	Nyali?	3. Eya<br>4. Ayi
b3_5	Wailesi?	3. Eya<br>4. Ayi
b3_6	Kanema?	3. Eya<br>4. Ayi
b3_7	Lamya ya M’manja?	3. Eya<br>4. Ayi
b3_8	Kama?	3. Eya

For peer review only

		4. Ayi
b3_9	Sofa?	3. Eya<br>4. Ayi
b3_10	Tebulo?	3. Eya<br>4. Ayi
b3_11	FIliji?	3. Eya<br>4. Ayi
b3_12	Matilesi?	3. Eya<br>4. Ayi
b3_13	Mipando	3. Eya<br>4. Ayi
b3_14	Ng'ombe?	3. Eya<br>4. Ayi
b3_15	Mbuzi?	3. Eya<br>4. Ayi
b3_16	Nkhosa?	3. Eya<br>4. Ayi
b3_17	Nkhumba?	3. Eya<br>4. Ayi
b3_18	Bulu?	3. Eya<br>4. Ayi
b3_19	Nkhuku?	3. Eya<br>4. Ayi
b3_20	Zoweta zina	3. Eya<br>4. Ayi
b4	Mu masiku makumi atatu apitawa mwamako mowa?	3. Eya<br>4. Ayi
b4b	Pa masiku makumi atatu apitawa mwamwa mowa masiku angati?	________________________
b4c	Mwataya ndalama zingati masiku omaliza omwe munapita ku mowa?	MWK:
b4d	Zonse pamodzi, mwataya ndalama zingati pa mowa mu masiku makumi atatu apitawa?	MWK:
Relationship		
Intro Note	Panthawi ino ndikufunsani za abwezi ogonana nawo?	
f8	Mwakhala pa ubwenzi ogonana ndi bwenzi lanu kwa nthawi yayitali bwanji?	Masiku ____________<br>Miyezi ______________

For peer review only

		Zaka_______________
f9	Muli ndi ana ndi bwenzi lanu logonana nalo? Ana angati?	_____________________
f10	Mumayankhulana mowirikiza bwanji ndi bwenzi lanu pakadali pano?	8. Tsiku ndi tsiku<br>9. Masiku angapo pasabata<br>10. Kamodzi pa sabata<br>11. Kangapo pa mwezi<br>12. Kamodzi pa mwezi<br>13. Kosakwana mwezi<br>14. Sitiyankhulani
f10b	Pa mwezi amalandila ndalama zambiri ndi ndani? Inu kapena bwenzi lanu?	5. Ine<br>6. Bwenzi langa<br>7. Timalandira ndalama zofanana<br>8. Sindikudziwa
Decision Making		
Intro Note	Pa nthawi ino ndikufunsani za momwe mumapangira maganizo ndi bwenzi lanu	
f11	Nthawi zambiri ndi ndani amene amapanga chiganizo cha momwe ndalama mumapeze zigwiritsidwe ntchito?	6. Ine<br>7. Timagwirizana<br>8. Nthawi zambiri bwenzi langa<br>9. Munthu wina<br>10. Sindipeza ndalama<br>89. Sindikufuna kuyankha
f11b	(Ngati yankho ndi 4) Amapanga chiganizo ndi ndani?	_____________________
f12	Amapanga chiganizo cha momwe ndalama za bwenzi lanu zigwiritsidwe ntchito ndi ndani?	6. Ine<br>7. Timagwirizana<br>8. Nthawi zambiri bwenzi langa<br>9. Munthu wina<br>10. Sindipeza ndalama<br>89. Sindikufuna kuyankha
f12b	(Ngati yankho ndi 4)	_____________________

For peer review only

f13	Nthawi zambiri amapanga chiganizo chokhudza thandizo la zaumoyo wanu ndi ndani?	6. Ine<br>7. Timagwirizana<br>8. Nthawi zambiri bwenzi langa<br>9. Munthu wina<br>10. Sindipeza ndalama<br>89. Sindikufuna kuyankha
f13b	(Ngati yankho ndi 4) Amapanga chiganizo ndi ndani?	________________________
f14	Nthawi zambiri amapanga chiganizo cha thandizo la zaumoyo la mwana yemwe muli naye ndi bwenzi lanu ndi ndani?	6. Ine<br>7. Mogwirizana<br>8. Nthawi zambiri bwenzi langa<br>9. Munthu wina<br>10. Ndilibe mwana<br>88. Sindikufuna kuyankha
f14b	(if above question=4) Who decides?	________________________
f15	Nthawi zambiri amapanga chiganizo chokhudza thandizo la zaumoyo la bwenzi lanu ndi ndani?	1. Ine<br>2. Mogwirizana<br>3. Nthawi zambiri bwenzi langa<br>4. Munthu wina<br>5. Ndilibe ana<br>88.Sindikufuna kuyankha
f15b	(Ngati yankho ndi 4) Amapanga chiganizo ndi ndani?	________________________
f16	Kodi ndi ndani amene amapanga ziganizo zogula katundu mkulumkulu wapa khomo panu?	1. Ine<br>2. Mogwirizana<br>3. Nthawi zambiri bwenzi langa<br>4. Munthu wina<br>5. Ndilibe ana<br>88.Sindikufuna kuyankha
f16b	(Ngati yankho ndi 4) Amapanga	

For peer review only

	chiganizo ndani?	
Note	Ndikufuna ndikufunseni zokhudzana za (Kuthekela/mwayi) kuti zinthu zina zichitike. Muli nyemba mu kapu. Ndikufunsani kuti musankhe zina mwa nyemba ndipo muyike mu mbale. Mulingo wa nyemba omwe muyike mu mbale udzafanizira kuti chinachake chichitika. Nyemba imodzi ikusonyeza kuti mwayi ndiochepa kuti chinachake chichitika. Ngati simuika nyemba mm'bale ndekuti mukutsimikiza kuti palibe mwayi oti chinachake chichitika	
note2	Ngati muyike nyemba zowonjezera mu mbale, zikutanthauza kuti mwayi oti chinachake chichitika uchuluka, mwachitsanzo ngati muyika nyemba imodzi kapena ziwiri mwayi oti chinachake chichitika. Ngakhale pali mwayi ochepa koma chinachake chichitika. Ngati muyike nyemba nkhumi zikutanthauza kuti pali mwayi ofanana oti chinachake chichika kapena ayi. Ngati muyike nyemba zisanu ndi imodzi zikutanthauza kuti mwayi woti chinachake chichitika uli ochulukilapo kuposa mwayi oti chinachake sichichitika. Ngati muyike nyemba zonse khumi ndekuti muli ndi chikhulupiliro chonse kuti chinachake chichitikadi pavute pasavute. Palibe yankho lokhonza kapena lolakwa ndikungofuna kudziwa zomwe mukuganiza.	
note3	Ofunsa: Pelekani yankho lanu pa funso lililonse nambala ya nyemba zomwe lili m'bale. Pakutha pa funso lililonse, bwezeletsani nyemba pa tebulo.	
Practice		

For peer review only

pr1	Sankhani mulingo wa nyemba omwe ukhale ndi kuthekela kumene mukuganiza kuti:	________________
pr1b	Mupita ku msika mosachepera kamodzi m'masiku awiri akudzawa	________________
pr1c	Mupita ku msika mosachepera kamodzi m'masabata awiri akudzawa.	________________
Practice		
pr2	Ofunsa: kodi oyankha anawonjezera nyamba pakati pa pr1b ndi pr1c	3. Eya<br>1. Ayi
pr3	Kumbukurani kuti pamene nthawi ikupita muzipeza mpata wambiri opita kunsika. Choncho, munayenela kuti mwaika nyemba zambiri m'bale<br><br>Kodi mukuganiza kuti kumsika mupita mosachepela kamodzi bwanji mu nyengo ya ma sabata awiriwa?	________________
f17	Sankhani mlingo wa nyemba umene ufanizile kaganidwe kanu:	

For peer review only

f17a	Mudzakhalabe pabanja ndi bwenzi lanu m'chaka chimodzi chikubwerachi	________________
f17b	Bwenzi lanu lidzapezeka ndi matenda a HIV m'miyezi khumi ndi iwiri ikubwerayi	________________
f17c	Bwenzi lanu lidzayamba kumwa ma ARV m'miyezi itatu ikubwerayi	________________
f17d	Bwenzi lanu lidzaulula kuti inuyo muli ndi kachilombo ka HIV kwa anzanu apamtima/abale anu m'miyezi itatu ikubwerayi	________________
SELF REPORTED HEALTH AND HAPPINESS		
Intro Note	Pa nthawi ino ndikufuna ndikambe nanu zokhudza umoyo ndi chisangalalo chanu.	
e1	Ndili ndi chidwi ndi mukudziwa za umoyo ndi kukhutitsidwa kwanu. Kodi muli okhutitsidwa bwanji ndi moyo wanu, pakutengela zonse.	6. Okhutitsidwa kwamburi<br>7. Okhutitsidwa pang'ono<br>8. Pakatikati<br>9. Okhutitsidwa pang'ono<br>10. Osakhutitsidwa olo pang'ono
e2	Kodi mukuona ngati muli okhutitsidwa mofanana kapena osakhutitsidwa pang'ono mosaposela anthu ena a muna kapena akazi a msinkhu wanu ndi opezeka m'mudzi mwanu?	4. Okhutitsidwa kwambiri<br>5. Okhutiotsidwa<br>6. Okhutitsidwa pang'ono
e3	Kutengela zonse, Kodi munganene kuti umoyo wanu tsopano uli bwino kwambiri, ulibwino, sulibwino, suli bwino olo pang'ono	5. Bwino kwambiri<br>6. Bwino<br>7. Silibwino<br>8. SIlibwino olo pang'ono
e4	Kodi mungazifananizile bwanji za thanzi lanu ndi la anthu ena a msikhu ofanana ndi wanu, amuna kapena	4. A thanzi kwambiri<br>5. A thanzi<br>6. Nthanzi lochepekela

	akazi a m'mudzi mwanu.	
e5	Kodi mwezi wathawu, ndi masiku angati omwe munadwara kwambiri ofika kukulepheretsani kugwira ncthito/kupita ku sukulu/kugwira ntchito za pa khomo	________________
Happiness		
e6	Kodi ziganizo izi ndi zowona bwanji kwa inu?	
e6_1	Ndinali ndi nkhawa	5. Ndikugwirizana nazo kwambiri<br>6. Ndikugwirizana nazo<br>7. Sindikugwirizana nazo<br>8. Sindikugwirizana nazo kwambiri
e6_2	Ndimanva ngati moyo wafika	5. Ndikugwirizana nazo kwambiri<br>6. Ndikugwirizana nazo<br>7. Sindikugwirizana nazo<br>8. Sindikugwirizana nazo kwambiri
e6_3	Ndimanva kukhutitsidwa	5. Ndikugwirizana nazo kwambiri<br>6. Ndikugwirizana nazo<br>7. Sindikugwirizana nazo<br>8. Sindikugwirizana nazo kwambiri
e6_4	Ndimanva kusalidwa	5. Ndikugwirizana nazo kwambiri<br>6. Ndikugwirizana nazo<br>7. Sindikugwirizana nazo<br>8. Sindikugwirizana nazo kwambiri
GENDER EQUITABLE MEN SCALE		
Note	Chonde ndiuzeni ngati mukugwirizana nazo kwabiri, mukugwirizana nazo, simukugwirizana nazo, simukugwirizana nazo olo pang'ono ziganizo izi:	

For peer review only

j1	Udindo ofunikila wa mzimayi ndi kusamala khomo lake ndi kuphika.	6. Ndikugwirizana nazo kwambiri<br>7. Ndikugwirizana nazo<br>8. Sindikukhulupilira<br>9. Sindikugwirizana nazo<br>10. Sindikugwirizana nazo olo pang'ono<br>89. Sindikufuna kuyankha
j2	Abambo amafuna kugonana kuposa amayi	6. Ndikugwirizana nazo kwambiri<br>7. Ndikugwirizana nazo<br>8. Sindikukhulupilira<br>9. Sindikugwirizana nazo<br>10. Sindikugwirizana nazo olo pang'ono<br>88. Sindikufuna kuyankha
j3	Azibambo sakonda kukambirana za kugonana amangochita	6. Ndikugwirizana nazo kwambiri<br>7. Ndikugwirizana nazo<br>8. Sindikukhulupilira<br>9. Sindikugwirizana nazo<br>10. Sindikugwirizana nazo olo pang'ono<br>88. Sindikufuna kuyankha
j4	Nthawi zina mzimayi amayenela kumenyedwa.	6. Ndikugwirizana nazo kwambiri<br>7. Ndikugwirizana nazo<br>8. Sindikukhulupilira<br>9. Sindikugwirizana nazo<br>10. Sindikugwirizana nazo olo pang'ono<br>88. Sindikufuna kuyankha
j5	Kusinta matewela, kusambitsa mwana, kudyetsa mwana ndi udindo wa mzimayi	6. Ndikugwirizana nazo kwambiri<br>7. Ndikugwirizana nazo<br>8. Sindikukhulupilira<br>9. Sindikugwirizana nazo<br>10. Sindikugwirizana nazo olo pang'ono<br>88. Sindikufuna kuyankha
j6	Ndi udindi wa mzimayi kupewa	6. Ndikugwirizana nazo kwambiri

For peer review only

	kutenga mimba.	7. Ndikugwirizana nazo<br>8. Sindikukhulupilira<br>9. Sindikugwirizana nazo<br>10. Sindikugwirizana nazo olo pang'ono<br>88. Sindikufuna kuyankha
j7	Mzibambo ayenela kukhala ndi chiganizo chomaliza cha mnyumba mwake.	6. Ndikugwirizana nazo kwambiri<br>7. Ndikugwirizana nazo<br>8. Sindikukhulupilira<br>9. Sindikugwirizana nazo<br>10. Sindikugwirizana nazo olo pang'ono<br>88. Sindikufuna kuyankha
j8	Azibambo amakhala okonzeka ku gonana	6. Ndikugwirizana nazo kwambiri<br>7. Ndikugwirizana nazo<br>8. Sindikukhulupilira<br>9. Sindikugwirizana nazo<br>10. Sindikugwirizana nazo olo pang'ono<br>Sindikufuna kuyankha
j9	Mzimayi akuyenela kulekelela nkhaza kuti asunge banja lake.	6. Ndikugwirizana nazo kwambiri<br>7. Ndikugwirizana nazo<br>8. Sindikukhulupilira<br>9. Sindikugwirizana nazo<br>10. Sindikugwirizana nazo olo pang'ono<br>Sindikufuna kuyankha
j10a	Ndikhoza kukwiya akaza anga atati andiuze kuti ndigwiritse ntchito kondumu.	6. Ndikugwirizana nazo kwambiri<br>7. Ndikugwirizana nazo<br>8. Sindikukhulupilira<br>9. Sindikugwirizana nazo<br>10. Sindikugwirizana nazo olo pang'ono<br>88. Sindikufuna kuyankha
j10b	Azibambo akhoza kukwiya akazi awo atawauza kuti agwiritse ntchito	6. Ndikugwirizana nazo kwambiri

For peer review only

	kondomu	7. Ndikugwirizana nazo<br>8. Sindikukhulupilira<br>9. Sindikugwirizana nazo<br>10. Sindikugwirizana nazo olo pang'ono<br>88. Sindikufuna kuyankha
j11	Mzimayi komanso nzibambo agwirizane limodzi kuti agwiritse ntchito njira yanji yakulela	6. Ndikugwirizana nazo kwambiri<br>7. Ndikugwirizana nazo<br>8. Sindikukhulupilira<br>9. Sindikugwirizana nazo<br>10. Sindikugwirizana nazo olo pang'ono<br>88. Sindikufuna kuyankha
j12	Sindingakhale ndi nzanga wopanga zamathanyula	6. Ndikugwirizana nazo kwambiri<br>7. Ndikugwirizana nazo<br>8. Sindikukhulupilira<br>9. Sindikugwirizana nazo<br>10. Sindikugwirizana nazo olo pang'ono<br>88. Sindikufuna kuyankha
j13a	Ngati munthu angandinyoze, ndiziteteza pogwiritsa ntchito mphanvu, ngati ndikufunika kutero.	6. Ndikugwirizana nazo kwambiri<br>7. Ndikugwirizana nazo<br>8. Sindikukhulupilira<br>9. Sindikugwirizana nazo<br>10. Sindikugwirizana nazo olo pang'ono<br>88. Sindikufuna kuyankha
j13b	Ngati munthu anganyoze mzibambo, aziteteze pogwiritsa ntchito mphanvu ngati akufunika kutelo.	6. Ndikugwirizana nazo kwambiri<br>7. Ndikugwirizana nazo<br>8. Sindikukhulupilira<br>9. Sindikugwirizana nazo<br>10. Sindikugwirizana nazo olo pang'ono<br>Sindikufuna kuyankha
j14	Kuti ukhale mzibambo ukufunika kukhala ovuta.	6. Ndikugwirizana nazo kwambiri<br>7. Ndikugwirizana nazo<br>8. Sindikukhulupilira

		9. Sindikugwirizana nazo<br>10. Sindikugwirizana nazo olo pang'ono<br>Sindikufuna kuyankha Sindikugwirizana nazo kwambiri<br>88. Refuse to say<br>Sindikufuna kuyankha
j15	Azibambo akuyenela kunva manyazi ngati akukanika kutota	6. Ndikugwirizana nazo kwambiri<br>7. Ndikugwirizana nazo<br>8. Sindikukhulupilira<br>9. Sindikugwirizana nazo<br>10. Sindikugwirizana nazo olo pang'ono<br>88. Sindikufuna kuyankha
j16	Ngati mzibambo wapeleka mimba kwa mzimayi, mwanayo ndi udindo wa anthu onse a wiri.	6. Ndikugwirizana nazo kwambiri<br>7. Ndikugwirizana nazo<br>8. Sindikukhulupilira<br>9. Sindikugwirizana nazo<br>10. Sindikugwirizana nazo olo pang'ono<br>88. Sindikufuna kuyankha
j17	Nzibambo akuyenela kudziwa zomwe bwenzi lake limakonda pogonana	6. Ndikugwirizana nazo kwambiri<br>7. Ndikugwirizana nazo<br>8. Sindikukhulupilira<br>9. Sindikugwirizana nazo<br>10. Sindikugwirizana nazo olo pang'ono<br>88. Sindikufuna kuyankha
j18	Kutenga nawo mbali kwa a bambo ndi kofunika polela mwana	6. Ndikugwirizana nazo kwambiri<br>7. Ndikugwirizana nazo<br>8. Sindikukhulupilira<br>9. Sindikugwirizana nazo<br>10. Sindikugwirizana nazo olo pang'ono<br>88. Sindikufuna kuyankha

For peer review only

j19	Ndi zofunika kuti abambo azikhala ndi anzawo okambilana nawo mavuto awo.	6. Ndikugwirizana nazo kwambiri<br>7. Ndikugwirizana nazo<br>8. Sindikukhulupilira<br>9. Sindikugwirizana nazo<br>10. Sindikugwirizana nazo olo pang'ono<br>88. Sindikufuna kuyankha
j20	Banja lizigwirizana limodzi ngati likufuna kukhala ndi mwana	6. Ndikugwirizana nazo kwambiri<br>7. Ndikugwirizana nazo<br>8. Sindikukhulupilira<br>9. Sindikugwirizana nazo<br>10. Sindikugwirizana nazo olo pang'ono<br>88. Sindikufuna kuyankha
Comment	Tafika pamapeto a kucheza kwathu. Zikomo chifukwa cha nthawi yanu. Pali chili chonse mukufuna kuwonjezera?	
End Note	Thank the participant for their time and give them transport reimbursement, if they did not come for an ART appointment	
Comments	Enumerator comments	
End of the survey!		

For peer review only

14.7. APPENDIX G: Follow-up Survey - Male

FOLLOW-UP SURVEY
Identifying efficient linkages strategies for HIVST (IDEaL)
Male

Complete this form for men who enrolled in the study 4-months ago

Date of Interview: ____________________
Site Code: ____________________________

Full Name of Interviewer: ______________________________

Participant Study ID#: ___________

#	Question	Response
c1a	Please think about your primary partner in the last 4 months.<br><br>Has your relationship changed in the last 4 months? How?	☐ Nothing changed (1)<br>☐ Married (2)<br>☐ Steady Girlfriend (3)<br>☐ Moved out of the house (4)<br>☐ Became an infrequent partner (5)<br>☐ Separated (6)<br>☐ Divorced (7)<br>☐ Other (8)
c4a	Have you disclosed your HIV status to this partner?	☐ Yes (1)<br>☐ No (0)
c5a	Have you had any new children with this partner since we last spoke?	☐ Yes (1)<br>☐ No (0)
c18a	Have you started ART in the past 4months?	☐ Yes (1)<br>☐ No (0)<br>☐ No, but I plan to link

For peer review only

c18b	IF YES: When did you start ART?	______________<br>Day/Month/Year
	**Unintended Consequences**	
c19a	Were unwantedly pressured to initiate ART?	☐ Yes (1)<br>☐ No (0)
c20a	After enrolling in the study, did anyone find out your HIV status against your will (unwanted disclosure)?	☐ Yes (1)<br>☐ No (0)
C20b	After enrolling in the study, did anyone find out your partners' HIV status against her will (unwanted disclosure)?	☐ Yes (1)<br>☐ No (0)
c21a	After enrolling in the study, did your partner….<br><br>Threaten to hurt or harm you or someone you cared about.	☐ Yes (1)<br>☐ No (0)<br>☐ Refused to respond (99)
c22a	Insulted you or made you feel bad about yourself.	☐ Yes (1)<br>☐ No (0)<br>☐ Refused to respond (99)
c23a	Hit, slapped, kicked or did anything else meant to physically hurt you.	☐ Yes (1)<br>☐ No (0)<br>☐ Refused to respond (99)
c21a	After enrolling in the study, did you ever do the following to your partner….<br><br>Threaten to hurt or harm her or someone she cared about.	☐ Yes (1)<br>☐ No (0)<br>☐ Refused to respond (99)

For peer review only

c22a	Insulted her or made her feel bad about herself.	☐ Yes (1)<br>☐ No (0)<br>☐ Refused to respond (99)
c23a	Hit, slapped, kicked or did anything else meant to physically hurt her.	☐ Yes (1)<br>☐ No (0)<br>☐ Refused to respond (99)
c24	Slept with another woman	☐ Yes (1)<br>☐ No (0)<br>☐ Refused to respond (99)
c25a	*Now, I am going to ask you a series of questions about who makes within this relationship. Please think about the last 4 months*<br><br>Who usually decides how the money you earn will be used?	☐ Yourself (respondent)<br>☐ Jointly (This partner and you together)<br>☐ Mainly this partner<br>☐ Someone else<br>☐ Do not earn money<br>☐ Refuse to say
c26a	Who usually decides how your partner's earnings will be used?	☐ Yourself (respondent)<br>☐ Jointly (This partner and you together)<br>☐ Mainly this partner<br>☐ Someone else<br>☐ Refuse to say

For peer review only

c27a	Who usually makes decisions about health care for yourself?	☐ Yourself (respondent)<br>☐ Jointly (This partner and you together)<br>☐ Mainly this partner<br>☐ Someone else<br>☐ Refuse to say

	**D. Additional Questions**	
#	**Question**	**Response**
d1	Would you recommend the ART intervention you were part of to other male friends or family?	☐ Yes (1)<br>☐ No (0)
d2	Are you happy that you participated in the ART intervention?	☐ Yes (1)<br>☐ No (0)

Thank the participant for their time and end the survey

For peer review only

BASELINE SURVEY
Identifying efficient linkages strategies for HIVST (IDEaL)
Male (Chichewa)

Complete this form for men who enrolled in the study 4-months ago

Date of Interview: ____________________
Site Code: _________________________
Full Name of Interviewer: ____________________________
Participant Study ID#: ___________

For peer review only

#	Question	Response
c1a	Taganizani za bwenzi lanu logonana nalo lomwe lakhala lodalilika kwa miyesi folo yapitayi.<br><br>Kodi ubwenzi wanu wasintha munjira ina iliyonse mu miyezi folo yapitayi?	☐ Palibe chasintha (1)<br>☐ Married (2)<br>☐ Chibwenzi chokhazikika (3)<br>☐ Chibwenzi Chinachoka pakhomo (4)<br>☐ Chibwenzi cha apo ndi apo (5)<br>☐ Separated (6)<br>☐ Banja linatha (7)<br>☐ Other (8)
c4a	Kodi munawauza abwenzi anuwa za mmene mulili mnthupi mwanu ku mbali ya HIV?	☐ Eya(1)<br>☐ Ayi(0)
c5a	Kodi mwakhala ndi ana ndi abwenzi anuwa kuchokera ulendo watha tinayankhulana?	☐ Eya(1)<br>☐ Ayi(0)
c18a	Kodi munayamba kumwa mankhwala a ma ARV mu miyezi folo yapitayi?	☐ Eya(1)<br>☐ Ayi(2)<br>☐Ayi, ndikulingalira zoyamba (3)

c18b	Ngati eya: Munayamba liti mankwala a ma ARV?	______<br>Tsiku/Mwezi/Chaka
**Unintended Consequences**		
c19a	Munayamba kumwa mankhwala a ma ARV mokakamizidwa?	☐ Eya(1)<br>☐ Ayi(0)
c20a	Chilowereni mu study, alipo omwe anadziwa za m'mene mulili mnthupi mwanu kumbari ya HIV inu musakufuna? (Kuwulula za HIV mosafuna)	☐ Eya(1)<br>☐ Ayi(0)
C20b	Chilowereni mu study, alipo omwe anadziwa za m'mene mulili mnthupi mwa bwenzi lanu kumbari ya HIV eni asakufuna? (Kuwulula za HIV mosafuna)	☐ Eya(1)<br>☐ Ayi(0)
c21a	Chilowereni mu study, kodi bwenzi lanu…..<br><br>Linaospyeza kuvulaza inu kapena wina aliyense amane mumamukonda?	☐ Eya(1)<br>☐ Ayi(0)<br>☐ Refused to respond (99)
c22a	Linakunyozani kapena kukunyogodolani	☐ Eya(1)<br>☐ Ayi(0)<br>☐ Refused to respond (99)
c23a	Anakumenyani ndikukupwetekani.	☐ Eya(1)<br>☐ Ayi(0)<br>☐ Refused to respond (99)
c21a	Chilowereni mu study kodi munayamba mwapangapo zotsatirazi kwa bwenzi anu….<br><br>Kuosyeza kuti muvulaza bwezi lanu kapena wina aliyense yemwe amamukonda.	☐ Eya(1)<br>☐ Ayi(0)<br>☐ Refused to respond (99)

c22a	Kunyoza kapenanso kumupangitsa kuti azizikayikila.	☐ Eya(1)<br>☐ Ayi(0)<br>☐ Refused to respond (99)
c23a	Kumumenya ndikumupweteka.	☐ Eya(1)<br>☐ Ayi(0)<br>☐ Refused to respond (99)
c24	Kuchita mchitidwe ogonana ndi mzimayi wina	☐ Eya(1)<br>☐ Ayi(0)<br>☐ Refused to respond (99)
c25a	Tsopano ndikufunsani mafunso okhuzana ndi omwe amalamula pakhomo panu.<br><br>Kodi amalamula za mmene ndalama zomwe mwapeza zitagwilitsidwile ntchito ndi ndani?	☐ Amene akuyankha<br>☐ Mogwirizana<br>☐ Nthawi zambiri bwenzi<br>☐ Munthu wina<br>☐ Sapeza ndalama<br>☐ Refuse to say
c26a	Kodi amalamulila za mmene ndalama za abwenzi anu zingagwilitsidwile ntchito ndi ndani?	☐ Amene akuyankha<br>☐ Mogwirizana<br>☐ Nthawi zambiri bwenzi<br>☐ Munthu wina<br>☐ Refuse to say
c27a	Kodi amene amakhala ndi ulamulilo pa chisamalilo cha moyo wanu ndi ndani?	☐ Amene akuyankha<br>☐ Mogwirizana<br>☐ Nthawi zambiri bwenzi<br>☐ Munthu wina<br>☐ Refuse to say

For peer review only

	Additional Questions	
d1	Kodi mungalimbikitse anzanu ena achizibambo kapena apabanja panu kutenga nawo mbali mu ART intervention munaliyi?	☐ Eya(1)<br>☐ Ayi(0)
d2	Kodi muli okondwa kuti munatenga nawo mbali mu ART intervention?	☐ Eya(1)<br>☐ Ayi(0)

For peer review only

14.8. APPENDIX H: Follow-up Survey – Female

FOLLOW-UP SURVEY
Identifying efficient linkages strategies for HIVST (IDEaL)
Female

Complete this form for women whose:

(1) Partners consented to be in the study 4-months ago

Date of Interview: ____________________
Site Code: _________________________

Full Name of Interviewer: ______________________________

Participant Study ID#: ___________

#	Question	Response
c1a	Please think about your primary partner in the last 4 months.<br><br>Has your relationship changed in the last 4 months? How?	☐ Nothing changed (1)<br>☐ Married (2)<br>☐ Steady Boyfriend (3)<br>☐ Moved out of the house (4)<br>☐ Became an infrequent partner (5)<br>☐ Separated (6)<br>☐ Divorced (7)<br>☐ Other (8)
c4a	Have you disclosed your HIV status to this partner?	☐ Yes (1)<br>☐ No (0)
c5a	Have you had any new children with this partner since we last spoke?	☐ Yes (1)<br>☐ No (0)
c18a	To your knowledge, did your partner start ART?	☐ Yes (1)<br>☐ No, they do not plan to link (2)<br>☐ No, but they plan to link (3)

For peer review only

		☐ Unsure (88)
**Unintended Consequences**		
c19a	Did you pressure your partner to initiate ART?	☐ Yes (1)<br>☐ No (0)
c20a	After enrolling in the study, did anyone find out your HIV status against your will (unwanted disclosure)?	☐ Yes (1)<br>☐ No (0)
C20b	After enrolling in the study, did anyone find out your partners' HIV status against his will (unwanted disclosure)?	☐ Yes (1)<br>☐ No (0)
c21a	After enrolling in the study, did your partner….<br><br>Threaten to hurt or harm you or someone you cared about.	☐ Yes (1)<br>☐ No (0)<br>☐ Refused to respond (99)
c22a	Insulted you or made you feel bad about yourself.	☐ Yes (1)<br>☐ No (0)<br>☐ Refused to respond (99)
c23a	Hit, slapped, kicked or did anything else meant to physically hurt you.	☐ Yes (1)<br>☐ No (0)<br>☐ Refused to respond (99)
C23b	Forced sexual intercourse and other forms of sexual coercion	☐ Yes (1)<br>☐ No (0)<br>☐ Refused to respond (99)

For peer review only

C23c	Slept with another woman.	☐ Yes (1)<br>☐ No (0)<br>☐ Refused to respond (99)
c24a	Ended the relationship	☐ Yes (1)<br>☐ No (0)<br>☐ Refused to respond (99)
c25a	*Now, I am going to ask you a series of questions about who makes within this relationship*<br><br>Who usually decides how the money you earn will be used?	☐ Yourself (respondent)<br>☐ Jointly (This partner and you together)<br>☐ Mainly this partner<br>☐ Someone else<br>☐ Do not earn money<br>☐ Refuse to say
c26a	Who usually decides how your partner's earnings will be used?	☐ Yourself (respondent)<br>☐ Jointly (This partner and you together)<br>☐ Mainly this partner<br>☐ Someone else<br>☐ Refuse to say
c27a	Who usually makes decisions about health care for yourself?	☐ Yourself (respondent)<br>☐ Jointly (This partner and you together)<br>☐ Mainly this partner<br>☐ Someone else<br>☐ Refuse to say

For peer review only

	Additional Questions	
d1	Would you recommend the ART intervention to other male friends or family?	☐ Yes (1)<br>☐ No (0)
d2	Are you happy your partner was in the ART intervention?	☐ Yes (1)<br>☐ No (0)

Thank the participant for their time and end the survey

For peer review only

FOLLOW-UP SURVEY
Identifying efficient linkages strategies for HIVST (IDEaL)
Female (Chichewa)

Complete this form for women whose:

(1) Partners consented to be in the study 4-months ago

Date of Interview: ______________________
Site Code: ____________________________

Full Name of Interviewer: _________________________________

Participant Study ID#: ____________

#	Question	Response
c1a	Taganizani za bwenzi lanu logonana nalo lomwe lakhala lodalilika kwa miyesi folo yapitayi.<br><br>Kodi ubwenzi wanu wasintha munjira ina iliyonse mu miyezi folo yapitayi?<br><br>Bwenzi lokhazikika	☐ Palibe chasintha (1)<br>☐ Married (2)<br>☐ Bwenzi lokhazikika (3)<br>☐ Chibwenzi Chinachoka pakhomo (4)<br>☐ Chibwenzi cha apo ndi apo (5)<br>☐ Separated (6)<br>☐ Banja linatha (7)<br>☐ Other (8)
c4a	Kodi munawauza abwenzi anuwa za mmene mulili mnthupi mwanu ku mbali ya HIV?	☐ Eya(1)<br>☐ Ayi(0)
c5a	Have you had any new children with this partner since we last spoke?<br><br>Kodi mwakhala ndi ana ndi abwenzi anuwa kuchokera ulendo watha tinayankhulana?	☐ Eya(1)<br>☐ Ayi(0)
c18a	Mongamukudziwira, kodi bwenzi lanu linayamba kumwa mwankhala a ama ARV?	☐ Eya<br>☐ Ayi, sakulingalira zoyamba kumwa mwankhwala a ma ARV<br>☐ Ayi, koma

		akulingalira zoyamba<br>☐ Unsure (88)
**Unintended Consequences**		
c19a	Kodi munawakakamiza a bwenzi anu kuti ayambe kumwa mankhwala a ama ARV?	☐ Eya(1)<br>☐ Ayi(0)
c20a	Chilowereni mu study, alipo omwe anadziwa za m'mene mulili mnthupi mwanu kumbari ya HIV inu musakufuna? (Kuwulula za HIV mosafuna)	☐ Eya(1)<br>☐ Ayi(0)
C20b	Chilowereni mu study, alipo omwe anadziwa za m'mene mulili mnthupi mwa bwenzi lanu kumbari ya HIV eni asakufuna? (Kuwulula za HIV mosafuna)	☐ Eya(1)<br>☐ Ayi(0)
c21a	Chilowereni mu study, kodi bwenzi lanu…..<br><br>Linaospyeza kuvulaza inu kapena wina aliyense amane mumamukonda?	☐ Eya(1)<br>☐ Ayi(0)<br>☐ Refused to respond (99)
c22a	Anakunyozani kapenanso kukupangitsani kuti muzizikayikila.	☐ Eya(1)<br>☐ Ayi(0)<br>☐ Refused to respond (99)
c23a	Anakumenyani ndikukupwetekani.	☐ Eya(1)<br>☐ Ayi(0)<br>☐ Refused to respond (99)
C23b	Kukukakamizani kugonana ndi zinthu zina?	☐ Eya(1)<br>☐ Ayi(0)<br>☐ Refused to respond (99)
C23c	Kuchita mtchitidwe ogonana ndi mzimayi wina	☐ Eya(1)<br>☐ Ayi(0)

For peer review only

		☐ Refused to respond (99)
c24a	Kuthetsa Chibwenzi	☐ Eya(1)<br>☐ Ayi(0)<br>☐ Refused to respond (99)
c25a	Tsopano ndikufunsani mafunso okhuzana ndi omwe amalamula pakhomo panu.<br><br>Kodi amalamula za mmene ndalama zomwe mwapeza zitagwilitsidwile ntchito ndi ndani?	☐ Amene akuyankha<br>☐ Mogwirizana<br>☐ Nthawi zambiri bwenzi<br>☐ Munthu wina<br>☐ Refuse to say
c26a	Kodi amalamulila za mmene ndalama za abwenzi anu zingagwilitsidwile ntchito ndi ndani?	☐ Amene akuyankha<br>☐ Mogwirizana<br>☐ Nthawi zambiri bwenzi<br>☐ Munthu wina<br>☐ Refuse to say
c27a	Kodi amene amakhala ndi ulamulilo pa chisamalilo cha moyo wanu ndi ndani?	☐ Amene akuyankha<br>☐ Mogwirizana<br>☐ Nthawi zambiri bwenzi<br>☐ Munthu wina<br>☐ Refuse to say

	**Additional Questions**	
d1	Kodi mungalimbikitse anzanu ena achizibambo kapena apabanja panu kutenga nawo mbali mu ART intervention munaliyi?	☐ Eya(1)<br>☐ Ayi(0)
d2	Kodi muli okondwa kuti munatenga nawo mbali mu ART intervention?	☐ Eya(1)<br>☐ Ayi(0)

For peer review only

14.9. APPENDIX I: Data Extraction Tool

DATA EXTRACTION TOOL
Identifying efficient linkages strategies for HIVST (IDEaL)
English only

INSTRUCTIONS:

The Medical Chart Review will be used to link the male study participant with the facilities ART records and to document their facility visits over the 4-months of study participation. Please follow the instructions to prepare for data collection (1) gather all ART registers that were active between DAY MONTH YEAR up to today (2) enter and re-enter the participant ID's who have reached the 4-month follow up period into the tablet (3) once you have re-entered, the tablet will provide you with identifying information about the male study participant. (5) match the participants information with information provided by the ART register to see if the participant initiated ART or not. If a participant did not initiate care (i.e. you cannot find him in the ART register), still enter the initial data points and indicate that the participant did not

Code	Question	Responses
pid	Please enter the Participant ID	
district	District	☐ Chickwawa<br>☐ Nkhotakota<br>☐ Lilongwe<br>☐ Kasungu
site	Facility name	☐ Chickwawa District Hospital<br>☐ St. Montford Mission Hospital<br>☐ Kalemba Community Hospital<br>☐ Kasungu District Hospital<br>☐ Nkhoma Community Hospital<br>☐ Mponela Rural Hospital<br>☐ Deayang Luke Hospital<br>☐ Nkhotakota District Hospital

For peer review only

		☐ Nsanje District Hospital<br>☐ Ngabu Rural Hospital
	We want to know if this participant initiated ART. Please look at the ART register used at the clinic between DATE MONTH YEAR up to today<br><br>*Instructions: Look for the below information in the ART register, matching the below participant with a name in the ART register.*<br><br>*Sometimes it is hard to find an exact match in the ART register. Consider it a match if 3 of the 4 data points match. For example, someone's name may be different, but the age, and village/residence matches. Consider this the same person.*<br><br>*CLIENT NAME:*<br>*AGE:*<br>*Ta:*<br>*Village:*	
found_art	Was the participant found in the ART register?	☐ Yes (1) – proceed to next question<br>☐ No (0) – end survey
art_number	What is the participant assigned ART number?	
art_date	What is the clients ART start date?	___ ___/___ ___/___ ___<br>Day Month Year

For peer review only - http://bmjopen.bmj.com/site/about/guidelines.xhtml

	Use the ART number to find the paper Mastercard OR look up the participant in the Baobab system	
mastercard_found	Did you find the mastercard/baobab record?	☐ Yes (1) – proceed to next question<br>☐ No (0) – end survey
tb	At initiation: TB	☐ Yes (1)<br>☐ No (0)
ks	At initiation: KS	☐ Yes (1)<br>☐ No (0)
pillcount	Number of pills given	
nextapp_date	Date of next appointment	___ ___/___ ___/___ ___<br>Day Month Year
nextconsult_date	Date of next consultation visit	___ ___/___ ___/___ ___<br>Day Month Year

END OF SURVEY

For peer review only

14.10. APPENDIX J: In-Depth Interview Guide – Female

IN-DEPTH INTERVIEW GUIDE
Identifying efficient linkages strategies for HIVST (IDEaL)
Female

BEGIN RECORDING

Original Study ID: ____________________

Repeat Original Study ID:

State if male or female respondent ____________________

Health Facility: ____________________

Date of Interview: ____________________

Full Name of Interviewer: ______________________________

District where respondent Lives: ______________________________

Open- Ended Questions

Note: The in-depth interview will be open-ended and guided by the respondent's answers. This outline reflects a general guide for the in-depth interviews.

The interviews are meant to help us understand barriers and facilitators to ART initiation. We are also interested in their thoughts on new interventions we are developing to help men start ART. The following questions are meant to guide interviewers. Actual questions asked during the interview will vary based on participant responses.

DEMOGRAPHICS:

Demographics on the female participant not collected. Already in the original study. Just make sure the Original Study ID is documented correctly.

1. What type of job/work does your partner do?
    a. If he does not work, why?
    b. Does he do anything else to earn money?
2. What time of day/week is your partner usually busy?
3. Where does he spend most of his time when he is not working?
    a. *Probe: At the bar, watching football, at church, at home…*
4. How long have you been in a relationship with your sexual partner (whom you gave the HIVST kit to)

HIV TESTING:

Now I'd like to talk to you about you and your partner's experience with HIV services

1) To your knowledge, has your partner tested for HIV before you gave him the HIV-self testing kit?
   a) IF YES, did he tell you the result? What was his result?
   b) IF HIV+: At that time, did he start ART? IF NO: Why not?
2) Think about when you gave the HIV self-test kit to your sexual partner (show the kit).
   a) How did your partner feel about his HIV positive test result? How did the result affect him?
   b) How did you feel after your partner received an HIV positive status on the HIV self-test kit? How did the result affect you?
   c) Did your relationship change at all after he received an HIV-positive status? How?
   d) Did he talk to anyone about it?/ who?
   e) Did he disclose to anyone besides you? Why/why not?

ART UPTAKE:

1) After he tested HIV-positive, did he initiate ART? Why/Why not?
   a) IF INITIATED ART: How long did it take him to initiate ART? (several weeks, several months?). Why did you initiate ART so quickly/slowly? /

We know starting ART is difficult.

1) What do you think is the most difficult thing about starting ART for your partner right now?
   a) Do you think this is the same for men and for women? How is it different?
   b) How does taking ART affect one's daily activities? (schedule/routine) In bad ways? In good ways? Is it different for men? How?
   c) How does taking ART affect relationships? In bad ways? In good ways? Is it different for men? How?
   d) Out of all these things you mentioned, what do you think is the biggest concern/problem for your partner?

<u>FOR MEN WHO ARE CONSIDERED LOSS-TO-FOLLOW UP</u>

1. Since we saw you last, we haven't been able to follow up with your partner. From your perspective, why do you think this is?
2. Since your partner was enrolled in our study, what kind of things did you notice or did he mention to you related to HIV health services?
   a. *Probe: phone calls/SMS or speak one-on-one to a health care worker? Was your partner visited by a health care professional within your home or in the community?*
3. Has your partner mentioned any of these interactions to anyone?
   a. *Probe: friends, family, community members*
4. How did your partner react to these things? Did he like/dislike them? What did he do when they happened?
   a. *Probe: phone call, SMS, in-person visit, home-based ART*
5. How did you react to these things? Did you like/dislike them?
   a. *Probe: Was there any reaction amongst your family, friends or the community to these things?*
   b. *If so, how did this affect your partner and/or you?*

SUGGESTIONS FOR ART SERVICES

For peer review only

Thank you for all the information. We would like to develop ART services that meet the needs of individuals in your community. We understand that men may face different challenges than women. I would like to know your opinion about what is needed in order to make ART services easy to access and use.

1) Could you describe the ideal way ART services would be given to your partner? If the clinic could do anything …
   a) When would he want to pick up ART?
      i) Probe: day of week, time of day?
      ii) *Why do you say this?*

   b) Where would he want to pick up ART*?*
      i) Probe: clinic near you, clinic far from you, somewhere in the community (WHERE SPECIFICALLY), at your home?
      ii) *Why do you say this?*

2) Do you think your partner needs more HIV-related information? About the benefits of ART, how to keep his status a secret, or how to disclose his status, about other aspects of his health?
   a) IF YES: How would he want to get this information? In person (one on one, pamphlet, radio, phone call, …)
      i) Probe: IF YES: WHO would he like to talk to about this information? (Provider, expert client, other community member)

3) Does he need support? For example, reminders to go to the health facility, someone to talk to regularly about what he is going through with ART, help disclosing his status, anything else).
   a) Probe: IF YES: How would he want to get this support? In person (one on one, pamphlet, radio, phone call)
   b) Probe: IF YES: WHO would he like to talk to about this support? (Provider, expert client, other community member)

4) Out of time of pick up, location, more information, or more support, what are the most important factors for your partner to use ART services?
   a) *PROBE: Think about other males. What do you think they would say is the most important factor for men to use ART services?*

SPECIFIC SUGGESTIONS ON CURRENT INTERVENTIONS

<u>FOR MEN WHO ARE CONSIDERED LOSS-TO-FOLLOW UP</u>

1. As discussed earlier, your partner received [phone calls, texts, in person counseling, home-based ART].
   a. *Do you feel that this was enough to encourage him to start ART?*
      i. *If NO, what would you have done differently? (frequency, content, location)*
      ii. *If YES, why do you think they were sufficient?*
   b. Are there any other ideas/services we should think about doing beside the ones we just talked about (appointment reminders, in-depth counseling, community/home ART)?/
      i. PROBE: What is it?
      ii. Why do you think this could work?

For peer review only

CONCLUSION

Thank you for your time.

1. Is there anything else you would like to say about men's use of ART?
2. Is there anything else you would like to say about your own use of ART and how we can help make your experience better?

STOP THE RECORDER AND MAKE SURE RECORDING IS SAVED.

THANK YOU FOR YOUR PARTICIPATION IN THIS INTERVIEW. LET ME ENCOURAGE YOU THAT ARVS CAN HELP YOU LIVE LONG AND HEALTHY.

The following general education messages should be conveyed to all male and female participants:

- All people who have been tested HIV positive should start ART as soon as possible for their own health and to prevent passing the virus on to others.
- Serious diseases can occur even in patients with high CD4 count (>500), without any previous symptoms. Immediate ART greatly reduces this risk.
- People that start ART and continue lifelong without interruptions can remain healthy and live as long as people without HIV.
- Even though you may not feel sick, ART is still important to keep you healthy for the rest of your life.
- ART reduces the amount of virus in your body and therefore can reduce the chance that HIV is passed to your sex partners.
- Current ART regimens are easy to take and rarely cause serious side-effects. Some people have side effects in the first few weeks of treatment and these almost always go away. IF there are persistent side effects, an alternative HIV regimen can be given.

NOTE: Be careful not to give any specific medical advice but rather refer respondents back to the clinic to speak to a provider.

IN-DEPTH INTERVIEW GUIDE
Identifying efficient linkages strategies for HIVST (IDEaL)
Female (Chichewa)

BEGIN RECORDING

Original Study ID: ______________________

Repeat Original Study ID:

State if male or female respondent ______________________

Health Facility: ______________________

Date of Interview: ______________________

Full Name of Interviewer: ________________________________

District where respondent Lives: ________________________________

Demographics

1) Kodi bwenzi lanu limagwira ntchito yanji kuti apeze ndalama?
   a) Ngati sagwira ntchito ndi chifukwa chani?
   b) Kodi amapanga zinthu zina zothandizira kupeza ndalama?
2) Kodi ntha nd nthawi iti ya tsiku/sabata lomwe bwenzi lanu limakhala lotanganidwa?
3) Kodi nthawi yawo yambiri amakhala ali kuti ngati sakugwira ntchito?
4) Kodi mwakhala pa ubwenzi kwa nthawi yayitali bwanji ndi bwenzi lanu logonana nalo (lomwe munalipatsa ka chida koziyezela wekha HIV)

HIV-Testing

Pano ndimafuna ndikufunseni mafunso okhudzana ndi zomwe mwakumanapo nazo zokhudza thandizo la HIV.

1. Momwe mukudziwira kodi bwenzi lanu linayamba layezetsapo HIV musanawapatse kachida koziyeza wekha HIV?
   a. Ngati eya, anakuwuzani zotsatira? Zotsatira zawo zinali zotani?
   b. Ngati anapezeka ndi HIV nthawi imeneyo, anayamba kumwa ma ARV? Ngati ayi chifukwa chani?
2. Ganizirani nthawi yomwe mudapereka kachipangizo kodziyezera wekha HIV (awonetseni) kwa bwenzi lanu.
   a. Kodi bwenzi lanu linanva bwanji atadziwa za zotsatira zawo za HIV? Zotsatirazo anazilandira bwanji?
   b. Kodi munamva bwanji bwenzi lanu litalandira zotsatira zoti lili ndi kachilombo ka HIV

pa kachipangizoka? Izi zinakukhudzani bwanji?

c. Kodi ubwenzi wanu udasintha atalandira zotsatira zoti ali ndi kachilombo? Motani?
d. Kodi analankhulapo ndi wina aliyense? Mosatchula dzina, ndani?
e. Kodi anawuzapo wina aliyense za mthupi mwake kupatula inu? Chifukwa chani?

ART Uptake

1) Atadziyeza ndi kupeza kuti ali ndi kachilombo, kodi adakayamba kumwa mankhwala? Chifukwa?
   a) Kodi zinatenga nthawi yaitali bwanji asanayambe kumwa mankhwala a ma ARV? (masabata angapo, miyezi ingapo?) Kodi anayamba kumwa mankhwala mnsanga/mochedwa chifukwa chani?
2) Tikudziwa kuti kuyamba kumwa mankhwala a ARV kumavutirapo. Kodi mukuona kuti chovuta chachikulu pa kuyamba kumwa mankhwala ndi chani?
   a) Kodi mukuganiza kuti izi ndi zofanana kwa amuna ndi akazi? Nzosiyana chifukwa chani?
   b) Kodi kumwa mankhwala a ma ARV kumakhudza bwanji kagwiridwe ka ntchito ka munthu tsiku ndi tsiku (ntchito zomwe amagwiragwira) Mu njira yabwino kapena yobwera mmbuyo? Ndi zosiyana kwa amuna? Motani?
   c) Kodi kumwa mankhwala a ma ARV kumakhudza bwanji mwaubwenzi? Mu njira yabwino kapena yobwera mmbuyo? Ndi zosiyana kwa amuna? Motani?
   d) Pa zinthu zonse zomwe tambambikalana, mukuona kuti vuto lalikuru kwa bwenzi lanu ndi chani?

<u>*FOR MEN WHO ARE CONSIDERED LOSS-TO-FOLLOW UP*</u>

1) Chiwonelaneni ulendo watha, talephela kuonananso ndi bwenzi lanu. Mumaganizo anu mukuona kuti zili motere chifukwa chani?
2) *Chilowereni cha bwenzi lanu mu study, mwaonako zinthu zotani kapena zomwe atchulako zokhudzana ndi thandizo la HIV?*
   a) *Funsitsani: Lamya ya m'manja/uthenga wa pa lamya kapena kuyankhula ndi wazaumoyo? Kodi bwenzi lanu linayendeledwapo ndi wazaumoyo mu dela lanu kapena pafupi ndi nyumba yanu?*
3) Kodi bwenzi lanu lathculako mikumano iyi kwa wina aliyense?
   a) *Funsitsani: Anzawo,achibale, anthu a mudela*
4) Kodi bwenzi lanu linapangapo chani pa zinthu izi? Anazikonda/sana zikonde? Anapanga chani zitachitika?
   a) *Funsitsani: Lamya ya m'manja/uthenga wa pa lamya, kupita okha , thandizo la ARV lapakhomo*
5) Munanva bwanji kuzinthuzi? Munazikonda/simunazikonde?
   a) *Achibale anapanga chilichonse, anzanu,anthu a mudela ku zinthu zimenezi?*
   b) *Ngati zili choncho, izi zinawatani a bwenzi anu /inuyo*

SUGGESTIONS FOR ART SERVICES

Zikomo chifukwa cha mayankho anu. Timafuna titakhazikitsa ndondomeko zokomera anthu mu dera lanu pokhudza thandizo la ma ARV. Tikudziwa kuti amuna amakumana ndi zophinja/mavuto osiyana

For peer review only

ndi amayi. Ndimafuna ndimve maganizo anu pa zofunika kuti tikonze bwino thandizoli kuti likhale losavuta kufikira ndi kugwiritsa ntchito.

1) Momwe mukuganizira, kodi mungandiuzeko njira yabwino kwambiri yomwe mukuganiza kuti thandizo la ARV lingamaperekedwere kwa bwenzi lanu? Chipatala chitati chichitepo kanthu….
   a) Kodi bwenzi lanu lingafune lidzipita nthawi zotani ku chipatala kukatenga mankhwala
      i) Tsiku la mu sabata, nthawi?
      ii) *Ndi chifukwa chani?*
   b) Kodi ndi malo ati omwe bwenzi lanu lingakonde kumakatengerako ARV?
      i) Pa chipatala cha pafupi nanu, kutali nanu, kwina mu dela lino (MALO ATI KWENIKWENI) pakhomo panu
      ii) *Ndi chifukwa chani?*
2) Kodi mukuganiza kwanu, mukuona kuti bwenzi lanu likufunika uthenga wina owonjezera? Pa zokhudza ubwino wa ARV, momwe lingasungire chinsinsi cha momwe mthupi mwawo mulili kapena kuwauza ena za mthupi mwawo, zina zikhuzana ndi thanzi lawo?
   a) Bwenzi lanu lingakonde litalandira mu njira yotani uthengawu? Kukawapeza paokha (paokha, kulembedwa mu bukhu, pa wailesi, kuimbiridwa foni)
   b) Ndi NDANI amene bwenzi lanu lingakonde kulankhula naye za uthengawu? (Dokotala, Expert Client, anthu ena a mu dera)
3) Kodi mukuganiza kuti bwenzi lanu likufunika thandizo lapadera? Mwachitsanzo, kukumbutsidwa kuti lipite ku chipatala, kulankhula ndi munthu wina mowirikiza pa zomwe akukumana nazo pakumwa ARV, kuthandizidwa kuuza ena za mmomwe mthupi mwawo mulili ndi zina)
   a) *Bwenzi lanu lingakonde litalandira mu njira yotani thandizoli? Kukawapeza pawokha (pawokha, kulembedwa mu bukhu, pa wailesi, kuimbiridwa foni)*
   b) *Ndi NDANI amene bwenzi lanu lingakonde kulankhula naye za thandizoli? (Dokotala, Expert Client, anthu ena a mu dera)*
4) Potengera thandizo ndi chisamaliro cha ma ARV, pakati pa nthawi yokatengera mankhwala, malo ake, uthenga owonjezera ndi thandizo la padera, kodi mukuganiza kuti chofunikira kwambiri ndi chiti kwa bwenzi lanu?
   a) *Ganizirani amuna ena a mdera lanu. Kodi mukuganiza kuti iwo angatchule chani ngati chinthu chofunikira kwambiri kwa amuna pa zomwe tachula zija?*

SPECIFIC SUGGESTIONS ON CURRENT INTERVENTIONS

<u>FOR MEN WHO ARE CONSIDERED LOSS-TO-FOLLOW UP</u>

1) Monga takambirana kale poyamba,bwenzi lanu linalandira (Kuyimbilidwa lanya, uthenga wa pa lamya, malangizo, thandizo la ma ARV la pakhomo)
   a) Mukuwona ngati izi zinali zokwanira kulimbikitsa bwezi lanu kuyamba ma ARV?
      i) Ngati ayi ndi chani mukanapanga mosiyana? (malo)
      ii) Ngati eya mukuwona ngati zili zofunika chifukwa chani?
2) Kodi pali maganizo ena omwe mukuona kuti angathandizepo kupatula omwe tatchulawa (kukumbutsana, uphungu wapadera, kugawa mankhwala kumudzi) ndi chani?

a) Ndi chani?
b) Ndi chifukwa chani mukuona kuti zikhoza kuyenda bwino

CONCLUSION

1) Zikomo chifukwa cha nthawi yanu. Pali choonjezera china chilichonse chomwe mungalankhulepo chokhudza kagwiritsidwe ntchito ka thandizo la ARV ndi amuna?
2) Pali chilichonse chokhudza kagwiritsidwe ntchito kanu ka thandizo la ma ARV ndi momwe tingakonzere thandizoli?

STOP THE RECORDER AND MAKE SURE RECORDING IS SAVED.

ZIKOMO CHIFUKWA CHOTENGA NAWO MBALI MU KAFUKUFUKUYU. NDIMAFUNA NDIKULIMBIKITSENI KUTI MA ARV AKHOZA KUTHANDIZA INU KUKHALA MOYO WAUTALI NDINSO WATHANZI.

The following general education messages should be conveyed to all male and female participants:

- Anthu onse omwe ayezetsa ndi kupezeka ndi kachilombo ka HIV akuyenera kuyamba kumwa mankhwala a ma ARV mwamsangamsanga kuti zipundulire thanzi lawo komanso apewe kupatsira ena kachilombo.
- Matenda oopsa akhoza kugwira munthu ngakhale amene chiwerengero cha asilikali a mnthupi ndi ochuluka (kuposera 500) posaonetsa zizindikiro zoyamba. Mankhwala a ma ARV amachepetsa chiopsezochi.
- Anthu omwe ayamba kumwa ma ARV ndipo akupitiliza moyo wawo onse osalekalekeza akhoza kukhala moyo wa thanzi ndi wautali chimodzimodzi anthu omwe alibe kachilombo ka HIV.
- Ngakhale simukumva kudwala, mankhwala a ARV ndi ofunikirabe kuti mukhale ndi thanzi moyo wanu onse.
- Makhwala a ma ARV amachepetsa mlingo wa tidzilombo mthupi mwanu kotero amachepetsa chiposezo chopatsira kachilombo kwa ena.
- Mankhwala omwe alipo pakadali pano a ma ARV ndiosavuta kumwa komanso sakhala ndi mavuto ambiri. Anthu ena amakhala ndi mavuto obwera kamba komwa mankhwala masabata oyambirira koma izi zimatha. Ngati zikupitilirabe pali mtundu wina wa mankhwala omwe akhoza kukupatsani.

14.11. APPENDIX K: In-Depth Interview Guide – Male

IN-DEPTH INTERVIEW GUIDE
Identifying efficient linkages strategies for HIVST (IDEaL)
Male

BEGIN RECORDING

Original Study ID: ____________________

Repeat Original Study ID:

State if male or female respondent ______________________

Health Facility: ______________________

Date of Interview: ______________________

Full Name of Interviewer: ________________________________

District where respondent Lives: ________________________________

DEMOGRAPHICS:

1. What is your current age in years?	Age in ☐☐ Completed Years
2. How would you rate your health today on a scale from 1-5 with 1 being excellent health and 5 being the very poor health?	☐ Excellent (1)<br>☐ Very good (2)<br>☐ Good (3)<br>☐ Fair (4)<br>☐ Poor (5)
3. Now I'd like to ask about your relationships. Are you currently in a sexual relationship?	☐ Yes (1)<br>☐ No (0)<br>**If NO, skip to QUALITATIVE**
4. Does your partner know <u>your</u> HIV status?	☐ Yes (1)<br>☐ No (0)

For peer review only - http://bmjopen.bmj.com/site/about/guidelines.xhtml

5. How many times have you tested for HIV?	
6. When was the first time you tested HIV-positive?	

Open- Ended Questions

Note: The in-depth interview will be open-ended and guided by the respondent's answers. This outline reflects a general guide for the in-depth interviews.

The interviews are meant to help us understand barriers and facilitators to ART initiation. We are also interested in their thoughts on new interventions we are developing to help men start ART. The following questions are meant to guide interviewers. Actual questions asked during the interview will vary based on participant responses.

SCRIPT: Now I'd like to talk to you about your experience with HIV services.

HIV TESTING

1. Think about when you used an HIV self-test kit (show the kit). When did you use it?
2. How did you feel after receiving the HIV positive status?
3. Can you talk to me about what happened after you tested HIV positive? Walk me through it so I can see the picture in detail?.
    a. Did you talk to anyone about it?
    b. Has your HIV status changed your daily activities at all? (Your schedule/routine)
    c. Has your HIV status impacted your relationships? How?
    d. Have you disclosed your status to anyone besides your partner? Why/why not

INTERVENTION

1. After you tested positive, we approached you to be a part of our study. Since you were enrolled in the study (ie in the last 3 months), can you walk me through what has happened related to HIV health services?
    a. Probe: What kinds of interactions have you had with expert clients or health personelle (phone call, SMS, in-person visits, home based ART).
    b. What was the frequency of these interactions (weekly, every other week, monthly)

ART INITIATION

2. Since you have been enrolled in the study, have you initiated ART?

INITIATED

3. How long did it take you to initiate ART? (several weeks, several months?). Where did you initiate?
    a. Why did you initiate ART so quickly/slowly?
4. Since you initiated treatment, have you continued to take you medication?

For peer review only - http://bmjopen.bmj.com/site/about/guidelines.xhtml

a. Have you returned to the clinic for another refill of ART? Have there been challenges to staying on treatment – how have you overcome them?

NOT INITIATED

1) Since you tested HIV-positive, have you been to a health facility?
   a) When did you attend? (year)
   b) Why did you attend? (guardian vs client; HIV vs OPD)
   c) Why did you not initiate ART during this visit?

ART UPTAKE

2) We know starting ART is difficult. What do you think is most the most difficult thing about starting ART?
   a) PROBE: Think about your male friends. What do you think they would say is the most difficult part about starting ART for men in your village?

INITITATED ART

1) How has taking ART affected your daily activities at all? (your schedule/routine) In bad ways? In good ways?
2) How has taking ART affected your relationships? In bad ways? In good ways?
3) Does being on ART change if you are able to hide/keep your HIV status from other people/ If you are able to hide/keep your status other people, does that make it easier to be on ART? How?

NOT INITITATED ART

1) How do you think taking ART would affect your daily activities at all? (your schedule/routine) In bad ways? In good ways?
2) How do you think taking ART would affect your relationships? In bad ways? In good ways?
3) Would being able to hide/keep your HIV status from other people make it easier for you to be on ART? How?

4) What do you think is most the SECOND most difficult thing about starting ART?

   a) PROBE: Think about your male friends. What do you think they would say is the SECOND most difficult part about starting ART for men in your village?

SUGGESTIONS FOR ART SERVICES

Thank you for all the information. We would like to develop ART services that meet the needs of men in your community. We understand that men are busy and may face different challenges than women. I would like to know your opinion about what is needed in order to make ART services easy to use for men in your community

1) Could you describe the ideal way ART services would be given to you? If the clinic could do anything
   a) When would you want to pick up ART?
   b) Probe: day of week, time of day?
   c) Why do you say this?
2) Where would you want to pick up ART?
   a) Clinic near you, clinic far from you, somewhere in the community (WHERE SPECIFICALLY), at your home?
   b) Why do you say this?

For peer review only

I would like to learn about how you felt about each of the interactions we talked about earlier (remind participant of what they mentioned – ex: phone calles, texts, in person visits, home based ART)

1) What did you like about them?
   a) Why? (ex: individual follow-up, sense of support, not having to travel to the clinic for homebased ART)
2) What did you dislike about them? What were challenges?
   a) Why? (ex: difficulty maintaining privacy with contact or visits, doesn't want to start ART for other reasons)
3) Do you feel like these things helped encourage you to seek health services?
   a) If YES, why?
   b) If NO, why?
4) Do you think these things would help other men in your community if they were to test positive for HIV?

<u>INITIATED</u>

1) Do you feel that these things helped to encourage you to initiate and stay on ART?
2) Do you think you would have started treatment without them?

<u>NOT INITIATED</u>

1) Why do you think these things failed to help you start/stay on treatment?
   a) Why?
2) If you could change anything about these interactions that you have listed, what would you change?
   a) Probe: Type of contact, frequency of contact, personelle, location, topics covered

3) We understand that everyone is different. Beyond what you have experienced, do you still have problems related to seeking health services for HIV? (i.e. are there still things that you need?)
   a) What are these unmet needs?
   b) What do you feel would be the best solution to meet those needs?
4) Are there any other ideas/services we should think about doing beside the ones we just talked about (appointment reminders, in-depth counseling, community/home ART)?
   a) What is it?
   b) Why do you think this could work?
5) Is there anything else that you would like to add as we are towards the end of the interview?

STOP THE RECORDER AND MAKE SURE RECORDING IS SAVED.

THANK YOU FOR YOUR PARTICIPATION IN THIS INTERVIEW. LET ME ENCOURAGE YOU THAT ARVS CAN HELP YOU LIVE LONG AND HEALTHY.

The following general education messages should be conveyed to all male and female participants:

- All people who have been tested HIV positive should start ART as soon as possible for their own health and to prevent passing the virus on to others.

For peer review only

- Serious diseases can occur even in patients with high CD4 count (>500), without any previous symptoms. Immediate ART greatly reduces this risk.
- People that start ART and continue lifelong without interruptions can remain healthy and live as long as people without HIV.
- Even though you may not feel sick, ART is still important to keep you healthy for the rest of your life.
- ART reduces the amount of virus in your body and therefore can reduce the chance that HIV is passed to your sex partners.
- Current ART regimens are easy to take and rarely cause serious side-effects. Some people have side effects in the first few weeks of treatment and these almost always go away. IF there are persistent side effects, an alternative HIV regimen can be given.

Be careful not to give any specific medical advice but rather refer respondents back to the clinic to speak to a provider.

For peer review only

IN-DEPTH INTERVIEW GUIDE
Identifying efficient linkages strategies for HIVST (IDEaL)
Male (Chichewa)

BEGIN RECORDING

Original Study ID: ______________________

Repeat Original Study ID:

State if male or female respondent ______________________

Health Facility: ______________________

Date of Interview: ______________________

Full Name of Interviewer: __________________________________

District where respondent Lives: __________________________________

1. Kodi pakadali pano muli ndi zaka zingati zakubadwa?	
2. Kodi thanzi lanu mungati lilibwanji lero pa mlingo wa 1 mpaka 5, pamene 1 akuyimira thanzi labwino kwambiri ndi 5 akuyimira kuti thanzi lanu silili bwino konse?	☐ Lili bwino kwambiri (1)<br>☐ Lilibwino (2)<br>☐ Pakati mpakati (3)<br>☐ Silili bwino (4)<br>☐ Silili bwino ngakhale pang'ono (5)
3. Pano ndimafuna ndikufunseni za maubwenzi. Kodi pakadali pano muli pa ubwenzi ogonana?	☐ Eya (1)<br>☐ Ayi (0)<br>**If NO, skip to QUALITATIVE**
4. Kodi bwenzi lanu limadziwa kuti mthupi mwanu muli bwanji kumbali ya HIV?	

For peer review only

5. Mwayezetsa kokwana kangati HIV?	
6. Kodi ulendo oyamba omwe mudayezetsa ndi kukupezani ndi kachilombo ka HIV ndi liti? (chaka)	

Pano ndimafuna ndikufunseni mafunso okhudzana ndi zomwe mwakumana nazo zokhudza ndi thandizo la HIV.

HIV TESTING

1) Ganizirani nthawi yomwe mudagwiritsa ntchito kachipangizo kodziyezera wekha HIV (awonetseni), kodi liti mudagwiritsa kachipangizoka?
2) Kodi munamva bwanji mutaona kuti zikuonetsa kuti muli ndi kachilombo ka HIV?
3) Mungandifotokozele zomwe zinachitika mutayezetsa ndikupezeka ndi HIV? Mugafotokoze mwatsatanetsatane kuti ndione chithunzithunzi cha momwe zinalili?
   a) Munalankhula ndi aliyense?
   b) Kodi kudziwa kuti muli ndi kachilombo ka HIV kunasintha ntchito zanu za tsiku ndi tsiku?
   c) Kodi kudziwa kuti muli ndi kachilombo ka HIV kunakhudza maubwenzi anu? Motani?
   d) Kodi munayamba mwauzapo za momwe mthupi mwanu mulili kupatula kwa okondedwa anu? Chifukwa chani?

INTERVENTION

1) Chifukwa choti munapezeka ndi HIV, tinakupezani kuti mutenge nawo mbali mu study yathu. Poti munatenga nawo mbali mu study (miyezi itatu yapitayi),mungandifotokozere zomwe zakhala zikuchitika zokhudza ndi thandizo la HIV?
   a) Mwakhala ndi mikumano yotani ndi omwe ali kale pa ma ARV kapena a zaumoyo(pa lamya,uthenga wa lamya yam'manja, kuyankhulana pamaso, kupita nokha kukakumana nawo,kulandira thandizo la ma ARV pakhomo).
   b) Kodi mikumanoyi imachitika pafupipafupi bwanji?

ART INITATION

1) Chiloweleni mu study, mwayamba kulandira ma ARV?

<u>*OYAMBA MANKHWALA*</u>

2) Kodi zinatenga nthawi yaitali bwanji musanayambe kumwa mankhwala a ARV? (masabata angapo, miyezi ingapo?) Kodi mukanayambira kuti? Kodi munayamba kumwa mankhwala mnsanga/mochedwa chifukwa chani?
3) Chiyambileni kulandira thandizo la ARV, kodi mukupitiliza kumwa mankwala anu?
   a) Mwapitako ulend wina kuchipatala kukalandira mankwala ena a ama ARV? Mwakhala mukukumana ndi zovuta zina chifukwa chokumwa mankhwala ama ARV, kodi mavutowa mwawathetsa bwanji?

For peer review only

OSAYAMBA MANKHWALA

4) Mutayezetsa nkupezeka ndi kachilombo ka HIV, munayamba mwapitapo ku chipatala?
   a) Munapita liti? (chaka)
   b) Munapita chifukwa chani? (kuperekeza odwala wina kapena munadwala?; munapitila HIV kapena ku OPD)
   c) Kodi simunayambe kumwa mankhwala a ma ARV nthawi imeneyi chifukwa chani?

ART UPTAKTE

1) Tikudziwa kuti kuyamba kumwa mankhwala a ARV kumavutirapo. Kodi mukuona kuti chovuta chachikulu pa kuyamba kumwa mankhwala ndi chani?
   a) Ganizirani anzanu aamuna mmudzi mwanu. Kodi mukuona kuti iwo angatchule chani ngati chovuta chachikulu pa kuyamba kumwa mankhwala a ART?

OYAMBA MANKHWALA

2) Kodi kumwa mankhwala kwakhudza bwanji ntchito zanu za tsiku ndi tsiku? Mu njira yabwino kapena yobwerera mmbuyo?
3) Kodi kumwa mankhwala kwakhudza bwanji maubwenzi anu? Mu njira yabwino kapena yobwerera mmbuyo?
4) Kodi kumwa mankhwala a ARV kumaphweka mukabisa momwe mthupi mulili kwa anthu ena? Motani?

OSAYAMBA MANKHWALA

5) Kodi mukuganiza kuti kumwa ma ARV kungakhudze bwanji ntchito zanu za tsiku ndi tsiku? Mu njira yabwino kapena yobwerera mmbuyo?
6) Kodi mukuganiza kuti kumwa mankhwala a ma ARV kungakhudze bwanji maubwenzi anu? Mu njira yabwino kapena yobwerera mmbuyo?
7) Kodi kumwa mankhwala a ARV kungaphweke/kumaphweka kuti mukabisa momwe mthupi mulili kwa anthu ena? Motani?

8) Kodi chinthu chachiwiri chovuta kwambiri pa kuyamba kumwa mankhwala ndi chani?
   a) Ganizirani amuna ena a mmudzi mwanu. Kodi mukuganiza kuti anganene kuti chovuta CHACHIWIRI pa kuyamba kumwa a ma ARV ndi chani mmudzi mwanu?

SUGGESTIONS FOR ART SERVICES

Zikomo chifukwa cha mayankho anu. Timafuna titakhazikitsa ndondomeko zokomera amuna mu dera lanu zokhudza thandizo la ma ARV. Tikumvetsetsa kuti amuna amakhala otangwanidwa ndipo amakumana ndi mavuto osiyana ndi amayi. Ndimafuna ndimve maganizo anu pa zofunika kuti tikonze bwino thandizoli maka kwa amuna mu dera lino.

1) Kodi mungandiuzeko njira yabwino kwambiri yomwe mukuganiza kuti thandizo la ma ARV lingamaperekedwere kwa inu, chipatala chitati chichitepo kanthu….
   a) Kodi mungafune mudzipita nthawi zotani ku chipatala kukatenga mankhwala?
      i) Tsiku la mu sabata, nthawi?
      ii) Ndi chifukwa chani?
   b) Kodi ndi malo ati omwe mungakonde kumakatengerako ARV?
      i) Pa chipatala cha pafupi nanu, kutali nanu, kwina mu dela lino (MALO ATI KWENIKWENI) pakhomo panu
      ii) Ndi chifukwa chani?
2) Ndimafuna ndinve za momwe mukunvera za kucheza konse tinali nako poyamba(Kumbutsani otenga nawo mbali zomwe anatchula-kuyimbilidwa lamya, uthenga wa pa lamya, kupitako okha, thandizo la pakhomo la ARV)
   a) Chomwe munakondapo chinali chani?
      i) Chifukwa?(Kuyendeledwa, kunva kuthandizidwa, osafunika kupita ku chipatala kukalandira ma AR olandilira pakhomo)
   b) Chomwe simunaonde ndi chani? Zovuta zinali chani?
      i) Chifukwa? (chitsanzo: kuvutika kusunga chinsinsi ndi owadziwa kapena mikumano,simukufuna kuyamba kumwa ma ARV pazifukwa zina)
   c) Mukuganiza kuti zinthu zimennezi zakulimbikitsani kupeza thandizo la zaumoyo?
      i) Ngati eya , chifukwa?
      ii) Ngati ayi, chifukwa chani?
   d) Kodi mukuona ngati zinthu zimenezi zingathandize anthu ena mu dela lanu ngati angakhale ndi HIV?

OYAMBA MANKHWALA

1) Kodi mukuona ngati zinthu zimenezi zinakuthandizani kulimbikitsika kuti muyambe komanso kupitiliza kumwa mankhwala a ma ARV?
   a) Kodi mukuganiza kuti mukanatha kuyamba thandizo la ma ARV popanda zimenezi?

OSAYAMBA MANKHWALA

1) Kodi mukuganiza kuti zinthu zimenezi zinalephera kukuthandizani kuyamba/kukhala mankhwala a ma ARV?
   a) Chifukwa?
2) Ngati mungathe kusintha chilichonse cha mikumano yomwe mwatchula,chingakhale chani?
   a) Mtundu wa mkumano, muligo wa mkumano, muthu, malo,mitu yokambilana
3) Tikunvetsa kuti anthu ndife osiyana. Kuposa zomwe mwakumana nazo, pakadali pano mukukumanabe ndi mavuto okhudzana ndi kupeza thandizo la zaumoyo la HIV? (pali zinthu zina zomwe mumafunabe?)
   a) Ndi zinthu ziti?
   b) Mukuganiza kuti ndi njira yanji yabwino yothandiza kupeza zofunikazi?

4) Kodi pali maganizo ena omwe mukuona kuti angathandizepo kupatula omwe tachulawa (kukumbutsana, uphungu wapadera, kugawa mankhwala kumudzi) ndi chani? Mukuona kuti maganizo anuwa angatheke chifukwa chani?\
5) Apa tikufunakumaliza kucheza kwathu, pali china chiwinjezera chomwe mungalankhulepo pa zomwe takambirana?

STOP THE RECORDER AND MAKE SURE RECORDING IS SAVED.

ZIKOMO CHIFUKWA CHOTENGA NAWO MBALI MU KAFUKUFUKUYU. NDIMAFUNA NDIKULIMBIKITSENI KUTI MA ARV AKHOZA KUTHANDIZA INU KUKHALA MOYO WAUTALI NDINSO WATHANZI.

The following general education messages should be conveyed to all male and female participants:

- Anthu onse omwe ayezetsa ndi kupezeka ndi kachilombo ka HIV akuyenera kuyamba kumwa mankhwala a ma ARV mwamsangamsanga kuti zipundulire thanzi lawo komanso apewe kupatsira ena kachilombo.
- Matenda oopsa akhoza kugwira munthu ngakhale amene chiwerengero cha asilikali a mnthupi ndi ochuluka (kuposera 500) posaonetsa zizindikiro zoyamba. Mankhwala a ma ARV amachepetsa chiopsezochi.
- Anthu omwe ayamba kumwa ma ARV ndipo akupitiliza moyo wawo onse osalekalekeza akhoza kukhala moyo wa thanzi ndi wautali chimodzimodzi anthu omwe alibe kachilombo ka HIV.
- Ngakhale simukumva kudwala, mankhwala a ARV ndi ofunikirabe kuti mukhale ndi thanzi moyo wanu onse.
- Makhwala a ma ARV amachepetsa mlingo wa tidzilombo mthupi mwanu kotero amachepetsa chiposezo chopatsira kachilombo kwa ena.
- Mankhwala omwe alipo pakadali pano a ma ARV ndiosavuta kumwa komanso sakhala ndi mavuto ambiri. Anthu ena amakhala ndi mavuto obwera kamba komwa mankhwala masabata oyambirira koma izi zimatha. Ngati zikupitilirabe pali mtundu wina wa mankhwala omwe akhoza kukupatsani.

Be careful not to give any specific medical advice but rather refer respondents back to the clinic to speak to a provider.

14.12. APPENDIX L: Personel CV's

Kathryn L. Dovel

Department of Medicine, Division of Infectious Diseases
David Geffen School of Medicine at UCLA
10833 Le Conte Ave 37-121 CHS
Los Angeles, CA 90095

Email: kdovel@mednet.ucla.edu
Office: +1 (310) 825-7225
Fax: +1 (310) 825-3632

POSITIONS HELD

2017- Adjunct Assistant Professor, Division of Infectious Disease
Department of Medicine, David Geffen School of Medicine - UCLA

2017- Research Director - Partners in Hope
Lilongwe, Malawi

2016-17 Postdoctoral Fellow in Global HIV Prevention Research
David Geffen School of Medicine - UCLA, 2016

2012-15 International Programs Director - 31Bits International
Gulu, Uganda

EDUCATION

PhD Health and Behavioral Sciences - University of Colorado Denver, 2016
Outstanding CLAS Ph.D. Student for the University of Colorado Denver
Outstanding Dissertation Award for the University of Colorado Denver
Dissertation: "Shifting Focus from Individuals to Institutions: The Role of Gendered Health Institutions on Men's use of HIV Services"

MPH Community Health Sciences - UCLA, 2010
Certificate in Global Health

BA Sociology & Anthropology (dual major), minor in Biology - Vanguard University, 2007
Magna Cum Laude

HONORS AND AWARDS

2019 5th place in the 2019 Department of Medicine Research Day poster competition, Department of Medicine, University of California Los Angeles

2019 Invited panelist, IAS 2019, Sticky and durable linkage: Latest evidence and new strategies

2019 Panel Chair, IAS Pre-Conference 2019, Men and HIV: What we know and what we don't know

2019 Top 5 abstracts at CROI, 2019

For peer review only

2019 Invited participant, Technical Consultation on HIV Linkage, International AIDS Society

2019 Invited participant, Differentiated Service Delivery Think Tank, Gates Foundation

2018 Joep Lange Award (best abstract at INTEREST, 2018)

2018 Female Global Scholar, The Women in Global Health Research Initiative (Weill Cornell Medicine, Cornell University)

2016 Outstanding Dissertation Award (UCDenver)

2016 Outstanding CLAS Ph.D. Student (UCDenver)

2012-16 Deans Travel Grant (UCDenver)

2007 Delta Kappa Honor Society (Vanguard University)

2007 Alpha Kappa Delta Honor Society (Vanguard University)

2007 Lambda Alpha Honor Society (Vanguard University)

2007 Anthropology Student of the Year (Vanguard University)

EXTERNAL GRANTS

2019-2023 Principle Investigator, Bill and Melinda Gates Foundation. (001423) "Identifying Effective Linkage Strategies for HIVST (IDEaL)"

2019-2024 Principle Investigator, Fogarty International Center. International Research Scientist Development Award (K01), K01TW011484. "Innovative strategies to increase ART Initiation and viral suppression among HIV+ men in Malawi".

2019-2020 Principle Investigator, Clinton Health Access Foundation. "The impact of facility HIV self-test scale up in Malawi: a mixed methods study"

2018-2021 Co-Investigator, The Conrad N. Hilton Foundation. Delivery of childhood development services as part of HIV treatment services in Malawi. Project implemented by UCLA and Partners in Hope.

2017-19 Principal Investigator, USAID. **Use of HIV self-test kits to increase identification of HIV-infected individuals and their partners: a Cluster Randomized Control Trial.** (sub-study within a large PEPFAR-USAID grant; PI: Risa Hoffman).

2017-19 Principle Investigator, Clinical Research Scholar, National Institutes of Health Loan Repayment Program.

2016-18 Co-Principle Investigaor, USAID. Test and Start: Tracking Uptake and Retention in Care using Standard Registry Data. (sub-study within a large PEPFAR-USAID grant; PI: Risa Hoffman).

2015 Principal Investigator, 31Bits International. "Evaluating the impact of a couple's livelihoods program on power dynamics and economic attainment among couples in northern Uganda." Project implemented by 31Bits International.

2014-16 Principal Investigator, NIMH National Research Service Award Predoctoral Individual Fellowship, F31-MH103078-01A1, "Gender Disparities in High-Risk PITC: The Role of Policy on Provider Practices", Impact Score: 14; Percentile: 2.0

2013-16 Principal Investigator, Stop AIDS Now!. "Evaluation of the 'Quality HIV and reproductive maternal and neonatal health services for women and young women in Africa through good

For peer review only

clinical governance and community-driven accountability'". Project implemented by the Clinton Health Access Initiative.

INTERNAL GRANTS

2016-18 Principal Investigator, UCLA Center for AIDS Research Seed Grant, University of California Los Angeles, "The Gendered Dynamics of ART Uptake and Retention under Universal Treatment Policies. Examining trends and ART barriers in Central Malawi"

2014 Principal Investigator, Calvin L Wilson Scholarship, University of Colorado Denver, "Gender and the provision of HIV testing: Examining how models of care influence men's use of testing services in southern Malawi"

2013-15 Principal Investigator, Dissertation Grant, University of Colorado Denver, "Gender Disparities in High-Risk PITC: The Role of Policy on Provider Practices"

2013-14 Principal Investigator, Robinson Durst Scholarship, University of Colorado Denver, "Gender disparities in high-risk PITC: Exploring the influence of feminized policy on provider practices in Malawi"

2009 Principal Investigator, Drabkin and Bixby International Scholarship, UCLA, "Evaluating barriers and facilitators of a nutrition program in the Bateyes of Dominican Republic"

2009 Principal Investigator, Global Health Grant, UCLA, "Evaluating a Nutrition Program in the Bateyes of Dominican Republic"

PUBLICATIONS

* represents MPH, PhD or medical students I mentored

2020 Cornell, Morna, Katherine Horton, Christopher Colvin, Andrew Medina-Marino, **Kathryn Dovel.** Raising the profile of men's health: the role of the research community: Letter to the editor. *Lancet*. Ahead of Print.

2020 **Dovel, Kathryn**, Mike Nyirenda, Frackson Shaba*, O. Agatha Offorjebe*, Kelvin Balakasi, Brooke Nichols, Khumbo Phiri*, Khumbo Ngona, Sundeep K Gupta, Risa Hoffman. "Facility-based HIV self-testing for outpatients dramatically increases HIV testing in Malawi: a cluster randomized trial." *Lancet Global Health*. Ahead of print

2020 **Dovel, Kathryn,** Khumbo Phii, Misheck Mphande, Deborah Mindry, Esnart Sanudi, McDaphton Bellos, Risa Hoffman. Optimizing Test and Treat in Malawi: Health care worker perspectives on barriers and facilitators to ART initiation among healthy clients. Global Health Action. Ahead of Print.

2020 Hubbard, Julie, Khumbo Phiri*, Corrina Moucheraud, Kaitlyn McBride, Ashley Bardon, Kelvin Balakasi, Eric Lungu, **Kathryn Dovel**, Gift Kakwesa, Risa Hoffman. A qualitative assessment of provider and client experiences with three- and six-month dispensing of antiretroviral therapy in Malawi. Global Health: Science and Practice. Ahead of Print.

For peer review only

2020 Hoffman, Risa M, Kelvin Balakasi, Ashley Bardon, Zumbe Siwale, Julie Hubbard, Gift Kakwesa, Mwiza Haambokoma, Thoko Kalua, Pedro Pisa, Crispin Moyo, **Kathryn Dovel**, Thembi Xulu, Ian Sanne, Matt Fox, Sydney Rosen. Eligibility for differentiated models of HIV treatment service delivery: an estimate from Malawi and Zambia. *AIDS.* 1;34(3):475-9.

2019 McBride, Kaitlyn, Julie Parent, Kondwani Mmanga, Mackenzie Chivwala, Mike H. Nyirenda, Alan Schooley, James B. Mwambene, **Kathryn Dovel**, Eric Lungu, Kelvin Balakasi, Risa M. Hoffman, Corrina Moucheraud. "ART Adherence Among Malawian Youth Enrolled in Teen Clubs: A Retrospective Chart Review." *AIDS Behav.* (2019): 1-5.

2019 Frackson Shaba*, Ogechukwu Offorjebe*, Phiri Khumbo, Lungu Eric, Kalande Pericles, Nyirenda Mike, Hoffman M Risa, Gupta Sundeep, **Dovel Kathryn**. Perceived Acceptability of a Facility-Based HIV Self-Test Intervention in Outpatient Waiting Spaces Among Adult Outpatients in Malawi: A Formative Study. *JAIDS*. 1;81(3):e92-4.

2019 Magaço Amílcar, **Dovel Kathryn**, Cataldo Fabian, Nhassengo Pedroso, Nuera Lucas, Tique José, Saide Mohomed, Couto Aleny, Mbofana Francisco, Gudo E Eduardo, Cuco Rosa Marlene, Chicumbe Sérgio. "Good health as a barrier and facilitator to ART initiation: a qualitative study in the era of Test and Treat in Mozambique." *Cult Health Sex. 11:1-5.*

2018 Cornell M, **Dovel K**. Reaching key adolescent populations. *Cur Opinion HIV AIDS.* 1;13(3):274-80.

2018 Sara, Yeatman, Stephanie Chamberlin*, **Kathryn Dovel.** Women's (health) work: A population-based, cross-sectional study of gender differences in time spent seeking health care in Malawi. *PLoS ONE.* 13(12): e0209586

2018 Nhassengo, Pedroso Fabian Cataldo, Amílcar Magaço, Risa Hoffman, Lucas Nuera, José Tique, Mohomed Saide, Aleny Couto, Francisco Mbofana, Eduardo Gudo, Rosa Marlene Cuco, Sérgio Chicumbe, **Kathryn Dovel**. "Barriers and facilitators to the uptake of universal treatment in Mozambique: a qualitative study on patient and provider perceptions." *PLoS ONE.* 13(12): e0205919

2018 Hubbard, Julie, Gift Kakwesa, Mike Nyirenda, James Mwambeneb, Ashley Bardona, Kelvin Balakasi, **Kathryn Dovel**, Thokozani Kaluac, Risa Hoffman. Towards the third 90: improving viral load testing with a simple quality improvement program in health facilities in Malawi. *International Public Health.* Ahead of print.

2018 **Dovel, Kathryn,** Frackson Shaba*, Ogechukwu Offorjebe*, Kelvin Balakasi, Khumbo Phiri*, Brooke Nichols, Chi-Hong Tseng, Ashley Bardon, Khumbo Ngona, Risa Hoffman. "Evaluating the integration of HIV self-testing into low-resource health systems: study protocol for a cluster randomized trial from EQUIP Innovations" *Trials.* 19:498.

2018 Moucheraud, Corrina, Dennis Chasweka, Mike Nyirenda, Alan Schooley, **Kathryn Dovel**, Risa Hoffman. "A simple screening tool may help identify high-risk children for targeted HIV testing in Malawian inpatient wards." *JAIDS.* 79:352-7.

2018 Cornell Morna, **Dovel Kathryn**. "Reaching key adolescent populations." *Current opinion*

For peer review only

in HIV and AIDS. 13(3):274-80.

2016 **Dovel, Kathryn**, Sara Yeatman, Joep Vanoosterhout, Adrienne Chan, Alfred Matengeni, Megan Landes, Richard Bedell, and Sumeet Sodhi. "Trends in ART Initiation among Men and Non-Pregnant/Non-Breastfeeding Women before and after Option B+ in Southern Malawi." *PLoS ONE.* (12): e0165025.

2016 Poulin, Michelle, **Kathryn Dovel** and Susan Watkins. "Men with money and the 'vulnerable women' client category in an AIDS epidemic." *World Development*. 85; 16-30.

2016 **Dovel, Kathryn**, Susan Watkins, Sara Yeatman, and Michelle Poulin. "Prioritizing strategies to reduce AIDS-related mortality for men in sub-Saharan Africa: Author's reply." *AIDS*. 30(1); 158-9.

2015 **Dovel, Kathryn,** Sara Yeatman, Susan Watkins, and Michelle Poulin. "Men's heightened risk of AIDS-related death: the legacy of gendered HIV testing and treatment strategies." *AIDS.* 29; 1123–5.

2015 **Dovel, Kathryn** and Kallie Thomson. "Financial obligations and economic barriers to antiretroviral therapy experienced by HIV positive women participating in a job-creation program in northern Uganda." *Culture, Health, and Sexuality*. 18(6).

2015 Krueger, Patrick, **Kathryn Dovel** and Justin Denney. "Democracy and self-rated health across 67 countries: A multilevel analysis." *Social Science and Medicine.* 143; 137-44.

2013 Conroy, Amy, Sara Yeatman and **Kathryn Dovel**. "The social construction of HIV/AIDS during a time of evolving access to antiretroviral therapy in rural Malawi." *Culture, Health and Sexuality.* 15(8); 924-37.

2012 Yeatman, Sara, **Kathryn Dovel**, Amy Conroy and Hazel Namadingo. "The predictors of HIV treatment optimism and its relationship with sexual risk behavior among a population-based sample of young adults in southern Malawi." *AIDS Care.* 25(8);1018-25.

TECHNICAL MANUSCRIPTS

2019 Hopkins, John, Laura Pascoe, Dean Peacock and Kathryn Dovel. "Accelerating Men's HIV service delivery and uptake in Eastern and Southern Africa UNAIDS Literature Review, Eastern and Southern Africa Regional Focus." UNAIDS, Johannesburg, South Africa.

2018 Masina, Tobias, **Kathryn Dovel**, Reuben Mwenda on behalf of the Malawi Ministry of Health. "National Guidelines for HIV self-testing." Malawi Ministry of Health Lilongwe Malawi.

2017 Pascoe, Laura, Dean Peacock and **Kathryn Dovel**. "To Get to Zero, We Must Also Get to Men – UNAIDS Literature Review, Eastern and Southern Africa Regional Focus." UNAIDS, Geneva.

2016 Macharia, Faith, Job Akuno, Faith Wanji, Julius Nguku, **Kathryn Dovel**, Caroline Ngare, Fred Nyagah, and Daniel Mwisunji. "National Guidelines for Male Engagement in HIV Services." Kenya Ministry of Health. Nairobi, Kenya.

2016 **Kathryn Dovel**, James Mkandawire, Susan Watkins, Nancy Mulauzi and Sydney Rodney Lungu. "Evaluation of the Good Clinical Governance Project: improving HIV and reproductive health services in Lilongwe, Malawi." Stop AIDS Now!. Lilongwe, Malawi.

For peer review only - http://bmjopen.bmj.com/site/about/guidelines.xhtml

OTHER PUBLICATIONS

2019 Kathryn Dovel, Stephanie Chamberlin, Sara, Yeatman. Malawi's Health System Puts Women First. This Isn't Always a Good Thing. *The Conversation: Africa.* Published February 19, 2019. Found at https://theconversation.com/malawis-health-system-puts-women-first-this-isnt-always-a-good-thing-111277

2016 **Dovel, Kathryn**, Sara Yeatman, and Susan Watkins. **Dying from a treatable disease: HIV and the men we neglect. *Huffington Post.* Published February 23, 2016. Found at** http://www.huffingtonpost.com/the-conversation-africa/dying-from-a-treatable-di_b_9295620.html

WORK IN PREPARATION

Dovel, Kathryn. "The gendered organization of HIV services and men's poor use of testing in southern Malawi: consequences of hegemonic masculinity within health institutions." (Revise & Resubmit, JIAS)

Offorjebe , Ogechukwu*, Frackson Shaba*, Kelvin Balakasi, Mike Nyrienda, Risa Hoffman, **Kathryn Dovel**. "Partner-delivered HIV self-testing increases the perceived acceptability of index partner testing among HIV-positive clients in Malawi." (Revise & Resubmit, PLoS ONE)

Dovel, Kathryn, Kelvin Balakasi, Khumbo Phiri*, Frackson Shaba*, O. Agatha Offorjebe*, Sundeep K Gupta, Vincent Wong, Eric Lungu, Brooke Nichols, Mike Nyirenda, Ngona K, Anteneh Worku, Risa Hoffman. "A randomized trial on index HIV self-testing for sexual partners of ART clients in Malawi." (Under Review)

Nichols, Brooke; Offorjebe, O. Agatha; Cele, Refiloe; Shaba , Frackson ; Balakasi, Kelvin; Chivwara, Mackenzie; Hoffman, Risa; Long, Lawrence; Rosen, Sydney; **Dovel, Kathryn**. "Economic evaluation of facility-based HIV self-testing among adult outpatients in Malawi. " (Under Review)

Dovel, Kathryn, Gladies Orobmi, Melanie Beagly*, Kallie Thomson. "Including men without sidelining women: the feasibility of male involvement within resource-strained gender equality programs in sub Saharan Africa." (Under Review)

Dovel, Kathryn and Kallie Thomson. "Evaluating the impact of a couple's livelihoods program on power dynamics and economic attainment among couples in northern Uganda." (In preparation)

Dovel, Kathryn. "Men in global HIV policy: examining discourses of blame and vulnerability." (In preparation)

SELECT PEER-REVIEWED PRESENTATIONS

2020 Moucheraud, Corrina, Samuel W. Lewis, Misheck Mphande, Ben Allan Banda, Hitler Sigauke, Paul Kawale, Aubrey Dkangoma, **Kathryn Dovel**, Alemayehu Amberbir, Agnes Moses, Sundeep Gupta, Risa M. Hoffman. Cervical cancer knowledge and attitudes among HIV-positive men in Malawi." Paper accepted for poster presentation. Conference on Retroviruses and Opportunistic Infections (CROI). Boston, Massachusetts, USA

2019 **Dovel, Kathryn**, Kelvin Balakasi, Khumbo Phiri*, Frackson Shaba*, O. Agatha Offorjebe*, Sundeep K Gupta, Vincent Wong, Eric Lungu, Brooke Nichols, Mike Nyirenda, Ngona K, Anteneh Worku, Risa Hoffman. "Index HIV self-testing among male partners in Malawi:

predictors of self-testing within a randomized controlled trial". Paper accepted for poster presentation. International AIDS Society. Mexico City, Mexico

2019 **Dovel Kathryn**, Salem Ejigu, Pericles Kalande, Evelyn Udedi, Chipawiru Mbalanga, Lauri Bruns, Thomas Coates. "Beyond the Caregiver: Diffusion of early childhood development knowledge and practices within the social networks of HIV-positive mothers in Malawi". Paper accepted for poster discussion. International AIDS Society. Mexico City, Mexico

2019 **Dovel, Kathryn**, Kelvin Balakasi, Khumbo Phiri*, Frackson Shaba*, O. Agatha Offorjebe*, Sundeep K Gupta, Vincent Wong, Eric Lungu, Brooke Nichols, Mike Nyirenda, Ngona K, Anteneh Worku, Risa Hoffman. "A randomized trial on index HIV self-testing for sexual partners of ART clients in Malawi." Paper accepted for oral presentation. Conference on Retroviruses and Opportunistic Infections (CROI). Seattle, Washington, USA

2019 Ogechukwu Offorjebe, **Kathryn Dovel**, Frackson Shaba, Kelvin Balakasi, Risa Hoffman, Sydney Rosen, Brooke Nichols, for the EQUIP Health team. Cost-effectiveness and national impact of index HIV self-testing in Malawi. Paper accepted for poster presentation. Conference on Retroviruses and Opportunistic Infections (CROI). Seattle, Washington, USA

2018 **Dovel, Kathryn**, Mike Nyirenda, Frackson Shaba*, Ogechukwu Offorjebe*, Kelvin Balakasi, Brooke Nichols, Khumbo Phiri*, Khumbo Ngona, Alan Schooley, Risa Hoffman on behalf of EQUIP Innovation for Health. "Facility-based HIV self-testing for outpatients dramatically increases HIV testing in Malawi: a cluster randomized trial." Paper accepted for oral presentation. International AIDS Society. Amsterdam, Netherlands

2018 Shaba, Frackson*, Kelvin Balakasi, Ogechukwu Offorjebe*, Mike Nyirenda, Risa Hoffman, **Kathryn Dovel** on behalf of EQUIP Innovation for Health. "Facility-based HIV self-testing in Malawi: an assessment of characteristics and concerns among clients who opt-out of testing." Paper accepted for poster presentation. International AIDS Society. Amsterdam, Netherlands

2018 **Dovel, Kathryn**, Mike Nyirenda, Frackson Shaba, Ogechukwu Offorjebe*, Kelvin Balakasi, Brooke Nichols, Khumbo Phiri*, Khumbo Ngona, Alan Schooley, Risa Hoffman on behalf of EQUIP Innovation for Health. "Facility-based HIV self-testing for outpatients dramatically increases HIV testing in Malawi: a cluster randomized trial." Paper accepted for oral presentation. INTEREST. Kigali, Rwanda - awarded the Joep Lange INTEREST award

2018 Offorjebe , Ogechukwu*, Frackson Shaba, Kelvin Balakasi, Mike Nyrienda, Risa Hoffman, **Kathryn Dovel** on behalf of EQUIP Innovation for Health. "Partner-delivered HIV self-testing increases the perceived acceptability of index partner testing among HIV-positive clients in Malawi." Paper accepted for mini-oral presentation. INTEREST. Kigali, Rwanda

2018 Stephanie Chamberlin*, Misheck Mphande, Pericles Kalande, **Kathryn Dovel** on behalf of EQUIP Innovation for Health. "Barriers and facilitators to consistent engagement in HIV care under Test and Treat in Malawi." Paper accepted for poster presentation. INTEREST. Kigali, Rwanda

2017 **Dovel Kathryn,** Khumbo Phiri*, Alan Schooley, Misheck Mphande, Mackenzie Chivwara, Risa Hoffman. "Facility-level barriers to antiretroviral therapy experienced by men in Malawi." Paper accepted for poster presentation. International AIDS Society. Paris, France

2017 Misheck Mphande, Khumbo Phiri*, Mackenzie Chivwara, Mike Nyirenda, Alan Schooley, Rachel Thomas, Risa Hoffman, **Kathryn Dovel**. "Examining Malawi's Rollout of Universal Treatment: Policy Implementation and Provider Perceptions." Paper accepted for poster presentation. International AIDS Society. Paris, France

2016 **Dovel, Kathryn**. “Factors influencing the implementation of provider-initiated testing and counseling (PITC) among STI clients in southern Malawi: A mixed methods study.” Paper accepted for poster presentation. International AIDS Society. Durbin, South Africa

2016 Westerhof, Nienke, Dzowela M, **Kathryn Dovel**, E. Banda, J. Chikonda. “Community-driven accountability through advocacy committees: a vehicle for improving HIV and reproductive health services for women living with HIV.” Paper accepted for poster presentation. International AIDS Society. Durbin, South Africa

2016 **Dovel, Kathryn**, Patrick Krueger, Shari Dworkin. “Predictors of men’s use of HIV testing services in low-income countries: the role of masculinity.” Paper accepted for poster presentation. Population Association of America. Washington D.C.

2014 **Dovel, Kathryn**. "Gender in HIV Policy: Examining how gender shapes the dissemination of HIV policies in southern Malawi." Paper accepted for roundtable presentation. American Public Health Association. New Orleans.

2014 **Dovel, Kathryn**. “Gendered care: examining how clinic experiences influence HIV testing decisions among STI patients in southern Malawi.” Paper accepted for oral presentation. National Women’s Studies Association, San Juan, Puerto Rico.

2013 **Dovel, Kathryn**. “HIV policies and their influence on men's use of care.” Paper accepted for oral presentation. International HIV Social Science and Humanities Conference, Paris, France.

2007 **Dovel, Kathryn**. “Social and structural impediments that limit proper healthcare in rural southern Kurdistan.” Paper accepted for oral presentation. The Anthropology and Sociology Research Conference, Santa Clara, CA.

INVITED PRESENTATIONS

2019 “Index HIV Self-Testing in Malawi”. World Health Organization webinar

2019 “Men’s (lack of) access to the health system”. UNAIDS. Regional meeting on Accelerating Men’s HIV service delivery and uptake in Eastern and Southern Africa.

2019 “Index HIVST in Malawi: a Randomized Control Trial. World Health Organization. Webinar

2019 “*Reaching men and engaging them in HIV care – lessons from Malawi*”. Men and HIV forum. International AIDS Society. Mexico City, Mexico

2018 “The impact of HIV self-testing on HIV testing among outpatients in high burden facilities in Malawi: preliminary findings from a cluster randomized control trial” USAID Washington. Washington D.C

2017 “Who benefits from Test and Treat? Understanding gender dimensions of universal treatment policies and gender-specific barriers to care” Malawi Ministry of Health, HIV Treatment Technical Working Group. Lilongwe, Malawi.

2016 “Facility-based barriers to HIV testing among men in Malawi: a systems approach” Malawi Ministry of Health, HIV Treatment Technical Working Group. Lilongwe, Malawi.

2015 “Men’s heightened risk of AIDS-related death: the legacy of gendered HIV testing and treatment strategies” United Nations Meeting on Male Engagement. Geneva, Switzerland

2015 “Facility-based barriers to men’s use of HIV testing: recommendations for male engagement guidelines.” National AIDS Control Council Meeting for the Development of the Male

Engagement Guidelines. Nairobi, Kenya

2014 "Gendered care: examining who 'does gender' in clinical settings and its influence on HIV services for men in southern Malawi." Health Working Group, UCLA. Los Angeles, CA

2013 "From questions to methods: mixed methods approach to disparities research." Course in Qualitative Methods (Doctoral Students). University of Colorado Denver. Denver, C

2010 "Lost in translation: examples of why best-practice nutrition programs fail in rural Dominican Republic." Drabkin and Bixby International Conference, UCLA. Los Angeles, CA

RELEVANT EMPLOYMENT ACTIVITIES

2015- Consultant for Mixed Methods, Invest in Knowledge, Zomba, Malawi

Activities: Oversaw data analysis and write-up for studies implemented by Invest in Knowledge. I focused on qualitative and mixed methods analysis and write-up.

2009-10 Research Specialist, Korean Resource Center, Los Angeles, CA

Activities: Managed data entry and data cleaning and led in data analysis and write-up of a study assessing use of non-communicable disease services among first- and second-generation Korean populations in Los Angeles.

2009 Program Evaluation Fellow, Bataye Relief Alliance, Santo Domingo, Dominican Republic

Activities: Led the assessment of a nutritional program aimed to improve child health outcomes in Haitian populated bateyes in Dominican Republic. I led tool development, training enumerators, data analysis, and write-up

2007-08 Program Coordinator, Orange County Department of Public Health, Santa Ana, CA

Activities: Conducted literature reviews and assisting in the development of interventions to address Alcohol and Drug abuse among young adults in Orange County. Assisted in the protocol development and implementation of interventions.

SERVICE

2019 Committee Member of the Men's HIV Forum at the International AIDS Conference, Mexico City

2018- Member of the Malawi Ministry of Health HIV Self-Testing Guidelines Task Force

2017- Member of the Malawi Ministry of Health HIV Testing Services Technical Working Group

2017- Member of the EQUIP HIV Self-Testing Technical Working Group

2016- Member of the UNAIDS Working Group "Engaging men in solutions for the HIV epidemic: Health systems."

2016- Member of the "Men and HIV Global Working Group"

2016 Reviewer for the APHA 2016 Annual Meeting & Expo

2012-13 Editor of the Health and Behavioral Sciences Peer-Reviewed Journal, University of Colorado, Denver

2011-12 Student Advisory Council Member, University of Colorado, Denver

For peer review only

COURSES TAUGHT

Adjunct Professor

Social determinants of health in the context of HIV services in sub-Saharan Africa – Field Rotation Series (UCLA)

Health, Disease & Globalization: Foundations of Epidemiology (Vanguard University)

Human Sexuality (co-taught, Vanguard University)

Cultural Anthropology (Vanguard University)

Applied Anthropology (Vanguard University)

Qualitative Methods (Vanguard University)

Teaching Assistant

AIDS and Other Sexually Transmitted Diseases (UCLA)

Global Health Issues (UCLA)

Social Determinants of Health (University of Colorado Denver)

Statistical Analysis (University of Colorado Denver)

MENTORSHIP

University of California Los Angeles. David Geffen School of Medicine. Medical Student. Kate Coursey. "Examining characteristics of women who engage in an integrated Early Childhood Development and PMTCT program in Malawi: endline evaluation." 2019-

University of California Los Angeles. David Geffen School of Medicine. Medical Student. "Provider acceptability of interventions to increase ART initiation among men who test HIV-positive through index HIV self-testing." 2019-

University of California Los Angeles. David Geffen School of Medicine. Medical Student. Tijana Temelkovska. "Examining the successes and challenges of implementing an early childhood development intervention with HIV-positive women in Malawi: a process evaluation." 2018-

University of California Los Angeles. Internal Medicine Residency, Global Health Track. Resident Physician. Marguerite Thorp. "Can a brief screening tool identify ART clients at risk of defaulting from treatment? a prospective study in Malawi." 2018-

University of California Los Angeles. Internal Medicine Residency, Global Health Track. Resident Physician. Adrian Mayo. "Predictors of early ART retention among adults who initiated under Universal Treatment policies in Malawi." 2018-

University of Colorado Denver. Health and Behavioral Sciences. Doctoral Student. Stephanie Chamberlin. "Exploring the association between education and ART retention in rural Malawi." 2017-

University of California Los Angeles. Fogarty GloCal Fellow. Medical Student. Ogechukwu Offorjebe. "Examining the feasibility and acceptability of HIV self-test kits for index testing among HIV+ clients and their partners in Malawi: A mixed methods study." 2017-18

College of Medicine, Malawi. MPH Student. Khumbo Phiri. "The role of lay cadre in ART initiation and retention under Test and Treat in Malawi." 2017-18

For peer review only

Brandeis University. Elisa Morales, Becca Sliwosk, and Melanie Morris (capstone project). "Developing a funding proposal for Men-to-Men, a gender-transformation and income-generating program for men in northern Uganda." 2015 (with 31Bits International)

Vanguard University. Medical Anthropology Honors Thesis. Joanna Takegami. "Barriers to Women's use of Antiretroviral Therapy in Northern Uganda: Exploring the Role of Structural Violence." 2011

AD HOC REVIEWER

AIDS, JAIDS, JIAS, BMC Public Health, Global Health Action, Culture, Health and Sexuality

PROFESSIONAL MEMBERSHIPS

Member, American Public Health Association (APHA), Present

Member, American Sociology Association (ASA), Present

Member, American Anthropological Association (AAA), Present

For peer review only

OMB No. 0925-0001 and 0925-0002 (Rev. 10/15 Approved Through 10/31/2018)

THOMAS J. COATES

eRA COMMONS USER NAME (credential, e.g., agency login): **TCOATS**

POSITION TITLE: **Professor Emeritus, Division of Infectious Diseases, Department of Medicine UCLA David Geffen School of Medicine**

EDUCATION/TRAINING *(Begin with baccalaureate or other initial professional education, such as nursing, include postdoctoral training and residency training if applicable. Add/delete rows as necessary.)*

INSTITUTION AND LOCATION	DEGREE *(if applicable)*	Completion Date MM/YYYY	FIELD OF STUDY
San Luis Rey College, San Luis Rey, California	BA	06/1968	Philosophy
San Jose State University, San Jose, California	MA	01/1971	Psychology
Stanford University, Stanford, California	PhD	06/1977	Counseling Psychology

A. Personal Statement

I am Director of the system-wide University of California Global Health Institute (founded in 2008) and was the Founding Director of the UCLA Center for World Health (founded in 2012) until 2018. In 1986 I co-founded the Center for AIDS Prevention Studies (CAPS) at UCSF and directed it from 1991 to 2003. I was also the founding Director of the UCSF AIDS Research Institute, leading it from 1996 to 2003.

I have substantial expertise in research on HIV prevention among heterosexual men and women in the HIV epidemic in sub-Saharan Africa and in the HIV testing and treatment trials in sub-Saharan Africa, especially Malawi through PEPFAR funding. As Distinguished Research Professor of Medicine, I continue with two NIH and two foundation grants focused in southern Africa. I also continue as a co-investigator on the UCLA-based Center for HIV Identification, Prevention and Treatment Studies (CHIPTS).

I have had extensive experience with large-scale, community-based, multi-site research and implementation projects spanning HIV prevention, care and treatment, and policy. I currently have funding to test and evaluate innovative strategies for bring men in South Africa into HIV testing and treatment, as well as for providing early childhood development training for HIV-infected mothers and their babies in Malawi through support from the Conrad N. Hilton Foundation. We are also in the first year of a 5-year NIH-funded grant to study pre-exposure prophylaxis for pregnant and post-partum women in South Africa.

B. Positions and Honors

For peer review only

1984 - 2003	Member, Medical Attending Staff, UCSF Hospitals and Clinics
1990 - 2003	Professor, Department of Medicine, UCSF
1991 - 2003	Director, Center for AIDS Prevention Studies, UCSF
1996 - 2003	Director, AIDS Research Institute, UCSF
2000	Elected to the Institute of Medicine (now the National Academy of Medicine)
2010 - 2014	Member, Institute of Medicine Board on Global Health
2003 - 2006	Professor Step VII, Division of Infectious Diseases, Department of Medicine, David Geffen School of Medicine, UCLA
2003 - Present	Joint Appointment, Department of Medicine, UCSF; Member, Executive Committee, UCLA AIDS Institute
2003 - 2011	Director, UCLA Program in Global Health
2004 - Present	Joint Appointment, Department of Epidemiology, UCLA School of Public Health
2006 – 2009	Professor Step IX, Division of Infectious Diseases, Department of Medicine, David Geffen School of Medicine, UCLA
2006 - 2018	Michael & Sue Steinberg Endowed Professor of Global AIDS Research, Division of Infectious Diseases, Department of Medicine, David Geffen School of Medicine, UCLA
2006 - 2018	Director, Global Capacity Building Core Center for HIV Identification, Prevention, and Treatment Services, UCLA Semel Neurosciences Institute
2006 - 2018	Associate Director for International and Policy Research UCLA AIDS Institute
2009 - 2016	Co-director, University of California Global Health Institute
2009 –2018	Distinguished Professor, Division of Infectious Diseases, Department of Medicine, David Geffen School of Medicine, University of California, Los Angeles
2011 – 2018	Director, UCLA Center for World Health at the David Geffen School of Medicine and UCLA Health
2016-Present	Director, University of California Global Health Institute
2018-Present	Distinguished Research Professor, Division of Infectious Diseases, UCLA David Geffen School of Medicine

C. Contribution to Science

1. **Combination HIV Prevention including Pre-Exposure Prophylaxis:** I have written extensively and conducted research on combination HIV prevention for MSM in the United States and Latin America and with a variety of populations in sub-Saharan Africa. My writing and research have been influential in shaping thinking about combination prevention, and in demonstrating the importance of considering HIV prevention as a combination of factors, as opposed to any single kind of program.

Joseph Davey D, Bekker LG, Gorbach P, **Coates T**, Myer L. Delivering PrEP to pregnant and breastfeeding women in sub-Saharan africa: The implementation science frontier. AIDS. 2017 Jul 18. doi: 10.1097/QAD.0000000000001604. PubMed PMID: 28723709.

For peer review only - http://bmjopen.bmj.com/site/about/guidelines.xhtml

Richter L, Komárek A, Desmond C, Celentano D, Morin S, Sweat M, Chariyalertsak S, Chingono A, Gray G, Mbwambo J, **Coates T**; Reported physical and sexual abuse in childhood and adult HIV risk behaviour in three African countries: findings from Project Accept (HPTN-043). AIDS and behavior. 2014; 18(2):381-9. PMCID: PMC3796176

Coates TJ; An expanded behavioral paradigm for prevention and treatment of HIV-1 infection. Journal of acquired immune deficiency syndromes (1999). 2013; 63 Suppl 2:S179-82. PMCID: PMC3943341

Coates TJ, Richter L, Caceres C. Behavioural strategies to reduce HIV transmission: how to make them work better. Lancet. 2008; 372(9639):669-84. PMCID: PMC2702246

2. **HIV Counseling and Testing (HTC):** I have conducted many significant and influential studies in HTC, beginning first with observational studies of the effect of HTC on risk behavior among men who have sex with men (MSM) in San Francisco. I was Principal Investigator for the first randomized controlled trial of HTC in Eastern Africa and the Caribbean, examining the effect of HTC on individual males and females, as well as couples presenting for HTC in Kenya, Tanzania, and Trinidad and Tobago, and these results were reported in *The Lancet* in 2000. I was the Principal Investigator for Project Accept, a cluster randomized trial conducted in South Africa, Zimbabwe, Tanzania, and Thailand, and these results were reported in *Lancet Global Health* in 2015. I also was the Principal Investigator of a randomized trial at Mulago Hospital in Uganda examining the effect of short vs. elaborated counseling on males and females presenting for care, and these results were reported in *Lancet Global Health* in 2014.

Coates TJ, Kulich M, Celentano DD, Zelaya CE, Chariyalertsak S, Chingono A, Gray G, Mbwambo JK, Morin SF, Richter L, Sweat M, van Rooyen H, McGrath N, Fiamma A, Laeyendecker O, Piwowar-Manning E, Szekeres G, Donnell D, Eshleman SH; NIMH Project Accept (HPTN 043) study team; Effect of community-based voluntary counselling and testing on HIV incidence and social and behavioural outcomes (NIMH Project Accept; HPTN 043): a cluster-randomised trial. The Lancet. Global Health. 2014; 2(5):e267-77. PMCID: PMC4131207

van Rooyen H1, McGrath N, Chirowodza A, Joseph P, Fiamma A, Gray G, Richter L, **Coates T**. Mobile VCT: reaching men and young people in urban and rural South African pilot studies (NIMH Project Accept, HPTN 043). AIDS and behavior. 2013; 17(9):2946-53. PMCID: PMC3597746

Wanyenze RK, Kamya MR, Fatch R, Mayanja-Kizza H, Baveewo S, Szekeres G, Bangsberg DR, **Coates T**, Hahn JA; Abbreviated HIV counselling and testing and enhanced referral to care in Uganda: a factorial randomised controlled trial. The Lancet. Global Health. 2013; 1(3):e137-45. PMCID: PMC4129546

Mhlongo S, Dietrich J, Otwombe KN, Robertson G, **Coates TJ**, Gray G.Factors associated with not testing for HIV and consistent condom use among men in Soweto, South Africa. PloS one. 2013; 8(5):e62637. PMCID: PMC3656000

3. **Global Health:** I have contributed to the literature on global health, especially from the perspective of engaging multiple disciplinary perspectives to attend to a variety of global health issues around the world.

For peer review only

Debas HT, **Coates TJ**; The University of California Global Health Institute opportunities and challenges. Infectious disease clinics of North America. 2011; 25(3):499-509, vii. PubMed [journal]PMID: 21896355

Duber HC, **Coates TJ**, Szekeras G, Kaji AH, Lewis RJ; Is there an association between PEPFAR funding and improvement in national health indicators in Africa? A retrospective study. Journal of the International AIDS Society. 2010; 13:21. PMCID: PMC2895577

Maman S, Abler L, Parker L, Lane T, Chirowodza A, Ntogwisangu J, Srirak N, Modiba P, Murima O, Fritz K.A comparison of HIV stigma and discrimination in five international sites: the influence of care and treatment resources in high prevalence settings. Social science & medicine (1982). 2009; 68(12):2271-8. PMCID: PMC2696587

Collins C, Coates TJ, Szekeres G; Accountability in the global response to HIV: measuring progress, driving change. AIDS (London, England). 2008; 22 Suppl 2:S105-111. PMCID: PMC2879260

Complete List of Published Work in MyBibliography:
http://www.ncbi.nlm.nih.gov/sites/myncbi/thomas.coates.1/bibliography/40839346/public/?sort=date&direction=descending

D. Research Support

Ongoing Research Support

R01MH105534-01A1 (Coates) 07/07/15 – 04/30/20
NIH/NIMH
Bringing South African Men into HIV Counseling and Testing (HCT) and Care
The objective of this project is to provide evidence-based strategies to improve treatment of HIV+ men through a three-step process: (1) Testing a significant proportion of the population, (2) linkage to care, and (3) maintaining in care a significant proportion of HIV+ individuals to the point of viral suppression. My role is as the Principal Investigator.

UM1 AI068619 (El Sadr) 07/01/14 – 11/30/20
Family Health International
NIH-NIAID
HIV Prevention Trials Network (HPTN) Leadership Group
The goals of this project are: 1) to develop the HPTN research agenda; 2) to review SWG research plans; 3) to review and approve concept plans; 4) to oversee the discretionary fund; 5) to review and revise HPTN policies and procedures; and 6) to evaluate the performance of the HPTN. My role is as Chair of the Manuscript Review Committee

P30 MH058107 (Shoptaw) 03/01/2017-02/28/2022

NIMH/NIH

For peer review only

Center for HIV Identification, Prevention, and Treatment Services

This project is a P30 and provides center grant services to HIV investigators at UCLA. I am a Co-Investigator in this center.

The Conrad N. Hilton Foundation 01/01/2018-12/31/2020

Delivery of Childhood Development Services as Part of HIV Treatment Services in Malawi

This grant supports the integration of early childhood development services within pre- and post-natal care for HIV+ mothers in Malawi.

R01 MH116771-01A1 09/30/2018-09-29-2023

NIMH/NIH

Evaluating the Prep-PP Cascade in HIV-negative Pregnant and Breastfeeding Women in South Africa.

The goal of this project is to test innovative models for delivering PrEP to pregnant and breastfeeding women age 16 and above in South Africa.

Entertainment Industry Foundation-Charlize Theron Africa Outreach Project 06/01/2018-05/30/2021

The goal of this project is to create a Youth Leaders Scholarship Fund to support promising young South Africans to attend South African tertiary education institutions.

Bill and Melinda Gates Foundation **(**Dovel) 12/4/2019-12/3/2023

Identifying Effective Linkage Strategies for HIVST (IDEaL)

This grant tests the effect of a staged intervention for ART initiation among men in Malawi, whereby additional interventions are added each month for individuals who have not yet initiated ART.

Completed Research Support

20150025 (Coates) 09/01/15 – 08/31/17
Conrad N. Hilton Foundation
Delivery of Early Childhood Development Services as a Part of HIV Treatment Services in Malawi
Pilot grant to assess the feasibility and acceptability, as well as initial outcomes, of supporting Option B+ mothers in Malawi to increase their responsiveness to their children and have positive impacts on early childhood development (ECD).

P30 MH58107 (Rotheram-Borus) 02/01/07 - 01/31/17

NIH/NIMH

Center for HIV Identification, Prevention, and Treatment Services (CHIPTS)

The mission of the Center for HIV Identification, Prevention, and Treatment Services (CHIPTS) is to promote collaborative research and education on effective HIV detection, prevention, and treatment programs for HIV at the societal, community, provider, and individual levels. My role is as the Director for International Care.

For peer review only

For peer review only

RISA MICHELLE HOFFMAN

CURRICULUM VITAE

PERSONAL HISTORY

David Geffen School of Medicine at UCLA
Division of Infectious Diseases
10833 Le Conte Ave 37-121 CHS
Los Angeles, CA 90095
rhoffman@mednet.ucla.edu
Tele: (310) 825-7225
Fax: (310) 825-3632

EDUCATION

Stanford University 1994, BA
University of California Los Angeles 2000, MD
Harvard School of Public Health 2000, MPH
Internship 2000-2001: Harvard Combined Medicine/Pediatrics Residency Program
Residency 2001-2004: Harvard Combined Medicine/Pediatrics Residency Program
Fellowship Infectious Diseases: 2005-2008: University of California, Los Angeles

LICENSURE

California, A85173, 01/31/2021

BOARD CERTIFICATION/OTHER CERTIFICATION

2004 & 2014 American Board of Internal Medicine
2007 & 2017 American Board of Internal Medicine, Infectious Diseases
2005 Certification in Travel Medicine from the London School of Hygiene and Tropical Medicine

PROFESSIONAL EXPERIENCE

Present Position

2016-present	Associate Clinical Professor, Division of Infectious Diseases, UCLA
2010-2016	Assistant Clinical Professor, Division of Infectious Diseases, UCLA Medical Center, Los Angeles, California
2008-2010	Clinical Instructor, Division of Infectious Diseases, UCLA Medical Center, Los Angeles, California

Previous Positions

2005-2008	Fellow in Infectious Diseases, UCLA Medical Center, Los Angeles, California
2001-2004	Resident Physician, Internal Medicine, Brigham and Women's Hospital, Boston, Massachusetts
2001-2004	Resident Physician, Pediatrics, Boston Children's Hospital and Massachusetts General Hospital, Boston, Massachusetts
2000-2001	Intern, Internal Medicine, Brigham and Women's Hospital, Boston, Massachusetts
2000-2001	Intern, Pediatrics, Boston Children's Hospital and Massachusetts General Hospital, Boston, Massachusetts

For peer review only

PROFESSIONAL ACTIVITIES & MEMBERSHIPS

2018-present	Interim Director, Global Health Education and Research Program, David Geffen School of Medicine at UCLA
2016-present	Co-Director UCLA AIDS Institute/CFAR International Health Services and Policy Research Program Section
2015-present	Associate Program Director, UCLA Infectious Diseases Fellowship Training Program
2013-present	Advisory Board Member for the University of California Global Health Institute GloCal Health Fellowship
2009-present	Research Co-Director, Partners in Hope Malawi and UCLA Research Collaboration
2009-present	Investigator, AIDS Clinical Trials Group (ACTG) and Maternal Child Adolescent Network (IMPAACT)
2009-present	HIV Clinical Consultant, To Help Everyone Clinic in Los Angeles, California
2009-present	Ad hoc Peer Reviewer (AIDS Care, International Journal of STD and AIDS, BMC Women's Health, American Society of Tropical Medicine and Hygiene, Journal of Infectious Diseases, International Health, JIAS)
2007-present	Member, Infectious Diseases Society of America (IDSA)
2016-2018	Committee Member, Antiretroviral Therapy Strategies (ARTs), AIDS Clinical Trials Group
2008-2016	Founder/Program Director, Sustainable Nutrition for Orphans and Vulnerable Children in Malawi, Central Africa: Provides education on nutrition and sustainable food sources for families caring for orphans in northern Malawi
2014-2016	Committee Lead, Infectious Diseases Quality Improvement M&M Program
2011-2016	Committee Member, AIDS Clinical Trials Group *Women's Health Inter-network Scientific Committee (WHISC)*
2007-2013	Founder/Program Co-Director, UCLA resident physician elective training program in Malawi, Africa
2010-2013	Co-Director, UCLA Program in Global Health and Global Health Education Program for the David Geffen School of Medicine at UCLA
2008-2012	Faculty for 'Multidisciplinary Approach to Global Health' elective course for first and second year medical students at UCLA
2007-2012	Committee Member, American Society of Tropical Medicine and Hygiene Education Committee
2005-2012	Advisory Board Member, UCLA Medicine/Pediatrics Residency Training Advisory Board
2005-2011	Interviewer, UCLA Medicine/Pediatrics Residency Training Program
2006-2008	Faculty Group Leader, Problem Based Learning Microbiology Block for second year medical students at UCLA
2006-2008	Creator/Organizer, UCLA Infectious Diseases Core Curriculum Program

HONORS AND AWARDS

2012	David Geffen School of Medicine Award for Excellence in Education
2011	Nomination for the Consortium of Universities for Global Health Early Career Award
2009	Nomination for UCLA Faculty Teaching Award

For peer review only

2007 Nomination for UCLA Fellow Teaching Award
2006 Nomination for UCLA Fellow Teaching Award

2000 Elected to the UCLA chapter of the Alpha Omega Alpha Honor Society
2000 Janet M. Glasgow Memorial Achievement Citation for Academic Achievement at the UCLA School of Medicine
2000 John M. Adams Award for Excellence in Pediatrics, UCLA School of Medicine
2000 Edith and Carl Lasky Memorial Award for Outstanding Research Achievement, UCLA School of Medicine
1999 Longmire Surgical Medal for outstanding performance in surgical clerkships, awarded by the Department of Surgery, UCLA School of Medicine
1999 Charles Wacker Summer Research Fellowship, UCLA School of Medicine
1994 Elected to the Stanford Chapter of Phi Beta Kappa
1994 Elected to the Stanford Cap and Gown Women's Honor Society
1994 Recipient of the Joshua Lederberg Award for Outstanding Academic Achievement in Human Biology

LECTURES AND PRESENTATIONS

"Management of Febrile Neutropenia" Presented as part of the UCLA Division of Infectious Diseases Core Curriculum Program. Los Angeles, California, November 2013

"Challenges and Successes of EQUIP Malawi" Presented at UCLA Infectious Diseases Grand Rounds. Los Angeles, California, April 2014

"Primary Care Issues in HIV Care" Presented at the UCLA Department of Medicine housestaff curriculum. Los Angeles, California, May 2014

"A Case of Multi-Class HIV Resistance" Presented at the UCLA HIV/Hepatitis C Case Conference Series, Los Angeles, California, May 2014

"Evidence Based Managed of Osteomyelitis" Presented at the UCLA Division of Infectious Diseases Case Conference Series, Los Angeles, California, June 2014

"Responding to Viral Load: A Primer for Malawi Clinical Mentors" Presented at a PEPFAR EQUIP Clinical Training Meeting in Malawi, Africa, January 2015

"Clinical Pearls in the Management of HIV/AIDS". Presented at the UCLA Internal Medicine Resident Core Curriculum Conference Series, Los Angeles, California, February 2015

"Quality Improvement in ID Care at UCLA: Lessons Learned from M&M" Presented at the UCLA Division of Infectious Diseases Core Conference Series, Los Angeles, California, April 2015

For peer review only

“Health & Safety Overseas: An orientation for medical students” Presented at the UCLA Global Health Education Medical Student Orientation Program, Los Angeles, California, April 2015

“ID Mimics”. Presented at the UCLA ID Fellow Core Curriculum Series, Los Angeles, California, May 2015

“Quality Improvement on the Infectious Diseases Service: Transition of Care.” Presented at the UCLA Division of Infectious Diseases Case Conference, Los Angeles, California, June 2015

“Quality Improvement on the Infectious Diseases Service: Notes and Documentation.” Presented at the UCLA Division of Infectious Diseases Case Conference, Los Angeles, California, December 2015

“Quality Improvement on the Infectious Diseases Service: HIV Care”. Presented at the UCLA Division of Infectious Diseases Case Conference, Los Angeles, California, February 2016

“Multi-month scripting to achieve improved outcomes in EQUIP”. Presented at the EQUIP annual meeting, Johannesburg, South Africa March 2016

“Clinical Management of HIV/AIDS for the Primary Care Resident”. Presented at the UCLA Internal Medicine Resident Core Curriculum Conference Series, Los Angeles, California, April 2016

“Update on Option B+ in Malawi”. Presented to the Women’s Health Committee of the AIDS Clinical Trials Group, Los Angeles, California, April 2016

EQUIP Malawi: A Partnership for HIV Care in Malawi. Presented at Harbor UCLA Infectious Diseases Grand Rounds, Los Angeles, California, July 2016

Speaker, Infectious Diseases Career Panel for Medical Students at the David Geffen School of Medicine. Los Angeles, September 2016

Systemwide Case Conference Faculty Discussant for the MultiCampus Infectious Diseases Fellowship Program. Presented at the VA Hospital, Los Angeles, California, December 2016

“Introduction to Global HIV Treatment in Resource Poor Settings,” Lecturer for the UCLA School of Public Health, February 2018, Los Angeles

UCLA Division of Infectious Diseases, Journal Club Faculty Discussant, MDR TB Treatment, March 2018, Los Angeles

For peer review only

Faculty Panelist. Global Health Career Night for the David Geffen School of Medicine. November 2018, Los Angeles

West LA VA Internal Medicine Grand Rounds Speaker: "The Intersection of HIV and Non-Communicable Diseases in Resource-Limited Settings" April 2019, Los Angeles

"Qualitative Client and Provider Experiences with Multi-Dispensing for HIV in Malawi and Zambia". Presented as part of the CQUIN Consortium. Webinar, April 2019

"Introduction to the Global Health Program". Presented as part of the DGSOM Global Health Selective, September 2019, Los Angeles

PUBLICATION/BIBLIOGRAPHY

RESEARCH PAPERS

RESEARCH PAPERS (PEER REVIEWED)

Hoffman RM, Umeh OC, Garris C, Givens N, Currier JS. Evaluation of Sex Differences of Fosamprenavir (With and Without Ritonavir) in HIV-infected Men and Women. HIV Clin Trials. 2007;8(6):371-380.

Hoffman RM, AboulHosn J, Child JS, Pegues DA. Bartonella Endocarditis in Complex Congenital Heart Disease. Congenit Heart Dis. 2007;2(1):79-84.

Black V, **Hoffman RM**, Sugar CA, Menon P, Venter FWD, Currier JS, Rees H. Safety and Efficacy of Initiating Highly Active Antiretroviral Therapy in an Integrated Antenatal and HIV Clinic in Johannesburg, South Africa. J Acquir Immune Defic Syndr. 2008;49(3):276-81. PMC2893046.

Hoffman RM, Black V, Technau K, van der Merwe KJ, Currier JS, Coovadia A, Chersich M. Effects of Highly Active Antiretroviral Therapy Duration and Regimen on Risk for Mother-to-Child Transmission of HIV in Johannesburg, South Africa. J Acquir Immune Defic Syndr. 2010;54(1):35-41. PMC2880466.

Pilotto JH, Velasque L, Khalili R, Ismerio R, Veloso VG, Grinsztejn B, Morgado MG, Watts DH, Currier JS, **Hoffman RM**. Maternal Outcomes after HAART for Prevention of Mother-to-Child Transmission in HIV-infected Women in Brazil. Antivir Ther. 2011;16(3):349-56. PMC3437753.

Hoffman RM, Jamieson BD, Bosch RJ, Currier JS, Kitchen CMR, Schmid I, Zhu Y, Bennett K, Mitsuyasu R. Baseline Immune Phenotypes and CD4+ T Lymphocyte Responses to Antiretroviral

For peer review only

Therapy in Younger versus Older HIV-infected Individuals. J Clin Immunol. 2011;31(5):873-81. PMC3194061.

Van der Merwe J, Hoffman RM, Black V, Chersich M, Coovadia A, Rees H. Birth outcomes in South African Women Receiving Highly Active Antiretroviral Therapy: a Retrospective Observational Study. J Int AIDS Soc. 2011;14:42. PMC3163172.

Mindry D, Wagner G, Lake JE, Smith A, Linnemayr S, Quinn M, **Hoffman RM**. Fertility Desires Among HIV-infected Men and Women in Los Angeles County: Client Needs and Provider Perspectives; Matern Child Health J. 2013 May;17(4):593-600. PMC N/A.

Burke Z, Chen J, Conceicao C, **Hoffman R**, Miller L, Taela A, DeUgarte DA. Evaluation of Preoperative and Intraoperative RBC Transfusion Practices in Maputo Central Hospital, Mozambique. Transfusion. 2013 May 21. doi: 10.1111/trf.12252. PMC3751985.

Hoffman RM, Leister E, Kacanek D, Shapiro DE, Read JS, Bryson Y, Currier JS. Biomarkers from late pregnancy to six weeks postpartum in HIV-infected women who continue versus discontinue antiretroviral therapy after delivery. JAIDS. 2013 May 8. PMC3868443.

Jaganath D, Mulenga C, **Hoffman R**, Hamilton J, Boneh G. This is My Story: Participatory Performance for HIV and AIDS Education at the University of Malawi. Health Education Research. 2013 Sep 18. PMC4155417.

Kawale P, Mindry D, Stramotas S, Chilikoh P, Phoya A, Henry K, Elashoff D, Jansen P, **Hoffman R**. Factors associated with desire for children among HIV-infected women and men: A quantitative and qualitative analysis from Malawi and implications for the delivery of safer conception counseling. AIDS Care. 2013 Jun;26(6). PMC3943633.

1. Iroezi N, Mindry D, Kawale P, Chikowi G, Jansen P, **Hoffman R**. A qualitative analysis of the barriers and facilitators to receiving care in a prevention of mother-to-child program in Nkhoma, Malawi. Afr JReprod Health. 2013 Dec;17(4). PMC4361063.

2. Russell E, Mohammed T, Smeaton L, Jorowe B, MacLeod I, **Hoffman R**, Currier JS, Moyo S, Essex M, Lockman S. Immune activation markers in peripartum women in Botswana: association with feeding strategy and maternal morbidity. PLoS One. 2014 Mar 21. PMC3962339.

3. Reddy D, Njala J, Stocker P, Schooley A, Flores M, Tseng C-H, Pfaff C, Jansen P, Mitsuyasu RT, **Hoffman RM**. High-risk human papillomavirus in HIV-infected women undergoing cervical cancer screening in Lilongwe, Malawi: A pilot study. International Journal of STDS and AIDS, 2014 Jun 13. PMC4363075.

4. Kamuyango A, Hirschhorn L, Wang W, Jansen P, **Hoffman R**. One-Year Outcomes of Women Started on Antiretroviral Therapy during Pregnancy before and after the Implementation of Option B+ in Malawi: A Retrospective Chart Review from Three Facilities. World Journal of AIDS. 2014 Sept;4(3). PMC4356991.

5. Shull H, Tymchuk C, Grogan T, Hamilton J, Friedman J, **Hoffman RM**. Evaluation of the UCLA Department of Medicine Malawi Global Health Clinical Elective: Lessons from the First Five Years. Am J Trop Med Hyg. 2014 Sep 15. PMC4228879.

For peer review only

6. Hoffman JC, Anton PA, Baldwin GC, Elliott J, Anisman-Posner D, Tanner K, Grogan T, Elashoff D, Sugar C, Yang OO, **Hoffman RM**. Seminal Plasma HIV-1 RNA Concentration is Strongly Associated with Altered Levels of Seminal Plasma Interferon Gamma, Interleukin-17, and Interleukin-5. AIDS Res Hum Retroviruses. 2014 Oct 2. PMC4208556.

7. Menon P, **Hoffman R**, Black V. Characteristics of HIV-infected women on antiretroviral therapy who develop preeclampsia in South Africa: A Case Series. The Journal of Global Health. 2014;4(2). PMC N/A.

8. Cheng Q, Engelage E, Grogan T, Currier JS, and **Hoffman RM**. Who provides primary care? A cross-sectional survey of HIV patients and providers in a Los Angeles clinic. AIDS Clinical Research. 27 Oct 2014;5(11). PMC4409003.

9. Kawale P, Mindry D, Phoya A, Jansen P**, Hoffman RM**. Provider attitudes about childbearing and knowledge of safer conception at two HIV clinics in Malawi. Reproductive Health. 2015 Mar 7;12(1):17. PMC4355153.

10. Yeatman S, **Hoffman RM,** Chilungo A, Lungu S, Namadingo H, Chimwaza A, and Trinitapoli JA. Health-seeking behavior and symptoms associated with early HIV infection: Results from a population-based cohort in southern Malawi. JAIDS. 2015 May 1;69(1):126-30. PMC4422188.

11. **Hoffman RM**, Jaycocks A, Vardavas V, Wagner G, Lake JE, Mindry D, Currier JS, Landovitz R. Benefits of PrEP as an adjunctive method of HIV prevention during attempted conception between HIV-uninfected women and HIV-infected male partners. Journal of Infectious Diseases, J Infect Dis. 2015 Jun 19. PMC4621256.

12. Lake JE, **Hoffman RM**, Tseng CH, Wilhalme HM, Currier JS. Success of Standard Dose Vitamin D Supplementation in Treated HIV Infection. Open Forum Infect Dis. 2015 May 15;2(2). PMC4462892.

13. Chipungu C, Veltman JA, Jansen P, Chiliko P, Lossa C, Namarika D, Benner B, **Hoffman RM**, Bristow CC, Klausner JD. Feasibility and Acceptability of Cryptococcal Antigen Screening and Prevalence of Cryptocococcemia in Patients Attending a Resource-limited HIV/AIDS clinic in Malawi. Journal of the International Association of Providers of AIDS Care, J Int Assoc Provid AIDS Care. 2015 Jul 2. PMC N/A.

14. Coelho L, Cardoso SW, Luz PM, **Hoffman RM**, Mendonca L, Veloso VG, Currier JS, Grinsztejn B, Lake JE. Vitamin D3 supplementation in HIV infection: effectiveness and associations with antiretroviral therapy. Nutr J. 2015 Aug 18: 14. PMC4538921.

15. **Hoffman RM**, Lake JE, Wilhame H, Tseng CH, Currier JD. Vitamin D levels and markers of inflammation and metabolism in HIV-infected individuals on suppressive antiretroviral therapy. AIDS Res and Human Retroviruses 2016 March;32(3). PMC4779972.

16. Herbst de Cortina S, Arora G, Wells T, **Hoffman RM**. Evaluation of a Structured Predeparture Orientation at the David Geffen School of Medicine's Global Health Education Programs. Am J Trop Med Hyg. 2016 Mar 2;94(3). PMC4775891.

For peer review only

17. Jordan J, **Hoffman R**, Arora G, Coates W. Activated learning: providing structure in global health education at the David Geffen School of Medicine at the University of California, Los Angeles – a pilot study. BMC Medical Education. 2016 Feb 16. PMC4755030.

18. Wagner GJ, Linnemayr S, Ghosh-Dastidar B, Currier JD, **Hoffman R**, Schneider S. Supporting Treatment Adherence Readiness through Training (START) for patients with HIV on antiretroviral therapy: a study protocol for a randomized controlled trial. Trials. 2016 March 24;17(1). PMC4806419.

19. Arora G, Perkins K, **Hoffman RM**. Optimizing global health electives through partnerships: A pilot study of pediatric residents. Academic Pediatrics. 2015 Sept-Oct;15(5). PMC N/A.

20. Chien E, Phiri K, Schooley S, Chivwala M, Hamilton J, **Hoffman RM**. Successes and Challenges of HIV Mentoring in Malawi: The Mentee Perspective. PLoS One. 2016 Jun 18;11(6). PMC4924818.

21. Schooley A, Kamudumuli P, Vangala S, Tseng CH, Soko C, Parent J, Phiri K, Jahn A, Namarika D, **Hoffman RM**. CD4 Variability in Malawi: Implications for Use of a CD4 Threshold of 500 Cells/mm3 Versus Universal Eligibility for Antiretroviral Therapy. Open Forum Infectious Diseases. 2016 Aug 29;3(3). PMC5047419.

22. Hemarajata P, Baghdadi JD, **Hoffman R**, Humphries RM. Burkholderia pseudomallei: Challenges for the Clinical Microbiology Laboratory. J Clin Microbiol 2016 Dec;54(12):2866-2873. PMC5121373.

23. Pfaff C, Scott V, **Hoffman R**, Mwagomba B. You can treat my HIV – But can you treat my blood pressure? Availability of integrated HIV and non-communicable disease care in northern Malawi. Afr J Prim Health Care Fam Med. 2017 Feb 15;9(1):e1-e8. PMC5320467.

24. Gibb J, Chitsulo J, Chipungu C, Chivwara M, Schooley A, **Hoffman RM**. Supporting Quality Data Systems: Lessons Learned from Early Implementation of Routine Viral Load Monitoring at a Large Clinic in Lilongwe, Malawi. Clinical Research in HIV AIDS and Prevention. 2017 Mar 14;2(4). PMC5502771

25. Arora G and **Hoffman RM**. Development of an HIV Postexposure Prophylaxis (PEP) Protocol for Trainees Engaging in Academic Global Health Experiences. Acad Med. 2017 Apr 25. PMC28445222.

26. **Hoffman RM**, Phiri K, Parent J, Grotts J, Elashoff D, Kawale P, Yeatman S, Currier JS, Schooley A. Factors associated with retention in Option B+ in Malawi: a case control study. J Int AIDS Soc. 2017 Apr 27;20(1):1-8. PMC28453243.

27. Currier JS, Britto P, **Hoffman RM**, Brummel S, Masheto G, Joao E, Santos B, Aurpibul L, Losso M, Pierre MF, Weinberg A, Gnanashanmugam D, Chakhtoura N, Klingman K, Browning R, Coletti A, Mofenson L, Shapiro D, Pilotto J. Randomized Trial of Stopping or Continuing ART among Postpartum Women with Pre-ART CD4 $\geq$ 400 cells/mm3 (PROMISE 1077HS). PLoS One. 2017 May 10;12(5):e0176009. PMC28489856.

28. **Hoffman R**, Bardon A, Rosen S, Fox M, Kalua T, Xulu T, Taylor A, Sanne I. Varying intervals of antiretroviral medication dispensing to improve outcomes for HIV patients (The INTERVAL

For peer review only

Study): study protocol for a randomized controlled trial. Trials. 2017 Oct 13;18(1):476. PMC29029644.

29. Judson SD, LeBreton M, Fuller T, **Hoffman RM**, Njabo K, Brewer TF, Dibongue E, Diffo J, Kameni JF, Loul S, Nchinda GW, Njouom R, Nwobegahay J, Takuo JM, Torimiro JN, Wade A, Smith TB. Translating Predictions of Zoonotic Viruses for Policymakers. Ecohealth. 2017 Dec 11. PMC29230614.

30. Fatti G, Ngorima-Mabhena N, Chirowa F, Chirwa B, Takarinda K, Tafuma TA, Mahachi N, Chikodzore R, Nyadundu S, Ajayi CA, Mutasa-Apollo T, Mugurungi O, Mothibi E, **Hoffman RM**, Grimwood A. The effectiveness and cost-effectiveness of 3- vs. 6-monthly dispensing of antiretroviral treatment (ART) for stable HIV patients in community ART-refill groups in Zimbabwe: study protocol for a pragmatic, cluster-randomized trial. Trials. 2018 Jan 29;19(1):79. PMC29378662.

31. Caplan MR, Phiri K, Parent J, Phoya A, Schooley A, **Hoffman RM**. Provider Perspectives on Barriers to Reproductive Health Services for HIV-Infected Clients in Central Malawi. Clinical Obstetrics, Gynecology and Reproductive Medicine. 2018 Feb 12;4(1). PMC6391881.

32. Canan T, **Hoffman RM**, Schooley A, Boas Z, Schwab K, Kahn D, Shih R, Phiri K, Parent J, Banda BA, Chagoma R, Chipungu C, Pool K-L. Training Course in Focused Assessment with Sonography for HIV/TB in HIV Prevalent Medical Centers in Malawi. Journal of Global Radiology. 2018 March;4(1). PMC N/A.

33. Rough K, Seage GR, Williams PL, Hernandez-Diaz S, Huo Y, Chadwick EG, Currier JS, **Hoffman RM**, Barr E, Shapiro DE, Patel K; PHACS and the IMPAACT P1025 Study Teams. Birth Outcomes for Pregnant Women with HIV Using Tenofovir–Emtricitabine. New England Journal of Medicine. 2018 April 26;378(17):1593-1603. PMC5984044

34. Dovel K, Shaba F, Nyirenda M, Offorjebe OA, Balakasi K, Phiri K, Nichols B, Tseng C-H, Bardon A, Ngona K, **Hoffman R**. Evaluating the Integration of HIV Self-Testing Into Low-Resource Health Systems: Study Protocol for a Cluster-Randomized Control Trial from EQUIP Innovations. Trials. 2018 Sept 17;19(1). PMC6142354

35. Schwab K, **Hoffman RM**, Phiri L, Kahn D, Longwe L, Banda BA, Gama K, Chimombo M, Shih R, Schooley A, Pool K-L. Remote Training and Oversight of Sonography for Human Immunodeficiency Virus–Associated Tuberculosis in Malawi. Journal of the American College of Radiology. 2018 Sept 26. PMC6540803

36. Hubbard J, Kakwesa G, Nyirenda M, Mwambene J, Bardon A, Balakasi K, Dovel K, Kalua T, **Hoffman RM**. Towards the Third 90: Improving Viral Load Testing with a Simple Quality Improvement Program in Health Facilities in Malawi. Int Health. 2018 Nov 1. PMC Pending

37. Moucheraud C, Chasweka D, Nyirenda M, Schooley A, Dovel K, **Hoffman RM**. Simple Screening Tool to Help Identify High-Risk Children for Targeted HIV Testing in Malawian Inpatient Wards. J Acquir Immune Def Syndr. 2018 Nov 1; 79(3). PMC Pending

38. **Hoffman RM**, Brummel SS, Britto P, Pilotto JH, Masheto G, Aurpibul L, Joao E, Purswani MU, Buschur S, Flore Pierre M, Coletti A, Chakhtoura N, Klingman KL, Currier JS for the 1077HS

For peer review only

PROMISE Team. Adverse Pregnancy Outcomes among Women who Conceive on Antiretroviral Therapy. Clin Infect Dis 2018 June 1. PMC6321847

39. Nhassengo P, Cataldo F, Magaco A, **Hoffman RM**, Nerua L, Saide M, Cuco R, Hoek R, Mbofana F, Couto A, Gudo E, Chicumbe S, Dovel K. Barriers and facilitators to the uptake of Test and Treat in Mozambique: a qualitative study on patient and provider perceptions. PLOS One. 2018 Dec 26; 13(12). PMC6306230

40. Magaço A, Dovel K, Cataldo F, Nhassengo P, **Hoffman RM**, Nerua L, Tique J, Saide M, Couto A, Mbofana F, Gudo E, Cuco R, Chicumbe S. 'Good health' as a barrier and facilitator to ART initiation: a qualitative study in the era of test-and-treat in Mozambique. Culture, Health and Sexuality. 2018. PMC Pending

41. **Hoffman RM**, Konstantia A, Brummel S, Saidi F, Violari A, Dula D, Mave V, Fairlie L, Gerhard T, Kamateeka M, Chipato T, Chi B, Stranix-Chibanda L, Nematadzira T, Moodley D, Bhattacharya D, Gupta A, Coletti A, McIntyre JA, Klingman KL, Chakhtoura N, Shapiro DE, Folwer MG, Currier JS. Maternal health outcomes among HIV-infected breastfeeding women with high CD4 counts: results of a treatment strategy trial. HIV Clinical Trials 2018 Dec;19 (6). PMC6428202 [Available on 2019-12-01]

42. Murnane PM, Bacchetti P, Currier JS, Brummel S, Okochi H, Phung N, Louie A, Kuncze K, **Hoffman RM**, Nematadzira T, Soko DK, Owor M, Saidi F, Flynn PM, Fowler MG, Gandhi M. Tenofovir concentrations in hair strongly predict virologic suppression in breastfeeding women. AIDS 2019 Apr 22. [Epub ahead of print]. PMC Pending

43. Shaba F, Offorjebe OA, Phiri K, Lungu E, Kalande, P, Nyirenda M, Gupta S, **Hoffman RM**, Dovel K. Perceived acceptability of a facility-based HIV self-test intervention in outpatient waiting spaces among adult outpatients in Malawi: A formative study. J Acquir Immune Defic Syndr. 2019 Jul 1;81(3):e92-e94. PMC Pending

44. Weinberg A, Huo Y, Kacanek D, Patel K, Watts DH, Wara D, **Hoffman RM**, Klawitter J, Christians U; for IMPAACT P1025 Team. Markers of spontaneous preterm delivery in women living with HIV: relationship with protease inhibitors and Vitamin D. J Acquir Immune Defic Syndr. 2019 May 28. [Epub ahead of print]. PMC Pending

45. McBride K, Parent J, Mmanga K, Chivwala M, Nyirenda MH, Schooley A, Mwambene JB, Dovel K, Lungu E, Balakasi K, **Hoffman RM**, Moucheraud C. ART Adherence Among Malawian Youth Enrolled in Teen Clubs: A Retrospective Chart Review. AIDS Behav. 2019 July 10. PMC Pending

46. Le C, Britto P, Brummel SS, **Hoffman RM**, Li JZ, Flynn PM, Taha TE, Coletti A, Fowler MG, Bosch RJ, Gandhi RT, Klingman KL, McIntyre JA, Currier JS. Time to Viral Rebound and Safety after Antiretroviral Treatment Interruption in Postpartum Women Compared to Men. In Press AIDS 2019, August 1. PMC Pending

For peer review only

OMB No. 0925-0001 and 0925-0002 (Rev. 09/17 Approved Through 03/31/2020)

MICHAL KULICH

eRA COMMONS USER NAME (credential, e.g., agency login):

POSITION TITLE: Associate Professor of Statistics

EDUCATION/TRAINING *(Begin with baccalaureate or other initial professional education, such as nursing, include postdoctoral training and residency training if applicable. Add/delete rows as necessary.)*

INSTITUTION AND LOCATION	DEGREE *(if applicable)*	Completion Date MM/YYYY	FIELD OF STUDY
Charles University, Prague	M.S.	9/1991	Math. Statistics
Limburgs Universitair Centrum, Diepenbeek	M.S.	9/1992	Biostatistics
University of Washington, Seattle	M.S.	9/1995	Biostatistics
University of Washington, Seattle	Ph.D.	10/1997	Biostatistics

A. Personal Statement

I have an extensive past experience with design, conduct and analysis of clinical trials, especially community randomized trials, in the context of HIV prevention research. I served as the Lead Statistician for the Behavioral Working Group within the HPTN in 2000–2003 and as the Protocol Statistician and Steering Committee member for Project ACCEPT (HPTN043) in 2003-2013. I have been also participating in protocol review groups in the HPTN. I was involved in the design of HPTN043, development of data collection procedures, and development and application of data quality control measures. I am a coauthor of 7 research papers on methodology and results of HPTN043. Since 2015, I am a protocol statstician on another community-randomized trial, Zwakala Ndoda Study: Diagnosing, Linking and Maintaining Men in Antiretroviral Treatment in Vulindlela and Greater Edendale Area, KwaZulu-Natal. The current application builds on my past experience with HIV prevention trials.

B. Positions and Honors

Professional Positions

For peer review only

1998–2000 Dept. of Probability and Statistics, Charles University, Prague, Czech Rep., Assistant Professor
2000–2003 Dept. of Biostatistics, University of Washington, Seattle, Research Assistant Professor
2004–2009 Dept. of Probability and Statistics, Charles University, Prague, Czech Rep., Assistant Professor
2010–2013 Dept. of Probability and Statistics, Charles University, Prague, Czech Rep., Associate Professor
2014– Dept. of Probability and Statistics, Charles University, Prague, Czech Rep., Chair

Professional Memberships

1995- Member, American Statistical Association

1998- Member, Czech Statistical Society

2004- Member, International Biometric Society

2005- Member, International Society for Clinical Biostatistics

Honors

1995 Donovan J. Thompson Award, University of Washington, Seattle, WA.

1997 Best Written Paper, International Biometric Society, Park City, UT.

2001 Bolzano Award, Bernard Bolzano Foundation, Prague, Czech Republic.

C. Contributions to Science

Design and conduct of HIV prevention trials

I have an expertise in design, conduct and analysis of large randomized HIV prevention trials. I was a protocol statistician in Project ACCEPT (HPTN043), a community-randomized trial conducted in five African and Asian sites, with HIV incidence calculated from cross-sectional blood samples as the primary endpoint. I designed methods for obtaining population samples by household-probability sampling, participated in data verification, performed analyses and collaborated on publications.

Genberg, B., **Kulich, M.,** Kawichai, S., Modiba, P., Chingono, A., Kilonzo, G., Richter, L., Pettifor, A., Sweat, M. & Celentano, D. HIV risk behaviors in Sub-Saharan Africa and Northern Thailand: Baseline behavioral data from Project Accept. *Journal of AIDS* 2008, 49(3):309-319. PMID: 18845954

Sweat, M., Morin, S., Celentano, D., Mulawa, M., Singh, B., Mbwambo, J., Kawichai, S., Chingono, A., Khumalo-Sakutukwa, G., Gray, G., Richter, L., **Kulich, M.,** Sadowski, A., Coates, T., and the Project Accept study team. Community-based intervention to increase HIV testing and case detection in people aged 16-32 years in Tanzania, Zimbabwe, and Thailand (NIMH Project Accept, HPTN 043): a randomised study. *The Lancet Infectious Diseases* 2011, 11(7), 525-532. PMID: 21546309

Coates, T.J., **Kulich, M.**, Celentano, D.D., Zelaya, C.E., Chariyalertsak, S., Chingono, A., Gray, G., Mbwambo, J.K.K., Morin, S.F., Richter, L., Sweat, M., van Rooyen, H., McGrath, N., Fiamma, A.,

Laeyendecker, O., Piwowar-Manning, E., Szekeres, G., Donnell, D., Eshleman, S.H. (2014) Effect of community-based voluntary counselling and testing on HIV incidence and social and behavioural outcomes (NIMH Project Accept; HPTN 043): A cluster-randomised trial. *The Lancet Global Health* 2014, 2 (5), e267-e277. PMID: 25103167

Salazar-Austin, N., **Kulich, M.**, Chingono, A., Chariyalertsak, S., Srithanaviboonchai, K., Gray, G., Richter, L., van Rooyen, H., Morin, S., Sweat, M., Mbwambo, J., Szekeres, G., Coates, T., Celentano, D. (2017) Age-Related Differences in Socio-Demographic and Behavioral Determinants of HIV Testing and Counseling in HPTN 043/NIMH Project Accept. *AIDS and Behavior* 2018, 22(2) 569-579. PMID: 28589504

Methods for cross-sectional incidence estimation

I participated in the development of laboratory and statistical methods for estimating HIV incidence from cross-sectional blood samples. These methods were needed for successful evaluation of the primary outcome in Project ACCEPT.

Laeyendecker, O., Piwowar-Manning, E., Fiamma, A., **Kulich, M.**, Donnell, D., Bassuk, D., Mullis, C. E., Chin, C., Swanson, P., Hackett, Jr, J., Clarke, W., Marzinke, M., Szekeres, G., Gray, G., Richter, L., Alexandre, M. W., Chariyalertsak, S., Chingono, A., Celentano, D. D., Morin, S. F., Sweat, M., Coates, T., Eshleman, S. H. Estimation of HIV Incidence in a Large, Community-Based, Randomized Clinical Trial: NIMH Project Accept (HIV Prevention Trials Network 043), *PLoS ONE* 2013, 8:7, e68349. PMID: 23874597

Laeyendecker, O., **Kulich, M.**, Donnell, D., Komárek, A., Omelka, M., Mullis, C. E., Szekeres, G., Piwowar-Manning, E., Fiamma, A., Gray, R. H., Lutalo, T., Morrison, C. S., Salata, R. A., Chipato, T., Celum, C., Kahle, E. M., Taha, T. E., Kumwenda, N. I., Karim, Q. A., Naranbhai, V., Lingappa, J. R., Sweat, M. D., Coates, T., Eshleman, S. H. Development of Methods for Cross-Sectional HIV Incidence Estimation in a Large, Community Randomized Trial. *PLoS ONE* 2013, 8:11, e78818. PMID: 2423605

Fogel, J.M., Piwowar-Manning, E., Donohue, K., Cummings, V., Marzinke, M.A., Clarke, W., Breaud, A., Fiamma, A., Donnell, D., **Kulich, M.**, Mbwambo, J., Richter, L., Gray, G., Sweat, M., Coates, T., Eshleman, S. Determination of HIV status in African adults with discordant HIV rapid tests. *Journal of Acquired Immune Deficiency Syndromes* 2015, 69, 430-438. PMID: 25835607

Fogel, J.M., Clarke, W., **Kulich, M.**, Piwowar-Manning, E., Breaud, A., Olson, M.T., Marzinke, M.A., Laeyendecker O., Fiamma, A., Donnell, D., Mbwambo, J., Richter, L., Gray, G., Sweat, M., Coates, T.J., Eshleman, S.H. Antiretroviral drug use in a cross-sectional population survey in Africa: NIMH Project Accept (HPTN 043). *Journal of Acquired Immune Deficiency Syndromes* 2017, 74, 158-165. PMID: 27828875

For peer review only

AUGUSTINE T CHOKO

Institution: Malawi Liverpool Wellcome Trust Clinical Research Programme

General Medical Council (or equivalent) registration number N/A

Do you currently have personal medical malpractice insurance? (if so, name of insurer)

N/A

Project role

Principal Investigator

Qualifications

Degree	Year	Subject	Awarding Institution
PhD	2018	Epidemiology	LSHTM
MSc	2012	Epidemiology	LSHTM
BSc	2009	Statistics & Computing	University of Malawi

Positions held (last ten years)

Start	End	Organisation	Position title, brief description of responsibilities
**2020**	**2024**	Malawi Liverpool Wellcome Trust (MLW)	Wellcome Trust & National Institute for Health Research International Intermediate Fellow
2019	2020	MLW	Protocol Lead; leading design, implementation and write up of a complex primary health clinic randomized trial.
2015	2018	MLW	Wellcome Trust Fellow in Public Health and Tropical Medicine<br>▪ PhD student

2013	2015	MLW	Research Assistant (Epidemiology)<br>▪ Data analysis and publication
2012	2013	MLW	Trial Manager<br>▪ Leading implementation of a community-based cluster randomized trial (HIV/TB)
2009	2012	MLW	Data Manager/Statistician<br>▪ Designing and administering study databases<br>▪ Preparing data for analysis and data analysis

GCP training: course provider, date

NIDA Clinical Trials Network, Obtained 11 November 2016, expires 2019

Please list any institutions with which you are affiliated to

Malawi Liverpool Wellcome Trust Clinical Research Programme, London School of Hygiene & Tropical Medicine

Ethics training: details of training, course provider, date

Clinical trials experience: title of trial, role, dates (if multiple, restrict to most recent and most relevant)

Secondary distribution of HIV self-tests through antenatal and HIV testing services: a pragmatic cluster-randomized trial (STAR-ANC); 2018-2019.

Partner-Provided Self-Testing and Linkage (PASTAL) adaptive multi-arm multi-stage cluster randomized trial; 2016-2017.

Intensified HIV / TB prevention linking home-based HIV testing, including the option of self-testing, with HIV care: a cluster-randomised trial in Blantyre, Malaw, Research Fellow, 2011-2015.

Other professional experience relevant to role in project

Experience in handling and analyzing large epidemiological datasets.

Relevant recent publications (maximum 5)

1. **Choko AT**, Corbett EL, Stallard N, Maheswaran H, Lepine A, Johnson CC, Sakala D, Kalua T, Kumwenda M, Hayes R, Fielding K. Effect of HIV self-testing alone or with additional

For peer review only

interventions including financial incentives on linkage to care or prevention among male partners of antenatal care attendees in Malawi: An adaptive multi-arm multi-stage cluster randomised trial. *PLoS Med* 2019 Jan 2;16(1):e1002719.

2. **Choko AT**, Fielding K, Stallard N, et al. Investigating interventions to increase uptake of HIV testing and linkage into care or prevention for male partners of pregnant women in antenatal clinics in Blantyre, Malawi: study protocol for a cluster randomised trial. *Trials.* 2017;18(1):349.
3. **Choko AT**, Kumwenda MK, Johnson CC, et al. Acceptability of woman-delivered HIV self-testing to the male partner, and additional interventions: a qualitative study of antenatal care participants in Malawi. *Journal of the International AIDS Society.* 2017;20(1):21610.
4. **Choko AT**, MacPherson P, Webb EL, et al. Uptake, Accuracy, Safety, and Linkage into Care over Two Years of Promoting Annual Self-Testing for HIV in Blantyre, Malawi: A Community-Based Prospective Study. *PLoS medicine.* 2015;12(9):e1001873.
5. **Choko AT**, Desmond N, Webb EL, et al. The uptake and accuracy of oral kits for HIV self-testing in high HIV prevalence setting: a cross-sectional feasibility study in Blantyre, Malawi. *PLoS medicine.* 2011;8(10):e1001102.

For peer review only

Khumbo Phiri Nyirenda

CONTACTS
Partners in Hope
PO Box 302, Lilongwe**.**
Cell: 265882400721/ 265999 840 946
Email: khumbophiri@gmail.com

ACADEMIC QUALIFICATIONS

I. MPH, University of Malawi, College of medicine, anticipating graduation in 2020
II. BSOC, Economics, University of Malawi, Chancellor College- February, 2006
III. Malawi School Certificate of Education (MSCE) Phwezi Girls Sec School-June, 2000

COURSES/ TRAININGS

- Qualitative research synthesis, university of cape town, faculty of health sciences February 2020
- Qualitative data analysis university of cape town, faculty of health sciences January 2020
- Biomedical Researchers and Staff modules (CITI program _May 2016)
- Certificate of Attendance in Value chain analysis training by Ron Black from CNFA's farmer to farmer USAID funded program, Washington DC (February 23-27, 2009)
- Output marketing training in grain grading by CNFA/RUMARK facilitated by a North Carolina Agriculture Department officer, held at Natural Resources College (August 25 -28, 2009).
- Certificate of Attendance in a Leadership workshop facilitated by Engineers without Boarders (October 26 – 28, 2009).
- Training of trainers course in Business management and technical knowledge by COMESA's ACTESA and IFDC in Lusaka, Zambia (September 6-15, 2010)

WORK EXPERIENCE

PARTNERS IN HOPE
Position: Implementation Science Manager
Period: September 2017 to date
Summary:
The Projects Research Coordinator is responsible for overseeing and implementing all research related activities at Partners in Hope (PIH) and in all program-supported sites. He/she is in charge of monitoring and evaluating projects and ensuring that PIH is accountable to research donors. This person works hand-in-hand with the University of California in Los Angeles (UCLA), Partners in Hope (PIH), the Ministry of Health (MoH) and other partners.
Responsibilities

For peer review only

1. Overseeing and implementing all research projects at Partners in Hope and all EQUIP-supported sites

2. Research applications, Reviews and Reports.

- Oversee applications for ethical review for the Malawi NHSRC and/or COMREC, including initial and renewal applications, as well as closeout of completed projects.
- Serve as the first line of direct communication with the NHSRC and/or COMREC to advocate for submitted applications.
- Work with UCLA, PIH, MoH and other partners to ensure all research is performed to the highest ethical standards and that data is securely managed.
- Make sure appropriate reporting is provided to the governing bodies (final reports, publications, etc.).
- Oversee submission of abstracts to research meetings.

3. Monitoring, Evaluation and Accountability to Donors

- Monitor all research projects and develop donor communications in collaboration with senior leadership, especially the M&E Team.
- Ensure timely production and submission of donor experts.
- Participate in development of strategies for expansion of research.
- Ensure continuous evaluation of projects and staff, including hiring and regular appraisals.

Period: December 2012 to March 2016
Organization: Partners in Hope
Position: Research coordinator

Description

- Coordinates and administers research study associated activities. Assists in project planning and ensures that pre-established work scope, study protocol and regulatory (ethical review in Malawi and at UCLA) requirements are followed. Oversees and coordinates research staff. Develops and maintains record keeping systems and procedures. My job as Research Coordinator involves these main tasks
- Assistance developing research proposals, data collection forms, and spreadsheets for organization of data. Develops and maintains record keeping systems and procedures.

- Ensures the smooth and efficient day to day operation of research and data collection
- activities; acts as the primary administrative point of contact for EQUIP research staff.
- Supervision of team of research assistants

- Assistance with recruitment and coordination of research subjects as appropriate.

- Supervision and assistance with quality control Data.

- Monitors the progress of research activities; develops and maintains records of research activities and prepares periodic and ad hoc reports as required by investigators, administrators and funding agencies(USAID quarterly reports) and regulatory bodies (NHSRC,UCLA,IRB)

For peer review only

• Assistance with preparing ethical review applications (HSRC) including frequent communication with NHSRC about status of pending applications.

I. CARANA COOPERATION

Position: M&E/MIS Assistant
Period: November 2010–September 2011

Description
Market Linkages Initiative was a project funded by **USAID** and implemented by ACDI/VOCA and CARANA Corporation. The two key objectives of the project are to strengthen and expand grain bulking systems and to integrate farmers to national and regional markets. My job as an M&E/MIS Specialist involved these main tasks:

• Assisting the M&E specialist in tracking MLI indicators, collecting and verifying information and maintaining PMP reports, work plans and reports(weekly, monthly updates, quarterly and annual) for Malawi activities

• Administering data collection tools to GBC/VACs and capacity building of grantees to keep relevant records and generate M&E reports as stipulated by the Grant agreement.

• Collating , analyzing and reporting in usable forms all data collected form GBC/VAC

• Supporting in the coming up of GIS map for MLI supported GBCs and its associated VACs in Malawi

• Administering M&E data collection tools and supervising M&E data collectors and ensure quality data collection

• Maintaining records of all source documents from grantees and other sources including filled questionnaires and interview reports
• Keeping records of field trip reports and monitor and updating field trip tracker for Malawi based MLI staff
• Undertaking case studies and documenting most significant change stories for selected GBCs/VACs/Farmers to monitor impacts of MLI work

• Maintaining an up to date filing system including project photos

• Ensuring that quality control procedures are met in terms of market data.

• Facilitating dissemination of market information to farmers on a timely and reliable basis using the E-platform

• Providing technical assistance on the E- platform to strategic partners

• Working alongside the new company and assisting/participating in development and deployment team to design and roll out a web to phone MIS platform

For peer review only

• Conducting weekly data checks on approved prices inside E-platform's price flagging module

• Manage a user, market and commodities database

II. **CNFA/RUMARK**

Position: Monitoring and Evaluation Coordinator
Period: January 2009 to October 2010

CNFA/RUMARK implemented the Malawi Agrodealer Strengthening Program funded by AGRA. Its main objective was to develop rural-based, commercially-viable agrodealer networks and to work with agrodealers to improve the management, technical and financial capacity of their enterprises, thereby creating a rural market driven economic environment specifically designed to meet the unique needs of smallholder farmers. My position of as M&E coordinator involved the following tasks

• Monitoring progress of the project activities by doing surveys which included development of survey tools which mostly use participatory methods.

• Monitoring and evaluating the agrodealers performance in terms of sales as well as their financial status.

• Analysis of information on Agrodealer performance

• Verifying and identifying operational and potential agrodealers and recommending them for training to enable them get registered with CNFA

• Organizing promotional activities i.e. lottery competitions with the intention of creating customer database for surveys
• Playing a facilitating role in managing relationships between RUMARK and input supply companies to ensure cordial relationships and partnerships.
• Consolidating and analyzing results across CNFA's programs.

• Conducting training needs assessment for different categories of agrodealers to ensure equal treatment so that their specific needs are taken on board.

• Organizing the Agrodealers Annual Convention.

• Production of monthly as well as interim semi-annual reports for the Project

• Involved in advocating for policies which are conducive for agrodealers' business growth and sustainability through Private–public partnerships which involves working with various stakeholders including Government and civil society organizations.

Research Abstracts
Provider perspectives on barriers to reproductive health services for HIV-infected clients

For peer review only

in Central Malawi: Khumbo Phiri, Margaret R Caplan , Julie Parent , Ann Phoya , Alan Schooley, and Risa M. Hoffman, Poster presentation at Interest 2017, Malawi

Barriers to ART uptake experienced by healthy clients in Malawi under Test and Treat: Dovel, Kathryn, Khumbo Phiri, Alan Schooley, McDaphton Bellos, Esnart Sanudi, Denis Chasweka, Risa Hoffman, poster exhibition at the 9th IAS Conference on HIV Science (IAS 2017, in Paris, France, 23-26 July 2017 and Interest 2017 in Malawi).

Facility-level barriers to antiretroviral therapy experienced by men in Malawi: Dovel, Kathryn, Khumbo Phiri, Alan Schooley, Misheck Mphande, Mackenzie Chivwara, Risa Hoffman(poster presentation at interest 2017, Malawi)

Examining Malawi's Rollout of Universal Treatment: Policy Implementation and Provider Perceptions: Misheck Mphande, Khumbo Phiri, Mackenzie Chivwara, Mike Nyirenda, Alan Schooley, Rachel Thomas, Risa Hoffman, Kathryn Dovel, (Poster presentation at IAS 2017 in Paris, France)

Low rates of successful defaulter tracing and re-engagement in care in Option B+ women in Central Malawi. K. Phiri, J. Parent, T. Mulitswa, A. Schooley, R. Hoffman. Poster presentation at the International AIDS Society (IAS) conference (Durban, 2016).

The successes and Challenges of collaborating with Health Surveillance Assistants (HSAs) to trace Option B+ defaulters. Khumbo Phiri Nyirenda, Julie Parent, Risa Hoffman, Alan Schooley, Temwanani Mulitswa

The Option B+ cascade: Characterizing uptake and retention in a USAID-PEPFAR program in rural Malawi. Khumbo Phiri, Alan Schooley, Mackenzie Chivwala, Joseph Njala, Judy Currier, Andreas Jahn, Anteneh Worku, Perry Jansen, Risa Hoffman

Improvements and on-going challenges in exposed infants care at rural sites in Malawi. Alan Schooley, Khumbo Phiri, Mackenzie Chivwala, Peter Chilikoh, Antenneh worku, Risa Hoffman

Health Surveillance Assistants Can Successfully Perform Defaulter Tracing In Rural Malawi. Mackenzie Chivwala, Khumbo Phiri, Risa Hoffman, Jimmy Chitsulo, Alan Schooley

Assessing the Potential Impact of Health Surveillance Assistants on HIV Care At The Facility And Community Level. Mackenzie Chivwala, Khumbo Phiri, Weston Njamwaha, Peter Chilikoh, Risa Hoffman, Alan Schooley

Mentee Perspectives on Factors Associated with a Successful HIV Mentorship Program Mike Nyrienda, Chiulemu Kussen, Savior Mwandira, Khumbo Phiri, Chiukepo Longwe, Peter Chilikoh, Risa Hoffman, Weston Njamwaha, Alan Schooley

Rapid Rollout of Viral Load Testing at Rural Health Facilities in Malawi. Alan Schooley, Risa Hoffman, Mike Nyirenda, Savior Mwandira, Weston Njamwaha, Khumbo Phiri, Chifundo Chipungu, Mackenzie Chivwala, James Kandulu

Increased HIV testing after implementation of an innovative CD4 results reporting system in rural Malawi. Alan Schooley, Mackenzie Chivwala, Reynier Ter Haar, George Mtonga,

For peer review only

Doreen Suwande, Kelvin Rambiki, Chiulemu Kussen, John Hamilton, Khumbo Phiri, Peter Chilikoh, Risa Hoffman, Perry Jansen. Accepted for poster presentation at the 6th South African AIDS Conference, Durban, South Africa, 18-21 June 2013.

Barriers to Adherence to ART in the Prevention of Mother-to-Child Transmission of HIV: Option B+ in Nkhoma, Malawi. Paul Kawale, Alan Schooley, Virginia Tancioco, Danielle Wickman, Khumbo Phiri, Ella Bwanausi, Risa Hoffman. Accepted for poster presentation at the 6th South African AIDS Conference, Durban, South Africa, 18-21 June 2013.

MANUSCRIPTS ACCEPTED/PUBLISHED

Successes and Challenges of HIV Mentoring in Malawi: The Mentee Perspective. E. Chien, K. Phiri, A. Schooley, M. Chivwala, J. Hamilton, R. Hoffman. PLoS One. 2016 Jun;11(6).

CD4 variability in Malawian adults and implications for universal eligibility. A.L. Schooley, P.S. Kamudumuli, S. Vangala, C.H. Tseng, C. Soko, J. Parent, K. Phiri, A. Jahn, D. Namarika, R. Hoffman. Open Forum Infect Dis. 2016 Aug;3(3).

Provider perspectives on barriers to reproductive health services for HIV-infected clients in Central Malawi: Margaret R Caplan, Khumbo Phiri, Julie Parent, Ann Phoya , Alan Schooley, and Risa M. Hoffman, PLOS ONE.

Factors Associated with Retention in Option B+ in Malawi: A Case Control Study: Risa M. Hoffman, khumbo phiri, Julie parent, J Grotts D Elashoff, Paul Kawale, Sara Yeatman, J S Currier, A Schooley, JIAS.

Training Course in Focused Assessment with Sonography for HIV/TB in HIV Prevalent Medical Centers in Malawi: Timothy Canan, R Hoffman, Alan Schooley, Zachary Boas, Kristin Schwab, Daniel Kahn, Roger Shih, Khumbo Phiri, Julie Parent, Ben Allan Banda, Ronald Chagoma, Chifundo Chipungu. Kara-Lee Pool, Journal of Global Radiology

REFEREES

Risa Hoffman (MD), Assistant Clinical Professor**,** David Gaffen school of medicine, UCLA**,** RHoffman@mednet.ucla.edu

Rachel Sibande (PhD), Program Director, United Nations Foundation rsibande@unfoundation.org, +27670236497

Godfrey Chapola (PhD), Managing Director, RUMARK, P.O Box 31290, Lilongwe.

Cel: 0999792 070, gchapola@rumark.org

For peer review only

Julie Hubbard

94 Culford Road London N1 4HN
+44 7845 445338
jhubbard@mednet.ucla.edu

EDUCATION
London School of Hygiene and Tropical Medicine
MSc Control of Infectious Diseases

Seattle Pacific University
Bachelor of Arts: Sociology & Women's Studies
Cum Laude GPA: 3.74
Graduated July 2012

PROFESSIONAL EXPERIENCE
University of California Los Angeles (UCLA), March 2017- Current
Research Coordinator – 'INTERVAL' Study
Lilongwe, Malawi and Lusaka, Zambia

- Supervise data collection by study personnel across 15 health facilities in southern and central Malawi. Coordinate field supervision to ensure data quality. Work with Principle Investigator (PI) to develop operating procedures for study implementation. Provide leadership and technical support to Zambia study team.

Harvest India USA, January 2016 – March 2017
Director of Operations
Costa Mesa, California and Andhrah Pradesh, India

- Managed all aspects of operations to support, fundraise, and raise awareness for education and poverty alleviation initiatives amongst the Dalit, or 'untouchable', caste. Drafted and executed marketing campaigns to meet fundraising goals.

31 Bits International, December 2012 – February 2015
Director of Operations
Gulu, Northern Uganda

- Directed 160 beneficiaries and 6 Ugandan counselors in income generating projects. Developed and implemented in-depth monthly reports to evaluate income. Used data to identify hindrances to livelihood, such as domestic violence and HIV health complications. Organized necessary support through internal management or accessing external resources.

One Days Wages, March 2011- December 2012
Chief Grant Analyst
Seattle, Washington

- Generated extensive research on project proposals pertaining to the UN Millennium Development Goals and presented analyses for grant decisions.

Seattle Pacific University, September 2011- July 2012
Research Assistant
Seattle, Washington

- Edited, reviewed, and prepared research documents for Assistant Director of Women's Studies Program.

PUBLICATIONS AND PRESENTATIONS

For peer review only

Publications
Julie Hubbard, Gift Kakwesa, Mike Nyirenda, James Mwambene, Ashley Bardon, Kelvin Balakasi, Kathryn Dovel, Thokozani Kalua, Risa M Hoffman; Towards the third 90: improving viral load testing with a simple quality improvement program in health facilities in Malawi, International Health, , ihy083, https://doi.org/10.1093/inthealth/ihy083

Hubbard J, Moucheraud C, Lungu E, Bardon A, Balakasi K, Kakwesa G, Hoffman R ""I forget that I am a patient": A qualitative assessment of 6 month dispensing of ART" (Under review)

Dovel K, Beagley M, Hubbard J, Orombi G, Thompson K "Including men without sidelining women: the feasibility of male involvement within a women's empowerment program in northern Uganda" (Under review)

Dovel K, Hubbard J, Phiri K. "Gender and HIV services: The role of gender norms on ART initiation among men and women in Malawi." (In preparation)

Peer reviewed poster presentations
"Gender and HIV services: The role of gender norms on ART initiation among men and women in Malawi." Women in Global Health Scientific Conference. New York, New York. April 2018

"Towards the third 90: improving viral load testing with a simple quality improvement program in health facilities in Malawi" International Aids Society (IAS) Conference, Amsterdam, Netherlands. July 2018

Presentations
"Innovations in differentiated service delivery: Six-month scripting lessons from Ethiopia, Malawi and Zambia" Colombia University Mailman School of Public Health. Webinar, April 2019

CERTIFICATIONS
Confronting Gender Based Violence: Global Lessons with Case Studies from India Certification Course
Coursera (Johns Hopkins University) - Online
October 2015

- Epidemiology of gender-based violence, clinical care issues and how to provide psychosocial support for victims.

FELLOWSHIP
Mennonite Central Committee
Community Development Associate, July-August 2011

- Rural and urban poverty field study in Recife, Brazil association under the direction of the Chair of the Sociology Department at Seattle Pacific University.

HONORS
Seattle Pacific University Deans Scholar, 2008-2012

For peer review only

BROOKE E. NICHOLS, PHD, MS

OFFICE ADDRESS:

801 Massachusetts Avenue

Crosstown Center, 3rd Floor, Room 304

Boston, MA, USA 02118

+1 617 358 2403

Email: brooken@bu.edu

ACADEMIC TRAINING:

2015	Ph.D.	Department of Viroscience, Erasmus Medical Center (Rotterdam, the Netherlands)<br>*Mathematical Modeling and Cost-Effectiveness of Antiretroviral-Based HIV-1 Prevention Strategies.*
2011	M.S.	School of Public Health & Health Science, University of Massachusetts, Amherst<br>(Amherst, MA, USA), Epidemiology
2009	B.A.	Mount Holyoke College (South Hadley, MA, USA), International Relations, *cum laude*

RESEARCH APPOINTMENTS:

2019 – Present	Assistant Professor	Department of Global Health, School of Public Health,<br>Boston University, Boston, MA
2018 – 2019	Instructor	Department of Global Health, School of Public Health,<br>Boston University, Boston, MA
2017 – 2018	Research Scientist	Department of Global Health, School of Public Health,

For peer review only

		Boston University, Boston, MA
2017 – Present	Principal Researcher	Health Economics & Epidemiology Research Office, Wits
		Health Consortium, Faculty of Health Sciences, University of
		Witwatersrand, Johannesburg, South Africa
2017 – Present	Researcher	Joint Faculty Appointment, School of Clinical Medicine,
		Faculty of Health Sciences, University of the Witwatersrand,
		Johannesburg, South Africa
2015 – 2016	Postdoctoral Fellow	Department of Viroscience, Erasmus Medical Center,
		Rotterdam, the Netherlands
2009 – 2010	Research Assistant	University of Massachusetts Amherst, School of Public Health
		& Health Sciences, Amherst, MA

OTHER RESEARCH EXPERIENCE:

2012 – 2014	Epidemiologist	Médicins Sans Frontières
		Amsterdam, the Netherlands.
		Project: *Spinal cord injury outcomes in Sri Lanka*
2008 – 2009	Researcher	Ministry of Health and Social Services, Lüderitz, Namibia
		Project: *Ecologic study on alcohol establishments and HIV prevalence*

PROFESSIONAL APPOINTMENTS:

2008 – 2010	Research Associate: Epidemiology	Environ Corporation, Amherst, MA, USA.

AWARDS AND HONOURS:

2019 **Mary Lyon Award.** Mount Holyoke College Alumni Association. Award honors an alumna who

For peer review only

has demonstrated sustained achievement in her life and career consistent with the humane values

that Mary Lyon exemplified and inspired in others.

CONFERENCE ORAL PRESENTATIONS:

*Denotes graduate student or mentee

1. Popping S*, Kall M, Stempher E, Versteegh L, **Nichols B**, van Sighem A, van de Vijver D, Boucher C, Verbon A, Delpech V. Country specific factors determine the quality of life among people with HIV in two western European countries.: 4th European Workshop on Health Living with HIV, Barcelona, Spain, September 2019.

2. Dovel K, Balakasi K, Shaba F, Offorjebe O, Gupta S, Wong S, Phiri K, Lungu E, Nyirenda M, **Nichols B**, Ngona K, Hoffman R. A randomized trial on index HIV self-testing for partners of ART clients in Malawi. Conference on Retroviruses and Opportunistic Infections (CROI), Seattle, USA, March 2019.

3. **Nichols BE**, Girdwood SJ*, Crompton T, Stewart-Isherwood L, Berrie L, Chimhamhiwa D, Moyo C, Kuehnle J, Rosen S. Monitoring viral load for the last mile: what will it cost? AIDS, Amsterdam, Netherlands, July 2018.

4. Girdwood SJ*, **Nichols BE**, Moyo C, Crompton T, Chimhamhiwa D, Rosen S. Optimizing access for the last mile: Geospatial cost model for point of care viral load instrument placement in Zambia. AIDS, Amsterdam, Netherlands, July 2018.

5. Dovel K, Nyirenda M, Shaba F, Offorjebe OA, Balakasi K, **Nichols BE**, Phiri K, Schooley A, Hoffman RM. Facility-based HIV self-testing for outpatients dramatically increases HIV testing in Malawi: a cluster randomized trial. AIDS, Amsterdam, Netherlands, July 2018.

6. **Nichols BE**, Hendrickson C, Sigwebela N, Moyo C, Fox MP, Rosen S. Prioritizing healthcare facilities for on-site mentorship to increase HIV treatment uptake: results from EQUIP. International AIDS Economics Network (IAEN) Conference, Amsterdam, Netherlands, July 2018.

7. van de Vijver DA, Richter A-K, Boucher CA, Gunsenheimer-Bartmeyer B, Kollan C, **Nichols BE**, Spinner C, Wasem J, Schewe K, Neumann A. Cost-effectiveness of pre-exposure prophylaxis in Germany (Kosteneffektivität der HIV-Präexpositionsprophylaxe in Deutschland). DGGÖ (German Society for health economics) Annual Meeting, Hamburg, Germay, March 2018.

8. van de Vijver DA, Richter A-K, Boucher CA, Gunsenheimer-Bartmeyer B, Kollan C, **Nichols BE**, Spinner C, Wasem J, Schewe K, Neumann A. Cost-effectiveness of pre-exposure prophylaxis for HIV-1 prevention in Germany. European AIDS Conference (EACS), Milan, Italy, October 2017.

9. Smit M, van Zoest RA, **Nichols BE**, Vaartjes I, Smit C, van der Valk M, van Sighem A, Wit FW, Hallett TB, Reiss P. Cardiovascular prevention policy in HIV: recommendations from a modeling study. Conference on Retroviruses and Opportunistic Infections (CROI), Seattle, WA. February 2017.

10. Popping S*, **Nichols BE**, van Kampen JJA, Verbon A, Boucher CAB, van de Vijver DA. Intensive hepatitis C monitoring in previously HCV infected HIV-positive MSM is a cost saving method to

reduce the HCV epidemic. Netherlands Conference on HIV Pathogenesis, Epidemiology, Prevention and Treatment (NCHIV), Amsterdam, the Netherlands, November 2016.

11. **Nichols BE**, Boucher CAB, van der Valk M, Rijnders BJA, van de Vijver DA. PrEP is Only Cost-Effective Among MSM in the Netherlands When Used on Demand. Conference on Retroviruses and Opportunistic Infections (CROI), Boston, MA. February 2016.

12. **Nichols BE**, Boucher CAB, van der Valk M, Rijnders BJA, van de Vijver DA. On demand PrEP among MSM in the Netherlands: a cost-effective approach for preventing HIV-1 infections. Netherlands Conference on HIV

Peer reviewed publications:

*Authors contributed equally

**Denotes graduate student or mentee

1. Dovel K, Nyirenda M, Shaba F, Offorjebe OA, Balakaksi K, **Nichols BE**, Cele R. Phiri K, Wong V, Gupta S, Hoffman RM. Effect of facility-based HIV self-testing on uptake of testing among adult outpatients in Malawi: a cluster-randomized trial. *The Lancet Global Health. In press.*

2. van Vliet MM**, Hendrickson C**, **Nichols BE**, Boucher CAB, Peters RPH, Polis CB, van de Vijver DAMC. Epidemiological impact and cost-effectiveness of long-acting pre-exposure prophylaxis combined with injectable contraceptives for HIV prevention in South Africa: a modelling study. *JIAS*. 2019, 22:e25427.

3. Long, L., Kuchukhidze, S., Pascoe, S., **Nichols, B.,** Cele R., Govathson, C., Flynn, D., Rosen, S. Differentiated Models of Service Delivery for Antiretroviral Treatment of HIV in sub-Saharan Africa: A Rapid Review Protocol. *Systematic Reviews*. 2019, 8:314.

4. Masuku S**, Berhanu R, van Rensburg C, Ndjeka N, Rosen S, Long L, Evans D, **Nichols BE**. The costs of managing multi drug-resistant tuberculosis in South Africa: an economic evaluation of moving to a short-course treatment regimen containing bedaquiline. *International Journal of Tuberculosis and Lung Disease. In press.*

5. Hendrickson C*,**, Long L*, van de Vijver DA, Boucher CA, O'Bra H, Claassen CW, Njelesani M, Moyo C, Mumba DB, Subedar H, Mulenga L, Rosen S, **Nichols BE**. Novel metric for evaluating PrEP program effectiveness in real-world settings. *Lancet HIV. In press.*

6. Girdwood SJ**, **Nichols BE,** Moyo C, Crompton T, Chimhamhiwa D, Rosen S. Optimizing access for the last mile: Geospatial cost model for point of care viral load instrument placement. *PLoS ONE*. 14(8):e0221586.

7. **Nichols BE**, Girdwood SJ**, Crompton T, Stewart-Isherwood L, Berrie L, Chimhamhiwa D, Moyo C, Kuehnle J, Stevens W, Rosen S. Monitoring viral load for the last mile: what will it cost? *JIAS*. 2019, 22:e25337.

8. Popping S**, **Nichols BE**, van Kampen JJA, Verbon A, Boucher CAB, van de Vijver DA. Targeted HCV core antigen monitoring among HIV-positive men-who-have-sex-with-men is cost-saving. *Journal of Virus Eradication*. 2019; 5:179-190.

For peer review only

9. **Nichols BE**, Girdwood SJ**, Shibemba A, Sikota S, Gill C, Mwananyanda L, Scott L, Noble L, Carmona S, Rosen S, Stevens W. Cost and impact of dried blood spot versus plasma separation card for viral load testing in resource limited settings. *Clinical Infectious Diseases.* Advance article: 10.1093/cid/ciz338.

10. van de Vijver DA, Richter A-K, Boucher CA, Gusenheimer-Bartmeyer B, Kollan C, **Nichols BE**, Spinner CD, Wasem J, Schewe K, Neumann A. Cost-effectiveness and budget impact of generic pre-exposure prophylaxis for HIV-1 prevention in Germany. *Eurosurveillance.* 2019 Feb; 24(7).

11. Popping S**, Hulligie SJ, Boerekamps A, Rijnders BJA, de Knegt RJ, Rockstroh JK, Verbon A, Boucher CAB, **Nichols BE**, van de Vijver DA. Early treatment of acute HCV infection is cost-effective in HIV-infected men-who-have-sex-with-men. *PLoS One*, 2019. 14(1):e0210179.

12. **Nichols BE**, Girdwood SJ**, Crompton T, Stewart-Isherwood L, Berrie L, Chimhamhiwa D, Moyo C, Kuehnle J, Stevens W, Rosen S. Impact of a borderless sample transport network for scaling up viral load monitoring: results of a geospatial optimization model for Zambia. *JIAS.* 2018, 21:e25206.

13. Dovel K, Shaba F, Nyirenda M, Ogechukwu AO, Balakasi K, Phiri K, **Nichols BE**, Tseng C-H, Bardon A, Namachapa KN, Hoffman RM. Evaluating the integration of HIV self-testing into low-resource health systems: a study protocol for a cluster randomized control trial from EQUIP Innovations. *Trials,* 2018. 19:498.

14. Phillips AN, Cambiano V, Nakagawa F, Revill P, Jordan MR, Hallett TB, Doherty M, De Luca A, Lundgren JD, Mhangara M, Apollo T, Mellors J, **Nichols B**, Parikh U, Pillay D, Rinke de Wit T, Sigaloff K, Havlir D, Kuritzkes DR, Pozniak A, van de Vijver D, Vitoria M, Wainberg MA, Raizes E, Bertagnolio S, Working Group on Modelling Potential Responses to High Levels of Pre-ART Drug Resistance in Sub-Saharan Africa. Cost-effectiveness of public-health policy options in the presence of pretreatment NNRTI drug resistance in sub-Saharan: a modelling study. *Lancet HIV*, 2018. 5(3):e146-e154.

15. Smit M, van Zoest RA, **Nichols BE**, Vaartjes I, Smit C, van der Valk M, van Sighem A, Wit FW, Hallett TB, Reiss P; Netherlands ATHENA observational HIV cohort. Cardiovascular disease prevention policy in HIV: recommendations from a modelling study. *Clinical Infectious Diseases*, 2018. 66(5):743-750.

16. Luiken GPM, Joore IK, Taselaar A, Schuit SCE, Geerlings SE, Govers A, Rood PPM, Prins JM, **Nichols BE**, Verbon A, de Vries-Sluijs TEMS. Non-targeted HIV screening in emergency departments in the Netherlands. *The Netherlands Journal of Medicine*, 2017. 75(9):386-393.

17. **Nichols BE**, Boucher CAB, van der Valk M, Rijnders BJA, van de Vijver DAMC. Cost-effectiveness analysis of pre-exposure prophylaxis for HIV-1 prevention in the Netherlands: a mathematical modelling study. *Lancet Infectious Diseases*, 2016. 16(12):1423-1429.

18. **Working Group on Modelling of ART Monitoring Strategies in Sub-Saharan Africa**. Sustainable HIV Treatment in Africa through Viral Load-Informed Differentiated Care. *Nature*, 2015. 528(7580):S68-76.

19. **Nichols BE**, Gotz HM, van Gorp ECM, Verbon A, Rokx C, Boucher CAB, van de Vijver DAMC. Partner notification for reduction of HIV-1 transmission and related costs among men who have sex with men: a mathematical modeling study. *PLoS One,* 2015. 10(11):e0142576.

For peer review only

20. **Nichols BE**, Sigaloff KC, Kityo C, Hamers RL, Baltussen R, Bertagnolio S, Jordan MR, Hallett TB, Boucher CA, Rinke de Wit TF, van de Vijver DA. Increasing the use of second-line therapy is a cost-effective approach to prevent the spread of drug-resistant HIV: a mathematical modelling study. *J Int AIDS Soc*, 2014. 17:19164.

21. **Nichols BE**, Baltussen R, van Dijk JH, Thuma PE, Nouwen JL, Boucher CA, van de Vijver DA. Cost-effectiveness of PrEP in HIV/AIDS control in Zambia: a stochastic league approach. *JAIDS*, 2014. 66(2):221-8.

22. Eaton JW, Menzies NA, Stover J, Cambiano V, Chindelevitch L, Cori A, Hontelez JAC, Humair S, Kerr CC, Klein DJ, Mishra S, Mitchell KM, **Nichols BE**, Vickerman P, Bakker R, Barnighausen T… Hallett TB. How should HIV programmes respond to evidence for the benefit of earlier treatment initiation? A combined analysis of twelve mathematical models. *Lancet Global Health*, 2014. 2:e23-34.

23. Armstrong JC*, **Nichols BE***, Wilson JM, Cosico RA, Shanks L. Spinal cord injury in the emergency context: review of program outcomes of a spinal cord injury rehabilitation program in Sri Lanka. *Conflict and Health*, 2014. 8(1):4.

24. **Nichols BE,** Sigaloff KC, Kityo C, Mandaliya K, Hamers RL, Bertagnolio S, Jordan MR, Boucher CA, Rinke de Wit TF, van de Vijver DA. Averted HIV infections due to expanded antiretroviral treatment eligibility offsets risk of transmitted drug resistance: A modeling study. *AIDS*, 2014. 28(1):73-83.

25. van de Vijver DA, **Nichols BE,** Abbas UL, Boucher CA, Cambiano V, Eaton JW, Glaubius R, Lythgoe K, Mellors J, Phillips A, Sigaloff KC, Hallett TB. Pre-Exposure Prophylaxis will have a limited impact on HIV-1 drug resistance in sub-Saharan Africa: A comparison of mathematical models. *AIDS*, 2013. 27(18):2943-2951.

For peer review only

Alemayehu Amberbir

POSITION TITLE: Epidemiologist
eRA Commons User Name: A.AMBERBIR_DI

EDUCATION/TRAINING

INSTITUTION AND LOCATION	DEGREE *(if applicable)*	YEAR(s)	FIELD OF STUDY
Haramaya University, Ethiopia	BSc	2004	Health Officer
Jimma University, Ethiopia	MPH	2007	Public Health
University of Nottingham, UK	PhD	2012	Epidemiology

Positions and Honors

Sept 2003 – Jun 2004 Intern (Clinical/Public Health), Haramaya University & Hiwot Fana Hospital, Ethiopia Oct 2004 – Sept 2005 HIV/AIDS Prevention and Care Program Officer, Menschen Für Menschen, Ethiopia
Aug 2007 - Mar 2008 Lecturer, Department of Epidemiology and Biostatistics, Jimma University, Ethiopia
Feb 2008 – Mar 2011 Honorary Lecturer, Addis Ababa University, Ethiopia
Feb 2008 – Feb 2012 Research Fellow, University of Nottingham, UK
Mar 2012 – Sep 2013 Research Fellow, London School of Hygiene and Tropical Medicine, UK
Oct 2013 – Jan 2016 Lecturer, London School of Hygiene and Tropical Medicine, UK
Jan 2016 – Jun 2019 Epidemiologist, Dignitas International
Jan 2018 – Jun 2019 Adjunct Lecturer, Dalla Lana School of Public Health, University of Toronto
Jan 2018 – Dec 2019 Postdoctoral Fellow; CIHR Canadian HIV Trials Network (CTN), Canada
Aug 2019 – present Science Director, University of California Los Angles; David Geffen School of Medicine

Contribution to Science

Investigating non-communicable diseases (hypertension, diabetes and asthma) in Africa (selected)

1. Soares ALG, Banda L, **Amberbir A**, Jaffar S, Musicha C, Price A, Nyirenda MJ, Lawlor DA, Crampin A. Sex and area differences in the association between adiposity and lipid profile in Malawi. *BMJ Glob Health*. 2019 Sep 11;4(5):e001542. doi: 10.1136/bmjgh-2019-001542.

2. **Amberbir A.** The challenge of worldwide tuberculosis control: and then came diabetes. Lancet GlobHealth. 2019 Apr;7(4):e390-e391. doi: 10.1016/S2214-109X(19)30053-1.

3. **Amberbir A**, S Lin, J Berman, et al A Muula, D Jacoby, E Wroe et al. Systematic review of hypertension and diabetes burden and risk factors and interventions for prevention and control in

For peer review only - http://bmjopen.bmj.com/site/about/guidelines.xhtml

Malawi: The NCD BRITE Consortium. Glob Heart. 2019 Jun;14(2):149-154. doi: 10.1016/j.gheart.2019.05.004. Review.

4. **Amberbir A**, Banda V, Singano V, Matengeni A, Pfaff C, Ismail Z, Allain TJ, Chan AK, Sodhi SK, van Oosterhout JJ. Effect of cardio-metabolic risk factors on all-cause mortality among HIV patients on antiretroviral therapy in Malawi: A prospective cohort study. ***PLoS ONE***. 2019 Jan 17;14(1):e0210629. doi: 10.1371/journal.pone.0210629. PMID: 30653539

5. **Amberbir A**, Singano V, Matengeni A, Ismail Z, Kawalazira G, Chan AK, et al. Dyslipidemia among rural and urban HIV patients in south-east Malawi. PLoS ONE 2018; 13(5): e0197728. https://doi.org/10.1371/journal.pone.0197728

6. Price AJ, Crampin AC, **Amberbir A**, et al. Prevalence of obesity, hypertension and diabetes, and cascade of care in sub-Saharan Africa: a cross-sectional population-based study in rural and urban Malawi. ***Lancet Diabetes Endocrinol 2018***; published online Jan 19. http://dx.doi.org/10.1016/S2213-8587 (17)30432-1.

7. *The GBD 2015 Obesity Collaborators et al **[inc Amberbir A].** Health Effects of Overweight and Obesity in 195 Countries over 25 Years. *N Engl J Med 2017*;377(1):13-27.

8. Divala OH, **Amberbir A**, Ismail Z, Beyene T, Garone D, Pfaff C, Singano V, Akello H, Joshua M, Nyirenda MJ, Matengeni A, Berman J, Mallewa J, Chinomba GS, Kayange N, Allain TJ, Chan AK, Sodhi SK, van Oosterhout JJ. The burden of hypertension, diabetes mellitus, and cardiovascular risk factors among adult Malawians in HIV care: consequences. *BMC Public Health*. 2016 Dec 12;16(1):1243. PMID:27955664

9. Crampin AC, Kayuni N, **Amberbir A**, Musicha C, Koole O, Tafatatha T, Branson K, Saul J, Mwaiyeghele E, Nkhwazi L, Phiri A, Price AJ, Mwagomba B, Mwansambo C, Jaffar S, Nyirenda MJ. Hypertension and Diabetes in Africa: Design and implementation of a large population-based study of burden and risk factors in rural and urban Malawi. *Emerging Themes in Epidemiology*. 2016, 13:3. DOI: 10.1186/s12982-015-0039-2

10. Jaffar S, **Amberbir A**, Kayuni N, Musicha C, Nyirenda M. Viewpoint: scaling up testing services for noncommunicable diseases in Africa: priorities for implementation research. Trop Med Int Health. 2013Nov;18(11):1353-6. doi: 10.1111/tmi.12180. Epub 2013 Sep

11. **Amberbir A**, Medhin G, Hanlon C, Britton J, Davey G, Venn A. Effects of early life paracetamol use on the incidence of allergic disease and sensitization: 5 year follow-up of an Ethiopian birth cohort. *PLoS ONE*. **2014** Apr 9;9(4):e93869. doi: 10.1371/journal.pone.0093869.

12. **Amberbir A**, Medhin G, Abegaz WE, Hanlon C, Robinson K, Fogarty A, Britton J, Venn A, Davey G. Exposure to Helicobacter pylori infection in early childhood and the risk of allergic disease and atopic sensitization: a longitudinal birth cohort study. Clin Exp Allergy. 2014 Apr;44(4):563-71. doi:

13. **Amberbir A**, Medhin G, Erku W, Alem A, Simms R, Robinson K, Fogarty A, Britton J, Venn A, Davey G. Effects of Helicobacter pylori, geohelminth infection and selected commensal bacteria on the risk of allergic disease and sensitization in 3-year-old Ethiopian children. *Clin Exp Allergy*. **2011** Oct;41(10):1422-30. doi: 10.1111/j.1365-2222.2011.03831.x. Epub 2011 Aug 10.

For peer review only

14. **Amberbir A**, Medhin G, Alem A, Britton J, Davey G, Venn A. The role of acetaminophen and geohelminth infection on the incidence of wheeze and eczema: a longitudinal birth-cohort study. *Am J Respir Crit Care Med*. **2011** Jan 15;183(2):165-70. doi: 10.1164/rccm.201006-0989OC. Epub 2010 Oct 8.

HIV risk behaviours and status disclosure in African settings (selected)

1) Dessalegn NG, Hailemichael RG, Shewa-Amare A, Sawleshwarkar S, Lodebo B, **Amberbir A**, Hillman RJ. HIV Disclosure: HIV-positive status disclosure to sexual partners among individuals receiving HIV care in Addis Ababa, Ethiopia. PLoS One**. 2019** Feb 15;14(2):e0211967. doi: 10.1371/journal.pone.0211967. eCollection 2019.

2) Deribe K, Woldemichael K, Wondafrash M, Haile A, **Amberbir A**. Disclosure experience and associated factors among HIV positive men and women clinical service users in Southwest Ethiopia. *BMC Public Health*. 2008 Feb 29;8:81. doi: 10.1186/1471-2458-8-81.

3) Biadgilign S, Deribew A, **Amberbir A**, Escudero HR, Deribe K. Factors associated with HIV/AIDS diagnostic disclosure to HIV infected children receiving HAART: a multi-center study in Addis Ababa, Ethiopia. *PLoS ONE*. 2011 Mar 21;6(3):e17572. doi: 10.1371/journal.pone.0017572.

Health system research in to infectious diseases – HIV – in Africa (selected)

1. Alhaj M[1,2¶],**Amberbir A**[1¶], Singogo E, Banda V, van Lettow M, Matengeni A, Kawalazira G, Theu J, Jagriti MR, Chan AK, van Oosterhout JJ. Retention on antiretroviral therapy during universal test and treat implementation in Zomba district, Malawi: a retrospective cohort study. J Int AIDS Soc. 2019 Feb;22(2):e25239. doi: 10.1002/jia2.25239.

2. Singano V[¶], **Amberbir A**[¶], Garone D, Kandionamaso C, Msonko J; van Lettow M, Kalima K, Mataka, Kawalazira G, Mateyu G, Kwekwesa A, Matengeni A; van Oosterhout JJ. The burden of gynecomastia among men on Antiretroviral Therapy in Zomba, Malawi. *PLoS ONE 12(11): e0188379: doi.org/10.1371/journal.pone.0188379*

3. Mpawa H, Kwekwesa A, **Amberbir A**, Garone D, Divala OH, Kawalazira G, van Schoor V , Ndindi H, van Oosterhout JJ. Virological outcomes of Antiretroviral Therapy in Zomba Central Prison, Malawi: *J Int AIDS Soc* 2017, 20:21623

4. **Amberbir A**, Woldemichael K, Getachew S, Girma B, Deribe K. Predictors of adherence to antiretroviral therapy among HIV-infected persons: a prospective study in Southwest Ethiopia. *BMC Public Health***. 2008** Jul 30;8:265. doi: 10.1186/1471-2458-8-265.

5. Biadgilign S, Deribew A, **Amberbir A**, Deribe K. Barriers and facilitators to antiretroviral medication adherence among HIV-infected paediatric patients in Ethiopia: A qualitative study. *SAHARA J***.** 2009 Dec;6(4):148-54.

For peer review only

6. Deribe K, Hailekiros F, Biadgilign S, **Amberbir A**, Beyene BK. Defaulters from antiretroviral treatment in Jimma University Specialized Hospital, Southwest Ethiopia. *Trop Med Int Health*. 2008 Mar;13(3):328-33. doi: 10.1111/j.1365-3156.2008.02006.x. Epub 2008 Feb 21.

For peer review only

LAWRENCE C. LONG, PHD, MCOM

OFFICE ADDRESS: 801 Massachusetts Avenue, Crosstown Center, 3rd Floor, Room 369
Boston, MA, 02119, USA.
+1 (617) 358-3122
Email: lclong@bu.edu

ACADEMIC TRAINING:

2016	Ph.D.	School of Public Health, University of Witwatersrand (Johannesburg, South Africa)
2009	M.Com.	School of Economic and Business Sciences, University of Witwatersrand (Johannesburg, South Africa), Economics, *cum laude*
2001	B.Bus.Sci.	School of Economics, University of Cape Town (Cape Town, South Africa), Economics and Finance

ACADEMIC LEADERSHIP:

2011 - 2017	Deputy Division Head	Health Economics & Epidemiology Research Office<br>Wits Health Consortium<br>Faculty of Health Sciences, University of Witwatersrand
2008 – 2011	Research Coordinator	Health Economics & Epidemiology Research Office<br>Wits Health Consortium<br>Faculty of Health Sciences, University of Witwatersrand

RESEARCH APPOINTMENTS:

2017 – Present	Research Assistant Professor	Department of Global Health, School of Public Health,<br>Boston University
2008 – Present	Associate Researcher	Joint Faculty Appointment, School of Clinical Medicine,<br>Faculty of Health Sciences, University of the Witwatersrand
2008 – Present	Principal Researcher (Visiting)	Health Economics & Epidemiology Research Office<br>Wits Health Consortium<br>Faculty of Health Sciences, University of

For peer review only

2006 – 2007	Research Associate	Witwatersrand<br>Health Economics & Epidemiology Research Office<br>Wits Health Consortium<br>Faculty of Health Sciences, University of Witwatersrand
2005	Study Coordinator	Health Economics & Epidemiology Research Office<br>Wits Health Consortium<br>Faculty of Health Sciences, University of Witwatersrand

OTHER RESEARCH EXPERIENCE:

2007 – 2008	Consultant	Center for International Health and Development,<br>Boston University School of Public Health<br>Project: *Cost and Outcomes of Models for Delivering Antiretroviral Therapy for HIV/AIDS in Zambia*
2007 – 2008	Consultant	Center for International Health and Development,<br>Boston University School of Public Health<br>Project: *Cost and Outcomes of Models for Delivering Antiretroviral Therapy for HIV/AIDS in Kenya*

TEACHING EXPERIENCE:

2019	**Structural factors affecting HIV – an economists perspective.** Audience: MPH Students. Guest Lecture in Individual, Community, and Population Health SPH GH 720 Boston University, Boston, USA. Presented in two classes (Profs Monica Onyango & Jennifer Schlezinger).
2019	**Economic evaluation.** Audience: MPH Students. Guest Lecture in Monitoring and Evaluation of Global Health Programs SPH GH 745, Boston University, Boston, USA.
2019	**Using economics to influence policy.** Audience: MPH Students. Guest Lecture in Essential of Economics and Finance for Global Health SPH GH 762, Boston University, Boston, USA.
2017	**From cost to clinic – Economics changing health policy.** Audience: MPH Students. Guest Lecture in Essentials of Economics and Finance for Global Health SPH GH 762, Boston University, Boston, USA.
2016	**Data collection and analysis for economic evaluations.** Audience: Technical implementing partners. EQUIP Partners Meeting, Johannesburg, South Africa.

For peer review only

2016 **From patient to policy – Ensuring that your clinical practice is positioned to inform evidenced based policy.** Audience: HIV Clinicians. Chair of Research Skills Building Session, Southern African HIV Clinicians Society Conference, Johannesburg, South Africa.

2014 **Introduction to Health Programme Evaluation**. Audience: MSc students – Module of Epidemiology for Health Researchers II. University of Witwatersrand, Johannesburg.

INVITED PRESENTATION:

2019 **Costs and resources needed to provide HIV services to key populations over the next 10 years.** Idea creation meeting – Defining and addressing HIV treatment and prevention needs of underserved and high-risk populations. BMGF & Journal of International AIDS Society, New York, USA.

2019 **Direct action to achieve a result.** Spotlight on "Think, Teach, Do", School of Public Health, Boston University, Boston, USA.

2019 **Learning Community – Take home.** Academy for Faculty Advancement, School of Medicine, Boston University, Boston, USA.

2018 **Urban public health issues – the transition to Boston.** Health Economics and Epidemiology Research Office, Johannesburg, South Africa.

2017 **Partner's Area of Expertise – Health Economics.** USAID South Africa, Partners Meeting, Pretoria, South Africa. Presented in absentia by Denise Evans.

2017 **Innovations Research on AIDS (INROADS).** Director Doug Arbuckle, Office HIV AIDS (OHA, USA). USAID, Johannesburg, South Africa. Presented in absentia by Denise Evans.

2016 **Test and Start – Research supporting evidence based policy change.** Zambian Department of Health & USAID, EQUIP Project, Johannesburg, South Africa.

2016 **Initiating ART at a patients first clinic visit: the RapIT randomised trial.** Faculty of Health Sciences Research Day, University of Witwatersrand, Johannesburg, South Africa.

CONFERENCE ORAL PRESENTATIONS:

1. Van Rensburg C, Berhanu R, Hirasen K, Evans D, Rosen S, **Long L**. Cost outcome analysis of decentralised care for drug-resistant tuberculosis in Johannesburg, South Africa. 49th Union World Conference on Lung Health, 24-27 October, The Hague, The Netherlands. 2018.

For peer review only

2. Evans D, Musakwa N, Nattey C, Bor J, Lonnermark E, Larshans C, Andreasson S, Nyasulu P, **Long L**. Barriers to accessing care for HIV and Tuberculosis among adolescents and young adults aged 18 – 25 years in Johannesburg, South Africa. 5th SA TB Conference, 12-15 June, Durban, South Africa. 2018.

3. Lince-Deroche N, Leuner R, **Long L**. When Donor Funding Leaves: The immediate impact on resources of withdrawal of support for direct HIV care and treatment at public health facilities in Johannesburg. 9th International AIDS Society (IAS) Conference, 23-26 July, Paris, France. 2017.

4. Musakwa N, Evans D, Feeley A, Magwete M, Patz S, McNamara L, **Long L**, Sanne I. Acute malnutrition and dietary intake among paediatric HIV-positive patients initiating antiretroviral therapy in Johannesburg, South Africa. 8th SA AIDS Conference, 13-15 June, Durban, South Africa. 2017.

5. Rosen S, Maskew M, Fox MP, Nyoni C, Mongwenyana C, Malete G, Sanne I, Bokaba D, Sauls C, Rohr J, **Long L**. Initiating ART at a Patient's First Clinic Visit: The RapIT Randomized Trial. Conference on Retroviruses and Opportunistic Infections (CROI) 2016, 22-25 February, Boston, USA.

6. Hirasen K, Berhanu R, Schnippel K, **Long L**, Rosen S, Sanne I. Twelve-month outcomes of patients initiating drug-resistant tuberculosis treatment at a decentralized outpatient clinic in Johannesburg, South Africa. 46th World Conference on Lung Health of the International Union Against Tuberculosis and Lung Disease (The Union), Cape Town, South Africa, 2-6 December 2015.

7. Evans D, Schnippel K, Budgell E, Shearer K, Berhanu R, **Long L**, Rosen S. Predictors of mortality in patients diagnosed with preXDR- and XDR-TB: results from the South African National TB Programme, 2009-2010. 46th World Conference on Lung Health of the International Union Against Tuberculosis and Lung Disease (The Union), Cape Town, South Africa, 2-6 December 2015.

BIBLIOGRAPHY:

ORCID ID: 0000-0003-4986-4988

Google Scholar: https://scholar.google.co.za/citations?user=fao8zDgAAAAJ&hl=en&oi=ao

Peer reviewed publications:

1. **Long L**, Kuchukhidze S, Pascoe S, Nichols B, Cele R, Govathson C, Huber A, Flynn D, Rosen S. Differentiated models of service delivery for antiretroviral treatment of HIV in sub-Saharan Africa: a rapid review protocol. *Systematic Reviews*. DOI: 10.1186/s13643-019-1210-6. 2019.

For peer review only

2. Jamieson L, Evans D, Berhanu R, Ismail N, Aucock S, Wallengren K, **Long L**. Data quality of drug-resistant tuberculosis and antiretroviral therapy electronic registers in South Africa. *BMC Public Health*. DOI: 10.1186/s12889-019-7965-9. 2019.

3. Mokhele I, Mashamaite S, Majuba P, Xulu T, **Long L**, Onoya D. Effective public-private partnerships for sustainable antiretroviral therapy: outcomes of the Right to Care health services GP down-referral program. *BMC Public Health*. DOI:
10.1186/s12889-019-7660-x. 2019.

4. Lince-Deroche N, Leuner R, Meyer-Rath G, Pillay Y, **Long L**. When Donor Funding Leaves: A retrospective review of the impact of integrating direct HIV care and treatment into public health services in a region of Johannesburg. *Cost Effectiveness and Resource Allocation*. DOI: 10.1186/s12962-019-0192-5. 2019.

5. Musakwa N, Feeley A, Magwete M, Patz S, McNamara L, Sanne I, **Long L**, Evans D. Dietary intake among paediatric HIV-positive patients initiating antiretroviral therapy in Johannesburg, South Africa. *Vulnerable Children and Youth Studies (RVCH)*. DOI: 10.1080/17450128.2019.166858. 2019.

6. Brennan A, Bonawitz R, Gill CJ, Thea DM, Kleinman M, **Long L**, McCallum C, Fox MP. A Meta-analysis Assessing Diarrhea and Pneumonia in HIV-Exposed Uninfected Compared With HIV-Unexposed Uninfected Infants and Children. *Journal of Acquired Immune Deficiency Syndrome (JAIDS)*. DOI: 10.1097/QAI.0000000000002097. 2019

7. Berry KM, Rodriguez CA, Berhanu R, Ismail N, Rosen S, **Long L**, Evans D. Treatment outcomes among pediatrics, adolescents, and adults on treatment for drug-sensitive TB in two metropolitan municipalities in Gauteng Province, South Africa. *BMC Public Health*. DOI: 10.1186/s12889-019-7257-4. 2019.

For peer review only

IN-DEPTH INTERVIEW FOR MEN – MIDLINE

Section 1: Pre-Trial Experiences

1. At some point in the past, you stopped taking ARV's (or didn't start ART), which is why you were recruited into this study. Can you walk me through some of the reasons that it was difficult to stay on (or start) ART in the past?

Section 2: Trial Experience

1. As a part of your participation in this study, you may have been visited a couple of times by different people. Did anything **bad** happen as a result of these visits?
2. Did someone discover your HIV status as a result of the study or as a result of taking ART since you enrolled?
3. How many times have you met with a health care worker (Patient Supporter/Nurse) about HIV (excluding ART clinic visits)?
    1. *If yes:* Where did you meet this person?
4. When this health care workers (Patient Supporter/Nurse) met with you, you may have gone through this Men's Counseling Flip Chart [SHOW THE FLIP CHART].
    1. Have you ever seen this flip chart?
        i. IF YES: How was the counseling from this flip chart different from the counseling you have received in the past (before joining the study)?
        ii. IF YES: What topic/idea from the flip chart was the most helpful to you?
        iii. IF YES: You described to me earlier that you have missed ART in the past because of [X] reason. Did the new counseling with this flip chart help you deal with [X]? How?
        iv. IF NO: When you met with the health care worker, can you briefly describe what you discussed?
5. As a part of the study. you may have been offered to be given ART in the home/community.
    1. Did you decide to get ART at home/in the community? *If NO: Skip to Question 6.*
    2. Why did you make the choice you made?
    3. Usually health care workers don't give people a choice of what service you get. But for you, you were given the option between home or facility. How did having this choice (having a say) make you feel?
6. How do you currently get ARTs?
    1. IF at HOME/COMMUNITY: How many times have you gotten your medication at home?
    2. If at the FACILITY: When you went back to the facility, did the did a health care worker/patient supporter offer you any other services?
        i. What did they do?
        ii. Was this helpful for you? Why/why not?
7. Has either HCW been supporting you or chatting with you about anything else in addition to HIV or ART?
    1. Do you ever contact either HCW directly to chat or ask questions?
        i. IF YES: How often do you talk with them? What do you talk about?
    2. Is your relationship with either HCW different to the ones you have had with other HCWs before joing the study? How?
        i. IF YES: Do you think this relationship helps you more than your relationship with other HCWs? IF YES: How?
8. Now think about overall your interactions with the health care workers you interacted with since you started the study – the Patient Supporter and the Nurse who brought you ART. What did you like about your intereactions with them? How do you think it helps you with ART?

For peer review only

9. With everything in life, there are some things we like and some things that could be a little better. What did you NOT like about your interactions with the Patient Supporter and the Nurse who brought you ART?
10. Is there anything else you think you need a health care worker or the health facility could do differently in order for you to be comfortable taking ART regularly? Is there anything else you need?

Section 3: Initiation
Now I'd like to ask you about what has happened with your ARVs since you were enrolled in the study.
11. Did you start taking ART (again) since enrolling in the study?
    If YES:
    a. Why did you (re) initiate ART? What convinced you it was good to take medication (again)?
    b. Who was most influential in your decision to start ART (again)?
    If NO:
    c. Why have you not initiated ART since enrolling in the study?
    d. Has anyone influenced you to not start ART? Who? Why?
    e. Is there anything that could motivate/help you to initiate ART?
12. We know that starting ART can be difficult. What do you think is the most difficult thing about re-starting ART for you
13. Was there anything that made it easy for you to (re)start ART? (opposite of probes below)
    1. For those who opted for HOME-BASED: Do you think getting ART at home helped you re-engage in care? Why/why not?
14. For the next set of questions please feel free to be honest. There is no right or wrong answer.
    1. Do you feel MORE confident you can stay on ARTs in the future? Why/why not?
    2. Is there someone in your life now that encourages you?
    3. Do you have a better relationship with HCWs / or trust them more? Why/why not?
    4. Do you have a plan so that you don't run out of ARVs if youre away or busy?
    5. Do you think ARVs help you reach your goals for making money and for your family? Why/why not?

IN-DEPTH INTERVIEW FOR MEN – ENDLINE

Section 1: Pre-Trial Experiences

1. Think about when you stopped taking ART **before** joining the study. Can you remember why you stopped swallowing ART pills?
2. Talk to me about some things that made it difficult for YOU to stay on (or start) ART BEFORE joining the study?

Section 2: Post-Trial Experiences
3. As a part of the study, you received [ONLY MENTION THE SERVICE THAT ALIGNS WITH THE CLIENTS STUDY ARM: counseling, mentorship, counseling + home-based 1 month, counseling + home-based 3 months].
    a. How did you feel about this service?
    b. Was this helpful to you? How?
4. Since you enrolled in the study did you restart ARVS?
    a. IF NEVER RE-INITIATED ART IN THE STUDY: Why have you not re-started ART since joining the study?

For peer review only

5. Think about the challenges you experienced BEFORE joining the study. [REFER BACK TO THE CHALLENGES MENTIONED IN QUESTION 1 and 2]. Have these challenges gone away or been reduced since you joined the study?
   a. How?
   b. Why has it changed/not changed?

6. Have you had any **new or different** challenges since joining the study (either to attend refill appointments or taking your ART)? What are they?
   a. Why do you think you experience them now?

7. Are there things about attending refill appointments or swallowing ART that are now easier since you joined the study?
   a. What are they? Why?

8. Think about what you discussed with the study HCW who met with you [he may or may not have used this flip chart]. When he chatted with you, he might have told you some things that were **new** to you about how to keep taking ARTs. Have you tried doing any of those things AFTER you met with the HCW?
   a. What was it?
   b. Did it help you? How?
   c. If it did not help you, what challenges did you face?

9. Have you continued to talk to the study HCW since you first met with him?
   a. Describe your relationship.
      i. How frequently do you connect? In person, on the phone, both?
      ii. What do you discuss?
      iii. How is this relationship helpful for you and your life?
      iv. Do you wish for your relationship with the HCW to continue [or continued in the case of the HCW being fired]? Why? Why not?

10. [GENERAL NOT JUST STUDY HCWS] What additional services/interventions [not materials/monitary] do you wish you could have to help you consistently access or swallow ART?
   a. Would you need this for a short period of time (one time, or for a month) or would you need it ALL the time (for years)? Why?

11. Do you have other health concerns outside of HIV? What are they?
   a. Is it easier or harder for you to get care for these concerns as compared to getting ART services? Why?

Section 3: Repeat Treatment Interruption [ONLY FOR THOSE WHO REINTIATED]
Now I want to talk to you about your use of ART services since joining the study.

12. Have you missed an ART appointment or missed swallowing your medication on any day since you restarted ART?

If **NO:**

13. How have you managed to stay on ART? What strategies do you think are most useful to help you stay on ART? Are they different from the one's you mentioned before? Does someone or something specifically help you?

If **YES:**

14. Can you tell me what happened?
   a. For how long did you stop taking ART?
   b. How many times has it happened?
   c. Why did you miss ART appointment/swallowing ART?

15. What do you think would make it easier for men like you to not miss doses of ART or ART appointments?

16. Have you started taking ARVS **again** after you missed doses or gone back to the facility after you missed a refill appointment?

If **NO** – skip to question 18

If **YES**

17. Did anything made it easy for you to (re)start ART AGAIN?

For peer review only - http://bmjopen.bmj.com/site/about/guidelines.xhtml

18. We know that restarting ART can be difficult. What fears/worries/concerns did you have to overcome in order to restart (again)?
19. What would make it easier for men like you to come back to the clinic after missing an appointment?

Section 4: Client centered care choice experiment

20. We know there is no such thing as a 'perfect' healthcare worker. We are all human. But today we want to give you the opportunity to think about key things you want if you had the perfect interaction with a health care worker. Here is a picture of a health care worker. Around him we have characteristics on different cards [McDaphton to read through the cards]
   1. He treats me with respect
   2. His counseling message is easy to understand
   3. His counseling addresses my specific concerns
   4. He maintains privacy/keeps my secrets
   5. He keeps in contact with me (not just a one time counseling)
   6. He comes to the community to find me
   7. He treats me like family and cares for me as a person
   8. He asks me questions about my life/circumstances
   9. He takes his time with me (the counseling session is not rushed)
   10. He shares his own experiences openly as a fellow man
   11. He provides fast services

21. Id like you to choose the 6 most important characteristics you think are essential for how you want to interact with health care workers. There is no right or wrong answers. This is about what matters to you.
   a. Why have you chosen these? What makes them very important to you?

22. Now you can only choose 3 of these characteristics. Look at your 6 characteristics in front of you. What THREE are the most important/essential characteristics for your health care worker to still provide you with the services you would like.
   a. Why have you chosen these? Why did you NOT choose the other three?

Section 5: Income

23. What things do you do to earn a living? Think about all the work you do – this may include several different things.
   a. How do you earn money (what kind of work do you do)?
   b. Where do you work?
   c. Do you travel for work?
   d. How often do you work?
   e. How do you usually find this work? How do employers/bosses or customers find you or how do you find markets for your products?

24. What would happen to you and/or your family if you spent a whole day at the clinic and did not earn any money that day?
   a. Is it possible for you to attend your ART appoint and not miss out on any income/money? How is this possible for you?
   b. In the WORST case scenario: how much missed opportunities to earn money would you experience? What would be the biggest impact on your financial situation be if you attend a clinic appointment and miss work/work opportunities (exp: do you miss a client, did your shop not open, did you miss the chance to get a long term job?)
   c. Does missing a day from making money impact your family? How?

For peer review only - http://bmjopen.bmj.com/site/about/guidelines.xhtml

BMJ Open

Identifying efficient linkage strategies for HIV self-testing (IDEaL): a study protocol for an individually randomized control trial

Journal:	*BMJ Open*
Manuscript ID	bmjopen-2022-070896.R2
Article Type:	Protocol
Date Submitted by the Author:	05-May-2023
Complete List of Authors:	Dovel , Kathryn ; University of California Los Angeles, Division of Infectious Diseases ; Partners in Hope Medical Center, Implementation Science Department<br>Balakasi, Kelvin; Partners in Hope Medical Center, Implementation Science Department<br>Hubbard, Julie; University of California Los Angeles, Division of Infectious Diseases<br>Phiri, Khumbo; Partners in Hope Medical Center, Implementation Science Department<br>Nichols, Brooke ; Boston University, Department of Global Health; Health Economics and Epidemiology Research Office,<br>Coates, Thomas; University of California Los Angeles, Division of Infectious Diseases, School of Medicine<br>Kulich, Michal; Charles University, Department of Probability and Math Statistics<br>Chikuse, Elijah; Partners in Hope Medical Center, Implementation Science Department<br>Phiri, Sam; Partners in Hope Medical Center, Implementation Science Department<br>Long, Lawrence; Boston University School of Public Health, Department of Global Health; Faculty of Health Sciences University of the Witwatersrand, Department of Internal Medicine<br>Hoffman, Risa; University of California Los Angeles, Division of Infectious Diseases<br>Choko, Augustine; University of Malawi, College of Medicine
**Primary Subject Heading**:	HIV/AIDS
Secondary Subject Heading:	Global health, Health services research, Infectious diseases, Public health
Keywords:	HIV & AIDS < INFECTIOUS DISEASES, Infection control < INFECTIOUS DISEASES, Clinical trials < THERAPEUTICS

For peer review only - http://bmjopen.bmj.com/site/about/guidelines.xhtml

Identifying efficient linkage strategies for HIV self-testing (IDEaL): a study protocol for an individually randomized control trial

Kathryn Dovel[1,2§], Kelvin Balakasi[1], Julie Hubbard[1], Khumbo Phiri[1], Brooke E. Nichols[3,4], Thomas J. Coates[1], Michal Kulich[5], Elijah Chikuse[2], Sam Phiri[2], Lawrence Long[3,4], Risa Hoffman[1], Augustine Choko[6].

Author affiliations:

[1] Division of Infectious Diseases, David Geffen School of Medicine, University of California Los Angeles, Los Angeles, CA, USA
[2] Partners in Hope, Lilongwe, Malawi
[3] Department of Global Health, Boston University School of Public Health, Boston, MA, USA
[4] Department of Internal Medicine, Faculty of Health Sciences, University of the Witwatersrand, Johannesburg, South Africa
[5] Department of Probability and Math. Statistics, Faculty of Mathematics and Physics, Charles University, Prague, Czech Republic
[6] College of Medicine, University of Malawi, Blantyre, Malawi

[§] **Corresponding Author:** Kathryn Dovel
Division of Infectious Diseases, David Geffen School of Medicine
10833 Le Conte Ave, Los Angeles, CA 90095
Phone: +13108838179
Email: kdovel@mednet.ucla.edu / kldovel@gmail.com

E-mail addresses of authors:

KD: KDovel@mednet.ucla.edu
KB: kelvin@pihmalawi.com
JH: jhubbard@mednet.ucla.edu
KP: khumbo@pihmalawi.com
BN: brooken@bu.edu
TC: TCoates@mednet.ucla.edu
MK: kulich@karlin.mff.cuni.cz
EC: elijah@pihmalawi.com
SP: samphiri@pihmalawi.com
LL: lclong@bu.edu
RH: RHoffman@mednet.ucla.edu
AC: augutc@gmail.com

Word Count: Abstract: 304; Manuscript: 3,967

Keywords: HIV, men, ART initiation, ART retention, Stepped interventions, sub-Saharan Africa

Date and version: 5 May 2023: Version 3

For peer review only - http://bmjopen.bmj.com/site/about/guidelines.xhtml

For peer review only

ABSTRACT

Introduction

Men in sub-Saharan Africa are less likely than women to initiate antiretroviral therapy (ART) and more likely to have longer cycles of disengagement from ART programs. Treatment interventions that meet the unique needs of men are needed, but they must be scalable. We will test the impact of various interventions on six-month retention in ART programs among men living with HIV who are not currently engaged in care (never initiated ART and ART clients with treatment interruption).

Methods and Analysis

We will conduct a programmatic, individually randomized, non-blinded, controlled trial. "Non-engaged" men will be randomized 1:1:1 to either a Low-Intensity, High-Intensity, or Stepped arm. The Low-Intensity Intervention includes one-time male-specific counseling + facility navigation only. The High-Intensity Intervention offers immediate outside-facility ART initiation + male-specific counseling + facility navigation for follow-up ART visits. In the Stepped arm, intervention activities build in intensity over time for those who do not reengage in care with the following steps: 1) one-time male-specific counseling + facility navigation→2) ongoing male mentorship + facility navigation→3) outside-facility ART initiation + male-specific counseling + facility navigation for follow-up ART visits. Our primary outcome is 6-month retention in care. Secondary outcomes include cost-effectiveness and rates of adverse events. The primary analysis will be intention to treat with all eligible men in the denominator and all men retain in care at 6 months in the numerator. The proportions achieving the primary outcome will be compared with a risk ratio, corresponding 95% confidence interval and p-value computed using binomial regression accounting for clustering at facility level.

Ethics and Dissemination

The Institutional Review Board of the University of California, Los Angeles and the National Health Sciences Research Council in Malawi have approved the trial protocol. Findings will be disseminated rapidly in national and international forums and in peer-reviewed journals and are expected to provide urgently needed information to other countries and donors.

Trial registration number: NCT05137210.

ARTICLE SUMMARY

Strengths and limitations

- IDEaL provides male-specific differentiated models of care aimed to improve men's ART

For peer review only - http://bmjopen.bmj.com/site/about/guidelines.xhtml

outcomes. We specifically focus on building trusting relationships with health care workers and developing client-led, individualized strategies to overcome barriers to care.

- IDEaL will test the impact of a stepped intervention for men. This approach promises to improve the efficiency and reach of HIV programs for men as the highest-resource interventions will only be received by the minority of men who are most in need.
- IDEaL develops and tests male-specific counseling curriculum that, if effective, could easily be taken to scale. Findings from the study will identify critical components for male-specific counseling, especially among men who struggle to be retained in HIV care.
- IDEaL interventions do not change facility characteristics that may act as barriers to men's use of facility-based services. IDEaL focuses on providing outside-facility services for reaching men.

For peer review only

INTRODUCTION

Men in sub-Saharan Africa (SSA) are underrepresented in HIV programs.[1] Men are less likely than women to know their HIV status and to initiate antiretroviral therapy (ART), and more likely to face treatment interruptions once in care.[2] Only 69% of men who start ART reach viral suppression compared to 77% of women.[2] As a result, men in the region are 37% more likely to die from AIDS-related causes as compared to women.[3]

One contributor to men's poor HIV outcomes is an increased risk of disengagement from care. Engagement in ART programs is not static – many ART clients cycle through care, starting and stopping HIV care multiple times throughout their lifetime.[4,5] Up to 46% of ART clients experience treatment interruption,[6–8] and between 30-40% of those who return experience repeat treatment interruption within 6 months.[9,10] Men are particularly prone to cycling through ART programs, with more frequent stop-start instances and longer periods outside of care as compared to women.[6,11–13] Improving men's long-term engagement in HIV care is critical for men's health and reducing HIV transmission.[14]

Men who disengage from HIV programs (either after testing HIV-positive or after enrolling in HIV care services) are frequently described as a difficult and 'hard-to-reach' population.[15,16] However, growing evidence suggests that men desire HIV services[17,18] but encounter multiple health systems barriers to care that make it impossible to stay in care long-term.[19] There is an urgent need to develop client-centered strategies tailored to men that facilitate men's engagement and re-engagement in HIV treatment programs.

Some men may require male-specific interventions to facilitate engagement in HIV care. Men have less exposure to HIV services than women[19,20] and work demands may conflict with ART clinic schedules.[21,22] Difficult interactions with health care workers (HCWs) can also prevent men from engaging or re-engaging in care.[23,24] Furthermore, most ART counseling curricula do not target men and often lack the client-centered counseling needed to develop internal motivation to engage and stay engaged in care.

Differentiated service delivery models (DSD) are now being developed to improve men's ART engagement throughout SSA.[25–27] As DSDs for men are developed, it is critical that strategies be feasible and cost-effective to allow scale-up. A "one size fits all" model is not as effective as more nuanced approaches.[28–30] Stepped interventions increase in intensity over time and are purposively designed to address prevailing barriers in the target population in order to positively affect the desired outcome.[31,32] An incremental, stepped approach may be the most appropriate and scalable way to improve men's care in low-resource settings. Men are not homogeneous: some men may require minimal support to engage in

For peer review only - http://bmjopen.bmj.com/site/about/guidelines.xhtml

care, while others may require extensive support. Stepped interventions allow programs to target the highest-resource interventions to the minority of men who need them most.

The *Identifying efficient linkage strategies for HIV self-testing (IDEaL) trial* is an individually randomized control trial aimed to test the impact of various interventions on ART (re-) initiation and six-month retention among men living with HIV who are not currently engaged in HIV care in Malawi. We will compare a Stepped intervention against Low-Intensity and High-Intensity interventions to assess the impact of the Stepped intervention on men's use of ART services over time (see Supporting Information S1). The trial contributes to existing literature by testing male-specific, client-centered strategies to re-engage men in care. This is one of the first trials specifically designed with men's re-engagement in care in mind. If effective, such interventions may decrease repeat treatment interruption and duration of treatment interruptions among men, which can improve viral suppression and reduce onward HIV transmission.[14]

METHODS AND ANALYSIS

Objectives

Our primary objective is to test the effect of a male-specific, Stepped intervention on men's 6-month retention in ART care compared to male-specific Low-Intensity and High-Intensity interventions (retention is defined as <28-days late for their ART appointment). Secondary objectives are to understand the effect of a Stepped intervention on: (1) ART initiation; (2) the presence of adverse events (i.e., unwanted disclosure, end of relationship, or intimate partner violence (IPV)); (3) intervention acceptability; and (4) cost-effectiveness.

Trial Design

IDEaL is a programmatic, individually randomized, non-blinded controlled trial design. We will recruit men from 15 high-burden health facilities in Malawi using medical chart reviews to identify men who are living with HIV but not engaged in HIV care.

Randomization

Individual men will be block randomized using R by a biostatistician using a 1:1:1 ratio to either the Stepped, Low-Intensity, or High-Intensity study arm using a computer-generated program. Participants will be randomized in blocks of 3 and 6, depending on the number of men available for recruitment at each facility. After enrolling in the trial and completing a baseline survey, men will be assigned to a study ID based on the randomization list. Study ID's will be linked with the pre-assigned blocked

For peer review only - http://bmjopen.bmj.com/site/about/guidelines.xhtml

randomization and pre-loaded into the tablet device, but will be unknown to the study staff until survey and randomization modules are completed and saved, ensuring randomization cannot be manipulated by the study staff. Once finalized, the randomization results will appear on the tablet device as a picture, and will be shown to the participant to maximize transparency and study buy-in.

Interventions

The effectiveness of the Stepped Intervention will be compared to a Low-Intensity Intervention (one time male-specific counseling + facility navigation defined as escort to the facility (if desired) and orientation to the ART clinic and procedures) and to a High-Intensity Intervention (outside-facility ART initiation + male-specific counseling + facility navigation for follow-up ART visits). Across all arms, men who do not (re-)initiate in ART will continue receiving follow-up visits for up to three months, depending on preferences of the client. The number of intervention visits delivered for each participant will be documented.

Arm 1: Low-Intensity Arm: Male-specific counseling + facility navigation

Participants randomized to the Low-Intensity Arm will be traced in the community and receive a one-time, one-on-one male-specific counseling session, using client-centered service techniques.[33] All counseling sessions will be completed by a lay cadre male HIV counselor (called Patient Supporter in Malawi) trained in the study counseling curriculum. Patient supporters are responsible for routine tracing, linkage support medical record documentation and counseling.

The male-specific curriculum is developed specifically for this trial. Ministry of Health counseling materials is adapted to meet the specific needs of men, based on formative in-depth interviews, focus group discussions, and a systematic literature review. Adaptations will include exploring topics of most concern to men in Malawi (i.e., earning money while HIV-positive, side effects and concerns regarding lifelong medication, ART as a tool to provide and care for family, etc.). The materials will also include language and pictures that resonate with men (i.e., emphasizing how HIV and HIV services interact with men's strength, responsibility, planning for the future), and male-specific case studies of challenges men face and how they overcome them. The adapted male-specific counseling curriculum will be developed into a standardized counseling flip chart (i.e., job aid).

Men who wish to (re-)initiate ART will be offered facility navigation and facility-based services at the facility of their choice. Participants will be escorted to the facility (if desired), orientated to the ART clinic

For peer review only - http://bmjopen.bmj.com/site/about/guidelines.xhtml

procedures, and introduced to other HCWs who routinely work at the facility. Facility navigation is intended to facilitate a positive experience for men by helping them feel comfortable and confident navigating clinic spaces. Men may access all ART clinical services at the health facility of their choice (but counseling described above will be provided in the community). The client will be responsible for all transport costs related to return to the facility in all study arms.

Participants who do not (re-)initiate in care within 14 days will be offered followed-up counseling every two weeks until participants (re-)initiate in care or inform the counselor that they do not wish to be contacted.

Arm 2: High-Intensity Arm: Male-specific counseling + outside-facility ART initiation + facility navigation

Participants in the High-Intensity Arm will be offered community-based male-specific counseling by HIV counselors (described above), and offered ART (re-)initiation outside-facility (either at home or another location in the community of their choice). Those who choose outside-facility ART initiation will be referred to a male study nurse who will meet participants one-on-one at times and locations that are convenient for participants. The nurse will offer a brief counseling session, reviewing key topics from male-specific counseling curriculum that is most relevant to the individual participant. Nurses will then conduct WHO staging. Individuals classified as WHO Stage 3 or 4 will be referred (and escorted, if desired) to the nearest public health facility for additional services. Participants classified as WHO Stage 1 or 2 will be given same day ART. Prior to their 4-week follow-up ART appointment participants will receive facility navigation by the same nurse and afterward access ART services.

Participants who choose facility-based ART (re-)initiation will be referred (and escorted, if desired) by the HIV counselor (lay cadre) to the nearest facility of their choice and receive facility navigation on a day and time that is convenient for them.

Men who do not (re-)initiate in care will be offered biweekly follow-up counseling at times and intervals determined by participants' preferences until they engage in care or inform the nurse that they no longer wish to be contacted.

Arm 3: Stepped Arm

The Stepped arm will build in intensity over time for those who have not (re-)initiate in care 14-days after study enrollment, or who do not return for their first ART follow-up appointment after (re-)initiation (see

For peer review only - http://bmjopen.bmj.com/site/about/guidelines.xhtml

Fig 1). Individuals will move to the next "step" every 2-weeks, moving from the lightest to the most intensive interventions over the course of 6 weeks until (re-)initiation has been achieved. The Stepped arm includes the following steps:

Step 1: Male-Specific Counseling + Facility Navigation and Facility-Based ART Services: Step 1 includes the same components described in the Low-Intensity Arm. Briefly, participants will be traced in the community and receive a one-time male-specific counseling session, with repeat counseling sessions if participants do not (re-)initiate within 14 days after the first counseling session. Men who wish to (re-) initiate in care will be provided facility navigation and standard of care facility-based services.

Step 2: Ongoing Motivational Interviewing + Facility Navigation and Facility-Based ART Services: Men who do not (re-)initiate in care (either have not engaged ART within 14 days of enrollment or do (re-)initiate ART but are ≥7-days late for a follow-up appointment) will move to the next 'step' of the intervention, which adds ongoing motivational interviewing to their package of activities. Motivational interviewing is a client-centered, client-led method for counseling that helps participants identify barriers to a desired outcome and develop personalized solutions.[34,35] The strategy has successfully been used with ART clients.[36] Mentors will work with participants to: (1) build self-efficacy, (2) identify internal motivations for the desired behavior, and (3) establish strategies and short- and long-term goals needed to reach ART initiation and retention. A male mentor specifically trained in motivational interviewing adapted to the local context and male population will provide ongoing, one-on-one in-depth counseling, motivational interviewing, and general "check-ins" approximately twice within a two-week period. The mentor will not necessarily be HIV-positive (unlike other mentorship models) as the Malawi Ministry of Health has moved away from HIV-positive peer mentor cadres. However, they will be experienced in HIV counseling and trained on male-specific needs. Motivational interviewing will take place in a location preferred by the participant, likely in the community. Participants who choose to (re) initiate ART can access ART services at the facility of their choice and will be given facility navigation as described in Arm 1.

Step 3: Outside-facility ART initiation + Male-Specific Counseling: Men who are not engaged after Step 2 (either have not (re-)initiated ART ≤ 14 days after moving to Step 2 or did (re-)initiate but are ≥7-days late for a follow-up ART appointment) will be offered outside-facility ART by a male nurse certified in HIV counseling. Steps will follow those outlined in the High Intensity Arm, with a brief counseling session, WHO staging, same-day ART re-initiation for those WHO stage 1 or 2, and facility navigation for their 4-week follow-up appointment.

For peer review only - http://bmjopen.bmj.com/site/about/guidelines.xhtml

[Figure 1 here]

Trial setting

The study will take place in central and southern Malawi. Malawi has an HIV prevalence of 9.6%[37] and, of the estimated 330,000 men living with HIV in the country, 54,500 are not in care.[38] Men in Malawi live in primarily rural settings, are self-employed, subsistence farmers, the minority have regular access to a private phone, and most are highly mobile.[39,40]

Population

We will recruit men from 13 high-burden health facilities in Malawi, using medical chart reviews to identify men living with HIV who are not engaged in HIV care. Study facilities will vary by facility type (hospital/health center), management (public/mission), location (rural/urban), and region (central/southern Malawi).

Eligibility criteria for men include: (1) ≥15years of age; (2) live in facility catchment area; and (3) tested HIV-positive and either (a) self-report having not yet initiated ART within 7-days of testing HIV-positive, (b) initiated ART but are at risk of immediate default (i.e., ≥7-days late for their 30-day ART refill appointment), or (c) initiated ART and attended their first refill appointment but later defaulted (i.e. ≥28-days late to care). For those who never initiated ART and do not have proof of a confirmatory HIV test, study staff will offer an HIV self-test kit prior to enrollment, to confirm a positive HIV status. Those who choose to initiate ART will receive the standard Determine and Unigold confirmatory tests prior to ART initiation, following routine care.

Study outcomes

The primary outcome is the proportion of men who are retained in ART care 6-months after (re-) engagement. Secondary outcomes include: (1) ART initiation; (2) adverse events experienced by men or their female partners (i.e., unwanted disclosure, end of relationship, or intimate partner violence (IPV); (3) intervention acceptability; and (4) cost-effectiveness. ART retention outcomes will be measured through medical chart reviews, while secondary outcomes will be measured through self-reports. Process outcomes include: (1) the proportion of men who were successfully traced; (2) the proportion of eligible men who consented to participate; (3) men's experience with the intervention; and (4) the quality of the intervention.

Sample size considerations

For peer review only - http://bmjopen.bmj.com/site/about/guidelines.xhtml

We powered the study to detect differences in 6-month retention between Stepped and Low-Intensity Arms, and the Stepped and High-Intensity Arms. Based on pilot data, we assumed that 40% of men in the Low-Intensity Arm, 60% in the Stepped Arm, and 80% in the High-Intensity Arm will engage in ART and be retained at 6-months. Any man lost to follow-up will be treated as a failure for the outcome evaluation. With 181 men per arm and 20% loss to follow-up from the study in all arms, the power for detecting the specified differences between Stepped and Low Intensity arms and between Stepped and High-Intensity arms will be 0.8, with test level 0.025 after Bonferroni adjustment for two comparisons. We will have 0.99 power to detect specified differences between Low Intensity and High Intensity arms. The calculation is based on asymptotic normality of log odds ratio.[41] We need to enroll and randomize 181 men per arm (a total of 543 men living with HIV).

Data Collection

Study recruitment, enrollment, and data collection will be conducted by study staff, who are distinct from local HCWs implementing the interventions.

Recruitment

Men will be identified through both medical register reviews and in-person recruitment at participating health facilities. Various medical charts will be reviewed to identify different types of eligible men: HIV testing and counseling (HTC) to identify men who tested HIV positive but never initiated ART; client follow-up registers to identify those who initiated but never returned for their first ART appointment, or those who defaulted from care; and index counseling and testing (ICT) registers to identify male partners of female ART clients (Figure 2). In-person recruitment will involve screening men at outpatient departments (OPD) because our previous research has found that men in Malawi frequent OPD settings for health needs,[17] and our formative work suggests that men who disengage from ART services still frequent the OPD for care. In-person recruitment will be used for all client types.

[Figure 2 here]

Tracing and Eligibility Screening

Study staff will trace potential participants identified through medical chart reviews via phone (if available) or home visits based on tracing data provided in medical documentation. All potential participants will be traced up to three times before being considered lost to follow-up. All screening and enrollment processes will take place in-person.

For peer review only

Consent, Enrollment, and Baseline Survey

Men who are eligible for the study will complete written informed consent and complete a baseline survey immediately following enrollment. The baseline survey will collect data on key demographic variables (marital status, number of children, employment, self-rated health) and previous engagement with HIV and non-HIV health services. All surveys will be conducted in the local language (Chichewa) by trained study staff using electronic tablets. Surveys will be programmed using SurveyCTO software (http://www.surveycto.com).

Follow-Up Data

Study staff will administer follow-up surveys at 2- and 4-months after enrollment. Follow-up surveys will measure exposure to (and acceptability of) the interventions, changes in key demographics since enrollment (i.e., marital status, number of children, employment, self-rated health), any adverse events since enrollment (i.e., unwanted status disclosure, termination of relationship due to the intervention), and use of ART services. The location and specific time of the follow-up survey will be based on participant preference.

Medical chart reviews will be conducted to assess men's engagement with ART services 6-months after study enrollment. Individuals without a medical chart outcome will be followed-up in person and their health passport, a pocket medical record where providers record data during health visits, will be reviewed to collect the ART outcome. Men who cannot be reached or are lost to follow-up in any arm will be counted as failures for that specific ART outcome of interest: (re-)initiation or 6-month retention).

Patient and Public Involvement

Extensive formative work informed the development of the study protocol including in-depth interviews, focus group discussions, and a systematic literature review. The study protocol and tools were presented to Ministry of Health, national stakeholders and implementing partners (see Supporting Information S2).

Cost data

The average cost per successful outcome (6-month retention) will be calculated and compared across arms incrementally. We will use micro-costing methods by creating an inventory of the resources used to achieve the observed study outcomes including: (1) standard counseling interactions (staff cadre, training received, duration of interaction and distance from facility travelled where applicable); (2) motivational interviewing interactions (staff cadre, training received, duration of interaction and distance from facility travelled where applicable); (3) provider interactions (staff cadre, training received, duration of

For peer review only - http://bmjopen.bmj.com/site/about/guidelines.xhtml

interaction and distance from facility travelled where applicable); and (4) cost of reminder messages sent, when messages are delivered telephonically instead of in person. For each study patient, the quantity (number of units) of resources used will be determined. Costs will be measured from the health care provider perspective. Unit costs of resources, which are not human subject data, will be obtained from external suppliers and the health facilities' finance and procurement records and multiplied by the resource usage data to provide an average cost per study patient in each study arm. A cost-effectiveness analysis will be conducted by dividing the incremental cost between two arms by the incremental effectiveness (number of people retained at 6-months) in the respective arms.

Analysis plan

Data analysis will be conducted in R: A Language and Environment for Statistical Computing (R Foundation for Statistical Computing). We will use the Consolidated Standards of Reporting Trials (CONSORT) standards for reporting trial outcomes.[42] Using an intention-to-treat analysis, all randomized men will be included in the analysis of the primary outcome; men with missing outcomes due to loss to follow-up will be treated as outcome failures. We will calculate descriptive statistics, including mean, standard deviation, range, and frequency distributions for the demographic characteristics and study outcomes by study arm. The primary outcome and all other binary outcomes will be analyzed by logistic regression models with age, marital status, health care facility and other key sociodemographic variables included as covariates. The intervention effects will be tested by Wald tests of the relevant regression parameters. The hypotheses of no difference between the stepped arm and the low/high intensity arm will be rejected if the p-value is smaller than 0.025 (Bonferroni adjustment). Confidence intervals for odds ratios comparing the stepped arm to the low intensity and high intensity arms with coverage probabilities 0.975 will be calculated by profile likelihood methods. Due to the Bonferroni adjustment, the simultaneous coverage probability of both intervals will be at least 0.95. To address the secondary objectives, more elaborate logistic regression models will be built for each of the binary outcomes with available individual-, community-, and facility-level factors included as covariates in addition to the intervention status.

Nested studies

A series of nested, mixed methods studies will be conducted to identify factors associated with ART engagement within each intervention arm, and to explore the implementation and acceptability of interventions.

Qualitative data collection

For peer review only - http://bmjopen.bmj.com/site/about/guidelines.xhtml

Guided by Grounded Theory, we will conduct cross-sectional in-depth interviews with a random subset of 40 male participants per arm (120 total) throughout the study period. Clients will be randomly selected at various times of the study using computer-generated randomization, stratifying the sample by arm and successful trial outcomes (i.e., did clients re-initiate ART and/or reach 6-month retention). Data will assess characteristics of men who fail to engage in care, contextualize decisions around ART initiation and retention, and identify additional strategies that may be needed for men to successfully engage and be retained in ART programs (see Supporting Information 3). Data collection tools and analysis plans will be informed by the Andersen's Emerging Model of Health Services Use, phase 4[43] that examines multi-level factors that influence health outcomes. Specifically, it examines the interaction of: 1) environment and structure of health services; 2) clients' enabling resources; and 3) clients' perceived need/motivation to access services. Qualitatively understanding how the IDEaL interventions influence these levels, and what barriers still remain, will help refine future interventions.

Interviews will be conducted by a trained male interviewer in the local language. Interviews will be digitally recorded, transcribed, and translated into English for analysis. Four investigators will pilot a codebook by independently reading and coding a randomly-selected subset of transcripts. Through an iterative consultative process and using iterative and deductive coding strategies, each investigator will revise their respective codebook until there is high interrater reliability among the group. All transcripts will be coded in Atlas.ti v8.3[44] and text analyzed using constant comparison methods[45] to compare and contrast themes that arise within and between interventions and trial outcomes.

Implementation Log Sheet

During the course of the intervention, HCWs will keep daily logs as one of the study monitoring and evaluation tools to assess the implementation of the intervention for each participant. Primary events to be recorded in the daily logs are: (1) unable to reach participant (and reason); (2) contacted participant; (3) intervention provided (and notes about the challenges and successes of the interaction); and (4) other comments relevant to intervention implementation. Each event will be recorded with a corresponding date. Logs will be digitized in English. Findings may influence how similar interventions are implemented in the future.

Ethics and Dissemination

The IDEaL trial is registered with ClinicalTrials.gov as NCT05137210. The protocol was approved by the Institutional Review Board of the University of California, Los Angeles and the National Health Sciences Research Council in Malawi. Study findings will be disseminated through peer-reviewed journal articles,

For peer review only

national and international conference presentations, and meetings with Malawi Ministry of Health, facility, and community stakeholders.

DISCUSSION

Studies have reported poorer outcomes for HIV testing, treatment initiation, and treatment adherence in men compared to women[2] for over a decade.[46,47] Men are often portrayed as difficult, hard-to-reach, and actively avoiding health facilities. In IDEaL, we aim to investigate whether men really are hard-to-reach or, will men engage in care when services are offered in ways that are accessible to them and resonate with their needs, as growing evidence suggests.[17] We propose to test a Stepped intervention that increases in intensity over time against Low- and High-intensity interventions – all tailored to men – to identify the most cost effective strategy to (re-)initiate men in HIV treatment services in Malawi.

IDEaL is different from other ART engagement and re-engagement interventions in several important ways. First, we will enroll men living with HIV across the treatment cascade, including those who have never initiated ART, those who are at risk of immediate default after initiation, and those who have been in care but subsequently default. Formative research suggests that barriers to ART initiation and re-initiation may be similar,[48] however most interventions focus specifically on either first-time initiation or re-engagement, but not both. Our study will assess if one overarching program can improve men's engagement across the treatment cascade, regardless of whether they are starting ART for the first time or returning to care after a period of disengagement. One overarching intervention may be more scalable than multiple, separate interventions across the cascade. Second, we tailor interventions to men's unique needs and motivations, based on extensive formative work. While innovative interventions for men are underway,[25–27] few have rigorously tested the impact of male-tailored interventions on ART engagement.[49]

Finally, we will test a Stepped intervention that builds in intensity over time until men (re-)initiate in care. This approach allows men who are ready to (re-)initiate to do so at minimal cost to the health system, while those who need additional support can receive more resource-intensive interventions to support their ART engagement.[31] Stepped interventions have been effective in other settings and can address multiple barriers faced by the target population with minimal cost.[31,32] Findings from IDEaL will provide crucial knowledge to how best men can be reached and can inform intervention scale-up.

Contributorship statement: KD and AC conceptualized the study. KD is responsible for funding acquisition. KD, KB, JH, KP, BN, RH and AC developed study protocol and materials. JH, KB, KP, and

For peer review only - http://bmjopen.bmj.com/site/about/guidelines.xhtml

EC will implement the study. KD, MK, KB, TC, BN, LL, TC, and AC developed the analysis plan and KD, MK, KB, and AC will analyze the data. KD and EC wrote the first draft and KB, JH, KP, BN, LL, RH, SP, EC, RH, TC, and AC edited following drafts. All authors have read and approved the final manuscript.

Competing interests: The authors declare that they have no competing interests.

Funding: The work was supported by the Bill and Melinda Gates Foundation grant number INV-001423. KD was supported by National Institute of Mental Health of the National Institutes of Health grant number R01-MH122308, Fogarty International grant number K01-TW011484-01 and UCLA GSTTP (grant number N/A). LL was supported by the National Institute of Mental Health of the National Institutes of Health under grant number K01MH119923. The content is solely the responsibility of the authors and does not necessarily represent the official views of the National Institutes of Health.

Acknowledgements: We wish to thank the Malawi Ministry of Health for their support of this trial. We would also like to acknowledge Joep van Oosterhout, Misheck Mphande, Isabella Robson, Thoko Banda, Peter Mwamlima, and Eric Lungu for their contributions to protocol development and for their support of study implementation.

For peer review only

REFERENCES

1. Cornell M, Majola M, Johnson LF, Dubula-Majola V. HIV services in sub-Saharan Africa: the greatest gap is men. *The Lancet*. 2021;397(10290):2130-2132.
2. AIDSinfo | UNAIDS. Accessed February 21, 2023. https://aidsinfo.unaids.org/
3. Druyts E, Dybul M, Kanters S, et al. Male sex and the risk of mortality among individuals enrolled in antiretroviral therapy programs in Africa: a systematic review and meta-analysis. *Aids*. 2013;27(3):417-425.
4. Ehrenkranz P, Rosen S, Boulle A, et al. The revolving door of HIV care: Revising the service delivery cascade to achieve the UNAIDS 95-95-95 goals. *PLoS Medicine*. 2021;18(5):e1003651.
5. Grimsrud A, Wilkinson L, Eshun-Wilson I, Holmes C, Sikazwe I, Katz IT. Understanding engagement in HIV programmes: how health services can adapt to ensure no one is left behind. *Current HIV/AIDS Reports*. 2020;17:458-466.
6. Kranzer K, Lewis JJ, Ford N, et al. Treatment interruption in a primary care antiretroviral therapy programme in South Africa: cohort analysis of trends and risk factors. *Journal of acquired immune deficiency syndromes (1999)*. 2010;55(3):e17.
7. Mee P, Rice B, Lemsalu L, et al. Changes in patterns of retention in HIV care and antiretroviral treatment in Tanzania between 2008 and 2016: an analysis of routinely collected national programme data. *Journal of global health*. 2019;9(1).
8. Tomescu S, Crompton T, Adebayo J, et al. Factors associated with an interruption in treatment of people living with HIV in USAID-supported states in Nigeria: a retrospective study from 2000–2020. *BMC public health*. 2021;21:1-8.
9. Nakiwogga-Muwanga A, Musaazi J, Katabira E, Worodria W, Talisuna SA, Colebunders R. Patients who return to care after tracking remain at high risk of attrition: experience from a large HIV clinic, Uganda. *International journal of STD & AIDS*. 2015;26(1):42-47.
10. Teklu AM, Yirdaw KD. Patients who restart antiretroviral medication after interruption remain at high risk of unfavorable outcomes in Ethiopia. *BMC Health Services Research*. 2017;17(1):1-8.
11. Lopez-Varela E, Augusto O, Fuente-Soro L, et al. Quantifying the gender gap in the HIV care cascade in southern Mozambique: We are missing the men. *Plos one*. 2021;16(2):e0245461.
12. Mody A, Eshun-Wilson I, Sikombe K, et al. Longitudinal engagement trajectories and risk of death among new ART starters in Zambia: A group-based multi-trajectory analysis. *PLOS medicine*. 2019;16(10):e1002959.
13. Gosset A, Protopopescu C, Larmarange J, et al. Retention in care trajectories of HIV-positive individuals participating in a universal test-and-treat program in rural South Africa (ANRS 12249 TasP Trial). *Journal of Acquired Immune Deficiency Syndromes (1999)*. 2019;80(4):375.
14. Monod M, Brizzi A, Galiwango RM, et al. Growing gender disparity in HIV infection in Africa: sources and policy implications. *medRxiv*. Published online 2023:2023-03.
15. Esacove AW. Good sex/bad sex: the individualised focus of US HIV prevention policy in sub-Saharan Africa, 1995–2005. *Sociology of health & illness*. 2013;35(1):33-48.
16. Poulin M, Dovel K, Watkins SC. Men with money and the "vulnerable women" client category in an AIDS epidemic. *World Development*. 2016;85:16-30.
17. Dovel K, Balakasi K, Gupta S, et al. Frequency of visits to health facilities and HIV services offered to men, Malawi. *Bulletin of the World Health Organization*. 2021;99(9):618.
18. Chamberlin S, Mphande M, Phiri K, Kalande P, Dovel K. How HIV Clients Find Their Way Back to the ART Clinic: A Qualitative Study of Disengagement and Re-engagement with HIV Care in Malawi. *AIDS and Behavior*. 2022;26(3):674-685.
19. Dovel K, Dworkin SL, Cornell M, Coates TJ, Yeatman S. Gendered health institutions: examining the organization of health services and men's use of HIV testing in Malawi. *Journal of the International AIDS Society*. 2020;23:e25517.

For peer review only - http://bmjopen.bmj.com/site/about/guidelines.xhtml

20. Yeatman S, Chamberlin S, Dovel K. Women's (health) work: a population-based, cross-sectional study of gender differences in time spent seeking health care in Malawi. *PLoS One*. 2018;13(12):e0209586.
21. Colvin CJ. Strategies for engaging men in HIV services. *The Lancet HIV*. 2019;6(3):e191-e200.
22. Phiri K, McBride K, Moucheraud C, et al. Community and health system factors associated with antiretroviral therapy initiation among men and women in Malawi: a mixed methods study exploring gender-specific barriers to care. *International health*. 2021;13(3):253-261.
23. Ware NC, Wyatt MA, Geng EH, et al. Toward an understanding of disengagement from HIV treatment and care in sub-Saharan Africa: a qualitative study. *PLoS medicine*. 2013;10(1):e1001369.
24. Layer EH, Brahmbhatt H, Beckham SW, et al. "I pray that they accept me without scolding:" Experiences with disengagement and re-engagement in HIV care and treatment services in Tanzania. *AIDS patient care and STDs*. 2014;28(9):483-488.
25. Bell J, Sharma S, Malone S, et al. Targeting interventions for HIV testing and treatment uptake: an attitudinal and behavioural segmentation of men aged 20–34 in KwaZulu-Natal and Mpumalanga, South Africa. *PloS one*. 2021;16(3):e0247483.
26. van Rooyen H, Makusha T, Joseph P, et al. Zwakala Ndoda: a cluster and individually randomized trial aimed at improving testing, linkage, and adherence to treatment for hard-to reach men in KwaZulu-Natal, South Africa. *Trials*. 2019;20(1):1-14.
27. MenStar Coalition | Ending AIDS for all. MenStar Coalition. Accessed March 15, 2023. https://www.menstarcoalition.org/
28. Grimsrud A, Bygrave H, Doherty M, et al. Reimagining HIV service delivery: the role of differentiated care from prevention to suppression. *Journal of the International AIDS Society*. 2016;19(1).
29. Duncombe C, Rosenblum S, Hellmann N, et al. Reframing HIV care: putting people at the centre of antiretroviral delivery. *Tropical Medicine & International Health*. 2015;20(4):430-447.
30. Hagey JM, Li X, Barr-Walker J, et al. Differentiated HIV care in sub-Saharan Africa: a scoping review to inform antiretroviral therapy provision for stable HIV-infected individuals in Kenya. *AIDS care*. 2018;30(12):1477-1487.
31. Bower P, Gilbody S. Stepped care in psychological therapies: access, effectiveness and efficiency: narrative literature review. *The British Journal of Psychiatry*. 2005;186(1):11-17.
32. McNairy ML, Lamb MR, Gachuhi AB, et al. Effectiveness of a combination strategy for linkage and retention in adult HIV care in Swaziland: The Link4Health cluster randomized trial. *PLoS medicine*. 2017;14(11):e1002420.
33. Hubbard J, Mphande M, Robson I, Balakasi K, Phiri K. Core components of male-specific person-centered care: a qualitative analysis from client and healthcare worker perspectives in Malawi. In: ; 2023.
34. Miller WR, Rose GS. Toward a theory of motivational interviewing. *American psychologist*. 2009;64(6):527.
35. Rollnick S, Miller WR, Butler CC, Aloia MS. Motivational interviewing in health care: helping patients change behavior. Published online 2008.
36. Hill S, Kavookjian J. Motivational interviewing as a behavioral intervention to increase HAART adherence in patients who are HIV-positive: a systematic review of the literature. *AIDS care*. 2012;24(5):583-592.
37. Focus on: Malawi | UNAIDS. Accessed March 15, 2023. https://www.unaids.org/en/20190402_country_focus_Malawi
38. Malawi | UNAIDS. Accessed March 15, 2023. https://www.unaids.org/en/regionscountries/countries/malawi
39. Feldacker C, Emch M, Ennett S. The who and where of HIV in rural Malawi: Exploring the effects of person and place on individual HIV status. *Health & place*. 2010;16(5):996-1006.
40. Mandiwa C, Namondwe B. Uptake and correlates of HIV testing among men in Malawi: evidence from a national population–based household survey. *BMC health services research*. 2019;19:1-8.

For peer review only - http://bmjopen.bmj.com/site/about/guidelines.xhtml

41. Jewell NP. *Statistics for Epidemiology*. CRC press; 2003.
42. Schulz KF, Altman DG, Moher D. CONSORT 2010 statement: Updated guidelines for reporting parallel group randomized trials. *Annals of Internal Medicine*. 2011;154(4):291-292.
43. Andersen RM. National health surveys and the behavioral model of health services use. *Medical care*. Published online 2008:647-653.
44. ATLAS.ti 9 [computer program].
45. Glaser BG. The constant comparative method of qualitative analysis. *Social problems*. 1965;12(4):436-445.
46. Higgins JA, Hoffman S, Dworkin SL. Rethinking gender, heterosexual men, and women's vulnerability to HIV/AIDS. *American journal of public health*. 2010;100(3):435-445.
47. Braitstein P, Boulle A, Nash D, et al. Gender and the use of antiretroviral treatment in resource-constrained settings: findings from a multicenter collaboration. *Journal of women's health*. 2008;17(1):47-55.
48. Coursey K, Kalande P, Phiri K, Chamberlin S, Coates T, Dovel K. Developing intervention strategies to improve ART program initiation and retention among HIV-positive men in Malawi.
49. Kusemererwa S, Akena D, Nakanjako D, et al. Strategies for retention of heterosexual men in HIV care in sub-Saharan Africa: a systematic review. *PloS one*. 2021;16(2):e0246471.

For peer review only

FIGURE LEGEND

Figure 1: Trial Design

Figure 2: Recruitment sources and ART disengagement criteria by recruitment type

SUPPORTING INFORMATION

Supporting Information 1: Spirit Checklist

Supporting Information 2: Approved protocol

Supporting Information 3: In-depth interview guides

Figure 1: Trial Design

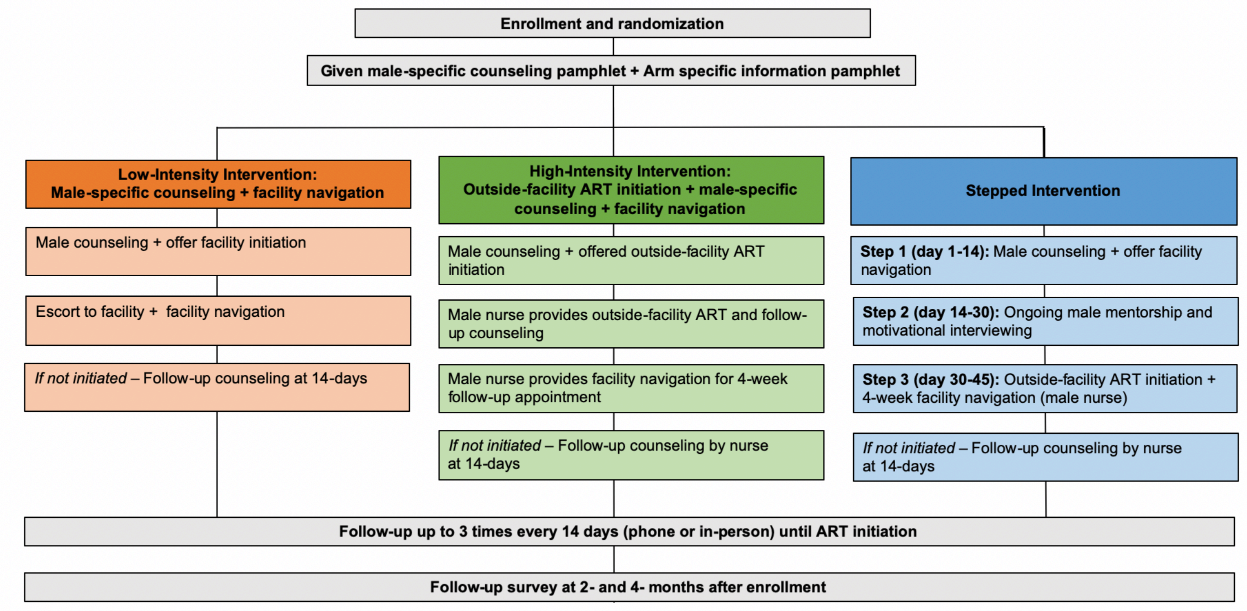

Fig 2. Recruitment sources and ART disengagement criteria by recruitment type

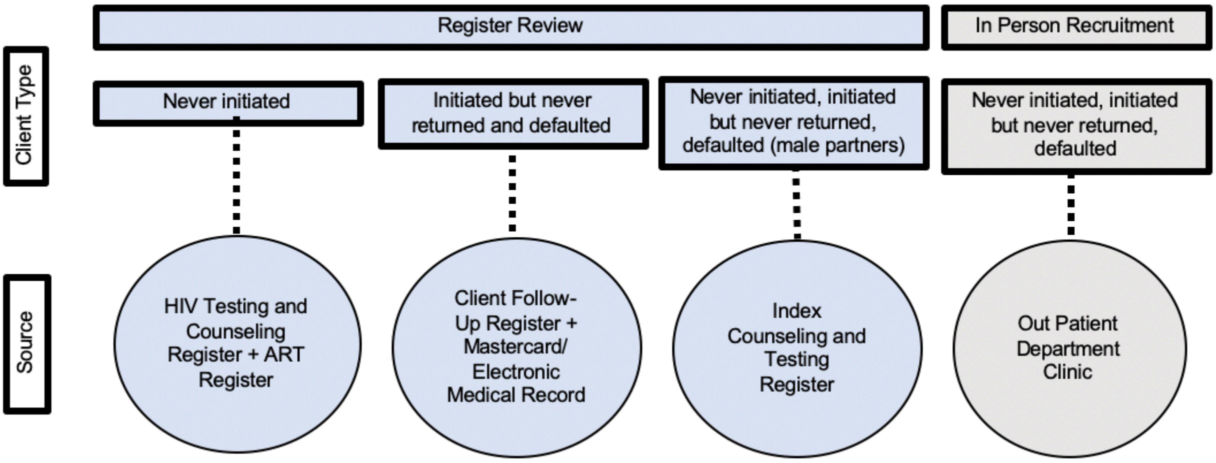

For peer review only

SPIRIT

STANDARD PROTOCOL ITEMS: RECOMMENDATIONS FOR INTERVENTIONAL TRIALS

SPIRIT 2013 Checklist: Recommended items to address in a clinical trial protocol and related documents*

Section/item	Item No	Description	Addressed on page number
**Administrative information**			
Title	1	Descriptive title identifying the study design, population, interventions, and, if applicable, trial acronym	______1_______
Trial registration	2a	Trial identifier and registry name. If not yet registered, name of intended registry	______1, 15_____
	2b	All items from the World Health Organization Trial Registration Data Set	______n/a______
Protocol version	3	Date and version identifier	______2_______
Funding	4	Sources and types of financial, material, and other support	______19______
Roles and responsibilities	5a	Names, affiliations, and roles of protocol contributors	_____18-19____
	5b	Name and contact information for the trial sponsor	______19______
	5c	Role of study sponsor and funders, if any, in study design; collection, management, analysis, and interpretation of data; writing of the report; and the decision to submit the report for publication, including whether they will have ultimate authority over any of these activities	______19_______
	5d	Composition, roles, and responsibilities of the coordinating centre, steering committee, endpoint adjudication committee, data management team, and other individuals or groups overseeing the trial, if applicable (see Item 21a for data monitoring committee)	_____n/a_______

For peer review only

Introduction

Background and rationale	6a	Description of research question and justification for undertaking the trial, including summary of relevant studies (published and unpublished) examining benefits and harms for each intervention	____4-5_______
	6b	Explanation for choice of comparators	_____ ________
Objectives	7	Specific objectives or hypotheses	_____5_______
Trial design	8	Description of trial design including type of trial (eg, parallel group, crossover, factorial, single group), allocation ratio, and framework (eg, superiority, equivalence, noninferiority, exploratory)	_______5_______

Methods: Participants, interventions, and outcomes

Study setting	9	Description of study settings (eg, community clinic, academic hospital) and list of countries where data will be collected. Reference to where list of study sites can be obtained	____9-10_______
Eligibility criteria	10	Inclusion and exclusion criteria for participants. If applicable, eligibility criteria for study centres and individuals who will perform the interventions (eg, surgeons, psychotherapists)	____10______
Interventions	11a	Interventions for each group with sufficient detail to allow replication, including how and when they will be administered	____6-9______
	11b	Criteria for discontinuing or modifying allocated interventions for a given trial participant (eg, drug dose change in response to harms, participant request, or improving/worsening disease)	_______n/a______
	11c	Strategies to improve adherence to intervention protocols, and any procedures for monitoring adherence (eg, drug tablet return, laboratory tests)	_______n/a______
	11d	Relevant concomitant care and interventions that are permitted or prohibited during the trial	_______n/a______
Outcomes	12	Primary, secondary, and other outcomes, including the specific measurement variable (eg, systolic blood pressure), analysis metric (eg, change from baseline, final value, time to event), method of aggregation (eg, median, proportion), and time point for each outcome. Explanation of the clinical relevance of chosen efficacy and harm outcomes is strongly recommended	_____10_____
Participant timeline	13	Time schedule of enrolment, interventions (including any run-ins and washouts), assessments, and visits for participants. A schematic diagram is highly recommended (see Figure)	__6,8-9, 11-12__

For peer review only - http://bmjopen.bmj.com/site/about/guidelines.xhtml

Sample size	14	Estimated number of participants needed to achieve study objectives and how it was determined, including clinical and statistical assumptions supporting any sample size calculations	____10-11_____
Recruitment	15	Strategies for achieving adequate participant enrolment to reach target sample size	____11-12______

Methods: Assignment of interventions (for controlled trials)

Allocation:

Sequence generation	16a	Method of generating the allocation sequence (eg, computer-generated random numbers), and list of any factors for stratification. To reduce predictability of a random sequence, details of any planned restriction (eg, blocking) should be provided in a separate document that is unavailable to those who enrol participants or assign interventions	_____5-6_______
Allocation concealment mechanism	16b	Mechanism of implementing the allocation sequence (eg, central telephone; sequentially numbered, opaque, sealed envelopes), describing any steps to conceal the sequence until interventions are assigned	_____5-6_______
Implementation	16c	Who will generate the allocation sequence, who will enrol participants, and who will assign participants to interventions	______5_______
Blinding (masking)	17a	Who will be blinded after assignment to interventions (eg, trial participants, care providers, outcome assessors, data analysts), and how	______6_______
	17b	If blinded, circumstances under which unblinding is permissible, and procedure for revealing a participant's allocated intervention during the trial	______n/a______

Methods: Data collection, management, and analysis

Data collection methods	18a	Plans for assessment and collection of outcome, baseline, and other trial data, including any related processes to promote data quality (eg, duplicate measurements, training of assessors) and a description of study instruments (eg, questionnaires, laboratory tests) along with their reliability and validity, if known. Reference to where data collection forms can be found, if not in the protocol	_____12_____
	18b	Plans to promote participant retention and complete follow-up, including list of any outcome data to be collected for participants who discontinue or deviate from intervention protocols	_____12_____

For peer review only - http://bmjopen.bmj.com/site/about/guidelines.xhtml

Data management	19	Plans for data entry, coding, security, and storage, including any related processes to promote data quality (eg, double data entry; range checks for data values). Reference to where details of data management procedures can be found, if not in the protocol	_______n/a______
Statistical methods	20a	Statistical methods for analysing primary and secondary outcomes. Reference to where other details of the statistical analysis plan can be found, if not in the protocol	____13-14______
	20b	Methods for any additional analyses (eg, subgroup and adjusted analyses)	___13-14____
	20c	Definition of analysis population relating to protocol non-adherence (eg, as randomised analysis), and any statistical methods to handle missing data (eg, multiple imputation)	______13-14_____
**Methods: Monitoring**			
Data monitoring	21a	Composition of data monitoring committee (DMC); summary of its role and reporting structure; statement of whether it is independent from the sponsor and competing interests; and reference to where further details about its charter can be found, if not in the protocol. Alternatively, an explanation of why a DMC is not needed	_______n/a______
	21b	Description of any interim analyses and stopping guidelines, including who will have access to these interim results and make the final decision to terminate the trial	_______n/a______
Harms	22	Plans for collecting, assessing, reporting, and managing solicited and spontaneously reported adverse events and other unintended effects of trial interventions or trial conduct	____12 ______
Auditing	23	Frequency and procedures for auditing trial conduct, if any, and whether the process will be independent from investigators and the sponsor	_______n/a______
**Ethics and dissemination**			
Research ethics approval	24	Plans for seeking research ethics committee/institutional review board (REC/IRB) approval	_______15_______ _
Protocol amendments	25	Plans for communicating important protocol modifications (eg, changes to eligibility criteria, outcomes, analyses) to relevant parties (eg, investigators, REC/IRBs, trial participants, trial registries, journals, regulators)	______n/a______

For peer review only - http://bmjopen.bmj.com/site/about/guidelines.xhtml

Consent or assent	26a	Who will obtain informed consent or assent from potential trial participants or authorised surrogates, and how (see Item 32)	______12_______
	26b	Additional consent provisions for collection and use of participant data and biological specimens in ancillary studies, if applicable	______n/a______
Confidentiality	27	How personal information about potential and enrolled participants will be collected, shared, and maintained in order to protect confidentiality before, during, and after the trial	______5______
Declaration of interests	28	Financial and other competing interests for principal investigators for the overall trial and each study site	______19_______
Access to data	29	Statement of who will have access to the final trial dataset, and disclosure of contractual agreements that limit such access for investigators	______n/a______
Ancillary and post-trial care	30	Provisions, if any, for ancillary and post-trial care, and for compensation to those who suffer harm from trial participation	______n/a______
Dissemination policy	31a	Plans for investigators and sponsor to communicate trial results to participants, healthcare professionals, the public, and other relevant groups (eg, via publication, reporting in results databases, or other data sharing arrangements), including any publication restrictions	____2,15______
	31b	Authorship eligibility guidelines and any intended use of professional writers	______n/a______
	31c	Plans, if any, for granting public access to the full protocol, participant-level dataset, and statistical code	______n/a______
**Appendices**			
Informed consent materials	32	Model consent form and other related documentation given to participants and authorised surrogates	_____ n/a ______
Biological specimens	33	Plans for collection, laboratory evaluation, and storage of biological specimens for genetic or molecular analysis in the current trial and for future use in ancillary studies, if applicable	______n/a______

*It is strongly recommended that this checklist be read in conjunction with the SPIRIT 2013 Explanation & Elaboration for important clarification on the items. Amendments to the protocol should be tracked and dated. The SPIRIT checklist is copyrighted by the SPIRIT Group under the Creative Commons "Attribution-NonCommercial-NoDerivs 3.0 Unported" license.

Partners in Hope Medical Center

Identifying efficient linkage strategies for HIV self-testing (IDEaL)

Kathyrn Dovel, Principle Investigator
Partners in Hope
PO Box 302
3-6-2019

For peer review only

ABSTRACT

Background: HIV self-testing (HIVST) has been found to be a highly acceptable approach for men to learn of their HIV status and has resulted in increased testing uptake (Dovel 2019, cite Augustines stuff). However, rates of antiretroviral therapy (ART) initiation among those tested with HIVST are difficult to capture and some studies have suggested that linkage rates are low (Ortblad 2017, MacPherson 2014), particularly amongst men. We propose a clinical trial to test varying approaches to ART initiation among men who test HIV-positive through HIVST. We will test three interventions:

Lightest Touch Intervention (Arm 1): simple reminders to visit the health facility (given every two weeks);

Staged Intervention (Arm 2): a staged intervention that consecutively increases in intensity every month that a participant does not initiate ART (intervals include reminders, motivational interviewing, and home-based ART initiation);

Intensive Intervention (Arm 3): home-based ART initiation followed by linkage to the health facility of their choice the following month.

Objective: Our primary objective is to identify a cost-effective package for ART initiation among men identified as HIV-positive through HIVST in Malawi. Our specific objectives are:

Objective 1. Evaluate the effectiveness of the Staged ART Intervention vs Lightest Touch Intervention (primary analysis) and the effectiveness of the Staged ART Intervention vs Intensive Intervention (secondary analysis) on ART initiation within 4-months after enrolment in the trial.

Objective 2. Identify individual-, community-, and facility-level factors associated with ART initiation within each intervention arm (Lightest Touch; Staged; and Intensive Interventions).

Objective 3. Determine the cost and scalability of each intervention (Lightest Touch; Staged; and Intensive Interventions).

Methods: We will preform an individually randomized control trial with 543 HIV-positive men identified through HIVST and their female partners. Men will be individually randomized 1:1:1 to one of the three intervention arms described above. The study will be preformed at 10 health facilities suppored by Partners in Hope (PIH). Data collection will include baseline and follow-up surveys and interviews with men and women; medical charter reviews at four-months after study enrollment; qualitative interviews; and a cost analysis of costs associated with each arm. Participants will be enrolled in the study for a total of 4 months with approximately 2 or 3 study visits throughout that period.

Anticipated results: We anticipate learning about the most effective stragty to engage men in ART. We also anticipate learning about the type and degree of followup necessary to support men's engagement in ART services. Finally, we anticipate learning about the cost-effectiveness of intervention, with the goal of improving cost-effectiveness for the Ministry of Health. Results from this study could be used to define best practices and to further scale ART-focused programs for men in Malawi.

For peer review only

TABLE OF CONTENTS

1. INTRODUCTION ... 4

1.1. Background ... 4
1.2. Problem statement ... 4
1.3. Justification ... 4

2. OBJECTIVES ... 4

2.1. Primary objective ... 4
2.2. Secondary objectives ... 4

3. LITERATURE REVIEW ... 5
4. METHODOLOGY ... 8

4.1. Intervention description ... 9
4.2. Place of study ... 11
4.3. OBJECTIVE 1 ... 12

4.3.1. Study design ... 12
4.3.2. Target population ... 12
4.3.3. Sampling techniques and enrollment ... 13
4.3.4. Study Activites ... 13
4.3.5. Data collection techniques and tools ... 15
4.3.6. Sample size determination ... 17
4.3.7. Data analysis ... 17

4.4. OBJECTIVE 2 ... 17

4.4.1. Study design ... 17
4.4.2. Target population ... 17
4.4.3. Sampling techniques and tools ... 18
4.4.4. Data collection techniques and tools ... 18
4.4.5. Sample size determination ... 19
4.4.6. Data analysis ... 19

4.5. OBJECTIVE 3 ... 20

4.5.1. Study design ... 20
4.5.2. Data collection techniques and tools ... 20
4.5.3. Data analysis ... 20

5. ETHICAL CONSIDERATIONS ... 21
6. DISSMINATION OF RESULTS ... 22
7. PERSONNEL ROLES AND INSTITUTIONS ... 23
8. REGULATORY OVERSIGHT ... 24
9. STUDY IMPLEMENTATION ... 24
10. PROTOCOL DEVIATION REPORTING ... 25
11. WORK PLAN TIMELINE ... 25
12. BUDGET AND JUSTIFICATION ... 25
13. REFERENCES ... 27
14. APPENDIX ... 30

14.1. APPENDIX A – Study Invitation Cards ... 30

14.2. APPENDIX B – Recruitment and Screening Script 32
14.3. APPENDIX C – Written Informed Consent- Female 36
14.4. APPENDIX D: Written Informed Consent – Male 46
14.5. APPENDIX E: Baseline Survey – Male 55
14.6. APPENDIX F: Baseline survey – Female 100
14.7. APPENDIX G: Follow-up Survey - Male 129
14.8. APPENDIX H: Follow-up Survey – Female 137
14.9. APPENDIX I: Data Extraction Tool 144
14.10. APPENDIX J: In-Depth Interview Guide – Female 147
14.11. APPENDIX K: In-Depth Interview Guide – Male 155
14.12. APPENDIX L: Personel CV's 165

For peer review only

1. INTRODUCTION

1.1. Background

HIV self-testing (HIVST) is an effective strategy to improve HIV testing coverage, especially among hard-to- reach populations such as men and youth. Index testing, whereby a HIV positive client gives an HIVST kit to their sexual partner to use at home, is considered beneficial for its ability to maintain a testers privacy. The method is now recommended by the World Health Organization (WHO) and is being adopted as policy throughout sub-Saharan Africa (SSA). However, uptake of antiretroviral therapy (ART) and adherence after utilizing HIVST remains sub-optimal among certain population, specifically men. Innovative ART initiation and early retention strategies are urgently needed for Index HIVST to be successful.

1.2. Problem statement

We must better understand how to engage men in HIV care. Specifically, there is limited literature on feasible differentiated models to support men to start and stay on treatment that can be taken to scale. Further, there is increased recognition that individuals are at greatest risk of loss-to-follow-up during transition periods across the cascade (i.e. when starting ART). ART initiation and early retention must be improved if HIVST is to become a viable option for high-risk groups in SSA. To address this gap, we propose to conduct a study to test and evaluate varying strategies for ART initiation and retention amongst men.

1.3. Justification

This study will combine HIVST with a second-level intervention focused on ART initiation to address the urgent gap in ART initiation and early retention among HIVST users. Additionlly, Objective 3 will allow us to develop the lowest cost intervention package while reaching the highest number of male partners.

2. OBJECTIVES

2.1. Primary objective

Objective 1. Test the impact of a staged ART intervention vs simple reminders and the effectiveness of a staged ART intervention vs home-based ART on ART initiation within 3-months of an HIV-positive diagnosis

2.2. Secondary objectives

Objective 2. Identify individual-, community-, and facility-level factors associated with ART initiation within each intervention.

Objective 3. Determine the cost and scalability of each intervention.

For peer review only

3. LITERATURE REVIEW

Background

Men in sub-Saharan Africa are less likely than women to use HIV services.[1] Men's absence from care is concerning not only for their own health, but also for the health of girls and young women who continue to be infected at unacceptably high rates.[2] HIV prevention and treatment programs have not traditionally been directed at men. Men are notably absent from international guidelines, national policies, and local HIV interventions. Research shows that women are 322% more likely to be mentioned in international HIV guidelines than men.[3] In the context of Malawi, national guidelines expect women of reproductive age to attend a health facility 5-17 times per year (equivalent to 19-63 hours)[4], and 180-472 times in their reproductive lifespan (15-44 years). There are no such expectations for men (see Table 1). The justification for the global attention of HIV program thus far on women and girls is without dispute. Gender inequality is a key driver which impacts women's health and access to HIV services and creates specific vulnerabilities for women to HIV infection.[5] However, framing HIV as a woman's concern means we have failed to understand how gender affects and drives the burden of ill health for men, and inadvertently perpetuates the epidemic for young women and girls. Targeted strategies specific to men are urgently needed if we are to engage them in care.

Table 1: *Malawi ministry of health recommended health services and estimated visits required across the reproductive life span (15-44 years)*

			Estimated number of visits between 15-44 years			
Service	Frequency	Target Population	Women: 5-year FP (Implant; 9%*)	Women: quarterly FG (injectables; 23%*)	Women: monthly FP (pills, 2%*)	Men
ANC	17.6	Women	18	18	18	-
Delivery	4.4	Women	4	4	4	-
Post-natal	4.4	Women	4	4	4	-
Family Planning	88	Women	7	88	264	-
Under five	120	Women	120	120	120	-
HIV testing	22.6	Women and men	23	23	23	29
Circumcision	3	Men	-	-	-	3
**Total**			**176**	**257**	**433**	**32**

Male partners of women who are already identified as HIV-positive (index partners) are still a major concern for epidemic control due to high rates of multiple and concurrent partnerships among men[6] and the fact that index male partners have two times the risk of being HIV-positive as compared to the general male population.[7] Data from a recent HIVST study show that there is a high need for index testing among men in Malawi: across 3 high-burden district hospitals in Malawi, men represented 73% of all index partners in need of testing. Among those who did test, male partners were 4 times more likely to test HIV-positive than female partners (23% versus 3%), representing urgent unmet need among men.[8]

A recent Index HIV Trial in Malawi found that HIV testing among men increased dramatically when HIV-positive clients give HIVST kits to their sexual partners to use at home.[9] The study found that 66% of male partners in the HIVST arm tested for HIV compared to only 22% of men in the standard partner referral slip arm. Within the HIVST arm, men who tested for HIV had an HIV-positivity rate of 23%, with no adverse events reported (see Fig 1).[2] Index HIVST is highly acceptable and allows men to test at times and locations convenient for them, with complete privacy in their own homes.[10,11]

However, innovative ART initiation and early retention strategies are urgently needed for Index HIVST

to be successful. The aforementioned study showed that ART initiation was unacceptably low, with only 22% of HIV-positive men in the HIVST arm initiating ART at 6-months versus 75% of men in standard partner referral slip arm).[3] (See Figure 1). Poor rates of ART initiation are commonly reported across most HIVST studies, with ART initiation rates ~20-45%[7,12–14], although ART initiation is notoriously difficult to measure within HIVST strategies. A cost analysis for national scale-up of Index HIVST in Malawi showed that 76% of men tested must initiate ART for Index HIVST to be cost-neutral at the national level as compared to using partner referral slips.

Figure 1: *Male partner use of HIV services Index HIVST vs. referral slips (n=285) from HIVST trial (PI: Dovel)*

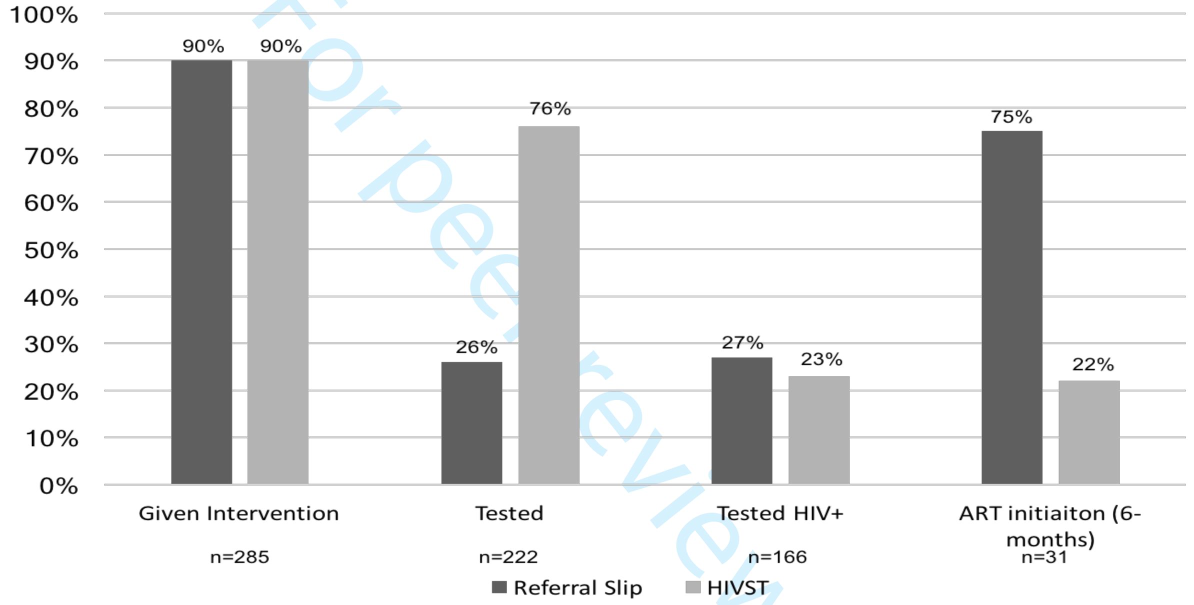

Two overarching barriers keep HIV-positive men from accessing ART services: (1) lack of male-friendly services;[15–17] and (2) harmful gender norms.[18–20] Male friendly services are private and convenient (requiring minimal time), and offered by health workers who understand the unique needs of men.[21] In addition, men are often unfamiliar with the health system, and are unsure how to navigate facility-based services. Gender norms that prioritize men as strong and self-reliant perpetuate fear of unwanted disclosure and stigma, and discourage men's engagement in ART.[18,21] Our research in Malawi found similar barriers to ART initiation for men who tested HIV-positive: men avoided ART services due to (1) fear of unwanted disclosure and stigma due to lack of privacy; (2) time/cost required to access care; (3) poor knowledge about the benefits of early ART initiation; and (4) beliefs that require men be strong, in control, and focused on short-term benefits such as daily financial earnings and respect from their male friends. Index HIVST must be combined with innovative ART interventions that address these barriers.

Evidence based for interventions that increase ART initiation

We have conducted a thorough search of the literature and have identified several intervention strategies that may increase ART initiation among men who use HIVST: (1) reminders + peer navigation; (2) motivational interviewing; and (3) home-based ART.

Reminders + Peer Navigation is shown to help clients overcome fears about facility-based services and

For peer review only

provide peer modeling how to live successfully with HIV.[22] While the strategy has been primarily tested within traditional HIV testing strategies, we hypothesize that the same mechanisms will work for men who test through Index HIVST. Reminders are usually done over the phone via phone calls or SMS and can vary in frequency based on the health care workers disgression. Peer Navigation is assisted guidance to the health clinic as well as overviews of where to go/what to do when at the facility once there to ensure men feel more comfortable in the clinic environment.

Motivational Interviewing is becoming widely recognized as a key strategy to help clients navigate barriers to the desired outcome by building client's self-efficacy, identifying internal motivation for the desired behavior, and establishing strategies and short- and long-term goals needed to reach a desired outcome.[8,23] Motivational interviewing is seen as particularly effective when clients need to make difficult decisions and overcome multi-level barriers to behavior change.[24,25] The strategy has been used to improve ART adherence[8,25] and reduce sexual risk behavior.[15]

In contrast, traditional counselling efforts are largely informational and directive, whereby health care workers deliver a pre-determined counseling package that is not responsive to a client's individual situation.[16,17] Such methods have been proven largely ineffective,[18] particularly with hard-to-reach populations such as men.[19] Motivational interviewing differs from traditional strategies by adopting a client-centered approach is based on collaboration, evocation and respect for autonomy. We hypothesize that these counseling techniques will encourage HIV status acceptance and disclosure, promote health seeking behavior, provide coping strategies men need to overcome barriers related to facility-based care, and ultimately, facilitate ART initiation.

Furthermore, motivational interviewing and client-centered care should resonate with and address the needs of men. Partners in Hope Malawi conducted 25 interviews with men and 6 focus group discussions with health care workers and female partners (n=42) to assess what health services men desired. Overwhelmingly, men reported wanting increased counseling on sexual health (including HIV) and marital concerns. Exit surveys with male ART clients (n=180) show that only 38% of men were aware of Treatment as Prevention and 65% aware of the benefits of early ART initiation, highlighting major knowledge gaps that may influence engagement in care. Motivational interviewing and client-centered counseling will be able to address both gaps in ART treatment and sexual health knowledge.

Home-based ART initiation has improved ART initiation across the region. A systematic review found that home-based ART is associated with ART retention, decreased mortality,[26] and in some cases, reduced stigma and increased privacy.[27,28] We conducted one of the only studies to examine home-based ART initiation within a community HIVST distribution strategy (Co-I: Choko). We found that home-based ART initiation alongside home-based HIVST significantly increased ART initiation as compared to standard facility-based initiation (RR 2.94; p-value<0.001).[13]

Home-based ART may be particularly attractive to hard-to-reach men because it reduces client time required to access services and provides an easy, opt-out entry point for men who otherwise may have never engaged with the health care system, or know how to navigate complicated, busy health facilities. Home-based ART has been associated with a three-fold reduction in financial costs to clients.[29] Further, home-based ART facilitates client-centered, one-on-one care that is often not feasible in busy clinic settings.

The Malawi Ministry of Health is in the process of rolling out community-based ART distribution strategies, and may consider home-based ART initiation for hard-to-reach populations. However, home-based ART is considered a resource intensive strategy, and therefore should (1) only be offered to the hardest-to-reach populations, and (2) requires that clients who initiate ART at home-based eventually link into facility-based care. Additionally, findings from a recent Index HIVST Trial show that home-based services for men is acceptable to female ART clients in the Malawian context, with minimal risk of adverse events. Over 90% of female ART clients had disclosed their HIV-status to their partner and were willing to have their male partners traced in their homes for additional services.

For peer review only - http://bmjopen.bmj.com/site/about/guidelines.xhtml

Finally, increased privacy and decreased wait-times are essential if men are to engage in HIV services. As part of a study on new Universal Treatment policies in Malawi, 15 in-depth interviews and 208 surveys were conducted with newly diagnosed HIV-positive men. Fear of unwanted disclosure due to limited privacy and a lack of trust in the health facility were the primary barriers to men's ART initiation. Home-based ART initiation can help address these barriers for ART initiation, and motivational interviewing can help provide men the skills needed to navigate these barriers within the health system in order to promote ART retention. Further, the vast majority (>95%) of men who used HIVST in the Index HIVST trial disclosed their HIV status to their female partner[8], meaning that home visits (i.e., reminders, peer navigation, motivational interviewing, or home-based ART) will not increase risk of unwanted disclosure to one's sexual partner. Table 2 below outlines how the proposed interventions will address barriers to ART initiaiton identified in the literature.

Table 2. *Levels and specific barriers to men's ART initiation, and intervention components*

<table>
<tr><th>Barriers to ART Initiation</th><th>Intervention Components</th><th>Level of Intervention</th></tr>
<tr><td>Unfamiliar with health system</td><td>Reminders and Peer Navigation</td><td>Health System</td></tr>
<tr><td>Belief about gender norms and focus on short-term benefits</td><td rowspan="2">Motivational Interviewing</td><td rowspan="2">Community/Individual</td></tr>
<tr><td>Poor knowledge</td></tr>
<tr><td>Time/cost requirements</td><td rowspan="3">Home-Based ART Initiation</td><td rowspan="3">Health System</td></tr>
<tr><td>Lack of privacy/fear of disclosure</td></tr>
<tr><td>Unfamiliar with health system</td></tr>
</table>

4. METHODOLOGY

This study will be an individually randomized trial comparing three dfferent strategies to improve ART initiation and early retention among men who test HIV-positive with HIVST. Study staff will utilize the Minsitry of Health Index Testing Register and trace HIV positive women and their male partners to be screened and enrolled if they meet the inclusion criteria. Enrolled men will be randomized to one of three arms and will receive follow up and varying degrees of support based on the arm assigned. Outcomes will be assessed after 90 days after enrollment. Survey data, qualitative data, medical chart data (Objectives 1 and 2) and costing data (Objective 3) will be collected over years 1-3. A study flow chart is illustrated below (Figure 2).

For peer review only

Figure 2: *Study flow chart*

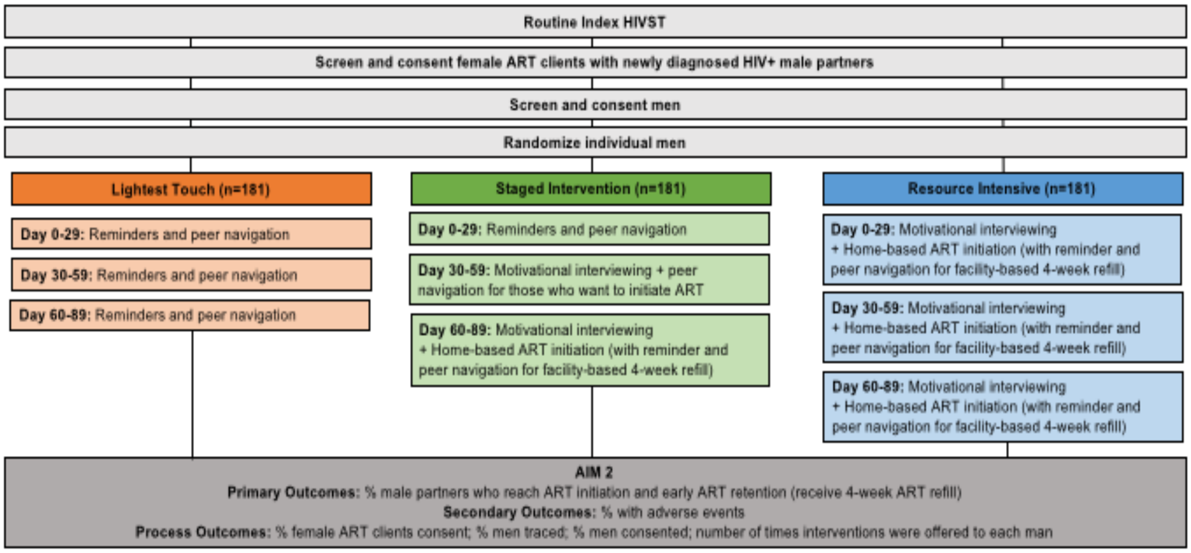

4.1. Intervention description

Each arm will offer an intervention immediately after study enrollment (that same day; day 0). Follow-up interventions will be offered every 14 days after that until 76 days or ART initiation is reached, whichever comes first.

Description of study arms:

Arm 1: "Lightest Touch" Intervention, whereby facility staff provide reminders and peer navigation (SMS and home visits) for men to encourage enrolment in facility-based ART programs. One reminder on the day of enrollment and every 14 days thereafter, until 76 days or ART initiation, whichever comes first. If initiation is not reached at 90 days, the patient will be classified as not initiated for study purposes. See Arm 1 diagram below:

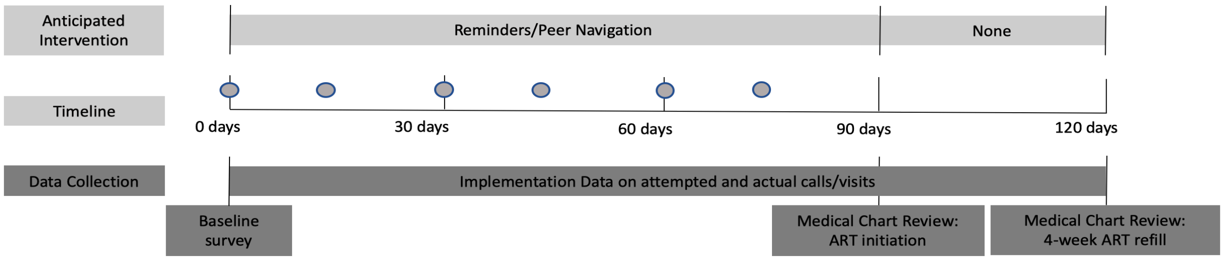

Fig 2. Timing of interventions to be offered in Aim 1

Legend:

Approximate time the intervention will be offered (approximately every 2 weeks until 76 days, or until ART initiation, whichever comes first

Arm 2: "Staged" Intervention, whereby the intervention will build in intensity each month for those

who have not initiated ART in the previous month, or for a maximum of 3 months, whichever comes first. The following intervention components that will be added each month (incrementally) until the first ART distribution is completed:

- Day 0-29: Reminders and, for those who agree to initiate, peer navigation;
- Day 30-59: Motivational interviewing and, for those who agree to initiate, peer navigation;
- Day 60-79: Motivational interviewing + home-based ART initiation and, for those who initiate, reminders and peer navigation for the facility-based 4-week ART refill appointment).

See Arm 2 diagram delow:

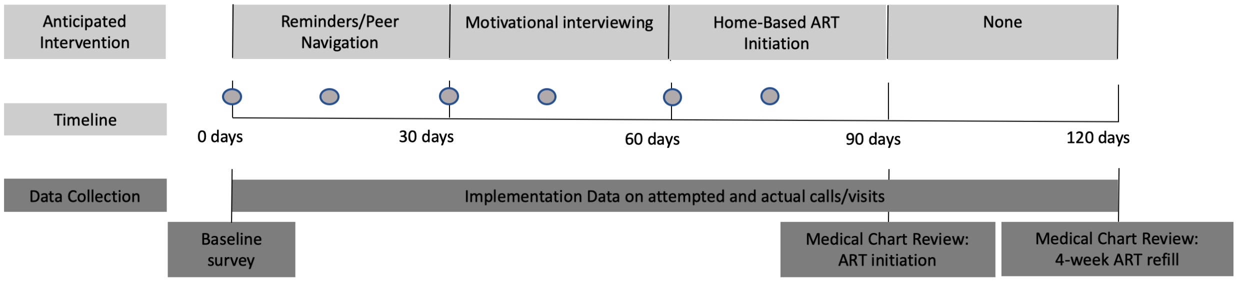

Fig 3. Timing of interventions to be offered in Aim 2

Legend:
Approximate time the intervention will be offered (approximately every 2 weeks until 76 days, or until ART initiation, whichever comes first

Arm 3: "Intensive" Intervention, whereby the most resource intensive intervention is offered immediately to all HIV-positive male partners. See Fig 4 for timeline. Components include:

- Motivational interviewing
- Home-based ART initiation
- 4-weeks after ART initiation: Reminders and peer navigation to facility for 4-week ART refill appointment

Home-based ART initiation will be scheduled at times convenient for men, including evening and weekend hours. Men who prefer to initiate in another private location in the community (besides their home) will be able to do so. The first home-visit will be conducted by a trained nurse and will include confirmatory HIV testing using Ministry of Health standard algorithm (Determine + Unigold), pre-ART counseling and motivational interviewing, a basic health evaluation, and ART initiation with a 30-day supply of first-line ART in Malawi – dolutegravir, tenofovir, and lamivudine as a single tablet. Clients will also be given a 30-day supply of cotrimoxazole, which is standard of care for all HIV-positive individuals.

Prior to ART initiation, a basic health evaluation will be performed by the nurse, including screening for tuberculosis with routine questions.[66] Any individual identified by the study nurse with concerns for an active opportunistic infection or other health problem(s) that could complicate home-based ART will be immediately referred and escorted to the facility.

At the same visit, motivational interviewing will be performed in preparation for men to engage in facility-based ART services. This includes counseling on the benefits of early ART, strategies for disclosure and positive living, strategies to overcome facility-based barriers to ART services, and addressing harmful gender norms that may discourage men from using care. Counseling will be adaptive to the needs and concerns of male clients. At 4-weeks after ART initiation, an expert client will escort the

man to a nearby facility of his choice to join the facility-based ART cohort. Peer navigation will be provided to ensure men become familiar with the facility-based program. Men who wish to attend a facility that is not nearby will be linked with a male counselor from the selected facility. After completing all facility-based ART services for that day, the male partner will receive additional client-centered counseling with the same counselor to discuss the experience, benefits and challenges associated with facility-based ART, and strategies to overcome barriers. See Arm 3 diagram below:

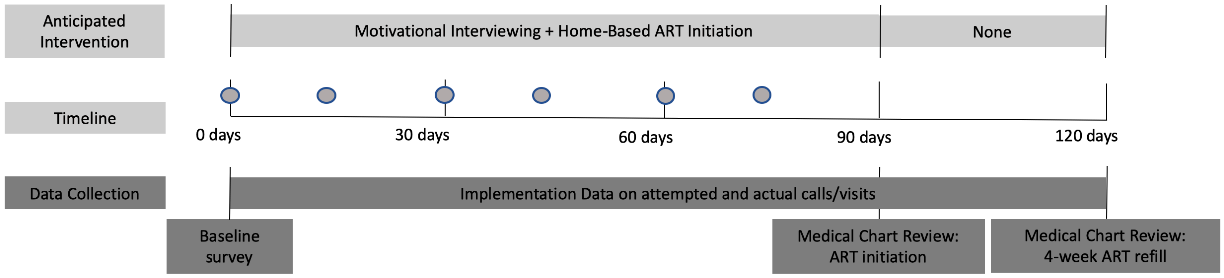

Fig 4. Timing of interventions to be offered in Aim 3

Legend:
○ Approximate time the intervention will be offered (approximately every 2 weeks until 76 days, or until ART initiation, whichever comes first

4.2. Place of study

All study activities will take place in 10 Partners in Hope supported facilities within the Lilongwe and Chikwawa districts, representing 19,198 adult female ART clients across all sites. These districts were chosen because they are priority districts for the Presidents Emergency Plan for AIDS Relief (PEPFAR) and have the highest HIV prevalence and unmet need of all Partners in Hope supported districts. See Table 3 for the selected 10 health facilities.

Table 3: *Selected study sites*

District	Facility name	ART Cohort Size
Chickwawa	St. Monftord Mission Hospital	3533
Chickwawa	Kalemba Community Hospital	2087
Chikwawa	Chickwawa District Hospital	4898
Kasungu	Kasungu District Hospital	5942
Lilongwe	Nkhoma Community Hospital	2166
Lilongwe	Mponela Rural Hospital	1099
Lilongwe	Daeyang Luke Hospital	1594
Nkhotakota	Nkhotakota District Hospital	5361

For peer review only - http://bmjopen.bmj.com/site/about/guidelines.xhtml

Nsanje	Nsanje District Hospital	3904
Nsanje	Ngabu Rural Hospital	1237

Specific methodology for each Objective is described below:

4.3. OBJECTIVE 1

Evaluate the effectiveness of the Staged ART Intervention vs Lightest Touch Intervention (primary analysis) and the effectiveness of the Staged ART Intervention vs Intensive Intervention (secondary analysis) on ART initiation within 4-months after enrolment in the trial.

- **Hypothesis 1.1:** 25% of men will initiate ART with simple reminders compared to 45% with motivational interviewing
- **Hypothesis 1.2:** 65% of men will initiate ART with home-based ART initiation compared to 45% with motivational interviewing

4.3.1. Study design

We will conduct an individually randomized controlled trial at 10 high-burden facilities in Malawi.

4.3.2. Target population

We will enroll 543 HIV-positive men identified through routine Index HIVST strategies and their female partners (1,086 participants total). While men are the primary focus of the study, female partners will be enrolled in order to understand their perception of their male partners use of ART services, acceptability of the intervention, and any unintended outcomes or adverse events.

Eligibility Critiera

Female ART clients will be enrolled in order to conduct baseline and follow-up surveys to understand their perception of their male partners use of ART services, acceptability of the intervention, and any unintended outcomes or adverse events.

Female Partner

- Inclusion criteria include: (1) client and partner are ≥15years of age; (2) partner lives in facility catchment area; (3) partner tested HIV-positive and has not initiated ART; and (4) ART client reports no interpersonal violence (IPV) as defined by WHO with their current sexual partner in the past 12 months.

- Exclusion criteria include: (1) client and partner are <15years of age; (2) partner does not live in facility catchment area; (3) partner has not tested HIV-positive or has testing positive and has initiated ART; and (4) ART client has reported interpersonal violence (IPV) as defined by WHO with their current sexual partner in the past 12 months.

Men will be enrolled as the primary recipient of the intervention.

Male partner

- Inclusion criteria include: (1) self and partner ≥15 years of age; (2) live in the facility catchment area (i.e., in the past 30 days, has spent ≥50% of all nights in the village); (3) has tested HIV-positive and has not initiated ART;

For peer review only

- Exclusion criteria include: (1) self and partner <15 years of age; (2) does not live in the facility catchment area (i.e., in the past 30 days, has spent <50% of all nights in the village); (3) has not tested HIV-positive or has tested HIV-positive and has initiated ART

4.3.3. *Sampling techniques and enrollment*

Sampling, screening, and enrolling male partners will be embedded within routine Index HIVST strategies.

Brief description of Routine MOH HIVST Guidelines

Briefly, routine Index HIVST includes three steps:

- *Identify ART clients with sexual partners in need of Index HIVST:* as outlined in the Malawi guidelines within the health care facility.
- *Distribute Index HIVST:* ART clients with a partner of unknown status will receive standard Index HIVST kit. They will be provided a demonstration, counseling, and an overview of risks and benefits.
- *Follow-Up on Index HIVST Use:* During their next ART appointments, ART clients are asked about HIVST distribution, use, and result of the HIVST kit, along with male partner linkage to a health facility for those who tested HIV-positive (i.e., confirmatory HIV testing and ART initiation). Data are documented in the Index HIVST register.

Once routine Index HIVST activities are completed, study recruitment and enrollment will commence. See Figure 3 for a complete description of particiant enrollment and study activities.

Figure 3: *Study screening and enrollment procedures flow chart*

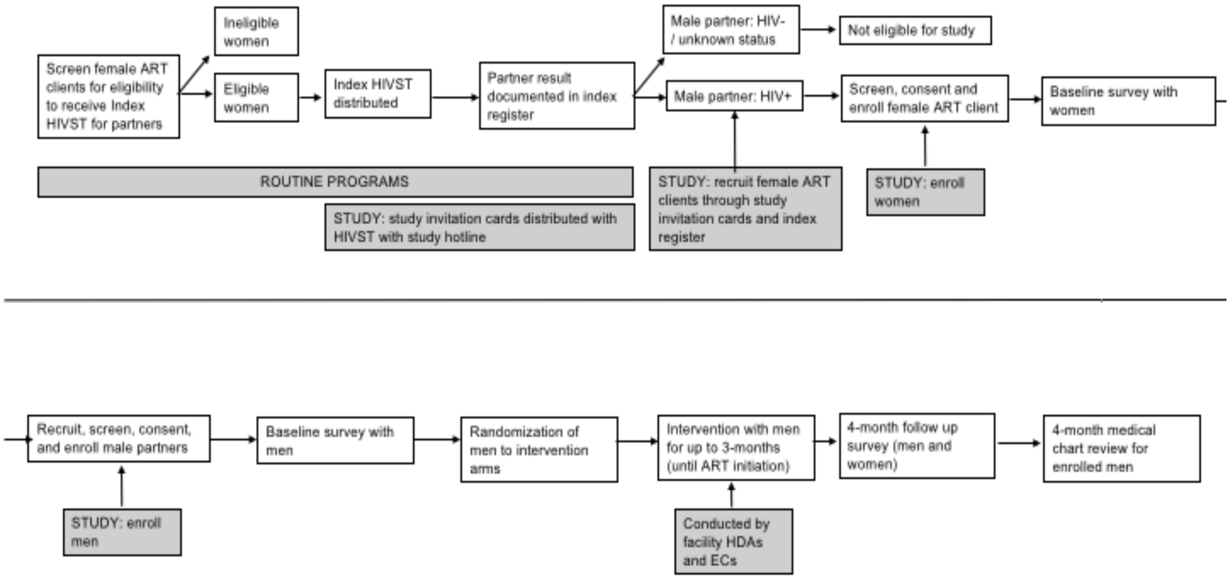

4.3.4. *Study Activites*

Female ART client recruitment

To maximize the number of male partners available for enrollment, the study team will introduce study invitation cards as part of Index HIVST activities and include them with HIVST kits distributed as a part of routine Index HIVST procedures (described above). Study invitation cards will include a ‘hotline’ phone number and will not provide any HIV specific information to ensure confidentiality. (see Appendix A for study invitation card). Female partners will be informed that should their male partner be found HIV-positive, they and their partner may be eligible for a study and should call the hotline number of the invitation card to receive more information.

In addition, the study team will review the Index Testing Register on a regular basis to identify female ART clients who reported HIV-positive male partners through routine Index HIVST procedures. Female ART clients identified will be contact by routine facility staff to be informed about the study and refered to study staff if interested in enrollment.

Female ART client enrollment

Female partners interested in the study will provide oral consent to participate in study screening and be screened for eligibility for the study (Appendix B). Study staff will conduct all consent and screening activities. If eligibile and willing to participant, written informed consent will be obtained (Appendix C). Female partners will be enrolled even if their male partner (1) does not consent to study participation (2) cannot be traced.

Upon enrollment in the study, female ART clients will work with study staff to make a plan for inviting male partners to enroll in the study. Baseline surveys with female ART clients will be conducted that same day or on a day convenient for the female ART client. Women whose partners agree to participate in the study will be followed up after 4-months for a follow up survey regarless of the outcome of their partner (ie loss-to-follow-up).

Male partner recruitment

Once female ART clients are enrolled, male partners will be recruited, screened, and consented. Study staff will work with the female ART client to establish a recruitment plan that is acceptable and feasible for the women. This may include women referring male partners to the study staff, study staff actively recruiting male partners based on information provided by the female client, or a joint approach where the study staff approaches male partners with the female ART client. Each female ART client enrollled in the study will be allowed to choose the recruitment strategy that best fits her individual situation and the needs of her partner.

Male partner enrollment

Men found eligibile and willing to participante, will provide written informed consent (Appendix D) and will be randomized to one of the three study arms. Randomization will occur using an electronic randomization system on tablet devices. A baseline survey will be concuted immediately following consent and the intervention will be carried out over the course of 3 months. Men will be contacted by the study staff 4-months after enrollment for a follow-up survey.

Randomization

Randomization will be conducted after completion of the baseline survey with male participants. Study staff will show randomization results as a picture on a pre-programmed tablet, allowing the participant to view the results themselves in order to maximize transparency and study buy-in. Male participants will be randomized to 1 of three arms and will be randomizied 1:1:1.

For peer review only

Intervention

Interventions will be offered immediately after study enrollment (that same day or the closest day that is convenient for the male participant). Follow-up interventions will be offered every 14 days after enrollment until 76 days after enrollment in the study or ARTinitiation is reached, whichever comes first. See a full description of the intervention in the Intervention Description section (4.1).

Intervention arms include:

1. ***Lightest touch arm:*** reminders and peer navigation to facility-based ART services
2. ***Staged arm:*** intervention builds in intensity each month for those who have not initiated ART in the previous month. Strategies include reminders and peer navigation, motivational interviewing, and home-based ART initiation
3. ***Intensive arm:*** home-based ART initiation + motivational interviewing + peer navigation to facility-based ART services for their 4-week follow-up appointment

Male expert clients and male nurses will complete all intervention activities to ensure it is as close to real-world implementation as possible (not implemented by Research Assistants). Research Assistants will support facility staff to ensure men are given the appropriate intervention (based on randomization arm), and that all staff activities related to the intervention are documented (i.e., how many reminders were given to each client, ect.). Intervention monitoring and evaluation tools will be developed and incorporated into the facility staff daily routine. Weekly reviews of all intervention monitoring tools and planning for the following week will be completed with study expert clients, nurses, and Research Assistant to ensure adherence to the study protocol. The Study coordinators and PI's will be highly involved throughout the implementation process to ensure protocol adherence.

4.3.5. Data collection techniques and tools

Data collection tools will include:

- *Baseline Survey:* Research assistants will administer baseline surveys with both female ART clients and male partners immediately following enrollment (before randomization). Surveys will collect data on male and female demographics, sexual partnerships and couple dynamics, and men's history with health services, and HIV services specifically. (Appendix E & F)

- *Follow-up Survey:* Research assistants will administer follow-up surveys with female ART clients and male participants 4-months after enrolment in the study. Follow-up surveys with men will assess the primary outcome of interest (ART initiation and completion of 4-week follow-up appointment), acceptability of the intervention, and any adverse events (ie., unwanted status disclosure). Men who cannot be reached will be counted as failures for true ART initiation. Follow-up survyes with female ART clients will assess acceptability of the intervention and any adverse events (i.e., IPV, end of the relationship, or unwanted disclosure) associated with intervention procedures. (Appendix G & H)

- *Medical Chart Reviews:* Identifiers will be collected for all men enrolled in the study, including name, age, village and address, and phone number. Identifiers will be used to conduct medical chart reviews at 4-months after enrollment as another measure of ART initiation (attendance to the 4-week follow-up ART appointment). Facility staff (established data clerks employed by Partners in Hope) will review medical records at study facilities and all other Partners in Hope

supported facilities within participating districts (61 facilities in total) to account for men who engage in ART outside study facilities. We successfully used this method in other HIVST studies to capture ART initiation.[3] Male partners who are not found in medical chart reviews will receive a follow-up home-visit to confirm ART outcomes through review of their individual medical record book (health passport) and self-reporting in the event that there are gaps in the record. Men who cannot be reached will be counted as failures for true ART initiation. (Appendix I)

- *Process Implementation Data:* Expert clients, nurses, and research assistants will keep daily logs as part of study monitoring and evaluation tools in order to assess the implementation of the intervention for each participant. Primary events to be recorded in the daily logs are: (1) unable to reach participant (and reason); (2) contacted participant; (3) intervention provided (and notes about the challenges and successes of the interaction; and (4) other comments relevant to intervention implementation. Each event will be recorded with a corresponding date.

Primary and secondary outcomes are measured through medical chart reviews and follow-up surveys (see Table 4).

Table 4: *Study Measures for Objective 1*

Outcome	Measurement	Source
Primary Outcomes		
Early ART Retention	Proportion of men who initiate ART at 3-months and attended their 4-week ART refill appointment at 4-months after enrollment	Medical chart review at 4-months
Secondary Outcomes		
ART initiation	Proportion of men who initiate ART at 3-months after enrollment	Medical chart review at 3-months
Adverse events by female ART client (IPV, unwanted disclosure, end of relationship) or male partner (unwanted disclosure)	Self-report from female ART client and their male partners who were identified as HIV-positive	Follow-up surveys at 4-months
Process Outcomes		
Proportion of female ART clients who consent	Proportion of eligible ART clients who consent to the participate in the study	Process implementation data
Proportion of men traced	Proportion of men who were successfully traced within 3-months after female ART client is enrolled	Process implementation data
Proportion of men who consented	Proportion of eligible men who consent to participate in the study	Process implementation data

- *Sensitivity analyses for men excluded from the trial***:** We recognize that men's consent to participate in the study may bias the sample enrolled in the study. There are two groups that we may not be able to include in the main study: (1) men we are unable to trace/contact (herein referred to as "unreachable men") and (2) men we are able to reach but who refuse to participate in the main trial (herein referred to as "male refusers"). We will take two approaches to address this potential bias
    - Unreachable Men*:* We will collect data on men who are unreachable via their female partner. Female partners for these men will complete a brief survey regarding Surveys will collect data on male and female demographics, sexual partnerships and couple dynamics, and men's history with health services, and HIV services specifically (as reported by female partners). We successfully used similar methods in the Index HIVST Trial.
    - Male Refusers*:* Men who are contacted but do not consent to the trial will be consented for a one-time survey immediately following refusal for the larger study. The same data will be collected, as described above.

4.3.6. Sample size determination

We powered the study to detect differences in ART initiation between Lightest Touch and Staged Interventions at 4-months after enrollment (primary outcome). We also assured we were powered to detect differences in ART initiation between Staged Interventions and the Intensive Intervention. We Assume that 25% of men in the Lightest Touch arm, 55% in the Staged Intervention arm, and 75% in the Resource Intensive arm initiate ART at 3-months and attend their 4-week follow-up appointment at 4-months. Any man lost to follow-up in any arm will be treated as failures for the outcome evaluation. The sample size needed to detect this difference with the power of 0.8 is 181 men per arm. The calculation is based on asymptotic normality of log odds ratio. We need to enroll and randomize a total of 543 HIV-positive men. Assuming that 25% of women have partners of unknown status, 65% of male partners will use the HIVST kit, 25% of them will be HIV-positive, and 80% of them will enroll in the study, we will need to screen over 3,000 women who were given index HIVST to reach the required sample size.

4.3.7. Data analysis

All randomized men will be included in the analysis of primary outcomes; men with missing outcome assessment due to loss to follow-up will be treated as outcome failures. All primary outcomes are binary; they will be analyzed by logistic regression models with intervention as a predictor, adjusted for baseline socioeconomic and demographic variables. We will conduct sensitivity analyses to account for men who we were never able to contact (unreachable men) and men who refused to participate in the full trial (refusers). We will run several analyses whereby the denominator includes (1) unreachable men and refusers; and (2) refusers.

4.4. OBJECTIVE 2

Identify individual-, community-, and facility-level factors associated with ART initiation within each intervention arm (Lightest Touch; Staged; and Intensive Interventions).

- **Hypothesis 2.1***:* In quanitative data, older men, men without strong social support networks, men with high levels of internalized and perceived HIV-related stigma, and men who hold rigid beliefs of gender norms and men's role as the provider and decision maker of the home will be less likely to initate ART.
- **Hypothesis 2.2***:* In qualitative data, primary factors influencing men's decision to start ART will be perceptions of feeling healthy, perceptions of one's ability to continue working and providing for their family without ART initiation, and perceptions of HIV-related stigma within one's community.

4.4.1. Study design

We will use baseline survey data from the randomized trial (Objective 1) to identify factors associated with ART initiation among men. We will also conduct 200 semi-structured in-depth qualitative interviews with a random sub-set of enrolled men (n=100) and their female partners (n=100) to assess in-depth characteristics of men who fail to engage in care, contextualize decisions around ART initiation and retention, and understand additional strategies that may be needed for male partners to successfully initiate and be retained in ART programs.

4.4.2. Target population

All men and women enrolled in the overarching trial will compelte a baseline survey. Eligibility criteria for study enrollment is described in detail under Objective 1.

A subset of men and women enrolled in the overarching trial will be randomly selected to complete an in-depth interview. Eligibility criteria for in-depth interview are as follows:

For peer review only

Male partners

- Inclusion criteria include: (1) randomly selected using electronic random selection techniques; (2) linked to care within 4-months after enrolling in the study (defined as completing the 4-week ART refill appointment) (n=50 respondetns); or (3) did not link to care within 4 months (n=50 respondents)
- Exclusion criteria include: (1) not randomly selected using electronic random selection techniques

Female partners

- Inclusion criteria include: (1) randomly selected using electronic random selection techniques; (2) partners could never be traced for study enrollment; or (3) partners were enrolled but were lost to follow up and unable to be reached again
- Exclusion criteria include: (1) not randomly selected using electronic random selection techniques; (2) partners could be traced for study enrollment; or (3) partners were enrolled and were not lost to follow up and were able to be reached again

4.4.3. Sampling techniques and tools

Survey data will include all men and women enrolled in the study (n=1,086; described in detail in Objective 1). 100 men (~ 33 per study arm) and 100 women (~ 33 per study arm) enrolled in the study will be randomly be selected for in-depth interviews.

4.4.4. Data collection techniques and tools

Surveys: Baseline and 4-month follow-up surveys will be conducted with men and women enrolled in the trial. They will focus on:

- *Health care system*: perceptions regarding the following aspects of ART services (i) privacy and confidentiality; (ii) availability of services; (iii) wait-time and distance to facility; (iv) quality of care and rude behavior from health care providers, using validated measures.
- *Sociodemographics*: (i) age; (ii) household assets; (iii) work; and (iv) substance use
- *Couple characteristics*: (i) relationship type and length; (ii) sexual activity and risk; (iii) frequency of communication; (iv) disclosure; (v) joint decision making using standard measures from Demographic Health Survey (DHS);[17] (vi) gender norms using validated Gender Equitable Men (GEM) Scale,[67] and (v) Revised Conflict Tactics Scale.[68]
- *Knowledge/perceptions and biomedical factors:* (i) knowledge about HIV and ART (treatment as prevention, benefits of early ART); (ii) risk perception (morbidity and mortality); standard DHS measures on (iii) previous use of HIV services and (iv) self-rated health;[17] and (v) WHO staging at enrollment.

In-depth interviews: Table 5 below describes in-depth interview participants and justification. In-depth interview guides for men and women are developed based on existing literature and our extensive experience conducting in-depth interviews with this population (Appendix J and K). Female partners will provide important insight into the circumstances of these men, and potential strategies to more effectively reach them. Men's qualitative feedback is particularly important for the staged intervention and intensive intervention since these are fairly novel and under explored.

For peer review only

Table 5. *Description of in-depth interview participants and justification*

Participant type	Number of interviews	Justification
Women whose male partners were **unreachable** during the study enrollment	50 (~ 16 per arm)	To understand the couple dynamics and charachteristics of men who were never treaced and additional strategies to reaching these men
Women whose male partners were **loss-to-follow-up after** study enrollment	50 (~ 16 per arm)	To understand the couple dynamics and charactersitcs of men who were loss-to-follow-up, and additional strategies to better engage these men in care
Male particiapnts who **did not** complete 4-week ART refill appointments by 4-months	50 (~ 16 per arm)	To understand what they liked and did not like about the intervention, why they did not link to ART, and suggestions on how to improve the intervention
Male participants who completed 4-week ART refill appointments by 4-months	50 (~ 16 per arm)	To understand what they liked and did not like about the intervention, why they linked to ART, and suggestions on how to improve the intervention

4.4.5. Sample size determination

Survey sample size details provided in Objective 1. The number of interviews required for qualitative data can be challenging to predict. Data should be collected until saturation is reached, meaning that no new themes or relevant information is emerging. The exact number of interviews required to reach saturation differs based on the aim of the study, the diversity in respondents, and the theoretical framework used for analysis.[30] However, a basic rule of thumb is that no sample size should be under 25 participants in order to reach saturation and identify all relevant themes or new information important to the study.

4.4.6. Data analysis

Surveys: Subjects with complete data in outcomes as well as predictors will be included in the analysis. We will calculate descriptive statistics, including mean/median, variation (standard deviation, kurtosis), range, and frequency distributions for the demographic and clinical characteristics, overall and by study arm. Logistic models will be developed for the probability of a positive outcome, with sociodemographic factors included as covariates in a suitable form (linear/spline/factor). Differences in the prevalence of each of the outcomes of interest will be examined by study arm as well as by other factors of interest including demographic characteristics (e.g., age), couple chararisterics, and knowledge/perceptions and biomedical knowledge. The differences will be evaluated using t-tests, Mann-Whitney U test (or other non-parametric tests), chi-square methods, and Fisher's exact test as appropriate.

In-depth interviews: Audio recordings of in-depth interviews will be transcribed and translated to English. A preliminary codebook will be developed for both interview types (male and female). Selected investigators will piloted a codebook by independently reading and coding a randomly-selected subset of transcripts. Through an iterative consultative process, each investigator will revised their respective codebook and repeated this process until there was high interrater reliability among the group. All

For peer review only

transcripts will be coded in Atlas.ti v8.3 using constant comparison, and coding disagreements were resolved by consensus.

4.5. OBJECTIVE 3

Determine the cost-effectiveness and scalability of the intervention arms through costing and mathematical modeling.

- **Hypothesis 3.1:** The staged intervention will be more cost effective at having men initiate ART than both the lightest touch intervention and the intensive intervention.

4.5.1. Study design

We will conduct an incremental cost-effectiveness analysis and mathematical modelling to determine national scale-up potential. The average cost per successful outcome (early ART retention) will be calculated and compared across arms incrementally.

4.5.2. Data collection techniques and tools

Costs will be measured from the health care provider. We will use micro-costing methods by first creating an inventory of all the resources used to achieve the observed study outcomes including:

- Standard counseling interactions (staff cadre, training received, duration of interaction and distance from facility travelled where applicable)
- Motivational interviewing interactions (staff cadre, training received, duration of interaction and distance from facility travelled where applicable)
- Provider interactions (staff cadre, training received, duration of interaction and distance from facility travelled where applicable)
- Cost of reminder messages sent (when messages delivered telephonically instead of in person)

For each study patient, the quantity (number of units) of resources used will be determined. Unit costs of resources, which are not human subject data, will be obtained from external suppliers and the site's finance and procurement records and multiplied by the resource usage data to provide an average cost per study patient across centers in each study arm.

4.5.3. Data analysis

Cost-Effectiveness: Using the average cost per patient as described above, we will then estimate the cost per outcome achieved in each arm. The main measure of effectiveness for the cost-effectiveness analysis will be both the primary study outcome (early ART retention). We will calculate the difference in cost divided by the difference in effectiveness among study arms. Costs will be reported as means (standard deviations) and medians (IQRs) in USD, using the exchange rate prevailing during the follow up period.

National scale-up modeling: To determine the budget impact and affordability of the intervention arms, we will parameterize a national scale-up model using the study output. To determine the total cost and impact of the three intervention arms, as well as combinations of interventions, we will model cost and impact out to early ART retention (ART initiation and completion of the 4-week ART refill appointment). The following parameters to be estimated from this trial include:

- Percent of men not linking after HIVST (and thus eligible for this trial)

For peer review only

- Proportion of men that have not linked that could be reached
- Proportion of men that are known HIV-positive and on ART (not disclosed to their partner)
- Proportion of men that initiate ART
- Proportion of men that complete the 4-week ART refill appointment

We will then estimate the expected increase in the number of men linked to ART after index HIVST, adjusted by facility type where possible, by each intervention arm. The number of facility-level HIV tests conducted through index testing at all 652 public healthcare facilities in Malawi from Oct 2019-Sept 2020 will be used for these national calculations. Each intervention will be tested separately in this model, as well as different combination of interventions. Different scenarios will be explored where interventions are used at different facilities (urban versus rural targeting of interventions, geospatial targeting of interventions), or different groups of men within the same facility (where data suggest that different demographics of men resnd differently to the different interventions).

The national-level costs and expected number of men linked to ART, by each intervention and combinations of interventions, will be reported from this model. We will then contextualize the national cost of each intervention with a short-term 3-year budget impact: percent increase (or decrease) of the national HIV treatment budget with the inclusion of one of these interventions

5. ETHICAL CONSIDERATIONS

There is minimal risk associated with the above-mentioned procedures. We have extensive experience measuring ART initiation within HIVST studies. We conducted the first trials in the region to objectively measure ART initiation among men after receiving HIVST through the Index HIVST Trial (PI: Dovel) and PASTAL Trial (male partners of antenatal clients; PI: Choko). We draw from lessons learned from our previous trials.

Informed Consent

Informed consent will be obtained before any study-specific procedures are performed. The informed consent process will include information exchange, detailed discussion, and assessment of understanding of all required elements of informed consent, including the potential risks, benefits, and alternatives to study participation. The process will emphasize the randomized nature of the study and the differences that participants may experience as part of the study relative to current local standards of care. The study will include children 15 years of age and older. Following Malawian protocol, adolescents <18 years of age will be required to attain assent before completing the survey. Based on prior studies, we anticipate <10% of participants to be under 18 years of age, providing a small sample size to explore the potential impact of facility-based testing for youth.

Potential Benefits

Men who participate in the study may have access to additional HIV services not usually provided through routine care, such as appointment reminders, peer navigation, motivational interviewing, and home-based ART initiation. Men can refuse these additional services at any point. Further, both men and women will have the opportunity to discuss their use of HIV services and any concerns with HIV as individuals or as a couple. Information learned in this study may be of benefit to participants and others in the future, particularly information that may lead to optimized testing guidelines.

Potential risks and discomforts

Study procedures have minimal risk to the client. For men, maintaining privacy and confidentiality is a potential risk, particularly with home-based ART initiation. In our prior work delivering routine Index

For peer review only

HIVST, we have had health workers visit a cluster of homes (not just one) to avoid unwanted questions about the individual's serostatus. This has worked quite well, with no reports of unwanted disclosure, and we will use this approach in our proposed study to minimize risk of unwanted disclosure. Further, men may refuse any ART service at anypoint if they are uncomfortable.

For women, increased intimate partner violence (IPV) may be a potential risk, particularly if their male partner is prone to violence. To reduse these risks, women who report IPV with their current partner in the past 12 months will be excluded from the study. Female ART clients who report IPV at anypoint of the intervention will be withdrawn from the study, along with their male partner, counseled, and referred to community-based resources for IPV. We also will provide extensive counseling on status disclosure and an IPV hotline to all female participants. Further, Our PASTAL and Index HIVST Trials show no sign of increased IPV and we have published extensively on risk factors for IPV in other settings.[71-73]

Finally, Participation includes completion of a survey that will assess previous use of health services, perceptions of health services received, and sociodemographic and biomedical factors that may be associated with health service utilization. Participants may feel some psychological stress or discomfort from some of the questions, although most questions are not sensitive in nature. Participants may decline to answer any questions that make them uncomfortable and may end participation at any time.

Reimbursement/compensation

Participants will be provided MK 7,500 (equivalent to 10USD) for each survey completed (MK 15,000 / 20USD across the duration of the study). They will receive the above compensation regardless if they use HIV services or not. Those who complete the additional in-depth interview 6-months after study enrollment will receive an additional MK 7,500 (equivalent to 10USD) for their time.

Privacy and confidentiality

All study procedures will be conducted in private, and every effort will be made to protect participant privacy and confidentiality to the extent possible. Participant information will not be released without written permission to do so except as necessary for review, monitoring, and/or auditing. All study-related information will be stored securely. Participant research records will be stored in locked areas with access limited to study staff. All study data will be identified by participant ID (PID) only. Likewise, communications between study staff and protocol team members regarding individual participants will identify participants by PID only. Process evaluation documents, such as intervention monitoring and evaluation tools, will only include PID and will not store PID and identifiers together. All local databases will be encrypted and secured with password-protected access systems. Lists, logbooks, appointment books, and any other documents that link PID numbers to personal identifying information will be stored in a separate, locked location in an area with limited access. For the intervention, home visits will be conducted by health workers who visit a cluster of homes (not just one) at one time in order to avoid unwanted questions about the individual's serostatus. This has been used in other interventions focused on partner testing and treatment with high success of removing unwanted disclosure to community members.

6. DISSMINATION OF RESULTS

This study will set the stage for interventions that combine HIVST with differentiated models for early ART retention in low-resource settings. The study is timely and of high-impact. Findings will establish the effectiveness of home-based ART among male HIVST users, and can directly inform HIV programs throughout the region. The dissemination plan was developed to achieve the most impact while still ensuring dissemination among local stakeholders who may immediately benefit from study findings.

Partners in Hope is already integrated into national technical working groups, so dissemination will follow standard meeting schedules and draw upon Partners in Hope's longstanding history with the

Ministry of Health. Additionally, we will disseminate results through presentations at international scientific meetings and through high-impact peer-reviewed journals. The mentorship team has extensive experience publishing in high-impact journals (e.g., *AJPH, AIDS, BMJ, Lancet HIV, JAIDS*, *PLOS Med*)

7. PERSONNEL ROLES AND INSTITUTIONS

The proposed research team includes clinical researchers and implimentation science professionals with substantial experience in HIV testing, HIV prevention and treatment, cost effectiveness, differentiated care model studies, and male-focused studies and programs in Malawi and Sub-Saharan Africa. The study will be implemented in partnership with Partners in Hope Medical Center in Lilongwe, which has years of experience collaborating with Ministry of Health and local health facilities on similar studies, mentoring staff, and running studies embedded within routine clinical care.

- Kathryn Dovel, MPH, PhD, Principle Investigator, Division of Infectious Disease University of California Los Angeles (UCLA) and Research Director for Partners in Hope
- Thomas Coates, PhD, Co-Investigator, Division of Infectious Disease UCLA
- Risa Hoffman, MPH, MD, Co-Investigator, Division of Infectious Disease UCLA
- Brooke Nichols, Co-Investigator, School of Global Health, Boston Univeristy
- Lawrence Long, Co-Investigator, School of Global Health, Boston Univeristy
- Alemayehu Amberbir, Co-Investigator, Partners in Hope
- Augustine Choko, PhD, Site Co-Investigator, Malawi Liverpool Wellcome Trust
- Michal Kulich, Biostatistician, Charles Univeristy in Prauge
- Julie Hubabrd, MSc, Study Coordinator, Partners in Hope
- Kelvin Balakasi, Study Data Manager, Parners in Hope
- Khumbo Phiri, Implimentation Science Manager, Partners in Hope

Dr. Kathryn Dovel, the Principle Investigator, is the Science Director at Partners in Hope and an Assitant Assistant Professor in the Division of Infectious Diseases at UCLA. Dr. Dovel has over ten years of experience in Malawi and collaborating with the study team. She is regularly involved in UNAIDS and WHO workshops and meetings regarding strategies for male engagement, and has been a consultant on two Ministry of Health guidelines on the topic in Malawi.

Dr. Augustine Choko will be responsible with Dr. Dovel for overall adherence to the study protocol and serve as the primary liaison with the local IRB and key stakeholders in Malawi. Dr. Thomas Coates will serve as the community-based trials specialist, with over two decades of experience conducting individual- and cluster-randomized trials in communities with the end goal of engagement in HIV services. Dr. Risa Hoffman is an established clinical investigator and will serve as the MD specializing in differentiated models of ART treatment delivery and HIV care, and ensuring client safety. Brooke Nichols and Lawrence Long will be responsible for reviewing all modeling data, making an analysis plan for the proposed models, and providing modeling for publications. Dr. Michal Kulich is the Chair of the Probability and Statistics Department at Charles University and has extensive experience with the design, conduct, and analysis of clinical trials in the context of HIV prevention research.

Partners in Hope's staff Kelvin Balakasi (Data Manager) Julie Hubbard (Research Coordinator), Khumbo Phiri (Implimentation Science Manager) and Alemayehu Amberbir (Science Director) will be responsible implimentation and oversight inlcuidng data collection, data management, quality control, and training and certification of data entry personnel. They will also be responsible for ensuring the intervention promotes client safety, meets Ministry of Health guidelines, and is implemented in such a way to promote sustainability and scalability.

CV's for participating personelle are provided in the Appendix L.

For peer review only

8. REGULATORY OVERSIGHT

This study is sponsored by the Bill and Melinda Gates Foundation and implemented through Partners in Hope (PIH), Malawi. PIH staff will perform monitoring visits. As part of these visits, monitors will inspect study-related documentation to ensure compliance with all applicable regulatory requirements.
All health facilities will receive an Initial Registration Notification from PIH that indicates successful completion of the protocol registration process. A copy of the Initial Registration Notification will be retained in the site's regulatory files.

We have developed a trial advisory group. See Table 6 for details about the group members. The group will meet every quarter to review progress, and challenges with study implementation, and provide input on the final interventions to be tested, based on qualitative findings in Aim 1.

Table 6. *Description of trial advisory group*

Name	Affiliation	Expertise
Dr. Morna Cornell	University of Cape Town	Epidemiologist, health system barriers to men's care, men's HIV services, advocacy and policy change
Dr. Heidi van Rooyen	SA Human Sciences Research Council	Social scientist, HIV vulnerability and inequality, interventions for men's ART initiation
Dr. Deborah Donnell	University of Washington, Fred Hutch Vaccine and Infectious Disease Division	Biostatistician, international HIV trials, PI of the HPTN Statistical and Data Management Center
Dr. Connie Celum	University of Washington	Infectious disease physician and epidemiologist, implementation science in Africa, HIV prevention trials
Dr. Thoko Kalua	Malawi Ministry of Health, Deputy Director at Department of HIV and AIDS	Epidemiologist. Extensive experience in national HIV programs, M&E, and scale-up of interventions on the ground
Dr. Sergio Chicumbe	Mozambique National Health Institute (INS), Health System Research Cluster	Clinical trials and implementation science. Extensive experience in national public health programs, methodology for health services research and quality care improvement.

For any future protocol amendments, upon receiving final IRB/EC and any other applicable regulatory entity approvals, sites should implement the amendment immediately. Sites are required to submit an amendment registration packet to the PIH Protocol Team. PIH key personnel will review the submitted protocol registration packet to ensure that all the required documents have been received.

9. STUDY IMPLEMENTATION

For peer review only - http://bmjopen.bmj.com/site/about/guidelines.xhtml

Study implementation at each site will be guided site-specific standard operating procedures (SOPs). These SOPs will be updated and/or supplemented as needed to describe roles, responsibilities, and procedures for this study.

10. PROTOCOL DEVIATION REPORTING

All protocol deviations will be documented in participant research records. Reasons for the deviations and corrective and preventive actions taken in response to the deviations will also be documented. Deviations will be reported to site IRBs/ECs and other applicable review bodies in accordance with the policies and procedures of these review bodies. Serious deviations that are associated with increased risk to one or more study participants and/or significant impacts on the integrity of study data must also be reported to the Protocol Team as soon as possible.

For peer review only

11. WORK PLAN TIMELINE

Table 7: *Anticipated workplan timeline of study activities, by year*

IdeAL Study Timeline																
**Activities**	**Year 1**				**Year 2**				**Year 3**				**Year 4**			
Quarter	1	2	3	4	1	2	3	4	1	2	3	4	1	2	3	4
Preparation	X	X	X	X												
Objective 1:3. Test the effectiveness of models of varying intensity to link patients who use HIVST to care																
Enrollment					X	X	X	X								
Intervention and follow-up					X	X	X	X	X	X						
Follow-up analyses & writing										X	X	X	X			
Objective 2: 4. Identify factors associated with early ART retention																
Baseline sociodemographic survey					X	X	X	X								
Qualitative assessment- rolling					X	X	X	X	X	X						
Analyses & writing						X	X			X	X	X	X			
Objective 3: 5. Determine the cost and scalability of the intervention																
Data collection					X	X	X	X	X	X						
Synthesis of results and parameter estimation										X						
Model development										X	X	X				
Analyses & writing										X	X	X	X			

12. BUDGET AND JUSTIFICATION

Table 8: *Study budget*

Description	USD	Justification
Study Coordinator	5000	25% LOE to coordinate RAs
Research Assistant for data collection	15000	5 RAs for 6 months at 500 USD per month
Incentive for participants	21720	10USD per study visit, 2 study visits per participant, 1086 participants (543 men and 543 female partners)

Expert Client/Nurse lunch allowance	720	6USD lunch allowance for 10 Expert Clients and 10 nurses on a monthly basis to hear from them how the intervention is going
Telecomunications	1000	Mobile data collection processing by RAs and communication with coordinator
NHSRC application fee	150	Application fee
Sub Total	*43,590*	
NHSRC 10% fee	4,359	10% contribution fee of study budget
**Grand total**	**47,949**	

For peer review only

13. REFERENCES

1. Dovel K, Yeatman S, Watkins S, Poulin M. Men's heightened risk of AIDS-related death: the legacy of gendered HIV testing and treatment strategies. *AIDS (London, England)*. 2015;29(10):1123.

2. UNAIDS. *Ending AIDS: Progress towards 90-90-90 Targets*.; 2017.

3. Choko AT, Kumwenda MK, Johnson CC, et al. Acceptability of woman-delivered HIV self-testing to the male partner, and additional interventions: a qualitative study of antenatal care participants in Malawi. *Journal of the International AIDS Society*. 2017;20(1):21610.

4. Yeatman S, Chamberlin S, Dovel K. Women's (health) work: A population-based, cross-sectional study of gender differences in time spent seeking health care in Malawi. *PloS one*. 2018;13(12).

5. Mills EJ, Beyrer C, Birungi J, Dybul MR. Engaging men in prevention and care for HIV/AIDS in Africa. *PLoS medicine*. 2012;9(2).

6. Malawi National Statistics Office. *Demographic Health Survey: Malawi*.; 2016.

7. Sharma M, Ying R, Tarr G, Barnabas R. Systematic review and meta-analysis of community and facility-based HIV testing to address linkage to care gaps in sub-Saharan Africa. *Nature*. 2015;528(7580):S77–S85.

8. Partners in Hope. Update on index partner testing in Partners in Hope supported sites: quarterly review. Presented at the: Malawi Ministry of Health Technical Working Group: HIV Self-Testing; 2019.

9. Dovel K, Balakasi K, Shaba F, Phiri K, Offorjebe O, Gupta S. A Randomized trial on index HIV self-testing for partners for ART clients in Malawi. In: *Conference on Retroviruses and Opportunistic Infections, Seattle*. ; 2019.

10. Johnson CC, Kennedy C, Fonner V, et al. Examining the effects of HIV self-testing compared to standard HIV testing services: a systematic review and meta-analysis. *Journal of the International AIDS Society*. 2017;20(1):21594.

11. Masters SH, Agot K, Obonyo B, Mavedzenge SN, Maman S, Thirumurthy H. Promoting partner testing and couples testing through secondary distribution of HIV self-tests: a randomized clinical trial. *PLoS medicine*. 2016;13(11).

12. Ortblad K, Musoke DK, Ngabirano T, et al. Direct provision versus facility collection of HIV self-tests among female sex workers in Uganda: a cluster-randomized controlled health systems trial. *PLoS medicine*. 2017;14(11).

13. MacPherson P, Lalloo DG, Webb EL, et al. Effect of optional home initiation of HIV care following HIV self-testing on antiretroviral therapy initiation among adults in Malawi: a randomized clinical trial. *Jama*. 2014;312(4):372–379.

14. Sibanda EL, Weller IV, Hakim JG, Cowan FM. The magnitude of loss to follow-up of HIV-exposed infants along the prevention of mother-to-child HIV transmission continuum of care: a systematic review and meta-analysis. *AIDS (London, England)*. 2013;27(17):2787.

15. Larsson EC, Thorson A, Nsabagasani X, Namusoko S, Popenoe R, Ekström AM. Mistrust in marriage-Reasons why men do not accept couple HIV testing during antenatal care-a qualitative study in eastern Uganda. *BMC public health*. 2010;10(1):769.

16. Koo K, Makin JD, Forsyth BW. Barriers to male-partner participation in programs to prevent mother-to-child HIV transmission in South Africa. *AIDS Education and Prevention*. 2013;25(1):14–24.

17. Theuring S, Nchimbi P, Jordan-Harder B, Harms G. Partner involvement in perinatal care and PMTCT services in Mbeya Region, Tanzania: the providers' perspective. *AIDS care*. 2010;22(12):1562–1568.

18. Sileo KM, Fielding-Miller R, Dworkin SL, Fleming PJ. What role do masculine norms play in men's HIV testing in sub-Saharan Africa?: a scoping review. *AIDS and Behavior*. 2018;22(8):2468–2479.

19. van den Berg W, Brittain K, Mercer G, et al. Improving men's participation in preventing mother-to-child transmission of HIV as a maternal, neonatal, and child health priority in South Africa. *PLoS medicine*. 2015;12(4).

20. Unaids HIV. *Addressing a Blind Spot in the Response to HIV—Reaching out to Men and Boys*. Joint United Nations Programme on HIV/AIDS Geneva; 2017.

21. Cornell M, Cox V, Wilkinson L. Public health blindness towards men in HIV programmes in Africa. *Tropical Medicine & International Health*. 2015;20(12):1634–1635.

22. Poulin M, Dovel K, Watkins SC. Men with money and the "vulnerable women" client category in an AIDS epidemic. *World Development*. 2016;85:16–30.

23. Pai NP, Sharma J, Shivkumar S, et al. Supervised and unsupervised self-testing for HIV in high-and low-risk populations: a systematic review. *PLOS medicine*. 2013;10(4).

24. Sharma M, Barnabas RV, Celum C. Community-based strategies to strengthen men's engagement in the HIV care cascade in sub-Saharan Africa. *PLoS medicine*. 2017;14(4).

25. Choko AT, MacPherson P, Webb EL, et al. Uptake, accuracy, safety, and linkage into care over two years of promoting annual self-testing for HIV in Blantyre, Malawi: a community-based prospective study. *PLoS medicine*. 2015;12(9).

26. Decroo T, Rasschaert F, Telfer B, Remartinez D, Laga M, Ford N. Community-based antiretroviral therapy programs can overcome barriers to retention of patients and decongest health services in sub-Saharan Africa: a systematic review. *International health*. 2013;5(3):169–179.

27. Wools-Kaloustian KK, Sidle JE, Selke HM, et al. A model for extending antiretroviral care beyond the rural health centre. *Journal of the International AIDS Society*. 2009;12(1):22–22.

28. Apondi R, Bunnell R, Awor A, et al. Home-based antiretroviral care is associated with positive social outcomes in a prospective cohort in Uganda. *JAIDS Journal of Acquired Immune Deficiency Syndromes*. 2007;44(1):71–76.

29. Jaffar S, Amuron B, Foster S, et al. Rates of virological failure in patients treated in a home-based versus a facility-based HIV-care model in Jinja, southeast Uganda: a cluster-randomised equivalence trial. *The Lancet*. 2009;374(9707):2080–2089.

For peer review only

30. Malterud K, Siersma VD, Guassora AD. Sample size in qualitative interview studies: guided by information power. *Qualitative health research*. 2016;26(13):1753–1760.

For peer review only

14. APPENDIX

14.1. APPENDIX A – Study Invitation Cards

STUDY INVITATION CARD
Identifying efficient linkage strategies for HIV self-testing (IDEaL)
English

Study invitation Card

Date: ……/……/2020

Dear: ____

At ______________________________________, we are conducting a study. The study will generate information that will help the Malawi government, Partners in Hope and other stakeholders develop programs that will help your community lead a healthy life. We are therefore inviting you and your partner to come to ______________________________ on______________________________ at__________, so that we can assess if you are eligible for the study. If eligible, you and your partner will each receive MK 7,500 (equivalent to 10USD).

Thank you for your kind attention to this request and we look forward to seeing you. When coming call or flash this number…………………… so that you are met by study personnel.

Sincerely,

[District Health Officer]

For peer review only

STUDY INVITATION CARD
Identifying efficient linkage strategies for HIV self-testing (IDEaL)
Chichewa

Khadi Lokuyitanani Ku Kafukufuku

Tsiku: ……/……/2020

Wokondedwa: ____

Ku chipatala cha, __________________________________, tikupanga kafukufuku. Kafukufukuyu adzathandiza kupeza mfundo zomwe zidzathandize boma la Malawi, bungwe la Partners In Hope ndi mabungwe/magulu ena kukhazikitsa ndondomeko zomwe zidzathandizire anthu a mdela lanu kukhala ndi umoyo wanthanzi. Choncho mukuyitanidwa pamodzi ndi okondedwa anu kuti mubwere ku_______________ tsiku nthawi ya ____________, ndicholinga choti tidzaone ngati muli oyenera kutenga nawo mbali mu kafukufukuyu. Mukadzapezeka kuti ndinu oyenera kutenga nawo mbali mu kafukufukuyu, inuyo komanso bwenzi lanu mudzalandira K7,500 (yokwana 10USD) aliyense.

Zikomo kwambiri chifukwa cha chidwi chanu pa pempholi ndipo ife tikuyembekezera kuzaonana nanu pa tsikuli. Mukamadzabwera, muzaimbe kapena kufulasha pa nambala iyi…………………………………kuti muzakumane ndi anthu a kafukufukuyu.

Ndine wanu,

[District Health Officer]

For peer review only

14.2. APPENDIX B – Recruitment and Screening Script

RECRUITMENT AND SCREENING SCRIPT
Identifying efficient linkage strategies for HIV self-testing (IDEaL)
Female

Thank you for speaking with me about the study entitled, *"Identifying efficient linkages strategies for HIVST"* conducted by Partners in Hope and the University of California Los Angeles in the United States. You are being approached because you recently reported that your male partner tested HIV-positive using a HIV self-test kit.

The purpose of this is to determine what are the best interventions that can help men who are diagnosed with HIV use other health services, if desired. The study will offer several different strategies for HIV services to see what works best for men who use HIV self-testing kits.

Would you be interested in participating in the eligibility screening to see if you are eligible to participate in the study? Your participation is voluntary and you will not be penalized if you choose not to participate in the screening or the project.

[*If no, thank the person and end the session*]

[*If yes, continue to the screening questions below or make an appointment to complete the screening questions*]

Before we continue with the study, we first need to determine if you are eligible to participate.

1. Are you and your partner 15 years old or older?
2. To the best of your knowledge, did your partner recently test HIV-positive using a HIV self-test kit?
3. To the best of your knowledge, your partner currently NOT taking ART?
4. Does your partner live in the facility catchment area?
5. In the last 30 days, your current partner has NEVER hit, slapped, or kicked you, or forced you to have sexual intercourse with them?

If you answered yes to all these questions, then you are eligible to participate in the study. You may stay here to continue with the study consent, and we will explain how the study will be conducted.

For peer review only

RECRUITMENT AND SCREENING SCRIPT
Identifying efficient linkage strategies for HIV self-testing (IDEaL)
Female (Chichewa)

Zikomo kwambiri povemera kucheza nane zokhudza study yotchedwa "Kupeza njira zabwino zothandizira anthu omwe akulandira ma ARV" amene akupangidwa ndi bungwe la Partners In Hope-EQUIP polojekiti ndi sukulu ya ukachenjede ya University of California Los Angeles yaku Amerika. Mukufunsidwa kuti mutenge nawo mbali mu gawo lakafukufukuyu chifukwa bwenzi lanu lalimuna lili ndi kachirombo ka HIV ndipo linadziwa zotsatirazi pogwiritsa ntchito ka chida koziyezera wekha.

Cholinga cha kafukufukuyu ndi kufuna kupeza njira zabwino zomwe zingathandize azibambo omwe ali ndi kachirombo ka HIV kugwiritsa ntchito thandizo lina la zaumoyo, ngati akonda kutero. Studyyi idzapeleka njira zingapo zosiyanasiyana za thandizo la HIV kuti awone njira yomwe ikugwira bwino kwa azibambo omwe amagwiritsa ncthito ka chida koziyezera wekha HIV.

Kodi muli okondwa kutenga nawo mbali mu mayele ofuna kuwona ngati muli oyenela kutenga nawo mbali mu study? Kutenga nawo mbali kwanu ndi kosakakamiza ndipo palibe chilango chilichonse ngati mungasankhe kusatenga nawo mbali mu mayele a study.

[*Ngati ayi, thokozani munthuyo ndipo malizani session*]

[Ngati eya, pitilizani kufunsa mafunso a mayele omwe ali munsiwa kapena sankhani tsiku loti muzamalize kufunsa mafunso]

Tisanapitilize ndi study, choyamba tifuna tidziwe ngati muli oyenera kutenga nawo mbali.

1. *Muli ndi zaka khumi ndi zisanu kapena kuposela apo?*
2. *Monga mukudziwira kodi bwenzi lanu laziyezera kachida koziyezera wekha ndikupezeka ndi kachirombo ka HIV?*
3. *Monga mene mukudziwira, kodi pakadali pano bwenzi lanu likumwa mankhwala a ma ARV?*
4. *Kodi bwenzi lanu limakhala mu dela lozungulira chipatala?*
5. *M'masiku makumi atatu apitawa, bwenzi lanu SILINAPANGE izi kumenyani kapena kukukakamizani kuti mugonane nalo?*

Ngati mwayankha eya pa mafunso onse mwafunsidwawa, zikusonyeza kuti ndinu oyenera kutenga nawo mbali mu study. Muli omasuka kukhala ndikupitiliza chilolezo cha study, ndipo ndikufotokozerani za momwe study ichitikire.

For peer review only - http://bmjopen.bmj.com/site/about/guidelines.xhtml

RECRUITMENT AND SCREENING SCRIPT
Identifying efficient linkage strategies for HIV self-testing (IDEaL)
Male

Thank you for speaking with me about the study entitled, *"Identifying efficient linkages strategies for HIVST"* conducted by Partners in Hope and the University of California Los Angeles in the United States. You are being approached because you recently reported testing HIV-positive using a HIV self-test kit.

The purpose of this is to determine what are the best interventions that can help men who are diagnosed with HIV use other health services, if desired. The study will offer several different strategies for HIV services to see what works best for men who use HIV self-testing kits.

Would you be interested in participating in the eligibility screening to see if you are eligible to participate in the study? Your participation is voluntary and you will not be penalized if you choose not to participate in the screening or the project.

[*If no, thank the person and end the session*]

[*If yes, continue to the screening questions below or make an appointment to complete the screening questions*]

Before we continue with the study, we first need to determine if you are eligible to participate.

1. Are you 15 years old or older?
2. You recently test HIV-positive using a HIV self-test kit?
3. You are NOT currently taking ART?
4. Do you live in the facility catchment area?

If you answered yes to all these questions, then you are eligible to participate in the study. You may stay here to continue with the study consent, and we will explain how the study will be conducted.

For peer review only

RECRUITMENT AND SCREENING SCRIPT
Identifying efficient linkage strategies for HIV self-testing (IDEaL)
Male (Chichewa)

Zikomo kwambiri povemera kucheza nane zokhudza study yotchedwa "Kupeza njira zabwino zothandizira anthu omwe akulandira ma ARV" amene akupangidwa ndi bungwe la Partners In Hope-EQUIP polojekiti ndi sukulu ya ukachenjede ya University of California Los Angeles yaku Amerika. Mukufunsidwa kuti mutenge nawo mbali mu gawo lakafukufukuyu chifukwa muli ndi kachirombo ka HIV ndipo munadziwa zotsatirazi pogwiritsa ntchito ka chida koziyezera wekha.

Cholinga cha kafukufukuyu ndi kufuna kuoeza njira zabwiino zomwe zingathandize azibambo omwe ali ndi kachirombo ka HIV kugwiritsa ntchito thandizo lina la zaumoyo, ngati akonda kutero. Studyyi idzapeleka njira zingapo zosiyanasiyana za thandizo la HIV kuti awone njira yomwe ikugwira bwino kwa azibambo omwe amagwiritsa ncthito ka chida koziyezera wekha HIV.

Kodi muli okondwa kutenga nawo mbali mu mayele ofuna kuwona ngati muli oyenela kutenga nawo mbali mu study? Kutenga nawo mbali kwanu ndi kosakakamiza ndipo palibe chilango chilichonse ngati mungasankhe kusatenga nawo mbali mu mayele a study.

[Ngati ayi, thokozani munthuyo ndipo malizani session]

[Ngati eya, pitilizani kufunsa mafunso a mayele omwe ali munsiwa kapena sankhani tsiku loti muzamalize kufunsa mafunso]

Tisanapitilize ndi study, choyamba tifuna tidziwe ngati muli oyenera kutenga nawo mbali.

1. *Muli ndi zaka khumi ndi zisanu kapena kuposela apo?*
2. *Kodi mwaziyezera kachida koziyezera wekha ndikupezeka ndi kachirombo ka HIV?*
3. *Kodi pakadali pano mukumwa mankhwala a ma ARV?*
4. *Kodi mumakhala mu dela lozungulira chipatala?*

Ngati mwayankha eya pa mafunso onse mwafunsidwawa, zikusonyeza kuti ndinu oyenera kutenga nawo mbali mu study. Muli omasuka kukhala ndikupitiliza chilolezo cha study, ndipo ndikufotokozerani za momwe study ichitikire.

For peer review only

14.3. APPENDIX C – Written Informed Consent- Female

WRITTEN INFORMED CONSENT
Identifying efficient linkage strategies for HIV self-testing (IDEaL)
Female

You are asked to participate in a research study entitled *"Identifying efficient linkages strategies for HIVST"* conducted by Partners in Hope and the University of California Los Angeles in the United States. You are being requested to take part in the study because you recently reported that your male partner tested HIV-positive using a HIV self-test kit. Your participation in this study is entirely voluntary. You will be read the information below, and you are free to ask questions about anything you do not understand, before deciding whether or not to participate. ***I as the field assistant for this study will take you through this consenting process.***

- **Why is this study being done?**

HIV self-testing is very helpful for people who want to know their status but do not usually go to the health facility. However, it can be hard for individuals who test HIV-positive with HIV self-testing to be able to access other health services. Researchers want to determine what are the best interventions that can help men who are diagnosed with HIV use other health services, if desired. The study will offer several different strategies for HIV services to see what works best for men who use HIV self-testing kits.

- **What will happen if you take part in this research study?**

There are several steps to this study. if you volunteer to participate in this study you will have the opportunity to participate in the following components:

1. Allow me to trace your male partner, or take me to your male partner in order to invite him to participate in the study as well. Note, you can choose to participate in the study even if your partner refuses or you think your partner would refuse.

2. Complete one or two study visits where a research assistant like myself will interview you and ask you information about yourself, including whether you are married, number of sexual partners, your level of education, information about your experiences with HIV services, and how you feel about HIV testing and treatment services. We will ask you questions today (or a day nearby that is convenient for you) and, if your partner enrolls in the study, we will ask you similar questions again in four months in order to see if anything has changed. Each interview will last about 45minutes. You can refuse a follow-up survey at any point

For peer review only

3. If your partner agrees to participate in the study, he will be randomized to one of three interventions. We will do the randomization together with him so he can see exactly what intervention he will be offered. The potential interventions are:

    1) Standard of care where providers may send him reminders about the benefits of health services.
    2) Motivational Interviewing where he can talk to someone about his life, challenges he faces, and strategies to make his life better and additional services as needed.
    3) Home-based health services whereby a provider will offer him HIV services and NCD screening at your home as a one-time event. He will then be visited after 4-weeks to be escorted to the clinic if desired.

    Regardless of what arm your partner is randomized to, he can always refuse health services or refuse talking to a health care provider and still remain in the study. You can remain in the study regardless of what your partner does.

4. Finally, you may be randomly selected to within 6-months of the study to complete a 1-hour in-depth interview so we can learn more about your experiences in the study. Not all participants will be contacted for the interview and you always have the right to decline an in-depth interview – refusal will not affect your participation in the larger study.

- **How long will you be in the research study?**

All study activities will be completed within 6-months of today.

- **Are there any potential risks or discomforts that you can expect from this study?**

You will be asked a series of questions by a research assistant about your sexual relationship and your perceptions of your partners use of HIV and other health services. We will NEVER disclose your HIV status to your partner. We will NEVER disclose to your partner that you told us he had tested HIV+. However, you may feel uncomfortable answering some questions asked during the interview or you may feel comfortable having your partner in the study. You are able to withdraw from the study at any time. During an interview you can say "I don't want to answer" to any questions that make you uncomfortable. All questions will be asked in a private place so that no one else will hear your answers.

If you experience distress or adverse events as a result of the study, we will provide you with counseling resources or refer you to resources for assistance.

- **Are there any potential benefits to participating?**

You will have the opportunity to discuss information about your well-being, your relationship with your partner, and HIV services for men with a Research Assistant in a confidential, private manner.

- **Are there any potential benefits to society?**

Information obtained as part of this work may be of benefit to the larger Malawi program, or similar programs in sub-Saharan Africa, since the work aims to determine if there are better ways to offer HIV services to men who use HIV self-test kits. If researchers better understand what

For peer review only

type of programs work better for men, the program in Malawi can be scaled up and strengthened to provide these specific types of increased support.

- **Will you receive payment for being part of this study?**

Your participation is entirely voluntary. You will be provided MK 7,500 (equivalent to 10USD) for each survey completed (MK 15,000 / 20USD across the duration of the study). You will receive the above compensation regardless if you use HIV services or not. Those who complete the additional in-depth interview 6-months after study enrollment will receive an additional MK 7,500 (equivalent to 10USD) for their time.

- **What is the cost of participating in this study?**

There is no cost to participate in this study.

- **Will information about me be kept confidential?**

The study team are the only people who will know about you or any information that you provide in this study. If necessary to protect your rights or welfare (for example, if you are injured and need emergency care) or if required by Malawian law, specific information about you may be made available to providers or officials.

Authorized representatives of the Malawi National Health Sciences Research Council who are responsible for ensuring the rules related to research are followed, may need to review records of study participants. As a result, they may see your name; but they will not to reveal your identity to others.

When the results of the research are published or discussed in meetings, no information will be included that would reveal your identity. Any paperwork related to the study which contains information about you will be kept in a locked cabinet in a locked office. Only staff members of the study will have access to this information. A code will be assigned to each individual participating in the study. This code will be stored on a computer in a locked file. The key to unlock the information will only be known by the research staff. All data entered into a computer will be entered using this code so information will no longer have any information that can identify you such as your name. Forms containing any identifying information will be destroyed two years after the study is finished.

- **Participation and Withdrawal**

Your participation in this research is VOLUNTARY. If you choose not to participate, that will not affect your relationship with the hospital, your health provider or health centre you usually get your medical care from, or your right to health care. If you decide to participate, you are free to withdraw your consent and stop your participation at any time and can still receive future health care at the hospital or health center you go to.

- **Withdrawal of Participation by the Investigator**

The research investigator may stop your participation in this research if he or she feels this is best for you. The investigators will make the decision and let you know if it is not possible for you to continue. The decision may be made to protect your health and safety.

For peer review only

- **Who can answer questions I might have about this study?**

In the event of a research related injury or if you experience a problem, please immediately return to the hospital or health centre you go to or contact Khumbo Phiri. The NHSRC Ministry of Health information (Dr. Mitambo) is also provided in case you have questions about your rights as a research participant.

Kusiyitsidwa kutenga nawo mbali mu kafukufuku ndi wakafukufuku

Anthu opangitsa kafukufukuyu akhonza kukuletsani kutenga nawo mbali mukafukufukuyu akaona kuti ndi bwino kuti mutero. Anthu akafukufukuwa azapanga chiganizochi ndikukudziwitsani kuti sizitheka kuti mupitirize. Chiganizochi chitha kupangidwa kuti ateteze thanzi ndi chitetezo chanu.

Investigator

Khumbo Phiri
Mobile: +265999840946
Partners in Hope Clinic
Area 36, Plot8
M1 Road South
Lilongwe, Malawi

OR

Dr.C. Mitambo
The Secretariate, NHSRC
Ministry of Health
P.O Box 30377
Lilongwe 3
Cell +265888344 443

SIGNATURE OF RESEARCH SUBJECT [OR LEGAL REPRESENTATIVE]

I have read (or someone has read to me) the information provided above. I have been given an opportunity to ask questions and all of my questions have been answered to my satisfaction. I have been given a copy of this form.

Ndawerenga (kapena munthu wina wandiwerengera) zonse zalembedwa mwambamu. Ndapatsidwa mwayi wofunsa mafunso ndi mafunso onse ndinafunsa ayankhidwa ndipo ndakhutusidwa. Ndapasidwa pepala ina yangati yomweyi.

BY SIGNING THIS FORM, I WILLINGLY AGREE TO PARTICIPATE IN THE RESEARCH:

____

Name of Subject

____

Name of Legal Representative (if applicable)

Fingerprint

For peer review only

____________________________________ DATE (DAY/MO/YR): _________________

Signature of Subject or Legal Representative
(may place an X OR fingerprint if unable to sign)

SIGNATURE OF INVESTIGATOR OR DESIGNEE

I have explained the research to the subject or his/her legal representative and answered all of his/her questions. I believe that he/she understands the information described in this document and freely consents to participate.

____

Name of Investigator or Designee

______________________________ __________________________

Signature of Investigator or Designee Date (must be the same as subject's)

For peer review only

WRITTEN INFORMED CONSENT
Identifying efficient linkage strategies for HIV self-testing (IDEaL)
Female (Chichewa)

Mukufunsidwa kuti mutenge nawo mbali mu gawo lino la kafukufuku wotchedwa"kupeza njira zoyenera zobwezeletsa anthu pa thandizo la ma ARV" amene akupangidwa ndi bungwe la Partners In Hope-EQUIP polojekiti ndi sukulu ya ukachenjede ya University of California Los Angeles yaku Amerika. Mukufunsidwa kuti mutenge nawo mbali mu gawo lakafukufukuyu chifukwa bwenzi lanu lalimuna linapezeka ndi kachirombo ka HIV pogwiritsa ntchito kachida koziyezela wekha HIV. Kutenga nawo mbali mukafukufuku ameneyi sikokakamiza. Tikuwerengerani uthenga omwe walembedwa pansipa, ndipo muli ololedwa kufunsa mafunso aliwonse pa zomwe simukumvetsetsa,musanapange chiganizo chotenga nawo mbali kapena ayi. ***Ine ngati othandizira mukafufuku ameneyu ndikuthandizani pa ndondomeko yotenga chilolezo***.

- **Chifukwa chiyani kafukufukuyu akuchitika?**

Kuziyeza wekha HIV ndi kofunika kwa anthu omwe akufuna kudziwa za momwe mthupi mwawo mulili ku mbali ya HIV koma sapita kuchipatala, kotelo, ndikovuta kwa anthu omwe apezeka ndi HIV kudzela chipangizo choziyezela wekha kuti apeze thandizo la zaumoyo. Akafukufuku akufuna apeze njira zabwino zomwe zingathe kuthandiza azibambo omwe apezeka ndi HIV kuti agwiritse ntchito thandizo lina lazaumoyo ngati angakonde kutero. Kafukufukuyu apeleka mwayi wa njira zosiyana siyana zothandizira HIV kuti aone zomwe zingagwire ntchito bwino kwa azibambo omwe amagwiritsa ntchito ka chida koziyezela wekha HIV.

- **Chichitike ndi chani mukatenga nawo mbali mu kafukufukuyu?**

Pali ma gawo angapo omwe adzachitike mukafukuku, Ngati mungazipereke kutenga nawo mbali mu kafukufukuyu mudzatenga nawo mbali mu magawo otsatirawa:

1. Mundilole kuti ndifufuze bwenzi lanu lalimuna kapena ndipelekezeni kwa bwenzi lanu lalimuna ndi cholinga choti nalonso litenge nawo gawo mu kafukufuku. Chidziwitso: muli omasuka kutenga nawo mbali mu kafukufu ngati bwenzi lanu lakana kutenga nawo mbali kapena mukuganiza kuti bwenzi lanu likana.

2. Pamapepo pa kucheza koyamba komanso kachiwiri othandiza kafukufuku ngati ine ndidzacheza nanu ndikukufunsani mafunso okhuza inuyo, kuphatikizapo ngati muli pa banja, muli ndi abwenzi ogonana nawo angati, maphunziro anu komanso zomwe munakumana nazo polandira thandizo la HIV komanso momwe mumnvera kumbali ya thandizo la HIV angakhalenso kuyezedwa HIV. Tikufunsani mafunso lelo (Ngati lelo muli okonzeka kuyankha mafuns)komanso miyezi inayi ikudzayi kuti tiwone ngati pali chomwe chasintha, kucheza kuli konse kuzitenga nthawi yosachepela makumi anayi ndi isanu. Muli ololedwa kukana kutenga nawo mbali mu kucheza kotsatira nthawi iliyonse.

3. Ngati bwenzi lanu lidzatenge nawo mbali mu study, adzaikidwa mu gulu limodzi mwa magulu atatu mwa mayere. Tidzachita mayere limodzi ndi bwenzi lanu kuti awone kuti ali 'gulu liti mwa magulu atatuwa. Maguluwa ali motere:

1) Chikumbutso cha ubwino wa thandizo la zaumoyo
2) Kucheza kwa chilimbitso komwe mungathe ndi mwayi ocheza ndi anthu ena ndi kuwafotokozela za umoyo wanu, zofuta zomwe mumakumana nazo komanso njira zomwe mumagwiritsa ntchito kuti moyo wanu ukhale wosavuta.
3) Thandizo la zaumoyo lomwe mumatha kulandira pakhomo monga thandizo la HIV komanso NCD lomwe mumalandila kamodzi.Pakatha masaba anayi mudzayendeledwa ndi wa zaumoyo yemwe adzakupelekezeni ku chipatala komwe mukapitilize kulandira thandizo ngati mwakonda kutelo.

Posatengera njira yomwe mwapatsidwa mongathe kukana kulandira thandizo la zaumoyo koma ndikupitiliza kutenga nawo mbali mu kafukufuku ndipo tingathe kupitilizabe kucheza komwe tatchula m'mwambamu.

4. Pamapeto tidzakuyendelani pakutha kwa miyezi isanu ndi umodzi ya kafukufuku kuti tidzacheze nanu komanso kuti tidzanve za momwe mukunvera za kafukufuku, maganizo anu okhudza njira zina za mtsogolo komanso, thandizo lina lowonjezera ngati ilipo. Si onse otenga nawo mbali omwe adzaonedwe ndipo muli ololedwa kukana kutenga nawo mbali mukucheza ndipo kukana kwanu sikudzaononga mwayi wanu otenga nawo mbali mu study.

- **Mutenga nthawi yaitali bwanji muli mukafukufuku?**

Zochitika zonse zakafukufuku zizamalizidwa mu miyezi isanu ndi imodzi.

- **Pali zinthu zosowetsa mtendere kapena zodetsa nkhawa zomwe mungayembekezere kuchokera mu kafukufukuyu?**

Mufunsidwa mafunso angapo ndi opangitsa kafukufuku okhudza maubwenzi anu ogonana komanso maganizo anu pa momwe abwenzi anu amagwiritsira thandizo la HIV ndi mathandizo ena a zaumoyo. SITIDZAULULA momwe mthupi mwanu muliri kumbali ya kachilombo ka HIV kwa bwenzi lanu. SITIDZAULURA kwa bwenzi lanu kuti munatiuza kuti ali ndi kachilombo ka HIV. Ngakhale zili choncho, mukhoza kusamasuka kuyankha mafunso ena mu kafukufuku kapenanso mukhoza kufuna kuti bwenzi lanu likhale nanu poyankha mafunsowa. Muli ndi ufulu osiya kutenga nawo gawo mu kafukufuku nthawi iliyonse. Mkati mwa kucheza kwathu, mukhoza kunena kuti "Sindikufuna kuyankha" ku funso lililonse lomwe sindinu omasuka kuyankha. Mafunso onse afunsidwa malo oduka mphepo kuti munthu wina aliyense asamve mayankho anu.

Ngati mungapeze mavuto kapena nkhawa mu mtima kamba kotenga nawo gawo mu kafukufukuyu, tikupatsani uphungu oyenera kapena kukulozerani koyenera kupeza thandizo.

- **Pali cholowa chilichonse potenga nawo mbali mukafukufukuyu?**

Mudzakhala ndi mwayi okambilana ndikunva zambiri zokhudza moyo wanu komanso thandizo la HIV ndi othandiza kafukufuku komanso dotolo munjira yachinsisi. Mudzakhalanso ndi mwayi oyamba mankhwala a ma ARV ku chipatala cha kufuna kwanu.

- **Pali cholowa chilichonse kwa anthu a mudera?**

Uthenga womwe udzatengedwe ngati mbali imodzi yakafukufukuyu uzakhala othandiza mu mapologalamu a dziko la Malawi, kapena ma pologalamu ena ofananirapo a kum'mwera kwa Africa, chifukwa choti ntchito imeneyi ikufuna kuona ngati pali njira yabwino yopeleka thandizo la HIV mwa

For peer review only

azibambo, ma pologalamu aku Malawi azapita patsogolo ndikulimbikitsa njira zopititsira patsogolo.

- **Kodi mulandira malipiro potenga nawo mbali mukafukufukuyu?**

Kutenga nawo mbali kosakakamiza. Mudzalandira chiongola dzanja cha ndalama zokwana 7,500(Pafupifupi MK 15,000/20USD). Mudzalandila ndalamazi olo mutakhala kuti simunatenge nawo kapena mwatenga nawo mbali mu thandizo la HIV. Kwa omwe adzamalize nawo kucheza kowonjezera patatha miyezi isanu ndi umodzi adzalandira ndalama yowonjezera yokwana MK 7,500 (yokwana pafupi fupi 10 USD0 chifukwa cha nthawi yawo.

- **Kodi pali kulipira kulikonse chifukwa chotenga nawo mbali mukafukufukuyu?**

Kutenga nawo mbali mukafukufukuyu ndi kwaulere.

- **Kodi uthenga wanga uzasungidwa mwa chinsinsi?**

Anthu ogwira nawo ntchito mu kafukufuku okhawo ndi amene adziwe za uthenga wanu kapena chilichonse chomwe mutiuze pa kafukufukuyu.

Anthu ovomerezeka oyimirira bungwe la National Health Sciences Research Council ndi UCLA office for Protection Of Research Subjects ndi amene ali ndi udindo woonetsetsa kuti malamulo a kafukufuku akutsatidwa, akhoza kufuna kuona nawo kaundula wa anthu amene akutenga nawo mbali mukafukufukuyu. Kutanthauza kuti akhonza kuzaona dzina lanu; koma sangaulure zokhuza inu kwa anthu ena.

Pa nthawi imene zotsatira za kafukufukuyu zidzatsindikidwa kapena kukambidwa mu misonkhano, palibe uthenga womwe udzayikidwe wokuzindikiritsani. Uthenga uliwonse wolembedwa pa pepala wokhunza inu uzasungidwa mu kabati yokhoma mu ofesi yokhomanso. Anthu ogwira nawo ntchito mukafukufukuyu okhawo ndi amene azathe kuona uthenga umenewu. Njira yotanthauzira uthenga umenewu izadziwika ndi anthu akafukufukuyu basi. Uthenga onse olowetsedwa pa makina a kompyuta uzalowetsedwa kugwiritsa ntchito nambala, ndekuti uthenga onse sudzakhalanso ndi zokuzindikiritsani monga dzina lanu. Ma pepala amene pali uthenga okuzindikitsani adzawonongedwa pakapita zaka ziwiri chimalizireni kafukufukuyu.

- **Kutenga mbali ndi kusiya kutenga mbali**

Kutenga nawo mbali mu kafukufukuyu SIKOKAKAMIZA. Ngati musankhe kuti simutenga nawo mbali, izi sizingasokoneze ubale wanu ndi chipatala chino, anthu ogwira ntchito kuchipatala,kapena chipatala chomwe mumalandilirako chithandizo, kapena ufulu wanu wolandira thandizo la chipatala. Ngati mwapanga chisankho chotenga nawo mbali, mukhoza kuchosa chilolezo chanu ndikusiya kutenga nawo mbali nthawi ina iliyonse ndipo mungathe kuzalandirabe chithandizo pa chipatala pano mtsogolo.

- **Kusiyitsidwa kutenga nawo mbali mu kafukufuku ndi wakafukufuku**

Anthu opangitsa kafukufukuyu akhonza kukuletsani kutenga nawo mbali mukafukufukuyu akaona kuti ndi bwino kuti mutero. Anthu akafukufukuwa azapanga chiganizochi ndikukudziwitsani kuti sizitheka kuti mupitirize. Chiganizochi chitha kupangidwa kuti ateteze thanzi ndi chitetezo chanu.

- **Angayankhe ndi ndani mafunso amene ndingakhale nawo wokhuza kafukufukuyu?**

Zitachitika kuti mwavulala kamba ka kafukufukuyu kapena mukukumana ndi vuto,chonde bwelerani mwachangu kuchipatala chomwe munapitako kapena lumikizanani ndi Mike Nyirenda. Bungwe la NHSRC Ministry of Health information kudzera mwa Dr Kathyola liliponso ngati mungakhale ndi mafunso okhudza ufulu wanu ngati munthu wotenga nawo mbali mu kafukufuku.

Investigator
Khumbo Phiri
Mobile: +265999840946
Partners in Hope Clinic
Area 36, Plot8
M1 Road South
Lilongwe, Malawi

OR

Dr.C. Mitambo
The Secretariate, NHSRC
Ministry of Health
P.O Box 30377
Lilongwe 3
Cell +265888344 443

SAYINI YA OTENGA NAWO MBALI [KAPENA YA OYIMIRA OTENGA NAWO MBALI]

Ndawerenga (kapena munthu wina wandiwerengera) zonse zalembedwa mwambamu. Ndapatsidwa mwayi wofunsa mafunso ndi mafunso onse ndinafunsa ayankhidwa ndipo ndakhutusidwa. Ndapasidwa pepala ina yangati yomweyi.

POSAYINA PA PEPALALI, NDIKUVOMERA MOSAKAKAMIZIDWA KUTENGA NAWO MBARI MU KAFUKUFUKU:

____

Name of Subject

____

Name of Legal Representative (if applicable)

Fingerprint

____________________________________ DATE (DAY/MO/YR): _________________

Signature of Subject or Legal Representative

(may place an X OR fingerprint if unable to sign)

For peer review only

SAYINI YA OFUNSA MAFUNSO

Ndamufotokozera otenga nawo mbali/oyimirira otenga nawo mbali za kafukufukuyu ndipo ndayankha mafunso onse amene anali nawo. Ndikukhulupirira kuti amvetsetsa uthenga onse omwe uli chikalata cha uthengawu ndipo avomera kutenga nawo mbali mwa kufuna kwao.

____

Dzina la ofunsa mafunso kapena oyimira akafukufuku

________________________________ ____________________________

Sayini ya ofusa mafunso kapena oyimira akafukufuku Date (must be the same as subject's)

For peer review only

14.4.APPENDIX D: Written Informed Consent – Male

WRITTEN INFORMED CONSENT
Identifying efficient linkage strategies for HIV self-testing (IDEaL)
Male

You are asked to participate in a research study entitled *"Identifying efficient linkages strategies for HIVST"* conducted by Partners in Hope and the University of California Los Angeles in the United States. You are being requested to take part in the study because you recently received a self-test kit and reported recently testing HIV-positive. Your participation in this study is entirely voluntary. You will be read the information below, and you are free to ask questions about anything you do not understand, before deciding whether or not to participate. ***I as the field assistant for this study will take you through this consenting process.***

- **Why is this study being done?**

HIV self-testing is very helpful for people who want to know their status but do not usually go to the health facility. However, it can be hard for individuals who test HIV-positive with HIV self-testing to be able to access other health services. Researchers want to determine what are the best interventions that can help men who are diagnosed with HIV use other health services, if desired. The study will offer several different strategies for HIV services to see what works best for men who use HIV self-testing kits.

- **What will happen if you take part in this research study?**

There are several steps to this study. If you volunteer to participate in this study you will have the opportunity to participate in the following components:

1. Complete two study visits where a research assistant like myself will interview you and ask you information about yourself, including whether you are married, number of sexual partners, your level of education, information about your experiences with HIV services, and how you feel about HIV testing and treatment services. We will ask you questions today (or a day that is convenient for you) and in four months in order to see if anything has changed. Each interview will last about 45minutes. You can refuse a follow-up survey at any point.
2. After completing the first interview you will be randomized to one of three interventions. We will do the randomization together so you can see exactly what you will be offered. Based on what intervention you are randomly selected for, you will also be offered a variety of health services from health facility staff. The potential interventions are:

   a) Reminders about the benefits of health services;

   b) Motivational Interviewing where you can talk to someone about your life, challenges you face, and strategies to make your life and better, and additional services as needed

   c) Home-based health services whereby a provider will offer you HIV services and NCD screening at your home as a one-time event. After four weeks, you will be visited by a health care worker who will escort you to the clinic for continued health services if desired.

Regardless of what arm you are randomized to, you can always refuse health services or refuse talking to a health care provider and still remain in the study. Even if you do not plan to use any additional

health services, don't worry, you can still be in the study and we can still complete the interviews we discussed above.

3. In the first 6 months of the study, we will also review medical records at your local health facility to see if you visited the health facility since enrolling in the study. This does not require interaction with you and will be completely confidential.

4. Finally, we may contact you within 6-months of the study to complete another in-depth interview so we can learn more about your experience with the study, recommendations for future interventions, and what additional services, if any, you would like. Not all participants will be contacted for the interview and you always have the right to decline an in-depth interview – refusal will not affect your participation in the larger study.

- **How long will you be in the research study?**

All study activities will be completed within 6-months of today.

- **Are there any potential risks or discomforts that you can expect from this study?**

You will be asked a series of questions by a research assistant about your sexual history and your experience receiving and using a self-test kit from your sexual partner. You may feel uncomfortable answering some of the questions asked by the interviewer. You can say "I don't want to answer" to any questions that make you uncomfortable. All questions will be asked in a private place so that no other patients or staff will hear your answers.

If you agree to participate in the assigned intervention, providers may ask to reach you at home or in the community. As with any health service, if you choose to initiate ART you may be at risk of unwanted status disclosure.

If you experience distress or adverse events, we will provide you with counseling resources or refer you to resources for assistance.

- **Are there any potential benefits to participating?**

You will have the opportunity to discuss information about your well-being and HIV services with a Research Assistant and possibly a health care provider and in a confidential, private manner. You will also have the chance to link to HIV care services at the facility of your choosing.

- **Are there any potential benefits to society?**

Information obtained as part of this work may be of benefit to the larger Malawi program, or similar programs in sub-Saharan Africa, since the work aims to determine if there are better ways to offer HIV services to men who use HIV self-test kits. If researchers better understand what type of programs work better for men, the program in Malawi can be scaled up and strengthened to provide these specific types of increased support.

- **Will you receive payment for being part of this study?**

Your participation is entirely voluntary. You will be provided MK 7,500 (equivalent to 10USD) for each survey completed (MK 15,000 / 20USD across the duration of the study). You will receive the above compensation regardless if you use HIV services or not. Those who complete the additional in-depth interview 6-months after study enrollment will receive an additional MK 7,500 (equivalent to 10USD) for their time.

- **What is the cost of participating in this study?**

There is no cost to participate in this study.

For peer review only

- **Will information about me be kept confidential?**

Authorized representatives of the Malawi National Health Sciences Research Council who are responsible for ensuring the rules related to research are followed, may need to review records of study participants. As a result, they may see your name; but they will not to reveal your identity to others.

When the results of the research are published or discussed in meetings, no information will be included that would reveal your identity. Any paperwork related to the study which contains information about you will be kept in a locked cabinet in a locked office. Only staff members of the study will have access to this information. A code will be assigned to each individual participating in the study. This code will be stored on a computer in a locked file. The key to unlock the information will only be known by the research staff. All data entered into a computer will be entered using this code so information will no longer have any information that can identify you such as your name. Forms containing any identifying information will be destroyed two years after the study is finished.

- **Participation and Withdrawal**

Your participation in this research is VOLUNTARY. If you choose not to participate, that will not affect your relationship with the hospital, your health provider or health centre you usually get your medical care from, or your right to health care. If you decide to participate, you are free to withdraw your consent and stop your participation at any time and can still receive future health care at the hospital or health center you go to.

- **Withdrawal of Participation by the Investigator**

The research investigator may stop your participation in this research if he or she feels this is best for you. The investigators will make the decision and let you know if it is not possible for you to continue. The decision may be made to protect your health and safety.

- **Who can answer questions I might have about this study?**

In the event of a research related injury or if you experience a problem, please immediately return to the hospital or health centre you go to or contact Mike Nyirenda. The NHSRC Ministry of Health information (Dr. Kathyola) is also provided in case you have questions about your rights as a research participant.

Investigator:

Khumbo Phiri
Mobile: +265999840946
Partners in Hope Clinic
Area 36, Plot8
M1 Road South
Lilongwe, Malawi

OR

Dr.C. Mitambo
The Secretariate, NHSRC
Ministry of Health
P.O Box 30377
Lilongwe 3
Cell +265888344 443

For peer review only

SIGNATURE OF RESEARCH SUBJECT [OR LEGAL REPRESENTATIVE]

I have read (or someone has read to me) the information provided above. I have been given an opportunity to ask questions and all of my questions have been answered to my satisfaction. I have been given a copy of this form.

BY SIGNING THIS FORM, I WILLINGLY AGREE TO PARTICIPATE IN THE RESEARCH:

____

Name of Subject

____

Name of Legal Representative (if applicable)

____________________________________ DATE (DAY/MO/YR): __________________

Signature of Subject or Legal Representative
(may place an X OR fingerprint if unable to sign)

For peer review only

SIGNATURE OF INVESTIGATOR OR DESIGNEE

I have explained the research to the subject or his/her legal representative and answered all of his/her questions. I believe that he/she understands the information described in this document and freely consents to participate.

____

Name of Investigator or Designee

______________________________ __________________________

Signature of Investigator or Designee Date (must be the same as subject's)

WRITTEN INFORMED CONSENT
Identifying efficient linkage strategies for HIV self-testing (IDEaL)
Male (Chichewa)

Mukufunsidwa kuti mutenge nawo mbali mu gawo lino la kafukufuku wotchedwa"kupeza njira zoyenera zobwezeletsa anthu pa thandizo la ma ARV" amene akupangidwa ndi bungwe la Partners In Hope-EQUIP polojekiti ndi sukulu ya ukachenjede ya University of California Los Angeles yaku Amerika. Mukufunsidwa kuti mutenge nawo mbali mu gawo lakafukufukuyu chifukwa muli ndi kachirombo ka HIV komanso mwalandila kachipangizo koziyezela wekha HIVi. Kutenga nawo mbali mukafukufuku ameneyi sikokakamiza. Tikuwerengerani uthenga omwe walembedwa pansipa, ndipo muli ololedwa kufunsa mafunso aliwonse pa zomwe simukumvetsetsa,musanapange chiganizo chotenga nawo mbali kapena ayi. Ine ngati othandizira mukafufuku ameneyu ndikuthandizani pa ndondomeko yotenga chilolezo.*

Chifukwa chiyani kafukufukuyu akuchitika?

Kuziyeza wekha HIV ndi kofunika kwa anthu omwe akufuna kudziwa za momwe mthupi mwawo mulili ku mbali ya HIV koma sapita kuchipatala, kotelo, ndikovuta kwa anthu omwe apezeka ndi HIV kudzela chipangizo choziyezela wekha kuti apeze thandizo la zaumoyo. Akafukufuku akufuna apeze njira zabwino zomwe zingathe kuthandiza azibambo omwe apezeka ndi HIV kuti agwiritse ntchito thandizo lina lazaumoyo ngati angakonde kutero. Kafukufukuyu apeleka mwayi wa njira zosiyana siyana zothandizira HIV kuti aone zomwe zingagwire ntchito bwino kwa azibambo omwe amagwiritsa ntchito ka chida koziyezela wekha HIV.

Chichitike ndi chani mukatenga nawo mbali mu kafukufukuyu?

Pali ma gawo angapo omwe adzachitike mukafukuku, Ngati mungazipereke kutenga nawo mbali mu kafukufukuyu mudzatenga nawo mbali mu magawo otsatirawa:

Akafukufuku adzakuyendera kawiri. Ulendo woyamba tidzapemphani kuti mutenge nawo mbali mu kafukufuku yemwe tidzakufunseni zokhuza inuyo, kuphatikizapo ngati muli pa banja, muli ndi abwenzi ogonana nawo angati, maphunziro anu komanso zomwe munakumana nazo polandira thandizo la HIV komanso momwe mumnvera kumbali ya thandizo la HIV angakhalenso kuyezedwa HIV. Tikufunsani mafunso lelo (Ngati lelo muli okonzeka kuyankha mafuns)komanso miyezi inayi ikudzayi kuti tiwone ngati pali chomwe chasintha, kucheza kuli konse kuzitenga nthawi yosachepela makumi anayi ndi isanu. Muli ololedwa kukana kutenga nawo mbali mu kucheza kotsatira nthawi iliyonse.

2. Pamapepo pa kucheza koyamba mudzayikidwa mu imodzi mwa njira zitatu popanda ndondomeko iliyonse,pa nthawi yomwe mudzakhale mukuyikidwa mu njirayi mudzakhala muli pomwepo kuti muone njira yomwe mwapatsidwa.Potengera njira yomwe mwayikidwa mudzapatsidwanso mwayi wa mathandizo a zaumoyo angapo ochoka kwa opeleka thandizo la zaumoyo pa chiptala, zina mwa njira ndi:

a) Chikumbutso cha ubwino wa thandizo la zaumoyo

b) Kucheza kwa chilimbitso komwe mungathe ndi mwayi ocheza ndi anthu ena ndi kuwafotokozela za umoyo wanu, zofuta zomwe mumakumana nazo komanso njira zomwe mumagwiritsa ntchito kuti moyo wanu ukhale wosavuta.

c) Thandizo la zaumoyo lomwe mumatha kulandira pakhomo monga thandizo la HIV komanso NCD lomwe mumalandila kamodzi.Pakatha masaba anayi mudzayendeledwa ndi wa zaumoyo yemwe adzakupelekezeni ku chipatala komwe mukapitilize kulandira thandizo ngati mwakonda kutelo.

Posatengera njira yomwe mwapatsidwa mongathe kukana kulandira thandizo la zaumoyo koma ndikupitiliza kutenga nawo mbali mu kafukufuku ndipo tingathe kupitilizabe kucheza komwe tatchula m'mwambamu.

3. Miyezi isanu ndi umodzi yoyambilira ya kafukufuku tidzaona zambiri ya umoyo wanu ku chipatala cha m'dela lanu ngati munapitako mutalowa kale mu kafukufuku, zimenezi sizidzafuika kulankhula nanu ndipo zidzachitika mwachinsinsi.

4.Pamapeto tidzakuyendelani pakutha kwa miyezi isanu ndi umodzi ya kafukufuku kuti tidzacheze nanu komanso kuti tidzanve za momwe mukunvera za kafukufuku, maganizo anu okhudza njira zina za mtsogolo komanso, thandizo lina lowonjezera ngati ilipo. Si onse otenga nawo mbali omwe adzaonedwe ndipo muli ololedwa kukana kutenga nawo mbali mukucheza ndipo kukana kwanu sikudzaononga mwayi wanu otenga nawo mbali mu study.

Mutenga nthawi yaitali bwanji muli mukafukufuku?

Zochitika zonse zakafukufuku zizamalizidwa pa miyezi isanu ndi umodzi (6) kuchokera lero.

Pali zinthu zosowetsa mtendere kapena zodetsa nkhawa zomwe mungayembekezere kuchokera mu kafukufukuyu?

Muzafunsidwa mndandanda wa mafunso ndi othandizira mukafukufuku ameneyi zokhuza mbiri yanu pankhani zogonana ndi zochitika mutalandira ka chida koziyeza wekha kuchokera kwa bwezi wanu ogonana naye komanso mmene munagwiritsira ntchito. Mutha kukhala osamasuka poyankha mafunso ena omwe wofunsa mafunso angafunse. Mutha kunena kuti "sindikufuna kuyankha" kufunso lilironse lomwe simukumasuka nalo. Mafunso onse azafunsidwa pa malo achinsinsi pomwe odwala anzanu kapena ogwira ntchito pachipatala sadzamva nawo mayankho anu.

Ngati mudzavomere kutenga nawo mbali mu imodzi mwa njira, a zaumoyo adzakufunsani kuti akupezeni kunyumba kwanu kapena mu dela lanu. Monga mwa thandizo lililonse la zaumoyo, ngati mungavomere kuyamba kumwa mankhwala a ma ARV mungathe kukhala pa chiospyezo choulula za momwe mulili m'mthupi mwano mosafuna.

Ngati mungakumane ndi masautso aliwonse, tidzakupatsani uphungu kapena kukutumizani koti mukathandizidwe ndi uphungu.

Pali cholowa chilichonse potenga nawo mbali mukafukufukuyu?

Mudzakhala ndi mwayi okambilana ndikunva zambiri zokhudza moyo wanu komanso thandizo la HIV ndi othandiza kafukufuku komanso dotolo munjira yachinsisi. Mudzakhalanso ndi mwayi oyamba mankhwala a ma ARV ku chipatala cha kufuna kwanu.

Pali cholowa chilichonse kwa anthu a mudera?

For peer review only

Uthenga womwe udzatengedwe ngati mbali imodzi yakafukufukuyu uzakhala othandiza mu mapologalamu a dziko la Malawi, kapena ma pologalamu ena ofananirapo a kum'mwera kwa Africa, chifukwa choti ntchito imeneyi ikufuna kuona ngati pali njira yabwino yopeleka thandizo la HIV mwa azibambo, ma pologalamu aku Malawi azapita patsogolo ndikulimbikitsa njira zopititsira patsogolo.

Kodi mulandira malipiro potenga nawo mbali mukafukufukuyu?

Kutenga nawo mbali kosakakamiza. Mudzalandira chiongola dzanja cha ndalama zokwana 7,500(Pafupifupi MK 15,000/20USD). Mudzalandila ndalamazi olo mutakhala kuti simunatenge nawo kapena mwatenga nawo mbali mu thandizo la HIV. Kwa omwe adzamalize nawo kucheza kowonjezera patatha miyezi isanu ndi umodzi adzalandira ndalama yowonjezera yokwana MK 7,500 (yokwana pafupi fupi 10 USD0 chifukwa cha nthawi yawo.

Kodi pali kulipira kulikonse chifukwa chotenga nawo mbali mukafukufukuyu?

Kutenga nawo mbali mukafukufukuyu ndi kwaulere.

Kodi uthenga wanga uzasungidwa mwa chinsinsi?

Anthu ogwira nawo ntchito mu kafukufuku okhawo ndi amene adziwe za uthenga wanu kapena chilichonse chomwe mutiuze pa kafukufukuyu.

Anthu ovomerezeka oyimirira bungwe la National Health Sciences Research Council ndi UCLA office for Protection Of Research Subjects ndi amene ali ndi udindo woonetsetsa kuti malamulo a kafukufuku akutsatidwa, akhoza kufuna kuona nawo kaundula wa anthu amene akutenga nawo mbali mukafukufukuyu. Kutanthauza kuti akhonza kuzaona dzina lanu; koma sangaulure zokhuza inu kwa anthu ena.

Pa nthawi imene zotsatira za kafukufukuyu zidzatsindikidwa kapena kukambidwa mu misonkhano, palibe uthenga womwe udzayikidwe wokuzindikiritsani. Uthenga uliwonse wolembedwa pa pepala wokhunza inu uzasungidwa mu kabati yokhoma mu ofesi yokhomanso. Anthu ogwira nawo ntchito mukafukufukuyu okhawo ndi amene azathe kuona uthenga umenewu. Njira yotanthauzira uthenga umenewu izadziwika ndi anthu akafukufukuyu basi. Uthenga onse olowetsedwa pa makina a kompyuta uzalowetsedwa kugwiritsa ntchito nambala, ndekuti uthenga onse sudzakhalanso ndi zokuzindikiritsani monga dzina lanu. Ma pepala amene pali uthenga okuzindikitsani adzawonongedwa pakapita zaka ziwiri chimalizireni kafukufukuyu.

Kutenga nawo mbali mu kafukufukuyu SIKOKAKAMIZA. Ngati musankhe kuti simutenga nawo mbali, izi sizingasokoneze ubale wanu ndi chipatala chino, anthu ogwira ntchito kuchipatala,kapena chipatala chomwe mumalandilirako chithandizo, kapena ufulu wanu wolandira thandizo la chipatala. Ngati mwapanga chisankho chotenga nawo mbali, mukhoza kuchosa chilolezo chanu ndikusiya kutenga nawo mbali nthawi ina iliyonse ndipo mungathe kuzalandirabe chithandizo pa chipatala pano mtsogolo.

Kusiyitsidwa kutenga nawo mbali mu kafukufuku ndi wakafukufuku

Anthu opangitsa kafukufukuyu akhonza kukuletsani kutenga nawo mbali mukafukufukuyu akaona kuti ndi bwino kuti mutero. Anthu akafukufukuwa azapanga chiganizochi ndikukudziwitsani kuti sizitheka kuti mupitirize. Chiganizochi chitha kupangidwa kuti ateteze thanzi ndi chitetezo chanu.

For peer review only

Kusiyitsidwa kutenga nawo mbali mu kafukufuku ndi wakafukufuku

Anthu opangitsa kafukufukuyu akhonza kukuletsani kutenga nawo mbali mukafukufukuyu akaona kuti ndi bwino kuti mutero. Anthu akafukufukuwa azapanga chiganizochi ndikukudziwitsani kuti sizitheka kuti mupitirize. Chiganizochi chitha kupangidwa kuti ateteze thanzi ndi chitetezo chanu.

Investigator:

Khumbo Phiri
Mobile: +265999840946
Partners in Hope Clinic
Area 36, Plot8
M1 Road South
Lilongwe, Malawi

OR

Dr.C. Mitambo
The Secretariate, NHSRC
Ministry of Health
P.O Box 30377
Lilongwe 3
Cell +265888344 443

Wotenga mbali kapena omuyimira asayine

Ndawerenga (kapena munthu wina wandiwerengera) zonse zalembedwa mwambamu. Ndapatsidwa mwayi wofunsa mafunso ndi mafunso onse ndinafunsa ayankhidwa ndipo ndakhutitsidwa. Ndapatsidwa pepala lina langati lomweyi.

____

Dzina la otenga mbali

____

Dzina la oyimola malamulo

____________________________________ Tsiku: __________________

Wotenga mbali kapena oyimira malamulo asayine

(Mutha kuyika X ngati simungathe kusayinila)

Ofufuza asayinire/Kapena othandizila Kafukufuku

Ndafotokoza za kafukufuku uyu kwa otenga nawo mbali kapena owayimila, ndayankhanso mafunso awo onse. Ndikukhulupilira kuti amvetsetsa zomwe zananedwa mu chikalata ichi

For peer review only

ndiponso avomereza mwakufuna kwawo kutenga nawo mbali.

____

Dzina la ofufuza kapena othandizila kafukufuku

________________________________ ______________________________

Ofufuza kapena othandizila kafukufuku asayine apa Tsiku

For peer review only

14.5. APPENDIX E: Baseline Survey – Male

BASELINE SURVEY
Identifying efficient linkage strategies for HIV self-testing (IDEaL)
Male

**Question Name**	**Label**	**Responses**
INTRODUCTION SECTION		
interviewer	Full Name of Interviewer	
Interview date	Interview date	
Time start	Time survey started	
District	District	
TA	TA	
village	Village	
SECTION A: DEMOGRAPHICS		
Intro Note	Thank you for agreeing to participate. Now I will ask you a few questions about yourself and who you are. Please feel free to answer honestly. There are no right or wrong answers.	
a7	What is your tribe?	1. Lomwe<br>2. Sena<br>3. Chewa<br>4. Mang'anja/Nyanja<br>5. Ngoni<br>6. Tumbuka<br>7. Tonga<br>8. Yao

For peer review only

		99. Other, specify
a3	What is the highest level of school you attended?	1. Primary<br>2. Secondary<br>3. Higher
a3b	What class did you complete in your highest level of school?	____________________
a4	Please think of the past 12months, how would you describe your primary occupation?	1. Working formally (employed full time)<br>2. Working informally (ganyu, farming, business)<br>3. Not working
a5	Are you currently married?	1. Married<br>2. Live-in partner<br>3. Steady Girlfriend/Boyfriend<br>4. Separated<br>5. Divorced<br>99. Other, specify
a6	How many living children do you have?	____________________
a6b	What is the age of your youngest child?	____________________
a6bc	What age is the child (in years or months)	____________________
a4b	How many children currently live with you?	____________________
a7	How many sexual partners have you had in the past 12 months?	____________________
a8	Have you had sex with someone besides your wife/husband without a condom in the past 12 months?	1. Yes<br>0. No<br>88. Don't know/ Not sure<br>89. Refused to answer

For peer review only

a8b	Have you had sex without a condom in the past 12 months?	1. Yes<br>0. No<br>88. Don't know/ Not sure<br>89. Refused to answer
SECTION B: INCOME QUESTIONS		
Intro Note	I will now discuss with you about the valuable items that you or your household possesses. As I will be chatting with you I will also some questions about money you have and activities that you indulge in to find money.	
b1	Please think of the past 12 months, how would you describe your primary occupation?	1. Working formally (employed full time)<br>2. Working informally (ganyu, farming, business)<br>3. Not working
b1b	Think about all the work you have done in the past month. How many days did you normally work this month that gave you pay?	________________
b2	Do you have any savings for the future, such as a bank account, savings group or cash?	1. Yes<br>0. No
Household Assets		
b3	Does your household have:<br>*The respondent said that his/her household doesn't have any of the household assets. Please probe and ensure that this is correct before you proceed.*	
b3_1	Metal Roof?	1. Yes<br>0. No
b3_2	Electricity?	1. Yes

For peer review only

		0. No
b3_3	Paraffin lamp with no glass?	1. Yes<br>0. No
b3_4	A paraffin lamp?	1. Yes<br>0. No
b3_5	A radio?	1. Yes<br>0. No
b3_6	A television?	1. Yes<br>0. No
b3_7	A cellular phone?	1. Yes<br>0. No
b3_8	A bed?	1. Yes<br>0. No
b3_9	A sofa set?	1. Yes<br>0. No
b3_10	A table?	1. Yes<br>0. No
b3_11	A refrigerator	1. Yes<br>0. No
b3_12	Mattress?	1. Yes<br>0. No
b3_13	Chair(s)?	1. Yes<br>0. No
b3_14	Cattle?	1. Yes<br>0. No
b3_15	Goat?	1. Yes<br>0. No
b3_16	Sheep?	1. Yes<br>0. No
b3_17	Pigs?	1. Yes<br>0. No

For peer review only

b3_18	Donkey?	1. Yes<br>0. No
b3_19	Chickens?	1. Yes<br>0. No
b3_20	Other poultry?	1. Yes<br>0. No
b4	In the past 30 days, have you drank beer?	1. Yes<br>0. No
b4b	How many days in the past 30 days have you drank beer?	______________________
b4c	How much money did you spend on beer the last time you went?	MWK:
b4d	In total, approximately how much money did you spend on beer in the past 30 days?	MWK:
Relationship		
Intro Note	Now I'd like to talk to you about your current sexual relationship	
f8	How long have you been/were you in a sexual relationship with your partner?	Days______________<br>Months_____________<br>Years_______________
f9	Do you have children with your partner? How many children?	______________________
f10	How often do you currently talk to your partner?	1. Everyday<br>2. A couple times a week<br>3. Once a week<br>4. A couple times a month<br>5. Once a month<br>6. Less than once a month<br>7. Not at all (never)
f10b	In a typical month, who earns more money? You, or your partner?	1. Myself<br>2. This partner<br>3. We earn the same amount<br>88. Don't know

For peer review only

Decision Making		
Intro Note	Now I would like to talk to you about how you and your partner make decisions.	
f11	Who usually decides how the money you earn will be used?	1. Yourself (Respondent)<br>2. Jointly (This partner and you together)<br>3. Mainly this partner<br>4. Someone else<br>5. Do not earn money<br>88. Refuse to say
f11b	(if above question=4) Who decides?	______________________
f12	Who usually decides how your partner's earnings will be used?	1. Yourself (Respondent)<br>2. Jointly (This partner and you together)<br>3. Mainly this partner<br>4. Someone else<br>5. Do not earn money<br>88. Refuse to say
f12b	(if above question=4) Who decides?	______________________
f13	Who usually makes decisions about health care for yourself?	1. Yourself (Respondent)<br>2. Jointly (This partner and you together)<br>3. Mainly this partner<br>4. Someone else<br>5. Not applicable/ Don’t have children<br>88. Refuse to say
f13b	(if above question=4) Who decides?	______________________
f14	Who usually makes decisions about health care for your child with this partner?	1. Yourself (Respondent)<br>2. Jointly (This partner and you together)<br>3. Mainly this partner<br>4. Someone else

For peer review only

		5. Not applicable/ Don't have children<br>88. Refuse to say
f14b	(if above question=4) Who decides?	______________________
f15	Who usually makes decisions about health care for your partner?	1. Yourself (Respondent)<br>2. Jointly (This partner and you together)<br>3. Mainly this partner<br>4. Someone else<br>5. Not applicable/ Don't have children<br>88. Refuse to say
f15b	(if above question=4) Who decides?	______________________
f16	Who usually makes decisions about making major household purchases?	1. Yourself (Respondent)<br>2. Jointly (This partner and you together)<br>3. Mainly this partner<br>4. Someone else<br>5. Not applicable/ Don't have children<br>88. Refuse to say
f16b	(if above question=4) Who decides?	
Note	I would like to ask you questions about [probability/chance/likelihood] that certain things will happen. There are ten beans in this cup. I will ask you to pick some of the beans and put them in the plate. The number of beans that you are going to put in the plate will reflect the probability that something will happen. One bean means there is very little chance that something will happen. If you do not put any bean in the plate it means you are certain that there is no likelihood that something will happen.	

For peer review only

note2	If you put additional beans in the plate it means the chance that something will happen will also increase. For example, if you put one or two beans in the plate, it means there is little chance that something will happen. Even though there is little chance but it can happen. If you put ten beans it means there is equal chance of something happening or not. If you put six beans it means the chance that something will happen is slightly greater than not happening. If you put all ten beans, it means you are certain that whatever the case something will really happen. There is no wrong or right answer I just want to know what you think.	
note3	INTERVIEWER: Report for each question the NUMBER OF BEANS put in the PLATE. After each question, replace the beans on the table (unless otherwise noted).	
	Practice	
pr1	Pick the number of beans that reflects how likely you think it is that:	________________
pr1b	You will go to the market at least once within the next 2 days.	________________
pr1c	You will go to the market at least once within the next 2 weeks.	________________
	Practice	
pr2	INTERVIEWER: Did Respondent add any beans between pr1b and pr1c?	1. Yes<br>0. No

For peer review only

pr3	Remember, as time goes by, you may find more time to go to the market. Therefore, you should have added beans to the plate. Let me ask you again. Now, add beans in the plate so that the number of beans in the plate reflects how likely you think it is that you will go to the market at least once within 2 weeks.<br><br>How likely you think it is that you will go to the market at least once within 2 weeks?	____________________
f17	Pick the number of beans that reflects how likely you think:	
f17b	You will still be married/with [partner one year from now.	____________________
f17c	You are currently infected with HIV/AIDS	____________________
f17d	You will become infected with HIV/AIDS during the next 12 months	____________________
f17e	You will become infected with HIV/AIDS during their lifetime	____________________
f17c	partner is infected with HIV/AIDS now.	____________________
f17d	partner will become infected with HIV/AIDS during the next 12 months.	____________________
SELF REPORTED HEALTH AND HAPPINESS		
Intro Note	Now I'd like to talk to you about how healthy and happy you feel.	
e1	I am interested in your general level of well-being or satisfaction with life. How satisfied are you with your life, all things considered?	1. Very satisfied<br>2. Somewhat satisfied<br>3. Neutral<br>4. Somewhat unsatisfied<br>5. Very unsatisfied

For peer review only

e2	Do you think that you are more, equally or less satisfied than other persons your age and sex living in your village?	1. More satisfied<br>2. Equally satisfied<br>3. Less satisfied
e3	In general, would you say your health now is: very good, good, poor or very poor?	1. Very good<br>2. Good<br>3. Poor<br>4. Very poor
e4	How would you compare your health to other people of the same age and sex in your village?	1. More healthy<br>2. Equally healthy<br>3. Less healthy
e5	In the past month, how many days were you too sick to work/go to school/complete household chores?	___________________
	Happiness	
e6	How true are the following statements for you in the last month?	
e6_1	I have felt depressed	1. Strongly Agree<br>2. Agree<br>3. Disagree<br>4. Strongly Disagree
e6_2	I have felt life was not worth living	1. Strongly Agree<br>2. Agree<br>3. Disagree<br>4. Strongly Disagree
e6_3	I have felt content.	1. Strongly Agree<br>2. Agree<br>3. Disagree<br>4. Strongly Disagree
e6_4	I have felt lonely	1. Strongly Agree<br>2. Agree<br>3. Disagree<br>4. Strongly Disagree
	GENDER EQUITABLE MEN SCALE	
Note	Please tell me if you strongly agree, agree, disagree, or strongly disagree with the following statements:	

For peer review only

j1	Woman's most important role is to take care of her home and cook (take care of home is about housekeeping)	1. Strongly Agree<br>2. Agree<br>3. Unsure<br>4. Disagree<br>5. Strongly Disagree<br>88. Refuse to say
j2	Men need sex more than women	1. Strongly Agree<br>2. Agree<br>3. Unsure<br>4. Disagree<br>5. Strongly Disagree<br>88. Refuse to say
j3	Men don't talk about sex, they just do it.	1. Strongly Agree<br>2. Agree<br>3. Unsure<br>4. Disagree<br>5. Strongly Disagree<br>88. Refuse to say
j4	There are times when a woman deserves to be beaten	1. Strongly Agree<br>2. Agree<br>3. Unsure<br>4. Disagree<br>5. Strongly Disagree<br>88. Refuse to say
j5	Changing diapers, giving kids a bath & feeding kids are mother's responsibility	1. Strongly Agree<br>2. Agree<br>3. Unsure<br>4. Disagree<br>5. Strongly Disagree<br>88. Refuse to say
j6	It is a woman's responsibility to avoid getting pregnant	1. Strongly Agree<br>2. Agree<br>3. Unsure<br>4. Disagree<br>5. Strongly Disagree<br>88. Refuse to say
j7	A man should have the final word about decisions in his home	1. Strongly Agree<br>2. Agree<br>3. Unsure<br>4. Disagree

For peer review only

		5. Strongly Disagree<br><br>88. Refuse to say
j8	Men are always ready to have sex	1. Strongly Agree<br>2. Agree<br>3. Unsure<br>4. Disagree<br>5. Strongly Disagree<br><br>88. Refuse to say
j9	A woman should tolerate violence in order to keep her family together	1. Strongly Agree<br>2. Agree<br>3. Unsure<br>4. Disagree<br>5. Strongly Disagree<br><br>88. Refuse to say
j10a	I would be outraged if my wife asked me to use a condom.	1. Strongly Agree<br>2. Agree<br>3. Unsure<br>4. Disagree<br>5. Strongly Disagree<br><br>88. Refuse to say
j10b	Men would be outraged if their wife asked them to use a condom	1. Strongly Agree<br>2. Agree<br>3. Unsure<br>4. Disagree<br>5. Strongly Disagree<br><br>88. Refuse to say
j11	A man and a woman should decide together what type of contraceptive to use	1. Strongly Agree<br>2. Agree<br>3. Unsure<br>4. Disagree<br>5. Strongly Disagree<br><br>88. Refuse to say
j12	I would never have a homosexual friend	1. Strongly Agree<br>2. Agree<br>3. Unsure<br>4. Disagree<br>5. Strongly Disagree<br><br>88. Refuse to say

For peer review only

j13a	If someone insults me, I will defend my reputation, with force if I have to.	1. Strongly Agree<br>2. Agree<br>3. Unsure<br>4. Disagree<br>5. Strongly Disagree<br><br>88. Refuse to say
j13b	If someone insults a man, he should defend his reputation, with force if he has to	1. Strongly Agree<br>2. Agree<br>3. Unsure<br>4. Disagree<br>5. Strongly Disagree<br><br>88. Refuse to say
j14	To be a man you need to be tough.	1. Strongly Agree<br>2. Agree<br>3. Unsure<br>4. Disagree<br>5. Strongly Disagree<br><br>88. Refuse to say
j15	Men should be embarrassed if unable to get an erection	1. Strongly Agree<br>2. Agree<br>3. Unsure<br>4. Disagree<br>5. Strongly Disagree<br><br>88. Refuse to say
j16	If a guy gets a woman pregnant, child is the responsibility of both the man and woman	1. Strongly Agree<br>2. Agree<br>3. Unsure<br>4. Disagree<br>5. Strongly Disagree<br><br>88. Refuse to say
j17	A man should know what his partner likes during sex	1. Strongly Agree<br>2. Agree<br>3. Unsure<br>4. Disagree<br>5. Strongly Disagree<br><br>88. Refuse to say
j18	The participation of the father is important in raising children	1. Strongly Agree<br>2. Agree<br>3. Unsure<br>4. Disagree

For peer review only

		5. Strongly Disagree<br>88. Refuse to say
j19	It's important for men to have friends to talk about their problems	1. Strongly Agree<br>2. Agree<br>3. Unsure<br>4. Disagree<br>5. Strongly Disagree<br>88. Refuse to say
j20	A couple should decide together if they want to have children.	1. Strongly Agree<br>2. Agree<br>3. Unsure<br>4. Disagree<br>5. Strongly Disagree<br>88. Refuse to say
STIGMA		
Note	In this next section I'd like to discuss your thoughts about people living with HIV in your community. Please feel free to talk openly, there is no right or wrong answer. I am interested in your own thoughts.	
i3	I would buy fresh vegetables from a shopkeeper or vendor if I knew that this person had HIV	1. Strongly Agree<br>2. Agree<br>3. Neutral<br>4. Disagree<br>5. Strongly Disagree
i4	If a member of my family became sick with AIDS, I would be willing to care for her or him in our own household	1. Strongly Agree<br>2. Agree<br>3. Neutral<br>4. Disagree<br>5. Strongly Disagree
i5	In my opinion, if a female teacher has HIV but is not sick, she should be allowed to continue teaching in the school	1. Strongly Agree<br>2. Agree<br>3. Neutral<br>4. Disagree<br>5. Strongly Disagree
EXPECTATIONS		

For peer review only

Intro Note	I would like to ask you questions about [probability/chance/likelihood] that certain things will happen. There are ten beans in this cup. I will ask you to pick some of the beans and put them in the plate. The number of beans that you are going to put in the place will reflect the probability that something will happen. One beans means there is very little chance that something will happen. If you do not put any bean in the plate it means you are certain that there is no likelihood that something will happen.	
h4	Pick the number of beans that reflects how likely you think it is that:	____________________
h4b	You will have to rely on family members for financial assistance in the next 12 months.	____________________
h4c	You will have to provide some family members with financial assistance in the next 12 months.	____________________
Note	Next, I would like to ask you a few questions about what you expect in the future. I know that nobody knows for sure what the future may bring, but let`s just talk about your best guess.	
h5	In the next year how likely is it that you will:	
h5a	You will be enrolled in school one year from now	____________________
h5b	Start a new business?	____________________
h5c	Open a bank account?	____________________
h5d	Buy land?	____________________

For peer review only

h5e	Save money?	____________________
h5f	Experience shortage of food?	____________________
h5g	Have steady work?	____________________
	Tested for HIV	
pd2	Approximately how many times have you ever been tested for HIV?<br>*Enter "-99" if client doesn't remember*	____________________
pd3	When was the last time you were tested for HIV?	a. Year______<br>b. Month________
pd5	Think about the very first time you received an HIV+ test result. Since that very first HIV+ test result, have you ever tested for HIV again (excluding a confirmatory test)?	1. Yes<br>0. No
pd6	Have you ever initiated ART?	1. Yes<br>0. No
	First Initiated ART	
pd6b	When did you first initiate ART?	a. Year_______<br>b. Month____
pd7	Have you ever been >14 days late for an ART appointment?	1. Yes<br>0. No
pd7b	How many times?	____________________
pd8	Do you know anyone who is on ART?	1. Yes<br>0. No
pd8b	Now think about the person on ART who you are closest with.<br><br>How often do you talk with them	1. Everyday<br>2. A couple times a week<br>3. Once a week<br>4. A couple times a month

For peer review only

	about ART?	5. Once a month<br>6. Less than once a month<br>7. Not at all (never)
pd9	Have you disclosed your HIV status to anyone besides your partner?	1. Yes<br>0. No
pd9b	Who else did you disclose to?<br>*Mark all that apply*	1. Sister<br>2. Brother<br>3. Father<br>4. Mother<br>5. Uncle<br>6. Aunt<br>7. Friend<br>8. Mother-in-Law<br>9. Father-in-Law<br>10. My children<br>11. Employee<br>12. Other sexual partner<br>99. Other, specify_____
pd10	Of those people you disclosed to, who do you talk to most often?	1. Sister<br>2. Brother<br>3. Father<br>4. Mother<br>5. Uncle<br>6. Aunt<br>7. Friend<br>8. Mother-in-Law<br>9. Father-in-Law<br>10. My children<br>11. Employee<br>12. Other sexual Partner<br>99. Other, specify_____
pd10c	How often do you talk to that person?	1. Everyday<br>2. A couple times a week<br>3. Once a week<br>4. A couple times a month<br>5. Once a month<br>6. Less than once a month<br>7. Not at all (never)

For peer review only

PREVIOUS USE OF HEALTH SERVICES		
Intro Note	Now I'd like to talk to you about your experience with using health services at health facilities.	
h1	Have you gone to a health facility in the past 12 months (either for yourself or someone else - AKA as a guardian)?	1. Yes<br>0. No
h2	How many times have you gone to the health facility in the past 12 months?	______________________
h3	Now think about yourself specifically. How many times have you gone to a health facility in the past 12 months for your own health care?	______________________
h_a1	When was the last time (the YEAR) you went to a health facility for YOUR OWN health?<br>NOTE: PUT WHAT YEAR. (i.e., 2015). If DO NOT REMEMBER, help them estimate. IF NEVER GONE, put -99	______________________
h4	What services did you receive at your last health facility visit for your own health?	1. ANC<br>2. Family Planning<br>3. Delivery<br>4. Post-natal<br>5. Under Five<br>6. HTC<br>7. ART<br>8. Feeling sick (OPD)<br>9. Injury (OPD)<br>10. Dentist<br>11. None<br>99. Other specify_____
h_a2	Now think about the SECOND most recent time you went to a health facility for YOUR OWN health. What year did you go to the health facility?	______________________
c3	Now please think about your MOST RECENT visit to a health facility, excluding today. When did you go?	a. Year___________<br>b. Month_________

c4	Which facility did you go to?	1. Current facility<br>0. Other facility, specify________________
c5	What was the main service you went for?	1. ANC<br>2. Family Planning<br>3. Delivery<br>4. Post-natal<br>5. Under Five<br>6. HTC<br>7. ART<br>8. Feeling sick (OPD)<br>9. Injury (OPD)<br>10. Dentist<br>11. None<br>99. Other specify_____
c5b	Who received services?	1. Myself<br>2. My child<br>3. My partner<br>4. Another family member<br>5. A friend<br>99. Other, specify_______
c6a	Did you (or the person you came with) receive another service?	1. Yes<br>0. No
c6	What was the second service you went for?	1. ANC<br>2. Family Planning<br>3. Delivery<br>4. Post-natal<br>5. Under Five<br>6. HTC<br>7. ART<br>8. Feeling sick (OPD)<br>9. Injury (OPD)<br>10. Dentist<br>11. None<br>99. Other specify_____
c6b	Who received services?	1. Myself<br>2. My child<br>3. My partner<br>4. Another family member<br>5. A friend<br>99. Other, specify_______

For peer review only

Service Satisfaction		
c10	Now I would like to talk to you about your satisfaction with the services you received that day. Please tell me whether any of these were problems for you at the VISIT YOU ARE THINKING ABOUT NOW, and if so, whether they were major or minor problems for you.	
c10_1	Time you waited to see a provider	1. Major<br>2. Minor<br>0. No-problem<br>88. Not applicable<br>89. Don't know
c10_2	Ability to discuss problems or concerns about your pregnancy	1. Major<br>2. Minor<br>0. No-problem<br>88. Not applicable<br>89. Don't know
c10_3	Amount of explanation you received about the problem or treatment	1. Major<br>2. Minor<br>0. No-problem<br>88. Not applicable<br>89. Don't know
c10_4	Privacy from having others see the examination	1. Major<br>2. Minor<br>0. No-problem<br>88. Not applicable<br>89. Don't know
c10_5	Privacy from having others hear your consultation discussion	1. Major<br>2. Minor<br>0. No-problem<br>88. Not applicable<br>89. Don't know
c10_6	Availability of medicines at this facility	1. Major<br>2. Minor

For peer review only

		0. No-problem<br>88. Not applicable<br>89. Don't know
c10_7	The hours of service at this facility, i.e., when they open and close	1. Major<br>2. Minor<br>0. No-problem<br>88. Not applicable<br>89. Don't know
c10_8	The number of days services are available to you	1. Major<br>2. Minor<br>0. No-problem<br>88. Not applicable<br>89. Don't know
c10_9	The cleanliness of the facility	1. Major<br>2. Minor<br>0. No-problem<br>88. Not applicable<br>89. Don't know
c10_10	How the staff treated you	1. Major<br>2. Minor<br>0. No-problem<br>88. Not applicable<br>89. Don't know
c10_11	Cost for services or treatments	1. Major<br>2. Minor<br>0. No-problem<br>88. Not applicable<br>89. Don't know
Satisfaction		
c11	In general, which of the following statements best describes your opinion of the services you either received or were provided at the facility	1. I am very satisfied with the services I received<br>2. I am satisfied with the services I received<br>3. I am not satisfied with the services I received

		4. I am very dissatisfied with the services I received
c12	Did you recommend this health facility to a friend or family member?	1. Yes<br>0. No
Comment	We have reached the end of the chat. Thank you for your time. Do you have anything else you would like to say?	
End Note	Thank the participant for their time and give them transport reimbursement, if they did not come for an ART appointment	
Comments	Enumerator comments	
End of the survey!		

For peer review only

BASELINE SURVEY
Identifying efficient linkage strategies for HIV self-testing (IDEaL)
Male (Chichewa)

**Question Name**	**Label**	**Responses**
INTRODUCTION SECTION		
interviewer	Full Name of Interviewer	
Interview date	Interview date	
Time start	Time survey started	
District	District	
Facility	Facility	
ID	ID	
SECTION A: DEMOGRAPHICS		
Intro Note	Zikomo povomera kutenga nawo mbali. Pa nthawi ino ndikufunsani ma funso ochepa okhudza za inu komanso kuti ndinu ndani. Chonde khalani omasuka kuyankha moona. Palibe yankho lokhoza komanso lolakwa.	
a7	Ndinu mtundu wanji wa munthu?	9. Lomwe<br>10. Sena<br>11. Chewa<br>12. Mang'anja/Nyanja<br>13. Ngoni<br>14. Tumbuka

For peer review only - http://bmjopen.bmj.com/site/about/guidelines.xhtml

		15. Tonga<br>16. Yao<br>99. Other, specify
a3	Kodi maphunziro anu mudapita nawo patali bwanji?	1. Pulayimale<br>2. Sekondale<br>3. Koleji
a3b	Ndi kalasi liti munamaliza ya maphunziro anu apamwamba?	________________
a4	Chonde ganizani za masabata khumi ndi awiri apitawa, mungafotokoze bwanji za ntchito yomwe mumagwira?	1. Ntchito yokhazikika (ya nthawi yayitali)<br>2. Ntchito yosakhazikika (ganyu, ulimi, bizinesi)<br>3. Sindikugwira ntchito
a5	Kodi pakadali pano muli pa banja?	1. Pa banja<br>2. Kukhala limodzi ngati banja<br>3. Chibwezi Chokhazikika<br>4. Tinasiyana<br>5. Banja linatha<br>99. Zina
a6	Muli ndi ana angati amoyo?	________________
a6b	Mwana wanu wang'ono ali ndi zaka zingati?	________________
a6bc	Mwana wanu wang'ono ali ndi zaka zingati?	________________
a4b	Pakadali pano ana omwe mumakhala nawo ndi angati?	________________
a7	Pa miyezi khumi ndi iwiri yapitayi mwakhala ndi abwenzi ogonana nawo angati?	________________
a8	Mwakhalapo ndi bwenzi logonana kupatula akazi anu?amuna anu osagwiritsa ntchito kondomu mu miyezi khumi ndi awiri yapitayi?	1. Eya<br>2. Ayi<br>88. Sindikudziwa<br>89. Akana kuyankha

For peer review only

a8b	Mwagonanapo ndi munthu osagwiritsa ntchito kondomu mu miyezi khumi ndi awiri yapitayi?	1. Eya<br>2. Ayi<br>88. Sindikudziwa<br>89. Akana kuyankha
SECTION B: INCOME QUESTIONS		
Intro Note	Pa nthawi ino ndikufunsani za zipangizo zomwe inu komanso apabanja panu alinazo. Mkati mwakucheza kwathu ndikufunsaninso za ndalama zomwe mulinazo komanso komanso zomwe mumachita kuti mupeze ndalama.	
b1	Chonde ganizirani za miyezi Khumi ndi iwiri yapitayi, mungafotokoze bwanji za ntchito yomwe mumagwira?	4. Ntchito yokhazikika<br>5. Ganyu/bisinesi<br>6. Sindikugwira ntchito
b1b	Ganizani za ntchito zonse mwagwira mwezi watha. Mwagwira masiku angati olipidwa?	__________________
b2	Muli ndi ndalama zilizonse zomwe mukusungira za mtsogolo monga, ku banki, gulu losugira ndalama kapena ndalama zosunga kunyumba?	2. Eya<br>3. Ayi
Household Assets		
b3	Does your household have:<br>*The respondent said that his/her household doesn't have any of the household assets. Please probe and ensure that this is correct before you proceed.*	
b3_1	Denga la malata?	1. Eya<br>2. Ayi
b3_2	Magetsi ?	1. Eya<br>2. Ayi
b3_3	Koloboyi?	1. Eya<br>2. Ayi
b3_4	Nyali?	1. Eya<br>2. Ayi

For peer review only

b3_5	Wailesi?	1. Eya<br>2. Ayi
b3_6	Kanema?	1. Eya<br>2. Ayi
b3_7	Lamya ya M'manja?	1. Eya<br>2. Ayi
b3_8	Kama?	1. Eya<br>2. Ayi
b3_9	Sofa?	1. Eya<br>2. Ayi
b3_10	Tebulo?	1. Eya<br>2. Ayi
b3_11	FIliji?	1. Eya<br>2. Ayi
b3_12	Matilesi?	1. Eya<br>2. Ayi
b3_13	Mipando	1. Eya<br>2. Ayi
b3_14	Ng'ombe?	1. Eya<br>2. Ayi
b3_15	Mbuzi?	1. Eya<br>2. Ayi
b3_16	Nkhosa?	1. Eya<br>2. Ayi
b3_17	Nkhumba?	1. Eya<br>2. Ayi
b3_18	Bulu?	1. Eya<br>2. Ayi
b3_19	Nkhuku?	1. Eya<br>2. Ayi
b3_20	Zoweta zina	1. Eya<br>2. Ayi
b4	Mu masiku makumi atatu apitawa mwamako mowa?	1. Eya<br>2. Ayi
b4b	Pa masiku makumi atatu apitawa mwamwa mowa masiku angati?	____________
b4c	Mwataya ndalama zingati masiku omaliza omwe munapita ku mowa?	MWK:
b4d	Zonse pamodzi, mwataya ndalama zingati pa mowa mu masiku makumi atatu apitawa?	MWK:
Relationship		

For peer review only

Intro Note	Panthawi ino ndikufunsani za abwezi ogonana nawo?	
f8	Mwakhala pa ubwenzi ogonana ndi bwenzi lanu kwa nthawi yayitali bwanji?	Masiku ___________<br>Miyezi _____________<br>Zaka________________
f9	Muli ndi ana ndi bwenzi lanu logonana nalo? Ana angati?	________________________
f10	Mumayankhulana mowirikiza bwanji ndi bwenzi lanu pakadali pano?	1. Tsiku ndi tsiku<br>2. Masiku angapo pasabata<br>3. Kamodzi pa sabata<br>4. Kangapo pa mwezi<br>5. Kamodzi pa mwezi<br>6. Kosakwana mwezi<br>7. Sitiyankhulani
f10b	Pa mwezi amalandila ndalama zambiri ndi ndani? Inu kapena bwenzi lanu?	1. Ine<br>2. Bwenzi langa<br>3. Timalandira ndalama zofanana<br>4. Sindikudziwa
Decision Making		
Intro Note	Pa nthawi ino ndikufunsani za momwe mumapangira maganizo ndi bwenzi lanu	
f11	Nthawi zambiri ndi ndani amene amapanga chiganizo cha momwe ndalama mumapeze zigwiritsidwe ntchito?	1. Ine<br>2. Timagwirizana<br>3. Nthawi zambiri bwenzi langa<br>4. Munthu wina<br>5. Sindipeza ndalama<br>88. Sindikufuna kuyankha
f11b	(Ngati yankho ndi 4) Amapanga chiganizo ndi ndani?	________________________
f12	Amapanga chiganizo cha momwe ndalama za bwenzi lanu zigwiritsidwe ntchito ndi ndani?	1. Ine<br>2. Timagwirizana<br>3. Nthawi zambiri bwenzi langa<br>4. Munthu wina<br>5. Sindipeza ndalama

For peer review only

		88. Sindikufuna kuyankha
f12b	(Ngati yankho ndi 4)	______________________
f13	Nthawi zambiri amapanga chiganizo chokhudza thandizo la zaumoyo wanu ndi ndani?	1. Ine<br>2. Timagwirizana<br>3. Nthawi zambiri bwenzi langa<br>4. Munthu wina<br>5. Sindipeza ndalama<br>88. Sindikufuna kuyankha
f13b	(Ngati yankho ndi 4) Amapanga chiganizo ndi ndani?	______________________
f14	Nthawi zambiri amapanga chiganizo cha thandizo la zaumoyo la mwana yemwe muli naye ndi bwenzi lanu ndi ndani?	6. Ine<br>7. Mogwirizana<br>8. Nthawi zambiri bwenzi langa<br>9. Munthu wina<br>10. Ndilibe mwana<br>88. Sindikufuna kuyankha
f14b	(if above question=4) Who decides?	______________________
f15	Nthawi zambiri amapanga chiganizo chokhudza thandizo la zaumoyo la bwenzi lanu ndi ndani?	1. Ine<br>2. Mogwirizana<br>3. Nthawi zambiri bwenzi langa<br>4. Munthu wina<br>5. Ndilibe ana<br>88.Sindikufuna kuyankha
f15b	(Ngati yankho ndi 4) Amapanga chiganizo ndi ndani?	______________________
f16	Kodi amapanga ziganizo zogula katundu mkulumkulu wapakhomo panu ndi ndani?	1. Ine<br>2. Mogwirizana<br>3. Nthawi zambiri bwenzi langa

		4. Munthu wina<br>5. Ndilibe ana<br>88.Sindikufuna kuyankha
f16b	(Ngati yankho ndi 4) Amapanga chiganizo ndani?	
Note	Ndikufuna ndikufunseni zokhudzana za (Kuthekela/mwayi) kuti zinthu zina zichitike. Muli nyemba mu kapu. Ndikufunsani kuti musankhe zina mwa nyemba ndipo muyike mu mbale. Mulingo wa nyemba omwe muyike mu mbale udzafanizira kuti chinachake chichitika. Nyemba imodzi ikusonyeza kuti mwayi ndiochepa kuti chinachake chichitika. Ngati simuika nyemba mm'bale ndekuti mukutsimikiza kuti palibe mwayi oti chinachake chichitika	
note2	Ngati muyike nyemba zowonjezera mu mbale, zikutanthauza kuti mwayi oti chinachake chichitika uchuluka, mwachitsanzo ngati muyika nyemba imodzi kapena ziwiri mwayi oti chinachake chichitika. Ngakhale pali mwayi ochepa koma chinachake chichitika. Ngati muyike nyemba nkhumi zikutanthauza kuti pali mwayi ofanana oti chinachake chichika kapena ayi. Ngati muyike nyemba zisanu ndi imodzi zikutanthauza kuti mwayi woti chinachake chichitika uli ochulukilapo kuposa mwayi oti chinachake sichichitika. Ngati muyike nyemba zonse khumi ndekuti muli ndi chikhulupiliro chonse kuti chinachake chichitikadi pavute pasavute. Palibe yankho lokhonza kapena lolakwa ndikungofuna kudziwa zomwe mukuganiza.	

For peer review only

note3	Ofunsa: Pelekani yankho lanu pa funso lililonse nambala ya nyemba zomwe lili m'bale. Pakutha pa funso lililonse, bwezeletsani nyemba pa tebulo.	
Practice		
pr1	Sankhani mulingo wa nyemba omwe ukhale ndi kuthekela kumene mukuganiza kuti:	________________
pr1b	Mupita ku msika mosachepera kamodzi m'masiku awiri akudzawa	________________
pr1c	Mupita ku msika mosachepera kamodzi m'masabata awiri akudzawa.	________________
Practice		
pr2	Ofunsa: kodi oyankha anawonjezera nyamba pakati pa pr1b ndi pr1c	2. Eya<br>0. Ayi
pr3	Kumbukurani kuti pamene nthawi ikupita muzipeza mpata wambiri opita kunsika. Choncho, munayenela kuti mwaika nyemba zambiri m'bale<br><br>Kodi mukuganiza kuti kumsika mupita mosachepela kamodzi bwanji mu nyengo ya ma sabata awiriwa?	________________
f17	Sankhani mlingo wa nyemba umene ufanizile kaganidwe kanu:	
f17b	Mukhalabe mukanali pa banja/ndi (bwenzi oposela chaka chimodzi kuchoka pano	________________
f17c	Mudzadwala mu miyezi khumi ndi iwiri ikubwelayi	________________
f17d	Mudzayamba kumwa ma ARV mu miyezi itatu ikubwelayi	________________

For peer review only

f17e	Mudzadziwitsa achibale ndi anzanu za zotsatira zanu za HIV m'miyezi itatu ikubwelayi	________________
f17c	Mukusafuna kwanu, anzanu ndi abale azadziwa kuti muli ndi HIV, mu miyezi itatu yapitayi.	________________
SELF REPORTED HEALTH AND HAPPINESS		
Intro Note	Pa nthawi ino ndikufuna ndikambe nanu zokhudza umoyo ndi chisangalalo chanu.	
e1	Ndili ndi chidwi ndi mukudziwa za umoyo ndi kukhutitsidwa kwanu. Kodi muli okhutitsidwa bwanji ndi moyo wanu, pakutengela zonse.	6. Okhutitsidwa kwamburi<br>7. Okhutitsidwa pang'ono<br>8. Pakatikati<br>9. Okhutitsidwa pang'ono<br>10. Osakhutitsidwa olo pang'ono
e2	Kodi mukuona ngati muli okhutitsidwa mofanana kapena osakhutitsidwa pang'ono mosaposela anthu ena a muna kapena akazi a msinkhu wanu ndi opezeka m'mudzi mwanu?	1. Okhutitsidwa kwambiri<br>2. Okhutiotsidwa<br>3. Okhutitsidwa pang'ono
e3	Kutengela zonse, Kodi munganene kuti umoyo wanu tsopano uli bwino kwambiri, ulibwino, sulibwino, suli bwino olo pang'ono	1. Bwino kwambiri<br>2. Bwino<br>3. Silibwino<br>4. SIlibwino olo pang'ono
e4	Kodi mungazifananizile bwanji za thanzi lanu ndi la anthu ena a msikhu ofanana ndi wanu, amuna kapena akazi a m'mudzi mwanu.	1. A thanzi kwambiri<br>2. A thanzi<br>3. Nthanzi lochepekela
e5	Kodi mwezi wathawu, ndi masiku angati omwe munadwara kwambiri ofika kukulepheretsani kugwira ncthito/kupita ku sukulu/kugwira ntchito za pa khomo	________________
Happiness		
e6	Kodi ziganizo izi ndi zowona bwanji kwa inu?	
e6_1	Ndinali ndi nkhawa	1. Ndikugwirizana nazo kwambiri

For peer review only

		2. Ndikugwirizana nazo<br>3. Sindikugwirizana nazo<br>4. Sindikugwirizana nazo kwambiri
e6_2	Ndimanva ngati moyo wafika	1. Ndikugwirizana nazo kwambiri<br>2. Ndikugwirizana nazo<br>3. Sindikugwirizana nazo<br>4. Sindikugwirizana nazo kwambiri
e6_3	Ndimanva kukhutitsidwa	1. Ndikugwirizana nazo kwambiri<br>2. Ndikugwirizana nazo<br>3. Sindikugwirizana nazo<br>4. Sindikugwirizana nazo kwambiri
e6_4	Ndimanva kusalidwa	1. Ndikugwirizana nazo kwambiri<br>2. Ndikugwirizana nazo<br>3. Sindikugwirizana nazo<br>4. Sindikugwirizana nazo kwambiri
GENDER EQUITABLE MEN SCALE		
Note	Chonde ndiuzeni ngati mukugwirizana nazo kwabiri, mukugwirizana nazo, simukugwirizana nazo, simukugwirizana nazo olo pang'ono ziganizo izi:	
j1	Udindo ofunikila wa mzimayi ndi kusamala khomo lake ndi kuphika.	1. Ndikugwirizana nazo kwambiri<br>2. Ndikugwirizana nazo<br>3. Sindikukhulupilira<br>4. Sindikugwirizana nazo<br>5. Sindikugwirizana nazo olo pang'ono<br>88. Sindikufuna kuyankha
j2	Abambo amafuna kugonana kuposa amayi	1. Ndikugwirizana nazo kwambiri<br>2. Ndikugwirizana nazo<br>3. Sindikukhulupilira<br>4. Sindikugwirizana nazo

For peer review only

		5. Sindikugwirizana nazo olo pang'ono<br>88. Sindikufuna kuyankha
j3	Azibambo sakonda kukambirana za kugonana amangochita	1. Ndikugwirizana nazo kwambiri<br>2. Ndikugwirizana nazo<br>3. Sindikukhulupilira<br>4. Sindikugwirizana nazo<br>5. Sindikugwirizana nazo olo pang'ono<br>88. Sindikufuna kuyankha
j4	Nthawi zina mzimayi amayenela kumenyedwa.	1. Ndikugwirizana nazo kwambiri<br>2. Ndikugwirizana nazo<br>3. Sindikukhulupilira<br>4. Sindikugwirizana nazo<br>5. Sindikugwirizana nazo olo pang'ono<br>88. Sindikufuna kuyankha
j5	Kusinta matewela, kusambitsa mwana, kudyetsa mwana ndi udindo wa mzimayi	1. Ndikugwirizana nazo kwambiri<br>2. Ndikugwirizana nazo<br>3. Sindikukhulupilira<br>4. Sindikugwirizana nazo<br>5. Sindikugwirizana nazo olo pang'ono<br>88. Sindikufuna kuyankha
j6	Ndi udindi wa mzimayi kupewa kutenga mimba.	1. Ndikugwirizana nazo kwambiri<br>2. Ndikugwirizana nazo<br>3. Sindikukhulupilira<br>4. Sindikugwirizana nazo<br>5. Sindikugwirizana nazo olo pang'ono<br>88. Sindikufuna kuyankha
j7	Mzibambo ayenela kukhala ndi chiganizo chomaliza cha mnyumba mwake.	1. Ndikugwirizana nazo kwambiri<br>2. Ndikugwirizana nazo<br>3. Sindikukhulupilira<br>4. Sindikugwirizana nazo

For peer review only

		5. Sindikugwirizana nazo olo pang'ono<br><br>88. Sindikufuna kuyankha
j8	Azibambo amakhala okonzeka ku gonana	1. Ndikugwirizana nazo kwambiri<br>2. Ndikugwirizana nazo<br>3. Sindikukhulupilira<br>4. Sindikugwirizana nazo<br>5. Sindikugwirizana nazo olo pang'ono<br><br>Sindikufuna kuyankha
j9	Mzimayi akuyenela kulekelela nkhaza kuti asunge banja lake.	1. Ndikugwirizana nazo kwambiri<br>2. Ndikugwirizana nazo<br>3. Sindikukhulupilira<br>4. Sindikugwirizana nazo<br>5. Sindikugwirizana nazo olo pang'ono<br><br>Sindikufuna kuyankha
j10a	Ndikhoza kukwiya akaza anga atati andiuze kuti ndigwiritse ntchito kondumu.	1. Ndikugwirizana nazo kwambiri<br>2. Ndikugwirizana nazo<br>3. Sindikukhulupilira<br>4. Sindikugwirizana nazo<br>5. Sindikugwirizana nazo olo pang'ono<br><br>88. Sindikufuna kuyankha
j10b	Azibambo akhoza kukwiya akazi awo atawauza kuti agwiritse ntchito kondomu	1. Ndikugwirizana nazo kwambiri<br>2. Ndikugwirizana nazo<br>3. Sindikukhulupilira<br>4. Sindikugwirizana nazo<br>5. Sindikugwirizana nazo olo pang'ono<br><br>88. Sindikufuna kuyankha
j11	Mzimayi komanso nzibambo agwirizane limodzi kuti agwiritse ntchito njira yanji yakulela	1. Ndikugwirizana nazo kwambiri<br>2. Ndikugwirizana nazo<br>3. Sindikukhulupilira<br>4. Sindikugwirizana nazo

For peer review only

		5. Sindikugwirizana nazo olo pang'ono<br><br>88. Sindikufuna kuyankha
j12	Sindingakhale ndi nzanga wopanga zamathanyula	1. Ndikugwirizana nazo kwambiri<br>2. Ndikugwirizana nazo<br>3. Sindikukhulupilira<br>4. Sindikugwirizana nazo<br>5. Sindikugwirizana nazo olo pang'ono<br><br>88. Sindikufuna kuyankha
j13a	Ngati munthu angandinyoze, ndiziteteza pogwiritsa ntchito mphanvu, ngati ndikufunika kutero.	1. Ndikugwirizana nazo kwambiri<br>2. Ndikugwirizana nazo<br>3. Sindikukhulupilira<br>4. Sindikugwirizana nazo<br>5. Sindikugwirizana nazo olo pang'ono<br><br>88. Sindikufuna kuyankha
j13b	Ngati munthu anganyoze mzibambo, aziteteze pogwiritsa ntchito mphanvu ngati akufunika kutelo.	1. Ndikugwirizana nazo kwambiri<br>2. Ndikugwirizana nazo<br>3. Sindikukhulupilira<br>4. Sindikugwirizana nazo<br>5. Sindikugwirizana nazo olo pang'ono<br><br>Sindikufuna kuyankha
j14	Kuti ukhale mzibambo ukufunika kukhala ovuta.	1. Ndikugwirizana nazo kwambiri<br>2. Ndikugwirizana nazo<br>3. Sindikukhulupilira<br>4. Sindikugwirizana nazo<br>5. Sindikugwirizana nazo olo pang'ono<br>Sindikufuna kuyankha<br>Sindikugwirizana nazo kwambiri<br><br>88. Refuse to say<br><br>Sindikufuna kuyankha
j15	Azibambo akuyenela kunva manyazi ngati akukanika kutota	1. Ndikugwirizana nazo kwambiri<br>2. Ndikugwirizana nazo

		3. Sindikukhulupilira<br>4. Sindikugwirizana nazo<br>5. Sindikugwirizana nazo olo pang'ono<br><br>88. Sindikufuna kuyankha
j16	Ngati mzibambo wapeleka mimba kwa mzimayi, mwanayo ndi udindo wa anthu onse a wiri.	1. Ndikugwirizana nazo kwambiri<br>2. Ndikugwirizana nazo<br>3. Sindikukhulupilira<br>4. Sindikugwirizana nazo<br>5. Sindikugwirizana nazo olo pang'ono<br><br>88. Sindikufuna kuyankha
j17	Nzibambo akuyenela kudziwa zomwe bwenzi lake limakonda pogonana	1. Ndikugwirizana nazo kwambiri<br>2. Ndikugwirizana nazo<br>3. Sindikukhulupilira<br>4. Sindikugwirizana nazo<br>5. Sindikugwirizana nazo olo pang'ono<br><br>88. Sindikufuna kuyankha
j18	Kutenga nawo mbali kwa a bambo ndi kofunika polela mwana	1. Ndikugwirizana nazo kwambiri<br>2. Ndikugwirizana nazo<br>3. Sindikukhulupilira<br>4. Sindikugwirizana nazo<br>5. Sindikugwirizana nazo olo pang'ono<br><br>88. Sindikufuna kuyankha
j19	Ndi zofunika kuti abambo azikhala ndi anzawo okambilana nawo mavuto awo.	1. Ndikugwirizana nazo kwambiri<br>2. Ndikugwirizana nazo<br>3. Sindikukhulupilira<br>4. Sindikugwirizana nazo<br>5. Sindikugwirizana nazo olo pang'ono<br><br>88. Sindikufuna kuyankha
j20	Banja lizigwirizana limodzi ngati likufuna kukhala ndi mwana	1. Ndikugwirizana nazo kwambiri<br>2. Ndikugwirizana nazo<br>3. Sindikukhulupilira<br>4. Sindikugwirizana nazo

For peer review only

		5. Sindikugwirizana nazo olo pang'ono<br>88. Sindikufuna kuyankha
STIGMA		
Note	Mugawo lotsatila ndifuna tikambilane zokhudza maganizo anu a anthu omwe ali ndi HIV komanso akukhala mu dela lanu. Chonde khalani omasuka kuyankhula momasuka, palibe yankho lohoza kapena lolakwa. Ndikufuna ndinve maganizo anu.	
i3	Ndikhoza kugula ndiwo zamasamba kwa munthu oti ndikudziwa kuti ali ndi HIV.	1. Ndikugwirizana zano kwambiri<br>2. Ndikugwirizana nazo<br>3. Pakatikati<br>4. Sindikugwurizana nazo<br>5. Sindikugwirizana nazo kwambiri
i4	Ngati wachibale wanga angadwale AIDS, ndingavomele kumusamala pakhomo panga.	1. Ndikugwirizana zano kwambiri<br>2. Ndikugwirizana nazo<br>3. Pakatikati<br>4. Sindikugwurizana nazo<br>5. Sindikugwirizana nazo kwambiri
i5	M'maganizo mwanga, ngati mphunzitsi wa mkazi ali ndi HIV koma sakudwala, aloledwe kupitiliza kuphunzitsa.	1. Ndikugwirizana zano kwambiri<br>2. Ndikugwirizana nazo<br>3. Pakatikati<br>4. Sindikugwurizana nazo<br>5. Sindikugwirizana nazo kwambiri
EXPECTATIONS		

For peer review only

Intro Note	Ndikufuna ndikufunseni zokhudzana za (Kuthekela/mwayi) kuti zinthu zina zichitike. Muli nyemba mu kapu. Ndikufunsani kuti musankhe zina mwa nyemba ndipo muyike mu mbale. Mulingo wa nyemba omwe muyike mu mbale udzafanizira kuti chinachake chichitika. Nyemba imodzi ikusonyeza kuti mwayi ndiochepa kuti chinachake chichitika. Ngati simuika nyemba mm'bale ndekuti mukutsimikiza kuti palibe mwayi oti chinachake chichitika	
h4	Sankhani mulingo wa nyemba omwe ukhale ndi kuthekela kumene mukuganiza kuti:	________________
h4b	Mudzakhala mukudalila apabanja panu pa nkhani ya zachuma mu miyezi itatu ikubwelayi.	________________
h4c	Mukhala mukuthandiza achibale ena pa nkhani za chuma miyezi itatu ikubweyi.	________________
Note	Kotsatira ndikufuna ndikufunseni mafunse ochepa okhudza chiyembekezo chanu cha mtsogolo. Ndikudziwa palibe yemmwe amadziwa za mtsogolo koma tiyeni tikambe mongoyelezeka.	
h5	Mu chaka chamawa chiyembekezero choti mudzakhala mu:	
h5a	Mudzakhala mutayamba sukulukuchoka lelo chaka chamawa	________________
h5b	Kuyamba buzinesi	________________
h5c	Kutsegula akaunti ku banki	________________
h5d	Ku gula malo?	________________

For peer review only

h5e	Kusunga ndalama?	______________________
h5f	Kukhala ndi chakudya chochepa?	______________________
h5g	Kukhala pa ntchito yokhazikika	______________________
Tested for HIV		
pd2	Mwayezetsapo HIV kokwana kangati?<br>*Enter "-99" if client doesn't remember*	______________________
pd3	Komaliza munayezetsa HIV kanali liti?	a) Chaka ______<br>b) Mwezi ________
pd5	Ganizani za ulendo wanu ayamba olandira zotsatira zakuyezadwa kwa HIV. Koyamba kulandira zotsatira zoti akupezani ndi HIV kanali liti?	a) Chaka ______<br>b) Mwezi ________
pd6	Have you ever initiated ART?<br>Munayamba mwamapo ma ARV?	2. Eya<br>0. Ayi
First Initiated ART		
pd6b	Koyamba kumwa ma ARV kanali liti?	c. Chaka ______<br>d. Mwezi ________
pd7	Munayamba mwachedwako kukatenga mwankhwala masiku ochepela khumi ndi folo?	1. Eya<br>0. Ayi
pd7b	Masiku angati?	______________________
Pd7c	Munasiya kumwa ma ARV chifukwa chani?	______________________<br>______________________
pd8	Mukudziwa munthu wina aliyense	

For peer review only

	yenwe akumwa ma ARV?	1. Eya<br>0. Ayi
pd8b	Pano ganizani za munthu yemwe akumwa ma ARV amene mulinali pafupi<br><br>Mumayankhula naye mowilikiza bwanji?	1. Siku ndi tsiku<br>2. Kangapo pa sabata<br>3. Kamodzi pa sabata<br>4. Kangapo pa sabata<br>5. Kamodzi kamwezi<br>6. Kochepera kamodzi pa mwezi<br>7. Sitiyankhulana
pd9	Munayamba mwaululapo za momwe nthupi mwanu mulili za HIV kwa anthu ena kupatura bwenzi lanu?	1. Eya<br>1. Ayi
pd9b	Wina munamuuza anali ndani?<br>*Mark all that apply*	1. Ntcemwali<br>2. Ntchimwene<br>3. Bambo anga<br>4. Mayi anga<br>5. Malume<br>6. Azakhali anga<br>7. Nzanga<br>8. Apongozi akazi<br>9. Apongozi amuna<br>10. Ana anga<br>11. Ogwira naye ntchito<br>12. Bwezi logonanalo<br>13. Zina, fotokozani
pd10	Pa munthu yemwe munamuwuza, ndi ndani yemwe mumayankhula naye kawirikawiri?	1. Ntcemwali<br>2. Ntchimwene<br>3. Bambo anga<br>4. Mayi anga<br>5. Malume<br>6. Azakhali anga<br>7. Nzanga<br>8. Apongozi akazi<br>9. Apongozi amuna<br>10. Ana anga<br>11. Ogwira naye ntchito<br>12. Bwezi logonanalo<br><br>Zina, fotokozani

For peer review only

pd10c	Muntjuyi mumayankhula naye mowirikiza bwanji?	1. Tsiku ndi tsiku<br>2. Kangapo pa sabata<br>3. Kamodzi pa sabata<br>4. Kangapo pa mwezi<br>5. Kamodzi pa mwezi<br>6. Kochepera kamodzi pa mwezi<br>7. Sitiyankhulana
PREVIOUS USE OF HEALTH SERVICES		
Intro Note	Pa ntahwi ino ndifuna tikambilane zokhudza za zomwe mwadutsamo pogwiritsa ntchito thandizo la za umoyo pa chipatala.	
h1	Munayamba mwapitako ku chipatala miyezi khumi ndi iwiri yapitayi.(chifukwa cha inu kapena kupelekeza munthu wina)?	1. Eya<br>0. Ayi
h2	Mwapita ku chipatala kangati miyezi khumi ndi iwiri yapitayi?	______________
h3	Pa nthawi ino ganizani za inu. Mwapita kangati ku chipatala miyezi khumi ndi awiri yapitayi panokha kukalandira thandizo la zaumoyo?	______________
h_a1	Komaliza kupita kuchipatala(chaka) chifukwa mwadwala ndinu kanali liti?<br>NOTE: PUT WHAT YEAR. (i.e., 2015). If DO NOT REMEMBER, help them estimate. IF NEVER GONE, put -99	______________
h4	Munalandira thandizo lanji ulendo umaliza munapita kuchipatala?	12. ANC<br>13. Family Planning<br>14. Delivery<br>15. Post-natal<br>16. Under Five Ku ana<br>17. Kukayezetsa HIV<br>18. ARV<br>19. (OPD) Kudwala<br>20. (OPD)Kuvulala<br>21. Kukonana ndi dotolo wa manu

For peer review only

		22. Palibe
h_a2	Tsopano ganizani za kachiwiri komwe munapita kuchipatala nokha cha posachedwapa. Munapita ku chiptala chaka chanji?	________________________
c3	Chonde ganizani za ulendo wanu munapita kuchipatala chaposachedwa, kuphatikizapo lelo. Munapita lelo?	c. Chaka___________<br>d. Mwezi_________<br>Mwezi
c4	Munapita ku chipatala chiti	1. Chipatala chomwe mumapita pakali pano<br>2. Chipatala china, tchulani
c5	Munapita kukalandila thandizo lanji ku chipatala?	1. ANC<br>2. Family Planning<br>3. Delivery<br>4. Post-natal<br>5. Under Five Ku ana<br>6. Kukayezetsa HIV<br>7. ARV<br>8. (OPD) Kudwala<br>9. (OPD)Kuvulala<br>10. Kukonana ndi dotolo wa manu<br>11. Palibe
c5b	Amene analandira thandizo la zaumoyo anali ndani?	1. Ineyo<br>2. Mwana wanga<br>3. Bwenzi langa<br>4. Wapabanja panga<br>5. Mzanga<br>99. Zina, fotokozani_______
c6a	Inu kapena munthu yemwe munapita naye kuchipatala analandila munthu wina wathandizo la zaumoyo?	2. Eya<br>0. Ayi
c6	Thandizo lachiwiri la zaumyo lomwe manalandira linali lanji?	1. ANC<br>2. Family Planning<br>3. Delivery<br>4. Post-natal<br>5. Under Five Ku ana<br>6. Kukayezetsa HIV<br>7. ARV<br>8. (OPD) Kudwala

For peer review only

		9. (OPD)Kuvulala<br>10. Kukonana ndi dotolo wa manu<br>11. Palibe
c6b	Analandira thandizo la zaumoyo anali ndani?	1. Ine<br>2. Mwana wanga<br>3. Bwezi langa<br>4. Wapabanja panga<br>5. Nzanga Zina, fotokozani
Service Satisfaction		
c10	Pa nthawi ino ndikufuna ndikufunseni za kukhutila kwanu ndi thandizo la zaumoyo munalandila pa tsikulo. Chinde nduuzeni ngai zina mwazotsatirazi zinli vito kwa inu patsiku lomwe munapita kuchipatala, ngati eya, ngati vutolo linali lalikulu kapena ayi.	
c10_1	Nthawi yomwe mumafuna kuonana ndi dotolo	1. Vuto kwambiri<br>2. Vuto pang'ono<br>3. Silinali vuto<br>88. Not applicable<br>98. Sindikudziwa
c10_2	Kuthekela kokambilana za mavuto anu a pakati.	1. Vuto kwambiri<br>2. Vuto pang'ono<br>0. Silinali vuto<br>88. Not applicable<br>98. Sindikudziwa
c10_3	Mulingo waku Kufotokozeledwa munalandira okhudza vuto lanu kapena thandizo.	1. Vuto kwambiri<br>2. Vuto pang'ono<br>0. Silinali vuto<br>88. Not applicable<br>98. Sindikudziwa
c10_4	Chinsinsi kuopetsa ena kuwona zotsatila za umoyo	1. Vuto kwambiri<br>2. Vuto pang'ono<br>0. Silinali vuto<br>88. Not applicable<br>98. Sindikudziwa

For peer review only

c10_5	Chinsinsi kuopa ena kunva za zokambilana zanu	1. Vuto kwambiri<br>2. Vuto pang'ono<br>0. Silinali vuto<br>88. Not applicable<br>98. Sindikudziwa
c10_6	Kupezeka kwa mankwala mzipatala	1. Vuto kwambiri<br>2. Vuto pang'ono<br>0. Silinali vuto<br>88. Not applicable<br>98. Sindikudziwa
c10_7	Ma ola omwe thandizo limapelekedwa pa chipatala i.e thawi yotsegulila ndi yotsekela	1. Vuto kwambiri<br>2. Vuto pang'ono<br>0. Silinali vuto<br>88. Not applicable<br>98. Sindikudziwa
c10_8	Masiku omwe mumalandila thandizo	1. Vuto kwambiri<br>2. Vuto pang'ono<br>0. Silinali vuto<br>88. Not applicable<br>98. Sindikudziwa Sindikudziwa
c10_9	Ukhondo wa pa chipatala	1. Vuto kwambiri<br>2. Vuto pang'ono<br>0. Silinali vuto<br>88. Not applicable<br>98. Sindikudziwa
c10_10	Momwe ogwira ntchito amakusamalilani	1. Vuto kwambiri<br>2. Vuto pang'ono<br>0. Silinali vuto<br>88. Not applicable<br>98. Sindikudziwa
c10_11	Mtengo wa thandizo ndi mankwala	1. Vuto kwambiri<br>2. Vuto pang'ono<br>1. Silinali vuto<br>88. Not applicable<br>98. Sindikudziwa

For peer review only

Satisfaction		
c11	Ndi chiganizo chiti chomwe chikufotokoza za maganizo anu okhudza thandizo lomwe munalandila pa chipatala?	1. Ndili okhutitsidwa kwabiri ndi thandizo ndinalandila.<br>2. Ndili okhutitsidwa ndi thandizo ndinalandila.<br>3. Sindili okhutitsidwa ndi thandizo ndinalandila<br>4. Sindili okhutitsidwa kwambiri ndi thandizo ndinalandila
c12	Munatchulako za chipatalachi kwa nzanu kapena wachibale?	2. Eya<br>0. Ayi
Comment	Tafika pamapeto a kucheza kwathu. Zikomo chifukwa cha nthawi yanu. Pali chili chonse mukufuna kuwonjezera?	
End Note	Thank the participant for their time and give them transport reimbursement, if they did not come for an ART appointment	
Comments	Enumerator comments	
End of the survey!		

For peer review only

14.6. APPENDIX F: Baseline survey – Female

BASELINE SURVEY
Identifying efficient linkage strategies for HIV self-testing (IDEaL)
Female

INTRODUCTION SECTION		
interviewer	Full Name of Interviewer	
Interview date	Interview date	
Time start	Time survey started	
District	District	
Facility	Facility	
ID	ID	

For peer review only

SECTION A: DEMOGRAPHICS		
Intro Note	Thank you for agreeing to participate. Now I will ask you a few questions about yourself and who you are. Please feel free to answer honestly. There are no right or wrong answers.	
a7	What is your tribe?	1. Lomwe<br>2. Sena<br>3. Chewa<br>4. Mang'anja/Nyanja<br>5. Ngoni<br>6. Tumbuka<br>7. Tonga<br>8. Yao<br>99. Other, specify
a3	What is the highest level of school you attended?	1. Primary<br>2. Secondary<br>3. Higher
a3b	What class did you complete in your highest level of school?	________________
a4	Please think of the past 12months, how would you describe your primary occupation?	1. Working formally (employed full time)<br>2. Working informally (ganyu, farming, business)<br>3. Not working
a5	Are you currently married?	1. Married<br>2. Live-in partner<br>3. Steady Boyfriend<br>4. Separated<br>5. Divorced

For peer review only

		99. Other, specify
a6	How many living children do you have?	______________________
a6b	What is the age of your youngest child?	______________________
a6bc	What age is the child (in years or months)	______________________
a4b	How many children currently live with you?	______________________
a7	How many sexual partners have you had in the past 12 months?	______________________
a8	Have you had sex with someone besides your husband without a condom in the past 12 months?	2. Yes<br>0. No<br>88. Don't know/ Not sure<br>89. Refused to answer
a8b	Have you had sex without a condom in the past 12 months?	2. Yes<br>0. No<br>88. Don't know/ Not sure<br>89. Refused to answer
SECTION B: INCOME QUESTIONS		
Intro Note	I will now discuss with you about the valuable items that you or your household possesses. As I will be chatting with you I will also some questions about money you have and activities that you indulge in to find money.	
b1	Please think of the past 12 months, how would you describe your primary occupation?	1. Working formally (employed full time)<br>2. Working informally (ganyu, farming, business)<br>3. Not working

For peer review only - http://bmjopen.bmj.com/site/about/guidelines.xhtml

b1b	Think about all the work you have done in the past month. How many days did you normally work this month that gave you pay?	________________
b2	Do you have any savings for the future, such as a bank account, savings group or cash?	2. Yes<br>0. No
Household Assets		
b3	Does your household have:<br>*The respondent said that his/her household doesn't have any of the household assets. Please probe and ensure that this is correct before you proceed.*	
b3_1	Metal Roof?	2. Yes<br>0. No
b3_2	Electricity?	2. Yes<br>0. No
b3_3	Paraffin lamp with no glass?	2. Yes<br>0. No
b3_4	A paraffin lamp?	2. Yes<br>0. No
b3_5	A radio?	2. Yes<br>0. No
b3_6	A television?	2. Yes<br>0. No
b3_7	A cellular phone?	2. Yes<br>0. No
b3_8	A bed?	2. Yes<br>0. No
b3_9	A sofa set?	2. Yes<br>0. No
b3_10	A table?	2. Yes

For peer review only

		0. No
b3_11	A refrigerator	2. Yes<br>0. No
b3_12	Mattress?	2. Yes<br>0. No
b3_13	Chair(s)?	2. Yes<br>0. No
b3_14	Cattle?	2. Yes<br>0. No
b3_15	Goat?	2. Yes<br>0. No
b3_16	Sheep?	2. Yes<br>0. No
b3_17	Pigs?	2. Yes<br>0. No
b3_18	Donkey?	2. Yes<br>0. No
b3_19	Chickens?	2. Yes<br>0. No
b3_20	Other poultry?	2. Yes<br>0. No
b4	In the past 30 days, have you drank beer?	2. Yes<br>0. No
b4b	How many days in the past 30 days have you drank beer?	___________________
b4c	How much money did you spend on beer the last time you went?	MWK:
b4d	In total, approximately how much money did you spend on beer in the past 30 days?	MWK:
Relationship		

For peer review only

Intro Note	Now I'd like to talk to you about your current sexual relationship	
f8	How long have you been/were you in a sexual relationship with your partner?	Days_______________<br>Months_____________<br>Years________________
f9	Do you have children with your partner? How many children?	________________________
f10	How often do you currently talk to your partner?	1. Everyday<br>2. A couple times a week<br>3. Once a week<br>4. A couple times a month<br>5. Once a month<br>6. Less than once a month<br>7. Not at all (never)
f10b	In a typical month, who earns more money? You, or your partner?	1. Myself<br>2. This partner<br>3. We earn the same amount<br>88. Don't know
Decision Making		
Intro Note	Now I would like to talk to you about how you and your partner make decisions.	
f11	Who usually decides how the money you earn will be used?	1. Yourself (Respondent)<br>2. Jointly (This partner and you together)<br>3. Mainly this partner<br>4. Someone else<br>5. Do not earn money<br>88. Refuse to say
f11b	(if above question=4) Who decides?	________________________
f12	Who usually decides how your partner's earnings will be used?	1. Yourself (Respondent)<br>2. Jointly (This partner and you together)<br>3. Mainly this partner<br>4. Someone else<br>5. Do not earn money<br>88. Refuse to say

For peer review only

f12b	(if above question=4) Who decides?	____________________
f13	Who usually makes decisions about health care for yourself?	1. Yourself (Respondent)<br>2. Jointly (This partner and you together)<br>3. Mainly this partner<br>4. Someone else<br>5. Not applicable/ Don't have children<br>88. Refuse to say
f13b	(if above question=4) Who decides?	____________________
f14	Who usually makes decisions about health care for your child with this partner?	1. Yourself (Respondent)<br>2. Jointly (This partner and you together)<br>3. Mainly this partner<br>4. Someone else<br>5. Not applicable/ Don't have children<br>88. Refuse to say
f14b	(if above question=4) Who decides?	____________________
f15	Who usually makes decisions about health care for your partner?	1. Yourself (Respondent)<br>2. Jointly (This partner and you together)<br>3. Mainly this partner<br>4. Someone else<br>5. Not applicable/ Don't have children<br>88. Refuse to say
f15b	(if above question=4) Who decides?	____________________
f16	Who usually makes decisions about making major household purchases?	1. Yourself (Respondent)<br>2. Jointly (This partner and you together)<br>3. Mainly this partner<br>4. Someone else

For peer review only

		5. Not applicable/ Don't have children<br><br>88. Refuse to say
f16b	(if above question=4) Who decides?	
Note	I would like to ask you questions about [probability/chance/likelihood] that certain things will happen. There are ten beans in this cup. I will ask you to pick some of the beans and put them in the plate. The number of beans that you are going to put in the plate will reflect the probability that something will happen. One bean means there is very little chance that something will happen. If you do not put any bean in the plate it means you are certain that there is no likelihood that something will happen.	
note2	If you put additional beans in the plate it means the chance that something will happen will also increase. For example, if you put one or two beans in the plate, it means there is little chance that something will happen. Even though there is little chance but it can happen. If you put ten beans it means there is equal chance of something happening or not. If you put six beans it means the chance that something will happen is slightly greater than not happening. If you put all ten beans, it means you are certain that whatever the case something will really happen. There is no wrong or right answer I just want to know what you think.	
note3	INTERVIEWER: Report for each question the NUMBER OF BEANS put in the PLATE. After each question, replace the beans on the table (unless otherwise noted).	
Practice		
pr1	Pick the number of beans that reflects how likely you think it is that:	____________________

pr1b	You will go to the market at least once within the next 2 days.	________________
pr1c	You will go to the market at least once within the next 2 weeks.	________________
	Practice	
pr2	INTERVIEWER: Did Respondent add any beans between pr1b and pr1c?	2. Yes<br>0. No
pr3	Remember, as time goes by, you may find more time to go to the market. Therefore, you should have added beans to the plate. Let me ask you again. Now, add beans in the plate so that the number of beans in the plate reflects how likely you think it is that you will go to the market at least once within 2 weeks.<br><br>How likely you think it is that you will go to the market at least once within 2 weeks?	________________
f17	Pick the number of beans that reflects how likely you think:	
f17a	You will still be married/with [partner one year from now.	________________
f17b	Your partner will become sick during the next 12 months	________________
f17c	Your partner will start ART treatment in the next 3 months	________________
f17d	Your partner will disclose your HIV status to your close friends/family in the next 3 months	________________
	SELF REPORTED HEALTH AND HAPPINESS	
Intro Note	Now I'd like to talk to you about how healthy and happy you feel.	

For peer review only

e1	I am interested in your general level of well-being or satisfaction with life. How satisfied are you with your life, all things considered?	1. Very satisfied<br>2. Somewhat satisfied<br>3. Neutral<br>4. Somewhat unsatisfied<br>5. Very unsatisfied
e2	Do you think that you are more, equally or less satisfied than other persons your age and sex living in your village?	1. More satisfied<br>2. Equally satisfied<br>3. Less satisfied
e3	In general, would you say your health now is: very good, good, poor or very poor?	1. Very good<br>2. Good<br>3. Poor<br>4. Very poor
e4	How would you compare your health to other people of the same age and sex in your village?	1. More healthy<br>2. Equally healthy<br>3. Less healthy
e5	In the past month, how many days were you too sick to work/go to school/complete household chores?	________________
Happiness		
e6	How true are the following statements for you in the last month?	
e6_1	I have felt depressed	1. Strongly Agree<br>2. Agree<br>3. Disagree<br>4. Strongly Disagree
e6_2	I have felt life was not worth living	1. Strongly Agree<br>2. Agree<br>3. Disagree<br>4. Strongly Disagree
e6_3	I have felt content.	1. Strongly Agree<br>2. Agree<br>3. Disagree<br>4. Strongly Disagree
e6_4	I have felt lonely	1. Strongly Agree<br>2. Agree<br>3. Disagree<br>4. Strongly Disagree
GENDER EQUITABLE MEN SCALE		

For peer review only

Note	Please tell me if you strongly agree, agree, disagree, or strongly disagree with the following statements:	
j1	Woman's most important role is to take care of her home and cook (take care of home is about housekeeping)	1. Strongly Agree<br>2. Agree<br>3. Unsure<br>4. Disagree<br>5. Strongly Disagree<br>88. Refuse to say
j2	Men need sex more than women	1. Strongly Agree<br>2. Agree<br>3. Unsure<br>4. Disagree<br>5. Strongly Disagree<br>88. Refuse to say
j3	Men don't talk about sex, they just do it.	1. Strongly Agree<br>2. Agree<br>3. Unsure<br>4. Disagree<br>5. Strongly Disagree<br>88. Refuse to say
j4	There are times when a woman deserves to be beaten	1. Strongly Agree<br>2. Agree<br>3. Unsure<br>4. Disagree<br>5. Strongly Disagree<br>88. Refuse to say
j5	Changing diapers, giving kids a bath & feeding kids are mother's responsibility	1. Strongly Agree<br>2. Agree<br>3. Unsure<br>4. Disagree<br>5. Strongly Disagree<br>88. Refuse to say
j6	It is a woman's responsibility to avoid getting pregnant	1. Strongly Agree<br>2. Agree<br>3. Unsure<br>4. Disagree<br>5. Strongly Disagree<br>88. Refuse to say

For peer review only

j7	A man should have the final word about decisions in his home	1. Strongly Agree<br>2. Agree<br>3. Unsure<br>4. Disagree<br>5. Strongly Disagree<br><br>88. Refuse to say
j8	Men are always ready to have sex	1. Strongly Agree<br>2. Agree<br>3. Unsure<br>4. Disagree<br>5. Strongly Disagree<br><br>88. Refuse to say
j9	A woman should tolerate violence in order to keep her family together	1. Strongly Agree<br>2. Agree<br>3. Unsure<br>4. Disagree<br>5. Strongly Disagree<br><br>88. Refuse to say
j10a	I would be outraged if my wife asked me to use a condom.	1. Strongly Agree<br>2. Agree<br>3. Unsure<br>4. Disagree<br>5. Strongly Disagree<br><br>88. Refuse to say
j10b	Men would be outraged if their wife asked them to use a condom	1. Strongly Agree<br>2. Agree<br>3. Unsure<br>4. Disagree<br>5. Strongly Disagree<br><br>88. Refuse to say
j11	A man and a woman should decide together what type of contraceptive to use	1. Strongly Agree<br>2. Agree<br>3. Unsure<br>4. Disagree<br>5. Strongly Disagree<br><br>88. Refuse to say
j12	I would never have a homosexual friend	1. Strongly Agree<br>2. Agree<br>3. Unsure<br>4. Disagree

For peer review only

		5. Strongly Disagree<br>88. Refuse to say
j13a	If someone insults me, I will defend my reputation, with force if I have to.	1. Strongly Agree<br>2. Agree<br>3. Unsure<br>4. Disagree<br>5. Strongly Disagree<br>88. Refuse to say
j13b	If someone insults a man, he should defend his reputation, with force if he has to	1. Strongly Agree<br>2. Agree<br>3. Unsure<br>4. Disagree<br>5. Strongly Disagree<br>88. Refuse to say
j14	To be a man you need to be tough.	1. Strongly Agree<br>2. Agree<br>3. Unsure<br>4. Disagree<br>5. Strongly Disagree<br>88. Refuse to say
j15	Men should be embarrassed if unable to get an erection	1. Strongly Agree<br>2. Agree<br>3. Unsure<br>4. Disagree<br>5. Strongly Disagree<br>88. Refuse to say
j16	If a guy gets a woman pregnant, child is the responsibility of both the man and woman	1. Strongly Agree<br>2. Agree<br>3. Unsure<br>4. Disagree<br>5. Strongly Disagree<br>88. Refuse to say
j17	A man should know what his partner likes during sex	1. Strongly Agree<br>2. Agree<br>3. Unsure<br>4. Disagree<br>5. Strongly Disagree<br>88. Refuse to say

For peer review only

j18	The participation of the father is important in raising children	1. Strongly Agree<br>2. Agree<br>3. Unsure<br>4. Disagree<br>5. Strongly Disagree<br><br>88. Refuse to say
j19	It's important for men to have friends to talk about their problems	1. Strongly Agree<br>2. Agree<br>3. Unsure<br>4. Disagree<br>5. Strongly Disagree<br><br>88. Refuse to say
j20	A couple should decide together if they want to have children.	1. Strongly Agree<br>2. Agree<br>3. Unsure<br>4. Disagree<br>5. Strongly Disagree<br><br>88. Refuse to say
Comment	We have reached the end of the chat. Thank you for your time. Do you have anything else you would like to say?	
End Note	Thank the participant for their time and give them transport reimbursement, if they did not come for an ART appointment	
Comments	Enumerator comments	
End of the survey!		

For peer review only

BASELINE SURVEY
Identifying efficient linkages strategies for HIVST (IDEaL)
Female (Chichewa)

**Question Name**	**Label**	**Responses**
INTRODUCTION SECTION		
interviewer	Full Name of Interviewer	
Interview date	Interview date	
Time start	Time survey started	
District	District	
Facility	Facility	
ID	ID	
SECTION A: DEMOGRAPHICS		
Intro Note	Zikomo povomera kutenga nawo mbali. Pa nthawi ino ndikufunsani ma funso ochepa okhudza za inu komanso kuti ndinu ndani. Chonde khalani omasuka kuyankha moona. Palibe yankho lokhoza komanso lolakwa.	
a7	Ndinu mtundu wanji wa munthu?	1. Lomwe<br>2. Sena<br>3. Chewa<br>4. Mang'anja/Nyanja<br>5. Ngoni<br>6. Tumbuka<br>7. Tonga<br>8. Yao<br>99. Other, specify

For peer review only

a3	Kodi maphunziro anu mudapita nawo patali bwanji?	1. Pulayimale<br>2. Sekondale<br>3. Koleji
a3b	Ndi kalasi liti munamaliza ya maphunziro anu apamwamba?	____________________
a4	Chonde ganizani za masabata khumi ndi awiri apitawa, mungafotokoze bwanji za ntchito yomwe mumagwira?	1. Ntchito yokhazikika<br>2. Ganyu/bisinesi<br>3. Sindikugwira ntchito
a5	Kodi pakadali pano muli pa banja?	1. Pa banja<br>2. Kukhala limodzi ngati banja<br>3. Chibwezi Chokhazikika<br>4. Tinasiyana<br>5. Banja linatha<br>99. Zina
a6	Muli ndi ana angati amoyo?	____________________
a6b	Mwana wanu wang'ono ali ndi zaka zingati?	____________________
a6bc	Mwana wanu wang'ono ali ndi zaka zingati?	____________________
a4b	Pakadali pano ana omwe mumakhala nawo ndi angati?	____________________
a7	Pa miyezi khumi ndi iwiri yapitayi mwakhala ndi abwenzi ogonana nawo angati?	____________________
a8	Mwakhalapo ndi bwenzi logonana kupatula akazi anu?amuna anu osagwiritsa ntchito kondomu mu miyezi khumi ndi awiri yapitayi?	3. Eya<br>4. Ayi<br>88. Sindikudziwa<br>89. Akana kuyankha
a8b	Mwagonanapo ndi munthu osagwiritsa ntchito kondomu mu miyezi khumi ndi awiri yapitayi?	3. Eya<br>4. Ayi<br>88. Sindikudziwa<br>89. Akana kuyankha

For peer review only

SECTION B: INCOME QUESTIONS		
Intro Note	Pa nthawi ino ndikufunsani za zipangizo zomwe inu komanso apabanja panu alinazo. Mkati mwakucheza kwathu ndikufunsaninso za ndalama zomwe mulinazo komanso komanso zomwe mumachita kuti mupeze ndalama.	
b1	Chonde ganizirani za miyezi Khumi ndi iwiri yapitayi, mungafotokoze bwanji za ntchito yomwe mumagwira?	1. Ntchito yokhazikika<br>2. Ganyu/bisinesi<br>3. Sindikugwira ntchito
b1b	Ganizani za ntchito zonse mwagwira mwezi watha. Mwagwira masiku angati olipidwa?	___________________
b2	Muli ndi ndalama zilizonse zomwe mukusungira za mtsogolo monga, ku banki, gulu losugira ndalama kapena ndalama zosunga kunyumba?	2. Eya<br>3. Ayi
Household Assets		
b3	Does your household have:<br>*The respondent said that his/her household doesn't have any of the household assets. Please probe and ensure that this is correct before you proceed.*	
b3_1	Denga la malata?	1. Eya<br>2. Ayi
b3_2	Magetsi ?	3. Eya<br>4. Ayi
b3_3	Koloboyi?	3. Eya<br>4. Ayi
b3_4	Nyali?	3. Eya<br>4. Ayi
b3_5	Wailesi?	3. Eya<br>4. Ayi
b3_6	Kanema?	3. Eya<br>4. Ayi
b3_7	Lamya ya M'manja?	3. Eya<br>4. Ayi
b3_8	Kama?	3. Eya

For peer review only

		4. Ayi
b3_9	Sofa?	3. Eya<br>4. Ayi
b3_10	Tebulo?	3. Eya<br>4. Ayi
b3_11	FIliji?	3. Eya<br>4. Ayi
b3_12	Matilesi?	3. Eya<br>4. Ayi
b3_13	Mipando	3. Eya<br>4. Ayi
b3_14	Ng'ombe?	3. Eya<br>4. Ayi
b3_15	Mbuzi?	3. Eya<br>4. Ayi
b3_16	Nkhosa?	3. Eya<br>4. Ayi
b3_17	Nkhumba?	3. Eya<br>4. Ayi
b3_18	Bulu?	3. Eya<br>4. Ayi
b3_19	Nkhuku?	3. Eya<br>4. Ayi
b3_20	Zoweta zina	3. Eya<br>4. Ayi
b4	Mu masiku makumi atatu apitawa mwamako mowa?	3. Eya<br>4. Ayi
b4b	Pa masiku makumi atatu apitawa mwamwa mowa masiku angati?	______________________
b4c	Mwataya ndalama zingati masiku omaliza omwe munapita ku mowa?	MWK:
b4d	Zonse pamodzi, mwataya ndalama zingati pa mowa mu masiku makumi atatu apitawa?	MWK:
Relationship		
Intro Note	Panthawi ino ndikufunsani za abwezi ogonana nawo?	
f8	Mwakhala pa ubwenzi ogonana ndi bwenzi lanu kwa nthawi yayitali bwanji?	Masiku ___________<br>Miyezi _____________

For peer review only

		Zaka_______________
f9	Muli ndi ana ndi bwenzi lanu logonana nalo? Ana angati?	_____________________
f10	Mumayankhulana mowirikiza bwanji ndi bwenzi lanu pakadali pano?	8. Tsiku ndi tsiku<br>9. Masiku angapo pasabata<br>10. Kamodzi pa sabata<br>11. Kangapo pa mwezi<br>12. Kamodzi pa mwezi<br>13. Kosakwana mwezi<br>14. Sitiyankhulani
f10b	Pa mwezi amalandila ndalama zambiri ndi ndani? Inu kapena bwenzi lanu?	5. Ine<br>6. Bwenzi langa<br>7. Timalandira ndalama zofanana<br>8. Sindikudziwa
	Decision Making	
Intro Note	Pa nthawi ino ndikufunsani za momwe mumapangira maganizo ndi bwenzi lanu	
f11	Nthawi zambiri ndi ndani amene amapanga chiganizo cha momwe ndalama mumapeze zigwiritsidwe ntchito?	6. Ine<br>7. Timagwirizana<br>8. Nthawi zambiri bwenzi langa<br>9. Munthu wina<br>10. Sindipeza ndalama<br>89. Sindikufuna kuyankha
f11b	(Ngati yankho ndi 4) Amapanga chiganizo ndi ndani?	_____________________
f12	Amapanga chiganizo cha momwe ndalama za bwenzi lanu zigwiritsidwe ntchito ndi ndani?	6. Ine<br>7. Timagwirizana<br>8. Nthawi zambiri bwenzi langa<br>9. Munthu wina<br>10. Sindipeza ndalama<br>89. Sindikufuna kuyankha
f12b	(Ngati yankho ndi 4)	_____________________

For peer review only

f13	Nthawi zambiri amapanga chiganizo chokhudza thandizo la zaumoyo wanu ndi ndani?	6. Ine<br>7. Timagwirizana<br>8. Nthawi zambiri bwenzi langa<br>9. Munthu wina<br>10. Sindipeza ndalama<br>89. Sindikufuna kuyankha
f13b	(Ngati yankho ndi 4) Amapanga chiganizo ndi ndani?	________________________
f14	Nthawi zambiri amapanga chiganizo cha thandizo la zaumoyo la mwana yemwe muli naye ndi bwenzi lanu ndi ndani?	6. Ine<br>7. Mogwirizana<br>8. Nthawi zambiri bwenzi langa<br>9. Munthu wina<br>10. Ndilibe mwana<br>88. Sindikufuna kuyankha
f14b	(if above question=4) Who decides?	________________________
f15	Nthawi zambiri amapanga chiganizo chokhudza thandizo la zaumoyo la bwenzi lanu ndi ndani?	1. Ine<br>2. Mogwirizana<br>3. Nthawi zambiri bwenzi langa<br>4. Munthu wina<br>5. Ndilibe ana<br>88.Sindikufuna kuyankha
f15b	(Ngati yankho ndi 4) Amapanga chiganizo ndi ndani?	________________________
f16	Kodi ndi ndani amene amapanga ziganizo zogula katundu mkulumkulu wapa khomo panu?	1. Ine<br>2. Mogwirizana<br>3. Nthawi zambiri bwenzi langa<br>4. Munthu wina<br>5. Ndilibe ana<br>88.Sindikufuna kuyankha
f16b	(Ngati yankho ndi 4) Amapanga	

For peer review only

	chiganizo ndani?	
Note	Ndikufuna ndikufunseni zokhudzana za (Kuthekela/mwayi) kuti zinthu zina zichitike. Muli nyemba mu kapu. Ndikufunsani kuti musankhe zina mwa nyemba ndipo muyike mu mbale. Mulingo wa nyemba omwe muyike mu mbale udzafanizira kuti chinachake chichitika. Nyemba imodzi ikusonyeza kuti mwayi ndiochepa kuti chinachake chichitika. Ngati simuika nyemba mm'bale ndekuti mukutsimikiza kuti palibe mwayi oti chinachake chichitika	
note2	Ngati muyike nyemba zowonjezera mu mbale, zikutanthauza kuti mwayi oti chinachake chichitika uchuluka, mwachitsanzo ngati muyika nyemba imodzi kapena ziwiri mwayi oti chinachake chichitika. Ngakhale pali mwayi ochepa koma chinachake chichitika. Ngati muyike nyemba nkhumi zikutanthauza kuti pali mwayi ofanana oti chinachake chichika kapena ayi. Ngati muyike nyemba zisanu ndi imodzi zikutanthauza kuti mwayi woti chinachake chichitika uli ochulukilapo kuposa mwayi oti chinachake sichichitika. Ngati muyike nyemba zonse khumi ndekuti muli ndi chikhulupiliro chonse kuti chinachake chichitikadi pavute pasavute. Palibe yankho lokhonza kapena lolakwa ndikungofuna kudziwa zomwe mukuganiza.	
note3	Ofunsa: Pelekani yankho lanu pa funso lililonse nambala ya nyemba zomwe lili m'bale. Pakutha pa funso lililonse, bwezeletsani nyemba pa tebulo.	
Practice		

For peer review only

pr1	Sankhani mulingo wa nyemba omwe ukhale ndi kuthekela kumene mukuganiza kuti:	________________
pr1b	Mupita ku msika mosachepera kamodzi m'masiku awiri akudzawa	________________
pr1c	Mupita ku msika mosachepera kamodzi m'masabata awiri akudzawa.	________________
Practice		
pr2	Ofunsa: kodi oyankha anawonjezera nyamba pakati pa pr1b ndi pr1c	3. Eya<br>1. Ayi
pr3	Kumbukurani kuti pamene nthawi ikupita muzipeza mpata wambiri opita kunsika. Choncho, munayenela kuti mwaika nyemba zambiri m'bale<br><br>Kodi mukuganiza kuti kumsika mupita mosachepela kamodzi bwanji mu nyengo ya ma sabata awiriwa?	________________
f17	Sankhani mlingo wa nyemba umene ufanizile kaganidwe kanu:	

For peer review only

f17a	Mudzakhalabe pabanja ndi bwenzi lanu m'chaka chimodzi chikubwerachi	__________________
f17b	Bwenzi lanu lidzapezeka ndi matenda a HIV m'miyezi khumi ndi iwiri ikubwerayi	__________________
f17c	Bwenzi lanu lidzayamba kumwa ma ARV m'miyezi itatu ikubwerayi	__________________
f17d	Bwenzi lanu lidzaulula kuti inuyo muli ndi kachilombo ka HIV kwa anzanu apamtima/abale anu m'miyezi itatu ikubwerayi	__________________
SELF REPORTED HEALTH AND HAPPINESS		
Intro Note	Pa nthawi ino ndikufuna ndikambe nanu zokhudza umoyo ndi chisangalalo chanu.	
e1	Ndili ndi chidwi ndi mukudziwa za umoyo ndi kukhutitsidwa kwanu. Kodi muli okhutitsidwa bwanji ndi moyo wanu, pakutengela zonse.	6. Okhutitsidwa kwamburi<br>7. Okhutitsidwa pang'ono<br>8. Pakatikati<br>9. Okhutitsidwa pang'ono<br>10. Osakhutitsidwa olo pang'ono
e2	Kodi mukuona ngati muli okhutitsidwa mofanana kapena osakhutitsidwa pang'ono mosaposela anthu ena a muna kapena akazi a msinkhu wanu ndi opezeka m'mudzi mwanu?	4. Okhutitsidwa kwambiri<br>5. Okhutiotsidwa<br>6. Okhutitsidwa pang'ono
e3	Kutengela zonse, Kodi munganene kuti umoyo wanu tsopano uli bwino kwambiri, ulibwino, sulibwino, suli bwino olo pang'ono	5. Bwino kwambiri<br>6. Bwino<br>7. Silibwino<br>8. SIlibwino olo pang'ono
e4	Kodi mungazifananizile bwanji za thanzi lanu ndi la anthu ena a msikhu ofanana ndi wanu, amuna kapena	4. A thanzi kwambiri<br>5. A thanzi<br>6. Nthanzi lochepekela

	akazi a m'mudzi mwanu.	
e5	Kodi mwezi wathawu, ndi masiku angati omwe munadwara kwambiri ofika kukulepheretsani kugwira ncthito/kupita ku sukulu/kugwira ntchito za pa khomo	____________
Happiness		
e6	Kodi ziganizo izi ndi zowona bwanji kwa inu?	
e6_1	Ndinali ndi nkhawa	5. Ndikugwirizana nazo kwambiri<br>6. Ndikugwirizana nazo<br>7. Sindikugwirizana nazo<br>8. Sindikugwirizana nazo kwambiri
e6_2	Ndimanva ngati moyo wafika	5. Ndikugwirizana nazo kwambiri<br>6. Ndikugwirizana nazo<br>7. Sindikugwirizana nazo<br>8. Sindikugwirizana nazo kwambiri
e6_3	Ndimanva kukhutitsidwa	5. Ndikugwirizana nazo kwambiri<br>6. Ndikugwirizana nazo<br>7. Sindikugwirizana nazo<br>8. Sindikugwirizana nazo kwambiri
e6_4	Ndimanva kusalidwa	5. Ndikugwirizana nazo kwambiri<br>6. Ndikugwirizana nazo<br>7. Sindikugwirizana nazo<br>8. Sindikugwirizana nazo kwambiri
GENDER EQUITABLE MEN SCALE		
Note	Chonde ndiuzeni ngati mukugwirizana nazo kwabiri, mukugwirizana nazo, simukugwirizana nazo, simukugwirizana nazo olo pang'ono ziganizo izi:	

For peer review only

j1	Udindo ofunikila wa mzimayi ndi kusamala khomo lake ndi kuphika.	6. Ndikugwirizana nazo kwambiri<br>7. Ndikugwirizana nazo<br>8. Sindikukhulupilira<br>9. Sindikugwirizana nazo<br>10. Sindikugwirizana nazo olo pang'ono<br>89. Sindikufuna kuyankha
j2	Abambo amafuna kugonana kuposa amayi	6. Ndikugwirizana nazo kwambiri<br>7. Ndikugwirizana nazo<br>8. Sindikukhulupilira<br>9. Sindikugwirizana nazo<br>10. Sindikugwirizana nazo olo pang'ono<br>88. Sindikufuna kuyankha
j3	Azibambo sakonda kukambirana za kugonana amangochita	6. Ndikugwirizana nazo kwambiri<br>7. Ndikugwirizana nazo<br>8. Sindikukhulupilira<br>9. Sindikugwirizana nazo<br>10. Sindikugwirizana nazo olo pang'ono<br>88. Sindikufuna kuyankha
j4	Nthawi zina mzimayi amayenela kumenyedwa.	6. Ndikugwirizana nazo kwambiri<br>7. Ndikugwirizana nazo<br>8. Sindikukhulupilira<br>9. Sindikugwirizana nazo<br>10. Sindikugwirizana nazo olo pang'ono<br>88. Sindikufuna kuyankha
j5	Kusinta matewela, kusambitsa mwana, kudyetsa mwana ndi udindo wa mzimayi	6. Ndikugwirizana nazo kwambiri<br>7. Ndikugwirizana nazo<br>8. Sindikukhulupilira<br>9. Sindikugwirizana nazo<br>10. Sindikugwirizana nazo olo pang'ono<br>88. Sindikufuna kuyankha
j6	Ndi udindi wa mzimayi kupewa	6. Ndikugwirizana nazo kwambiri

For peer review only

	kutenga mimba.	7. Ndikugwirizana nazo<br>8. Sindikukhulupilira<br>9. Sindikugwirizana nazo<br>10. Sindikugwirizana nazo olo pang'ono<br>88. Sindikufuna kuyankha
j7	Mzibambo ayenela kukhala ndi chiganizo chomaliza cha mnyumba mwake.	6. Ndikugwirizana nazo kwambiri<br>7. Ndikugwirizana nazo<br>8. Sindikukhulupilira<br>9. Sindikugwirizana nazo<br>10. Sindikugwirizana nazo olo pang'ono<br>88. Sindikufuna kuyankha
j8	Azibambo amakhala okonzeka ku gonana	6. Ndikugwirizana nazo kwambiri<br>7. Ndikugwirizana nazo<br>8. Sindikukhulupilira<br>9. Sindikugwirizana nazo<br>10. Sindikugwirizana nazo olo pang'ono<br>Sindikufuna kuyankha
j9	Mzimayi akuyenela kulekelela nkhaza kuti asunge banja lake.	6. Ndikugwirizana nazo kwambiri<br>7. Ndikugwirizana nazo<br>8. Sindikukhulupilira<br>9. Sindikugwirizana nazo<br>10. Sindikugwirizana nazo olo pang'ono<br>Sindikufuna kuyankha
j10a	Ndikhoza kukwiya akaza anga atati andiuze kuti ndigwiritse ntchito kondumu.	6. Ndikugwirizana nazo kwambiri<br>7. Ndikugwirizana nazo<br>8. Sindikukhulupilira<br>9. Sindikugwirizana nazo<br>10. Sindikugwirizana nazo olo pang'ono<br>88. Sindikufuna kuyankha
j10b	Azibambo akhoza kukwiya akazi awo atawauza kuti agwiritse ntchito	6. Ndikugwirizana nazo kwambiri

For peer review only

	kondomu	7. Ndikugwirizana nazo<br>8. Sindikukhulupilira<br>9. Sindikugwirizana nazo<br>10. Sindikugwirizana nazo olo pang'ono<br>88. Sindikufuna kuyankha
j11	Mzimayi komanso nzibambo agwirizane limodzi kuti agwiritse ntchito njira yanji yakulela	6. Ndikugwirizana nazo kwambiri<br>7. Ndikugwirizana nazo<br>8. Sindikukhulupilira<br>9. Sindikugwirizana nazo<br>10. Sindikugwirizana nazo olo pang'ono<br>88. Sindikufuna kuyankha
j12	Sindingakhale ndi nzanga wopanga zamathanyula	6. Ndikugwirizana nazo kwambiri<br>7. Ndikugwirizana nazo<br>8. Sindikukhulupilira<br>9. Sindikugwirizana nazo<br>10. Sindikugwirizana nazo olo pang'ono<br>88. Sindikufuna kuyankha
j13a	Ngati munthu angandinyoze, ndiziteteza pogwiritsa ntchito mphanvu, ngati ndikufunika kutero.	6. Ndikugwirizana nazo kwambiri<br>7. Ndikugwirizana nazo<br>8. Sindikukhulupilira<br>9. Sindikugwirizana nazo<br>10. Sindikugwirizana nazo olo pang'ono<br>88. Sindikufuna kuyankha
j13b	Ngati munthu anganyoze mzibambo, aziteteze pogwiritsa ntchito mphanvu ngati akufunika kutelo.	6. Ndikugwirizana nazo kwambiri<br>7. Ndikugwirizana nazo<br>8. Sindikukhulupilira<br>9. Sindikugwirizana nazo<br>10. Sindikugwirizana nazo olo pang'ono<br>Sindikufuna kuyankha
j14	Kuti ukhale mzibambo ukufunika kukhala ovuta.	6. Ndikugwirizana nazo kwambiri<br>7. Ndikugwirizana nazo<br>8. Sindikukhulupilira

		9. Sindikugwirizana nazo<br>10. Sindikugwirizana nazo olo pang'ono<br>Sindikufuna kuyankha<br>Sindikugwirizana nazo kwambiri<br>88. Refuse to say<br>Sindikufuna kuyankha
j15	Azibambo akuyenela kunva manyazi ngati akukanika kutota	6. Ndikugwirizana nazo kwambiri<br>7. Ndikugwirizana nazo<br>8. Sindikukhulupilira<br>9. Sindikugwirizana nazo<br>10. Sindikugwirizana nazo olo pang'ono<br>88. Sindikufuna kuyankha
j16	Ngati mzibambo wapeleka mimba kwa mzimayi, mwanayo ndi udindo wa anthu onse a wiri.	6. Ndikugwirizana nazo kwambiri<br>7. Ndikugwirizana nazo<br>8. Sindikukhulupilira<br>9. Sindikugwirizana nazo<br>10. Sindikugwirizana nazo olo pang'ono<br>88. Sindikufuna kuyankha
j17	Nzibambo akuyenela kudziwa zomwe bwenzi lake limakonda pogonana	6. Ndikugwirizana nazo kwambiri<br>7. Ndikugwirizana nazo<br>8. Sindikukhulupilira<br>9. Sindikugwirizana nazo<br>10. Sindikugwirizana nazo olo pang'ono<br>88. Sindikufuna kuyankha
j18	Kutenga nawo mbali kwa a bambo ndi kofunika polela mwana	6. Ndikugwirizana nazo kwambiri<br>7. Ndikugwirizana nazo<br>8. Sindikukhulupilira<br>9. Sindikugwirizana nazo<br>10. Sindikugwirizana nazo olo pang'ono<br>88. Sindikufuna kuyankha

For peer review only

j19	Ndi zofunika kuti abambo azikhala ndi anzawo okambilana nawo mavuto awo.	6. Ndikugwirizana nazo kwambiri<br>7. Ndikugwirizana nazo<br>8. Sindikukhulupilira<br>9. Sindikugwirizana nazo<br>10. Sindikugwirizana nazo olo pang'ono<br>88. Sindikufuna kuyankha
j20	Banja lizigwirizana limodzi ngati likufuna kukhala ndi mwana	6. Ndikugwirizana nazo kwambiri<br>7. Ndikugwirizana nazo<br>8. Sindikukhulupilira<br>9. Sindikugwirizana nazo<br>10. Sindikugwirizana nazo olo pang'ono<br>88. Sindikufuna kuyankha
Comment	Tafika pamapeto a kucheza kwathu. Zikomo chifukwa cha nthawi yanu. Pali chili chonse mukufuna kuwonjezera?	
End Note	Thank the participant for their time and give them transport reimbursement, if they did not come for an ART appointment	
Comments	Enumerator comments	
End of the survey!		

For peer review only

14.7. APPENDIX G: Follow-up Survey - Male

FOLLOW-UP SURVEY
Identifying efficient linkages strategies for HIVST (IDEaL)
Male

Complete this form for men who enrolled in the study 4-months ago

Date of Interview: ____________________
Site Code: __________________________

Full Name of Interviewer: ______________________________

Participant Study ID#: ___________

#	Question	Response
c1a	Please think about your primary partner in the last 4 months.<br><br>Has your relationship changed in the last 4 months? How?	☐ Nothing changed (1)<br>☐ Married (2)<br>☐ Steady Girlfriend (3)<br>☐ Moved out of the house (4)<br>☐ Became an infrequent partner (5)<br>☐ Separated (6)<br>☐ Divorced (7)<br>☐ Other (8)
c4a	Have you disclosed your HIV status to this partner?	☐ Yes (1)<br>☐ No (0)
c5a	Have you had any new children with this partner since we last spoke?	☐ Yes (1)<br>☐ No (0)
c18a	Have you started ART in the past 4months?	☐ Yes (1)<br>☐ No (0)<br>☐ No, but I plan to link

For peer review only

c18b	IF YES: When did you start ART?	________ Day/Month/Year
	**Unintended Consequences**	
c19a	Were unwantedly pressured to initiate ART?	☐ Yes (1)<br>☐ No (0)
c20a	After enrolling in the study, did anyone find out your HIV status against your will (unwanted disclosure)?	☐ Yes (1)<br>☐ No (0)
C20b	After enrolling in the study, did anyone find out your partners' HIV status against her will (unwanted disclosure)?	☐ Yes (1)<br>☐ No (0)
c21a	After enrolling in the study, did your partner….<br><br>Threaten to hurt or harm you or someone you cared about.	☐ Yes (1)<br>☐ No (0)<br>☐ Refused to respond (99)
c22a	Insulted you or made you feel bad about yourself.	☐ Yes (1)<br>☐ No (0)<br>☐ Refused to respond (99)
c23a	Hit, slapped, kicked or did anything else meant to physically hurt you.	☐ Yes (1)<br>☐ No (0)<br>☐ Refused to respond (99)
c21a	After enrolling in the study, did you ever do the following to your partner….<br><br>Threaten to hurt or harm her or someone she cared about.	☐ Yes (1)<br>☐ No (0)<br>☐ Refused to respond (99)

For peer review only

c22a	Insulted her or made her feel bad about herself.	☐ Yes (1)<br>☐ No (0)<br>☐ Refused to respond (99)
c23a	Hit, slapped, kicked or did anything else meant to physically hurt her.	☐ Yes (1)<br>☐ No (0)<br>☐ Refused to respond (99)
c24	Slept with another woman	☐ Yes (1)<br>☐ No (0)<br>☐ Refused to respond (99)
c25a	*Now, I am going to ask you a series of questions about who makes within this relationship. Please think about the last 4 months*<br><br>Who usually decides how the money you earn will be used?	☐ Yourself (respondent)<br>☐ Jointly (This partner and you together)<br>☐ Mainly this partner<br>☐ Someone else<br>☐ Do not earn money<br>☐ Refuse to say
c26a	Who usually decides how your partner's earnings will be used?	☐ Yourself (respondent)<br>☐ Jointly (This partner and you together)<br>☐ Mainly this partner<br>☐ Someone else<br>☐ Refuse to say

For peer review only

c27a	Who usually makes decisions about health care for yourself?	☐ Yourself (respondent)<br>☐ Jointly (This partner and you together)<br>☐ Mainly this partner<br>☐ Someone else<br>☐ Refuse to say

	**D. Additional Questions**	
#	**Question**	**Response**
d1	Would you recommend the ART intervention you were part of to other male friends or family?	☐ Yes (1)<br>☐ No (0)
d2	Are you happy that you participated in the ART intervention?	☐ Yes (1)<br>☐ No (0)

Thank the participant for their time and end the survey

For peer review only

BASELINE SURVEY
Identifying efficient linkages strategies for HIVST (IDEaL)
Male (Chichewa)

Complete this form for men who enrolled in the study 4-months ago

Date of Interview: ____________________
Site Code: _________________________
Full Name of Interviewer: ____________________________
Participant Study ID#: ___________

For peer review only

#	Question	Response
c1a	Taganizani za bwenzi lanu logonana nalo lomwe lakhala lodalilika kwa miyesi folo yapitayi.<br><br>Kodi ubwenzi wanu wasintha munjira ina iliyonse mu miyezi folo yapitayi?	☐ Palibe chasintha (1)<br>☐ Married (2)<br>☐ Chibwenzi chokhazikika (3)<br>☐ Chibwenzi Chinachoka pakhomo (4)<br>☐ Chibwenzi cha apo ndi apo (5)<br>☐ Separated (6)<br>☐ Banja linatha (7)<br>☐ Other (8)
c4a	Kodi munawauza abwenzi anuwa za mmene mulili mnthupi mwanu ku mbali ya HIV?	☐ Eya(1)<br>☐ Ayi(0)
c5a	Kodi mwakhala ndi ana ndi abwenzi anuwa kuchokera ulendo watha tinayankhulana?	☐ Eya(1)<br>☐ Ayi(0)
c18a	Kodi munayamba kumwa mankhwala a ma ARV mu miyezi folo yapitayi?	☐ Eya(1)<br>☐ Ayi(2)<br>☐Ayi, ndikulingalira zoyamba (3)

c18b	Ngati eya: Munayamba liti mankwala a ma ARV?	________<br>Tsiku/Mwezi/Chaka
**Unintended Consequences**		
c19a	Munayamba kumwa mankhwala a ma ARV mokakamizidwa?	☐ Eya(1)<br>☐ Ayi(0)
c20a	Chilowereni mu study, alipo omwe anadziwa za m'mene mulili mnthupi mwanu kumbari ya HIV inu musakufuna? (Kuwulula za HIV mosafuna)	☐ Eya(1)<br>☐ Ayi(0)
C20b	Chilowereni mu study, alipo omwe anadziwa za m'mene mulili mnthupi mwa bwenzi lanu kumbari ya HIV eni asakufuna? (Kuwulula za HIV mosafuna)	☐ Eya(1)<br>☐ Ayi(0)
c21a	Chilowereni mu study, kodi bwenzi lanu…..<br><br>Linaospyeza kuvulaza inu kapena wina aliyense amane mumamukonda?	☐ Eya(1)<br>☐ Ayi(0)<br>☐ Refused to respond (99)
c22a	Linakunyozani kapena kukunyogodolani	☐ Eya(1)<br>☐ Ayi(0)<br>☐ Refused to respond (99)
c23a	Anakumenyani ndikukupwetekani.	☐ Eya(1)<br>☐ Ayi(0)<br>☐ Refused to respond (99)
c21a	Chilowereni mu study kodi munayamba mwapangapo zotsatirazi kwa bwenzi anu….<br><br>Kuosyeza kuti muvulaza bwezi lanu kapena wina aliyense yemwe amamukonda.	☐ Eya(1)<br>☐ Ayi(0)<br>☐ Refused to respond (99)

For peer review only

c22a	Kunyoza kapenanso kumupangitsa kuti azizikayikila.	☐ Eya(1)<br>☐ Ayi(0)<br>☐ Refused to respond (99)
c23a	Kumumenya ndikumupweteka.	☐ Eya(1)<br>☐ Ayi(0)<br>☐ Refused to respond (99)
c24	Kuchita mchitidwe ogonana ndi mzimayi wina	☐ Eya(1)<br>☐ Ayi(0)<br>☐ Refused to respond (99)
c25a	Tsopano ndikufunsani mafunso okhuzana ndi omwe amalamula pakhomo panu.<br><br>Kodi amalamula za mmene ndalama zomwe mwapeza zitagwilitsidwile ntchito ndi ndani?	☐ Amene akuyankha<br>☐ Mogwirizana<br>☐ Nthawi zambiri bwenzi<br>☐ Munthu wina<br>☐ Sapeza ndalama<br>☐ Refuse to say
c26a	Kodi amalamulila za mmene ndalama za abwenzi anu zingagwilitsidwile ntchito ndi ndani?	☐ Amene akuyankha<br>☐ Mogwirizana<br>☐ Nthawi zambiri bwenzi<br>☐ Munthu wina<br>☐ Refuse to say
c27a	Kodi amene amakhala ndi ulamulilo pa chisamalilo cha moyo wanu ndi ndani?	☐ Amene akuyankha<br>☐ Mogwirizana<br>☐ Nthawi zambiri bwenzi<br>☐ Munthu wina<br>☐ Refuse to say

For peer review only

	Additional Questions	
d1	Kodi mungalimbikitse anzanu ena achizibambo kapena apabanja panu kutenga nawo mbali mu ART intervention munaliyi?	☐ Eya(1)<br>☐ Ayi(0)
d2	Kodi muli okondwa kuti munatenga nawo mbali mu ART intervention?	☐ Eya(1)<br>☐ Ayi(0)

For peer review only

14.8. APPENDIX H: Follow-up Survey – Female

FOLLOW-UP SURVEY
Identifying efficient linkages strategies for HIVST (IDEaL)
Female

Complete this form for women whose:

(1) Partners consented to be in the study 4-months ago

Date of Interview: ____________________
Site Code: _________________________

Full Name of Interviewer: ______________________________

Participant Study ID#: ___________

#	Question	Response
c1a	Please think about your primary partner in the last 4 months.<br><br>Has your relationship changed in the last 4 months? How?	☐ Nothing changed (1)<br>☐ Married (2)<br>☐ Steady Boyfriend (3)<br>☐ Moved out of the house (4)<br>☐ Became an infrequent partner (5)<br>☐ Separated (6)<br>☐ Divorced (7)<br>☐ Other (8)
c4a	Have you disclosed your HIV status to this partner?	☐ Yes (1)<br>☐ No (0)
c5a	Have you had any new children with this partner since we last spoke?	☐ Yes (1)<br>☐ No (0)
c18a	To your knowledge, did your partner start ART?	☐ Yes (1)<br>☐ No, they do not plan to link (2)<br>☐ No, but they plan to link (3)

For peer review only

		☐ Unsure (88)
**Unintended Consequences**		
c19a	Did you pressure your partner to initiate ART?	☐ Yes (1)<br>☐ No (0)
c20a	After enrolling in the study, did anyone find out your HIV status against your will (unwanted disclosure)?	☐ Yes (1)<br>☐ No (0)
C20b	After enrolling in the study, did anyone find out your partners' HIV status against his will (unwanted disclosure)?	☐ Yes (1)<br>☐ No (0)
c21a	After enrolling in the study, did your partner….<br><br>Threaten to hurt or harm you or someone you cared about.	☐ Yes (1)<br>☐ No (0)<br>☐ Refused to respond (99)
c22a	Insulted you or made you feel bad about yourself.	☐ Yes (1)<br>☐ No (0)<br>☐ Refused to respond (99)
c23a	Hit, slapped, kicked or did anything else meant to physically hurt you.	☐ Yes (1)<br>☐ No (0)<br>☐ Refused to respond (99)
C23b	Forced sexual intercourse and other forms of sexual coercion	☐ Yes (1)<br>☐ No (0)<br>☐ Refused to respond (99)

For peer review only

C23c	Slept with another woman.	☐ Yes (1)<br>☐ No (0)<br>☐ Refused to respond (99)
c24a	Ended the relationship	☐ Yes (1)<br>☐ No (0)<br>☐ Refused to respond (99)
c25a	*Now, I am going to ask you a series of questions about who makes within this relationship*<br><br>Who usually decides how the money you earn will be used?	☐ Yourself (respondent)<br>☐ Jointly (This partner and you together)<br>☐ Mainly this partner<br>☐ Someone else<br>☐ Do not earn money<br>☐ Refuse to say
c26a	Who usually decides how your partner's earnings will be used?	☐ Yourself (respondent)<br>☐ Jointly (This partner and you together)<br>☐ Mainly this partner<br>☐ Someone else<br>☐ Refuse to say
c27a	Who usually makes decisions about health care for yourself?	☐ Yourself (respondent)<br>☐ Jointly (This partner and you together)<br>☐ Mainly this partner<br>☐ Someone else<br>☐ Refuse to say

For peer review only

	Additional Questions	
d1	Would you recommend the ART intervention to other male friends or family?	☐ Yes (1)<br>☐ No (0)
d2	Are you happy your partner was in the ART intervention?	☐ Yes (1)<br>☐ No (0)

Thank the participant for their time and end the survey

For peer review only

FOLLOW-UP SURVEY
Identifying efficient linkages strategies for HIVST (IDEaL)
Female (Chichewa)

Complete this form for women whose:

(1) Partners consented to be in the study 4-months ago

Date of Interview: ______________________
Site Code: ____________________________

Full Name of Interviewer: _________________________________

Participant Study ID#: ____________

#	Question	Response
c1a	Taganizani za bwenzi lanu logonana nalo lomwe lakhala lodalilika kwa miyesi folo yapitayi.<br><br>Kodi ubwenzi wanu wasintha munjira ina iliyonse mu miyezi folo yapitayi?<br><br>Bwenzi lokhazikika	☐ Palibe chasintha (1)<br>☐ Married (2)<br>☐ Bwenzi lokhazikika (3)<br>☐ Chibwenzi Chinachoka pakhomo (4)<br>☐ Chibwenzi cha apo ndi apo (5)<br>☐ Separated (6)<br>☐ Banja linatha (7)<br>☐ Other (8)
c4a	Kodi munawauza abwenzi anuwa za mmene mulili mnthupi mwanu ku mbali ya HIV?	☐ Eya(1)<br>☐ Ayi(0)
c5a	Have you had any new children with this partner since we last spoke?<br>Kodi mwakhala ndi ana ndi abwenzi anuwa kuchokera ulendo watha tinayankhulana?	☐ Eya(1)<br>☐ Ayi(0)
c18a	Mongamukudziwira, kodi bwenzi lanu linayamba kumwa mwankhala a ama ARV?	☐ Eya<br>☐ Ayi, sakulingalira zoyamba kumwa mwankhwala a ma ARV<br>☐ Ayi, koma

		akulingalira zoyamba<br>☐ Unsure (88)
**Unintended Consequences**		
c19a	Kodi munawakakamiza a bwenzi anu kuti ayambe kumwa mankhwala a ama ARV?	☐ Eya(1)<br>☐ Ayi(0)
c20a	Chilowereni mu study, alipo omwe anadziwa za m'mene mulili mnthupi mwanu kumbari ya HIV inu musakufuna? (Kuwulula za HIV mosafuna)	☐ Eya(1)<br>☐ Ayi(0)
C20b	Chilowereni mu study, alipo omwe anadziwa za m'mene mulili mnthupi mwa bwenzi lanu kumbari ya HIV eni asakufuna? (Kuwulula za HIV mosafuna)	☐ Eya(1)<br>☐ Ayi(0)
c21a	Chilowereni mu study, kodi bwenzi lanu…..<br><br>Linaospyeza kuvulaza inu kapena wina aliyense amane mumamukonda?	☐ Eya(1)<br>☐ Ayi(0)<br>☐ Refused to respond (99)
c22a	Anakunyozani kapenanso kukupangitsani kuti muzizikayikila.	☐ Eya(1)<br>☐ Ayi(0)<br>☐ Refused to respond (99)
c23a	Anakumenyani ndikukupwetekani.	☐ Eya(1)<br>☐ Ayi(0)<br>☐ Refused to respond (99)
C23b	Kukukakamizani kugonana ndi zinthu zina?	☐ Eya(1)<br>☐ Ayi(0)<br>☐ Refused to respond (99)
C23c	Kuchita mtchitidwe ogonana ndi mzimayi wina	☐ Eya(1)<br>☐ Ayi(0)

		☐ Refused to respond (99)
c24a	Kuthetsa Chibwenzi	☐ Eya(1)<br>☐ Ayi(0)<br>☐ Refused to respond (99)
c25a	Tsopano ndikufunsani mafunso okhuzana ndi omwe amalamula pakhomo panu.<br><br>Kodi amalamula za mmene ndalama zomwe mwapeza zitagwilitsidwile ntchito ndi ndani?	☐ Amene akuyankha<br>☐ Mogwirizana<br>☐ Nthawi zambiri bwenzi<br>☐ Munthu wina<br>☐ Refuse to say
c26a	Kodi amalamulila za mmene ndalama za abwenzi anu zingagwilitsidwile ntchito ndi ndani?	☐ Amene akuyankha<br>☐ Mogwirizana<br>☐ Nthawi zambiri bwenzi<br>☐ Munthu wina<br>☐ Refuse to say
c27a	Kodi amene amakhala ndi ulamulilo pa chisamalilo cha moyo wanu ndi ndani?	☐ Amene akuyankha<br>☐ Mogwirizana<br>☐ Nthawi zambiri bwenzi<br>☐ Munthu wina<br>☐ Refuse to say

	**Additional Questions**	
d1	Kodi mungalimbikitse anzanu ena achizibambo kapena apabanja panu kutenga nawo mbali mu ART intervention munaliyi?	☐ Eya(1)<br>☐ Ayi(0)
d2	Kodi muli okondwa kuti munatenga nawo mbali mu ART intervention?	☐ Eya(1)<br>☐ Ayi(0)

For peer review only

14.9. APPENDIX I: Data Extraction Tool

DATA EXTRACTION TOOL
Identifying efficient linkages strategies for HIVST (IDEaL)
English only

INSTRUCTIONS:

The Medical Chart Review will be used to link the male study participant with the facilities ART records and to document their facility visits over the 4-months of study participation. Please follow the instructions to prepare for data collection (1) gather all ART registers that were active between DAY MONTH YEAR up to today (2) enter and re-enter the participant ID's who have reached the 4-month follow up period into the tablet (3) once you have re-entered, the tablet will provide you with identifying information about the male study participant. (5) match the participants information with information provided by the ART register to see if the participant initiated ART or not. If a participant did not initiate care (i.e. you cannot find him in the ART register), still enter the initial data points and indicate that the participant did not

Code	Question	Responses
pid	Please enter the Participant ID	
district	District	☐ Chickwawa<br>☐ Nkhotakota<br>☐ Lilongwe<br>☐ Kasungu
site	Facility name	☐ Chickwawa District Hospital<br>☐ St. Montford Mission Hospital<br>☐ Kalemba Community Hospital<br>☐ Kasungu District Hospital<br>☐ Nkhoma Community Hospital<br>☐ Mponela Rural Hospital<br>☐ Deayang Luke Hospital<br>☐ Nkhotakota District Hospital

For peer review only - http://bmjopen.bmj.com/site/about/guidelines.xhtml

		☐ Nsanje District Hospital<br>☐ Ngabu Rural Hospital
	We want to know if this participant initiated ART. Please look at the ART register used at the clinic between DATE MONTH YEAR up to today<br><br>*Instructions: Look for the below information in the ART register, matching the below participant with a name in the ART register.*<br><br>*Sometimes it is hard to find an exact match in the ART register. Consider it a match if 3 of the 4 data points match. For example, someone's name may be different, but the age, and village/residence matches. Consider this the same person.*<br><br>*CLIENT NAME:*<br>*AGE:*<br>*Ta:*<br>*Village:*	
found_art	Was the participant found in the ART register?	☐ Yes (1) – proceed to next question<br>☐ No (0) – end survey
art_number	What is the participant assigned ART number?	
art_date	What is the clients ART start date?	___ ___/___ ___/___ ___<br>Day Month Year

For peer review only

	Use the ART number to find the paper Mastercard OR look up the participant in the Baobab system	
mastercard_found	Did you find the mastercard/baobab record?	☐ Yes (1) – proceed to next question<br>☐ No (0) – end survey
tb	At initiation: TB	☐ Yes (1)<br>☐ No (0)
ks	At initiation: KS	☐ Yes (1)<br>☐ No (0)
pillcount	Number of pills given	
nextapp_date	Date of next appointment	___ ___/___ ___/___ ___<br>Day Month Year
nextconsult_date	Date of next consultation visit	___ ___/___ ___/___ ___<br>Day Month Year

END OF SURVEY

For peer review only

14.10. APPENDIX J: In-Depth Interview Guide – Female

IN-DEPTH INTERVIEW GUIDE
Identifying efficient linkages strategies for HIVST (IDEaL)
Female

BEGIN RECORDING

Original Study ID: ______________________

Repeat Original Study ID:

State if male or female respondent ______________________

Health Facility: ______________________

Date of Interview: ______________________

Full Name of Interviewer: ___________________________________

District where respondent Lives: ___________________________________

Open- Ended Questions

Note: The in-depth interview will be open-ended and guided by the respondent's answers. This outline reflects a general guide for the in-depth interviews.

The interviews are meant to help us understand barriers and facilitators to ART initiation. We are also interested in their thoughts on new interventions we are developing to help men start ART. The following questions are meant to guide interviewers. Actual questions asked during the interview will vary based on participant responses.

DEMOGRAPHICS:

Demographics on the female participant not collected. Already in the original study. Just make sure the Original Study ID is documented correctly.

1. What type of job/work does your partner do?
    a. If he does not work, why?
    b. Does he do anything else to earn money?
2. What time of day/week is your partner usually busy?
3. Where does he spend most of his time when he is not working?
    a. *Probe: At the bar, watching football, at church, at home…*
4. How long have you been in a relationship with your sexual partner (whom you gave the HIVST kit to)

For peer review only

HIV TESTING:

Now I'd like to talk to you about you and your partner's experience with HIV services

1) To your knowledge, has your partner tested for HIV before you gave him the HIV-self testing kit?
    a) IF YES, did he tell you the result? What was his result?
    b) IF HIV+: At that time, did he start ART? IF NO: Why not?
2) Think about when you gave the HIV self-test kit to your sexual partner (show the kit).
    a) How did your partner feel about his HIV positive test result? How did the result affect him?
    b) How did you feel after your partner received an HIV positive status on the HIV self-test kit? How did the result affect you?
    c) Did your relationship change at all after he received an HIV-positive status? How?
    d) Did he talk to anyone about it?/ who?
    e) Did he disclose to anyone besides you? Why/why not?

ART UPTAKE:

1) After he tested HIV-positive, did he initiate ART? Why/Why not?
    a) IF INITIATED ART: How long did it take him to initiate ART? (several weeks, several months?). Why did you initiate ART so quickly/slowly? /

We know starting ART is difficult.

1) What do you think is the most difficult thing about starting ART for your partner right now?
    a) Do you think this is the same for men and for women? How is it different?
    b) How does taking ART affect one's daily activities? (schedule/routine) In bad ways? In good ways? Is it different for men? How?
    c) How does taking ART affect relationships? In bad ways? In good ways? Is it different for men? How?
    d) Out of all these things you mentioned, what do you think is the biggest concern/problem for your partner?

<u>FOR MEN WHO ARE CONSIDERED LOSS-TO-FOLLOW UP</u>

1. Since we saw you last, we haven't been able to follow up with your partner. From your perspective, why do you think this is?
2. Since your partner was enrolled in our study, what kind of things did you notice or did he mention to you related to HIV health services?
    a. *Probe: phone calls/SMS or speak one-on-one to a health care worker? Was your partner visited by a health care professional within your home or in the community?*
3. Has your partner mentioned any of these interactions to anyone?
    a. *Probe: friends, family, community members*
4. How did your partner react to these things? Did he like/dislike them? What did he do when they happened?
    a. *Probe: phone call, SMS, in-person visit, home-based ART*
5. How did you react to these things? Did you like/dislike them?
    a. *Probe: Was there any reaction amongst your family, friends or the community to these things?*
    b. *If so, how did this affect your partner and/or you?*

SUGGESTIONS FOR ART SERVICES

Thank you for all the information. We would like to develop ART services that meet the needs of individuals in your community. We understand that men may face different challenges than women. I would like to know your opinion about what is needed in order to make ART services easy to access and use.

1) Could you describe the ideal way ART services would be given to your partner? If the clinic could do anything …
   a) When would he want to pick up ART?
      i) Probe: day of week, time of day?
      ii) *Why do you say this?*

   b) Where would he want to pick up ART*?*
      i) Probe: clinic near you, clinic far from you, somewhere in the community (WHERE SPECIFICALLY), at your home?
      ii) *Why do you say this?*

2) Do you think your partner needs more HIV-related information? About the benefits of ART, how to keep his status a secret, or how to disclose his status, about other aspects of his health?
   a) IF YES: How would he want to get this information? In person (one on one, pamphlet, radio, phone call, …)
      i) Probe: IF YES: WHO would he like to talk to about this information? (Provider, expert client, other community member)

3) Does he need support? For example, reminders to go to the health facility, someone to talk to regularly about what he is going through with ART, help disclosing his status, anything else).
   a) Probe: IF YES: How would he want to get this support? In person (one on one, pamphlet, radio, phone call)
   b) Probe: IF YES: WHO would he like to talk to about this support? (Provider, expert client, other community member)

4) Out of time of pick up, location, more information, or more support, what are the most important factors for your partner to use ART services?
   a) *PROBE: Think about other males. What do you think they would say is the most important factor for men to use ART services?*

SPECIFIC SUGGESTIONS ON CURRENT INTERVENTIONS

<u>FOR MEN WHO ARE CONSIDERED LOSS-TO-FOLLOW UP</u>

1. As discussed earlier, your partner received [phone calls, texts, in person counseling, home-based ART].
   a. *Do you feel that this was enough to encourage him to start ART?*
      i. *If NO, what would you have done differently? (frequency, content, location)*
      ii. *If YES, why do you think they were sufficient?*
   b. Are there any other ideas/services we should think about doing beside the ones we just talked about (appointment reminders, in-depth counseling, community/home ART)?/
      i. PROBE: What is it?
      ii. Why do you think this could work?

For peer review only

CONCLUSION

Thank you for your time.

1. Is there anything else you would like to say about men's use of ART?
2. Is there anything else you would like to say about your own use of ART and how we can help make your experience better?

STOP THE RECORDER AND MAKE SURE RECORDING IS SAVED.

THANK YOU FOR YOUR PARTICIPATION IN THIS INTERVIEW. LET ME ENCOURAGE YOU THAT ARVS CAN HELP YOU LIVE LONG AND HEALTHY.

The following general education messages should be conveyed to all male and female participants:

- All people who have been tested HIV positive should start ART as soon as possible for their own health and to prevent passing the virus on to others.
- Serious diseases can occur even in patients with high CD4 count (>500), without any previous symptoms. Immediate ART greatly reduces this risk.
- People that start ART and continue lifelong without interruptions can remain healthy and live as long as people without HIV.
- Even though you may not feel sick, ART is still important to keep you healthy for the rest of your life.
- ART reduces the amount of virus in your body and therefore can reduce the chance that HIV is passed to your sex partners.
- Current ART regimens are easy to take and rarely cause serious side-effects. Some people have side effects in the first few weeks of treatment and these almost always go away. IF there are persistent side effects, an alternative HIV regimen can be given.

NOTE: Be careful not to give any specific medical advice but rather refer respondents back to the clinic to speak to a provider.

IN-DEPTH INTERVIEW GUIDE
Identifying efficient linkages strategies for HIVST (IDEaL)
Female (Chichewa)

BEGIN RECORDING

Original Study ID: ______________________

Repeat Original Study ID:

State if male or female respondent ______________________

Health Facility: ______________________

Date of Interview: ______________________

Full Name of Interviewer: __________________________________

District where respondent Lives: __________________________________

Demographics

1) Kodi bwenzi lanu limagwira ntchito yanji kuti apeze ndalama?
   a) Ngati sagwira ntchito ndi chifukwa chani?
   b) Kodi amapanga zinthu zina zothandizira kupeza ndalama?
2) Kodi ntha nd nthawi iti ya tsiku/sabata lomwe bwenzi lanu limakhala lotanganidwa?
3) Kodi nthawi yawo yambiri amakhala ali kuti ngati sakugwira ntchito?
4) Kodi mwakhala pa ubwenzi kwa nthawi yayitali bwanji ndi bwenzi lanu logonana nalo (lomwe munalipatsa ka chida koziyezela wekha HIV)

HIV-Testing

Pano ndimafuna ndikufunseni mafunso okhudzana ndi zomwe mwakumanapo nazo zokhudza thandizo la HIV.

1. Momwe mukudziwira kodi bwenzi lanu linayamba layezetsapo HIV musanawapatse kachida koziyeza wekha HIV?
   a. Ngati eya, anakuwuzani zotsatira? Zotsatira zawo zinali zotani?
   b. Ngati anapezeka ndi HIV nthawi imeneyo, anayamba kumwa ma ARV? Ngati ayi chifukwa chani?
2. Ganizirani nthawi yomwe mudapereka kachipangizo kodziyezera wekha HIV (awonetseni) kwa bwenzi lanu.
   a. Kodi bwenzi lanu linanva bwanji atadziwa za zotsatira zawo za HIV? Zotsatirazo anazilandira bwanji?
   b. Kodi munamva bwanji bwenzi lanu litalandira zotsatira zoti lili ndi kachilombo ka HIV

For peer review only

pa kachipangizoka? Izi zinakukhudzani bwanji?

c. Kodi ubwenzi wanu udasintha atalandira zotsatira zoti ali ndi kachilombo? Motani?
d. Kodi analankhulapo ndi wina aliyense? Mosatchula dzina, ndani?
e. Kodi anawuzapo wina aliyense za mthupi mwake kupatula inu? Chifukwa chani?

ART Uptake

1) Atadziyeza ndi kupeza kuti ali ndi kachilombo, kodi adakayamba kumwa mankhwala? Chifukwa?
   a) Kodi zinatenga nthawi yaitali bwanji asanayambe kumwa mankhwala a ma ARV? (masabata angapo, miyezi ingapo?) Kodi anayamba kumwa mankhwala mnsanga/mochedwa chifukwa chani?
2) Tikudziwa kuti kuyamba kumwa mankhwala a ARV kumavutirapo. Kodi mukuona kuti chovuta chachikulu pa kuyamba kumwa mankhwala ndi chani?
   a) Kodi mukuganiza kuti izi ndi zofanana kwa amuna ndi akazi? Nzosiyana chifukwa chani?
   b) Kodi kumwa mankhwala a ma ARV kumakhudza bwanji kagwiridwe ka ntchito ka munthu tsiku ndi tsiku (ntchito zomwe amagwiragwira) Mu njira yabwino kapena yobwera mmbuyo? Ndi zosiyana kwa amuna? Motani?
   c) Kodi kumwa mankhwala a ma ARV kumakhudza bwanji mwaubwenzi? Mu njira yabwino kapena yobwera mmbuyo? Ndi zosiyana kwa amuna? Motani?
   d) Pa zinthu zonse zomwe tambambikalana, mukuona kuti vuto lalikuru kwa bwenzi lanu ndi chani?

FOR MEN WHO ARE CONSIDERED LOSS-TO-FOLLOW UP

1) Chiwonelaneni ulendo watha, talephela kuonananso ndi bwenzi lanu. Mumaganizo anu mukuona kuti zili motere chifukwa chani?
2) *Chilowereni cha bwenzi lanu mu study, mwaonako zinthu zotani kapena zomwe atchulako zokhudzana ndi thandizo la HIV?*
   a) *Funsitsani: Lamya ya m'manja/uthenga wa pa lamya kapena kuyankhula ndi wazaumoyo? Kodi bwenzi lanu linayendeledwapo ndi wazaumoyo mu dela lanu kapena pafupi ndi nyumba yanu?*
3) Kodi bwenzi lanu lathculako mikumano iyi kwa wina aliyense?
   a) *Funsitsani: Anzawo,achibale, anthu a mudela*
4) Kodi bwenzi lanu linapangapo chani pa zinthu izi? Anazikonda/sana zikonde? Anapanga chani zitachitika?
   a) *Funsitsani: Lamya ya m'manja/uthenga wa pa lamya, kupita okha , thandizo la ARV lapakhomo*
5) Munanva bwanji kuzinthuzi? Munazikonda/simunazikonde?
   a) *Achibale anapanga chilichonse, anzanu,anthu a mudela ku zinthu zimenezi?*
   b) *Ngati zili choncho, izi zinawatani a bwenzi anu /inuyo*

SUGGESTIONS FOR ART SERVICES

Zikomo chifukwa cha mayankho anu. Timafuna titakhazikitsa ndondomeko zokomera anthu mu dera lanu pokhudza thandizo la ma ARV. Tikudziwa kuti amuna amakumana ndi zophinja/mavuto osiyana

For peer review only

ndi amayi. Ndimafuna ndimve maganizo anu pa zofunika kuti tikonze bwino thandizoli kuti likhale losavuta kufikira ndi kugwiritsa ntchito.

1) Momwe mukuganizira, kodi mungandiuzeko njira yabwino kwambiri yomwe mukuganiza kuti thandizo la ARV lingamaperekedwere kwa bwenzi lanu? Chipatala chitati chichitepo kanthu….
   a) Kodi bwenzi lanu lingafune lidzipita nthawi zotani ku chipatala kukatenga mankhwala
      i) Tsiku la mu sabata, nthawi?
      ii) *Ndi chifukwa chani?*
   b) Kodi ndi malo ati omwe bwenzi lanu lingakonde kumakatengerako ARV?
      i) Pa chipatala cha pafupi nanu, kutali nanu, kwina mu dela lino (MALO ATI KWENIKWENI) pakhomo panu
      ii) *Ndi chifukwa chani?*
2) Kodi mukuganiza kwanu, mukuona kuti bwenzi lanu likufunika uthenga wina owonjezera? Pa zokhudza ubwino wa ARV, momwe lingasungire chinsinsi cha momwe mthupi mwawo mulili kapena kuwauza ena za mthupi mwawo, zina zikhuzana ndi thanzi lawo?
   a) Bwenzi lanu lingakonde litalandira mu njira yotani uthengawu? Kukawapeza paokha (paokha, kulembedwa mu bukhu, pa wailesi, kuimbiridwa foni)
   b) Ndi NDANI amene bwenzi lanu lingakonde kulankhula naye za uthengawu? (Dokotala, Expert Client, anthu ena a mu dera)
3) Kodi mukuganiza kuti bwenzi lanu likufunika thandizo lapadera? Mwachitsanzo, kukumbutsidwa kuti lipite ku chipatala, kulankhula ndi munthu wina mowirikiza pa zomwe akukumana nazo pakumwa ARV, kuthandizidwa kuuza ena za mmomwe mthupi mwawo mulili ndi zina)
   a) *Bwenzi lanu lingakonde litalandira mu njira yotani thandizoli? Kukawapeza pawokha (pawokha, kulembedwa mu bukhu, pa wailesi, kuimbiridwa foni)*
   b) *Ndi NDANI amene bwenzi lanu lingakonde kulankhula naye za thandizoli? (Dokotala, Expert Client, anthu ena a mu dera)*
4) Potengera thandizo ndi chisamaliro cha ma ARV, pakati pa nthawi yokatengera mankhwala, malo ake, uthenga owonjezera ndi thandizo la padera, kodi mukuganiza kuti chofunikira kwambiri ndi chiti kwa bwenzi lanu?
   a) *Ganizirani amuna ena a mdera lanu. Kodi mukuganiza kuti iwo angatchule chani ngati chinthu chofunikira kwambiri kwa amuna pa zomwe tachula zija?*

SPECIFIC SUGGESTIONS ON CURRENT INTERVENTIONS

<u>FOR MEN WHO ARE CONSIDERED LOSS-TO-FOLLOW UP</u>

1) Monga takambirana kale poyamba,bwenzi lanu linalandira (Kuyimbilidwa lanya, uthenga wa pa lamya, malangizo, thandizo la ma ARV la pakhomo)
   a) Mukuwona ngati izi zinali zokwanira kulimbikitsa bwezi lanu kuyamba ma ARV?
      i) Ngati ayi ndi chani mukanapanga mosiyana? (malo)
      ii) Ngati eya mukuwona ngati zili zofunika chifukwa chani?
2) Kodi pali maganizo ena omwe mukuona kuti angathandizepo kupatula omwe tatchulawa (kukumbutsana, uphungu wapadera, kugawa mankhwala kumudzi) ndi chani?

a) Ndi chani?
b) Ndi chifukwa chani mukuona kuti zikhoza kuyenda bwino

CONCLUSION

1) Zikomo chifukwa cha nthawi yanu. Pali choonjezera china chilichonse chomwe mungalankhulepo chokhudza kagwiritsidwe ntchito ka thandizo la ARV ndi amuna?
2) Pali chilichonse chokhudza kagwiritsidwe ntchito kanu ka thandizo la ma ARV ndi momwe tingakonzere thandizoli?

STOP THE RECORDER AND MAKE SURE RECORDING IS SAVED.

ZIKOMO CHIFUKWA CHOTENGA NAWO MBALI MU KAFUKUFUKUYU. NDIMAFUNA NDIKULIMBIKITSENI KUTI MA ARV AKHOZA KUTHANDIZA INU KUKHALA MOYO WAUTALI NDINSO WATHANZI.

The following general education messages should be conveyed to all male and female participants:

- Anthu onse omwe ayezetsa ndi kupezeka ndi kachilombo ka HIV akuyenera kuyamba kumwa mankhwala a ma ARV mwamsangamsanga kuti zipundulire thanzi lawo komanso apewe kupatsira ena kachilombo.
- Matenda oopsa akhoza kugwira munthu ngakhale amene chiwerengero cha asilikali a mnthupi ndi ochuluka (kuposera 500) posaonetsa zizindikiro zoyamba. Mankhwala a ma ARV amachepetsa chiopsezochi.
- Anthu omwe ayamba kumwa ma ARV ndipo akupitiliza moyo wawo onse osalekalekeza akhoza kukhala moyo wa thanzi ndi wautali chimodzimodzi anthu omwe alibe kachilombo ka HIV.
- Ngakhale simukumva kudwala, mankhwala a ARV ndi ofunikirabe kuti mukhale ndi thanzi moyo wanu onse.
- Makhwala a ma ARV amachepetsa mlingo wa tidzilombo mthupi mwanu kotero amachepetsa chiposezo chopatsira kachilombo kwa ena.
- Mankhwala omwe alipo pakadali pano a ma ARV ndiosavuta kumwa komanso sakhala ndi mavuto ambiri. Anthu ena amakhala ndi mavuto obwera kamba komwa mankhwala masabata oyambirira koma izi zimatha. Ngati zikupitilirabe pali mtundu wina wa mankhwala omwe akhoza kukupatsani.

For peer review only - http://bmjopen.bmj.com/site/about/guidelines.xhtml

14.11. APPENDIX K: In-Depth Interview Guide – Male

IN-DEPTH INTERVIEW GUIDE
Identifying efficient linkages strategies for HIVST (IDEaL)
Male

BEGIN RECORDING

Original Study ID: ____________________

Repeat Original Study ID:

State if male or female respondent ______________________

Health Facility: ______________________

Date of Interview: ______________________

Full Name of Interviewer: _______________________________

District where respondent Lives: ________________________________

DEMOGRAPHICS:

1. What is your current age in years?	Age in ☐☐ Completed Years
2. How would you rate your health today on a scale from 1-5 with 1 being excellent health and 5 being the very poor health?	☐ Excellent (1)<br>☐ Very good (2)<br>☐ Good (3)<br>☐ Fair (4)<br>☐ Poor (5)
3. Now I'd like to ask about your relationships. Are you currently in a sexual relationship?	☐ Yes (1)<br>☐ No (0)<br>**If NO, skip to QUALITATIVE**
4. Does your partner know <u>your</u> HIV status?	☐ Yes (1)<br>☐ No (0)

For peer review only

5. How many times have you tested for HIV?	
6. When was the first time you tested HIV-positive?	

Open- Ended Questions

Note: The in-depth interview will be open-ended and guided by the respondent's answers. This outline reflects a general guide for the in-depth interviews.

The interviews are meant to help us understand barriers and facilitators to ART initiation. We are also interested in their thoughts on new interventions we are developing to help men start ART. The following questions are meant to guide interviewers. Actual questions asked during the interview will vary based on participant responses.

SCRIPT: Now I'd like to talk to you about your experience with HIV services.

HIV TESTING

1. Think about when you used an HIV self-test kit (show the kit). When did you use it?
2. How did you feel after receiving the HIV positive status?
3. Can you talk to me about what happened after you tested HIV positive? Walk me through it so I can see the picture in detail?.
    a. Did you talk to anyone about it?
    b. Has your HIV status changed your daily activities at all? (Your schedule/routine)
    c. Has your HIV status impacted your relationships? How?
    d. Have you disclosed your status to anyone besides your partner? Why/why not

INTERVENTION

1. After you tested positive, we approached you to be a part of our study. Since you were enrolled in the study (ie in the last 3 months), can you walk me through what has happened related to HIV health services?
    a. Probe: What kinds of interactions have you had with expert clients or health personelle (phone call, SMS, in-person visits, home based ART).
    b. What was the frequency of these interactions (weekly, every other week, monthly)

ART INITIATION

2. Since you have been enrolled in the study, have you initiated ART?

<u>INITIATED</u>

3. How long did it take you to initiate ART? (several weeks, several months?). Where did you initiate?
    a. Why did you initiate ART so quickly/slowly?
4. Since you initiated treatment, have you continued to take you medication?

a. Have you returned to the clinic for another refill of ART? Have there been challenges to staying on treatment – how have you overcome them?

NOT INITIATED

1) Since you tested HIV-positive, have you been to a health facility?
   a) When did you attend? (year)
   b) Why did you attend? (guardian vs client; HIV vs OPD)
   c) Why did you not initiate ART during this visit?

ART UPTAKE

2) We know starting ART is difficult. What do you think is most the most difficult thing about starting ART?
   a) PROBE: Think about your male friends. What do you think they would say is the most difficult part about starting ART for men in your village?

INITITATED ART

1) How has taking ART affected your daily activities at all? (your schedule/routine) In bad ways? In good ways?
2) How has taking ART affected your relationships? In bad ways? In good ways?
3) Does being on ART change if you are able to hide/keep your HIV status from other people/ If you are able to hide/keep your status other people, does that make it easier to be on ART? How?

NOT INITITATED ART

1) How do you think taking ART would affect your daily activities at all? (your schedule/routine) In bad ways? In good ways?
2) How do you think taking ART would affect your relationships? In bad ways? In good ways?
3) Would being able to hide/keep your HIV status from other people make it easier for you to be on ART? How?

4) What do you think is most the SECOND most difficult thing about starting ART?

   a) PROBE: Think about your male friends. What do you think they would say is the SECOND most difficult part about starting ART for men in your village?

SUGGESTIONS FOR ART SERVICES

Thank you for all the information. We would like to develop ART services that meet the needs of men in your community. We understand that men are busy and may face different challenges than women. I would like to know your opinion about what is needed in order to make ART services easy to use for men in your community

1) Could you describe the ideal way ART services would be given to you? If the clinic could do anything
   a) When would you want to pick up ART?
   b) Probe: day of week, time of day?
   c) Why do you say this?
2) Where would you want to pick up ART?
   a) Clinic near you, clinic far from you, somewhere in the community (WHERE SPECIFICALLY), at your home?
   b) Why do you say this?

For peer review only

I would like to learn about how you felt about each of the interactions we talked about earlier (remind participant of what they mentioned – ex: phone calles, texts, in person visits, home based ART)

1) What did you like about them?
   a) Why? (ex: individual follow-up, sense of support, not having to travel to the clinic for homebased ART)
2) What did you dislike about them? What were challenges?
   a) Why? (ex: difficulty maintaining privacy with contact or visits, doesn't want to start ART for other reasons)
3) Do you feel like these things helped encourage you to seek health services?
   a) If YES, why?
   b) If NO, why?
4) Do you think these things would help other men in your community if they were to test positive for HIV?

<u>INITIATED</u>

1) Do you feel that these things helped to encourage you to initiate and stay on ART?
2) Do you think you would have started treatment without them?

<u>NOT INITIATED</u>

1) Why do you think these things failed to help you start/stay on treatment?
   a) Why?
2) If you could change anything about these interactions that you have listed, what would you change?
   a) Probe: Type of contact, frequency of contact, personelle, location, topics covered

3) We understand that everyone is different. Beyond what you have experienced, do you still have problems related to seeking health services for HIV? (i.e. are there still things that you need?)
   a) What are these unmet needs?
   b) What do you feel would be the best solution to meet those needs?
4) Are there any other ideas/services we should think about doing beside the ones we just talked about (appointment reminders, in-depth counseling, community/home ART)?
   a) What is it?
   b) Why do you think this could work?
5) Is there anything else that you would like to add as we are towards the end of the interview?

STOP THE RECORDER AND MAKE SURE RECORDING IS SAVED.

THANK YOU FOR YOUR PARTICIPATION IN THIS INTERVIEW. LET ME ENCOURAGE YOU THAT ARVS CAN HELP YOU LIVE LONG AND HEALTHY.

The following general education messages should be conveyed to all male and female participants:

- All people who have been tested HIV positive should start ART as soon as possible for their own health and to prevent passing the virus on to others.

For peer review only

- Serious diseases can occur even in patients with high CD4 count (>500), without any previous symptoms. Immediate ART greatly reduces this risk.
- People that start ART and continue lifelong without interruptions can remain healthy and live as long as people without HIV.
- Even though you may not feel sick, ART is still important to keep you healthy for the rest of your life.
- ART reduces the amount of virus in your body and therefore can reduce the chance that HIV is passed to your sex partners.
- Current ART regimens are easy to take and rarely cause serious side-effects. Some people have side effects in the first few weeks of treatment and these almost always go away. IF there are persistent side effects, an alternative HIV regimen can be given.

Be careful not to give any specific medical advice but rather refer respondents back to the clinic to speak to a provider.

For peer review only

IN-DEPTH INTERVIEW GUIDE
Identifying efficient linkages strategies for HIVST (IDEaL)
Male (Chichewa)

BEGIN RECORDING

Original Study ID: ______________________

Repeat Original Study ID:

State if male or female respondent ______________________

Health Facility: ______________________

Date of Interview: ______________________

Full Name of Interviewer: ________________________________

District where respondent Lives: ________________________________

1. Kodi pakadali pano muli ndi zaka zingati zakubadwa?	
2. Kodi thanzi lanu mungati lilibwanji lero pa mlingo wa 1 mpaka 5, pamene 1 akuyimira thanzi labwino kwambiri ndi 5 akuyimira kuti thanzi lanu silili bwino konse?	☐ Lili bwino kwambiri (1)<br>☐ Lilibwino (2)<br>☐ Pakati mpakati (3)<br>☐ Silili bwino (4)<br>☐ Silili bwino ngakhale pang'ono (5)
3. Pano ndimafuna ndikufunseni za maubwenzi. Kodi pakadali pano muli pa ubwenzi ogonana?	☐ Eya (1)<br>☐ Ayi (0)<br>**If NO, skip to QUALITATIVE**
4. Kodi bwenzi lanu limadziwa kuti mthupi mwanu muli bwanji kumbali ya HIV?	

For peer review only

5. Mwayezetsa kokwana kangati HIV?	
6. Kodi ulendo oyamba omwe mudayezetsa ndi kukupezani ndi kachilombo ka HIV ndi liti? (chaka)	

Pano ndimafuna ndikufunseni mafunso okhudzana ndi zomwe mwakumana nazo zokhudza ndi thandizo la HIV.

HIV TESTING

1) Ganizirani nthawi yomwe mudagwiritsa ntchito kachipangizo kodziyezera wekha HIV (awonetseni), kodi liti mudagwiritsa kachipangizokoa?
2) Kodi munamva bwanji mutaona kuti zikuonetsa kuti muli ndi kachilombo ka HIV?
3) Mungandifotokozele zomwe zinachitika mutayezetsa ndikupezeka ndi HIV? Mugafotokoze mwatsatanetsatane kuti ndione chithunzithunzi cha momwe zinalili?
   a) Munalankhula ndi aliyense?
   b) Kodi kudziwa kuti muli ndi kachilombo ka HIV kunasintha ntchito zanu za tsiku ndi tsiku?
   c) Kodi kudziwa kuti muli ndi kachilombo ka HIV kunakhudza maubwenzi anu? Motani?
   d) Kodi munayamba mwauzapo za momwe mthupi mwanu mulili kupatula kwa okondedwa anu? Chifukwa chani?

INTERVENTION

1) Chifukwa choti munapezeka ndi HIV, tinakupezani kuti mutenge nawo mbali mu study yathu. Poti munatenga nawo mbali mu study (miyezi itatu yapitayi),mungandifotokozere zomwe zakhala zikuchitika zokhudza ndi thandizo la HIV?
   a) Mwakhala ndi mikumano yotani ndi omwe ali kale pa ma ARV kapena a zaumoyo(pa lamya,uthenga wa lamya yam'manja, kuyankhulana pamaso, kupita nokha kukakumana nawo,kulandira thandizo la ma ARV pakhomo).
   b) Kodi mikumanoyi imachitika pafupipafupi bwanji?

ART INITATION

1) Chiloweleni mu study, mwayamba kulandira ma ARV?

<u>*OYAMBA MANKHWALA*</u>

2) Kodi zinatenga nthawi yaitali bwanji musanayambe kumwa mankhwala a ARV? (masabata angapo, miyezi ingapo?) Kodi mukanayambira kuti? Kodi munayamba kumwa mankhwala mnsanga/mochedwa chifukwa chani?

3) Chiyambileni kulandira thandizo la ARV, kodi mukupitiliza kumwa mankwala anu?
   a) Mwapitako ulend wina kuchipatala kukalandira mankwala ena a ama ARV? Mwakhala mukukumana ndi zovuta zina chifukwa chokumwa mankhwala ama ARV, kodi mavutowa mwawathetsa bwanji?

For peer review only

OSAYAMBA MANKHWALA

4) Mutayezetsa nkupezeka ndi kachilombo ka HIV, munayamba mwapitapo ku chipatala?
   a) Munapita liti? (chaka)
   b) Munapita chifukwa chani? (kuperekeza odwala wina kapena munadwala?; munapitila HIV kapena ku OPD)
   c) Kodi simunayambe kumwa mankhwala a ma ARV nthawi imeneyi chifukwa chani?

ART UPTAKTE

1) Tikudziwa kuti kuyamba kumwa mankhwala a ARV kumavutirapo. Kodi mukuona kuti chovuta chachikulu pa kuyamba kumwa mankhwala ndi chani?
   a) Ganizirani anzanu aamuna mmudzi mwanu. Kodi mukuona kuti iwo angatchule chani ngati chovuta chachikulu pa kuyamba kumwa mankhwala a ART?

OYAMBA MANKHWALA

2) Kodi kumwa mankhwala kwakhudza bwanji ntchito zanu za tsiku ndi tsiku? Mu njira yabwino kapena yobwerera mmbuyo?
3) Kodi kumwa mankhwala kwakhudza bwanji maubwenzi anu? Mu njira yabwino kapena yobwerera mmbuyo?
4) Kodi kumwa mankhwala a ARV kumaphweka mukabisa momwe mthupi mulili kwa anthu ena? Motani?

OSAYAMBA MANKHWALA

5) Kodi mukuganiza kuti kumwa ma ARV kungakhudze bwanji ntchito zanu za tsiku ndi tsiku? Mu njira yabwino kapena yobwerera mmbuyo?
6) Kodi mukuganiza kuti kumwa mankhwala a ma ARV kungakhudze bwanji maubwenzi anu? Mu njira yabwino kapena yobwerera mmbuyo?
7) Kodi kumwa mankhwala a ARV kungaphweke/kumaphweka kuti mukabisa momwe mthupi mulili kwa anthu ena? Motani?

8) Kodi chinthu chachiwiri chovuta kwambiri pa kuyamba kumwa mankhwala ndi chani?
   a) Ganizirani amuna ena a mmudzi mwanu. Kodi mukuganiza kuti anganene kuti chovuta CHACHIWIRI pa kuyamba kumwa a ma ARV ndi chani mmudzi mwanu?

SUGGESTIONS FOR ART SERVICES

Zikomo chifukwa cha mayankho anu. Timafuna titakhazikitsa ndondomeko zokomera amuna mu dera lanu zokhudza thandizo la ma ARV. Tikumvetsetsa kuti amuna amakhala otangwanidwa ndipo amakumana ndi mavuto osiyana ndi amayi. Ndimafuna ndimve maganizo anu pa zofunika kuti tikonze bwino thandizoli maka kwa amuna mu dera lino.

1) Kodi mungandiuzeko njira yabwino kwambiri yomwe mukuganiza kuti thandizo la ma ARV lingamaperekedwere kwa inu, chipatala chitati chichitepo kanthu….
   a) Kodi mungafune mudzipita nthawi zotani ku chipatala kukatenga mankhwala?
      i) Tsiku la mu sabata, nthawi?
      ii) Ndi chifukwa chani?
   b) Kodi ndi malo ati omwe mungakonde kumakatengerako ARV?
      i) Pa chipatala cha pafupi nanu, kutali nanu, kwina mu dela lino (MALO ATI KWENIKWENI) pakhomo panu
      ii) Ndi chifukwa chani?
2) Ndimafuna ndinve za momwe mukunvera za kucheza konse tinali nako poyamba(Kumbutsani otenga nawo mbali zomwe anatchula-kuyimbilidwa lamya, uthenga wa pa lamya, kupitako okha, thandizo la pakhomo la ARV)
   a) Chomwe munakondapo chinali chani?
      i) Chifukwa?(Kuyendeledwa, kunva kuthandizidwa, osafunika kupita ku chipatala kukalandira ma AR olandilira pakhomo)
   b) Chomwe simunaonde ndi chani? Zovuta zinali chani?
      i) Chifukwa? (chitsanzo: kuvutika kusunga chinsinsi ndi owadziwa kapena mikumano,simukufuna kuyamba kumwa ma ARV pazifukwa zina)
   c) Mukuganiza kuti zinthu zimennezi zakulimbikitsani kupeza thandizo la zaumoyo?
      i) Ngati eya , chifukwa?
      ii) Ngati ayi, chifukwa chani?
   d) Kodi mukuona ngati zinthu zimenezi zingathandize anthu ena mu dela lanu ngati angakhale ndi HIV?

OYAMBA MANKHWALA

1) Kodi mukuona ngati zinthu zimenezi zinakuthandizani kulimbikitsika kuti muyambe komanso kupitiliza kumwa mankhwala a ma ARV?
   a) Kodi mukuganiza kuti mukanatha kuyamba thandizo la ma ARV popanda zimenezi?

OSAYAMBA MANKHWALA

1) Kodi mukuganiza kuti zinthu zimenezi zinalephera kukuthandizani kuyamba/kukhala mankhwala a ma ARV?
   a) Chifukwa?
2) Ngati mungathe kusintha chilichonse cha mikumano yomwe mwatchula,chingakhale chani?
   a) Mtundu wa mkumano, muligo wa mkumano, muthu, malo,mitu yokambilana
3) Tikunvetsa kuti anthu ndife osiyana. Kuposa zomwe mwakumana nazo, pakadali pano mukukumanabe ndi mavuto okhudzana ndi kupeza thandizo la zaumoyo la HIV? (pali zinthu zina zomwe mumafunabe?)
   a) Ndi zinthu ziti?
   b) Mukuganiza kuti ndi njira yanji yabwino yothandiza kupeza zofunikazi?

For peer review only

4) Kodi pali maganizo ena omwe mukuona kuti angathandizepo kupatula omwe tachulawa (kukumbutsana, uphungu wapadera, kugawa mankhwala kumudzi) ndi chani? Mukuona kuti maganizo anuwa angatheke chifukwa chani?\
5) Apa tikufunakumaliza kucheza kwathu, pali china chiwinjezera chomwe mungalankhulepo pa zomwe takambirana?

STOP THE RECORDER AND MAKE SURE RECORDING IS SAVED.

ZIKOMO CHIFUKWA CHOTENGA NAWO MBALI MU KAFUKUFUKUYU. NDIMAFUNA NDIKULIMBIKITSENI KUTI MA ARV AKHOZA KUTHANDIZA INU KUKHALA MOYO WAUTALI NDINSO WATHANZI.

The following general education messages should be conveyed to all male and female participants:

- Anthu onse omwe ayezetsa ndi kupezeka ndi kachilombo ka HIV akuyenera kuyamba kumwa mankhwala a ma ARV mwamsangamsanga kuti zipundulire thanzi lawo komanso apewe kupatsira ena kachilombo.
- Matenda oopsa akhoza kugwira munthu ngakhale amene chiwerengero cha asilikali a mnthupi ndi ochuluka (kuposera 500) posaonetsa zizindikiro zoyamba. Mankhwala a ma ARV amachepetsa chiopsezochi.
- Anthu omwe ayamba kumwa ma ARV ndipo akupitiliza moyo wawo onse osalekalekeza akhoza kukhala moyo wa thanzi ndi wautali chimodzimodzi anthu omwe alibe kachilombo ka HIV.
- Ngakhale simukumva kudwala, mankhwala a ARV ndi ofunikirabe kuti mukhale ndi thanzi moyo wanu onse.
- Makhwala a ma ARV amachepetsa mlingo wa tidzilombo mthupi mwanu kotero amachepetsa chiposezo chopatsira kachilombo kwa ena.
- Mankhwala omwe alipo pakadali pano a ma ARV ndiosavuta kumwa komanso sakhala ndi mavuto ambiri. Anthu ena amakhala ndi mavuto obwera kamba komwa mankhwala masabata oyambirira koma izi zimatha. Ngati zikupitilirabe pali mtundu wina wa mankhwala omwe akhoza kukupatsani.

Be careful not to give any specific medical advice but rather refer respondents back to the clinic to speak to a provider.

14.12. APPENDIX L: Personel CV's

Kathryn L. Dovel

Department of Medicine, Division of Infectious Diseases
Email: kdovel@mednet.ucla.edu

David Geffen School of Medicine at UCLA
Office: +1 (310) 825-7225

10833 Le Conte Ave 37-121 CHS
Fax: +1 (310) 825-3632

Los Angeles, CA 90095

POSITIONS HELD

2017- Adjunct Assistant Professor, Division of Infectious Disease
Department of Medicine, David Geffen School of Medicine - UCLA

2017- Research Director - Partners in Hope
Lilongwe, Malawi

2016-17 Postdoctoral Fellow in Global HIV Prevention Research
David Geffen School of Medicine - UCLA, 2016

2012-15 International Programs Director - 31Bits International
Gulu, Uganda

EDUCATION

PhD Health and Behavioral Sciences - University of Colorado Denver, 2016

Outstanding CLAS Ph.D. Student for the University of Colorado Denver

Outstanding Dissertation Award for the University of Colorado Denver

Dissertation: "Shifting Focus from Individuals to Institutions: The Role of Gendered Health Institutions on Men's use of HIV Services"

MPH Community Health Sciences - UCLA, 2010

Certificate in Global Health

BA Sociology & Anthropology (dual major), minor in Biology - Vanguard University, 2007

Magna Cum Laude

HONORS AND AWARDS

2019 5th place in the 2019 Department of Medicine Research Day poster competition, Department of Medicine, University of California Los Angeles

2019 Invited panelist, IAS 2019, Sticky and durable linkage: Latest evidence and new strategies

2019 Panel Chair, IAS Pre-Conference 2019, Men and HIV: What we know and what we don't know

2019 Top 5 abstracts at CROI, 2019

2019 Invited participant, Technical Consultation on HIV Linkage, International AIDS Society

2019 Invited participant, Differentiated Service Delivery Think Tank, Gates Foundation

2018 Joep Lange Award (best abstract at INTEREST, 2018)

2018 Female Global Scholar, The Women in Global Health Research Initiative (Weill Cornell Medicine, Cornell University)

2016 Outstanding Dissertation Award (UCDenver)

2016 Outstanding CLAS Ph.D. Student (UCDenver)

2012-16 Deans Travel Grant (UCDenver)

2007 Delta Kappa Honor Society (Vanguard University)

2007 Alpha Kappa Delta Honor Society (Vanguard University)

2007 Lambda Alpha Honor Society (Vanguard University)

2007 Anthropology Student of the Year (Vanguard University)

EXTERNAL GRANTS

2019-2023 Principle Investigator, Bill and Melinda Gates Foundation. (001423) "Identifying Effective Linkage Strategies for HIVST (IDEaL)"

2019-2024 Principle Investigator, Fogarty International Center. International Research Scientist Development Award (K01), K01TW011484. "Innovative strategies to increase ART Initiation and viral suppression among HIV+ men in Malawi".

2019-2020 Principle Investigator, Clinton Health Access Foundation. "The impact of facility HIV self-test scale up in Malawi: a mixed methods study"

2018-2021 Co-Investigator, The Conrad N. Hilton Foundation. Delivery of childhood development services as part of HIV treatment services in Malawi. Project implemented by UCLA and Partners in Hope.

2017-19 Principal Investigator, USAID. **Use of HIV self-test kits to increase identification of HIV-infected individuals and their partners: a Cluster Randomized Control Trial.** (sub-study within a large PEPFAR-USAID grant; PI: Risa Hoffman).

2017-19 Principle Investigator, Clinical Research Scholar, National Institutes of Health Loan Repayment Program.

2016-18 Co-Principle Investigaor, USAID. Test and Start: Tracking Uptake and Retention in Care using Standard Registry Data. (sub-study within a large PEPFAR-USAID grant; PI: Risa Hoffman).

2015 Principal Investigator, 31Bits International. "Evaluating the impact of a couple's livelihoods program on power dynamics and economic attainment among couples in northern Uganda." Project implemented by 31Bits International.

2014-16 Principal Investigator, NIMH National Research Service Award Predoctoral Individual Fellowship, F31-MH103078-01A1, "Gender Disparities in High-Risk PITC: The Role of Policy on Provider Practices", Impact Score: 14; Percentile: 2.0

2013-16 Principal Investigator, Stop AIDS Now!. "Evaluation of the 'Quality HIV and reproductive maternal and neonatal health services for women and young women in Africa through good

For peer review only

clinical governance and community-driven accountability'". Project implemented by the Clinton Health Access Initiative.

INTERNAL GRANTS

2016-18 Principal Investigator, UCLA Center for AIDS Research Seed Grant, University of California Los Angeles, "The Gendered Dynamics of ART Uptake and Retention under Universal Treatment Policies. Examining trends and ART barriers in Central Malawi"

2014 Principal Investigator, Calvin L Wilson Scholarship, University of Colorado Denver, "Gender and the provision of HIV testing: Examining how models of care influence men's use of testing services in southern Malawi"

2013-15 Principal Investigator, Dissertation Grant, University of Colorado Denver, "Gender Disparities in High-Risk PITC: The Role of Policy on Provider Practices"

2013-14 Principal Investigator, Robinson Durst Scholarship, University of Colorado Denver, "Gender disparities in high-risk PITC: Exploring the influence of feminized policy on provider practices in Malawi"

2009 Principal Investigator, Drabkin and Bixby International Scholarship, UCLA, "Evaluating barriers and facilitators of a nutrition program in the Bateyes of Dominican Republic"

2009 Principal Investigator, Global Health Grant, UCLA, "Evaluating a Nutrition Program in the Bateyes of Dominican Republic"

PUBLICATIONS

* represents MPH, PhD or medical students I mentored

2020 Cornell, Morna, Katherine Horton, Christopher Colvin, Andrew Medina-Marino, **Kathryn Dovel.** Raising the profile of men's health: the role of the research community: Letter to the editor. *Lancet*. Ahead of Print.

2020 **Dovel, Kathryn**, Mike Nyirenda, Frackson Shaba*, O. Agatha Offorjebe*, Kelvin Balakasi, Brooke Nichols, Khumbo Phiri*, Khumbo Ngona, Sundeep K Gupta, Risa Hoffman. "Facility-based HIV self-testing for outpatients dramatically increases HIV testing in Malawi: a cluster randomized trial." *Lancet Global Health*. Ahead of print

2020 **Dovel, Kathryn,** Khumbo Phii, Misheck Mphande, Deborah Mindry, Esnart Sanudi, McDaphton Bellos, Risa Hoffman. Optimizing Test and Treat in Malawi: Health care worker perspectives on barriers and facilitators to ART initiation among healthy clients. Global Health Action. Ahead of Print.

2020 Hubbard, Julie, Khumbo Phiri*, Corrina Moucheraud, Kaitlyn McBride, Ashley Bardon, Kelvin Balakasi, Eric Lungu, **Kathryn Dovel**, Gift Kakwesa, Risa Hoffman. A qualitative assessment of provider and client experiences with three- and six-month dispensing of antiretroviral therapy in Malawi. Global Health: Science and Practice. Ahead of Print.

For peer review only

2020	Hoffman, Risa M, Kelvin Balakasi, Ashley Bardon, Zumbe Siwale, Julie Hubbard, Gift Kakwesa, Mwiza Haambokoma, Thoko Kalua, Pedro Pisa, Crispin Moyo, **Kathryn Dovel**, Thembi Xulu, Ian Sanne, Matt Fox, Sydney Rosen. Eligibility for differentiated models of HIV treatment service delivery: an estimate from Malawi and Zambia. *AIDS.* 1;34(3):475-9.
2019	McBride, Kaitlyn, Julie Parent, Kondwani Mmanga, Mackenzie Chivwala, Mike H. Nyirenda, Alan Schooley, James B. Mwambene, **Kathryn Dovel**, Eric Lungu, Kelvin Balakasi, Risa M. Hoffman, Corrina Moucheraud. "ART Adherence Among Malawian Youth Enrolled in Teen Clubs: A Retrospective Chart Review." *AIDS Behav.* (2019): 1-5.
2019	Frackson Shaba*, Ogechukwu Offorjebe*, Phiri Khumbo, Lungu Eric, Kalande Pericles, Nyirenda Mike, Hoffman M Risa, Gupta Sundeep, **Dovel Kathryn**. Perceived Acceptability of a Facility-Based HIV Self-Test Intervention in Outpatient Waiting Spaces Among Adult Outpatients in Malawi: A Formative Study. *JAIDS*. 1;81(3):e92-4.
2019	Magaço Amílcar, **Dovel Kathryn**, Cataldo Fabian, Nhassengo Pedroso, Nuera Lucas, Tique José, Saide Mohomed, Couto Aleny, Mbofana Francisco, Gudo E Eduardo, Cuco Rosa Marlene, Chicumbe Sérgio. "Good health as a barrier and facilitator to ART initiation: a qualitative study in the era of Test and Treat in Mozambique." *Cult Health Sex. 11:1-5.*
2018	Cornell M, **Dovel K**. Reaching key adolescent populations. *Cur Opinion HIV AIDS.* 1;13(3):274-80.
2018	Sara, Yeatman, Stephanie Chamberlin*, **Kathryn Dovel.** Women's (health) work: A population-based, cross-sectional study of gender differences in time spent seeking health care in Malawi. *PLoS ONE.* 13(12): e0209586
2018	Nhassengo, Pedroso Fabian Cataldo, Amílcar Magaço, Risa Hoffman, Lucas Nuera, José Tique, Mohomed Saide, Aleny Couto, Francisco Mbofana, Eduardo Gudo, Rosa Marlene Cuco, Sérgio Chicumbe, **Kathryn Dovel**. "Barriers and facilitators to the uptake of universal treatment in Mozambique: a qualitative study on patient and provider perceptions." *PLoS ONE.* 13(12): e0205919
2018	Hubbard, Julie, Gift Kakwesa, Mike Nyirenda, James Mwambeneb, Ashley Bardona, Kelvin Balakasi, **Kathryn Dovel**, Thokozani Kaluac, Risa Hoffman. Towards the third 90: improving viral load testing with a simple quality improvement program in health facilities in Malawi. *International Public Health.* Ahead of print.
2018	**Dovel, Kathryn,** Frackson Shaba*, Ogechukwu Offorjebe*, Kelvin Balakasi, Khumbo Phiri*, Brooke Nichols, Chi-Hong Tseng, Ashley Bardon, Khumbo Ngona, Risa Hoffman. "Evaluating the integration of HIV self-testing into low-resource health systems: study protocol for a cluster randomized trial from EQUIP Innovations" *Trials.* 19:498.
2018	Moucheraud, Corrina, Dennis Chasweka, Mike Nyirenda, Alan Schooley, **Kathryn Dovel**, Risa Hoffman. "A simple screening tool may help identify high-risk children for targeted HIV testing in Malawian inpatient wards." *JAIDS.* 79:352-7.
2018	Cornell Morna, **Dovel Kathryn**. "Reaching key adolescent populations." *Current opinion*

in HIV and AIDS. 13(3):274-80.

2016 **Dovel, Kathryn**, Sara Yeatman, Joep Vanoosterhout, Adrienne Chan, Alfred Matengeni, Megan Landes, Richard Bedell, and Sumeet Sodhi. “Trends in ART Initiation among Men and Non-Pregnant/Non-Breastfeeding Women before and after Option B+ in Southern Malawi.” *PLoS ONE.* (12): e0165025.

2016 Poulin, Michelle, **Kathryn Dovel** and Susan Watkins. “Men with money and the 'vulnerable women' client category in an AIDS epidemic.” *World Development*. 85; 16-30.

2016 **Dovel, Kathryn**, Susan Watkins, Sara Yeatman, and Michelle Poulin. "Prioritizing strategies to reduce AIDS-related mortality for men in sub-Saharan Africa: Author’s reply.” *AIDS*. 30(1); 158-9.

2015 **Dovel, Kathryn,** Sara Yeatman, Susan Watkins, and Michelle Poulin. “Men’s heightened risk of AIDS-related death: the legacy of gendered HIV testing and treatment strategies.” *AIDS.* 29; 1123–5.

2015 **Dovel, Kathryn** and Kallie Thomson. “Financial obligations and economic barriers to antiretroviral therapy experienced by HIV positive women participating in a job-creation program in northern Uganda.” *Culture, Health, and Sexuality*. 18(6).

2015 Krueger, Patrick, **Kathryn Dovel** and Justin Denney. “Democracy and self-rated health across 67 countries: A multilevel analysis.*” Social Science and Medicine.* 143; 137-44.

2013 Conroy, Amy, Sara Yeatman and **Kathryn Dovel**. “The social construction of HIV/AIDS during a time of evolving access to antiretroviral therapy in rural Malawi.” *Culture, Health and Sexuality.* 15(8); 924-37.

2012 Yeatman, Sara, **Kathryn Dovel**, Amy Conroy and Hazel Namadingo. “The predictors of HIV treatment optimism and its relationship with sexual risk behavior among a population-based sample of young adults in southern Malawi.” *AIDS Care.* 25(8);1018-25.

TECHNICAL MANUSCRIPTS

2019 Hopkins, John, Laura Pascoe, Dean Peacock and Kathryn Dovel. “Accelerating Men’s HIV service delivery and uptake in Eastern and Southern Africa UNAIDS Literature Review, Eastern and Southern Africa Regional Focus.” UNAIDS, Johannesburg, South Africa.

2018 Masina, Tobias, **Kathryn Dovel**, Reuben Mwenda on behalf of the Malawi Ministry of Health. “National Guidelines for HIV self-testing.” Malawi Ministry of Health Lilongwe Malawi.

2017 Pascoe, Laura, Dean Peacock and **Kathryn Dovel**. “To Get to Zero, We Must Also Get to Men – UNAIDS Literature Review, Eastern and Southern Africa Regional Focus.” UNAIDS, Geneva.

2016 Macharia, Faith, Job Akuno, Faith Wanji, Julius Nguku, **Kathryn Dovel**, Caroline Ngare, Fred Nyagah, and Daniel Mwisunji. “National Guidelines for Male Engagement in HIV Services.” Kenya Ministry of Health. Nairobi, Kenya.

2016 **Kathryn Dovel**, James Mkandawire, Susan Watkins, Nancy Mulauzi and Sydney Rodney Lungu. “Evaluation of the Good Clinical Governance Project: improving HIV and reproductive health services in Lilongwe, Malawi.” Stop AIDS Now!. Lilongwe, Malawi.

For peer review only

OTHER PUBLICATIONS

2019 Kathryn Dovel, Stephanie Chamberlin, Sara, Yeatman. Malawi's Health System Puts Women First. This Isn't Always a Good Thing. *The Conversation: Africa.* Published February 19, 2019. Found at https://theconversation.com/malawis-health-system-puts-women-first-this-isnt-always-a-good-thing-111277

2016 **Dovel, Kathryn**, Sara Yeatman, and Susan Watkins. **Dying from a treatable disease: HIV and the men we neglect. *Huffington Post.* Published February 23, 2016. Found at** http://www.huffingtonpost.com/the-conversation-africa/dying-from-a-treatable-di_b_9295620.html

WORK IN PREPARATION

Dovel, Kathryn. "The gendered organization of HIV services and men's poor use of testing in southern Malawi: consequences of hegemonic masculinity within health institutions." (Revise & Resubmit, JIAS)

Offorjebe , Ogechukwu*, Frackson Shaba*, Kelvin Balakasi, Mike Nyrienda, Risa Hoffman, **Kathryn Dovel**. "Partner-delivered HIV self-testing increases the perceived acceptability of index partner testing among HIV-positive clients in Malawi." (Revise & Resubmit, PLoS ONE)

Dovel, Kathryn, Kelvin Balakasi, Khumbo Phiri*, Frackson Shaba*, O. Agatha Offorjebe*, Sundeep K Gupta, Vincent Wong, Eric Lungu, Brooke Nichols, Mike Nyirenda, Ngona K, Anteneh Worku, Risa Hoffman. "A randomized trial on index HIV self-testing for sexual partners of ART clients in Malawi." (Under Review)

Nichols, Brooke; Offorjebe, O. Agatha; Cele, Refiloe; Shaba , Frackson ; Balakasi, Kelvin; Chivwara, Mackenzie; Hoffman, Risa; Long, Lawrence; Rosen, Sydney; **Dovel, Kathryn**. "Economic evaluation of facility-based HIV self-testing among adult outpatients in Malawi. " (Under Review)

Dovel, Kathryn, Gladies Orobmi, Melanie Beagly*, Kallie Thomson. "Including men without sidelining women: the feasibility of male involvement within resource-strained gender equality programs in sub Saharan Africa." (Under Review)

Dovel, Kathryn and Kallie Thomson. "Evaluating the impact of a couple's livelihoods program on power dynamics and economic attainment among couples in northern Uganda." (In preparation)

Dovel, Kathryn. "Men in global HIV policy: examining discourses of blame and vulnerability." (In preparation)

SELECT PEER-REVIEWED PRESENTATIONS

2020 Moucheraud, Corrina, Samuel W. Lewis, Misheck Mphande, Ben Allan Banda, Hitler Sigauke, Paul Kawale, Aubrey Dkangoma, **Kathryn Dovel**, Alemayehu Amberbir, Agnes Moses, Sundeep Gupta, Risa M. Hoffman. Cervical cancer knowledge and attitudes among HIV-positive men in Malawi." Paper accepted for poster presentation. Conference on Retroviruses and Opportunistic Infections (CROI). Boston, Massachusetts, USA

2019 **Dovel, Kathryn**, Kelvin Balakasi, Khumbo Phiri*, Frackson Shaba*, O. Agatha Offorjebe*, Sundeep K Gupta, Vincent Wong, Eric Lungu, Brooke Nichols, Mike Nyirenda, Ngona K, Anteneh Worku, Risa Hoffman. "Index HIV self-testing among male partners in Malawi:

For peer review only - http://bmjopen.bmj.com/site/about/guidelines.xhtml

predictors of self-testing within a randomized controlled trial". Paper accepted for poster presentation. International AIDS Society. Mexico City, Mexico

2019 **Dovel Kathryn**, Salem Ejigu, Pericles Kalande, Evelyn Udedi, Chipawiru Mbalanga, Lauri Bruns, Thomas Coates. "Beyond the Caregiver: Diffusion of early childhood development knowledge and practices within the social networks of HIV-positive mothers in Malawi". Paper accepted for poster discussion. International AIDS Society. Mexico City, Mexico

2019 **Dovel, Kathryn**, Kelvin Balakasi, Khumbo Phiri*, Frackson Shaba*, O. Agatha Offorjebe*, Sundeep K Gupta, Vincent Wong, Eric Lungu, Brooke Nichols, Mike Nyirenda, Ngona K, Anteneh Worku, Risa Hoffman. "A randomized trial on index HIV self-testing for sexual partners of ART clients in Malawi." Paper accepted for oral presentation. Conference on Retroviruses and Opportunistic Infections (CROI). Seattle, Washington, USA

2019 Ogechukwu Offorjebe, **Kathryn Dovel**, Frackson Shaba, Kelvin Balakasi, Risa Hoffman, Sydney Rosen, Brooke Nichols, for the EQUIP Health team. Cost-effectiveness and national impact of index HIV self-testing in Malawi. Paper accepted for poster presentation. Conference on Retroviruses and Opportunistic Infections (CROI). Seattle, Washington, USA

2018 **Dovel, Kathryn**, Mike Nyirenda, Frackson Shaba*, Ogechukwu Offorjebe*, Kelvin Balakasi, Brooke Nichols, Khumbo Phiri*, Khumbo Ngona, Alan Schooley, Risa Hoffman on behalf of EQUIP Innovation for Health. "Facility-based HIV self-testing for outpatients dramatically increases HIV testing in Malawi: a cluster randomized trial." Paper accepted for oral presentation. International AIDS Society. Amsterdam, Netherlands

2018 Shaba, Frackson*, Kelvin Balakasi, Ogechukwu Offorjebe*, Mike Nyirenda, Risa Hoffman, **Kathryn Dovel** on behalf of EQUIP Innovation for Health. "Facility-based HIV self-testing in Malawi: an assessment of characteristics and concerns among clients who opt-out of testing." Paper accepted for poster presentation. International AIDS Society. Amsterdam, Netherlands

2018 **Dovel, Kathryn**, Mike Nyirenda, Frackson Shaba, Ogechukwu Offorjebe*, Kelvin Balakasi, Brooke Nichols, Khumbo Phiri*, Khumbo Ngona, Alan Schooley, Risa Hoffman on behalf of EQUIP Innovation for Health. "Facility-based HIV self-testing for outpatients dramatically increases HIV testing in Malawi: a cluster randomized trial." Paper accepted for oral presentation. INTEREST. Kigali, Rwanda - awarded the Joep Lange INTEREST award

2018 Offorjebe , Ogechukwu*, Frackson Shaba, Kelvin Balakasi, Mike Nyrienda, Risa Hoffman, **Kathryn Dovel** on behalf of EQUIP Innovation for Health. "Partner-delivered HIV self-testing increases the perceived acceptability of index partner testing among HIV-positive clients in Malawi." Paper accepted for mini-oral presentation. INTEREST. Kigali, Rwanda

2018 Stephanie Chamberlin*, Misheck Mphande, Pericles Kalande, **Kathryn Dovel** on behalf of EQUIP Innovation for Health. "Barriers and facilitators to consistent engagement in HIV care under Test and Treat in Malawi." Paper accepted for poster presentation. INTEREST. Kigali, Rwanda

2017 **Dovel Kathryn,** Khumbo Phiri*, Alan Schooley, Misheck Mphande, Mackenzie Chivwara, Risa Hoffman. "Facility-level barriers to antiretroviral therapy experienced by men in Malawi." Paper accepted for poster presentation. International AIDS Society. Paris, France

2017 Misheck Mphande, Khumbo Phiri*, Mackenzie Chivwara, Mike Nyirenda, Alan Schooley, Rachel Thomas, Risa Hoffman, **Kathryn Dovel**. "Examining Malawi's Rollout of Universal Treatment: Policy Implementation and Provider Perceptions." Paper accepted for poster presentation. International AIDS Society. Paris, France

2016 **Dovel, Kathryn**. "Factors influencing the implementation of provider-initiated testing and counseling (PITC) among STI clients in southern Malawi: A mixed methods study." Paper accepted for poster presentation. International AIDS Society. Durbin, South Africa

2016 Westerhof, Nienke, Dzowela M, **Kathryn Dovel**, E. Banda, J. Chikonda. "Community-driven accountability through advocacy committees: a vehicle for improving HIV and reproductive health services for women living with HIV." Paper accepted for poster presentation. International AIDS Society. Durbin, South Africa

2016 **Dovel, Kathryn**, Patrick Krueger, Shari Dworkin. "Predictors of men's use of HIV testing services in low-income countries: the role of masculinity." Paper accepted for poster presentation. Population Association of America. Washington D.C.

2014 **Dovel, Kathryn**. "Gender in HIV Policy: Examining how gender shapes the dissemination of HIV policies in southern Malawi." Paper accepted for roundtable presentation. American Public Health Association. New Orleans.

2014 **Dovel, Kathryn**. "Gendered care: examining how clinic experiences influence HIV testing decisions among STI patients in southern Malawi." Paper accepted for oral presentation. National Women's Studies Association, San Juan, Puerto Rico.

2013 **Dovel, Kathryn**. "HIV policies and their influence on men's use of care." Paper accepted for oral presentation. International HIV Social Science and Humanities Conference, Paris, France.

2007 **Dovel, Kathryn**. "Social and structural impediments that limit proper healthcare in rural southern Kurdistan." Paper accepted for oral presentation. The Anthropology and Sociology Research Conference, Santa Clara, CA.

INVITED PRESENTATIONS

2019 "Index HIV Self-Testing in Malawi". World Health Organization webinar

2019 "Men's (lack of) access to the health system". UNAIDS. Regional meeting on Accelerating Men's HIV service delivery and uptake in Eastern and Southern Africa.

2019 "Index HIVST in Malawi: a Randomized Control Trial. World Health Organization. Webinar

2019 "*Reaching men and engaging them in HIV care – lessons from Malawi*". Men and HIV forum. International AIDS Society. Mexico City, Mexico

2018 "The impact of HIV self-testing on HIV testing among outpatients in high burden facilities in Malawi: preliminary findings from a cluster randomized control trial" USAID Washington. Washington D.C

2017 "Who benefits from Test and Treat? Understanding gender dimensions of universal treatment policies and gender-specific barriers to care" Malawi Ministry of Health, HIV Treatment Technical Working Group. Lilongwe, Malawi.

2016 "Facility-based barriers to HIV testing among men in Malawi: a systems approach" Malawi Ministry of Health, HIV Treatment Technical Working Group. Lilongwe, Malawi.

2015 "Men's heightened risk of AIDS-related death: the legacy of gendered HIV testing and treatment strategies" United Nations Meeting on Male Engagement. Geneva, Switzerland

2015 "Facility-based barriers to men's use of HIV testing: recommendations for male engagement guidelines." National AIDS Control Council Meeting for the Development of the Male

Engagement Guidelines. Nairobi, Kenya

2014 “Gendered care: examining who ‘does gender’ in clinical settings and its influence on HIV services for men in southern Malawi.” Health Working Group, UCLA. Los Angeles, CA

2013 “From questions to methods: mixed methods approach to disparities research.” Course in Qualitative Methods (Doctoral Students). University of Colorado Denver. Denver, C

2010 “Lost in translation: examples of why best-practice nutrition programs fail in rural Dominican Republic.” Drabkin and Bixby International Conference, UCLA. Los Angeles, CA

RELEVANT EMPLOYMENT ACTIVITIES

2015- Consultant for Mixed Methods, Invest in Knowledge, Zomba, Malawi

Activities: Oversaw data analysis and write-up for studies implemented by Invest in Knowledge. I focused on qualitative and mixed methods analysis and write-up.

2009-10 Research Specialist, Korean Resource Center, Los Angeles, CA

Activities: Managed data entry and data cleaning and led in data analysis and write-up of a study assessing use of non-communicable disease services among first- and second-generation Korean populations in Los Angeles.

2009 Program Evaluation Fellow, Bataye Relief Alliance, Santo Domingo, Dominican Republic

Activities: Led the assessment of a nutritional program aimed to improve child health outcomes in Haitian populated bateyes in Dominican Republic. I led tool development, training enumerators, data analysis, and write-up

2007-08 Program Coordinator, Orange County Department of Public Health, Santa Ana, CA

Activities: Conducted literature reviews and assisting in the development of interventions to address Alcohol and Drug abuse among young adults in Orange County. Assisted in the protocol development and implementation of interventions.

SERVICE

2019 Committee Member of the Men’s HIV Forum at the International AIDS Conference, Mexico City

2018- Member of the Malawi Ministry of Health HIV Self-Testing Guidelines Task Force

2017- Member of the Malawi Ministry of Health HIV Testing Services Technical Working Group

2017- Member of the EQUIP HIV Self-Testing Technical Working Group

2016- Member of the UNAIDS Working Group “Engaging men in solutions for the HIV epidemic: Health systems.”

2016- Member of the “Men and HIV Global Working Group”

2016 Reviewer for the APHA 2016 Annual Meeting & Expo

2012-13 Editor of the Health and Behavioral Sciences Peer-Reviewed Journal, University of Colorado, Denver

2011-12 Student Advisory Council Member, University of Colorado, Denver

For peer review only

COURSES TAUGHT

Adjunct Professor

Social determinants of health in the context of HIV services in sub-Saharan Africa – Field Rotation Series (UCLA)

Health, Disease & Globalization: Foundations of Epidemiology (Vanguard University)

Human Sexuality (co-taught, Vanguard University)

Cultural Anthropology (Vanguard University)

Applied Anthropology (Vanguard University)

Qualitative Methods (Vanguard University)

Teaching Assistant

AIDS and Other Sexually Transmitted Diseases (UCLA)

Global Health Issues (UCLA)

Social Determinants of Health (University of Colorado Denver)

Statistical Analysis (University of Colorado Denver)

MENTORSHIP

University of California Los Angeles. David Geffen School of Medicine. Medical Student. Kate Coursey. "Examining characteristics of women who engage in an integrated Early Childhood Development and PMTCT program in Malawi: endline evaluation." 2019-

University of California Los Angeles. David Geffen School of Medicine. Medical Student. "Provider acceptability of interventions to increase ART initiation among men who test HIV-positive through index HIV self-testing." 2019-

University of California Los Angeles. David Geffen School of Medicine. Medical Student. Tijana Temelkovska. "Examining the successes and challenges of implementing an early childhood development intervention with HIV-positive women in Malawi: a process evaluation." 2018-

University of California Los Angeles. Internal Medicine Residency, Global Health Track. Resident Physician. Marguerite Thorp. "Can a brief screening tool identify ART clients at risk of defaulting from treatment? a prospective study in Malawi." 2018-

University of California Los Angeles. Internal Medicine Residency, Global Health Track. Resident Physician. Adrian Mayo. "Predictors of early ART retention among adults who initiated under Universal Treatment policies in Malawi." 2018-

University of Colorado Denver. Health and Behavioral Sciences. Doctoral Student. Stephanie Chamberlin. "Exploring the association between education and ART retention in rural Malawi." 2017-

University of California Los Angeles. Fogarty GloCal Fellow. Medical Student. Ogechukwu Offorjebe. "Examining the feasibility and acceptability of HIV self-test kits for index testing among HIV+ clients and their partners in Malawi: A mixed methods study." 2017-18

College of Medicine, Malawi. MPH Student. Khumbo Phiri. "The role of lay cadre in ART initiation and retention under Test and Treat in Malawi." 2017-18

For peer review only

Brandeis University. Elisa Morales, Becca Sliwosk, and Melanie Morris (capstone project). “Developing a funding proposal for Men-to-Men, a gender-transformation and income-generating program for men in northern Uganda.” 2015 (with 31Bits International)

Vanguard University. Medical Anthropology Honors Thesis. Joanna Takegami. “Barriers to Women’s use of Antiretroviral Therapy in Northern Uganda: Exploring the Role of Structural Violence.” 2011

AD HOC REVIEWER

AIDS, JAIDS, JIAS, BMC Public Health, Global Health Action, Culture, Health and Sexuality

PROFESSIONAL MEMBERSHIPS

Member, American Public Health Association (APHA), Present

Member, American Sociology Association (ASA), Present

Member, American Anthropological Association (AAA), Present

For peer review only

OMB No. 0925-0001 and 0925-0002 (Rev. 10/15 Approved Through 10/31/2018)

THOMAS J. COATES

eRA COMMONS USER NAME (credential, e.g., agency login): **TCOATS**

POSITION TITLE: **Professor Emeritus, Division of Infectious Diseases, Department of Medicine UCLA David Geffen School of Medicine**

EDUCATION/TRAINING *(Begin with baccalaureate or other initial professional education, such as nursing, include postdoctoral training and residency training if applicable. Add/delete rows as necessary.)*

INSTITUTION AND LOCATION	DEGREE *(if applicable)*	Completion Date MM/YYYY	FIELD OF STUDY
San Luis Rey College, San Luis Rey, California	BA	06/1968	Philosophy
San Jose State University, San Jose, California	MA	01/1971	Psychology
Stanford University, Stanford, California	PhD	06/1977	Counseling Psychology

A. Personal Statement

I am Director of the system-wide University of California Global Health Institute (founded in 2008) and was the Founding Director of the UCLA Center for World Health (founded in 2012) until 2018. In 1986 I co-founded the Center for AIDS Prevention Studies (CAPS) at UCSF and directed it from 1991 to 2003. I was also the founding Director of the UCSF AIDS Research Institute, leading it from 1996 to 2003.

I have substantial expertise in research on HIV prevention among heterosexual men and women in the HIV epidemic in sub-Saharan Africa and in the HIV testing and treatment trials in sub-Saharan Africa, especially Malawi through PEPFAR funding. As Distinguished Research Professor of Medicine, I continue with two NIH and two foundation grants focused in southern Africa. I also continue as a co-investigator on the UCLA-based Center for HIV Identification, Prevention and Treatment Studies (CHIPTS).

I have had extensive experience with large-scale, community-based, multi-site research and implementation projects spanning HIV prevention, care and treatment, and policy. I currently have funding to test and evaluate innovative strategies for bring men in South Africa into HIV testing and treatment, as well as for providing early childhood development training for HIV-infected mothers and their babies in Malawi through support from the Conrad N. Hilton Foundation. We are also in the first year of a 5-year NIH-funded grant to study pre-exposure prophylaxis for pregnant and post-partum women in South Africa.

B. Positions and Honors

For peer review only

1984 - 2003	Member, Medical Attending Staff, UCSF Hospitals and Clinics
1990 - 2003	Professor, Department of Medicine, UCSF
1991 - 2003	Director, Center for AIDS Prevention Studies, UCSF
1996 - 2003	Director, AIDS Research Institute, UCSF
2000	Elected to the Institute of Medicine (now the National Academy of Medicine)
2010 - 2014	Member, Institute of Medicine Board on Global Health
2003 - 2006	Professor Step VII, Division of Infectious Diseases, Department of Medicine, David Geffen School of Medicine, UCLA
2003 - Present	Joint Appointment, Department of Medicine, UCSF; Member, Executive Committee, UCLA AIDS Institute
2003 - 2011	Director, UCLA Program in Global Health
2004 - Present	Joint Appointment, Department of Epidemiology, UCLA School of Public Health
2006 – 2009	Professor Step IX, Division of Infectious Diseases, Department of Medicine, David Geffen School of Medicine, UCLA
2006 - 2018	Michael & Sue Steinberg Endowed Professor of Global AIDS Research, Division of Infectious Diseases, Department of Medicine, David Geffen School of Medicine, UCLA
2006 - 2018	Director, Global Capacity Building Core Center for HIV Identification, Prevention, and Treatment Services, UCLA Semel Neurosciences Institute
2006 - 2018	Associate Director for International and Policy Research UCLA AIDS Institute
2009 - 2016	Co-director, University of California Global Health Institute
2009 –2018	Distinguished Professor, Division of Infectious Diseases, Department of Medicine, David Geffen School of Medicine, University of California, Los Angeles
2011 – 2018	Director, UCLA Center for World Health at the David Geffen School of Medicine and UCLA Health
2016-Present	Director, University of California Global Health Institute
2018-Present	Distinguished Research Professor, Division of Infectious Diseases, UCLA David Geffen School of Medicine

C. Contribution to Science

1. **Combination HIV Prevention including Pre-Exposure Prophylaxis:** I have written extensively and conducted research on combination HIV prevention for MSM in the United States and Latin America and with a variety of populations in sub-Saharan Africa. My writing and research have been influential in shaping thinking about combination prevention, and in demonstrating the importance of considering HIV prevention as a combination of factors, as opposed to any single kind of program.

Joseph Davey D, Bekker LG, Gorbach P, **Coates T**, Myer L. Delivering PrEP to pregnant and breastfeeding women in sub-Saharan africa: The implementation science frontier. AIDS. 2017 Jul 18. doi: 10.1097/QAD.0000000000001604. PubMed PMID: 28723709.

For peer review only

Richter L, Komárek A, Desmond C, Celentano D, Morin S, Sweat M, Chariyalertsak S, Chingono A, Gray G, Mbwambo J, **Coates T**; Reported physical and sexual abuse in childhood and adult HIV risk behaviour in three African countries: findings from Project Accept (HPTN-043). AIDS and behavior. 2014; 18(2):381-9. PMCID: PMC3796176

Coates TJ; An expanded behavioral paradigm for prevention and treatment of HIV-1 infection. Journal of acquired immune deficiency syndromes (1999). 2013; 63 Suppl 2:S179-82. PMCID: PMC3943341

Coates TJ, Richter L, Caceres C. Behavioural strategies to reduce HIV transmission: how to make them work better. Lancet. 2008; 372(9639):669-84. PMCID: PMC2702246

2. **HIV Counseling and Testing (HTC):** I have conducted many significant and influential studies in HTC, beginning first with observational studies of the effect of HTC on risk behavior among men who have sex with men (MSM) in San Francisco. I was Principal Investigator for the first randomized controlled trial of HTC in Eastern Africa and the Caribbean, examining the effect of HTC on individual males and females, as well as couples presenting for HTC in Kenya, Tanzania, and Trinidad and Tobago, and these results were reported in *The Lancet* in 2000. I was the Principal Investigator for Project Accept, a cluster randomized trial conducted in South Africa, Zimbabwe, Tanzania, and Thailand, and these results were reported in *Lancet Global Health* in 2015. I also was the Principal Investigator of a randomized trial at Mulago Hospital in Uganda examining the effect of short vs. elaborated counseling on males and females presenting for care, and these results were reported in *Lancet Global Health* in 2014.

Coates TJ, Kulich M, Celentano DD, Zelaya CE, Chariyalertsak S, Chingono A, Gray G, Mbwambo JK, Morin SF, Richter L, Sweat M, van Rooyen H, McGrath N, Fiamma A, Laeyendecker O, Piwowar-Manning E, Szekeres G, Donnell D, Eshleman SH; NIMH Project Accept (HPTN 043) study team; Effect of community-based voluntary counselling and testing on HIV incidence and social and behavioural outcomes (NIMH Project Accept; HPTN 043): a cluster-randomised trial. The Lancet. Global Health. 2014; 2(5):e267-77. PMCID: PMC4131207

van Rooyen H1, McGrath N, Chirowodza A, Joseph P, Fiamma A, Gray G, Richter L, **Coates T**. Mobile VCT: reaching men and young people in urban and rural South African pilot studies (NIMH Project Accept, HPTN 043). AIDS and behavior. 2013; 17(9):2946-53. PMCID: PMC3597746

Wanyenze RK, Kamya MR, Fatch R, Mayanja-Kizza H, Baveewo S, Szekeres G, Bangsberg DR, **Coates T**, Hahn JA; Abbreviated HIV counselling and testing and enhanced referral to care in Uganda: a factorial randomised controlled trial. The Lancet. Global Health. 2013; 1(3):e137-45. PMCID: PMC4129546

Mhlongo S, Dietrich J, Otwombe KN, Robertson G, **Coates TJ**, Gray G.Factors associated with not testing for HIV and consistent condom use among men in Soweto, South Africa. PloS one. 2013; 8(5):e62637. PMCID: PMC3656000

3. **Global Health:** I have contributed to the literature on global health, especially from the perspective of engaging multiple disciplinary perspectives to attend to a variety of global health issues around the world.

For peer review only

Debas HT, **Coates TJ**; The University of California Global Health Institute opportunities and challenges. Infectious disease clinics of North America. 2011; 25(3):499-509, vii. PubMed [journal]PMID: 21896355

Duber HC, **Coates TJ**, Szekeras G, Kaji AH, Lewis RJ; Is there an association between PEPFAR funding and improvement in national health indicators in Africa? A retrospective study. Journal of the International AIDS Society. 2010; 13:21. PMCID: PMC2895577

Maman S, Abler L, Parker L, Lane T, Chirowodza A, Ntogwisangu J, Srirak N, Modiba P, Murima O, Fritz K.A comparison of HIV stigma and discrimination in five international sites: the influence of care and treatment resources in high prevalence settings. Social science & medicine (1982). 2009; 68(12):2271-8. PMCID: PMC2696587

Collins C, Coates TJ, Szekeres G; Accountability in the global response to HIV: measuring progress, driving change. AIDS (London, England). 2008; 22 Suppl 2:S105-111. PMCID: PMC2879260

Complete List of Published Work in MyBibliography:
http://www.ncbi.nlm.nih.gov/sites/myncbi/thomas.coates.1/bibliography/40839346/public/?sort=date&direction=descending

D. Research Support

Ongoing Research Support

R01MH105534-01A1 (Coates) 07/07/15 – 04/30/20
NIH/NIMH
Bringing South African Men into HIV Counseling and Testing (HCT) and Care
The objective of this project is to provide evidence-based strategies to improve treatment of HIV+ men through a three-step process: (1) Testing a significant proportion of the population, (2) linkage to care, and (3) maintaining in care a significant proportion of HIV+ individuals to the point of viral suppression. My role is as the Principal Investigator.

UM1 AI068619 (El Sadr) 07/01/14 – 11/30/20
Family Health International
NIH-NIAID
HIV Prevention Trials Network (HPTN) Leadership Group
The goals of this project are: 1) to develop the HPTN research agenda; 2) to review SWG research plans; 3) to review and approve concept plans; 4) to oversee the discretionary fund; 5) to review and revise HPTN policies and procedures; and 6) to evaluate the performance of the HPTN. My role is as Chair of the Manuscript Review Committee

P30 MH058107 (Shoptaw) 03/01/2017-02/28/2022
NIMH/NIH

Center for HIV Identification, Prevention, and Treatment Services

This project is a P30 and provides center grant services to HIV investigators at UCLA. I am a Co-Investigator in this center.

The Conrad N. Hilton Foundation 01/01/2018-12/31/2020

Delivery of Childhood Development Services as Part of HIV Treatment Services in Malawi

This grant supports the integration of early childhood development services within pre- and post-natal care for HIV+ mothers in Malawi.

R01 MH116771-01A1 09/30/2018-09-29-2023

NIMH/NIH

Evaluating the Prep-PP Cascade in HIV-negative Pregnant and Breastfeeding Women in South Africa.

The goal of this project is to test innovative models for delivering PrEP to pregnant and breastfeeding women age 16 and above in South Africa.

Entertainment Industry Foundation-Charlize Theron Africa Outreach Project 06/01/2018-05/30/2021

The goal of this project is to create a Youth Leaders Scholarship Fund to support promising young South Africans to attend South African tertiary education institutions.

Bill and Melinda Gates Foundation **(**Dovel) 12/4/2019-12/3/2023

Identifying Effective Linkage Strategies for HIVST (IDEaL)

This grant tests the effect of a staged intervention for ART initiation among men in Malawi, whereby additional interventions are added each month for individuals who have not yet initiated ART.

Completed Research Support

20150025 (Coates) 09/01/15 – 08/31/17
Conrad N. Hilton Foundation
Delivery of Early Childhood Development Services as a Part of HIV Treatment Services in Malawi
Pilot grant to assess the feasibility and acceptability, as well as initial outcomes, of supporting Option B+ mothers in Malawi to increase their responsiveness to their children and have positive impacts on early childhood development (ECD).

P30 MH58107 (Rotheram-Borus) 02/01/07 - 01/31/17

NIH/NIMH

Center for HIV Identification, Prevention, and Treatment Services (CHIPTS)

The mission of the Center for HIV Identification, Prevention, and Treatment Services (CHIPTS) is to promote collaborative research and education on effective HIV detection, prevention, and treatment programs for HIV at the societal, community, provider, and individual levels. My role is as the Director for International Care.

For peer review only

For peer review only

RISA MICHELLE HOFFMAN

CURRICULUM VITAE

PERSONAL HISTORY

David Geffen School of Medicine at UCLA
Division of Infectious Diseases
10833 Le Conte Ave 37-121 CHS
Los Angeles, CA 90095
rhoffman@mednet.ucla.edu
Tele: (310) 825-7225
Fax: (310) 825-3632

EDUCATION

Stanford University 1994, BA
University of California Los Angeles 2000, MD
Harvard School of Public Health 2000, MPH
Internship 2000-2001: Harvard Combined Medicine/Pediatrics Residency Program
Residency 2001-2004: Harvard Combined Medicine/Pediatrics Residency Program
Fellowship Infectious Diseases: 2005-2008: University of California, Los Angeles

LICENSURE

California, A85173, 01/31/2021

BOARD CERTIFICATION/OTHER CERTIFICATION

2004 & 2014 American Board of Internal Medicine
2007 & 2017 American Board of Internal Medicine, Infectious Diseases
2005 Certification in Travel Medicine from the London School of Hygiene and Tropical Medicine

PROFESSIONAL EXPERIENCE

Present Position

2016-present	Associate Clinical Professor, Division of Infectious Diseases, UCLA
2010-2016	Assistant Clinical Professor, Division of Infectious Diseases, UCLA Medical Center, Los Angeles, California
2008-2010	Clinical Instructor, Division of Infectious Diseases, UCLA Medical Center, Los Angeles, California

Previous Positions

2005-2008	Fellow in Infectious Diseases, UCLA Medical Center, Los Angeles, California
2001-2004	Resident Physician, Internal Medicine, Brigham and Women's Hospital, Boston, Massachusetts
2001-2004	Resident Physician, Pediatrics, Boston Children's Hospital and Massachusetts General Hospital, Boston, Massachusetts
2000-2001	Intern, Internal Medicine, Brigham and Women's Hospital, Boston, Massachusetts
2000-2001	Intern, Pediatrics, Boston Children's Hospital and Massachusetts General Hospital, Boston, Massachusetts

For peer review only

PROFESSIONAL ACTIVITIES & MEMBERSHIPS

2018-present	Interim Director, Global Health Education and Research Program, David Geffen School of Medicine at UCLA
2016-present	Co-Director UCLA AIDS Institute/CFAR International Health Services and Policy Research Program Section
2015-present	Associate Program Director, UCLA Infectious Diseases Fellowship Training Program
2013-present	Advisory Board Member for the University of California Global Health Institute GloCal Health Fellowship
2009-present	Research Co-Director, Partners in Hope Malawi and UCLA Research Collaboration
2009-present	Investigator, AIDS Clinical Trials Group (ACTG) and Maternal Child Adolescent Network (IMPAACT)
2009-present	HIV Clinical Consultant, To Help Everyone Clinic in Los Angeles, California
2009-present	Ad hoc Peer Reviewer (AIDS Care, International Journal of STD and AIDS, BMC Women's Health, American Society of Tropical Medicine and Hygiene, Journal of Infectious Diseases, International Health, JIAS)
2007-present	Member, Infectious Diseases Society of America (IDSA)
2016-2018	Committee Member, Antiretroviral Therapy Strategies (ARTs), AIDS Clinical Trials Group
2008-2016	Founder/Program Director, Sustainable Nutrition for Orphans and Vulnerable Children in Malawi, Central Africa: Provides education on nutrition and sustainable food sources for families caring for orphans in northern Malawi
2014-2016	Committee Lead, Infectious Diseases Quality Improvement M&M Program
2011-2016	Committee Member, AIDS Clinical Trials Group *Women's Health Inter-network Scientific Committee (WHISC)*
2007-2013	Founder/Program Co-Director, UCLA resident physician elective training program in Malawi, Africa
2010-2013	Co-Director, UCLA Program in Global Health and Global Health Education Program for the David Geffen School of Medicine at UCLA
2008-2012	Faculty for 'Multidisciplinary Approach to Global Health' elective course for first and second year medical students at UCLA
2007-2012	Committee Member, American Society of Tropical Medicine and Hygiene Education Committee
2005-2012	Advisory Board Member, UCLA Medicine/Pediatrics Residency Training Advisory Board
2005-2011	Interviewer, UCLA Medicine/Pediatrics Residency Training Program
2006-2008	Faculty Group Leader, Problem Based Learning Microbiology Block for second year medical students at UCLA
2006-2008	Creator/Organizer, UCLA Infectious Diseases Core Curriculum Program

HONORS AND AWARDS

2012	David Geffen School of Medicine Award for Excellence in Education
2011	Nomination for the Consortium of Universities for Global Health Early Career Award
2009	Nomination for UCLA Faculty Teaching Award

For peer review only

2007 Nomination for UCLA Fellow Teaching Award
2006 Nomination for UCLA Fellow Teaching Award

2000 Elected to the UCLA chapter of the Alpha Omega Alpha Honor Society
2000 Janet M. Glasgow Memorial Achievement Citation for Academic Achievement at the UCLA School of Medicine
2000 John M. Adams Award for Excellence in Pediatrics, UCLA School of Medicine
2000 Edith and Carl Lasky Memorial Award for Outstanding Research Achievement, UCLA School of Medicine
1999 Longmire Surgical Medal for outstanding performance in surgical clerkships, awarded by the Department of Surgery, UCLA School of Medicine
1999 Charles Wacker Summer Research Fellowship, UCLA School of Medicine
1994 Elected to the Stanford Chapter of Phi Beta Kappa
1994 Elected to the Stanford Cap and Gown Women's Honor Society
1994 Recipient of the Joshua Lederberg Award for Outstanding Academic Achievement in Human Biology

LECTURES AND PRESENTATIONS

"Management of Febrile Neutropenia" Presented as part of the UCLA Division of Infectious Diseases Core Curriculum Program. Los Angeles, California, November 2013

"Challenges and Successes of EQUIP Malawi" Presented at UCLA Infectious Diseases Grand Rounds. Los Angeles, California, April 2014

"Primary Care Issues in HIV Care" Presented at the UCLA Department of Medicine housestaff curriculum. Los Angeles, California, May 2014

"A Case of Multi-Class HIV Resistance" Presented at the UCLA HIV/Hepatitis C Case Conference Series, Los Angeles, California, May 2014

"Evidence Based Managed of Osteomyelitis" Presented at the UCLA Division of Infectious Diseases Case Conference Series, Los Angeles, California, June 2014

"Responding to Viral Load: A Primer for Malawi Clinical Mentors" Presented at a PEPFAR EQUIP Clinical Training Meeting in Malawi, Africa, January 2015

"Clinical Pearls in the Management of HIV/AIDS". Presented at the UCLA Internal Medicine Resident Core Curriculum Conference Series, Los Angeles, California, February 2015

"Quality Improvement in ID Care at UCLA: Lessons Learned from M&M" Presented at the UCLA Division of Infectious Diseases Core Conference Series, Los Angeles, California, April 2015

For peer review only

"Health & Safety Overseas: An orientation for medical students" Presented at the UCLA Global Health Education Medical Student Orientation Program, Los Angeles, California, April 2015

"ID Mimics". Presented at the UCLA ID Fellow Core Curriculum Series, Los Angeles, California, May 2015

"Quality Improvement on the Infectious Diseases Service: Transition of Care." Presented at the UCLA Division of Infectious Diseases Case Conference, Los Angeles, California, June 2015

"Quality Improvement on the Infectious Diseases Service: Notes and Documentation." Presented at the UCLA Division of Infectious Diseases Case Conference, Los Angeles, California, December 2015

"Quality Improvement on the Infectious Diseases Service: HIV Care". Presented at the UCLA Division of Infectious Diseases Case Conference, Los Angeles, California, February 2016

"Multi-month scripting to achieve improved outcomes in EQUIP". Presented at the EQUIP annual meeting, Johannesburg, South Africa March 2016

"Clinical Management of HIV/AIDS for the Primary Care Resident". Presented at the UCLA Internal Medicine Resident Core Curriculum Conference Series, Los Angeles, California, April 2016

"Update on Option B+ in Malawi". Presented to the Women's Health Committee of the AIDS Clinical Trials Group, Los Angeles, California, April 2016

EQUIP Malawi: A Partnership for HIV Care in Malawi. Presented at Harbor UCLA Infectious Diseases Grand Rounds, Los Angeles, California, July 2016

Speaker, Infectious Diseases Career Panel for Medical Students at the David Geffen School of Medicine. Los Angeles, September 2016

Systemwide Case Conference Faculty Discussant for the MultiCampus Infectious Diseases Fellowship Program. Presented at the VA Hospital, Los Angeles, California, December 2016

"Introduction to Global HIV Treatment in Resource Poor Settings," Lecturer for the UCLA School of Public Health, February 2018, Los Angeles

UCLA Division of Infectious Diseases, Journal Club Faculty Discussant, MDR TB Treatment, March 2018, Los Angeles

For peer review only

Faculty Panelist. Global Health Career Night for the David Geffen School of Medicine. November 2018, Los Angeles

West LA VA Internal Medicine Grand Rounds Speaker: "The Intersection of HIV and Non-Communicable Diseases in Resource-Limited Settings" April 2019, Los Angeles

"Qualitative Client and Provider Experiences with Multi-Dispensing for HIV in Malawi and Zambia". Presented as part of the CQUIN Consortium. Webinar, April 2019

"Introduction to the Global Health Program". Presented as part of the DGSOM Global Health Selective, September 2019, Los Angeles

PUBLICATION/BIBLIOGRAPHY

RESEARCH PAPERS

RESEARCH PAPERS (PEER REVIEWED)

Hoffman RM, Umeh OC, Garris C, Givens N, Currier JS. Evaluation of Sex Differences of Fosamprenavir (With and Without Ritonavir) in HIV-infected Men and Women. HIV Clin Trials. 2007;8(6):371-380.

Hoffman RM, AboulHosn J, Child JS, Pegues DA. Bartonella Endocarditis in Complex Congenital Heart Disease. Congenit Heart Dis. 2007;2(1):79-84.

Black V, **Hoffman RM**, Sugar CA, Menon P, Venter FWD, Currier JS, Rees H. Safety and Efficacy of Initiating Highly Active Antiretroviral Therapy in an Integrated Antenatal and HIV Clinic in Johannesburg, South Africa. J Acquir Immune Defic Syndr. 2008;49(3):276-81. PMC2893046.

Hoffman RM, Black V, Technau K, van der Merwe KJ, Currier JS, Coovadia A, Chersich M. Effects of Highly Active Antiretroviral Therapy Duration and Regimen on Risk for Mother-to-Child Transmission of HIV in Johannesburg, South Africa. J Acquir Immune Defic Syndr. 2010;54(1):35-41. PMC2880466.

Pilotto JH, Velasque L, Khalili R, Ismerio R, Veloso VG, Grinsztejn B, Morgado MG, Watts DH, Currier JS, **Hoffman RM**. Maternal Outcomes after HAART for Prevention of Mother-to-Child Transmission in HIV-infected Women in Brazil. Antivir Ther. 2011;16(3):349-56. PMC3437753.

Hoffman RM, Jamieson BD, Bosch RJ, Currier JS, Kitchen CMR, Schmid I, Zhu Y, Bennett K, Mitsuyasu R. Baseline Immune Phenotypes and CD4+ T Lymphocyte Responses to Antiretroviral

For peer review only

Therapy in Younger versus Older HIV-infected Individuals. J Clin Immunol. 2011;31(5):873-81. PMC3194061.

Van der Merwe J, Hoffman RM, Black V, Chersich M, Coovadia A, Rees H. Birth outcomes in South African Women Receiving Highly Active Antiretroviral Therapy: a Retrospective Observational Study. J Int AIDS Soc. 2011;14:42. PMC3163172.

Mindry D, Wagner G, Lake JE, Smith A, Linnemayr S, Quinn M, **Hoffman RM**. Fertility Desires Among HIV-infected Men and Women in Los Angeles County: Client Needs and Provider Perspectives; Matern Child Health J. 2013 May;17(4):593-600. PMC N/A.

Burke Z, Chen J, Conceicao C, **Hoffman R**, Miller L, Taela A, DeUgarte DA. Evaluation of Preoperative and Intraoperative RBC Transfusion Practices in Maputo Central Hospital, Mozambique. Transfusion. 2013 May 21. doi: 10.1111/trf.12252. PMC3751985.

Hoffman RM, Leister E, Kacanek D, Shapiro DE, Read JS, Bryson Y, Currier JS. Biomarkers from late pregnancy to six weeks postpartum in HIV-infected women who continue versus discontinue antiretroviral therapy after delivery. JAIDS. 2013 May 8. PMC3868443.

Jaganath D, Mulenga C, **Hoffman R**, Hamilton J, Boneh G. This is My Story: Participatory Performance for HIV and AIDS Education at the University of Malawi. Health Education Research. 2013 Sep 18. PMC4155417.

Kawale P, Mindry D, Stramotas S, Chilikoh P, Phoya A, Henry K, Elashoff D, Jansen P, **Hoffman R**. Factors associated with desire for children among HIV-infected women and men: A quantitative and qualitative analysis from Malawi and implications for the delivery of safer conception counseling. AIDS Care. 2013 Jun;26(6). PMC3943633.

1. Iroezi N, Mindry D, Kawale P, Chikowi G, Jansen P, **Hoffman R**. A qualitative analysis of the barriers and facilitators to receiving care in a prevention of mother-to-child program in Nkhoma, Malawi. Afr JReprod Health. 2013 Dec;17(4). PMC4361063.

2. Russell E, Mohammed T, Smeaton L, Jorowe B, MacLeod I, **Hoffman R**, Currier JS, Moyo S, Essex M, Lockman S. Immune activation markers in peripartum women in Botswana: association with feeding strategy and maternal morbidity. PLoS One. 2014 Mar 21. PMC3962339.

3. Reddy D, Njala J, Stocker P, Schooley A, Flores M, Tseng C-H, Pfaff C, Jansen P, Mitsuyasu RT, **Hoffman RM**. High-risk human papillomavirus in HIV-infected women undergoing cervical cancer screening in Lilongwe, Malawi: A pilot study. International Journal of STDS and AIDS, 2014 Jun 13. PMC4363075.

4. Kamuyango A, Hirschhorn L, Wang W, Jansen P, **Hoffman R**. One-Year Outcomes of Women Started on Antiretroviral Therapy during Pregnancy before and after the Implementation of Option B+ in Malawi: A Retrospective Chart Review from Three Facilities. World Journal of AIDS. 2014 Sept;4(3). PMC4356991.

5. Shull H, Tymchuk C, Grogan T, Hamilton J, Friedman J, **Hoffman RM**. Evaluation of the UCLA Department of Medicine Malawi Global Health Clinical Elective: Lessons from the First Five Years. Am J Trop Med Hyg. 2014 Sep 15. PMC4228879.

For peer review only

6. Hoffman JC, Anton PA, Baldwin GC, Elliott J, Anisman-Posner D, Tanner K, Grogan T, Elashoff D, Sugar C, Yang OO, **Hoffman RM**. Seminal Plasma HIV-1 RNA Concentration is Strongly Associated with Altered Levels of Seminal Plasma Interferon Gamma, Interleukin-17, and Interleukin-5. AIDS Res Hum Retroviruses. 2014 Oct 2. PMC4208556.

7. Menon P, **Hoffman R**, Black V. Characteristics of HIV-infected women on antiretroviral therapy who develop preeclampsia in South Africa: A Case Series. The Journal of Global Health. 2014;4(2). PMC N/A.

8. Cheng Q, Engelage E, Grogan T, Currier JS, and **Hoffman RM**. Who provides primary care? A cross-sectional survey of HIV patients and providers in a Los Angeles clinic. AIDS Clinical Research. 27 Oct 2014;5(11). PMC4409003.

9. Kawale P, Mindry D, Phoya A, Jansen P**, Hoffman RM**. Provider attitudes about childbearing and knowledge of safer conception at two HIV clinics in Malawi. Reproductive Health. 2015 Mar 7;12(1):17. PMC4355153.

10. Yeatman S, **Hoffman RM,** Chilungo A, Lungu S, Namadingo H, Chimwaza A, and Trinitapoli JA. Health-seeking behavior and symptoms associated with early HIV infection: Results from a population-based cohort in southern Malawi. JAIDS. 2015 May 1;69(1):126-30. PMC4422188.

11. **Hoffman RM**, Jaycocks A, Vardavas V, Wagner G, Lake JE, Mindry D, Currier JS, Landovitz R. Benefits of PrEP as an adjunctive method of HIV prevention during attempted conception between HIV-uninfected women and HIV-infected male partners. Journal of Infectious Diseases, J Infect Dis. 2015 Jun 19. PMC4621256.

12. Lake JE, **Hoffman RM**, Tseng CH, Wilhalme HM, Currier JS. Success of Standard Dose Vitamin D Supplementation in Treated HIV Infection. Open Forum Infect Dis. 2015 May 15;2(2). PMC4462892.

13. Chipungu C, Veltman JA, Jansen P, Chiliko P, Lossa C, Namarika D, Benner B, **Hoffman RM**, Bristow CC, Klausner JD. Feasibility and Acceptability of Cryptococcal Antigen Screening and Prevalence of Cryptocococcemia in Patients Attending a Resource-limited HIV/AIDS clinic in Malawi. Journal of the International Association of Providers of AIDS Care, J Int Assoc Provid AIDS Care. 2015 Jul 2. PMC N/A.

14. Coelho L, Cardoso SW, Luz PM, **Hoffman RM**, Mendonca L, Veloso VG, Currier JS, Grinsztejn B, Lake JE. Vitamin D3 supplementation in HIV infection: effectiveness and associations with antiretroviral therapy. Nutr J. 2015 Aug 18: 14. PMC4538921.

15. **Hoffman RM**, Lake JE, Wilhame H, Tseng CH, Currier JD. Vitamin D levels and markers of inflammation and metabolism in HIV-infected individuals on suppressive antiretroviral therapy. AIDS Res and Human Retroviruses 2016 March;32(3). PMC4779972.

16. Herbst de Cortina S, Arora G, Wells T, **Hoffman RM**. Evaluation of a Structured Predeparture Orientation at the David Geffen School of Medicine's Global Health Education Programs. Am J Trop Med Hyg. 2016 Mar 2;94(3). PMC4775891.

For peer review only

17. Jordan J, **Hoffman R**, Arora G, Coates W. Activated learning: providing structure in global health education at the David Geffen School of Medicine at the University of California, Los Angeles – a pilot study. BMC Medical Education. 2016 Feb 16. PMC4755030.

18. Wagner GJ, Linnemayr S, Ghosh-Dastidar B, Currier JD, **Hoffman R**, Schneider S. Supporting Treatment Adherence Readiness through Training (START) for patients with HIV on antiretroviral therapy: a study protocol for a randomized controlled trial. Trials. 2016 March 24;17(1). PMC4806419.

19. Arora G, Perkins K, **Hoffman RM**. Optimizing global health electives through partnerships: A pilot study of pediatric residents. Academic Pediatrics. 2015 Sept-Oct;15(5). PMC N/A.

20. Chien E, Phiri K, Schooley S, Chivwala M, Hamilton J, **Hoffman RM**. Successes and Challenges of HIV Mentoring in Malawi: The Mentee Perspective. PLoS One. 2016 Jun 18;11(6). PMC4924818.

21. Schooley A, Kamudumuli P, Vangala S, Tseng CH, Soko C, Parent J, Phiri K, Jahn A, Namarika D, **Hoffman RM**. CD4 Variability in Malawi: Implications for Use of a CD4 Threshold of 500 Cells/mm3 Versus Universal Eligibility for Antiretroviral Therapy. Open Forum Infectious Diseases. 2016 Aug 29;3(3). PMC5047419.

22. Hemarajata P, Baghdadi JD, **Hoffman R**, Humphries RM. Burkholderia pseudomallei: Challenges for the Clinical Microbiology Laboratory. J Clin Microbiol 2016 Dec;54(12):2866-2873. PMC5121373.

23. Pfaff C, Scott V, **Hoffman R**, Mwagomba B. You can treat my HIV – But can you treat my blood pressure? Availability of integrated HIV and non-communicable disease care in northern Malawi. Afr J Prim Health Care Fam Med. 2017 Feb 15;9(1):e1-e8. PMC5320467.

24. Gibb J, Chitsulo J, Chipungu C, Chivwara M, Schooley A, **Hoffman RM**. Supporting Quality Data Systems: Lessons Learned from Early Implementation of Routine Viral Load Monitoring at a Large Clinic in Lilongwe, Malawi. Clinical Research in HIV AIDS and Prevention. 2017 Mar 14;2(4). PMC5502771

25. Arora G and **Hoffman RM**. Development of an HIV Postexposure Prophylaxis (PEP) Protocol for Trainees Engaging in Academic Global Health Experiences. Acad Med. 2017 Apr 25. PMC28445222.

26. **Hoffman RM**, Phiri K, Parent J, Grotts J, Elashoff D, Kawale P, Yeatman S, Currier JS, Schooley A. Factors associated with retention in Option B+ in Malawi: a case control study. J Int AIDS Soc. 2017 Apr 27;20(1):1-8. PMC28453243.

27. Currier JS, Britto P, **Hoffman RM**, Brummel S, Masheto G, Joao E, Santos B, Aurpibul L, Losso M, Pierre MF, Weinberg A, Gnanashanmugam D, Chakhtoura N, Klingman K, Browning R, Coletti A, Mofenson L, Shapiro D, Pilotto J. Randomized Trial of Stopping or Continuing ART among Postpartum Women with Pre-ART CD4 ≥ 400 cells/mm3 (PROMISE 1077HS). PLoS One. 2017 May 10;12(5):e0176009. PMC28489856.

28. **Hoffman R**, Bardon A, Rosen S, Fox M, Kalua T, Xulu T, Taylor A, Sanne I. Varying intervals of antiretroviral medication dispensing to improve outcomes for HIV patients (The INTERVAL

For peer review only

Study): study protocol for a randomized controlled trial. Trials. 2017 Oct 13;18(1):476. PMC29029644.

29. Judson SD, LeBreton M, Fuller T, **Hoffman RM**, Njabo K, Brewer TF, Dibongue E, Diffo J, Kameni JF, Loul S, Nchinda GW, Njouom R, Nwobegahay J, Takuo JM, Torimiro JN, Wade A, Smith TB. Translating Predictions of Zoonotic Viruses for Policymakers. Ecohealth. 2017 Dec 11. PMC29230614.

30. Fatti G, Ngorima-Mabhena N, Chirowa F, Chirwa B, Takarinda K, Tafuma TA, Mahachi N, Chikodzore R, Nyadundu S, Ajayi CA, Mutasa-Apollo T, Mugurungi O, Mothibi E, **Hoffman RM**, Grimwood A. The effectiveness and cost-effectiveness of 3- vs. 6-monthly dispensing of antiretroviral treatment (ART) for stable HIV patients in community ART-refill groups in Zimbabwe: study protocol for a pragmatic, cluster-randomized trial. Trials. 2018 Jan 29;19(1):79. PMC29378662.

31. Caplan MR, Phiri K, Parent J, Phoya A, Schooley A, **Hoffman RM**. Provider Perspectives on Barriers to Reproductive Health Services for HIV-Infected Clients in Central Malawi. Clinical Obstetrics, Gynecology and Reproductive Medicine. 2018 Feb 12;4(1). PMC6391881.

32. Canan T, **Hoffman RM**, Schooley A, Boas Z, Schwab K, Kahn D, Shih R, Phiri K, Parent J, Banda BA, Chagoma R, Chipungu C, Pool K-L. Training Course in Focused Assessment with Sonography for HIV/TB in HIV Prevalent Medical Centers in Malawi. Journal of Global Radiology. 2018 March;4(1). PMC N/A.

33. Rough K, Seage GR, Williams PL, Hernandez-Diaz S, Huo Y, Chadwick EG, Currier JS, **Hoffman RM**, Barr E, Shapiro DE, Patel K; PHACS and the IMPAACT P1025 Study Teams. Birth Outcomes for Pregnant Women with HIV Using Tenofovir–Emtricitabine. New England Journal of Medicine. 2018 April 26;378(17):1593-1603. PMC5984044

34. Dovel K, Shaba F, Nyirenda M, Offorjebe OA, Balakasi K, Phiri K, Nichols B, Tseng C-H, Bardon A, Ngona K, **Hoffman R**. Evaluating the Integration of HIV Self-Testing Into Low-Resource Health Systems: Study Protocol for a Cluster-Randomized Control Trial from EQUIP Innovations. Trials. 2018 Sept 17;19(1). PMC6142354

35. Schwab K, **Hoffman RM**, Phiri L, Kahn D, Longwe L, Banda BA, Gama K, Chimombo M, Shih R, Schooley A, Pool K-L. Remote Training and Oversight of Sonography for Human Immunodeficiency Virus–Associated Tuberculosis in Malawi. Journal of the American College of Radiology. 2018 Sept 26. PMC6540803

36. Hubbard J, Kakwesa G, Nyirenda M, Mwambene J, Bardon A, Balakasi K, Dovel K, Kalua T, **Hoffman RM**. Towards the Third 90: Improving Viral Load Testing with a Simple Quality Improvement Program in Health Facilities in Malawi. Int Health. 2018 Nov 1. PMC Pending

37. Moucheraud C, Chasweka D, Nyirenda M, Schooley A, Dovel K, **Hoffman RM**. Simple Screening Tool to Help Identify High-Risk Children for Targeted HIV Testing in Malawian Inpatient Wards. J Acquir Immune Def Syndr. 2018 Nov 1; 79(3). PMC Pending

38. **Hoffman RM**, Brummel SS, Britto P, Pilotto JH, Masheto G, Aurpibul L, Joao E, Purswani MU, Buschur S, Flore Pierre M, Coletti A, Chakhtoura N, Klingman KL, Currier JS for the 1077HS

For peer review only

PROMISE Team. Adverse Pregnancy Outcomes among Women who Conceive on Antiretroviral Therapy. Clin Infect Dis 2018 June 1. PMC6321847

39. Nhassengo P, Cataldo F, Magaco A, **Hoffman RM**, Nerua L, Saide M, Cuco R, Hoek R, Mbofana F, Couto A, Gudo E, Chicumbe S, Dovel K. Barriers and facilitators to the uptake of Test and Treat in Mozambique: a qualitative study on patient and provider perceptions. PLOS One. 2018 Dec 26; 13(12). PMC6306230

40. Magaço A, Dovel K, Cataldo F, Nhassengo P, **Hoffman RM**, Nerua L, Tique J, Saide M, Couto A, Mbofana F, Gudo E, Cuco R, Chicumbe S. 'Good health' as a barrier and facilitator to ART initiation: a qualitative study in the era of test-and-treat in Mozambique. Culture, Health and Sexuality. 2018. PMC Pending

41. **Hoffman RM**, Konstantia A, Brummel S, Saidi F, Violari A, Dula D, Mave V, Fairlie L, Gerhard T, Kamateeka M, Chipato T, Chi B, Stranix-Chibanda L, Nematadzira T, Moodley D, Bhattacharya D, Gupta A, Coletti A, McIntyre JA, Klingman KL, Chakhtoura N, Shapiro DE, Folwer MG, Currier JS. Maternal health outcomes among HIV-infected breastfeeding women with high CD4 counts: results of a treatment strategy trial. HIV Clinical Trials 2018 Dec;19 (6). PMC6428202 [Available on 2019-12-01]

42. Murnane PM, Bacchetti P, Currier JS, Brummel S, Okochi H, Phung N, Louie A, Kuncze K, **Hoffman RM**, Nematadzira T, Soko DK, Owor M, Saidi F, Flynn PM, Fowler MG, Gandhi M. Tenofovir concentrations in hair strongly predict virologic suppression in breastfeeding women. AIDS 2019 Apr 22. [Epub ahead of print]. PMC Pending

43. Shaba F, Offorjebe OA, Phiri K, Lungu E, Kalande, P, Nyirenda M, Gupta S, **Hoffman RM**, Dovel K. Perceived acceptability of a facility-based HIV self-test intervention in outpatient waiting spaces among adult outpatients in Malawi: A formative study. J Acquir Immune Defic Syndr. 2019 Jul 1;81(3):e92-e94. PMC Pending

44. Weinberg A, Huo Y, Kacanek D, Patel K, Watts DH, Wara D, **Hoffman RM**, Klawitter J, Christians U; for IMPAACT P1025 Team. Markers of spontaneous preterm delivery in women living with HIV: relationship with protease inhibitors and Vitamin D. J Acquir Immune Defic Syndr. 2019 May 28. [Epub ahead of print]. PMC Pending

45. McBride K, Parent J, Mmanga K, Chivwala M, Nyirenda MH, Schooley A, Mwambene JB, Dovel K, Lungu E, Balakasi K, **Hoffman RM**, Moucheraud C. ART Adherence Among Malawian Youth Enrolled in Teen Clubs: A Retrospective Chart Review. AIDS Behav. 2019 July 10. PMC Pending

46. Le C, Britto P, Brummel SS, **Hoffman RM**, Li JZ, Flynn PM, Taha TE, Coletti A, Fowler MG, Bosch RJ, Gandhi RT, Klingman KL, McIntyre JA, Currier JS. Time to Viral Rebound and Safety after Antiretroviral Treatment Interruption in Postpartum Women Compared to Men. In Press AIDS 2019, August 1. PMC Pending

For peer review only

OMB No. 0925-0001 and 0925-0002 (Rev. 09/17 Approved Through 03/31/2020)

MICHAL KULICH

eRA COMMONS USER NAME (credential, e.g., agency login):

POSITION TITLE: Associate Professor of Statistics

EDUCATION/TRAINING *(Begin with baccalaureate or other initial professional education, such as nursing, include postdoctoral training and residency training if applicable. Add/delete rows as necessary.)*

INSTITUTION AND LOCATION	DEGREE *(if applicable)*	Completion Date MM/YYYY	FIELD OF STUDY
Charles University, Prague	M.S.	9/1991	Math. Statistics
Limburgs Universitair Centrum, Diepenbeek	M.S.	9/1992	Biostatistics
University of Washington, Seattle	M.S.	9/1995	Biostatistics
University of Washington, Seattle	Ph.D.	10/1997	Biostatistics

A. Personal Statement

I have an extensive past experience with design, conduct and analysis of clinical trials, especially community randomized trials, in the context of HIV prevention research. I served as the Lead Statistician for the Behavioral Working Group within the HPTN in 2000–2003 and as the Protocol Statistician and Steering Committee member for Project ACCEPT (HPTN043) in 2003-2013. I have been also participating in protocol review groups in the HPTN. I was involved in the design of HPTN043, development of data collection procedures, and development and application of data quality control measures. I am a coauthor of 7 research papers on methodology and results of HPTN043. Since 2015, I am a protocol statstician on another community-randomized trial, Zwakala Ndoda Study: Diagnosing, Linking and Maintaining Men in Antiretroviral Treatment in Vulindlela and Greater Edendale Area, KwaZulu-Natal. The current application builds on my past experience with HIV prevention trials.

B. Positions and Honors

Professional Positions

For peer review only

1998–2000 Dept. of Probability and Statistics, Charles University, Prague, Czech Rep., Assistant Professor
2000–2003 Dept. of Biostatistics, University of Washington, Seattle, Research Assistant Professor
2004–2009 Dept. of Probability and Statistics, Charles University, Prague, Czech Rep., Assistant Professor
2010–2013 Dept. of Probability and Statistics, Charles University, Prague, Czech Rep., Associate Professor
2014– Dept. of Probability and Statistics, Charles University, Prague, Czech Rep., Chair

Professional Memberships

1995- Member, American Statistical Association

1998- Member, Czech Statistical Society

2004- Member, International Biometric Society

2005- Member, International Society for Clinical Biostatistics

Honors

1995 Donovan J. Thompson Award, University of Washington, Seattle, WA.

1997 Best Written Paper, International Biometric Society, Park City, UT.

2001 Bolzano Award, Bernard Bolzano Foundation, Prague, Czech Republic.

C. Contributions to Science

Design and conduct of HIV prevention trials

I have an expertise in design, conduct and analysis of large randomized HIV prevention trials. I was a protocol statistician in Project ACCEPT (HPTN043), a community-randomized trial conducted in five African and Asian sites, with HIV incidence calculated from cross-sectional blood samples as the primary endpoint. I designed methods for obtaining population samples by household-probability sampling, participated in data verification, performed analyses and collaborated on publications.

Genberg, B., **Kulich, M.,** Kawichai, S., Modiba, P., Chingono, A., Kilonzo, G., Richter, L., Pettifor, A., Sweat, M. & Celentano, D. HIV risk behaviors in Sub-Saharan Africa and Northern Thailand: Baseline behavioral data from Project Accept. *Journal of AIDS* 2008, 49(3):309-319. PMID: 18845954

Sweat, M., Morin, S., Celentano, D., Mulawa, M., Singh, B., Mbwambo, J., Kawichai, S., Chingono, A., Khumalo-Sakutukwa, G., Gray, G., Richter, L., **Kulich, M.,** Sadowski, A., Coates, T., and the Project Accept study team. Community-based intervention to increase HIV testing and case detection in people aged 16-32 years in Tanzania, Zimbabwe, and Thailand (NIMH Project Accept, HPTN 043): a randomised study. *The Lancet Infectious Diseases* 2011, 11(7), 525-532. PMID: 21546309

Coates, T.J., **Kulich, M.**, Celentano, D.D., Zelaya, C.E., Chariyalertsak, S., Chingono, A., Gray, G., Mbwambo, J.K.K., Morin, S.F., Richter, L., Sweat, M., van Rooyen, H., McGrath, N., Fiamma, A.,

Laeyendecker, O., Piwowar-Manning, E., Szekeres, G., Donnell, D., Eshleman, S.H. (2014) Effect of community-based voluntary counselling and testing on HIV incidence and social and behavioural outcomes (NIMH Project Accept; HPTN 043): A cluster-randomised trial. *The Lancet Global Health* 2014, 2 (5), e267-e277. PMID: 25103167

Salazar-Austin, N., **Kulich, M.**, Chingono, A., Chariyalertsak, S., Srithanaviboonchai, K., Gray, G., Richter, L., van Rooyen, H., Morin, S., Sweat, M., Mbwambo, J., Szekeres, G., Coates, T., Celentano, D. (2017) Age-Related Differences in Socio-Demographic and Behavioral Determinants of HIV Testing and Counseling in HPTN 043/NIMH Project Accept. *AIDS and Behavior* 2018, 22(2) 569-579. PMID: 28589504

Methods for cross-sectional incidence estimation

I participated in the development of laboratory and statistical methods for estimating HIV incidence from cross-sectional blood samples. These methods were needed for successful evaluation of the primary outcome in Project ACCEPT.

Laeyendecker, O., Piwowar-Manning, E., Fiamma, A., **Kulich, M.**, Donnell, D., Bassuk, D., Mullis, C. E., Chin, C., Swanson, P., Hackett, Jr, J., Clarke, W., Marzinke, M., Szekeres, G., Gray, G., Richter, L., Alexandre, M. W., Chariyalertsak, S., Chingono, A., Celentano, D. D., Morin, S. F., Sweat, M., Coates, T., Eshleman, S. H. Estimation of HIV Incidence in a Large, Community-Based, Randomized Clinical Trial: NIMH Project Accept (HIV Prevention Trials Network 043), *PLoS ONE* 2013, 8:7, e68349. PMID: 23874597

Laeyendecker, O., **Kulich, M.**, Donnell, D., Komárek, A., Omelka, M., Mullis, C. E., Szekeres, G., Piwowar-Manning, E., Fiamma, A., Gray, R. H., Lutalo, T., Morrison, C. S., Salata, R. A., Chipato, T., Celum, C., Kahle, E. M., Taha, T. E., Kumwenda, N. I., Karim, Q. A., Naranbhai, V., Lingappa, J. R., Sweat, M. D., Coates, T., Eshleman, S. H. Development of Methods for Cross-Sectional HIV Incidence Estimation in a Large, Community Randomized Trial. *PLoS ONE* 2013, 8:11, e78818. PMID: 2423605

Fogel, J.M., Piwowar-Manning, E., Donohue, K., Cummings, V., Marzinke, M.A., Clarke, W., Breaud, A., Fiamma, A., Donnell, D., **Kulich, M.**, Mbwambo, J., Richter, L., Gray, G., Sweat, M., Coates, T., Eshleman, S. Determination of HIV status in African adults with discordant HIV rapid tests. *Journal of Acquired Immune Deficiency Syndromes* 2015, 69, 430-438. PMID: 25835607

Fogel, J.M., Clarke, W., **Kulich, M.**, Piwowar-Manning, E., Breaud, A., Olson, M.T., Marzinke, M.A., Laeyendecker O., Fiamma, A., Donnell, D., Mbwambo, J., Richter, L., Gray, G., Sweat, M., Coates, T.J., Eshleman, S.H. Antiretroviral drug use in a cross-sectional population survey in Africa: NIMH Project Accept (HPTN 043). *Journal of Acquired Immune Deficiency Syndromes* 2017, 74, 158-165. PMID: 27828875

For peer review only

AUGUSTINE T CHOKO

Institution: Malawi Liverpool Wellcome Trust Clinical Research Programme

General Medical Council (or equivalent) registration number N/A

Do you currently have personal medical malpractice insurance? (if so, name of insurer)

N/A

Project role

Principal Investigator

Qualifications

Degree	Year	Subject	Awarding Institution
PhD	2018	Epidemiology	LSHTM
MSc	2012	Epidemiology	LSHTM
BSc	2009	Statistics & Computing	University of Malawi

Positions held (last ten years)

Start	End	Organisation	Position title, brief description of responsibilities
**2020**	**2024**	Malawi Liverpool Wellcome Trust (MLW)	Wellcome Trust & National Institute for Health Research International Intermediate Fellow
2019	2020	MLW	Protocol Lead; leading design, implementation and write up of a complex primary health clinic randomized trial.
2015	2018	MLW	Wellcome Trust Fellow in Public Health and Tropical Medicine<br>▪ PhD student

For peer review only

2013	2015	MLW	Research Assistant (Epidemiology)<br>▪ Data analysis and publication
2012	2013	MLW	Trial Manager<br>▪ Leading implementation of a community-based cluster randomized trial (HIV/TB)
2009	2012	MLW	Data Manager/Statistician<br>▪ Designing and administering study databases<br>▪ Preparing data for analysis and data analysis

GCP training: course provider, date

NIDA Clinical Trials Network, Obtained 11 November 2016, expires 2019

Please list any institutions with which you are affiliated to

Malawi Liverpool Wellcome Trust Clinical Research Programme, London School of Hygiene & Tropical Medicine

Ethics training: details of training, course provider, date

Clinical trials experience: title of trial, role, dates (if multiple, restrict to most recent and most relevant)

Secondary distribution of HIV self-tests through antenatal and HIV testing services: a pragmatic cluster-randomized trial (STAR-ANC); 2018-2019.

Partner-Provided Self-Testing and Linkage (PASTAL) adaptive multi-arm multi-stage cluster randomized trial; 2016-2017.

Intensified HIV / TB prevention linking home-based HIV testing, including the option of self-testing, with HIV care: a cluster-randomised trial in Blantyre, Malaw, Research Fellow, 2011-2015.

Other professional experience relevant to role in project

Experience in handling and analyzing large epidemiological datasets.

Relevant recent publications (maximum 5)

1. **Choko AT**, Corbett EL, Stallard N, Maheswaran H, Lepine A, Johnson CC, Sakala D, Kalua T, Kumwenda M, Hayes R, Fielding K. Effect of HIV self-testing alone or with additional

For peer review only

interventions including financial incentives on linkage to care or prevention among male partners of antenatal care attendees in Malawi: An adaptive multi-arm multi-stage cluster randomised trial. *PLoS Med* 2019 Jan 2;16(1):e1002719.

2. **Choko AT**, Fielding K, Stallard N, et al. Investigating interventions to increase uptake of HIV testing and linkage into care or prevention for male partners of pregnant women in antenatal clinics in Blantyre, Malawi: study protocol for a cluster randomised trial. *Trials.* 2017;18(1):349.
3. **Choko AT**, Kumwenda MK, Johnson CC, et al. Acceptability of woman-delivered HIV self-testing to the male partner, and additional interventions: a qualitative study of antenatal care participants in Malawi. *Journal of the International AIDS Society.* 2017;20(1):21610.
4. **Choko AT**, MacPherson P, Webb EL, et al. Uptake, Accuracy, Safety, and Linkage into Care over Two Years of Promoting Annual Self-Testing for HIV in Blantyre, Malawi: A Community-Based Prospective Study. *PLoS medicine.* 2015;12(9):e1001873.
5. **Choko AT**, Desmond N, Webb EL, et al. The uptake and accuracy of oral kits for HIV self-testing in high HIV prevalence setting: a cross-sectional feasibility study in Blantyre, Malawi. *PLoS medicine.* 2011;8(10):e1001102.

For peer review only

Khumbo Phiri Nyirenda

CONTACTS
Partners in Hope
PO Box 302, Lilongwe.
Cell: 265882400721/ 265999 840 946
Email: khumbophiri@gmail.com

ACADEMIC QUALIFICATIONS

I. MPH, University of Malawi, College of medicine, anticipating graduation in 2020
II. BSOC, Economics, University of Malawi, Chancellor College- February, 2006
III. Malawi School Certificate of Education (MSCE) Phwezi Girls Sec School-June, 2000

COURSES/ TRAININGS

- Qualitative research synthesis, university of cape town, faculty of health sciences February 2020
- Qualitative data analysis university of cape town, faculty of health sciences January 2020
- Biomedical Researchers and Staff modules (CITI program _May 2016)
- Certificate of Attendance in Value chain analysis training by Ron Black from CNFA's farmer to farmer USAID funded program, Washington DC (February 23-27, 2009)
- Output marketing training in grain grading by CNFA/RUMARK facilitated by a North Carolina Agriculture Department officer, held at Natural Resources College (August 25 -28, 2009).
- Certificate of Attendance in a Leadership workshop facilitated by Engineers without Boarders (October 26 – 28, 2009).
- Training of trainers course in Business management and technical knowledge by COMESA's ACTESA and IFDC in Lusaka, Zambia (September 6-15, 2010)

WORK EXPERIENCE

PARTNERS IN HOPE
Position: Implementation Science Manager
Period: September 2017 to date
Summary:
The Projects Research Coordinator is responsible for overseeing and implementing all research related activities at Partners in Hope (PIH) and in all program-supported sites. He/she is in charge of monitoring and evaluating projects and ensuring that PIH is accountable to research donors. This person works hand-in-hand with the University of California in Los Angeles (UCLA), Partners in Hope (PIH), the Ministry of Health (MoH) and other partners.
Responsibilities

For peer review only

1. Overseeing and implementing all research projects at Partners in Hope and all EQUIP-supported sites

2. Research applications, Reviews and Reports.

- Oversee applications for ethical review for the Malawi NHSRC and/or COMREC, including initial and renewal applications, as well as closeout of completed projects.
- Serve as the first line of direct communication with the NHSRC and/or COMREC to advocate for submitted applications.
- Work with UCLA, PIH, MoH and other partners to ensure all research is performed to the highest ethical standards and that data is securely managed.
- Make sure appropriate reporting is provided to the governing bodies (final reports, publications, etc.).
- Oversee submission of abstracts to research meetings.

3. Monitoring, Evaluation and Accountability to Donors

- Monitor all research projects and develop donor communications in collaboration with senior leadership, especially the M&E Team.
- Ensure timely production and submission of donor experts.
- Participate in development of strategies for expansion of research.
- Ensure continuous evaluation of projects and staff, including hiring and regular appraisals.

Period: December 2012 to March 2016
Organization: Partners in Hope
Position: Research coordinator

Description

- Coordinates and administers research study associated activities. Assists in project planning and ensures that pre-established work scope, study protocol and regulatory (ethical review in Malawi and at UCLA) requirements are followed. Oversees and coordinates research staff. Develops and maintains record keeping systems and procedures. My job as Research Coordinator involves these main tasks
- Assistance developing research proposals, data collection forms, and spreadsheets for organization of data. Develops and maintains record keeping systems and procedures.

- Ensures the smooth and efficient day to day operation of research and data collection
- activities; acts as the primary administrative point of contact for EQUIP research staff.
- Supervision of team of research assistants
- Assistance with recruitment and coordination of research subjects as appropriate.
- Supervision and assistance with quality control Data.
- Monitors the progress of research activities; develops and maintains records of research activities and prepares periodic and ad hoc reports as required by investigators, administrators and funding agencies(USAID quarterly reports) and regulatory bodies (NHSRC,UCLA,IRB)

For peer review only

• Assistance with preparing ethical review applications (HSRC) including frequent communication with NHSRC about status of pending applications.

I. CARANA COOPERATION

Position: M&E/MIS Assistant
Period: November 2010–September 2011

Description
Market Linkages Initiative was a project funded by **USAID** and implemented by ACDI/VOCA and CARANA Corporation. The two key objectives of the project are to strengthen and expand grain bulking systems and to integrate farmers to national and regional markets. My job as an M&E/MIS Specialist involved these main tasks:

- Assisting the M&E specialist in tracking MLI indicators, collecting and verifying information and maintaining PMP reports, work plans and reports(weekly, monthly updates, quarterly and annual) for Malawi activities

- Administering data collection tools to GBC/VACs and capacity building of grantees to keep relevant records and generate M&E reports as stipulated by the Grant agreement.

- Collating , analyzing and reporting in usable forms all data collected form GBC/VAC

- Supporting in the coming up of GIS map for MLI supported GBCs and its associated VACs in Malawi

- Administering M&E data collection tools and supervising M&E data collectors and ensure quality data collection

- Maintaining records of all source documents from grantees and other sources including filled questionnaires and interview reports
- Keeping records of field trip reports and monitor and updating field trip tracker for Malawi based MLI staff
- Undertaking case studies and documenting most significant change stories for selected GBCs/VACs/Farmers to monitor impacts of MLI work

- Maintaining an up to date filing system including project photos

- Ensuring that quality control procedures are met in terms of market data.

- Facilitating dissemination of market information to farmers on a timely and reliable basis using the E-platform

- Providing technical assistance on the E- platform to strategic partners

- Working alongside the new company and assisting/participating in development and deployment team to design and roll out a web to phone MIS platform

- Conducting weekly data checks on approved prices inside E-platform's price flagging module

- Manage a user, market and commodities database

II. **CNFA/RUMARK**

Position: Monitoring and Evaluation Coordinator
Period: January 2009 to October 2010

CNFA/RUMARK implemented the Malawi Agrodealer Strengthening Program funded by AGRA. Its main objective was to develop rural-based, commercially-viable agrodealer networks and to work with agrodealers to improve the management, technical and financial capacity of their enterprises, thereby creating a rural market driven economic environment specifically designed to meet the unique needs of smallholder farmers. My position of as M&E coordinator involved the following tasks

- Monitoring progress of the project activities by doing surveys which included development of survey tools which mostly use participatory methods.

- Monitoring and evaluating the agrodealers performance in terms of sales as well as their financial status.

- Analysis of information on Agrodealer performance

- Verifying and identifying operational and potential agrodealers and recommending them for training to enable them get registered with CNFA

- Organizing promotional activities i.e. lottery competitions with the intention of creating customer database for surveys
- Playing a facilitating role in managing relationships between RUMARK and input supply companies to ensure cordial relationships and partnerships.
- Consolidating and analyzing results across CNFA's programs.

- Conducting training needs assessment for different categories of agrodealers to ensure equal treatment so that their specific needs are taken on board.

- Organizing the Agrodealers Annual Convention.

- Production of monthly as well as interim semi-annual reports for the Project

- Involved in advocating for policies which are conducive for agrodealers' business growth and sustainability through Private–public partnerships which involves working with various stakeholders including Government and civil society organizations.

Research Abstracts
Provider perspectives on barriers to reproductive health services for HIV-infected clients

For peer review only

in Central Malawi: Khumbo Phiri, Margaret R Caplan , Julie Parent , Ann Phoya , Alan Schooley, and Risa M. Hoffman, Poster presentation at Interest 2017, Malawi

Barriers to ART uptake experienced by healthy clients in Malawi under Test and Treat: Dovel, Kathryn, Khumbo Phiri, Alan Schooley, McDaphton Bellos, Esnart Sanudi, Denis Chasweka, Risa Hoffman, poster exhibition at the 9th IAS Conference on HIV Science (IAS 2017, in Paris, France, 23-26 July 2017 and Interest 2017 in Malawi).

Facility-level barriers to antiretroviral therapy experienced by men in Malawi: Dovel, Kathryn, Khumbo Phiri, Alan Schooley, Misheck Mphande, Mackenzie Chivwara, Risa Hoffman(poster presentation at interest 2017, Malawi)

Examining Malawi's Rollout of Universal Treatment: Policy Implementation and Provider Perceptions: Misheck Mphande, Khumbo Phiri, Mackenzie Chivwara, Mike Nyirenda, Alan Schooley, Rachel Thomas, Risa Hoffman, Kathryn Dovel, (Poster presentation at IAS 2017 in Paris, France)

Low rates of successful defaulter tracing and re-engagement in care in Option B+ women in Central Malawi. K. Phiri, J. Parent, T. Mulitswa, A. Schooley, R. Hoffman. Poster presentation at the International AIDS Society (IAS) conference (Durban, 2016).

The successes and Challenges of collaborating with Health Surveillance Assistants (HSAs) to trace Option B+ defaulters. Khumbo Phiri Nyirenda, Julie Parent, Risa Hoffman, Alan Schooley, Temwanani Mulitswa

The Option B+ cascade: Characterizing uptake and retention in a USAID-PEPFAR program in rural Malawi. Khumbo Phiri, Alan Schooley, Mackenzie Chivwala, Joseph Njala, Judy Currier, Andreas Jahn, Anteneh Worku, Perry Jansen, Risa Hoffman

Improvements and on-going challenges in exposed infants care at rural sites in Malawi. Alan Schooley, Khumbo Phiri, Mackenzie Chivwala, Peter Chilikoh, Antenneh worku, Risa Hoffman

Health Surveillance Assistants Can Successfully Perform Defaulter Tracing In Rural Malawi. Mackenzie Chivwala, Khumbo Phiri, Risa Hoffman, Jimmy Chitsulo, Alan Schooley

Assessing the Potential Impact of Health Surveillance Assistants on HIV Care At The Facility And Community Level. Mackenzie Chivwala, Khumbo Phiri, Weston Njamwaha, Peter Chilikoh, Risa Hoffman, Alan Schooley

Mentee Perspectives on Factors Associated with a Successful HIV Mentorship Program Mike Nyrienda, Chiulemu Kussen, Savior Mwandira, Khumbo Phiri, Chiukepo Longwe, Peter Chilikoh, Risa Hoffman, Weston Njamwaha, Alan Schooley

Rapid Rollout of Viral Load Testing at Rural Health Facilities in Malawi. Alan Schooley, Risa Hoffman, Mike Nyirenda, Savior Mwandira, Weston Njamwaha, Khumbo Phiri, Chifundo Chipungu, Mackenzie Chivwala, James Kandulu

Increased HIV testing after implementation of an innovative CD4 results reporting system in rural Malawi. Alan Schooley, Mackenzie Chivwala, Reynier Ter Haar, George Mtonga,

For peer review only

Doreen Suwande, Kelvin Rambiki, Chiulemu Kussen, John Hamilton, Khumbo Phiri, Peter Chilikoh, Risa Hoffman, Perry Jansen. Accepted for poster presentation at the 6th South African AIDS Conference, Durban, South Africa, 18-21 June 2013.

Barriers to Adherence to ART in the Prevention of Mother-to-Child Transmission of HIV: Option B+ in Nkhoma, Malawi. Paul Kawale, Alan Schooley, Virginia Tancioco, Danielle Wickman, Khumbo Phiri, Ella Bwanausi, Risa Hoffman. Accepted for poster presentation at the 6th South African AIDS Conference, Durban, South Africa, 18-21 June 2013.

MANUSCRIPTS ACCEPTED/PUBLISHED

Successes and Challenges of HIV Mentoring in Malawi: The Mentee Perspective. E. Chien, K. Phiri, A. Schooley, M. Chivwala, J. Hamilton, R. Hoffman. PLoS One. 2016 Jun;11(6).

CD4 variability in Malawian adults and implications for universal eligibility. A.L. Schooley, P.S. Kamudumuli, S. Vangala, C.H. Tseng, C. Soko, J. Parent, K. Phiri, A. Jahn, D. Namarika, R. Hoffman. Open Forum Infect Dis. 2016 Aug;3(3).

Provider perspectives on barriers to reproductive health services for HIV-infected clients in Central Malawi: Margaret R Caplan, Khumbo Phiri, Julie Parent, Ann Phoya , Alan Schooley, and Risa M. Hoffman, PLOS ONE.

Factors Associated with Retention in Option B+ in Malawi: A Case Control Study: Risa M. Hoffman, khumbo phiri, Julie parent, J Grotts D Elashoff, Paul Kawale, Sara Yeatman, J S Currier, A Schooley, JIAS.

Training Course in Focused Assessment with Sonography for HIV/TB in HIV Prevalent Medical Centers in Malawi: Timothy Canan, R Hoffman, Alan Schooley, Zachary Boas, Kristin Schwab, Daniel Kahn, Roger Shih, Khumbo Phiri, Julie Parent, Ben Allan Banda, Ronald Chagoma, Chifundo Chipungu. Kara-Lee Pool, Journal of Global Radiology

REFEREES

Risa Hoffman (MD), Assistant Clinical Professor**,** David Gaffen school of medicine, UCLA**,** RHoffman@mednet.ucla.edu

Rachel Sibande (PhD), Program Director, United Nations Foundation rsibande@unfoundation.org, +27670236497

Godfrey Chapola (PhD), Managing Director, RUMARK, P.O Box 31290, Lilongwe.

Cel: 0999792 070, gchapola@rumark.org

For peer review only

Julie Hubbard

94 Culford Road London N1 4HN
+44 7845 445338
jhubbard@mednet.ucla.edu

EDUCATION

London School of Hygiene and Tropical Medicine
MSc Control of Infectious Diseases

Seattle Pacific University
Bachelor of Arts: Sociology & Women's Studies
Cum Laude GPA: 3.74
Graduated July 2012

PROFESSIONAL EXPERIENCE

University of California Los Angeles (UCLA), March 2017- Current
Research Coordinator – 'INTERVAL' Study
Lilongwe, Malawi and Lusaka, Zambia

- Supervise data collection by study personnel across 15 health facilities in southern and central Malawi. Coordinate field supervision to ensure data quality. Work with Principle Investigator (PI) to develop operating procedures for study implementation. Provide leadership and technical support to Zambia study team.

Harvest India USA, January 2016 – March 2017
Director of Operations
Costa Mesa, California and Andhrah Pradesh, India

- Managed all aspects of operations to support, fundraise, and raise awareness for education and poverty alleviation initiatives amongst the Dalit, or 'untouchable', caste. Drafted and executed marketing campaigns to meet fundraising goals.

31 Bits International, December 2012 – February 2015
Director of Operations
Gulu, Northern Uganda

- Directed 160 beneficiaries and 6 Ugandan counselors in income generating projects. Developed and implemented in-depth monthly reports to evaluate income. Used data to identify hindrances to livelihood, such as domestic violence and HIV health complications. Organized necessary support through internal management or accessing external resources.

One Days Wages, March 2011- December 2012
Chief Grant Analyst
Seattle, Washington

- Generated extensive research on project proposals pertaining to the UN Millennium Development Goals and presented analyses for grant decisions.

Seattle Pacific University, September 2011- July 2012
Research Assistant
Seattle, Washington

- Edited, reviewed, and prepared research documents for Assistant Director of Women's Studies Program.

PUBLICATIONS AND PRESENTATIONS

For peer review only

Publications
Julie Hubbard, Gift Kakwesa, Mike Nyirenda, James Mwambene, Ashley Bardon, Kelvin Balakasi, Kathryn Dovel, Thokozani Kalua, Risa M Hoffman; Towards the third 90: improving viral load testing with a simple quality improvement program in health facilities in Malawi, International Health, , ihy083, https://doi.org/10.1093/inthealth/ihy083

Hubbard J, Moucheraud C, Lungu E, Bardon A, Balakasi K, Kakwesa G, Hoffman R "“I forget that I am a patient”: A qualitative assessment of 6 month dispensing of ART" (Under review)

Dovel K, Beagley M, Hubbard J, Orombi G, Thompson K "Including men without sidelining women: the feasibility of male involvement within a women's empowerment program in northern Uganda" (Under review)

Dovel K, Hubbard J, Phiri K. "Gender and HIV services: The role of gender norms on ART initiation among men and women in Malawi." (In preparation)

Peer reviewed poster presentations
"Gender and HIV services: The role of gender norms on ART initiation among men and women in Malawi." Women in Global Health Scientific Conference. New York, New York. April 2018

"Towards the third 90: improving viral load testing with a simple quality improvement program in health facilities in Malawi" International Aids Society (IAS) Conference, Amsterdam, Netherlands. July 2018

Presentations
"Innovations in differentiated service delivery: Six-month scripting lessons from Ethiopia, Malawi and Zambia" Colombia University Mailman School of Public Health. Webinar, April 2019

CERTIFICATIONS
Confronting Gender Based Violence: Global Lessons with Case Studies from India Certification Course
Coursera (Johns Hopkins University) - Online
October 2015

- Epidemiology of gender-based violence, clinical care issues and how to provide psychosocial support for victims.

FELLOWSHIP
Mennonite Central Committee
Community Development Associate, July-August 2011

- Rural and urban poverty field study in Recife, Brazil association under the direction of the Chair of the Sociology Department at Seattle Pacific University.

HONORS
Seattle Pacific University Deans Scholar, 2008-2012

For peer review only

BROOKE E. NICHOLS, PHD, MS

OFFICE ADDRESS: 801 Massachusetts Avenue

Crosstown Center, 3rd Floor, Room 304

Boston, MA, USA 02118

+1 617 358 2403

Email: brooken@bu.edu

ACADEMIC TRAINING:

2015	Ph.D.	Department of Viroscience, Erasmus Medical Center (Rotterdam, the Netherlands)<br>*Mathematical Modeling and Cost-Effectiveness of Antiretroviral-Based HIV-1 Prevention Strategies.*
2011	M.S.	School of Public Health & Health Science, University of Massachusetts, Amherst<br>(Amherst, MA, USA), Epidemiology
2009	B.A.	Mount Holyoke College (South Hadley, MA, USA), International Relations, *cum laude*

RESEARCH APPOINTMENTS:

2019 – Present	Assistant Professor	Department of Global Health, School of Public Health,<br>Boston University, Boston, MA
2018 – 2019	Instructor	Department of Global Health, School of Public Health,<br>Boston University, Boston, MA
2017 – 2018	Research Scientist	Department of Global Health, School of Public Health,

For peer review only

		Boston University, Boston, MA
2017 – Present	Principal Researcher	Health Economics & Epidemiology Research Office, Wits
		Health Consortium, Faculty of Health Sciences, University of
		Witwatersrand, Johannesburg, South Africa
2017 – Present	Researcher	Joint Faculty Appointment, School of Clinical Medicine,
		Faculty of Health Sciences, University of the Witwatersrand,
		Johannesburg, South Africa
2015 – 2016	Postdoctoral Fellow	Department of Viroscience, Erasmus Medical Center,
		Rotterdam, the Netherlands
2009 – 2010	Research Assistant	University of Massachusetts Amherst, School of Public Health
		& Health Sciences, Amherst, MA

OTHER RESEARCH EXPERIENCE:

2012 – 2014	Epidemiologist	Médicins Sans Frontières
		Amsterdam, the Netherlands.
		Project: *Spinal cord injury outcomes in Sri Lanka*
2008 – 2009	Researcher	Ministry of Health and Social Services, Lüderitz, Namibia
		Project: *Ecologic study on alcohol establishments and HIV prevalence*

PROFESSIONAL APPOINTMENTS:

2008 – 2010	Research Associate: Epidemiology	Environ Corporation, Amherst, MA, USA.

AWARDS AND HONOURS:

2019	**Mary Lyon Award.** Mount Holyoke College Alumni Association. Award honors an alumna who

For peer review only

has demonstrated sustained achievement in her life and career consistent with the humane values

that Mary Lyon exemplified and inspired in others.

CONFERENCE ORAL PRESENTATIONS:

*Denotes graduate student or mentee

1. Popping S*, Kall M, Stempher E, Versteegh L, **Nichols B**, van Sighem A, van de Vijver D, Boucher C, Verbon A, Delpech V. Country specific factors determine the quality of life among people with HIV in two western European countries.: 4th European Workshop on Health Living with HIV, Barcelona, Spain, September 2019.

2. Dovel K, Balakasi K, Shaba F, Offorjebe O, Gupta S, Wong S, Phiri K, Lungu E, Nyirenda M, **Nichols B**, Ngona K, Hoffman R. A randomized trial on index HIV self-testing for partners of ART clients in Malawi. Conference on Retroviruses and Opportunistic Infections (CROI), Seattle, USA, March 2019.

3. **Nichols BE**, Girdwood SJ*, Crompton T, Stewart-Isherwood L, Berrie L, Chimhamhiwa D, Moyo C, Kuehnle J, Rosen S. Monitoring viral load for the last mile: what will it cost? AIDS, Amsterdam, Netherlands, July 2018.

4. Girdwood SJ*, **Nichols BE**, Moyo C, Crompton T, Chimhamhiwa D, Rosen S. Optimizing access for the last mile: Geospatial cost model for point of care viral load instrument placement in Zambia. AIDS, Amsterdam, Netherlands, July 2018.

5. Dovel K, Nyirenda M, Shaba F, Offorjebe OA, Balakasi K, **Nichols BE**, Phiri K, Schooley A, Hoffman RM. Facility-based HIV self-testing for outpatients dramatically increases HIV testing in Malawi: a cluster randomized trial. AIDS, Amsterdam, Netherlands, July 2018.

6. **Nichols BE**, Hendrickson C, Sigwebela N, Moyo C, Fox MP, Rosen S. Prioritizing healthcare facilities for on-site mentorship to increase HIV treatment uptake: results from EQUIP. International AIDS Economics Network (IAEN) Conference, Amsterdam, Netherlands, July 2018.

7. van de Vijver DA, Richter A-K, Boucher CA, Gunsenheimer-Bartmeyer B, Kollan C, **Nichols BE**, Spinner C, Wasem J, Schewe K, Neumann A. Cost-effectiveness of pre-exposure prophylaxis in Germany (Kosteneffektivität der HIV-Präexpositionsprophylaxe in Deutschland). DGGÖ (German Society for health economics) Annual Meeting, Hamburg, Germay, March 2018.

8. van de Vijver DA, Richter A-K, Boucher CA, Gunsenheimer-Bartmeyer B, Kollan C, **Nichols BE**, Spinner C, Wasem J, Schewe K, Neumann A. Cost-effectiveness of pre-exposure prophylaxis for HIV-1 prevention in Germany. European AIDS Conference (EACS), Milan, Italy, October 2017.

9. Smit M, van Zoest RA, **Nichols BE**, Vaartjes I, Smit C, van der Valk M, van Sighem A, Wit FW, Hallett TB, Reiss P. Cardiovascular prevention policy in HIV: recommendations from a modeling study. Conference on Retroviruses and Opportunistic Infections (CROI), Seattle, WA. February 2017.

10. Popping S*, **Nichols BE**, van Kampen JJA, Verbon A, Boucher CAB, van de Vijver DA. Intensive hepatitis C monitoring in previously HCV infected HIV-positive MSM is a cost saving method to

reduce the HCV epidemic. Netherlands Conference on HIV Pathogenesis, Epidemiology, Prevention and Treatment (NCHIV), Amsterdam, the Netherlands, November 2016.

11. **Nichols BE**, Boucher CAB, van der Valk M, Rijnders BJA, van de Vijver DA. PrEP is Only Cost-Effective Among MSM in the Netherlands When Used on Demand. Conference on Retroviruses and Opportunistic Infections (CROI), Boston, MA. February 2016.

12. **Nichols BE**, Boucher CAB, van der Valk M, Rijnders BJA, van de Vijver DA. On demand PrEP among MSM in the Netherlands: a cost-effective approach for preventing HIV-1 infections. Netherlands Conference on HIV

Peer reviewed publications:

*Authors contributed equally

**Denotes graduate student or mentee

1. Dovel K, Nyirenda M, Shaba F, Offorjebe OA, Balakaksi K, **Nichols BE**, Cele R. Phiri K, Wong V, Gupta S, Hoffman RM. Effect of facility-based HIV self-testing on uptake of testing among adult outpatients in Malawi: a cluster-randomized trial. *The Lancet Global Health. In press.*

2. van Vliet MM**, Hendrickson C**, **Nichols BE**, Boucher CAB, Peters RPH, Polis CB, van de Vijver DAMC. Epidemiological impact and cost-effectiveness of long-acting pre-exposure prophylaxis combined with injectable contraceptives for HIV prevention in South Africa: a modelling study. *JIAS*. 2019, 22:e25427.

3. Long, L., Kuchukhidze, S., Pascoe, S., **Nichols, B.,** Cele R., Govathson, C., Flynn, D., Rosen, S. Differentiated Models of Service Delivery for Antiretroviral Treatment of HIV in sub-Saharan Africa: A Rapid Review Protocol. *Systematic Reviews*. 2019, 8:314.

4. Masuku S**, Berhanu R, van Rensburg C, Ndjeka N, Rosen S, Long L, Evans D, **Nichols BE**. The costs of managing multi drug-resistant tuberculosis in South Africa: an economic evaluation of moving to a short-course treatment regimen containing bedaquiline. *International Journal of Tuberculosis and Lung Disease. In press.*

5. Hendrickson C*,**, Long L*, van de Vijver DA, Boucher CA, O'Bra H, Claassen CW, Njelesani M, Moyo C, Mumba DB, Subedar H, Mulenga L, Rosen S, **Nichols BE**. Novel metric for evaluating PrEP program effectiveness in real-world settings. *Lancet HIV. In press.*

6. Girdwood SJ**, **Nichols BE,** Moyo C, Crompton T, Chimhamhiwa D, Rosen S. Optimizing access for the last mile: Geospatial cost model for point of care viral load instrument placement. *PLoS ONE*. 14(8):e0221586.

7. **Nichols BE**, Girdwood SJ**, Crompton T, Stewart-Isherwood L, Berrie L, Chimhamhiwa D, Moyo C, Kuehnle J, Stevens W, Rosen S. Monitoring viral load for the last mile: what will it cost? *JIAS*. 2019, 22:e25337.

8. Popping S**, **Nichols BE**, van Kampen JJA, Verbon A, Boucher CAB, van de Vijver DA. Targeted HCV core antigen monitoring among HIV-positive men-who-have-sex-with-men is cost-saving. *Journal of Virus Eradication*. 2019; 5:179-190.

For peer review only

9. **Nichols BE**, Girdwood SJ**, Shibemba A, Sikota S, Gill C, Mwananyanda L, Scott L, Noble L, Carmona S, Rosen S, Stevens W. Cost and impact of dried blood spot versus plasma separation card for viral load testing in resource limited settings. *Clinical Infectious Diseases.* Advance article: 10.1093/cid/ciz338.

10. van de Vijver DA, Richter A-K, Boucher CA, Gusenheimer-Bartmeyer B, Kollan C, **Nichols BE**, Spinner CD, Wasem J, Schewe K, Neumann A. Cost-effectiveness and budget impact of generic pre-exposure prophylaxis for HIV-1 prevention in Germany. *Eurosurveillance.* 2019 Feb; 24(7).

11. Popping S**, Hulligie SJ, Boerekamps A, Rijnders BJA, de Knegt RJ, Rockstroh JK, Verbon A, Boucher CAB, **Nichols BE**, van de Vijver DA. Early treatment of acute HCV infection is cost-effective in HIV-infected men-who-have-sex-with-men. *PLoS One*, 2019. 14(1):e0210179.

12. **Nichols BE**, Girdwood SJ**, Crompton T, Stewart-Isherwood L, Berrie L, Chimhamhiwa D, Moyo C, Kuehnle J, Stevens W, Rosen S. Impact of a borderless sample transport network for scaling up viral load monitoring: results of a geospatial optimization model for Zambia. *JIAS.* 2018, 21:e25206.

13. Dovel K, Shaba F, Nyirenda M, Ogechukwu AO, Balakasi K, Phiri K, **Nichols BE**, Tseng C-H, Bardon A, Namachapa KN, Hoffman RM. Evaluating the integration of HIV self-testing into low-resource health systems: a study protocol for a cluster randomized control trial from EQUIP Innovations. *Trials,* 2018. 19:498.

14. Phillips AN, Cambiano V, Nakagawa F, Revill P, Jordan MR, Hallett TB, Doherty M, De Luca A, Lundgren JD, Mhangara M, Apollo T, Mellors J, **Nichols B**, Parikh U, Pillay D, Rinke de Wit T, Sigaloff K, Havlir D, Kuritzkes DR, Pozniak A, van de Vijver D, Vitoria M, Wainberg MA, Raizes E, Bertagnolio S, Working Group on Modelling Potential Responses to High Levels of Pre-ART Drug Resistance in Sub-Saharan Africa. Cost-effectiveness of public-health policy options in the presence of pretreatment NNRTI drug resistance in sub-Saharan: a modelling study. *Lancet HIV*, 2018. 5(3):e146-e154.

15. Smit M, van Zoest RA, **Nichols BE**, Vaartjes I, Smit C, van der Valk M, van Sighem A, Wit FW, Hallett TB, Reiss P; Netherlands ATHENA observational HIV cohort. Cardiovascular disease prevention policy in HIV: recommendations from a modelling study. *Clinical Infectious Diseases*, 2018. 66(5):743-750.

16. Luiken GPM, Joore IK, Taselaar A, Schuit SCE, Geerlings SE, Govers A, Rood PPM, Prins JM, **Nichols BE**, Verbon A, de Vries-Sluijs TEMS. Non-targeted HIV screening in emergency departments in the Netherlands. *The Netherlands Journal of Medicine*, 2017. 75(9):386-393.

17. **Nichols BE**, Boucher CAB, van der Valk M, Rijnders BJA, van de Vijver DAMC. Cost-effectiveness analysis of pre-exposure prophylaxis for HIV-1 prevention in the Netherlands: a mathematical modelling study. *Lancet Infectious Diseases*, 2016. 16(12):1423-1429.

18. **Working Group on Modelling of ART Monitoring Strategies in Sub-Saharan Africa**. Sustainable HIV Treatment in Africa through Viral Load-Informed Differentiated Care. *Nature*, 2015. 528(7580):S68-76.

19. **Nichols BE**, Gotz HM, van Gorp ECM, Verbon A, Rokx C, Boucher CAB, van de Vijver DAMC. Partner notification for reduction of HIV-1 transmission and related costs among men who have sex with men: a mathematical modeling study. *PLoS One,* 2015. 10(11):e0142576.

For peer review only

20. **Nichols BE**, Sigaloff KC, Kityo C, Hamers RL, Baltussen R, Bertagnolio S, Jordan MR, Hallett TB, Boucher CA, Rinke de Wit TF, van de Vijver DA. Increasing the use of second-line therapy is a cost-effective approach to prevent the spread of drug-resistant HIV: a mathematical modelling study. *J Int AIDS Soc*, 2014. 17:19164.

21. **Nichols BE**, Baltussen R, van Dijk JH, Thuma PE, Nouwen JL, Boucher CA, van de Vijver DA. Cost-effectiveness of PrEP in HIV/AIDS control in Zambia: a stochastic league approach. *JAIDS*, 2014. 66(2):221-8.

22. Eaton JW, Menzies NA, Stover J, Cambiano V, Chindelevitch L, Cori A, Hontelez JAC, Humair S, Kerr CC, Klein DJ, Mishra S, Mitchell KM, **Nichols BE**, Vickerman P, Bakker R, Barnighausen T… Hallett TB. How should HIV programmes respond to evidence for the benefit of earlier treatment initiation? A combined analysis of twelve mathematical models. *Lancet Global Health*, 2014. 2:e23-34.

23. Armstrong JC*, **Nichols BE***, Wilson JM, Cosico RA, Shanks L. Spinal cord injury in the emergency context: review of program outcomes of a spinal cord injury rehabilitation program in Sri Lanka. *Conflict and Health*, 2014. 8(1):4.

24. **Nichols BE,** Sigaloff KC, Kityo C, Mandaliya K, Hamers RL, Bertagnolio S, Jordan MR, Boucher CA, Rinke de Wit TF, van de Vijver DA. Averted HIV infections due to expanded antiretroviral treatment eligibility offsets risk of transmitted drug resistance: A modeling study. *AIDS*, 2014. 28(1):73-83.

25. van de Vijver DA, **Nichols BE,** Abbas UL, Boucher CA, Cambiano V, Eaton JW, Glaubius R, Lythgoe K, Mellors J, Phillips A, Sigaloff KC, Hallett TB. Pre-Exposure Prophylaxis will have a limited impact on HIV-1 drug resistance in sub-Saharan Africa: A comparison of mathematical models. *AIDS*, 2013. 27(18):2943-2951.

For peer review only

Alemayehu Amberbir

POSITION TITLE: Epidemiologist
eRA Commons User Name: A.AMBERBIR_DI

EDUCATION/TRAINING

INSTITUTION AND LOCATION	DEGREE *(if applicable)*	YEAR(s)	FIELD OF STUDY
Haramaya University, Ethiopia	BSc	2004	Health Officer
Jimma University, Ethiopia	MPH	2007	Public Health
University of Nottingham, UK	PhD	2012	Epidemiology

Positions and Honors

Sept 2003 – Jun 2004 Intern (Clinical/Public Health), Haramaya University & Hiwot Fana Hospital, Ethiopia Oct 2004 – Sept 2005 HIV/AIDS Prevention and Care Program Officer, Menschen Für Menschen, Ethiopia
Aug 2007 - Mar 2008 Lecturer, Department of Epidemiology and Biostatistics, Jimma University, Ethiopia
Feb 2008 – Mar 2011 Honorary Lecturer, Addis Ababa University, Ethiopia
Feb 2008 – Feb 2012 Research Fellow, University of Nottingham, UK
Mar 2012 – Sep 2013 Research Fellow, London School of Hygiene and Tropical Medicine, UK
Oct 2013 – Jan 2016 Lecturer, London School of Hygiene and Tropical Medicine, UK
Jan 2016 – Jun 2019 Epidemiologist, Dignitas International
Jan 2018 – Jun 2019 Adjunct Lecturer, Dalla Lana School of Public Health, University of Toronto
Jan 2018 – Dec 2019 Postdoctoral Fellow; CIHR Canadian HIV Trials Network (CTN), Canada
Aug 2019 – present Science Director, University of California Los Angles; David Geffen School of Medicine

Contribution to Science

Investigating non-communicable diseases (hypertension, diabetes and asthma) in Africa (selected)

1. Soares ALG, Banda L, **Amberbir A**, Jaffar S, Musicha C, Price A, Nyirenda MJ, Lawlor DA, Crampin A. Sex and area differences in the association between adiposity and lipid profile in Malawi. *BMJ Glob Health*. 2019 Sep 11;4(5):e001542. doi: 10.1136/bmjgh-2019-001542.

2. **Amberbir A.** The challenge of worldwide tuberculosis control: and then came diabetes. Lancet GlobHealth. 2019 Apr;7(4):e390-e391. doi: 10.1016/S2214-109X(19)30053-1.

3. **Amberbir A**, S Lin, J Berman, et al A Muula, D Jacoby, E Wroe et al. Systematic review of hypertension and diabetes burden and risk factors and interventions for prevention and control in

Malawi: The NCD BRITE Consortium. Glob Heart. 2019 Jun;14(2):149-154. doi: 10.1016/j.gheart.2019.05.004. Review.

4. **Amberbir A**, Banda V, Singano V, Matengeni A, Pfaff C, Ismail Z, Allain TJ, Chan AK, Sodhi SK, van Oosterhout JJ. Effect of cardio-metabolic risk factors on all-cause mortality among HIV patients on antiretroviral therapy in Malawi: A prospective cohort study. ***PLoS ONE***. 2019 Jan 17;14(1):e0210629. doi: 10.1371/journal.pone.0210629. PMID: 30653539

5. **Amberbir A**, Singano V, Matengeni A, Ismail Z, Kawalazira G, Chan AK, et al. Dyslipidemia among rural and urban HIV patients in south-east Malawi. PLoS ONE 2018; 13(5): e0197728. https://doi.org/10.1371/journal.pone.0197728

6. Price AJ, Crampin AC, **Amberbir A**, et al. Prevalence of obesity, hypertension and diabetes, and cascade of care in sub-Saharan Africa: a cross-sectional population-based study in rural and urban Malawi. ***Lancet Diabetes Endocrinol 2018***; published online Jan 19. http://dx.doi.org/10.1016/S2213-8587 (17)30432-1.

7. *The GBD 2015 Obesity Collaborators et al **[inc Amberbir A].** Health Effects of Overweight and Obesity in 195 Countries over 25 Years. *N Engl J Med 2017*;377(1):13-27.

8. Divala OH, **Amberbir A**, Ismail Z, Beyene T, Garone D, Pfaff C, Singano V, Akello H, Joshua M, Nyirenda MJ, Matengeni A, Berman J, Mallewa J, Chinomba GS, Kayange N, Allain TJ, Chan AK, Sodhi SK, van Oosterhout JJ. The burden of hypertension, diabetes mellitus, and cardiovascular risk factors among adult Malawians in HIV care: consequences. *BMC Public Health*. 2016 Dec 12;16(1):1243. PMID:27955664

9. Crampin AC, Kayuni N, **Amberbir A**, Musicha C, Koole O, Tafatatha T, Branson K, Saul J, Mwaiyeghele E, Nkhwazi L, Phiri A, Price AJ, Mwagomba B, Mwansambo C, Jaffar S, Nyirenda MJ. Hypertension and Diabetes in Africa: Design and implementation of a large population-based study of burden and risk factors in rural and urban Malawi. *Emerging Themes in Epidemiology*. 2016, 13:3. DOI: 10.1186/s12982-015-0039-2

10. Jaffar S, **Amberbir A**, Kayuni N, Musicha C, Nyirenda M. Viewpoint: scaling up testing services for noncommunicable diseases in Africa: priorities for implementation research. Trop Med Int Health. 2013Nov;18(11):1353-6. doi: 10.1111/tmi.12180. Epub 2013 Sep

11. **Amberbir A**, Medhin G, Hanlon C, Britton J, Davey G, Venn A. Effects of early life paracetamol use on the incidence of allergic disease and sensitization: 5 year follow-up of an Ethiopian birth cohort. *PLoS ONE*. **2014** Apr 9;9(4):e93869. doi: 10.1371/journal.pone.0093869.

12. **Amberbir A**, Medhin G, Abegaz WE, Hanlon C, Robinson K, Fogarty A, Britton J, Venn A, Davey G. Exposure to Helicobacter pylori infection in early childhood and the risk of allergic disease and atopic sensitization: a longitudinal birth cohort study. Clin Exp Allergy. 2014 Apr;44(4):563-71. doi:

13. **Amberbir A**, Medhin G, Erku W, Alem A, Simms R, Robinson K, Fogarty A, Britton J, Venn A, Davey G. Effects of Helicobacter pylori, geohelminth infection and selected commensal bacteria on the risk of allergic disease and sensitization in 3-year-old Ethiopian children. *Clin Exp Allergy*. **2011** Oct;41(10):1422-30. doi: 10.1111/j.1365-2222.2011.03831.x. Epub 2011 Aug 10.

For peer review only

14. **Amberbir A**, Medhin G, Alem A, Britton J, Davey G, Venn A. The role of acetaminophen and geohelminth infection on the incidence of wheeze and eczema: a longitudinal birth-cohort study. *Am J Respir Crit Care Med*. **2011** Jan 15;183(2):165-70. doi: 10.1164/rccm.201006-0989OC. Epub 2010 Oct 8.

HIV risk behaviours and status disclosure in African settings (selected)

1) Dessalegn NG, Hailemichael RG, Shewa-Amare A, Sawleshwarkar S, Lodebo B, **Amberbir A**, Hillman RJ. HIV Disclosure: HIV-positive status disclosure to sexual partners among individuals receiving HIV care in Addis Ababa, Ethiopia. PLoS One**. 2019** Feb 15;14(2):e0211967. doi: 10.1371/journal.pone.0211967. eCollection 2019.

2) Deribe K, Woldemichael K, Wondafrash M, Haile A, **Amberbir A**. Disclosure experience and associated factors among HIV positive men and women clinical service users in Southwest Ethiopia. *BMC Public Health*. 2008 Feb 29;8:81. doi: 10.1186/1471-2458-8-81.

3) Biadgilign S, Deribew A, **Amberbir A**, Escudero HR, Deribe K. Factors associated with HIV/AIDS diagnostic disclosure to HIV infected children receiving HAART: a multi-center study in Addis Ababa, Ethiopia. *PLoS ONE*. 2011 Mar 21;6(3):e17572. doi: 10.1371/journal.pone.0017572.

Health system research in to infectious diseases – HIV – in Africa (selected)

1. Alhaj M[1,2¶],**Amberbir A**[1¶], Singogo E, Banda V, van Lettow M, Matengeni A, Kawalazira G, Theu J, Jagriti MR, Chan AK, van Oosterhout JJ. Retention on antiretroviral therapy during universal test and treat implementation in Zomba district, Malawi: a retrospective cohort study. J Int AIDS Soc. 2019 Feb;22(2):e25239. doi: 10.1002/jia2.25239.

2. Singano V[¶], **Amberbir A**[¶], Garone D, Kandionamaso C, Msonko J; van Lettow M, Kalima K, Mataka, Kawalazira G, Mateyu G, Kwekwesa A, Matengeni A; van Oosterhout JJ. The burden of gynecomastia among men on Antiretroviral Therapy in Zomba, Malawi. *PLoS ONE 12(11): e0188379: doi.org/10.1371/journal.pone.0188379*

3. Mpawa H, Kwekwesa A, **Amberbir A**, Garone D, Divala OH, Kawalazira G, van Schoor V , Ndindi H, van Oosterhout JJ. Virological outcomes of Antiretroviral Therapy in Zomba Central Prison, Malawi: *J Int AIDS Soc* 2017, 20:21623

4. **Amberbir A**, Woldemichael K, Getachew S, Girma B, Deribe K. Predictors of adherence to antiretroviral therapy among HIV-infected persons: a prospective study in Southwest Ethiopia. *BMC Public Health***. 2008** Jul 30;8:265. doi: 10.1186/1471-2458-8-265.

5. Biadgilign S, Deribew A, **Amberbir A**, Deribe K. Barriers and facilitators to antiretroviral medication adherence among HIV-infected paediatric patients in Ethiopia: A qualitative study. *SAHARA J***.** 2009 Dec;6(4):148-54.

For peer review only

6. Deribe K, Hailekiros F, Biadgilign S, **Amberbir A**, Beyene BK. Defaulters from antiretroviral treatment in Jimma University Specialized Hospital, Southwest Ethiopia. *Trop Med Int Health*. 2008 Mar;13(3):328-33. doi: 10.1111/j.1365-3156.2008.02006.x. Epub 2008 Feb 21.

For peer review only

LAWRENCE C. LONG, PHD, MCOM

OFFICE ADDRESS: 801 Massachusetts Avenue, Crosstown Center, 3rd Floor, Room 369
Boston, MA, 02119, USA.
+1 (617) 358-3122
Email: lclong@bu.edu

ACADEMIC TRAINING:

2016	Ph.D.	School of Public Health, University of Witwatersrand (Johannesburg, South Africa)
2009	M.Com.	School of Economic and Business Sciences, University of Witwatersrand (Johannesburg, South Africa), Economics, *cum laude*
2001	B.Bus.Sci.	School of Economics, University of Cape Town (Cape Town, South Africa), Economics and Finance

ACADEMIC LEADERSHIP:

2011 - 2017	Deputy Division Head	Health Economics & Epidemiology Research Office<br>Wits Health Consortium<br>Faculty of Health Sciences, University of Witwatersrand
2008 – 2011	Research Coordinator	Health Economics & Epidemiology Research Office<br>Wits Health Consortium<br>Faculty of Health Sciences, University of Witwatersrand

RESEARCH APPOINTMENTS:

2017 – Present	Research Assistant Professor	Department of Global Health, School of Public Health,<br>Boston University
2008 – Present	Associate Researcher	Joint Faculty Appointment, School of Clinical Medicine,<br>Faculty of Health Sciences, University of the Witwatersrand
2008 – Present	Principal Researcher (Visiting)	Health Economics & Epidemiology Research Office<br>Wits Health Consortium<br>Faculty of Health Sciences, University of

2006 – 2007	Research Associate	Witwatersrand<br>Health Economics & Epidemiology Research Office<br>Wits Health Consortium<br>Faculty of Health Sciences, University of Witwatersrand
2005	Study Coordinator	Health Economics & Epidemiology Research Office<br>Wits Health Consortium<br>Faculty of Health Sciences, University of Witwatersrand

OTHER RESEARCH EXPERIENCE:

2007 – 2008	Consultant	Center for International Health and Development,<br>Boston University School of Public Health<br>Project: *Cost and Outcomes of Models for Delivering Antiretroviral Therapy for HIV/AIDS in Zambia*
2007 – 2008	Consultant	Center for International Health and Development,<br>Boston University School of Public Health<br>Project: *Cost and Outcomes of Models for Delivering Antiretroviral Therapy for HIV/AIDS in Kenya*

TEACHING EXPERIENCE:

2019	**Structural factors affecting HIV – an economists perspective.** Audience: MPH Students. Guest Lecture in Individual, Community, and Population Health SPH GH 720 Boston University, Boston, USA. Presented in two classes (Profs Monica Onyango & Jennifer Schlezinger).
2019	**Economic evaluation.** Audience: MPH Students. Guest Lecture in Monitoring and Evaluation of Global Health Programs SPH GH 745, Boston University, Boston, USA.
2019	**Using economics to influence policy.** Audience: MPH Students. Guest Lecture in Essential of Economics and Finance for Global Health SPH GH 762, Boston University, Boston, USA.
2017	**From cost to clinic – Economics changing health policy.** Audience: MPH Students. Guest Lecture in Essentials of Economics and Finance for Global Health SPH GH 762, Boston University, Boston, USA.
2016	**Data collection and analysis for economic evaluations.** Audience: Technical implementing partners. EQUIP Partners Meeting, Johannesburg, South Africa.

For peer review only

2016 **From patient to policy – Ensuring that your clinical practice is positioned to inform evidenced based policy.** Audience: HIV Clinicians. Chair of Research Skills Building Session, Southern African HIV Clinicians Society Conference, Johannesburg, South Africa.

2014 **Introduction to Health Programme Evaluation**. Audience: MSc students – Module of Epidemiology for Health Researchers II. University of Witwatersrand, Johannesburg.

INVITED PRESENTATION:

2019 **Costs and resources needed to provide HIV services to key populations over the next 10 years.** Idea creation meeting – Defining and addressing HIV treatment and prevention needs of underserved and high-risk populations. BMGF & Journal of International AIDS Society, New York, USA.

2019 **Direct action to achieve a result.** Spotlight on "Think, Teach, Do", School of Public Health, Boston University, Boston, USA.

2019 **Learning Community – Take home.** Academy for Faculty Advancement, School of Medicine, Boston University, Boston, USA.

2018 **Urban public health issues – the transition to Boston.** Health Economics and Epidemiology Research Office, Johannesburg, South Africa.

2017 **Partner's Area of Expertise – Health Economics.** USAID South Africa, Partners Meeting, Pretoria, South Africa. Presented in absentia by Denise Evans.

2017 **Innovations Research on AIDS (INROADS).** Director Doug Arbuckle, Office HIV AIDS (OHA, USA). USAID, Johannesburg, South Africa. Presented in absentia by Denise Evans.

2016 **Test and Start – Research supporting evidence based policy change.** Zambian Department of Health & USAID, EQUIP Project, Johannesburg, South Africa.

2016 **Initiating ART at a patients first clinic visit: the RapIT randomised trial.** Faculty of Health Sciences Research Day, University of Witwatersrand, Johannesburg, South Africa.

CONFERENCE ORAL PRESENTATIONS:

1. Van Rensburg C, Berhanu R, Hirasen K, Evans D, Rosen S, **Long L**. Cost outcome analysis of decentralised care for drug-resistant tuberculosis in Johannesburg, South Africa. 49th Union World Conference on Lung Health, 24-27 October, The Hague, The Netherlands. 2018.

For peer review only

2. Evans D, Musakwa N, Nattey C, Bor J, Lonnermark E, Larshans C, Andreasson S, Nyasulu P, **Long L**. Barriers to accessing care for HIV and Tuberculosis among adolescents and young adults aged 18 – 25 years in Johannesburg, South Africa. 5th SA TB Conference, 12-15 June, Durban, South Africa. 2018.

3. Lince-Deroche N, Leuner R, **Long L**. When Donor Funding Leaves: The immediate impact on resources of withdrawal of support for direct HIV care and treatment at public health facilities in Johannesburg. 9th International AIDS Society (IAS) Conference, 23-26 July, Paris, France. 2017.

4. Musakwa N, Evans D, Feeley A, Magwete M, Patz S, McNamara L, **Long L**, Sanne I. Acute malnutrition and dietary intake among paediatric HIV-positive patients initiating antiretroviral therapy in Johannesburg, South Africa. 8th SA AIDS Conference, 13-15 June, Durban, South Africa. 2017.

5. Rosen S, Maskew M, Fox MP, Nyoni C, Mongwenyana C, Malete G, Sanne I, Bokaba D, Sauls C, Rohr J, **Long L**. Initiating ART at a Patient's First Clinic Visit: The RapIT Randomized Trial. Conference on Retroviruses and Opportunistic Infections (CROI) 2016, 22-25 February, Boston, USA.

6. Hirasen K, Berhanu R, Schnippel K, **Long L**, Rosen S, Sanne I. Twelve-month outcomes of patients initiating drug-resistant tuberculosis treatment at a decentralized outpatient clinic in Johannesburg, South Africa. 46th World Conference on Lung Health of the International Union Against Tuberculosis and Lung Disease (The Union), Cape Town, South Africa, 2-6 December 2015.

7. Evans D, Schnippel K, Budgell E, Shearer K, Berhanu R, **Long L**, Rosen S. Predictors of mortality in patients diagnosed with preXDR- and XDR-TB: results from the South African National TB Programme, 2009-2010. 46th World Conference on Lung Health of the International Union Against Tuberculosis and Lung Disease (The Union), Cape Town, South Africa, 2-6 December 2015.

BIBLIOGRAPHY:

ORCID ID: 0000-0003-4986-4988

Google Scholar: https://scholar.google.co.za/citations?user=fao8zDgAAAAJ&hl=en&oi=ao

Peer reviewed publications:

1. **Long L**, Kuchukhidze S, Pascoe S, Nichols B, Cele R, Govathson C, Huber A, Flynn D, Rosen S. Differentiated models of service delivery for antiretroviral treatment of HIV in sub-Saharan Africa: a rapid review protocol. *Systematic Reviews*. DOI: 10.1186/s13643-019-1210-6. 2019.

For peer review only

2. Jamieson L, Evans D, Berhanu R, Ismail N, Aucock S, Wallengren K, **Long L**. Data quality of drug-resistant tuberculosis and antiretroviral therapy electronic registers in South Africa. *BMC Public Health*. DOI: 10.1186/s12889-019-7965-9. 2019.

3. Mokhele I, Mashamaite S, Majuba P, Xulu T, **Long L**, Onoya D. Effective public-private partnerships for sustainable antiretroviral therapy: outcomes of the Right to Care health services GP down-referral program. *BMC Public Health*. DOI:
10.1186/s12889-019-7660-x. 2019.

4. Lince-Deroche N, Leuner R, Meyer-Rath G, Pillay Y, **Long L**. When Donor Funding Leaves: A retrospective review of the impact of integrating direct HIV care and treatment into public health services in a region of Johannesburg. *Cost Effectiveness and Resource Allocation*. DOI: 10.1186/s12962-019-0192-5. 2019.

5. Musakwa N, Feeley A, Magwete M, Patz S, McNamara L, Sanne I, **Long L**, Evans D. Dietary intake among paediatric HIV-positive patients initiating antiretroviral therapy in Johannesburg, South Africa. *Vulnerable Children and Youth Studies (RVCH)*. DOI: 10.1080/17450128.2019.166858. 2019.

6. Brennan A, Bonawitz R, Gill CJ, Thea DM, Kleinman M, **Long L**, McCallum C, Fox MP. A Meta-analysis Assessing Diarrhea and Pneumonia in HIV-Exposed Uninfected Compared With HIV-Unexposed Uninfected Infants and Children. *Journal of Acquired Immune Deficiency Syndrome (JAIDS)*. DOI: 10.1097/QAI.0000000000002097. 2019

7. Berry KM, Rodriguez CA, Berhanu R, Ismail N, Rosen S, **Long L**, Evans D. Treatment outcomes among pediatrics, adolescents, and adults on treatment for drug-sensitive TB in two metropolitan municipalities in Gauteng Province, South Africa. *BMC Public Health*. DOI: 10.1186/s12889-019-7257-4. 2019.

For peer review only

IN-DEPTH INTERVIEW FOR MEN – MIDLINE

Section 1: Pre-Trial Experiences

1. At some point in the past, you stopped taking ARV's (or didn't start ART), which is why you were recruited into this study. Can you walk me through some of the reasons that it was difficult to stay on (or start) ART in the past?

Section 2: Trial Experience

1. As a part of your participation in this study, you may have been visited a couple of times by different people. Did anything **bad** happen as a result of these visits?
2. Did someone discover your HIV status as a result of the study or as a result of taking ART since you enrolled?
3. How many times have you met with a health care worker (Patient Supporter/Nurse) about HIV (excluding ART clinic visits)?
   1. *If yes:* Where did you meet this person?
4. When this health care workers (Patient Supporter/Nurse) met with you, you may have gone through this Men's Counseling Flip Chart [SHOW THE FLIP CHART].
   1. Have you ever seen this flip chart?
      i. IF YES: How was the counseling from this flip chart different from the counseling you have received in the past (before joining the study)?
      ii. IF YES: What topic/idea from the flip chart was the most helpful to you?
      iii. IF YES: You described to me earlier that you have missed ART in the past because of [X] reason. Did the new counseling with this flip chart help you deal with [X]? How?
      iv. IF NO: When you met with the health care worker, can you briefly describe what you discussed?
5. As a part of the study. you may have been offered to be given ART in the home/community.
   1. Did you decide to get ART at home/in the community? *If NO: Skip to Question 6.*
   2. Why did you make the choice you made?
   3. Usually health care workers don't give people a choice of what service you get. But for you, you were given the option between home or facility. How did having this choice (having a say) make you feel?
6. How do you currently get ARTs?
   1. IF at HOME/COMMUNITY: How many times have you gotten your medication at home?
   2. If at the FACILITY: When you went back to the facility, did the did a health care worker/patient supporter offer you any other services?
      i. What did they do?
      ii. Was this helpful for you? Why/why not?
7. Has either HCW been supporting you or chatting with you about anything else in addition to HIV or ART?
   1. Do you ever contact either HCW directly to chat or ask questions?
      i. IF YES: How often do you talk with them? What do you talk about?
   2. Is your relationship with either HCW different to the ones you have had with other HCWs before joing the study? How?
      i. IF YES: Do you think this relationship helps you more than your relationship with other HCWs? IF YES: How?
8. Now think about overall your interactions with the health care workers you interacted with since you started the study – the Patient Supporter and the Nurse who brought you ART. What did you like about your intereactions with them? How do you think it helps you with ART?

For peer review only - http://bmjopen.bmj.com/site/about/guidelines.xhtml

9. With everything in life, there are some things we like and some things that could be a little better. What did you NOT like about your interactions with the Patient Supporter and the Nurse who brought you ART?
10. Is there anything else you think you need a health care worker or the health facility could do differently in order for you to be comfortable taking ART regularly? Is there anything else you need?

Section 3: Initiation
Now I'd like to ask you about what has happened with your ARVs since you were enrolled in the study.

11. Did you start taking ART (again) since enrolling in the study?
    If YES:
    a. Why did you (re) initiate ART? What convinced you it was good to take medication (again)?
    b. Who was most influential in your decision to start ART (again)?
    If NO:
    c. Why have you not initiated ART since enrolling in the study?
    d. Has anyone influenced you to not start ART? Who? Why?
    e. Is there anything that could motivate/help you to initiate ART?
12. We know that starting ART can be difficult. What do you think is the most difficult thing about re-starting ART for you
13. Was there anything that made it easy for you to (re)start ART? (opposite of probes below)
    1. For those who opted for HOME-BASED: Do you think getting ART at home helped you re-engage in care? Why/why not?
14. For the next set of questions please feel free to be honest. There is no right or wrong answer.
    1. Do you feel MORE confident you can stay on ARTs in the future? Why/why not?
    2. Is there someone in your life now that encourages you?
    3. Do you have a better relationship with HCWs / or trust them more? Why/why not?
    4. Do you have a plan so that you don't run out of ARVs if youre away or busy?
    5. Do you think ARVs help you reach your goals for making money and for your family? Why/why not?

IN-DEPTH INTERVIEW FOR MEN – ENDLINE

Section 1: Pre-Trial Experiences

1. Think about when you stopped taking ART **before** joining the study. Can you remember why you stopped swallowing ART pills?
2. Talk to me about some things that made it difficult for YOU to stay on (or start) ART BEFORE joining the study?

Section 2: Post-Trial Experiences

3. As a part of the study, you received [ONLY MENTION THE SERVICE THAT ALIGNS WITH THE CLIENTS STUDY ARM: counseling, mentorship, counseling + home-based 1 month, counseling + home-based 3 months].
    a. How did you feel about this service?
    b. Was this helpful to you? How?
4. Since you enrolled in the study did you restart ARVS?
    a. IF NEVER RE-INITIATED ART IN THE STUDY: Why have you not re-started ART since joining the study?

For peer review only

5. Think about the challenges you experienced BEFORE joining the study. [REFER BACK TO THE CHALLENGES MENTIONED IN QUESTION 1 and 2]. Have these challenges gone away or been reduced since you joined the study?
   a. How?
   b. Why has it changed/not changed?

6. Have you had any **new or different** challenges since joining the study (either to attend refill appointments or taking your ART)? What are they?
   a. Why do you think you experience them now?

7. Are there things about attending refill appointments or swallowing ART that are now easier since you joined the study?
   a. What are they? Why?

8. Think about what you discussed with the study HCW who met with you [he may or may not have used this flip chart]. When he chatted with you, he might have told you some things that were **new** to you about how to keep taking ARTs. Have you tried doing any of those things AFTER you met with the HCW?
   a. What was it?
   b. Did it help you? How?
   c. If it did not help you, what challenges did you face?

9. Have you continued to talk to the study HCW since you first met with him?
   a. Describe your relationship.
      i. How frequently do you connect? In person, on the phone, both?
      ii. What do you discuss?
      iii. How is this relationship helpful for you and your life?
      iv. Do you wish for your relationship with the HCW to continue [or continued in the case of the HCW being fired]? Why? Why not?

10. [GENERAL NOT JUST STUDY HCWS] What additional services/interventions [not materials/monitary] do you wish you could have to help you consistently access or swallow ART?
   a. Would you need this for a short period of time (one time, or for a month) or would you need it ALL the time (for years)? Why?

11. Do you have other health concerns outside of HIV? What are they?
   a. Is it easier or harder for you to get care for these concerns as compared to getting ART services? Why?

Section 3: Repeat Treatment Interruption [ONLY FOR THOSE WHO REINTIATED]
Now I want to talk to you about your use of ART services since joining the study.

12. Have you missed an ART appointment or missed swallowing your medication on any day since you restarted ART?

If **NO:**

13. How have you managed to stay on ART? What strategies do you think are most useful to help you stay on ART? Are they different from the one's you mentioned before? Does someone or something specifically help you?

If **YES:**

14. Can you tell me what happened?
   a. For how long did you stop taking ART?
   b. How many times has it happened?
   c. Why did you miss ART appointment/swallowing ART?

15. What do you think would make it easier for men like you to not miss doses of ART or ART appointments?

16. Have you started taking ARVS **again** after you missed doses or gone back to the facility after you missed a refill appointment?

If **NO** – skip to question 18

If **YES**

17. Did anything made it easy for you to (re)start ART AGAIN?

For peer review only - http://bmjopen.bmj.com/site/about/guidelines.xhtml

18. We know that restarting ART can be difficult. What fears/worries/concerns did you have to overcome in order to restart (again)?

19. What would make it easier for men like you to come back to the clinic after missing an appointment?

Section 4: Client centered care choice experiment

20. We know there is no such thing as a 'perfect' healthcare worker. We are all human. But today we want to give you the opportunity to think about key things you want if you had the perfect interaction with a health care worker. Here is a picture of a health care worker. Around him we have characteristics on different cards [McDaphton to read through the cards]

1. He treats me with respect
2. His counseling message is easy to understand
3. His counseling addresses my specific concerns
4. He maintains privacy/keeps my secrets
5. He keeps in contact with me (not just a one time counseling)
6. He comes to the community to find me
7. He treats me like family and cares for me as a person
8. He asks me questions about my life/circumstances
9. He takes his time with me (the counseling session is not rushed)
10. He shares his own experiences openly as a fellow man
11. He provides fast services

21. Id like you to choose the 6 most important characteristics you think are essential for how you want to interact with health care workers. There is no right or wrong answers. This is about what matters to you.

a. Why have you chosen these? What makes them very important to you?

22. Now you can only choose 3 of these characteristics. Look at your 6 characteristics in front of you. What THREE are the most important/essential characteristics for your health care worker to still provide you with the services you would like.

a. Why have you chosen these? Why did you NOT choose the other three?

Section 5: Income

23. What things do you do to earn a living? Think about all the work you do – this may include several different things.

a. How do you earn money (what kind of work do you do)?
b. Where do you work?
c. Do you travel for work?
d. How often do you work?
e. How do you usually find this work? How do employers/bosses or customers find you or how do you find markets for your products?

24. What would happen to you and/or your family if you spent a whole day at the clinic and did not earn any money that day?

a. Is it possible for you to attend your ART appoint and not miss out on any income/money? How is this possible for you?
b. In the WORST case scenario: how much missed opportunities to earn money would you experience? What would be the biggest impact on your financial situation be if you attend a clinic appointment and miss work/work opportunities (exp: do you miss a client, did your shop not open, did you miss the chance to get a long term job?)
c. Does missing a day from making money impact your family? How?

For peer review only - http://bmjopen.bmj.com/site/about/guidelines.xhtml
